# Genome-Scale CRISPR Knockout Screening Identifies BACH1 as a Key Regulator of Aflatoxin B_1_-Induced Oxidative Damage

**DOI:** 10.3390/antiox11091787

**Published:** 2022-09-10

**Authors:** Jinfu Zhang, Siyi Hu, Changzhi Zhao, Yuan Zhou, Lu Zhang, Hailong Liu, Peng Zhou, Sheng Li, Liangliang Fu, Zhuqing Zheng, Yue Xiang, Xuewen Xu, Jinxue Ruan, Xinyun Li, Lvhui Sun, Gang Cao, Shuhong Zhao, Xu Wang, Shengsong Xie

**Affiliations:** 1 Key Laboratory of Agricultural Animal Genetics, Breeding and Reproduction of Ministry of Education & Key Lab of Swine Genetics and Breeding of Ministry of Agriculture and Rural Affairs, Huazhong Agricultural University, Wuhan 430070, China; jinfuzh@webmail.hzau.edu.cn (J.Z.); czzhao@mail.hzau.edu.cn (C.Z.); 7251314520@webmail.hzau.edu.cn (Y.Z.); luzhang@webmail.hzau.edu.cn (L.Z.); hailongliu@webmail.hzau.edu.cn (H.L.); zhou-peng@webmail.hzau.edu.cn (P.Z.); lisheng@webmail.hzau.edu.cn (S.L.); fuliangliang2017@hzau.edu.cn (L.F.); zzq_1207@mail.hzau.edu.cn (Z.Z.); yxiang@webmail.hzau.edu.cn (Y.X.); xuewen_xu@mail.hzau.edu.cn (X.X.); ruanjinxue@mail.hzau.edu.cn (J.R.); xyli@mail.hzau.edu.cn (X.L.); 2 Guangdong Laboratory of Lingnan Modern Agriculture, Guangzhou 510642, China; 3 National Reference Laboratory of Veterinary Drug Residues (HZAU) and MAO Key Laboratory for Detection of Veterinary Drug Residues, Huazhong Agricultural University, Wuhan 430070, China; 2019302110084_hsy@webmail.hzau.edu.cn; 4 Hubei Hongshan Laboratory, Huazhong Agricultural University, Wuhan 430070, China; lvhuisun@mail.hzau.edu.cn (L.S.); gcao@mail.hzau.edu.cn (G.C.); 5 The Cooperative Innovation Center for Sustainable Pig Production, Huazhong Agricultural University, Wuhan 430070, China

**Keywords:** AFB_1_, BACH1, CRISPR screening, inhibitor

## Abstract

Aflatoxin B_1_ (AFB_1_) is amongst the mycotoxins commonly affecting human and animal health, raising global food safety and control concerns. The mechanisms underlying AFB_1_ toxicity are poorly understood. Moreover, antidotes against AFB_1_ are lacking. Genome-wide CRISPR/Cas9 knockout screening in porcine kidney cells identified the transcription factor BTB and CNC homolog 1 (BACH1) as a gene required for AFB_1_ toxicity. The inhibition of BACH1 expression in porcine kidney cells and human hepatoma cells resulted in increased resistance to AFB_1_. BACH1 depletion attenuates AFB_1_-induced oxidative damage via the upregulation of antioxidant genes. Subsequently, virtual structural screening identified the small molecule 1-Piperazineethanol, α-[(1,3-benzodioxol-5-yloxy)methyl] -4-(2-methoxyphenyl) (M2) as an inhibitor of BACH1. M2 and its analogues inhibited AFB_1_-induced porcine and human cell death in vitro, while M2 administration significantly improved AFB_1_-induced symptoms of weight loss and liver injury in vivo. These findings demonstrate that BACH1 plays a central role in AFB_1_-induced oxidative damage by regulating antioxidant gene expression. We also present a potent candidate small-molecule inhibitor in developing novel treatments for AFB_1_ toxicity.

## 1. Introduction

Aflatoxins are secondary metabolites produced mainly by the fungi *Aspergillus flavus* and *Aspergillus parasiticus,* four of which are known to occur naturally, including aflatoxin B_1_ (AFB_1_), B_2_ (AFB_2_), G_1_ (AFG_1_), and G_2_ (AFG_2_) [1,2]. AFB_1_ exhibits the highest toxicity and is an unavoidable contaminant in foods and livestock feeds, endangering human and animal health [3]. For instance, AFB_1_ is highly carcinogenic and can contribute to the development of hepatocellular carcinoma (HCC) [4]. The toxic effects of AFB_1_ are both dose- and time-dependent, and aflatoxicosis can be either acute or chronic. Exposure to AFB_1_ affects multiple organs like kidneys, liver, heart, testes, and ovaries, causing mutagenesis, growth retardation, and reproductive damage [5,6]. Moreover, AFB_1_ toxicity significantly suppresses the immune system, increasing the risk of acquiring concurrent infectious diseases [7].

In addition to the AFB_1_-associated effects in humans, severe health issues such as hepatotoxicity, teratogenicity, and immunotoxicity occur in animals [6,8,9]. Pigs exposed to aflatoxin-contaminated feed are highly susceptible to secondary infections [10]. AFB_1_ can also negatively impact meat quality due to associated oxidative stress, inflammation, and the dysregulation of gut microbiota and dysbiosis in animals [11,12]. Various physical, chemical, and biological approaches to preventing AFB_1_ contamination have been developed [13,14,15]. For instance, transgenic pigs expressing aflatoxin-detoxifizyme (ADTZ) exhibit resistance to AFB_1_ toxicity [16]. Nevertheless, economic losses and threats to public health caused by AFB_1_ contamination persist [13,17]. Thus, further efforts to identify the molecular mechanisms underlying AFB_1_ toxicity are necessary for reducing aflatoxicosis and decreasing the risk to animal and human health.

AFB_1_ requires metabolic activation (biotransformation) by cytochrome P450 enzymes to generate AFB_1_-exo-8,9-epoxide (AFBO), which forms adducts through binding with DNA, RNA, and proteins [18]. The detoxification of AFBO mainly occurs through conjugation with glutathione (GSH) mediated by glutathione S-transferase (GST), a phase II conjugative (detoxification) enzyme [19]. In addition, except for AFBO, animals can metabolize aflatoxin into relatively less toxic intermediates or metabolites, such as aflatoxin M_1_ (AFM_1_) [20]. Previous studies have revealed that AFB_1_ can induce the overexpression of *CYP1A1* and *CYP1A2* genes, accompanied by the increased transcription of Aryl hydrocarbon receptor (AhR), which was linked to AFB_1_-related toxicity in a recent genome-wide loss-of-function screening [21,22]. Another study identified cyclooxygenase-2 (COX-2) as a factor in AFB_1_-induced mitophagy and lipid accumulation [21,22]. It has also been shown that caveolin-1 plays an essential role in the AFB_1_-induced apoptosis of hepatic cells via the regulation of oxidation and autophagy [23]. Nonetheless, the mechanisms involved in AFB_1_ toxicity remain poorly understood.

In this study, we adopted a high-throughput forward screening strategy to identify the host factors required for AFB_1_-induced cell death using our previously designed porcine genome-scale CRISPR/Cas9 knockout (PigGeCKO) library [24]. We found that the transcription factor BACH1 was paramount in AFB_1_ cytotoxicity. Knockout (KO) of *BACH1* could inhibit AFB_1_-induced oxidative damage through the transcriptional upregulation of antioxidant genes. Moreover, structure-based virtual screening led to the identification of several potential small-molecule inhibitors of BACH1 and confirmed the effectiveness of 1-Piperazineethanol, α-[(1,3-benzodioxol-5-yloxy)methyl] -4-(2-methoxyphenyl) (hereinafter called M2) as an inhibitor both in vitro and in vivo. Accordingly, BACH1 is a potential drug target for AFB_1_-induced oxidative damage. Moreover, M2 can serve as an efficient inhibitor of AFB_1_.

## 2. Materials and Methods

### 2.1. Plasmid Construction

Lentivirus small-guide RNA (sgRNA) expression vectors were generated by digesting the lenti-sgRNA-EGFP and LentiCRISPRv2 plasmids (Addgene #52961) using the *Bbs*I and *BsmB*I restriction enzymes, respectively. The paired sgRNA oligonucleotides were annealed and cloned into the linearized vector. These sgRNAs were designed by sgRNAcas9-AI (http://123.57.239.141:8080/home, accessed on 6 December 2019). The sgRNA sequences are listed in Table S1.

### 2.2. Cell Culture and Transfection

The porcine Kidney-15 (PK-15), human embryonic kidney 293T (HEK293T), and Huh7 cell lines were purchased from the Cell Bank of the Chinese Academy of Sciences (Shanghai, China). These cell lines were tested for mycoplasma and maintained at 37 °C with 5% CO_2_ in Dulbecco’s Modified Eagle Medium (DMEM) (Cat No: C11995500BT, Gibco, Beijing, China) supplemented with 10% fetal bovine serum (FBS) (Cat No: 10270-106, Gibco), 100 U/mL penicillin and 100 μg/mL streptomycin (Cat No: 15140122). The JetPrime reagent (Cat No: B180306, PolyPlus) was used for transfection according to the manufacturers protocol.

### 2.3. Genome-Wide CRISPR/Cas9 Screening

Genome-wide CRISPR/Cas9-based screening in PK-15 cells was conducted using the PigGeCKO lentiviral sgRNA library as previously described [24]. The PigGeCKO library contains 85,674 single-guide RNAs targeting 17,743 protein-coding genes, 11,053 long ncRNAs, and 551 miRNAs. A total of ~2 × 10^8^ PK-15 cells stably expressing Cas9 (hereafter referred to as PK-15-Cas9) were seeded into T225 flasks and infected with the sgRNA lentiviral library at a multiplicity of infection (MOI) of 0.3. Three days post-infection, GFP-positive cells were collected by fluorescence-activated cell sorting (FACS), reseeded into 100 mm dishes, and preserved and the coverage was examined of the mutant cell libraries. For the CRISPR screening, a total of ~1 × 10^8^ mutant cells were exposed to AFB_1_ (Cat No: 1162-65-8) at 0.2 μg/mL, and the toxin was refreshed daily until all the PK-15-Cas9 (Control) died. The surviving mutant cells were then exposed to two additional rounds of AFB1 at different concentrations (Round 2 at 1 μg/mL and round 3 at 6 μg/mL). Genomic DNA was isolated from ~1 × 10^6^ surviving cells from each round of exposure, integrated sgRNA sequences obtained by polymerase chain reaction (PCR) and decoded by Illumina-based sequencing. The remaining surviving mutant cells were cultured in medium containing 15 μg/mL H_2_O_2_ until all the control cells died. The surviving cells were collected for genomic DNA isolation and the identification of sgRNAs by Illumina-based sequencing.

### 2.4. Generation of PK-15 and Huh7 KO Cell Lines by CRISPR/Cas9 Technology

The sgRNA sequence targeting the porcine *BACH1* was cloned into the linearized lenti-sgRNA-EGFP vector, and lentivirus was assembled to infect PK-15-Cas9 cells. GFP-expressing cells were enriched by FACS on the 3rd day post-infection and seeded in 96-well plates to generate clonal KO cells. Approximately 7 days after infection, the genotypes of the cell colonies were analyzed by extracting genomic DNA using the TIANamp Genomic DNA Kit (Cat No: DP304, TIANGEN, Beijing, China), and the nucleotide sequences were revealed by Sanger sequencing (Tsingke, Wuhan, China). Mutant colonies identified from the sequences were characterized by homozygous TA clone sequencing. The sgRNA sequence targeting porcine *BACH1* was also cloned into the linearized lentiCRISPRv2 and generated the PK-15 mutant colonies without a green fluorescent protein (GFP) label. The sgRNA targeting the human *BACH1* gene was cloned into the linearized lentiCRISPRv2, and lentivirus was assembled to infect the Huh7 cells. On the 3rd day post-infection, infected cells were selected using 2.5 μg/mL puromycin to generate the human *BACH1* KO pooled cells. The Sanger sequencing primers used are shown in Table S1.

### 2.5. Cell Counting Kit-8 (CCK-8) Viability Assay

Approximately 1 × 10^4^ cells/well were seeded in 96-well flat-bottomed plates (Cat No: 701001, NEST, Wuxi, China) and incubated at 37 °C + 5% CO_2_ for 24 hours (h) to obtain 50–60% confluence. The AFB_1_ and other inhibitors were added to all the test sample cells except the control group and incubated for 36 h. The CCK-8 stock solution (Cat No: KGA317, KeyGEN, Jiangsu, China) was diluted 1:10 in unsupplemented DMEM to make the CCK-8 working solution. 100 μL of the CCK-8 working solution was added to each well, and the plate was incubated for 1 h at 37 °C. Absorbance at 450 nm was measured using a microplate reader (EnVision, PerkinElmer, Waltham, MA, USA). Absolute (100%) viability was defined as the absorbance of the control groups.

### 2.6. Flow Cytometry Analysis for Reactive Oxygen Species

Approximately 1 × 10^5^ cells/well were seeded in 12-well plates for 24 h to obtain 50–60% confluence. The wells were treated with 0.1 μg/mL AFB_1_, and the plates were incubated at 37 °C for 36 h. The cell monolayer was detached using 0.25% trypsin without EDTA (Cat No: 15050-065, Gibco) and resuspended in supplemented DMEM. Reactive oxygen species (ROS) were detected by adding the ROS detection green fluorescent probe reagent (Cat No: KGAF018, KeyGEN) and incubating in a water bath at 37 °C for 30 minutes (min), with gentle agitation at 3 min intervals, away from light. The excess probe was discarded by washing the cells 5-times at 650× *g* for 10 min in pre-chilled phosphate-buffered saline (PBS). The cell pellet was resuspended in FACS buffer to make a homogenous suspension analyzed in a BD Accuri C6 Plus flow cytometer (Becton, Dickinson and Company, Franklin Lakes, NJ, USA) using the Fluorescein (FITC) green fluorescence channel. FCS files were analyzed in FlowJoV10 analysis software.

### 2.7. Structure-Based Virtual Screening

To identify inhibitors that confer high resistance to AFB_1_ exposure, structure-based virtual screening was conducted targeting BACH1 using the known structure of human BACH1 protein obtained from the RCSB protein database (PDB) (https://www.rcsb.org/, accessed on 30 May 2020). The Specs small-molecule compound database of 55,024 compounds was used as the ligand library for virtual docking with human BACH1(https://specs.net/, accessed on 30 May 2020). The virtual screening was performed using the Surflex-Dock of SYBYL-X 2.0 (Tripos, St. Louis, MO, USA) based on the crystal structure of human BACH1 (PDB ID: 2IHC). The SYBYL software was used to assign the standard AMBER atomic partial charges on the human BACH1 protein and the Gasteiger–Hückel atomic partial charges on the ligand candidates to be docked. After the preparation, the docking was performed using the default settings, and the figures were generated using PyMol software (https://pymol.org/2/, accessed on 30 May 2020). The abbreviations and synonyms of corresponding inhibitors (M1, M2, M3, M4, M5, M6, M7, M8, and M9) are shown in Table S2.

### 2.8. RNA Sequencing and Transcriptome Analysis

PK-15 and BACH1-KO cells were seeded into 6-well plates (Cat No: 703001, NEST, Wuxi, China) in triplicate to 60% confluence and treated with 2 μg/mL AFB_1_. The cells were harvested 36 h post-treatment, and total RNA was isolated using TRIzol RNA Isolation Reagent (Cat No: 15596-026, Invitrogen). Library construction and sequencing were conducted at the National Key Laboratory of Crop Genetic Improvement using the MGISEQ-2000RS platform. Sequence quality was assessed using FastQC (v0.11.7, https://sourceforge.net/projects/fastqc.mirror/, accessed on 1 September 2020), and quality trimming was performed using the FASTX-Toolkit (v0.0.14, http://hannonlab.cshl.edu/fastx_toolkit/, accessed on 1 September 2020) to remove bases with a Phred33 score of less than 30 while retaining the resulting reads of at least 50 bases in length. The quality-trimmed reads were mapped against the reference genome of *Sus scrofa* (v11.1) using HISAT2 (v2.1.0, http://daehwankimlab.github.io/hisat2/, accessed on 15 September 2020). Gene expression profiling was based on the number of reads. Fragments per kb of exon model per million mapped reads (FPKM) was used to estimate the expressed values and transcript levels using SAMtools (v1.7, https://www.htslib.org/, accessed on 15 September 2020) and HTSeq-count (v0.9.1, https://github.com/htseq/htseq, accessed on 15 September 2020). Differently expressed genes (DEGs) were obtained using DESeq2 (v1.30.1, https://bioconductor.org/packages/release/bioc/html/DESeq2.html, accessed on 20 September 2020) with a *p* value cutoff ≤0.05 and an absolute fold change of ≥1. The STRING database (https://string-db.org, accessed on 25 September 2020) was used to construct the DEGs’ protein–protein interaction (PPI) networks, and the core genes from gene set enrichment analysis (GSEA) (https://www.gsea-msigdb.org/gsea, accessed on 25 September 2020); then, the degree values were determined using Cytoscape v3.7.2 (https://cytoscape.org/what_is_cytoscape.html, accessed on 25 September 2020).

### 2.9. Quantitative Real-Time PCR Assay

Total cell RNA was extracted using TRIzol Reagent (Cat No: 15596-026, Invitrogen, Waltham, MA, USA). The cDNAs were synthesized using the PrimeScript™ RT reagent Kit with gDNA Eraser (Cat No: RR047A, TaKaRa, Kusatsu, Japan) in a total reaction volume of 20 μL. Each RT-qPCR reaction was carried out with 50 ng of cDNA and 5 nM primer pairs using SYBR Green Mix (Cat No: Q711-02-AA, Vazyme, Nanjing, China). The results were monitored using a CFX384 Real-Time PCR Detection System (Bio-Rad, Hercules, CA, USA) programmed for 1 cycle of 10 min at 95 °C, followed by 39 cycles of 10 s at 95 °C and 10 s at 60 °C. The relative expression levels were calculated using the 2^−ΔΔCt^ method. The *Glyceraldehyde-3-phosphate dehydrogenase (GAPDH)* gene was used as a normalization control. All primers used in quantitative PCR are listed in Table S1.

### 2.10. Western Blot Analysis

After treatment, cells were collected, and the total proteins were extracted using RIPA solution (Servicebio, Wuhan, China) containing a mixture of protease phosphatase inhibitors (Beyotime, Nanjing, China). A BCA protein assay kit was used to measure the total concentrations of proteins according to the manufacturer’s instructions. Twenty micrograms of cellular protein from each group was electro-blotted onto a PVDF membrane (Millipore, Burlington, MA, USA) following separation on 10% SDS-PAGE (Sangon Biotech, Shanghai, China). The membranes were blocked with 5% BSA in 0.1% Tween 20/Tris-buffered saline (TBST) for 2 h at room temperature and then incubated with either anti-BACH1 ((F-9): sc-271211) or anti-HOMX1 (10701-1-AP) antibodies overnight at 4 °C. Afterwards, samples were washed thrice with 0.1% TBST and incubated with secondary antibodies (1:2000) (Beyotime, Nanjing, China) at room temperature for 2 h. The enhanced chemiluminescence kit (Cat No: WBKLS0500) was used to visualize the protein bands.

### 2.11. Edu Cell Proliferation Assay

To assess cell proliferation, BACH1-KO and PK-15 cells were seeded into 12-well plates. The cells were maintained in DMEM with 10% FBS, 100 U/mL penicillin and 100 μg/mL streptomycin, incubated at 37 °C with 5% CO_2_. After 24 h, EdU cell proliferation assays were performed using the BeyoClick™ EdU Cell Proliferation Kit with Alexa Fluor 555 (Cat No: C0075S, Beyotime) according to the manufacturer’s protocol. The cell nuclei were stained with 4’,6-diamidino-2-phenylindole (DAPI) (Cat No: C1005, Beyotime) for 10 min at room temperature in the dark. Stained cells were visualized under a fluorescence microscope (DP80, Olympus, Tokyo, Japan). The proportion of EdU-positive cells were calculated in ImageJ software (three independent wells were imaged, and one random field per well was captured for each experimental phase).

### 2.12. Immunofluorescence Assay

Differences in AFB_1_-DNA adducts in BACH1-KO and WT cells were assessed with immunofluorescence. Cells were seeded in a 24-well cell culture plate with pre-attached cell slides. The plated cells were treated with 1 μg/mL AFB_1_ diluted with 2% FBS in DMEM and incubated for 36 h at 37 °C + 5% CO_2_. The cells were then fixed in 4% paraformaldehyde at 4 °C for 10 min and permeabilized at 4 °C for 10 min in cold 0.3% Triton X-100. This was followed by incubating with anti-AFB_1_ antibody (Cat No: NB600-443, NOVUS, Saint Louis, MO, USA, 1:200) at 4 °C overnight. The primary antibodies were detected using Cy3 conjugated Goat anti-Mouse IgG (Cat No: HA1102, HUABIO, Hangzhou, China, 1:1000). Cell nuclei were counter-stained with DAPI (Cat No: G1012, Servicebio) for 10 min at room temperature in the dark. The slides were mounted with an anti-fluorescence quenching mountant and observed under a fluorescence microscope (Olympus, Tokyo, Japan) using the 40× objective lens. The proportion of fluorescing cells was calculated using ImageJ software on three independent slides, each with two or three random fields of view for each experimental group.

### 2.13. M2 Anti-AFB_1_ In Vivo Experiment

Sprague-Dawley (SD) rats were used for in vivo experiments. The rats were stratified into five groups, namely, control, dimethyl sulfoxide (DMSO), M2, AFB_1_, and AFB_1_ + M2. There were five rats in each group. The rats were acclimatized to the laboratory environment for 3 days with routine clinical scoring. Each group was inoculated with the appropriate material, i.e., saline, DMSO, M2 (20 mg/kg.bw intraperitoneal injection), AFB_1_ (5 mg/kg.bw by gavage), or the combination of AFB_1_ and M2, respectively. Gross sections of rat liver were collected, stained with hematoxylin and eosin (H&E), and assessed for pathological changes. Additionally, immunohistochemistry to detect AFB_1_-DNA adduct and the extraction of liver tissue proteins for malondialdehyde (MDA) detection were conducted.

### 2.14. Hematoxylin and Eosin (H&E) Staining and Immunohistochemistry

The *SD* rats were euthanized and their liver tissue collected, fixed in 4% paraformaldehyde, and embedded in paraffin wax. Subsequently, the liver tissue was stained with H&E to make pathological sections. Sections were observed and captured using the Olympus BX53 microscope. Immunohistochemistry was performed on paraffin-embedded sections using an indirect immunoperoxidase method. The same embedded liver tissues were stained with anti-AFB_1_ antibody (Cat No: NB600-443, NOVUS, Saint Louis, MO, USA, 1:200) to detect changes of AFB_1_-DNA adducts.

### 2.15. Statistical Analysis

Statistical analysis was performed using R programming language. The means ± S.D. were determined for each treatment group. Two-tailed Student’s *t*-test was used to determine significant differences between treatment and control groups (* *p* < 0.05; ** *p* < 0.01; *** *p* < 0.001; **** *p* < 0.0001; ns, no significant).

## 3. Results

### 3.1. Genome-Scale CRISPR Screening Identifies Targets Required for AFB_1_-Induced Cell Death

**Figure 1:**
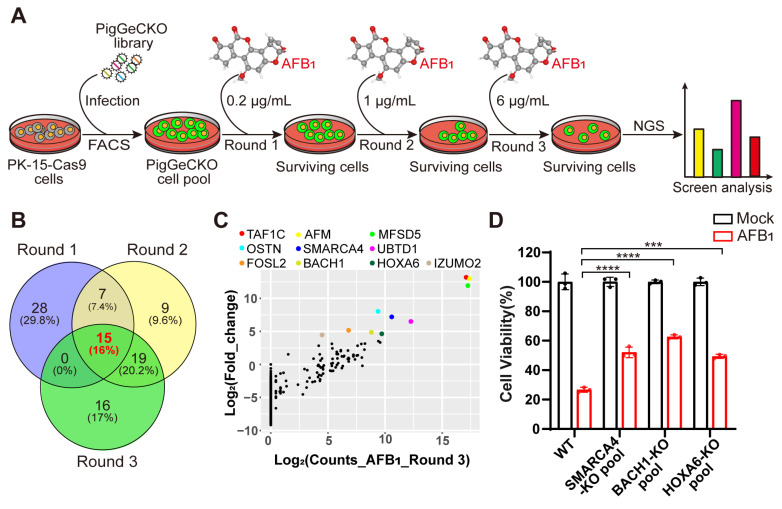
Genome-wide CRISPR/Cas9-based screening to identify genes involved in aflatoxin B_1_ toxicity. (**A**) Strategy for CRISPR/Cas9-based aflatoxin B_1_ (AFB_1_) resistance screening. PK-15 cells expressing Cas9 (PK-15-Cas9) were transduced with a lentiviral PigGeCKO library to generate a pool of mutant cells (PigGeCKO cell pool). Mutant cells were then subjected to sequential rounds of exposure to AFB_1_ at increasing concentrations (Round 1: 0.2 μg/mL, Round 2: 1.0 μg/mL, and Round 3: 6.0 μg/mL). AFB_1_ was refreshed daily until all control cells were killed. Surviving cells were harvested, and sgRNA sequences were identified by high-throughput sequencing. (**B**) Venn diagram of the top ~0.1% of shared and unique sgRNA target sequences obtained from each round of AFB_1_ screening. (**C**) Scatter plots of the frequencies of sgRNA target sequence and the extent of enrichment in transformed PK-15-Cas9 cells in Round 3 of AFB_1_ screening. (**D**) Cell viability assays for WT and KO cell pool of candidate genes after AFB_1_ exposure at 2 μg/mL for 36 h. *** *p*< 0.001, **** *p* < 0.0001. *p* values were determined with two-tailed Student’s *t*-tests. AFB_1_, aflatoxin B_1_; FACS, fluorescence-activated cell sorting; NGS, next-generation sequencing; Round 1 (2 or 3), the first (second or third) round of AFB_1_ challenge; WT, wild-type; KO, knockout.

To systematically identify the host genes required for AFB_1_-induced cell death, we performed genome-wide CRISPR KO screening in the porcine kidney (PK)-15 cells stably expressing Cas9 (hereafter referred to as PK-15-Cas9), as described in previous work [24] (Figure 1A). The overall CRISPR screening strategy is illustrated in Figure 1A. We first performed three rounds of challenge with AFB_1_, using untreated PK-15-Cas9 cells as a negative control to confirm that cell death was attributable to AFB_1_ exposure in each round of treatment. A total of 3 rounds of the AFB_1_ challenge were carried out with increasing doses of 0.2 µg/mL, 1 µg/mL, and 6 µg/mL, respectively (Figure 1A). Since oxidative damage caused by AFB_1_ is a central mechanism of AFB_1_-induced cell death [25], we sought to identify the genes involved in AFB_1_-induced oxidative damage. Thus, the surviving cells in the second round were simultaneously exposed to hydrogen peroxide (H_2_O_2_), a classical model inducer of oxidative stress, and integrated sgRNA constructs were obtained from the cells that survived AFB_1_ and H_2_O_2_ by PCR and Illumina-based sequencing.

Our CRISPR screening identified 94 unique sgRNA sequences, targeting 91 unique protein-coding genes that were present in at least ~0.1% of the total cells analyzed in 3 rounds of AFB_1_ exposure (Tables S3–S5). Among the top 0.1% of the most frequently detected sgRNAs, those targeting *BACH1, SMARCC1* and *RAB15* were highly enriched in the second round of AFB_1_ challenge (Table S4). To reduce the false positive or poorly resistant cells in the early rounds of AFB_1_ challenge, we conducted consecutive rounds of selection with increasing concentrations of AFB_1_. A comparison of enriched genes from the top 0.1% of hits revealed that 15 targets were common to all 3 rounds of the AFB_1_ challenge (Figure 1B). Interestingly, 16 genes were enriched after both AFB_1_ exposure and H_2_O_2_ stimulation, implying that KO of these genes conferred high resistance to both AFB_1_- and H_2_O_2_-induced oxidative stress in these cells (Figure S1A and Table S6). Among the top hits of H_2_O_2_ selection, *BACH1* emerged as the most significantly enriched (Figure S1B).

Subsequently, we selected 10 genes that appeared as top hits in Round 3 to test their effects on tolerance to AFB_1_ (Figure 1C). Then, utilizing the CRISPR/Cas9 genome-editing system, we created KO mutants for the selected genes in PK-15 cells. Successful disruption of each gene was confirmed by Sanger sequencing (Figure S2). Quantification of AFB_1_-induced cell death in heterogeneous pools of three PK-15 KO cells indicated that KO of the gene (i.e., *HOXA6, SMARCA4, BACH1*) conferred significant resistance to AFB_1_-induced cell death, with BACH1-deficient cells exhibiting the highest resistance (Figure 1D). Collectively, these results showed that several host factors were involved in AFB_1_-induced cell death and that BACH1 as the greatest outlier played a key role, leading us to select this target for further study.

### 3.2. BACH1 Is Required for AFB_1_-Induced Cell Death

**Figure 2:**
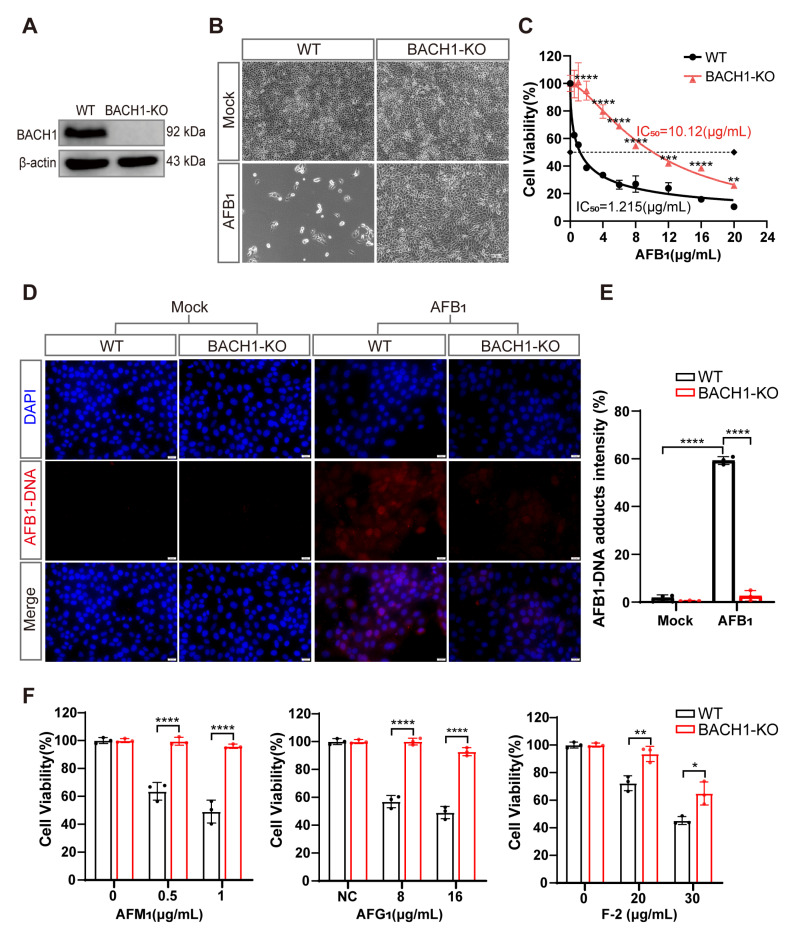
BACH1 knockout cells exhibit higher resistance to aflatoxin B_1_. (**A**) Western blot analysis of BACH1 expression in WT and KO cells. (**B**) Representative images of WT and BACH1-KO PK-15 cells challenged with 2 μg/mL AFB_1_ for 48 h. Scale bar, 100 μM. (**C**) The IC_50_ values for AFB_1_ in WT and BACH1-KO cells determined by CCK-8 assays. (**D**,**E**) Immunofluorescence staining of AFB_1_-induced DNA adduct formation in WT and BACH1-KO cells; relative fluorescence intensity calculated using ImageJ software. Scale bar, 20 μM. (**F**) Enhanced resistance to AFM_1_, AFG_1_, and F-2 in BACH1-KO cells. WT and BACH1-KO cells were treated with AFM_1_ (at 0.5 μg/mL and 1 μg/mL), AFG_1_ (at 8 μg/mL and 10 μg/mL), and F-2 (at 20 μg/mL and 30 μg/mL) for 36 h. Cell viability was measured with CCK-8 assays. * *p* < 0.05, ** *p* < 0.01, *** *p* < 0.001, **** *p* < 0.0001. *p* values were determined with two-tailed Student’s *t*-tests. AFB_1_, aflatoxin B_1_; AFG_1_, aflatoxin G1; F2, zearalenone; WT, wild-type; KO, knockout.

To explore the role of the transcriptional repressor of BACH1 in AFB_1_-induced cell death, we isolated a monoclonal BACH1-KO PK-15 cell line (BACH1-KO cells) and confirmed that a +1-bp frameshift mutation in exon 6 resulted in its disruption (Figure S3A). We also confirmed that BACH1 protein was almost completely abolished in the BACH1-KO cells, and that lack of BACH1 had no apparent effect on either cell growth or proliferation (Figure 2A and Figure S3B). Subsequent observation by light microscopy showed that BACH1-KO cells had a near complete resistance to AFB_1_, in contrast with WT cells (Figure 2B). Using CCK-8 assays, we calculated the half maximal inhibitory concentration (IC_50_) of AFB_1_ and found that BACH1-KO cells can tolerate remarkably high AFB_1_ concentrations, with an IC_50_ ~8-fold higher than WT cells (Figure 2C). Additionally, since the amino acid sequence of BACH1 is highly conserved amongst pigs and humans (Figure S4), we hypothesized that its function may be conserved across swine and *homo* *sapiens*. To test this hypothesis, we generated human BACH1-deficient Huh7 cells (hBACH1-KO cells) and confirmed that hBACH1-KO led to significantly higher resistance to AFB_1_-induced cell death (Figure S5A,B).

Furthermore, immunofluorescent staining indicated that BACH1-KO cells showed significantly less formation of AFB_1_-DNA adducts after AFB_1_ exposure compared with WT cells, with approximately 20-fold lower relative fluorescence intensity than WT cells (Figure 2D,E). Through cell-passaging assays, we found that BACH1-KO cells exposed to AFB_1_ could be swiftly restored to normal proliferation cycles and cell viability after passaging. In contrast, WT cells exposed to AFB_1_ were almost non-viable during passaging, which implied that KO of BACH1 may also confer resistance to AFB_1_-induced genotoxicity (Figure S6A,B). Notably, we observed that cells with KO of BACH1 could tolerate multiple mycotoxins, such as AFM_1_, AFG_1_, and Zearalenone (F-2) (Figure 2F). Taken together, these results indicated that BACH1 functions as an essential mediator of cytotoxicity induced by AFB_1_ or other mycotoxins.

### 3.3. BACH1 Knockout Significantly Alleviates AFB_1_-Induced Oxidative Damage by Upregulating Antioxidant Genes

**Figure 3:**
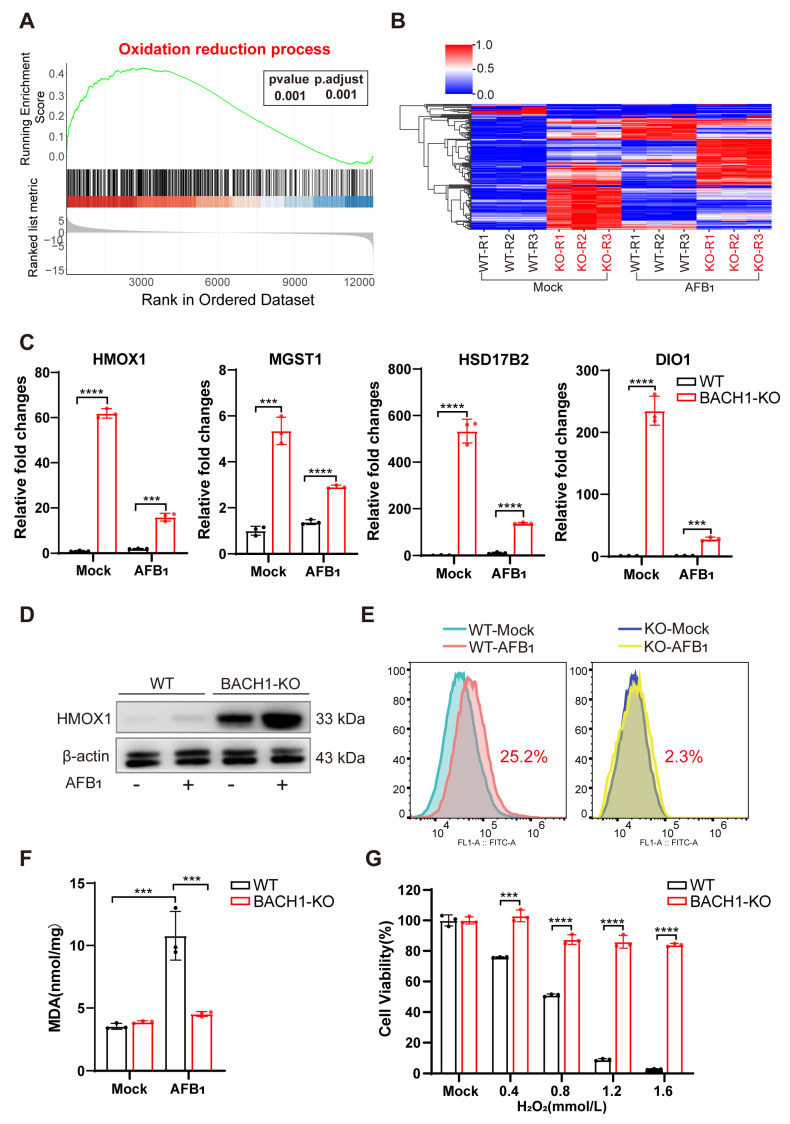
BACH1 deficiency attenuates oxidative damage from AFB_1_ via upregulation of antioxidant factors. (**A**) Gene set enrichment analysis (GSEA) identified the oxidation –reduction process as significantly enriched with differentially upregulated genes in RNA-Seq data of BACH1-KO cells treated with AFB_1_. (**B**) Heatmap of the relative expression levels of genes in the core enrichment subset of GSEA. (**C**) qPCR validation of RNA-Seq data showing differential mRNA expression of antioxidant genes in oxidation reduction. (**D**) Western blot analysis of HMOX1 in WT and BACH1-KO cells with and without AFB_1_ exposure. (**E**) BACH1-KO cells exhibit lower levels of AFB_1_-induced ROS. (**F**) Comparison of MDA levels induced by AFB_1_ in WT and BACH1-KO cells. (**G**) H_2_O_2_-induced cell death in WT and BACH1-KO cells. *** *p* < 0.001, **** *p* < 0.0001. *p* values were determined with two-tailed Student’s *t*-tests. AFB_1_, aflatoxin B_1_; WT, wild-type; KO, knockout; RT-qPCR, Real-time quantitative PCR.

Our findings support that knocking out BACH1, a transcriptional repressor, can lead to the potent inhibition of AFB_1_-induced cell death. We sought to identify the BACH1 downstream target genes to determine whether they also participated in AFB_1_-induced cell death. RNA sequencing (RNA-Seq) on BACH1-KO and WT cells with and without AFB_1_ exposure revealed a total of 2406 DEGs between the AFB_1_-treated and untreated cells, 889 DEGs (WT vs. KO), and 1915 DEGs (WT-AFB_1_ vs KO-AFB_1_) (|log_2_(FoldChange)| ≤ 2 and *p* value ≤ 0.001) (Figure S7A–C, Tables S7–S9). GSEA indicated that BACH1-depleted cells exhibited enhanced expressions of genes involved in oxidation–reduction processes compared with the WT cells (Figure 3A). Heatmap visualization of the core set of enriched DEGs from GSEA revealed a trend of significant upregulation among antioxidant genes in both WT and BACH1-KO cells after 36 h exposure to AFB_1_ (Figure 3B). The STRING database was next used to construct a DEG PPI network, and the core genes from GSEA with the highest values were determined using Cytoscape v. 3.7.2, which showed that knocking out BACH1 could affect the expression of multiple downstream genes involved in oxidation reduction (Figure S8).

Among these AFB_1_-inducible antioxidant genes, the mRNA expression of *HMOX1*, *MGST1*, *HSD17B2*, and *DIO1*, were upregulated in BACH1-KO cells with and without AFB_1_ exposure (Figure 3C and Figure S9). In addition, Western blot analysis confirmed that HMOX1 protein levels were also obviously upregulated in BACH1-KO, but not with WT cells, regardless of their exposure to AFB_1_ (Figure 3D). The results indicated that KO of BACH1 mitigated the inhibition of these antioxidant genes. To test the effects of this inhibition, we quantified ROS and MDA content, the markers of oxidative stress and lipid peroxidation, using DCFH-DA assay and TBARS assay, respectively. The results showed that ROS production was induced by AFB_1_ in WT cells and that this production was significantly reduced (i.e., from 22.5% to 2.3%) in BACH1-KO cells (Figure 3E). Furthermore, BACH1 KO also resulted in decreased levels of MDA in response to AFB_1_ (Figure 3F) and significantly inhibited cell death induced by H_2_O_2_, indicating that these BACH1-KO cells displayed a high antioxidant capacity. Taken together, these results demonstrated that BACH1 deficiency can reduce AFB_1_-induced oxidative damage by activating the expression of antioxidant factors, thus alleviating cytotoxicity.

### 3.4. Identifying Inhibitors of AFB_1_-Induced Cell Death That Target BACH1

**Figure 4:**
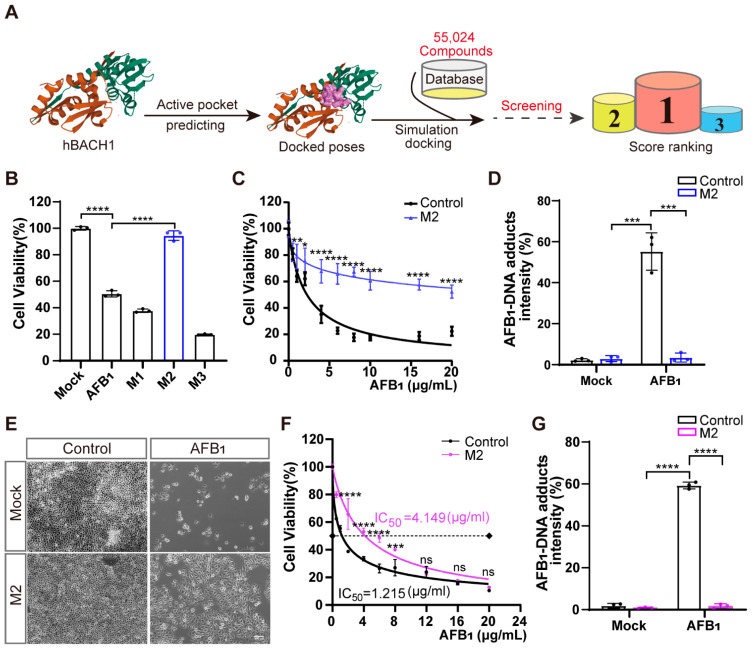
Treatment with inhibitor M2 leads to the highest resistance to aflatoxin B_1_ in vitro. (**A**) Workflow of the structure-based virtual screening to identify inhibitors targeting BACH1. (**B**) Validation of the top three inhibitors (M1, M2, and M3) in Huh7 cells by CCK-8 assays. (**C**) Comparation of Huh7 tolerance to different AFB_1_ concentrations with and without M2 treatment. (**D**) The relative fluorescence intensity of AFB_1_-DNA adducts in Huh7 cells with and without M2 treatment. (**E**) Representative light microscopy images of AFB_1_-treated PK-15 cells with or without M2 treatment. Scale bar, 100 μm. (**F**) The IC_50_ assays for AFB_1_ in PK-15 cells with and without M2 treatment determined with CCK-8 assays. (**G**) The relative fluorescence intensity of AFB_1_-DNA adducts in PK-15 cells with and without M2 treatment. * *p* < 0.05, ** *p* < 0.01, *** *p* < 0.001, **** *p* < 0.0001, ns, not significant. *p* values were determined with two-tailed Student’s *t*-tests. AFB_1_, aflatoxin B_1_; M1, 1-Piperazineethanol, 4-phenyl-α-[[(3,4,5-trimethoxyphenyl)methoxy]methyl]; M2, 1-Piperazineethanol,α-[(1,3-benzodioxol-5-yloxy)methyl]-4-(2-methoxyphenyl); M3, 1,2-Ethanediamine, N1, N1, N2, N2-tetrakis (1H-benzimidazol-2-ylmethyl).

To identify novel potential inhibitors that confer high resistance to AFB_1_ exposure, we conducted structure-based virtual screening targeting BACH1 using the known structure of human BACH1 protein obtained from the PDB database. We used the Specs small-molecule compound database of 55,024 compounds as the ligand library for virtual docking with human BACH1 (Figure 4A). Ranking the predicted activity scores revealed that compounds M1, M2, and M3 exhibited strong binding energy interacting with hBACH1 (Figure 4A and Figure S10A; abbreviations of and synonyms for M1, M2, and M3 are listed in Table S2). The treatment of Huh7 cells with 10 μg/mL of each compound showed that only exposure to M2 resulted in any significant inhibition of AFB_1_-induced cell death (Figure 4B). Furthermore, M2 did not affect the cell proliferation in cytotoxicity assays, suggesting that it has no obvious toxic effects on cells (Figure S10B). Subsequently, we found that M2 treatment could also protect cells under increasingly high concentrations of AFB_1_ in Huh7 cells (Figure 4C). To further study the effects of this inhibitor on cellular resistance to AFB_1_, we quantified the levels of AFB_1_-DNA adducts in Huh7 cells exposed to AFB_1_. The results indicated that M2-treated cells had reduced formations of AFB_1_-DNA adducts, with approximately fifteen-fold lower relative fluorescence intensity than control cells (Figure 4D).

Molecular docking and dynamic simulation of M2 on the surface of human BACH1 revealed the three amino acid residues (Ser-13, Ser-14, and Ser-17) with the lowest binding energy (2.0, 1.9, and 2.4 kcal/mol, respectively), suggesting that these residues were candidate binding sites for M2 (Figure S11A). Given the high level of conservation between human and pig BACH1 (Figure S4), we then explored these potential M2 docking loci (Ser-13, Ser-14, and Ser-17) in porcine BACH1. Sequence alignments confirmed that these residues were highly conserved (Figure S11B), which led us to test the effects of M2 in PK-15 cells. We found that M2 treatment could also confer resistance to AFB_1_-induced cell death in porcine cells (Figure 4E). Further IC_50_ assays indicated that AFB_1_ resistance was enhanced by over threefold in the presence of M2 (Figure 4F), while the formation of AFB_1_-DNA adducts was significantly reduced by M2 treatment in PK-15 cells (Figure 4G).

We next sought to determine whether other small-molecule inhibitors with a similar structure to M2 could similarly affect the cellular response to AFB_1_. We speculated that the 1,3-benzodioxin functional moieties at M2 may play a central role in mediating resistance to AFB_1_ (Figure S10A). We then selected 182 compounds with >70% similarity to M2 from the Specs small-molecule compound database. Using a virtual screening strategy, six M2 analogues, designated as M4, M5, M6, M7, M8, and M9, were chosen as candidates (Figure S12A, Table S2). Further cell viability assays showed that treatment with five of the six analogues resulted in lower AFB_1_-induced cell death in Huh7 cells, while only three analogues led to lower cell death from AFB_1_ in PK-15 cells (Figure S12B,C). These results suggested that the same inhibitors have different effects on AFB_1_ toxicity through BACH1 in different species. However, our overall results showed that M2 targeting of BACH1 resulted in significant resistance to AFB_1_ exposure in both human and porcine cells.

### 3.5. BACH1 Inhibitor Reduces the AFB_1_-Induced Liver Damage In Vivo

**Figure 5:**
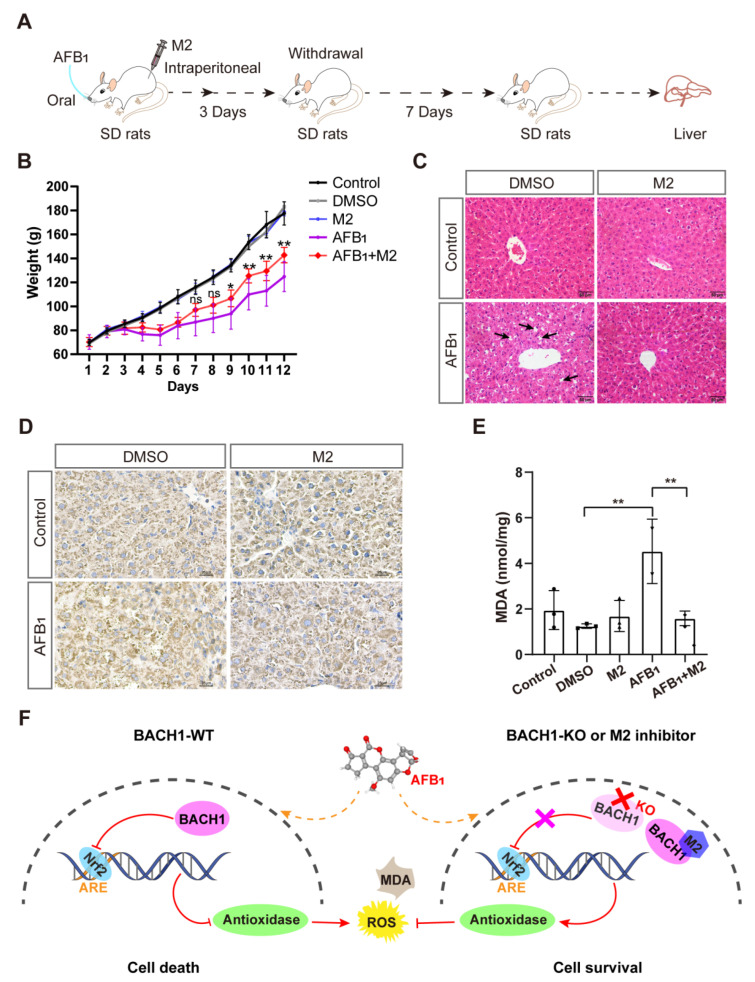
Liver damage from aflatoxin B_1_ is significantly lower in M2-treated rats. (**A**) Schematic of M2 treatment of AFB_1_-induced SD rats. (**B**) Body weights of SD rats during AFB_1_ exposure with and without M2 treatment. (**C**) Liver histopathology of AFB_1_-exposed rats and control group. The specific inflammatory cell infiltration is indicated with an arrow. Scale bar, 50 μm. (**D**) Representative light microscopy images of immunohistochemical staining for AFB_1_-DNA adducts in liver tissues with and without M2 treatment. Scale bar, 25 μm. (**E**) MDA levels in liver tissues with and without M2 treatment. (**F**) Proposed molecular model of BACH1 and M2 in AFB_1_-exposed cells. * *p* < 0.05, ** *p* < 0.01, ns, not significant. *p* values were determined with two-tailed Students’ *t*-tests. ROS, reactive oxygen species; MDA, malondialdehyde; AFB_1_, aflatoxin B_1_; WT, wild-type; KO, knockout; DMSO, dimethyl sulfoxide; DMSO used as a negative control; Nrf2, NF-E2-related factor 2; ARE, antioxidant response element; M2, 1-Piperazineethanol,α-[(1,3-benzodioxol-5-yloxy)methyl]-4-(2-methoxyphenyl); SD rats, Sprague-Dawley (SD) rats.

Given the above findings that the predicted docking locus was conserved between pigs and humans, and that M2 could mitigate the effects of AFB_1_ in both porcine and human cells, we investigated the effects of the BACH1 inhibitor M2 during exposure to AFB_1_ in vivo. To this end, we challenged healthy SD rats with AFB_1_ with and without M2 treatment (Figure S13). The overall workflow for M2 treatment assays is illustrated in Figure 5A. Briefly, healthy rats were treated with AFB_1_ with or without M2 or with M2 alone by intraperitoneal injection for three days. After challenge with AFB_1_ rats, were given a 7-day recovery period. We observed that the body weights of rats treated with AFB_1_ + M2 were higher than those of rats given AFB_1_ alone, while M2 alone at 20 mg/kg showed no cytotoxicity in rats (Figure 5B). In addition, comparisons of liver pathological tissue slices showed that symptoms of hepatocellular edema and inflammatory cell infiltration in the liver tissue caused by AFB_1_ exposure were effectively alleviated in rats treated with M2 (Figure 5C). Similarly, immunohistochemical staining of liver tissues to detect the formation of AFB_1_-DNA adducts indicated that adduct levels were lower in rats in the AFB_1_ + M2 treatment group (Figure 5D). Moreover, M2 treatment was associated with lower AFB_1_-induced MDA production in liver tissue, which implied that M2 might alleviate AFB_1_-induced liver damage by decreasing oxidative stress (Figure 5E). Taken together, these results indicated that BACH1 could serve as an effective target for the treatment of oxidative damage induced by AFB_1_, while the M2 inhibitor of BACH1 could reduce AFB_1_-induced liver damage (Figure 5F).

## 4. Discussion

Aflatoxin B_1_ (AFB_1_) and its metabolites (e.g., AFM_1_) can accumulate in animal tissues and be assimilated through human consumption of animal products. AFB_1_ toxicity in livestock and humans can lead to adverse economic losses accompanied by serious health concerns. Determining host factors required for AFB_1_ toxicity and revealing their molecular mechanisms is essential for resolving aflatoxicosis through developing novel therapeutic targets and through the targeted breeding of AFB_1_-resistant livestock, such as swine. We executed a genome-wide CRISPR/Cas9 KO screening in PK-15 cells and identified several host factors potentially related to the toxic effects of AFB_1_. Among the top candidates, the transcriptional repressor BACH1 was the most significant target of AFB_1_. We subsequently characterized BACH1 as a central mediator of AFB_1_-induced oxidative damage. Furthermore, using a structure-based virtual screening strategy, we developed a small-molecule inhibitor (M2) that attenuates AFB_1_ toxicity via targeting BACH1.

BACH1 regulates the genes involved in mitotic chromatin dynamics, apoptosis, oxidative stress response, and cell cycle [26]. However, no studies have yet reported the link between AFB_1_ and the molecular mechanism of BACH1 in AFB_1_ toxicity. We conducted an RNA-Seq analysis to uncover the gene regulatory network relevant to AFB_1_ toxicity and found significant enrichment for differentially upregulated genes involved in the oxidation–reduction process in BACH1-KO cells. Other studies have reported that AFB_1_ negatively affects the balance between antioxidants and pro-oxidants, resulting in elevated lipid peroxidation and oxidative damage [23,27]. Oxidative stress is associated with multiple disorders such as cancers, atherosclerosis, and Alzheimer’s disease [28]. Our results showed reduced ROS and MDA in BACH1 KO cells with an increased expression of antioxidant genes such as *HMOX1* and *MGST1* (Figure 5F). Therefore, BACH1 is a crucial factor in AFB_1_-induced oxidative damage by regulating the expression of antioxidant genes.

Currently, reports on BACH1 inhibitors are limited, with little or no information on their characterization and outcomes on AFB_1_ resistance [29,30,31]. Structure-based virtual screening has several advantages in identifying potential inhibitors of specific genes, such as reduced time constraints, cost-effectiveness, and higher throughput than the conventional screening of drug panels [32]. Using a structure-based virtual screening approach, we developed several inhibitors that effectively decreased AFB_1_ toxicity by targeting BACH1. Although M1 has a similar backbone to M2, M1 lacks the 1,3-benzodioxin functional moieties and has no resistance to AFB_1_ toxicity (Figure 4B and Figure S10A). Therefore, the 1,3-benzodioxin functional moieties at the chemical structure of M2 may play a central role in inhibiting AFB_1_ toxicity. We selected six other inhibitors with similar conformations to M2 and tested their effects on AFB_1_ resistance. In human cells, M4, M5, M6, M7, and M8 exhibited anti-AFB_1_ effects (Figure S12B). Structural analyses of these compounds revealed a 1,3-benzodioxin structure. Specifically, M4, M5, and M6 contain tertiary amine positive ions and elicit enhanced anti-AFB_1_ effects compared with M7 and M8 (Figure S12A,B). The phenyl or benzyloxy moieties of M5, M6, and M8 may endow the tertiary amine cation with stronger electrophilicity and enhance the polarity of the compounds with increased protein-binding capacity. In addition, we found that the same inhibitors lead to increased resistance to AFB_1_ in human cells than in porcine cells (Figure S12B,C). We used the human BACH1 structure to conduct virtual screening since the porcine BACH1 structure is unknown. This likely explains why the inhibitors conferred higher AFB_1_ resistance in human cells than in porcine cells. We, therefore, suppose that the protein structure of BACH1 may differ slightly between humans and pigs, even though the essential BACH1 binding amino acids for M2 (Ser-13, Ser-14, and Ser-17) were conserved in both species. Overall, BACH1 is a potential druggable target for AFB_1_ (Figure 5F). Further investigations are warranted for comprehensively characterizing BACH1 and M2 interactions.

Finally, we found that an M2 inhibitor can attenuate AFB_1_ toxicity in rats. Since we confirmed that M2 is effective in cells with acute exposure to AFB_1_, SD rats were used to establish an in vivo model for oxidative damage induced by acute AFB_1_ exposure. The results indicated that treatment with M2 reduces the formation of AFB_1_-DNA adducts and MDA in liver tissue, thus alleviating liver damage. AFB_1_ is hepatotoxic and is a significant factor in promoting the development of primary hepatocellular carcinoma (HCC) [5]. Moreover, other studies reported that BACH1 is upregulated in HCC samples, with BACH1 facilitating the growth and metastasis of HCC [33,34]. Therefore, we hypothesize that M2 may be effective in treating HCC, although further studies are needed to confirm this possibility.

## 5. Conclusions

In summary, we identified BACH1 as a novel host factor mediating AFB_1_ toxicity. BACH1-defficient cells can tolerate high concentrations of AFB_1_ by increasing the expression of antioxidant genes. Additionally, the inhibitor M2, which targets BACH1, can provide strong resistance to AFB_1_ toxicity in vitro and in vivo, suggesting its potential for clinical development as a therapeutic intervention against AFB_1_ toxicity.

## Data Availability

The deep-sequencing and RNA-Seq data generated in this study have been deposited and are available in the GEO database under accessions GSE199384 and GSE199385, respectively. Additional data related to this paper may be requested from the authors.

## Supplementary Materials

The following supporting information can be downloaded at: https://www.mdpi.com/2076-3921/11/9/1787/s1; Figure S1: The surviving cells in the second round were challenged with H_2_O_2_, Figure S2: Sanger sequencing of mutants aligned to reference sequences for *HOXA6*, *SMARCA4*, and *BACH1*, Figure S3: Construction of single-clone-originated *BACH1* knockout PK-15 cells, Figure S4: Alignment of the amino acid sequences of BACH1 between pig and human, Figure S5: Confirmed resistance to AFB_1_ in Huh7 cells, Figure S6: Exploration of the tolerance to AFB_1_ in WT and BACH1-KO cells with cell-passaging assays, Figure S7: Volcano map of differentially expressed genes (DEGs), Figure S8: Protein–protein interaction (PPI) network of the core genes from GSEA was visualized by Cytoscape (wild-type vs knockout), Figure S9: The mRNA expression of antioxidant genes in oxidation reduction process was tested with RT-qPCR, Figure S10: The inhibitors from the structure-based virtual screening, Figure S11: The analysis of the structure of human and pig BACH1, Figure S12: Other inhibitors with similar structures to M2, Figure S13: Alignment of the amino acid sequences of BACH1 from human and rat, Table S1: Primer pairs and sgRNA-targeting sequences used in this study, Table S2: Inhibitors used in this study, Table S3: Sequencing results for the top ~0.1% of sgRNA (PigGeCKO) in the first round of AFB_1_ screens after challenge, Table S4: Sequencing results for the top ~0.1% of sgRNA (PigGeCKO) in the second round of AFB_1_ screens after challenge, Table S5: Sequencing results for the top ~0.1% of sgRNA (PigGeCKO) in the third round of AFB_1_ screens after challenge, Table S6: Sequencing results for the top ~0.1% of sgRNA (PigGeCKO) in H_2_O_2_ screens after challenge, Table S7: Differential gene expression analysis between PK-15 cells (WT) and BACH1-KO cells by RNA-Seq, Table S8: Differential gene expression analysis between untreated PK-15 cells and AFB_1_-treated PK-15 cells by RNA-Seq, Table S9: Differential gene expression analysis between AFB_1_-treated PK-15 cells (WT) and AFB_1_-treated BACH1-KO cells by RNA-Seq.
