# Correction: Zhang et al. Genome-Scale CRISPR Knockout Screening Identifies BACH1 as a Key Regulator of Aflatoxin B_1_-Induced Oxidative Damage. *Antioxidants* 2022, *11*, 1787

**DOI:** 10.3390/antiox12020446

**Published:** 2023-02-10

**Authors:** Jinfu Zhang, Siyi Hu, Changzhi Zhao, Yuan Zhou, Lu Zhang, Hailong Liu, Peng Zhou, Sheng Li, Liangliang Fu, Zhuqing Zheng, Yue Xiang, Xuewen Xu, Jinxue Ruan, Xinyun Li, Lvhui Sun, Gang Cao, Shuhong Zhao, Xu Wang, Shengsong Xie

**Affiliations:** 1 Key Laboratory of Agricultural Animal Genetics, Breeding and Reproduction of Ministry of Education & Key Lab of Swine Genetics and Breeding of Ministry of Agriculture and Rural Affairs, Huazhong Agricultural University, Wuhan 430070, China; 2 Guangdong Laboratory of Lingnan Modern Agriculture, Guangzhou 510642, China; 3 National Reference Laboratory of Veterinary Drug Residues (HZAU) and MAO Key Laboratory for Detection of Veterinary Drug Residues, Huazhong Agricultural University, Wuhan 430070, China; 4 Hubei Hongshan Laboratory, Huazhong Agricultural University, Wuhan 430070, China; 5 The Cooperative Innovation Center for Sustainable Pig Production, Huazhong Agricultural University, Wuhan 430070, China

In the original publication [1], a mistake was identified in Figure 4 as published. The explanation in Figure 4E is wrong. The corrected Figure 4 appears below. The authors state that the scientific conclusions are unaffected. This correction was approved by the Academic Editor. The original publication has also been updated.

## Figures and Tables

**Figure 4:**
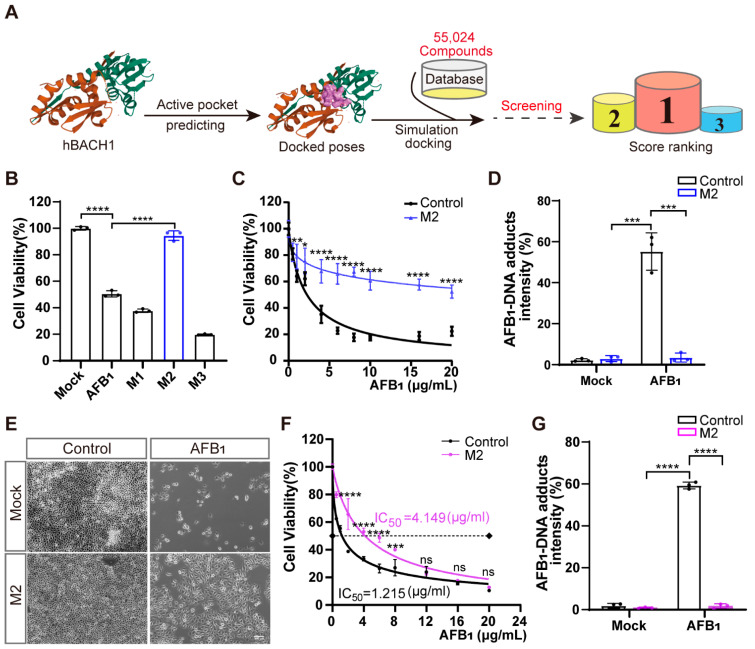
Treatment with inhibitor M2 leads to the highest resistance to aflatoxin B_1_ in vitro. (**A**) Workflow of the structure-based virtual screening to identify inhibitors targeting BACH1. (**B**) Validation of the top three inhibitors (M1, M2, and M3) in Huh7 cells by CCK-8 assays. (**C**) Comparation of Huh7 tolerance to different AFB_1_ concentrations with and without M2 treatment. (**D**) The relative fluorescence intensity of AFB_1_-DNA adducts in Huh7 cells with and without M2 treatment. (**E**) Representative light microscopy images of AFB_1_-treated PK-15 cells with or without M2 treatment. Scale bar, 100 μm. (**F**) The IC_50_ assays for AFB_1_ in PK-15 cells with and without M2 treatment determined with CCK-8 assays. (**G**) The relative fluorescence intensity of AFB_1_-DNA adducts in PK-15 cells with and without M2 treatment. * *p* < 0.05, ** *p* < 0.01, *** *p* < 0.001, **** *p* < 0.0001, ns, not significant. *p* values were determined with two-tailed Student’s *t*-tests. AFB_1_, aflatoxin B_1_; M1, 1-Piperazineethanol, 4-phenyl-α-[[(3,4,5-trimethoxyphenyl)methoxy]methyl]; M2, 1-Piperazineethanol,α-[(1,3-benzodioxol-5-yloxy)methyl]-4-(2-methoxyphenyl); M3, 1,2-Ethanediamine, N1, N1, N2, N2-tetrakis (1H-benzimidazol-2-ylmethyl).
