# Supplementary material for: Genome-Scale CRISPR Knockout Screening Identifies BACH1 as a Key Regulator of Aflatoxin B1-Induced Oxidative Damage
Source: Antioxidants (Basel). 2022 Sep 10;11(9):1787. doi: 10.3390/antiox11091787 (PMC9495794; doi:10.3390/antiox11091787)
Supplement: Supplementary file 1 [file antioxidants-11-01787-s001.zip › antioxidants-1890170-supplementary.pdf]

Supplementary Materials

Supplementary Figures

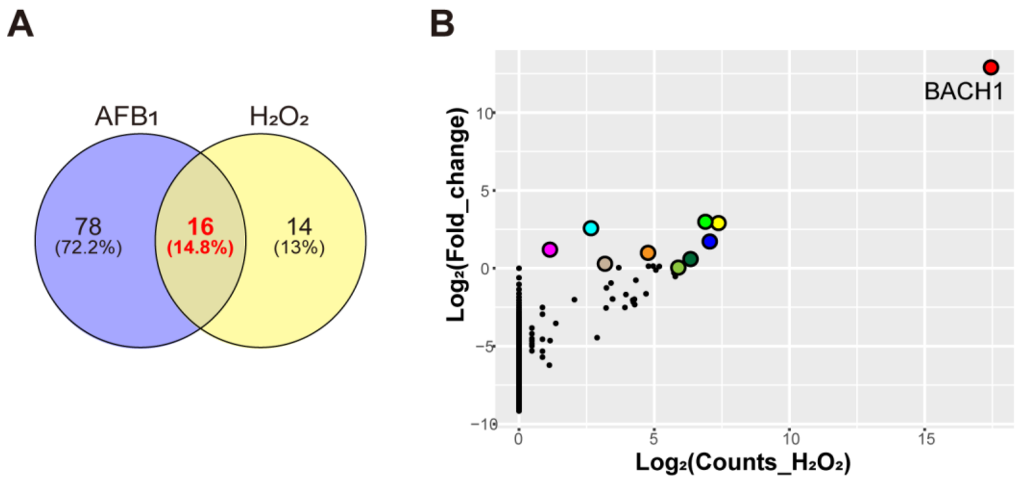

Figure S1. The surviving cells in the second round were challenged with H_2O_2. (A) Venn diagram for the overlaping enrichment in specific sgRNA targeting sequences for the three rounds of AFB_1 screening and one round of H_2O_2 screening among the top ~0.1% sgRNAs reads. **(B)** Scatter plots of the frequencies of sgRNA target sequence and the extent of enrichment in transformed PK-15-Cas9 cells in H_2O_2 challenge. AFB_1, Aflatoxin B_1; H_2O_2, hydrogen peroxide.

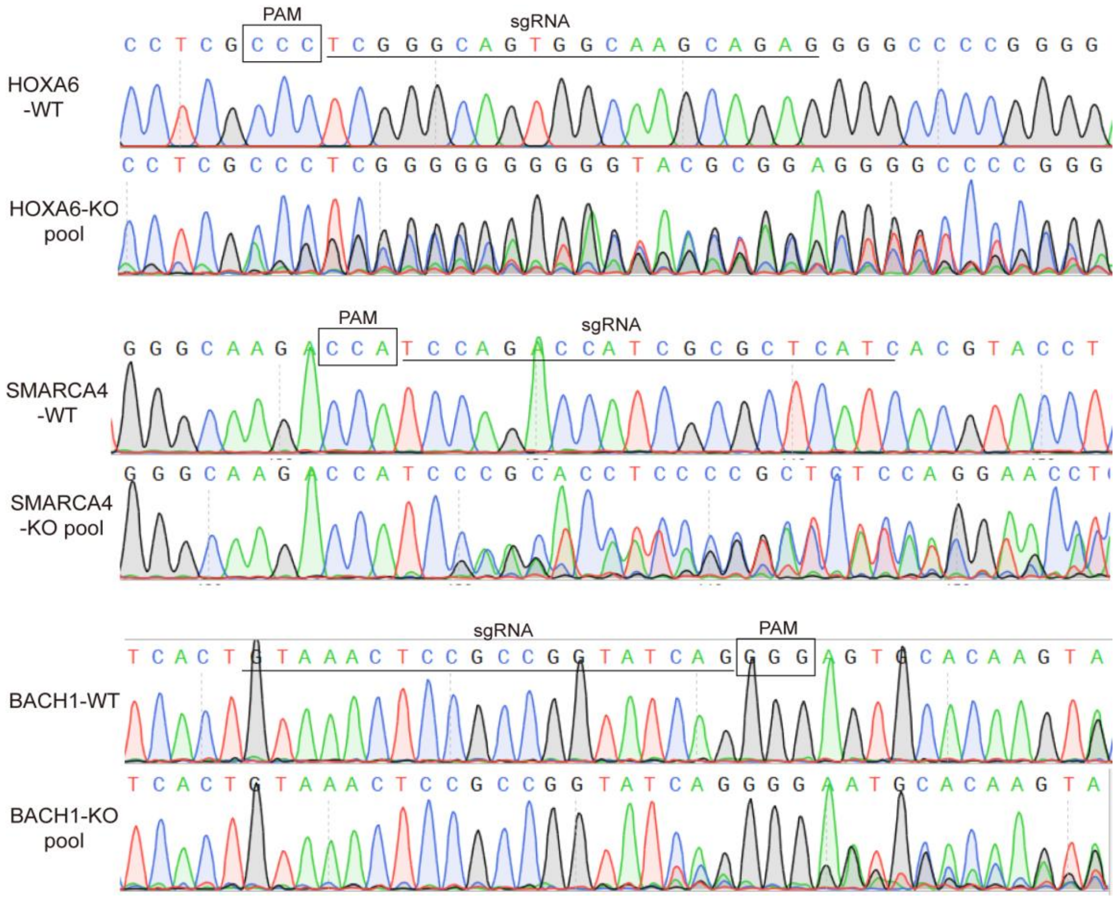

Figure S2. Sanger sequencing of mutants aligned to reference sequences for HOXA6, SMARCA4 and BACH1. WT, wild-type; KO, knockout; PAM, protospacer adjacent motif; sgRNA, small guide RNA.

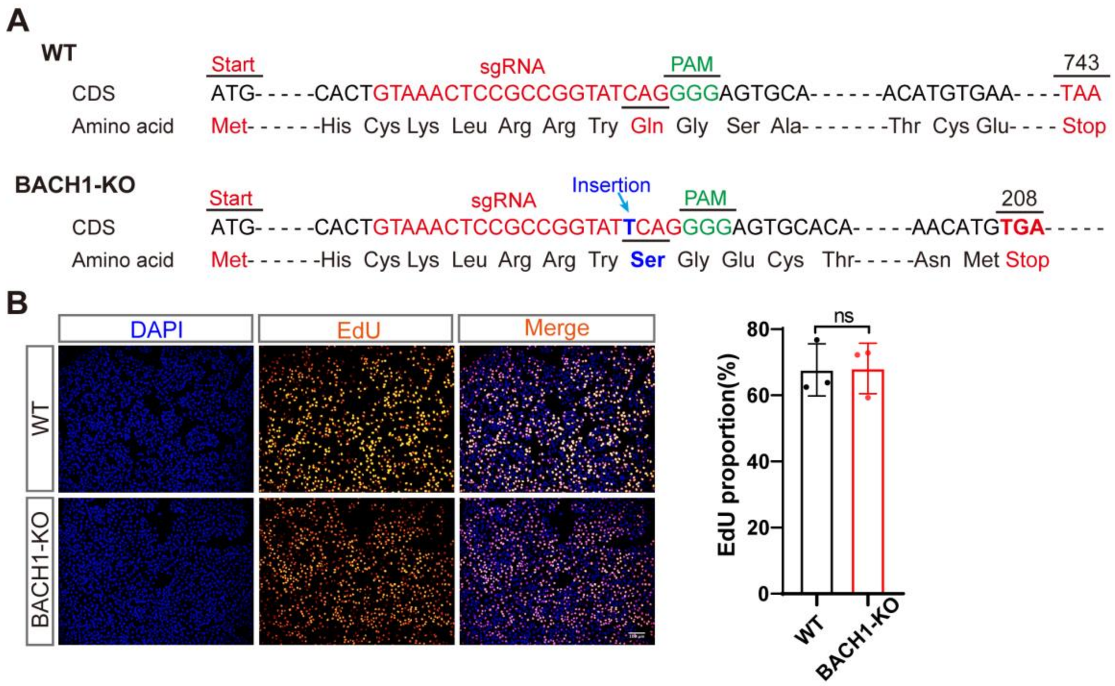

Figure S3. Construction of single-clone-originated BACH1 knockout PK-15 cells. **(A)** Alignment of the nucleic acid and amino acid sequences of BACH1-KO cells with WT cells. The inserted base (Thymine) is highlighted in blue. **(B)** Comparison of cell proliferation of BACH1-KO and WT cells by EdU fluorescence assays. Scale bar, 100 μm. WT, wild-type; KO, knockout; PAM, protospacer adjacent motif; CDS, coding sequences; ns, not significant; DAPI, 4',6-diamidino-2-phenylindole.

```
BACH1_pig      MSLGESAVFAYESSVHSAHVLLSLDEQRRKDVLCDVTVLVEGQPFRAHRAVLAACSAYFH
BACH1_human    MSLSENSVFAYESSVHSTNVLLSLNDQRKKDVLCDVTIFVEGQRFRAHRSVLAACSSYFH
               ***.*.:**********::*****::**:********::**** *****:******:***

BACH1_pig      ARLSGQADAELRITLPEEVTVKGFEPLIQFAYTAKLILSKDNVDEVCKCVEFLGVSDIEE
BACH1_human    SRIVGQADGELNITLPEEVTVKGFEPLIQFAYTAKLILSKENVDEVCKCVEFLSVHNIEE
               :*: ****.**.****************************:************.* :***

BACH1_pig      SCFQFLKYKFLDSAADQQECPRKKCFSSSCQKPDFKFSLLDQKDLEIDEVEEFLEHKNVR
BACH1_human    SCFQFLKFKFLDSTADQQECPRKKCFSSHCQKTDLKLSLLDQRDLETDEVEEFLENKNVQ
               *******:*****:************** ***.*:*:*****:*** ********:***:

BACH1_pig      TPHCKLRRYQGSAQVLPPLQDSASQTCESMCLEKDAALSLPSLCPKYRKFQKAFGTDRVR
BACH1_human    TPQCKLRRYQGNAKASPPLQDSASQTYESMCLEKDAALALPSLCPKYRKFQKAFGTDRVR
               **:********.*:. ********** ***********:*********************
```

```
BACH1_pig        AVESSVKDIHTSSVQPNEPSERECSGGVQDCADLQVILKCEERKLAMEHEEAKKKDPASQ
BACH1_human      TGESSVKDIHAS-VQPNERSENECLGGVPECRDLQVMLKCDESKLAMEPEETKK-DPASQ
                 : ********:* ***** **.** *** :* ****:***:* ***** **:** *****

BACH1_pig        CPSEKTGATPFPP-SSTDPHGLYSLSLLHTYDQYGDLNFAGMQNTAVLTEKPLSGSDVQE
BACH1_human      CPTEKSEVTPFPHNSSIDPHGLYSLSLLHTYDQYGDLNFAGMQNTTVLTEKPLSGTDVQE
                 **:**: .****  ** ****************************:*********:****

BACH1_pig        EKTFGESQDLRLKSDSGSREDSSLASSDLSSVEREVAEHLAKGFWTDICSTDSPCQMQLS
BACH1_human      K-TFGESQDLPLKSDLGTREDSSVASSDRSSVEREVAEHLAKGFWSDICSTDTPCQMQLS
                 : ******** **** *:*****:**** ****************:******:*******

BACH1_pig        PAMAKDGSEQIYSQKRSECPWLGIRISESPEPGQRTFTTLSSVNCPFISTLSTEGSSSNL
BACH1_human      PAVAKDGSEQIS-QKRSECPWLGIRISESPEPGQRTFTTLSSVNCPFISTLSTEGCSSNL
                 **:********  ******************************************.****

BACH1_pig        EIGNDDYVSEPQQEPCPYACVISLGDDSETDTEGDSEPCSAREQECEVKLPFNAQRIISL
BACH1_human      EIGNDDYVSEPQQEPCPYACVISLGDDSETDTEGDSESCSAREQECEVKLPFNAQRIISL
                 *************************************.**********************

BACH1_pig        SRNDFQSLLKMHKLTPEQLDCIHDIRRRSKNRIAAQRCRKRKLDCIQNLESEIEKLQNEK
BACH1_human      SRNDFQSLLKMHKLTPEQLDCIHDIRRRSKNRIAAQRCRKRKLDCIQNLESEIEKLQSEK
                 *********************************************************.**

BACH1_pig        ESLLKERDHILSTLGETKQNLTGLCQQVCKEAALSQEQIQILAKYSASDCPLSFLVSEKG
BACH1_human      ESLLKERDHILSTLGETKQNLTGLCQKVCKEAALSQEQIQILAKYSAADCPLSFLISEKD
                 **************************:********************:*******:***.

BACH1_pig        KSSSDGELVLPSILSLPEGPAAAPPSGEQSPHYPSAKGSEAGQESSATVSAPSEPAGPGE
BACH1_human      KSTPDGELALPSIFSLSDRPPAVLPPCARG---NSEPGYARGQESQQMSTATSEQAGPAE
                 **:.****.****:**.: *.*. *.  :.    *  *   ****.   :*.** ***.*

BACH1_pig        QCRQSGGISDFCQQMTDKCTTDE
BACH1_human      QCRQSGGISDFCQQMTDKCTTDE
                 ***********************
```

Figure S4. Alignment of the amino acid sequences of BACH1 between pig and human. Porcine BACH1 (ENSSSCP00000057103), ~83.15% identity to human BACH1 (ENSP00000382805).

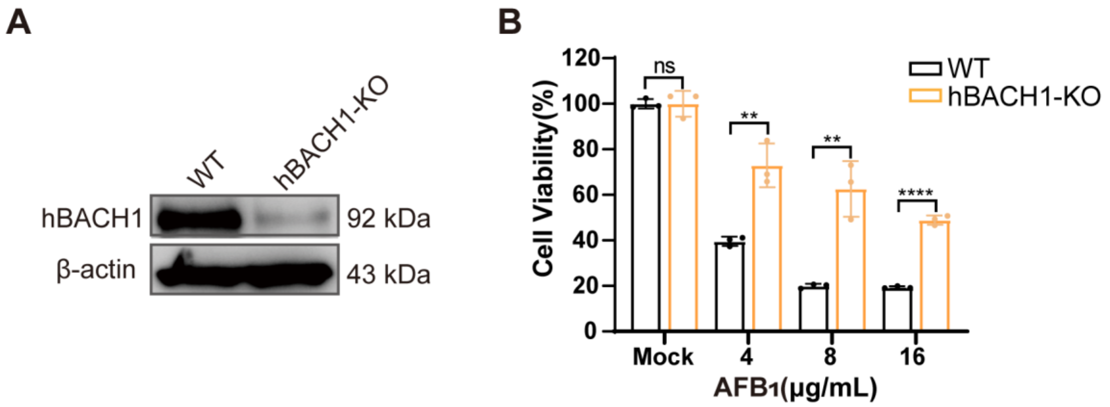

Figure S5. Comfirmed the resistance to AFB$_1$ in Huh7 cells. (A) Western blot analysis of BACH1 in WT (Huh7) and human BACH1 knockout cells (hBACH1-KO). **(B)** Cell viability assay of WT and hBACH1-KO cells with AFB$_1$ challenge by CCK-8 assays. $**p < 0.01$; $****p < 0.0001$, ns, not significant. *P* values were determined by two-tailed Student's t-tests. Huh7, human hepatocyte cells; AFB$_1$, Aflatoxin B$_1$; WT, wild-type; KO, knockout; kDa, kilodaltons; ns, not significant.

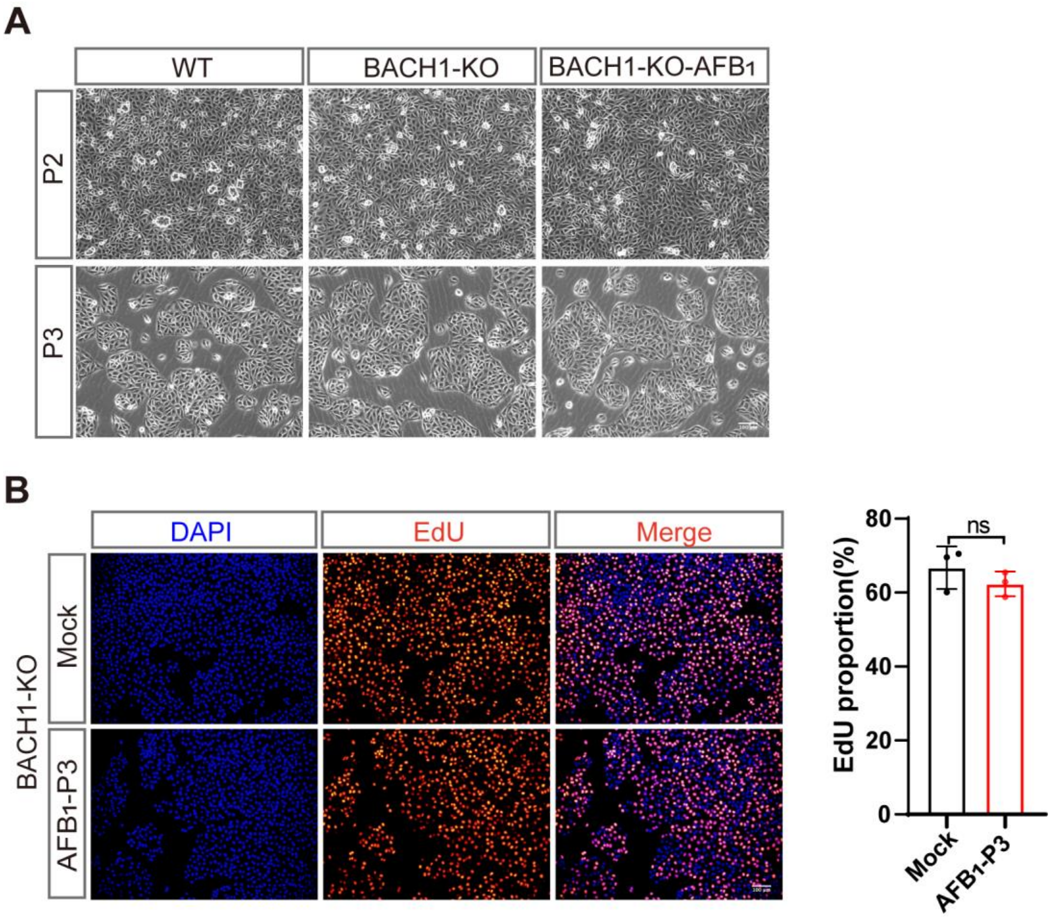

Figure S6. Exploration of the tolerance to AFB_1 in WT and BACH1-KO cells by cell passaging assays. (A) Typical cell images of second and third passage WT and BACH1-KO cells. Scale bar, 100 μm. **(B)** Cell viability of third-generation BACH1-KO cells with or without AFB_1 challenge after passaging by EdU assay. Scale bar, 100 μm. P1, the first generation of cell passage; P2, the second generation of cell passage; P3, the third generation of cell passage; AFB_1, Aflatoxin B_1; WT, wild-type; KO, knockout; DAPI, 4', 6-diamidino-2-phenylindole; ns, not significant.

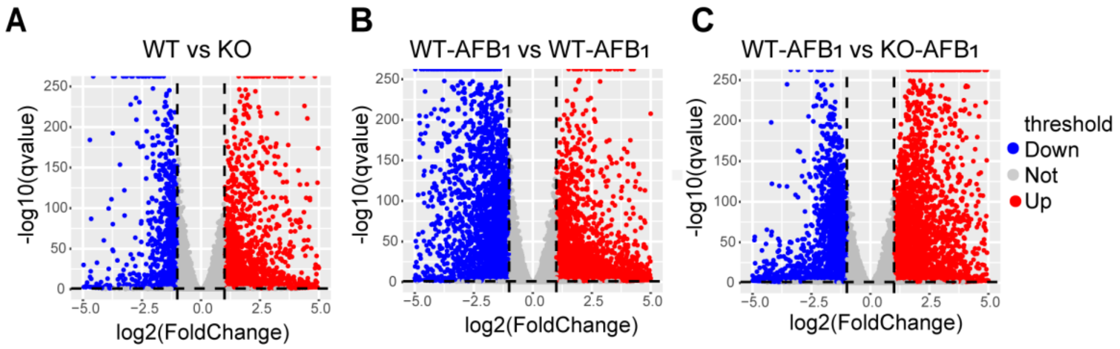

Figure S7. Volcano map of differentially expressed genes (DEGs). (A) Volcano map of DEGs between WT and BACH1-KO cells. **(B)** Volcano map of DEGs between AFB_1-treated and -untreated cells. **(C)** Volcano map of DEGs between AFB_1-treated WT and BACH1-KO cells. AFB_1, Aflatoxin B_1; WT, wild-type; KO, knockout.

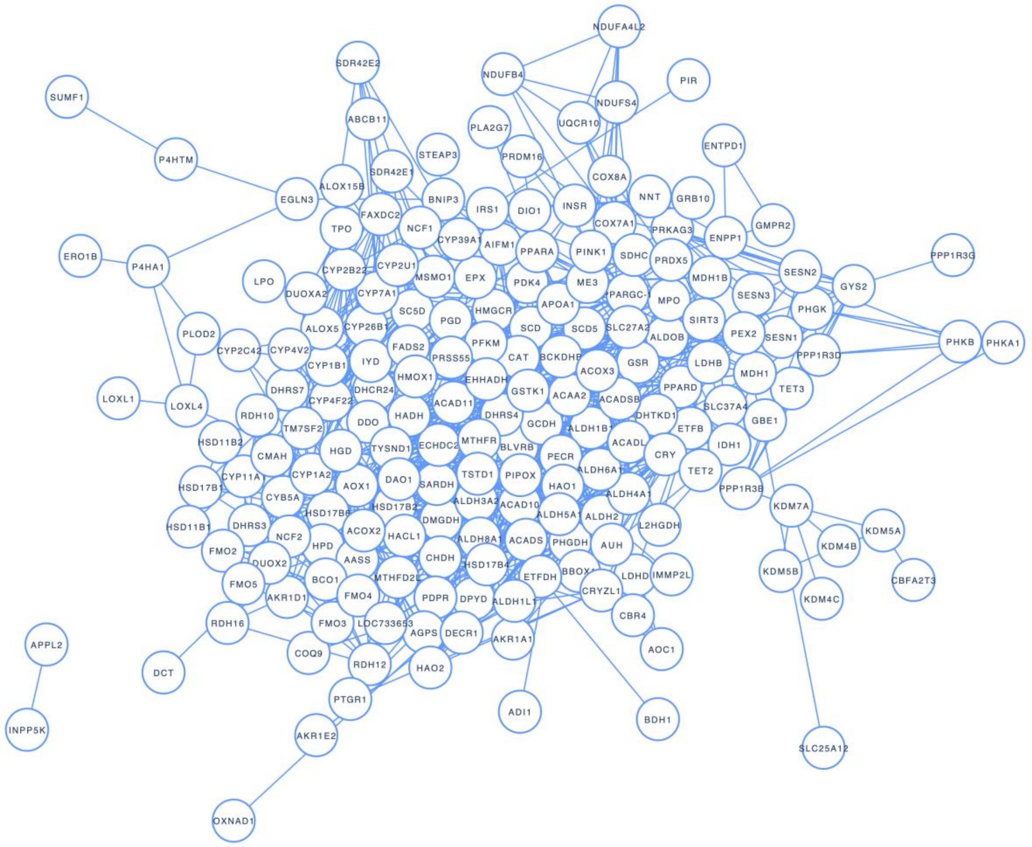

Figure S8. Protein-protein interaction (PPI) network of the core genes from GSEA was visualized by Cytoscape (wild-type vs knockout).

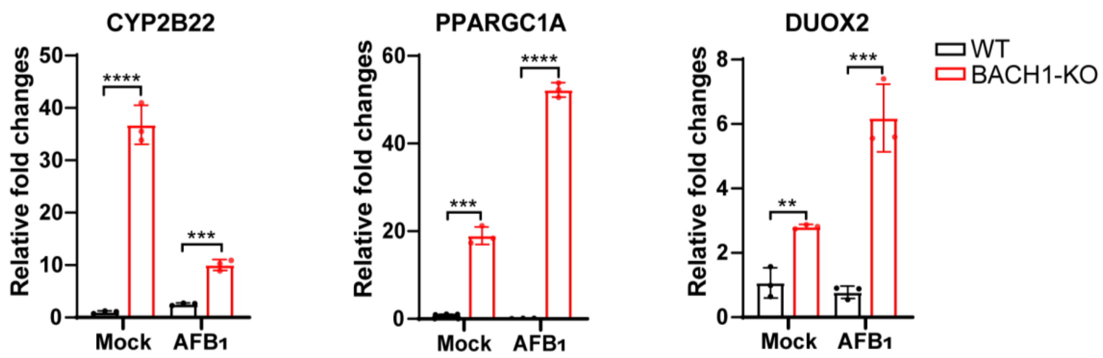

Figure S9. The mRNA expression of antioxidant genes in oxidation reduction process were tested by RT-qPCR. **p < 0.01; ***p < 0.001; ****p <0.0001. *P* values were determined by two-tailed Student's t-tests. AFB_1, Aflatoxin B_1; WT, wild-type; KO, knockout.

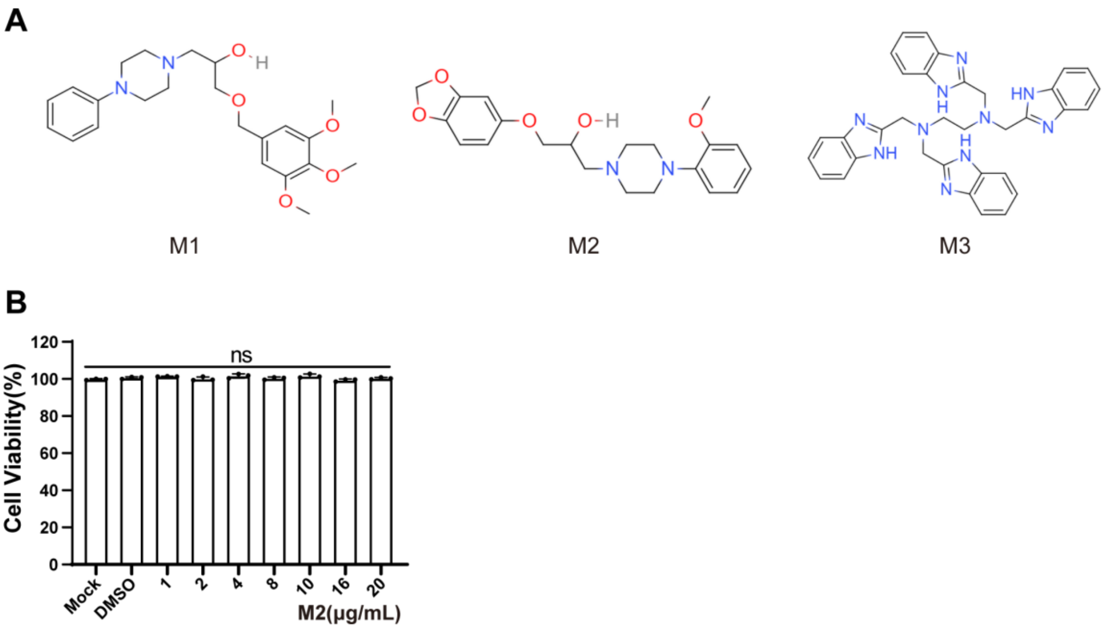

Figure S10. The inhibitors from the structure-based virtual screening. (A) The chemical structures of M1, M2 and M3 inhibitors. **(B)** Cytotoxicity assay of M2 inhibitor in Huh7. DMSO, Dimethyl sulfoxide; *P* values were determined by two-tailed Student's t-tests. ns, not significant.

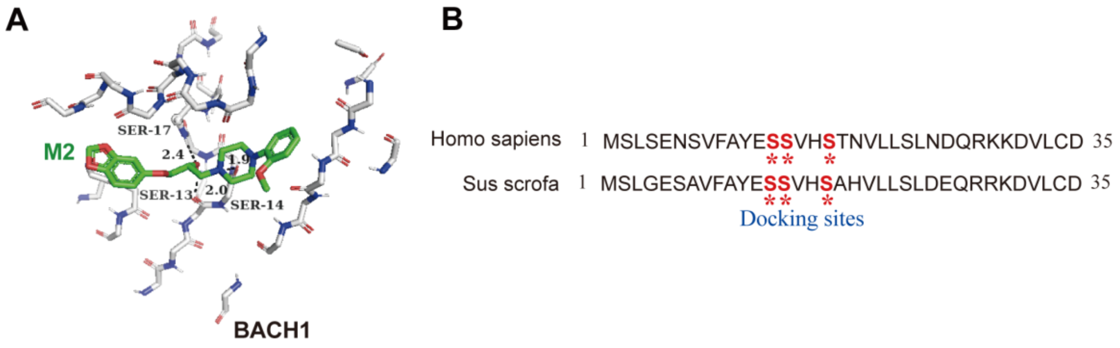

Figure S11. The analysis of the structure human and pig BACH1. (A) Modeling and dynamics simulation of M2 on the molecular surface of BACH1. The coordinate for the truncated peptide was generated using a hybrid docking molecular dynamics simulation and the graphic was generated using PyMOL. The predicted binding sites are three serine (Ser-13, Ser-14 and Ser-17). **(B)** Alignment of the amino acid sequences of BACH1 from human and pig. The docking sites for virtual screening are highlighted in red (Ser-13, Ser-14 and Ser-17).

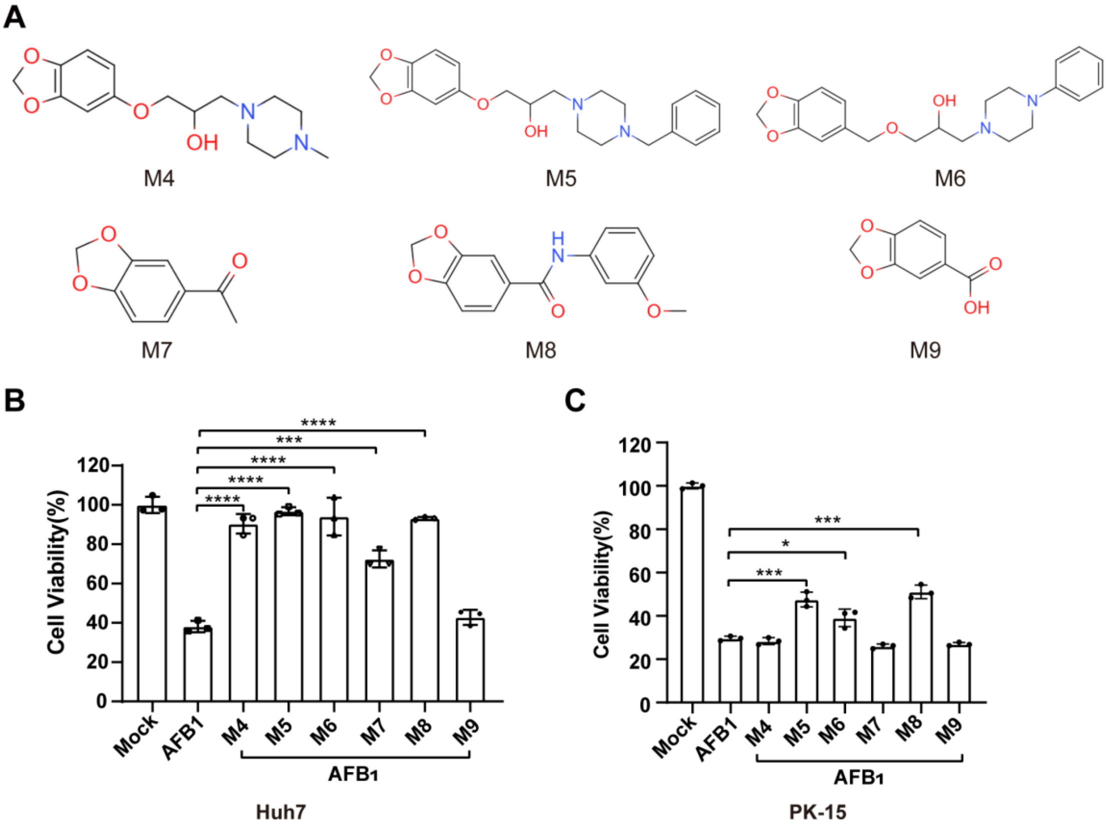

Figure S12. Other inhibitors with similar structure to M2. (A) The chemical structures of M2 analogues (M4, M5, M6, M7, M8 and M9). **(B)** Cell viability assay for AFB_1-treated Huh7 with or without M2 analogues treatment. **(C)** Cell viability assay for AFB_1-treated PK-15 with or without M2 analogues treatment. $*p < 0.05$; $***p < 0.001$; $****p < 0.0001$. *P* values were determined by two-tailed Student's t-tests. AFB_1, Aflatoxin B_1.

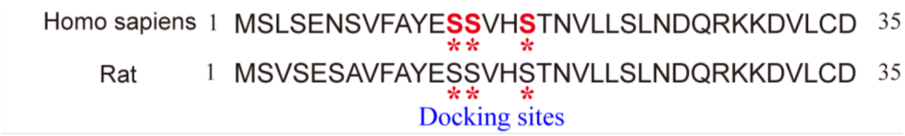

Figure S13. Alignment of the amino acid sequences of BACH1 from human and rat. The docking sites for virtual screening are highlighted in red (Ser-13, Ser-14 and Ser-17).

Supplementary Tables

Table S1. Primer pairs and sgRNAs targeting sequences used in this study.

**Name**	**Sequence (5'-3')**
BACH1-sgRNA	GTAAACTCCGCCGGTATCAGGGG
HOXA6-sgRNA	CTCTGCTTGCCACTGCCCGAGGG
SMARCA4-sgRNA	GATGAGCGCGATGGTCTGGATGG
hBACH1-sgRNA	TGTTCAGCTCTCCATCAGCCTGG
BACH1-seq-385-F	AGTAAGGACAATGTGGACGAAG
BACH1-seq-385-R	ATTTGGGGCATAAAGACGG
HOXA6-seq-657-F	TGTGAATCCCACTTTCCCCG
HOXA6-seq-657-R	CACTAGTGCCCTGCCTTCAT
SMARCA4-seq-503-F	GTCACTGAGTTCCCCTGGTG
SMARCA4-seq-503-R	TTGATCCGCTTGTGCTCCAT
hBACH1-seq-619-F	GCTTTCCACTGAACTTCCCG
hBACH1-seq-619-R	TGGAAAGGGCCCCTAGAAGA
MGST1-qPCR-172-F	TCATGGCCTTTGCCTCCTAT
MGST1-qPCR-172-R	TCCACTCTTTCATCTGTCCG
DUOX2-qPCR-200-F	GAAGACAAGTCCCGCCTCAT
DUOX2-qPCR-200-R	TCCTCCCACGTCAGCTCCTG
HMOX1-qPCR-195-F	CAATGTCGCCAACGCCACCA
HMOX1-qPCR-195-R	TCGGATGCCTGCGAGGGTCT
CYP2B22-qPCR-142-F	CTCTGCCCTTCCTGGGGAAC
CYP2B22-qPCR-142-R	GCCTCTGTCCCGCATATCAT
PPARGC1A-qPCR-127-F	GGACTCAAGTGGTGCAGTGA
PPARGC1A-qPCR-127-R	GCGTCTCTGTGAGAACTGCT
DIO1-qPCR-180-F	GGGACTTCATGCAAGGCAAC
DIO1-qPCR-180-R	TGTTCTTAAAAGCCCAGCCA
HSD17B2-qPCR-170-F	GAACATCAGCAGCATGGCAG
HSD17B2-qPCR-170-R	TGCCTGCGATGTTTGTTTGG

Table S2. Inhibitors used in this study.

CAS ID	Name	Abbreviation
325730-17-4	1-Piperazineethanol,4-phenyl-α-[[(3,4,5-trimethoxyphenyl)methoxy]methyl]	M1
380192-64-3	1-Piperazineethanol,α-[(1,3-benzodioxol-5-yloxy)methyl]-4-(2-methoxyphenyl)	M2
72583-85-8	1,2-Ethanediamine, N1,N1,N2,N2-tetrakis(1H-benzimidazol-2-ylmethyl)	M3
325742-47-0	1-Piperazineethanol,α-[(1,3-benzodioxol-5-yloxy)methyl]-4-methyl	M4
503429-84-3	1-Piperazineethanol,α-[(1,3-benzodioxol-5-yloxy)methyl]-4-(phenylmethyl)	M5
325738-10-1	1-Piperazineethanol,α-[(1,3-benzodioxol-5-ylmethoxy)methyl]-4-phenyl	M6
3162-29-6	Ethanone, 1-(1,3-benzodioxol-5-yl)	M7
214553-48-7	1,3-Benzodioxole-5-carboxamide,N-(3-methoxyphenyl)	M8
94-53-1	1,3-Benzodioxole-5-carboxylic acid	M9

Table S3. Sequencing results of the top ~0.1% of sgRNA (PigGeCKO) in the first round of AFB_1 screens after challenge.

sgRNA ID	Gene ID	Average(PigGeCKO cells)	Average(AFB_1_R1)	Fold_change(AFB_1_R1/PigGeCKO cells)	log2(Fold_change(AFB_1_R1/PigGeCKO cells))
ENSSSCG00000022945_8_33973548_33973577_m	UCHL1	1.57	12647.79	8055.92	12.98
ENSSSCG00000013856_2_61008355_61008384_p	AP1M1	0.53	1355.27	2557.11	11.32
ENSSSCG00000027396_3_117490128_117490157_p	FOSL2	2.10	3554.14	1692.45	10.72
ENSSSCG00000026564_11_12285065_12285094_m	CCDC169	7.33	8107.29	1106.04	10.11
ENSSSCG00000008950_8_73811506_73811535_m	AFM	20.47	16528.76	807.46	9.66
ENSSSCG00000012029_13_203136492_203136521_p	BACH1	22.04	16360.04	742.29	9.54
ENSSSCG00000008226_3_61535860_61535889_m	POLR1A	5.77	4135.26	716.68	9.49
ENSSSCG00000011815_13_137965913_137965942_m	OSTN	1.57	1052.25	670.23	9.39
ENSSSCG00000013629_2_70273494_70273523_p	SMARCA4	9.45	6311.26	667.86	9.38
ENSSSCG00000028881_10_15382528_15382557_p	CNIH3	11.01	5515.12	500.92	8.97
ENSSSCG00000014365_2_148257036_148257065_p	SRA1	11.02	5494.20	498.57	8.96

ENSSSCG00000017075_16_75022525_75022554_p	MFAP3	1.57	746.77	475.65	8.89
ENSSSCG00000002680_6_5035332_5035361_p	TAF1C	14.16	6477.81	457.47	8.84
ENSSSCG00000006806_4_120107475_120107504_p	SLC16A4	2.10	939.01	447.15	8.80
ENSSSCG00000013474_2_75915745_75915774_m	S1PR4	2.62	994.29	379.50	8.57
ENSSSCG00000006957_4_1126695_1126724_m	RHPN1	24.14	8963.19	371.30	8.54
ENSSSCG00000024236_14_24959657_24959686_m	MMP17	18.35	6437.15	350.80	8.45
ENSSSCG00000016949_16_47506658_47506687_p	ENSSSCG00000016949	3.67	1287.16	350.73	8.45
ENSSSCG00000012272_X_46693387_46693416_m	ZNF157	7.87	2693.24	342.22	8.42
ENSSSCG00000012029_13_203136500_203136529_m	BACH1	14.69	4705.38	320.31	8.32
ENSSSCG00000010554_14_120970518_120970547_m	SCD	2.62	837.20	319.54	8.32
ENSSSCG00000008041_3_42316011_42316040_p	PKD1	33.06	9744.08	294.74	8.20
ENSSSCG00000021297_15_71285249_71285278_m	ERMN	22.57	6598.05	292.34	8.19
ENSSSCG00000009848_14_36226		12.59	3627.47	288.12	8.17

663_36226692_p	ENSSSCG00000009848				
ENSSSCG00000021275_12_40732637_40732666_p	DDX52	3.14	891.97	284.07	8.15
ENSSSCG00000016702_18_50059679_50059708_m	HOXA6	32.54	8949.44	275.03	8.10
ENSSSCG00000002671_6_4709289_4709318_p	ATP2C2	2.63	697.62	265.25	8.05
ENSSSCG00000028150_12_19389568_19389597_m	LSM12	9.98	2583.81	258.90	8.02
ENSSSCG00000014233_2_130984709_130984738_p	ENSSSCG00000014233	20.99	5434.16	258.89	8.02
ENSSSCG00000009370_11_15687673_15687702_p	FOXO1	62.98	14948.47	237.35	7.89
ENSSSCG00000006871_4_129731482_129731511_m	SLC35A3	5.26	1162.60	221.03	7.79
ENSSSCG00000017108_16_82436229_82436258_p	ENSSSCG00000017108	8.39	1832.22	218.38	7.77
ENSSSCG00000014874_9_12270726_12270755_p	B3GNT6	35.14	7625.30	217.00	7.76
ENSSSCG00000022417_12_40846103_40846132_m	HNF1B	37.77	8135.46	215.39	7.75
ENSSSCG00000010081_14_53601771_53601800_p	MAPK1	1.58	339.31	214.75	7.75

ENSSSCG00000014988_9_37503309_37503338_p	MMP13	5.76	1229.19	213.40	7.74
ENSSSCG00000006276_4_87369205_87369234_m	CEBPD	8.39	1780.28	212.19	7.73
ENSSSCG00000000234_5_17945655_17945684_p	GRASP	5.77	1219.90	211.42	7.72
ENSSSCG00000021902_16_57490152_57490181_p	GABRP	13.65	2876.66	210.74	7.72
ENSSSCG00000007432_17_53792339_53792368_m	ZNF335	0.52	109.56	210.70	7.72
ENSSSCG00000012148_X_14166417_14166446_m	SYAP1	1.05	218.37	207.97	7.70
ENSSSCG00000012030_13_204166142_204166171_p	CLDN8	19.93	4121.82	206.81	7.69
ENSSSCG00000022498_8_19709864_19709893_p	ZCCHC4	2.62	504.16	192.43	7.59
ENSSSCG00000010070_14_53344124_53344153_p	SMARCB1	6.30	1124.76	178.53	7.48
ENSSSCG00000000264_5_18913263_18913292_p	MFSD5	39.33	6957.30	176.90	7.47
ENSSSCG00000003229_6_51672837_51672866_m	ETFB	7.34	1245.34	169.67	7.41
ENSSSCG00000009936_14_44139447_44139476_p	FOXN4	37.79	6385.71	168.98	7.40
ENSSSCG00000009164_8_127129539_127129568_m	CISD2	24.67	3933.23	159.43	7.32

ENSSSCG00000020901_6_50053548_50053577_m	ENSSSCG00000020901	51.93	8109.63	156.16	7.29
ENSSSCG00000003636_6_85555942_85555971_p	THRAP3	3.67	551.15	150.18	7.23

Table S4. Sequencing results of the top ~0.1% of sgRNA (PigGeCKO) in the second round of AFB_1 screens after challenge.

**sgRNA ID**	**Gene ID**	**Average(PigGeCKO cells)**	**Average(AFB_1_R2)**	**Fold_change(AFB_1_R2/PigGeCKO cells)**	**log2(Fold_change(AFB_1_R2/PigGeCKO cells))**
ENSSSCG00000022945_8_33973548_33973577_m	UCHL1	1.57	64251.61	40924.59	15.32
ENSSSCG00000008950_8_73811506_73811535_m	AFM	20.47	245759.27	12005.83	13.55
ENSSSCG00000002680_6_5035332_5035361_p	TAF1C	14.16	161691.17	11418.87	13.48
ENSSSCG00000008041_3_42316011_42316040_p	PKD1	33.06	162212.08	4906.60	12.26
ENSSSCG00000000264_5_18913263_18913292_p	MFSD5	39.33	180673.85	4593.79	12.17
ENSSSCG00000013629_2_70273494_70273523_p	SMARCA4	9.45	35094.99	3713.76	11.86
ENSSSCG00000016702_18_50059679_50059708_m	HOXA6	32.54	103225.09	3172.25	11.63
ENSSSCG00000011815_13_13796	OSTN	1.57	4620.00	2942.68	11.52

5913_137965942_m					
ENSSSCG00000027396_3_117490128_117490157_p	FOSL2	2.10	4101.30	1953.00	10.93
ENSSSCG00000024236_14_24959657_24959686_m	MMP17	18.35	33843.67	1844.34	10.85
ENSSSCG00000012029_13_203136500_203136529_m	BACH1	14.69	23694.39	1612.96	10.66
ENSSSCG00000014233_2_130984709_130984738_p	ENSSSCG00000014233	20.99	33512.70	1596.60	10.64
ENSSSCG00000009848_14_36226663_36226692_p	ENSSSCG00000009848	12.59	19744.37	1568.26	10.61
ENSSSCG00000009706_14_18150996_18151025_p	GALNTL6	13.64	20605.87	1510.69	10.56
ENSSSCG00000011713_13_99606295_99606324_p	P2RY12	75.04	110384.82	1471.01	10.52
ENSSSCG00000014365_2_148257036_148257065_p	SRA1	11.02	7969.07	723.15	9.50
ENSSSCG00000008226_3_61535860_61535889_m	POLR1A	5.77	3486.76	604.29	9.24
ENSSSCG00000012029_13_203136492_203136521_p	BACH1	22.04	11908.08	540.29	9.08
ENSSSCG00000021297_15_71285249_71285278_m	ERMN	22.57	11144.55	493.78	8.95
ENSSSCG00000000466_5_320661	XPOT	60.86	29373.41	482.64	8.91

42_32066171_p					
ENSSSCG00000023332_7_107227692_107227721_p	ENSSSCG00000023332	21.52	9252.00	429.93	8.75
ENSSSCG00000003207_6_50845894_50845923_p	IZUMO2	58.21	22633.75	388.83	8.60
ENSSSCG00000003636_6_85555942_85555971_p	THRAP3	3.67	1319.20	359.45	8.49
ENSSSCG00000030733_17_39862161_39862190_m	DEFB124	97.06	31718.43	326.79	8.35
ENSSSCG00000021439_13_42297920_42297949_p	CCDC66	55.07	17651.35	320.53	8.32
ENSSSCG00000026076_2_95592885_95592914_m	ENSSSCG00000014165	65.05	20392.21	313.49	8.29
ENSSSCG00000013828_2_62032013_62032042_p	OR1I1	55.63	15498.32	278.60	8.12
ENSSSCG00000027964_JH118887.1_2269_2298_p	NF2	61.87	17097.89	276.35	8.11
ENSSSCG00000022417_12_40846103_40846132_m	HNF1B	37.77	9928.84	262.88	8.04
ENSSSCG00000026405_10_34558268_34558297_p	NTRK2	120.67	31573.51	261.65	8.03
ENSSSCG00000004408_1_84788450_84788479_p	SMPD2	27.28	6763.13	247.92	7.95
ENSSSCG00000017379_12_19910	ETV4	6.82	1682.73	246.74	7.95

437_19910466_p					
ENSSSCG00000006957_4_1126695_1126724_m	RHPN1	24.14	5780.93	239.48	7.90
ENSSSCG00000004027_1_4653405_4653434_m	PDE10A	54.01	12884.64	238.56	7.90
ENSSSCG00000004191_1_34824268_34824297_p	MOXD1	17.31	3795.19	219.25	7.78
ENSSSCG00000028077_GL892321.2_43041_43070_m	SMARCC1	34.10	7242.57	212.39	7.73
ENSSSCG00000005731_1_306494303_306494332_m	GTF3C4	55.61	11437.21	205.67	7.68
ENSSSCG00000009164_8_127129539_127129568_m	CISD2	24.67	4904.28	198.80	7.64
ENSSSCG00000007897_3_32446530_32446559_m	SNN	241.26	45061.09	186.77	7.55
ENSSSCG00000008215_3_60604189_60604218_m	SMYD1	18.87	3110.19	164.82	7.36
ENSSSCG00000013049_2_7352436_7352465_p	RCOR2	11.03	1556.92	141.15	7.14
ENSSSCG00000008337_3_76322684_76322713_p	AAK1	34.11	4274.67	125.32	6.97
ENSSSCG00000009936_14_44139447_44139476_p	FOXN4	37.79	4380.19	115.91	6.86
ENSSSCG00000002823_6_26784633_26784662_p	GNAO1	68.74	7588.02	110.39	6.79
ENSSSCG00000017264_12_12369	AMZ2	98.12	10385.52	105.85	6.73

421_12369450_p					
ENSSSCG00000007538_17_69243300_69243329_p	GATA5	67.66	7152.46	105.71	6.72
ENSSSCG00000017945_12_54921259_54921288_m	TMEM95	14.69	1483.19	100.97	6.66
ENSSSCG00000005485_1_285722107_285722136_m	AMBP	35.16	3410.96	97.01	6.60
ENSSSCG00000010250_14_78234246_78234275_m	VPS26A	24.13	2245.21	93.05	6.54
ENSSSCG00000012735_X_139157055_139157084_m	ENSSSCG00000034848	64.01	5880.38	91.87	6.52

Table S5. Sequencing results of the top ~0.1% of sgRNA (PigGeCKO) in the third round of AFB_1 screens after challenge.

**sgRNA ID**	**Gene ID**	**Average(PigGeCKO cells)**	**Average(AFB_1_R3)**	**Fold_change(AFB_1_R3/PigGeCKO cells)**	**log2(Fold_change(AFB_1_R3/PigGeCKO cells))**
ENSSSCG00000002680_6_5035332_5035361_p	TAF1C	14.16	140814.17	9944.50	13.28
ENSSSCG00000008950_8_73811506_73811535_m	AFM	20.47	172942.18	8448.57	13.04
ENSSSCG00000000264_5_18913263_18913292_p	MFSD5	39.33	155051.87	3942.33	11.94
ENSSSCG00000011815_13_137965913_137965942_m	OSTN	1.57	667.31	425.04	8.73

ENSSSCG00000013629_2_70273494_70273523_p	SMARCA4	9.45	1532.14	162.13	7.34
ENSSSCG00000010521_14_118445553_118445582_p	UBTD1	51.91	4869.69	93.81	6.55
ENSSSCG00000027396_3_117490128_117490157_p	FOSL2	2.10	110.21	52.48	5.71
ENSSSCG00000012029_13_203136500_203136529_m	BACH1	14.69	447.75	30.48	4.93
ENSSSCG00000016702_18_50059679_50059708_m	HOXA6	32.54	842.11	25.88	4.69
ENSSSCG00000003207_6_50845894_50845923_p	IZUMO2	58.21	679.87	11.68	3.55
ENSSSCG00000022945_8_33973548_33973577_m	UCHL1	1.57	17.27	11.00	3.46
ENSSSCG00000003636_6_85555942_85555971_p	THRAP3	3.67	39.58	10.78	3.43
ENSSSCG00000011713_13_99606295_99606324_p	P2RY12	75.04	758.37	10.11	3.34
ENSSSCG00000008041_3_42316011_42316040_p	PKD1	33.06	300.70	9.10	3.19
ENSSSCG00000004408_1_84788450_84788479_p	SMPD2	27.28	241.52	8.85	3.15
ENSSSCG00000023036_13_33722567_33722596_m	SMARCC1	26.23	191.97	7.32	2.87
ENSSSCG00000017379_12_19910437_19910466_p	ETV4	6.82	40.86	5.99	2.58

ENSSSCG00000000466_5_32066142_32066171_p	XPOT	60.86	291.14	4.78	2.26
ENSSSCG00000017945_12_54921259_54921288_m	TMEM95	14.69	68.88	4.69	2.23
ENSSSCG00000010250_14_78234246_78234275_m	VPS26A	24.13	106.78	4.43	2.15
ENSSSCG00000029236_2_75232960_75232989_p	ZBTB7A	16.27	68.64	4.22	2.08
ENSSSCG00000009706_14_18150996_18151025_p	GALNTL6	13.64	53.62	3.93	1.97
ENSSSCG00000008226_3_61535860_61535889_m	POLR1A	5.77	18.51	3.21	1.68
ENSSSCG00000004191_1_34824268_34824297_p	MOXD1	17.31	53.44	3.09	1.63
ENSSSCG00000008215_3_60604189_60604218_m	SMYD1	18.87	57.73	3.06	1.61
ENSSSCG00000004027_1_4653405_4653434_m	PDE10A	54.01	149.74	2.77	1.47
ENSSSCG00000013828_2_62032013_62032042_p	OR1I1	55.63	147.96	2.66	1.41
ENSSSCG00000002280_7_95401896_95401925_m	RAB15	73.95	195.05	2.64	1.40
ENSSSCG00000002280_7_95401898_95401927_p	RAB15	71.33	182.04	2.55	1.35
ENSSSCG00000028077_GL892321.2_43041_43070_m	SMARCC1	34.10	84.38	2.47	1.31

ENSSSCG00000016894_16_35350808_35350837_m	ARL15	30.93	73.43	2.37	1.25
ENSSSCG00000012735_X_139157055_139157084_m	ENSSSCG00000034848	64.01	148.99	2.33	1.22
ENSSSCG00000005485_1_285722107_285722136_m	AMBP	35.16	81.55	2.32	1.21
ENSSSCG00000026405_10_34558268_34558297_p	NTRK2	120.67	249.20	2.07	1.05
ENSSSCG00000005731_1_306494303_306494332_m	GTF3C4	55.61	110.82	1.99	0.99
ENSSSCG00000001849_7_60254653_60254682_p	ANPEP	6.30	12.35	1.96	0.97
ENSSSCG00000014365_2_148257036_148257065_p	SRA1	11.02	21.35	1.94	0.95
ENSSSCG00000024230_17_45270342_45270371_m	MYL9	29.37	55.89	1.90	0.93
ENSSSCG00000027964_JH118887.1_2269_2298_p	NF2	61.87	113.82	1.84	0.88
ENSSSCG00000021297_15_71285249_71285278_m	ERMN	22.57	41.31	1.83	0.87
ENSSSCG00000003512_6_73311888_73311917_m	EIF4G3	59.31	105.53	1.78	0.83
ENSSSCG00000030733_17_39862161_39862190_m	DEFB124	97.06	164.32	1.69	0.76
ENSSSCG00000009848_14_36226		12.59	19.35	1.54	0.62

663_36226692_p	ENSSSCG00000009848				
ENSSSCG00000016290_15_147077530_147077559_p	EFHD1	81.81	111.70	1.37	0.45
ENSSSCG00000006748_4_115673644_115673673_p	TSPAN2	36.19	41.85	1.16	0.21
ENSSSCG00000013098_2_9930116_9930145_m	ENSSSCG00000013098	51.38	58.76	1.14	0.19
ENSSSCG00000014579_9_259963_259992_p	TMEM41B	35.14	38.42	1.09	0.13
ENSSSCG00000007646_3_7364517_7364546_m	C7orf43	17.85	16.99	0.95	-0.07
ENSSSCG00000022425_6_147700506_147700535_p	CC2D1B	57.19	54.14	0.95	-0.08
ENSSSCG00000014004_2_79287769_79287798_p	PLPP2	26.75	23.95	0.90	-0.16

Table S6. Sequencing results of the top ~0.1% of sgRNA (PigGeCKO) in H_2O_2 screens after challenge.

**sgRNA ID**	**Gene ID**	**Average(PigGeCKO cells)**	**Average(H_2O_2)**	**Fold_change(H_2O_2/PigGeCKO cells)**	**log2(Fold_change(H_2O_2/PigGeCKO cells))**
ENSSSCG00000012029_13_203136492_203136521_p	BACH1	22.04	175708.2622	7972.244201	12.96077019
ENSSSCG00000002680_6_5035332_	TAF1C	14.16	115.93945	8.18781447	3.033478412

5035361_p			29		
ENSSSCG00000008950_8_73811506_73811535_m	AFM	20.47	164.692779	8.0455681	3.008194294
ENSSSCG00000000264_5_18913263_18913292_p	MFSD5	39.33	131.424383	3.341581058	1.74053087
ENSSSCG00000009848_14_36226663_36226692_p	ENSSSCG00000009848	12.59	26.37135925	2.094627423	1.06669365
ENSSSCG00000005731_1_306494303_306494332_m	GTF3C4	55.61	79.84457932	1.435795348	0.521850129
ENSSSCG00000001849_7_60254653_60254682_p	ANPEP	6.3	7.965495173	1.264364313	0.338412221
ENSSSCG00000004408_1_84788450_84788479_p	SMPD2	27.28	30.15819194	1.10550557	0.144706294
ENSSSCG00000006957_4_1126695_1126724_m	RHPN1	24.14	26.57427635	1.100839948	0.13860473
ENSSSCG00000016702_18_50059679_50059708_m	HOXA5	32.54	35.44589628	1.089302283	0.123404359
ENSSSCG00000025794_3_18191044_18191073_m	FBRS	54.56	58.05771676	1.064107712	0.089644192
ENSSSCG00000016692_18_49340371_49340400_p	TAX1BP1	11.55	11.88736763	1.029209319	0.041536425
ENSSSCG00000014579_9_259963_259992_p	TMEM41B	35.14	32.3759182	0.921340871	-0.118193082
ENSSSCG00000012735_X_139157055_139157084_m	ENSSSCG00000	64.01	51.98563793	0.812148694	-0.300184204

	034848				
ENSSSCG00000001702_7_45122795_45122824_p	SLC35B2	78.15	53.85176036	0.689082026	-0.537252367
ENSSSCG00000008041_3_42316011_42316040_p	PKD1	33.06	19.00096843	0.574741937	-0.799013774
ENSSSCG00000009270_11_118882_118911_m	IFT88	19.41	9.574827575	0.493293538	-1.019481706
ENSSSCG00000023332_7_107227692_107227721_p	ENSSSCG00000023332	21.52	8.398265839	0.390253989	-1.357514717
ENSSSCG00000026898_14_82092628_82092657_m	FAM149B1	79.74	24.92436053	0.31257036	-1.677747115
ENSSSCG00000008827_8_40588991_40589020_p	FRYL	48.78	14.48399162	0.296924797	-1.751830511
ENSSSCG00000003481_6_70192250_70192279_p	PADI1	75.55	18.25717736	0.241656881	-2.04896802
ENSSSCG00000016692_18_49340409_49340438_p	TAX1BP1	41.97	9.967014821	0.237479505	-2.074125084
ENSSSCG00000001849_7_60254627_60254656_m	ANPEP	42.49	10.04818166	0.236483447	-2.080188892
ENSSSCG00000011713_13_99606295_99606324_p	P2RY12	75.04	17.47280286	0.23284652	-2.102548774
ENSSSCG00000003773_6_125965019_125965048_m	AK5	83.93	17.74323985	0.211405217	-2.241917116
ENSSSCG00000009142_8_120826836_120826865_p	SEC24B	15.75	3.137497965	0.19920622	-2.3276654

ENSSSCG00000030733_17_39862161_39862190_m	DEFB124	97.06	18.40586408	0.189633877	-2.39871138
ENSSSCG00000011811_13_135363665_135363694_m	LPP	85.52	14.09180438	0.164777881	-2.6014055
ENSSSCG00000016692_18_49340410_49340439_p	TAX1BP1	53.53	8.317098998	0.155372669	-2.686195343
ENSSSCG00000023653_3_39067801_39067830_m	GLIS2	9.44	0.824957911	0.087389609	-3.516394439

Table S7. Differential gene expression analysis between PK-15 cells (WT) and BACH1-KO cells by RNA-Seq.

Gene names	Gene ID	baseMean	log2FoldChange	lfcSE	stat	pvalue	padj
ENSSSCG00000024067	KRT23	78.28	-9.75	1.19	-8.18	0.00	0.00
ENSSSCG00000008809	GABRG1	95.89	-9.07	1.19	-7.63	0.00	0.00
ENSSSCG00000015320	CALCR	70.12	-8.62	1.19	-7.23	0.00	0.00
ENSSSCG00000027093	FOLH1B	31.88	-8.45	1.21	-6.99	0.00	0.00
ENSSSCG00000032321	MGAT4C	98.92	-8.22	1.03	-7.97	0.00	0.00
ENSSSCG00000006764	PTPN22	25.40	-8.12	1.22	-6.68	0.00	0.00
ENSSSCG00000014924	CTSC	1137.53	-8.04	0.29	-27.69	0.00	0.00
ENSSSCG00000007807	CD19	46.46	-8.03	1.20	-6.69	0.00	0.00
ENSSSCG00000038419		21.58	-7.89	1.22	-6.44	0.00	0.00
ENSSSCG00000033919	DCLK1	73.49	-7.79	1.04	-7.53	0.00	0.00
ENSSSCG00000017012	SLIT3	14.86	-7.35	1.24	-5.93	0.00	0.00
ENSSSCG00000009239		53.11	-7.32	1.04	-7.03	0.00	0.00
ENSSSCG00000028908	DYNLT3	13.69	-7.23	1.24	-5.81	0.00	0.00
ENSSSCG00000003326		12.51	-7.10	1.25	-5.66	0.00	0.00

ENSSSCG00000013307	LMO2	12.37	-7.08	1.25	-5.65	0.00	0.00
ENSSSCG00000004207		12.21	-7.06	1.26	-5.63	0.00	0.00
ENSSSCG00000020984	LXN	11.02	-6.92	1.27	-5.47	0.00	0.00
ENSSSCG00000033894		19.04	-6.73	1.23	-5.49	0.00	0.00
ENSSSCG00000008930	TMPRSS11F	64.00	-6.58	0.75	-8.83	0.00	0.00
ENSSSCG00000008929		8.68	-6.57	1.28	-5.13	0.00	0.00
ENSSSCG00000023032	SCG2	7.18	-6.30	1.30	-4.84	0.00	0.00
ENSSSCG00000033369		7.18	-6.30	1.31	-4.81	0.00	0.00
ENSSSCG00000034731		6.34	-6.12	1.32	-4.63	0.00	0.00
ENSSSCG00000009489		11.18	-5.96	1.26	-4.73	0.00	0.00
ENSSSCG00000037832	PMP22	11.02	-5.93	1.26	-4.71	0.00	0.00
ENSSSCG00000017470	TNS4	1598.27	-5.91	0.13	-46.25	0.00	0.00
ENSSSCG00000015037	IL18	173.80	-5.85	0.47	-12.42	0.00	0.00
ENSSSCG00000003051	CD177	5.02	-5.78	1.36	-4.24	0.00	0.00
ENSSSCG00000003011		5.02	-5.78	1.36	-4.26	0.00	0.00
ENSSSCG00000002134	RNASE10	4.84	-5.73	1.36	-4.21	0.00	0.00
ENSSSCG00000002838	ZNF423	9.51	-5.72	1.27	-4.49	0.00	0.00
ENSSSCG00000031615	SERPINB9	9.36	-5.69	1.27	-4.47	0.00	0.00
ENSSSCG00000014093	CRHBP	16.84	-5.65	1.08	-5.21	0.00	0.00
ENSSSCG00000039053	VGF	4016.55	-5.64	0.14	-39.34	0.00	0.00
ENSSSCG00000029388	PDE2A	239.31	-5.61	0.29	-19.57	0.00	0.00
ENSSSCG00000004807	SCG5	4.02	-5.46	1.41	-3.87	0.00	0.00
ENSSSCG00000032320	TCIM	87.79	-5.44	0.45	-12.15	0.00	0.00
ENSSSCG00000005707	FIBCD1	3.84	-5.40	1.40	-3.84	0.00	0.00
ENSSSCG00000008758	SLC34A2	3.83	-5.39	1.45	-3.72	0.00	0.00

ENSSSCG00000026958	WNT10A	7.18	-5.30	1.30	-4.07	0.00	0.00
ENSSSCG00000008159	IL1RL1	3.51	-5.27	1.43	-3.68	0.00	0.00
ENSSSCG00000001987	RIPK3	25.04	-5.21	0.78	-6.66	0.00	0.00
ENSSSCG00000003073	LYPD5	12.53	-5.20	1.11	-4.70	0.00	0.00
ENSSSCG00000003069	KCNN4	3.35	-5.20	1.45	-3.58	0.00	0.00
ENSSSCG00000003585	GJB5	3.35	-5.20	1.45	-3.60	0.00	0.00
ENSSSCG00000002980		3.35	-5.20	1.46	-3.57	0.00	0.00
ENSSSCG00000039101	C1QL1	6.35	-5.12	1.34	-3.82	0.00	0.00
ENSSSCG00000029761		3.17	-5.12	1.45	-3.52	0.00	0.00
ENSSSCG00000024666		3.16	-5.12	1.46	-3.51	0.00	0.00
ENSSSCG00000005890	ZNF7	23.55	-5.11	0.79	-6.51	0.00	0.00
ENSSSCG00000024568		22.72	-5.06	0.79	-6.38	0.00	0.00
ENSSSCG00000003481	PADI1	287.54	-5.05	0.22	-22.54	0.00	0.00
ENSSSCG00000022865	GRM8	3.01	-5.05	1.47	-3.44	0.00	0.00
ENSSSCG00000010312	PLAU	4509.97	-5.05	0.07	-69.21	0.00	0.00
ENSSSCG00000032023		3.01	-5.04	1.46	-3.46	0.00	0.00
ENSSSCG00000009434	RGCC	74.94	-4.97	0.43	-11.70	0.00	0.00
ENSSSCG00000040416		2.85	-4.96	1.48	-3.35	0.00	0.00
ENSSSCG00000007486	CYP24A1	5.51	-4.91	1.34	-3.67	0.00	0.00
ENSSSCG00000012151	NHS	2.68	-4.88	1.52	-3.21	0.00	0.00
ENSSSCG00000038818		2.68	-4.87	1.49	-3.26	0.00	0.00
ENSSSCG00000014952	IZUMO1R	2.67	-4.87	1.50	-3.26	0.00	0.00
ENSSSCG00000035598	EDN1	10.00	-4.87	1.14	-4.26	0.00	0.00
ENSSSCG00000032341	AIF1	19.39	-4.82	0.80	-6.02	0.00	0.00
ENSSSCG00000000854		4.84	-4.72	1.36	-3.48	0.00	0.00

ENSSSCG00000003047		505.50	-4.71	0.16	-29.15	0.00	0.00
ENSSSCG00000002737	CHST4	35.75	-4.70	0.57	-8.28	0.00	0.00
ENSSSCG00000037184	GPR4	52.62	-4.67	0.47	-10.04	0.00	0.00
ENSSSCG00000016239	NYAP2	4.68	-4.67	1.37	-3.41	0.00	0.00
ENSSSCG00000036871	EXOC3L4	211.74	-4.65	0.23	-20.00	0.00	0.00
ENSSSCG00000023165	SEMA7A	30.20	-4.65	0.61	-7.58	0.00	0.00
ENSSSCG00000004733	PLA2G4D	2.18	-4.58	1.59	-2.88	0.00	0.01
ENSSSCG00000012789	AVPR2	2.18	-4.58	1.58	-2.90	0.00	0.01
ENSSSCG00000035077	INHBA	2.17	-4.57	1.56	-2.94	0.00	0.01
ENSSSCG00000025052	BPIFB6	2.17	-4.57	1.56	-2.93	0.00	0.01
ENSSSCG00000015403	HGF	2.17	-4.57	1.58	-2.90	0.00	0.01
ENSSSCG00000036566	LY6G6C	2.17	-4.57	1.58	-2.89	0.00	0.01
ENSSSCG00000005979	ANXA13	1549.04	-4.57	0.09	-49.38	0.00	0.00
ENSSSCG00000012485	NOX1	4.35	-4.56	1.38	-3.31	0.00	0.00
ENSSSCG00000017466	CCR7	4.34	-4.55	1.41	-3.24	0.00	0.00
ENSSSCG00000034154	WNK2	2.00	-4.46	1.60	-2.79	0.01	0.01
ENSSSCG00000039364		2.00	-4.46	1.59	-2.81	0.01	0.01
ENSSSCG00000009865	TBX3	2.00	-4.45	1.63	-2.74	0.01	0.01
ENSSSCG00000026945	LRRC2	2.00	-4.45	1.63	-2.74	0.01	0.01
ENSSSCG00000002891	FFAR3	14.87	-4.43	0.82	-5.40	0.00	0.00
ENSSSCG00000000699	LPAR5	77.00	-4.41	0.36	-12.23	0.00	0.00
ENSSSCG00000013385	INSC	1.84	-4.34	1.66	-2.62	0.01	0.02
ENSSSCG00000032902	KLK7	1.84	-4.34	1.64	-2.65	0.01	0.01
ENSSSCG00000038842	PCDH9	1.84	-4.33	1.62	-2.68	0.01	0.01
ENSSSCG00000039875	NKD1	1.84	-4.33	1.63	-2.66	0.01	0.01

ENSSSCG00000027331	COL6A3	10.36	-4.32	0.96	-4.48	0.00	0.00
ENSSSCG00000011208	ZNF385D	6.86	-4.31	1.19	-3.61	0.00	0.00
ENSSSCG00000021473		3.67	-4.30	1.41	-3.05	0.00	0.00
ENSSSCG00000016999	TLX3	19.72	-4.24	0.68	-6.26	0.00	0.00
ENSSSCG00000005109	ESR2	1.67	-4.20	1.67	-2.52	0.01	0.02
ENSSSCG00000036896	RF00026	1.67	-4.20	1.66	-2.53	0.01	0.02
ENSSSCG00000030694	PKP1	1.67	-4.19	1.67	-2.51	0.01	0.02
ENSSSCG00000032079		1.67	-4.19	1.66	-2.52	0.01	0.02
ENSSSCG00000030505	PLEKHN1	3.34	-4.16	1.43	-2.90	0.00	0.01
ENSSSCG00000009314	FLT3	142.62	-4.12	0.25	-16.65	0.00	0.00
ENSSSCG00000024048	MMRN2	6.03	-4.10	1.18	-3.46	0.00	0.00
ENSSSCG00000031977	PTCH2	1.51	-4.05	1.75	-2.32	0.02	0.03
ENSSSCG00000012733		1.51	-4.05	1.72	-2.36	0.02	0.03
ENSSSCG00000017095	SEMA5A	1.51	-4.05	1.72	-2.36	0.02	0.03
ENSSSCG00000033456	GPR157	1.51	-4.05	1.72	-2.36	0.02	0.03
ENSSSCG00000004584	GCNT3	8.69	-4.05	0.97	-4.16	0.00	0.00
ENSSSCG00000017500	TCAP	1.50	-4.04	1.72	-2.35	0.02	0.03
ENSSSCG00000007463	PTGIS	1.50	-4.04	1.75	-2.31	0.02	0.03
ENSSSCG00000021910	GAST	1.50	-4.04	1.71	-2.37	0.02	0.03
ENSSSCG00000008873	GASK1B	1.50	-4.04	1.71	-2.37	0.02	0.03
ENSSSCG00000002821	CCL22	3.01	-4.00	1.46	-2.74	0.01	0.01
ENSSSCG00000032690		2.99	-4.00	1.46	-2.73	0.01	0.01
ENSSSCG00000031143		8.53	-4.00	1.68	-2.38	0.02	0.03
ENSSSCG00000033878	RAB38	2.98	-3.99	1.50	-2.67	0.01	0.01
ENSSSCG00000002684	CDH13	11.20	-3.99	0.85	-4.69	0.00	0.00

ENSSSCG00000034282	ABCA12	30.08	-3.96	0.52	-7.67	0.00	0.00
ENSSSCG00000032434	PLAUR	246.18	-3.91	0.18	-21.42	0.00	0.00
ENSSSCG00000023684	MT1A	58.26	-3.91	0.37	-10.71	0.00	0.00
ENSSSCG00000035583	PKDREJ	5.15	-3.88	1.22	-3.18	0.00	0.00
ENSSSCG00000004225	TPD52L1	5.18	-3.88	1.21	-3.20	0.00	0.00
ENSSSCG00000006169	ZFHX4	1.34	-3.88	1.78	-2.18	0.03	0.05
ENSSSCG00000038764	RF00026	1.34	-3.88	1.78	-2.18	0.03	0.05
ENSSSCG00000023305		1.34	-3.88	1.78	-2.18	0.03	0.05
ENSSSCG00000003721	CHST9	1.33	-3.87	1.78	-2.17	0.03	0.05
ENSSSCG00000033070		5.18	-3.87	1.22	-3.18	0.00	0.00
ENSSSCG00000033256		10.16	-3.86	0.87	-4.46	0.00	0.00
ENSSSCG00000007007	IDO1	2.68	-3.82	1.53	-2.50	0.01	0.02
ENSSSCG00000003669	MFSD2A	274.66	-3.79	0.17	-22.62	0.00	0.00
ENSSSCG00000001798	BNC1	119.03	-3.75	0.25	-14.95	0.00	0.00
ENSSSCG00000015850	DUSP4	499.10	-3.74	0.13	-29.79	0.00	0.00
ENSSSCG00000023630	CPM	195.98	-3.74	0.19	-19.22	0.00	0.00
ENSSSCG00000000521	PHLDA1	1063.65	-3.72	0.09	-39.58	0.00	0.00
ENSSSCG00000013425	MISP	112.28	-3.72	0.25	-14.70	0.00	0.00
ENSSSCG00000011793	LIPH	2.51	-3.72	1.51	-2.47	0.01	0.02
ENSSSCG00000011734	ARL14	2.50	-3.72	1.51	-2.46	0.01	0.02
ENSSSCG00000039927		1.17	-3.68	1.85	-1.99	0.05	0.07
ENSSSCG00000008134	EDAR	1.17	-3.68	1.82	-2.02	0.04	0.07
ENSSSCG00000019431	ssc-mir-1307	1.17	-3.68	1.82	-2.02	0.04	0.07
ENSSSCG00000026130	EPHA3	1.17	-3.68	1.82	-2.02	0.04	0.07
ENSSSCG00000023162	CDH3	1.17	-3.68	1.84	-2.00	0.05	0.07

ENSSSCG00000031998		1.17	-3.68	1.84	-2.00	0.05	0.07
ENSSSCG00000011047	FAM171A1	1.16	-3.68	1.85	-1.98	0.05	0.07
ENSSSCG00000003709	LAMA3	1037.63	-3.66	0.09	-40.57	0.00	0.00
ENSSSCG00000015326	COL1A2	817.16	-3.58	0.10	-36.61	0.00	0.00
ENSSSCG00000004980	THSD4	85.61	-3.54	0.28	-12.76	0.00	0.00
ENSSSCG00000028214		28.90	-3.52	0.48	-7.40	0.00	0.00
ENSSSCG00000000186	RHEBL1	4.01	-3.48	1.27	-2.74	0.01	0.01
ENSSSCG00000021598	EVA1C	14.02	-3.48	0.68	-5.15	0.00	0.00
ENSSSCG00000035891	ADGRB1	14.05	-3.47	0.69	-5.02	0.00	0.00
ENSSSCG00000000668	APOBEC1	41.11	-3.43	0.39	-8.70	0.00	0.00
ENSSSCG00000012967	FOSL1	414.31	-3.43	0.13	-26.63	0.00	0.00
ENSSSCG00000039868		3.82	-3.42	1.29	-2.65	0.01	0.01
ENSSSCG00000017874	ATP2A3	36.54	-3.41	0.42	-8.10	0.00	0.00
ENSSSCG00000035256		39.64	-3.39	0.40	-8.45	0.00	0.00
ENSSSCG00000028810		5.66	-3.39	1.06	-3.19	0.00	0.00
ENSSSCG00000033726		1.99	-3.37	1.58	-2.14	0.03	0.05
ENSSSCG00000040760		1.99	-3.37	1.59	-2.12	0.03	0.05
ENSSSCG00000022017	LGALS4	5.50	-3.34	1.04	-3.21	0.00	0.00
ENSSSCG00000008595	APOB	43.38	-3.32	0.37	-8.86	0.00	0.00
ENSSSCG00000029592	GPRC5A	1309.57	-3.30	0.08	-40.33	0.00	0.00
ENSSSCG00000015396	SEMA3D	2087.42	-3.29	0.07	-46.94	0.00	0.00
ENSSSCG00000020953		26.72	-3.28	0.48	-6.83	0.00	0.00
ENSSSCG00000022447	F3	1342.38	-3.28	0.08	-42.84	0.00	0.00
ENSSSCG00000007084	BFSP1	60.04	-3.28	0.32	-10.41	0.00	0.00
ENSSSCG00000018698	ssc-mir-221	8.84	-3.28	0.82	-4.02	0.00	0.00

ENSSSCG00000007703	TRIM50	12.15	-3.26	0.71	-4.61	0.00	0.00
ENSSSCG00000023229	ETV5	334.21	-3.25	0.14	-23.72	0.00	0.00
ENSSSCG00000034390	CARD11	254.05	-3.24	0.16	-20.46	0.00	0.00
ENSSSCG00000021899		1.84	-3.23	1.61	-2.02	0.04	0.07
ENSSSCG00000034113		1.82	-3.23	1.63	-1.98	0.05	0.07
ENSSSCG00000028274	FAM131B	1.83	-3.23	1.64	-1.96	0.05	0.07
ENSSSCG00000013940	NLRP3	32.24	-3.21	0.42	-7.57	0.00	0.00
ENSSSCG00000015618	LAMB3	2041.80	-3.19	0.06	-50.09	0.00	0.00
ENSSSCG00000006350		3.35	-3.18	1.30	-2.44	0.01	0.02
ENSSSCG00000036723	EMP1	2460.23	-3.15	0.06	-49.54	0.00	0.00
ENSSSCG00000015839	NRG1	80.09	-3.15	0.27	-11.75	0.00	0.00
ENSSSCG00000001787	IL16	14.68	-3.15	0.62	-5.08	0.00	0.00
ENSSSCG00000000253	KRT18	1011.39	-3.15	0.08	-38.68	0.00	0.00
ENSSSCG00000001595	DAAM2	3.15	-3.12	1.33	-2.34	0.02	0.03
ENSSSCG00000002241	NUTM1	3.15	-3.12	1.33	-2.34	0.02	0.03
ENSSSCG00000002908	ZBTB32	6.34	-3.10	0.94	-3.31	0.00	0.00
ENSSSCG00000025529	FAM3D	3.19	-3.10	1.33	-2.32	0.02	0.03
ENSSSCG00000000234	GRASP	3.19	-3.10	1.33	-2.32	0.02	0.03
ENSSSCG00000005475		3.19	-3.09	1.33	-2.33	0.02	0.03
ENSSSCG00000006472	CRABP2	13.87	-3.06	0.66	-4.64	0.00	0.00
ENSSSCG00000017552	NXPH3	15.22	-3.03	0.61	-5.00	0.00	0.00
ENSSSCG00000008259	LRRTM4	3.01	-3.03	1.43	-2.11	0.03	0.05
ENSSSCG00000020188	RF00090	7.51	-3.02	0.85	-3.56	0.00	0.00
ENSSSCG00000013501	CREB3L3	49.19	-3.01	0.33	-9.15	0.00	0.00
ENSSSCG00000024134	MGLL	767.83	-2.98	0.09	-32.43	0.00	0.00

ENSSSCG00000036008	TACR1	118.45	-2.97	0.21	-14.03	0.00	0.00
ENSSSCG00000002135	PNP	5340.89	-2.95	0.06	-48.48	0.00	0.00
ENSSSCG00000040155		2.83	-2.93	1.34	-2.18	0.03	0.05
ENSSSCG00000036056	DNAH17	2.83	-2.92	1.34	-2.18	0.03	0.05
ENSSSCG00000006233	CA8	34.02	-2.92	0.39	-7.51	0.00	0.00
ENSSSCG00000017515	TBKBP1	5.68	-2.92	0.95	-3.08	0.00	0.00
ENSSSCG00000012678		2.84	-2.91	1.36	-2.15	0.03	0.05
ENSSSCG00000033657	GREM1	5139.04	-2.90	0.06	-51.74	0.00	0.00
ENSSSCG00000017578	ITGA3	36239.51	-2.89	0.04	-79.49	0.00	0.00
ENSSSCG00000013505	FSD1	32.04	-2.89	0.40	-7.27	0.00	0.00
ENSSSCG00000026890	GALNT6	110.69	-2.89	0.22	-13.11	0.00	0.00
ENSSSCG00000017548	NGFR	4.18	-2.88	1.13	-2.56	0.01	0.02
ENSSSCG00000017892	PITPNM3	57.29	-2.86	0.30	-9.51	0.00	0.00
ENSSSCG00000002512	DEGS2	12.19	-2.86	0.69	-4.16	0.00	0.00
ENSSSCG00000015366	PRPS1L1	2.66	-2.85	1.39	-2.05	0.04	0.06
ENSSSCG00000014233		2.65	-2.84	1.39	-2.05	0.04	0.06
ENSSSCG00000011436		5.31	-2.84	0.97	-2.91	0.00	0.01
ENSSSCG00000019245	RF00213	2.67	-2.83	1.37	-2.07	0.04	0.06
ENSSSCG00000013115	CD5	2.68	-2.81	1.38	-2.04	0.04	0.06
ENSSSCG00000017472	IGFBP4	197.94	-2.78	0.16	-17.09	0.00	0.00
ENSSSCG00000038455	RBM24	6.52	-2.78	0.89	-3.14	0.00	0.00
ENSSSCG00000013880		83.04	-2.78	0.24	-11.40	0.00	0.00
ENSSSCG00000006391	ATP1A2	31.01	-2.78	0.41	-6.84	0.00	0.00
ENSSSCG00000008006	FBXL16	11.68	-2.77	0.65	-4.26	0.00	0.00
ENSSSCG00000017982	HES7	3.82	-2.76	1.13	-2.44	0.01	0.02

ENSSSCG00000015136	UBASH3B	1085.88	-2.75	0.08	-33.00	0.00	0.00
ENSSSCG00000013092	PGA5	13.98	-2.75	0.60	-4.58	0.00	0.00
ENSSSCG00000006970	DLC1	756.96	-2.74	0.09	-31.06	0.00	0.00
ENSSSCG00000038677	GJB3	53.90	-2.72	0.31	-8.87	0.00	0.00
ENSSSCG00000017379	ETV4	711.44	-2.70	0.09	-28.86	0.00	0.00
ENSSSCG00000006651	ADAMTSL4	640.45	-2.69	0.09	-28.80	0.00	0.00
ENSSSCG00000003278		9.87	-2.69	0.71	-3.77	0.00	0.00
ENSSSCG00000014310	CXCL14	7.33	-2.68	0.80	-3.34	0.00	0.00
ENSSSCG00000003600	TINAGL1	2357.12	-2.67	0.06	-43.91	0.00	0.00
ENSSSCG00000023743	CLDN6	25.32	-2.66	0.44	-6.08	0.00	0.00
ENSSSCG00000036377	OVOL2	28.80	-2.66	0.41	-6.43	0.00	0.00
ENSSSCG00000031788	PTAFR	56.95	-2.63	0.29	-9.08	0.00	0.00
ENSSSCG00000015556	LAMC2	2263.49	-2.63	0.06	-46.32	0.00	0.00
ENSSSCG00000037215	ZNF3	10.68	-2.62	0.66	-3.95	0.00	0.00
ENSSSCG00000010475	CYP26A1	143.61	-2.62	0.19	-14.13	0.00	0.00
ENSSSCG00000001572	FGD2	3.49	-2.61	1.16	-2.25	0.02	0.04
ENSSSCG00000007032	PLAT	936.48	-2.61	0.08	-32.62	0.00	0.00
ENSSSCG00000006734	CD101	8.17	-2.60	0.75	-3.47	0.00	0.00
ENSSSCG00000004572		47.60	-2.60	0.32	-8.01	0.00	0.00
ENSSSCG00000027030	BDKRB2	9.36	-2.59	0.70	-3.68	0.00	0.00
ENSSSCG00000031118	PREX1	719.56	-2.58	0.09	-28.46	0.00	0.00
ENSSSCG00000023181	ZNF385B	6.85	-2.56	0.85	-3.02	0.00	0.00
ENSSSCG00000012950	RIN1	315.03	-2.54	0.13	-19.32	0.00	0.00
ENSSSCG00000030300		235.58	-2.53	0.15	-16.38	0.00	0.00
ENSSSCG00000028373	ENO2	3.32	-2.53	1.17	-2.16	0.03	0.05

ENSSSCG00000029230	ECM1	314.59	-2.53	0.13	-19.65	0.00	0.00
ENSSSCG00000017008	DOCK2	3.36	-2.51	1.17	-2.14	0.03	0.05
ENSSSCG00000015398	SEMA3A	7.66	-2.50	0.77	-3.24	0.00	0.00
ENSSSCG00000001565	CDKN1A	151.55	-2.50	0.18	-13.95	0.00	0.00
ENSSSCG00000010607	COL17A1	1996.40	-2.49	0.06	-42.55	0.00	0.00
ENSSSCG00000032178		5.53	-2.49	1.07	-2.32	0.02	0.03
ENSSSCG00000006858	OLFM3	26.04	-2.47	0.43	-5.78	0.00	0.00
ENSSSCG00000012652	SASH3	9.66	-2.47	0.68	-3.61	0.00	0.00
ENSSSCG00000040689	APOA4	7.51	-2.46	0.77	-3.21	0.00	0.00
ENSSSCG00000024351	MET	9117.58	-2.46	0.05	-47.57	0.00	0.00
ENSSSCG00000008963	AREG	756.02	-2.45	0.09	-28.54	0.00	0.00
ENSSSCG00000038148	PROKR1	3.15	-2.44	1.20	-2.03	0.04	0.07
ENSSSCG00000021944	RAC2	15.87	-2.43	0.53	-4.57	0.00	0.00
ENSSSCG00000037132	POU2F2	3.18	-2.42	1.18	-2.05	0.04	0.06
ENSSSCG00000032980		29.03	-2.40	0.39	-6.13	0.00	0.00
ENSSSCG00000006309	CD247	5.17	-2.40	0.91	-2.63	0.01	0.01
ENSSSCG00000000709	PLEKHG6	62.03	-2.39	0.27	-8.96	0.00	0.00
ENSSSCG00000010892	KCNT2	5.17	-2.39	0.93	-2.56	0.01	0.02
ENSSSCG00000001097		38.02	-2.38	0.34	-7.02	0.00	0.00
ENSSSCG00000015287	TMCC2	18.31	-2.37	0.49	-4.85	0.00	0.00
ENSSSCG00000021453	RIMS4	14.19	-2.36	0.56	-4.20	0.00	0.00
ENSSSCG00000004509	LIPG	10489.50	-2.35	0.05	-48.28	0.00	0.00
ENSSSCG00000038185	EREG	5190.07	-2.33	0.05	-44.22	0.00	0.00
ENSSSCG00000021536	CLDN9	50.88	-2.33	0.29	-7.99	0.00	0.00
ENSSSCG00000007453	EYA2	12.03	-2.33	0.61	-3.80	0.00	0.00

ENSSSCG00000027607	IER3	663.17	-2.32	0.09	-25.09	0.00	0.00
ENSSSCG00000026587	BATF3	121.72	-2.32	0.19	-11.91	0.00	0.00
ENSSSCG00000011260	SCN10A	9.83	-2.30	0.68	-3.36	0.00	0.00
ENSSSCG00000040207	P2RY2	965.63	-2.30	0.08	-29.98	0.00	0.00
ENSSSCG00000032656	BRSK1	9.86	-2.30	0.66	-3.47	0.00	0.00
ENSSSCG00000017380	ARL4D	4.81	-2.30	0.97	-2.37	0.02	0.03
ENSSSCG00000013378	ABCC8	7.87	-2.29	0.76	-3.01	0.00	0.00
ENSSSCG00000015232	ST3GAL4	742.74	-2.29	0.08	-27.37	0.00	0.00
ENSSSCG00000040893	ZNF488	3.82	-2.28	1.09	-2.09	0.04	0.06
ENSSSCG00000002781	KCTD19	5.85	-2.27	0.89	-2.56	0.01	0.02
ENSSSCG00000037524	LY6D	67.33	-2.27	0.26	-8.81	0.00	0.00
ENSSSCG00000019600	RF00265	3.85	-2.26	1.06	-2.13	0.03	0.05
ENSSSCG00000024588		35.34	-2.25	0.35	-6.42	0.00	0.00
ENSSSCG00000036904	SMAGP	393.61	-2.25	0.11	-20.86	0.00	0.00
ENSSSCG00000035772	CDH5	233.99	-2.25	0.14	-15.97	0.00	0.00
ENSSSCG00000024823	RCN3	399.09	-2.22	0.11	-19.75	0.00	0.00
ENSSSCG00000027677		10.33	-2.22	0.64	-3.46	0.00	0.00
ENSSSCG00000022496	STK39	580.50	-2.22	0.10	-22.19	0.00	0.00
ENSSSCG00000033293		6.49	-2.21	0.81	-2.73	0.01	0.01
ENSSSCG00000014072	ENC1	296.90	-2.21	0.12	-17.68	0.00	0.00
ENSSSCG00000008147	FHL2	3059.82	-2.20	0.06	-38.23	0.00	0.00
ENSSSCG00000020439	RF00009	5.47	-2.20	0.88	-2.50	0.01	0.02
ENSSSCG00000037252		15.69	-2.19	0.63	-3.46	0.00	0.00
ENSSSCG00000028053	RF00325	3.66	-2.19	1.07	-2.04	0.04	0.06
ENSSSCG00000036036	XG	29.82	-2.16	0.37	-5.78	0.00	0.00

ENSSSCG00000003561	ZDHHC18	1956.50	-2.16	0.06	-37.13	0.00	0.00
ENSSSCG00000023803	ELK3	359.93	-2.16	0.11	-19.30	0.00	0.00
ENSSSCG00000010339	DYDC2	24.37	-2.15	0.41	-5.20	0.00	0.00
ENSSSCG00000021902	GABRP	18.85	-2.15	0.47	-4.56	0.00	0.00
ENSSSCG00000028530	TMEM52	168.19	-2.15	0.16	-13.33	0.00	0.00
ENSSSCG00000022741	PDGFRB	11.66	-2.15	0.60	-3.58	0.00	0.00
ENSSSCG00000035058	PID1	7.18	-2.15	0.78	-2.74	0.01	0.01
ENSSSCG00000006466	SH2D2A	260.07	-2.14	0.13	-16.58	0.00	0.00
ENSSSCG00000033521		115.92	-2.14	0.19	-11.16	0.00	0.00
ENSSSCG00000015953	DLX1	53.30	-2.13	0.28	-7.52	0.00	0.00
ENSSSCG00000015664		422.41	-2.12	0.11	-19.84	0.00	0.00
ENSSSCG00000040919	RTN4RL2	76.47	-2.11	0.24	-8.96	0.00	0.00
ENSSSCG00000033539	CRYBG2	101.34	-2.09	0.21	-10.06	0.00	0.00
ENSSSCG00000031295	SMCO4	54.60	-2.09	0.28	-7.53	0.00	0.00
ENSSSCG00000031232	RF00271	14.61	-2.09	0.55	-3.80	0.00	0.00
ENSSSCG00000022925		289.68	-2.08	0.12	-16.99	0.00	0.00
ENSSSCG00000038610	INHBB	487.76	-2.08	0.10	-20.93	0.00	0.00
ENSSSCG00000011489		641.09	-2.07	0.09	-22.79	0.00	0.00
ENSSSCG00000036768	PRAG1	71.03	-2.06	0.24	-8.57	0.00	0.00
ENSSSCG00000010604	SH3PXD2A	652.65	-2.06	0.09	-22.66	0.00	0.00
ENSSSCG00000002383	FOS	374.51	-2.06	0.11	-18.68	0.00	0.00
ENSSSCG00000003509	SH2D5	211.75	-2.06	0.15	-14.03	0.00	0.00
ENSSSCG00000010608	SFR1	255.12	-2.05	0.13	-15.76	0.00	0.00
ENSSSCG00000017879		1033.61	-2.05	0.07	-29.35	0.00	0.00
ENSSSCG00000001727	TNFRSF21	990.77	-2.04	0.07	-27.75	0.00	0.00

ENSSSCG00000003451		1746.24	-2.04	0.07	-28.63	0.00	0.00
ENSSSCG00000022380	SH2D3A	140.17	-2.04	0.17	-11.83	0.00	0.00
ENSSSCG00000008729	LYAR	1866.94	-2.03	0.06	-33.80	0.00	0.00
ENSSSCG00000005981	FBXO32	832.80	-2.03	0.08	-26.24	0.00	0.00
ENSSSCG00000039204	SOST	14.10	-2.03	0.55	-3.67	0.00	0.00
ENSSSCG00000004241	GJA1	244.09	-2.02	0.14	-14.74	0.00	0.00
ENSSSCG00000040698	PRR7	64.69	-2.02	0.26	-7.73	0.00	0.00
ENSSSCG00000004094	PPP1R14C	14.16	-2.02	0.53	-3.82	0.00	0.00
ENSSSCG00000040815	DUSP5	1020.15	-2.01	0.08	-26.45	0.00	0.00
ENSSSCG00000016887	ITGA2	4037.88	-2.01	0.05	-41.94	0.00	0.00
ENSSSCG00000003684	MTCL1	895.07	-2.00	0.07	-26.93	0.00	0.00
ENSSSCG00000005986	FAM83A	187.49	-2.00	0.15	-13.19	0.00	0.00
ENSSSCG00000006688	ANKRD35	20.47	-1.99	0.44	-4.53	0.00	0.00
ENSSSCG00000002662	C16orf74	101.05	-1.99	0.20	-9.81	0.00	0.00
ENSSSCG00000003973	CTPS1	1833.88	-1.98	0.06	-34.51	0.00	0.00
ENSSSCG00000030388	UPP1	454.16	-1.97	0.10	-20.08	0.00	0.00
ENSSSCG00000016175	MREG	375.56	-1.96	0.12	-16.65	0.00	0.00
ENSSSCG00000035867	GFOD1	146.29	-1.96	0.17	-11.82	0.00	0.00
ENSSSCG00000022370	TNFSF9	201.82	-1.95	0.15	-12.88	0.00	0.00
ENSSSCG00000033018	TM4SF1	6402.77	-1.95	0.04	-46.08	0.00	0.00
ENSSSCG00000025698	SERPINE1	1390.61	-1.95	0.07	-28.64	0.00	0.00
ENSSSCG00000016614	PTPRZ1	1610.27	-1.94	0.06	-31.74	0.00	0.00
ENSSSCG00000005620	SH2D3C	19.16	-1.94	0.45	-4.32	0.00	0.00
ENSSSCG00000023351	PLA2G4A	68.06	-1.94	0.24	-7.93	0.00	0.00
ENSSSCG00000005135		3539.30	-1.94	0.05	-38.59	0.00	0.00

ENSSSCG00000012408	NHSL2	186.94	-1.93	0.15	-12.73	0.00	0.00
ENSSSCG00000032242	HMGA1	4391.56	-1.93	0.05	-37.50	0.00	0.00
ENSSSCG00000023522	TGM2	328.76	-1.92	0.12	-16.28	0.00	0.00
ENSSSCG00000029037	DRD1	8.66	-1.92	0.67	-2.88	0.00	0.01
ENSSSCG00000017199		307.97	-1.91	0.12	-15.96	0.00	0.00
ENSSSCG00000023737	CSF2	26.62	-1.91	0.40	-4.82	0.00	0.00
ENSSSCG00000034754	PLEKHS1	476.11	-1.91	0.10	-19.67	0.00	0.00
ENSSSCG00000038459	SURF2	839.12	-1.91	0.08	-25.49	0.00	0.00
ENSSSCG00000003928	PLK3	167.88	-1.91	0.16	-11.61	0.00	0.00
ENSSSCG00000033189	FAM107A	67.89	-1.90	0.25	-7.65	0.00	0.00
ENSSSCG00000038806	AXL	972.33	-1.90	0.08	-25.31	0.00	0.00
ENSSSCG00000037070	GRID2IP	5.54	-1.90	0.87	-2.19	0.03	0.05
ENSSSCG00000003768	NEXN	826.82	-1.89	0.08	-22.44	0.00	0.00
ENSSSCG00000040359	GOLGA7B	70.47	-1.89	0.24	-7.77	0.00	0.00
ENSSSCG00000011618	GATA2	11.69	-1.89	0.59	-3.19	0.00	0.00
ENSSSCG00000017265	AXIN2	72.78	-1.88	0.23	-8.13	0.00	0.00
ENSSSCG00000010012	SLC35E4	93.57	-1.88	0.21	-8.88	0.00	0.00
ENSSSCG00000015662	C4BPA	8.51	-1.88	0.67	-2.81	0.00	0.01
ENSSSCG00000005437		552.29	-1.87	0.10	-19.69	0.00	0.00
ENSSSCG00000016810	PDZD2	87.26	-1.86	0.22	-8.64	0.00	0.00
ENSSSCG00000026454	PMAIP1	204.59	-1.86	0.14	-13.00	0.00	0.00
ENSSSCG00000027991		143.87	-1.85	0.17	-11.22	0.00	0.00
ENSSSCG00000006187	MSC	15.10	-1.85	0.51	-3.63	0.00	0.00
ENSSSCG00000001989	CIDEB	36.46	-1.85	0.32	-5.74	0.00	0.00
ENSSSCG00000016233	SERPINE2	554.90	-1.85	0.10	-18.40	0.00	0.00

ENSSSCG00000038698	RF00614	6.82	-1.85	0.74	-2.49	0.01	0.02
ENSSSCG00000003017	TGFB1	2182.45	-1.85	0.05	-34.12	0.00	0.00
ENSSSCG00000027735		10.67	-1.85	0.61	-3.01	0.00	0.00
ENSSSCG00000038508	SPTBN2	279.23	-1.85	0.12	-15.15	0.00	0.00
ENSSSCG00000037645	COTL1	4011.29	-1.84	0.05	-39.70	0.00	0.00
ENSSSCG00000033005	RF00309	6.82	-1.84	0.76	-2.44	0.01	0.02
ENSSSCG00000020701	TTC9	338.93	-1.84	0.11	-16.41	0.00	0.00
ENSSSCG00000003832	TACSTD2	305.84	-1.84	0.13	-14.42	0.00	0.00
ENSSSCG00000020791	RF00603	5.34	-1.84	0.87	-2.12	0.03	0.05
ENSSSCG00000006477	BCAN	12.85	-1.84	0.56	-3.27	0.00	0.00
ENSSSCG00000014450	TCOF1	2166.71	-1.84	0.06	-31.13	0.00	0.00
ENSSSCG00000032749	PCDH18	50.45	-1.84	0.29	-6.37	0.00	0.00
ENSSSCG00000036155	FAT4	158.78	-1.83	0.16	-11.65	0.00	0.00
ENSSSCG00000027689		1878.94	-1.83	0.06	-29.53	0.00	0.00
ENSSSCG00000004894	SERPINB5	3873.19	-1.83	0.05	-37.26	0.00	0.00
ENSSSCG00000011440	SEMA3G	112.21	-1.82	0.20	-9.13	0.00	0.00
ENSSSCG00000033207	SULT2A1	11.17	-1.81	0.58	-3.11	0.00	0.00
ENSSSCG00000038618	RFLNB	90.66	-1.81	0.21	-8.75	0.00	0.00
ENSSSCG00000026849	CCNO	6.68	-1.80	0.75	-2.39	0.02	0.03
ENSSSCG00000037016	ID1	559.35	-1.80	0.10	-18.67	0.00	0.00
ENSSSCG00000012409	CITED1	28.00	-1.79	0.37	-4.79	0.00	0.00
ENSSSCG00000033260	TNNI2	17.62	-1.79	0.47	-3.85	0.00	0.00
ENSSSCG00000020837		1774.74	-1.78	0.06	-28.90	0.00	0.00
ENSSSCG00000009833	SH2B3	470.90	-1.78	0.10	-18.01	0.00	0.00
ENSSSCG00000011703	TM4SF4	6937.63	-1.78	0.04	-40.07	0.00	0.00

ENSSSCG00000017178	SPHK1	389.87	-1.78	0.10	-17.27	0.00	0.00
ENSSSCG00000007305	SPAG4	5.81	-1.78	0.82	-2.17	0.03	0.05
ENSSSCG00000010477	CEP55	855.04	-1.77	0.08	-20.98	0.00	0.00
ENSSSCG00000018058	ARHGAP23	512.42	-1.77	0.10	-17.76	0.00	0.00
ENSSSCG00000038404	SERPINB6	977.59	-1.76	0.07	-24.98	0.00	0.00
ENSSSCG00000012386	FAM155B	1224.97	-1.76	0.07	-25.97	0.00	0.00
ENSSSCG00000011579	PPARG	1501.67	-1.76	0.07	-26.62	0.00	0.00
ENSSSCG00000017275	NOL11	1300.09	-1.75	0.06	-27.53	0.00	0.00
ENSSSCG00000023362	RHBDF2	1029.73	-1.75	0.07	-25.48	0.00	0.00
ENSSSCG00000014391	ARAP3	667.78	-1.74	0.09	-19.73	0.00	0.00
ENSSSCG00000012160	SH3KBP1	482.47	-1.74	0.09	-18.33	0.00	0.00
ENSSSCG00000017605	MMD	222.04	-1.74	0.13	-12.92	0.00	0.00
ENSSSCG00000023441	KLHDC8A	4.99	-1.74	0.88	-1.98	0.05	0.07
ENSSSCG00000017384	AOC2	4.99	-1.74	0.87	-2.00	0.05	0.07
ENSSSCG00000022483	GPBAR1	29.68	-1.73	0.38	-4.61	0.00	0.00
ENSSSCG00000038948	ETS2	352.01	-1.73	0.11	-15.88	0.00	0.00
ENSSSCG00000026068	GRHL1	30.00	-1.73	0.35	-4.93	0.00	0.00
ENSSSCG00000017934		111.69	-1.73	0.19	-9.09	0.00	0.00
ENSSSCG00000033537		404.20	-1.73	0.10	-16.77	0.00	0.00
ENSSSCG00000015550	RGS16	7.81	-1.73	0.70	-2.48	0.01	0.02
ENSSSCG00000040377	FCER2	32.66	-1.72	0.34	-5.12	0.00	0.00
ENSSSCG00000032328	MAP6D1	126.59	-1.72	0.18	-9.72	0.00	0.00
ENSSSCG00000003818	DOCK7	4060.53	-1.72	0.05	-37.75	0.00	0.00
ENSSSCG00000002294	ARG2	665.76	-1.72	0.09	-19.59	0.00	0.00
ENSSSCG00000028076	ZBTB7C	5.67	-1.72	0.84	-2.05	0.04	0.06

ENSSSCG00000014989	DCUN1D5	942.88	-1.71	0.07	-23.76	0.00	0.00
ENSSSCG00000021597	PHLDA2	1396.46	-1.71	0.08	-21.51	0.00	0.00
ENSSSCG00000021359	CDC42EP3	667.83	-1.70	0.08	-20.17	0.00	0.00
ENSSSCG00000016635	CAV2	1414.85	-1.70	0.08	-22.08	0.00	0.00
ENSSSCG00000013300	EHF	386.06	-1.69	0.11	-15.54	0.00	0.00
ENSSSCG00000039703	EEPD1	29.71	-1.68	0.37	-4.53	0.00	0.00
ENSSSCG00000033120	PALM2	1403.56	-1.68	0.06	-27.75	0.00	0.00
ENSSSCG00000006987	SLC7A2	506.09	-1.68	0.09	-17.70	0.00	0.00
ENSSSCG00000038765	FAM89A	78.87	-1.68	0.22	-7.48	0.00	0.00
ENSSSCG00000011730	IL12A	9.63	-1.68	0.62	-2.69	0.01	0.01
ENSSSCG00000021027	PGBD5	63.93	-1.67	0.25	-6.65	0.00	0.00
ENSSSCG00000024060	LIPT2	54.04	-1.67	0.27	-6.27	0.00	0.00
ENSSSCG00000009739	NOC4L	1110.54	-1.66	0.07	-25.01	0.00	0.00
ENSSSCG00000034501	RIOX2	1295.99	-1.66	0.06	-26.56	0.00	0.00
ENSSSCG00000023054	IDO2	6.83	-1.66	0.79	-2.09	0.04	0.06
ENSSSCG00000011217	NEK10	90.23	-1.66	0.21	-7.82	0.00	0.00
ENSSSCG00000009412	LCP1	105.83	-1.66	0.19	-8.67	0.00	0.00
ENSSSCG00000006235	TOX	271.45	-1.66	0.13	-12.94	0.00	0.00
ENSSSCG00000039419	SLCO4A1	658.29	-1.66	0.08	-19.58	0.00	0.00
ENSSSCG00000032613	SNAI1	71.10	-1.65	0.23	-7.19	0.00	0.00
ENSSSCG00000009937	ACACB	6.83	-1.65	0.74	-2.24	0.03	0.04
ENSSSCG00000031166		109.19	-1.65	0.20	-8.33	0.00	0.00
ENSSSCG00000017995	USP43	464.57	-1.65	0.10	-17.14	0.00	0.00
ENSSSCG00000033780	B4GALNT4	387.77	-1.65	0.11	-15.69	0.00	0.00
ENSSSCG00000035262	TMEM74B	16.96	-1.65	0.47	-3.52	0.00	0.00

ENSSSCG00000017164	TIMP2	280.87	-1.65	0.12	-13.54	0.00	0.00
ENSSSCG00000013049	RCOR2	317.94	-1.64	0.11	-14.33	0.00	0.00
ENSSSCG00000023662	CHST3	55.13	-1.64	0.26	-6.23	0.00	0.00
ENSSSCG00000012087	SLC37A1	696.28	-1.63	0.08	-20.63	0.00	0.00
ENSSSCG00000022151	B3GALT6	96.76	-1.63	0.20	-7.98	0.00	0.00
ENSSSCG00000000010	FBLN1	10.87	-1.63	0.60	-2.71	0.01	0.01
ENSSSCG00000008898		14.80	-1.63	0.51	-3.21	0.00	0.00
ENSSSCG00000003018	LIPE	128.86	-1.62	0.17	-9.46	0.00	0.00
ENSSSCG00000019224	RF00606	16.85	-1.62	0.48	-3.36	0.00	0.00
ENSSSCG00000006774	PPM1J	25.52	-1.61	0.39	-4.17	0.00	0.00
ENSSSCG00000033998	SLC43A3	2369.01	-1.61	0.06	-26.30	0.00	0.00
ENSSSCG00000002718	FA2H	431.75	-1.60	0.11	-14.60	0.00	0.00
ENSSSCG00000000743	FKBP4	3425.07	-1.60	0.06	-28.77	0.00	0.00
ENSSSCG00000006842		169.03	-1.60	0.16	-10.31	0.00	0.00
ENSSSCG00000023880	RRP8	1182.69	-1.60	0.07	-22.11	0.00	0.00
ENSSSCG00000016286	PRSS56	8.01	-1.60	0.68	-2.36	0.02	0.03
ENSSSCG00000019194	RF00289	7.35	-1.60	0.71	-2.26	0.02	0.04
ENSSSCG00000014934	CHORDC1	3021.34	-1.60	0.05	-30.59	0.00	0.00
ENSSSCG00000020912	HECTD2	22.44	-1.59	0.41	-3.88	0.00	0.00
ENSSSCG00000027110	RPP38	395.73	-1.59	0.10	-15.21	0.00	0.00
ENSSSCG00000028355	ACTG1	143912.68	-1.58	0.04	-43.00	0.00	0.00
ENSSSCG00000038440	GTPBP3	536.11	-1.58	0.09	-17.61	0.00	0.00
ENSSSCG00000034835	SLC7A6	1406.82	-1.58	0.06	-25.93	0.00	0.00
ENSSSCG00000017236	CD300C	29.44	-1.57	0.35	-4.47	0.00	0.00
ENSSSCG00000036341		67.47	-1.57	0.24	-6.51	0.00	0.00

ENSSSCG00000037319	PDCD1	108.96	-1.56	0.19	-8.32	0.00	0.00
ENSSSCG00000030102	RF00568	22.11	-1.56	0.41	-3.82	0.00	0.00
ENSSSCG00000021220	CKB	1018.86	-1.56	0.07	-22.78	0.00	0.00
ENSSSCG00000013105	CCDC86	895.53	-1.56	0.07	-21.73	0.00	0.00
ENSSSCG00000040581	CISH	97.56	-1.56	0.19	-8.05	0.00	0.00
ENSSSCG00000018032	TRPV2	12.36	-1.55	0.57	-2.72	0.01	0.01
ENSSSCG00000002639		147.10	-1.55	0.16	-9.55	0.00	0.00
ENSSSCG00000036157	BARX2	156.04	-1.55	0.16	-9.86	0.00	0.00
ENSSSCG00000038005		23.98	-1.55	0.39	-4.02	0.00	0.00
ENSSSCG00000038521	CHAC1	208.75	-1.55	0.14	-11.35	0.00	0.00
ENSSSCG00000035314		17.50	-1.55	0.47	-3.32	0.00	0.00
ENSSSCG00000036317	VASP	4200.34	-1.55	0.05	-32.22	0.00	0.00
ENSSSCG00000011226	TGFBR2	9579.39	-1.54	0.04	-35.54	0.00	0.00
ENSSSCG00000040636	KRT80	356.80	-1.54	0.11	-14.11	0.00	0.00
ENSSSCG00000007189	SDCBP2	34.09	-1.54	0.34	-4.55	0.00	0.00
ENSSSCG00000027325	TRNP1	27.62	-1.53	0.37	-4.19	0.00	0.00
ENSSSCG00000015241	TMEM45B	424.56	-1.53	0.10	-15.32	0.00	0.00
ENSSSCG00000017561	ABCC3	5196.75	-1.53	0.04	-34.51	0.00	0.00
ENSSSCG00000015649	DYRK3	379.84	-1.53	0.10	-14.93	0.00	0.00
ENSSSCG00000009622	POLR3D	1609.94	-1.53	0.06	-26.02	0.00	0.00
ENSSSCG00000003848	LRP8	611.81	-1.52	0.08	-18.02	0.00	0.00
ENSSSCG00000014410	SH3RF2	330.11	-1.52	0.11	-14.02	0.00	0.00
ENSSSCG00000009256	ANTXR2	1424.85	-1.52	0.06	-23.95	0.00	0.00
ENSSSCG00000008187	KIAA1211L	126.39	-1.52	0.17	-8.79	0.00	0.00
ENSSSCG00000008427	KCNK12	101.48	-1.52	0.19	-7.93	0.00	0.00

ENSSSCG00000030177	EBNA1BP2	1764.83	-1.52	0.06	-25.45	0.00	0.00
ENSSSCG00000005131	DMRTA1	144.55	-1.51	0.16	-9.49	0.00	0.00
ENSSSCG00000006850	FAM102B	4047.72	-1.51	0.05	-32.64	0.00	0.00
ENSSSCG00000009474	SCEL	58.82	-1.51	0.26	-5.85	0.00	0.00
ENSSSCG00000017427	HAP1	473.00	-1.51	0.09	-16.19	0.00	0.00
ENSSSCG00000015570	IVNS1ABP	4655.67	-1.51	0.04	-34.38	0.00	0.00
ENSSSCG00000017338	PLCD3	458.60	-1.51	0.10	-15.83	0.00	0.00
ENSSSCG00000006474	NES	303.22	-1.51	0.12	-12.51	0.00	0.00
ENSSSCG00000007682	SH2B2	19.61	-1.50	0.46	-3.28	0.00	0.00
ENSSSCG00000037910	DUSP6	3775.59	-1.50	0.05	-31.05	0.00	0.00
ENSSSCG00000036852	TSEN54	652.55	-1.50	0.08	-18.16	0.00	0.00
ENSSSCG00000033983	RRP7A	858.52	-1.50	0.07	-20.72	0.00	0.00
ENSSSCG00000015961	CDCA7	2032.53	-1.50	0.05	-27.71	0.00	0.00
ENSSSCG00000032996	SLC7A5	6429.87	-1.50	0.06	-26.66	0.00	0.00
ENSSSCG00000033479	NEUROG2	8.82	-1.50	0.63	-2.37	0.02	0.03
ENSSSCG00000004974	LARP6	267.81	-1.50	0.12	-12.12	0.00	0.00
ENSSSCG00000036213	FGF2	57.54	-1.50	0.26	-5.79	0.00	0.00
ENSSSCG00000033695	KCTD15	637.23	-1.49	0.08	-18.30	0.00	0.00
ENSSSCG00000003395	PIK3CD	32.85	-1.49	0.33	-4.54	0.00	0.00
ENSSSCG00000033337	ARHGDIB	1652.72	-1.49	0.07	-22.07	0.00	0.00
ENSSSCG00000026590	HGH1	993.99	-1.49	0.07	-21.13	0.00	0.00
ENSSSCG00000015411	PTPN12	5601.99	-1.49	0.05	-27.36	0.00	0.00
ENSSSCG00000025499		1148.08	-1.48	0.07	-20.78	0.00	0.00
ENSSSCG00000031115	CD70	382.52	-1.47	0.12	-12.47	0.00	0.00
ENSSSCG00000013468	THOP1	1245.37	-1.47	0.07	-22.25	0.00	0.00

ENSSSCG00000037669	PTPN7	476.04	-1.47	0.09	-16.08	0.00	0.00
ENSSSCG00000009432	DGKD	694.15	-1.47	0.08	-17.66	0.00	0.00
ENSSSCG00000022780	UGCG	527.19	-1.47	0.09	-16.20	0.00	0.00
ENSSSCG00000032164	PEA15	5281.49	-1.47	0.04	-33.19	0.00	0.00
ENSSSCG00000024062		449.31	-1.47	0.10	-14.06	0.00	0.00
ENSSSCG00000032728	EFNB1	1916.11	-1.47	0.05	-26.72	0.00	0.00
ENSSSCG00000033893		6.81	-1.46	0.73	-2.00	0.04	0.07
ENSSSCG00000010247	DDX21	6484.22	-1.46	0.05	-32.28	0.00	0.00
ENSSSCG00000017414	KAT2A	1855.44	-1.46	0.06	-25.75	0.00	0.00
ENSSSCG00000016634	CAV1	1470.22	-1.46	0.06	-24.05	0.00	0.00
ENSSSCG00000017882	MYBBP1A	10629.44	-1.46	0.04	-34.76	0.00	0.00
ENSSSCG00000006820	EPS8L3	1055.11	-1.46	0.07	-21.85	0.00	0.00
ENSSSCG00000017066	GEMIN5	4035.92	-1.46	0.04	-33.35	0.00	0.00
ENSSSCG00000017397	MLX	1585.36	-1.46	0.06	-24.49	0.00	0.00
ENSSSCG00000004956	SKOR1	8.66	-1.45	0.63	-2.29	0.02	0.03
ENSSSCG00000024424	FAM91A1	3292.60	-1.45	0.06	-24.06	0.00	0.00
ENSSSCG00000023526	RAPGEF3	177.16	-1.45	0.15	-9.35	0.00	0.00
ENSSSCG00000035715	GCH1	292.35	-1.45	0.12	-11.83	0.00	0.00
ENSSSCG00000001518	ITPR3	3660.43	-1.45	0.05	-30.56	0.00	0.00
ENSSSCG00000023784	SEMA3C	4031.33	-1.45	0.05	-30.05	0.00	0.00
ENSSSCG00000015011		1132.32	-1.44	0.07	-21.64	0.00	0.00
ENSSSCG00000006689	PIAS3	812.82	-1.44	0.08	-18.02	0.00	0.00
ENSSSCG00000016686	PRR15	135.04	-1.44	0.17	-8.74	0.00	0.00
ENSSSCG00000001394		8.00	-1.44	0.67	-2.15	0.03	0.05
ENSSSCG00000018540	RF00569	24.46	-1.44	0.39	-3.68	0.00	0.00

ENSSSCG00000017471	CDC6	853.54	-1.44	0.08	-19.01	0.00	0.00
ENSSSCG00000010464	PPP1R3C	977.71	-1.44	0.07	-20.68	0.00	0.00
ENSSSCG00000023013	RF00412	42.33	-1.44	0.29	-4.94	0.00	0.00
ENSSSCG00000022208	TNFRSF1B	1438.65	-1.44	0.06	-23.41	0.00	0.00
ENSSSCG00000015368	HDAC9	2140.97	-1.43	0.06	-23.36	0.00	0.00
ENSSSCG00000003134	GRWD1	1718.54	-1.43	0.06	-25.35	0.00	0.00
ENSSSCG00000001906	CYP1A1	172.59	-1.43	0.17	-8.57	0.00	0.00
ENSSSCG00000035212	KLF6	1124.63	-1.43	0.07	-21.38	0.00	0.00
ENSSSCG00000007700	HIP1	166.47	-1.43	0.15	-9.46	0.00	0.00
ENSSSCG00000024672	KLF16	958.91	-1.43	0.07	-19.59	0.00	0.00
ENSSSCG00000034814	MRTO4	1413.11	-1.43	0.06	-23.84	0.00	0.00
ENSSSCG00000021145	AP1S1	2285.10	-1.42	0.06	-23.81	0.00	0.00
ENSSSCG00000039770	SLC6A9	684.94	-1.42	0.09	-15.43	0.00	0.00
ENSSSCG00000017155	EIF4A3	4088.36	-1.42	0.05	-29.38	0.00	0.00
ENSSSCG00000017245	CDC42EP4	697.97	-1.41	0.08	-17.10	0.00	0.00
ENSSSCG00000005376	TBC1D2	494.88	-1.41	0.09	-15.59	0.00	0.00
ENSSSCG00000003189	PRMT1	4831.72	-1.41	0.05	-30.69	0.00	0.00
ENSSSCG00000013554	TRIP10	682.00	-1.41	0.08	-17.57	0.00	0.00
ENSSSCG00000036593		1060.60	-1.41	0.07	-20.65	0.00	0.00
ENSSSCG00000025965	SPDL1	1571.91	-1.41	0.06	-23.27	0.00	0.00
ENSSSCG00000017540	HOXB5	84.21	-1.40	0.21	-6.84	0.00	0.00
ENSSSCG00000003551	PAQR7	287.96	-1.40	0.12	-12.02	0.00	0.00
ENSSSCG00000016734	CCM2	1091.92	-1.40	0.07	-19.83	0.00	0.00
ENSSSCG00000028159		265.22	-1.40	0.12	-11.40	0.00	0.00
ENSSSCG00000014950	VSTM5	17.55	-1.40	0.47	-2.95	0.00	0.01

ENSSSCG00000035051	ADORA2B	725.40	-1.40	0.08	-17.92	0.00	0.00
ENSSSCG00000039393	SPRED1	681.61	-1.39	0.08	-16.57	0.00	0.00
ENSSSCG00000030278	MLLT11	132.47	-1.39	0.17	-8.28	0.00	0.00
ENSSSCG00000006521	FAM189B	953.88	-1.39	0.07	-18.57	0.00	0.00
ENSSSCG00000008090	IL1A	78.62	-1.39	0.22	-6.36	0.00	0.00
ENSSSCG00000007772	STX1A	28.64	-1.39	0.35	-3.95	0.00	0.00
ENSSSCG00000024696		3727.32	-1.39	0.05	-25.37	0.00	0.00
ENSSSCG00000016585	IMPDH1	944.55	-1.39	0.07	-18.82	0.00	0.00
ENSSSCG00000035273		250.05	-1.38	0.14	-10.00	0.00	0.00
ENSSSCG00000017333	FMNL1	266.92	-1.38	0.13	-10.97	0.00	0.00
ENSSSCG00000016036	WDR75	1369.64	-1.38	0.06	-23.12	0.00	0.00
ENSSSCG00000026962	A4GALT	1149.22	-1.38	0.07	-20.67	0.00	0.00
ENSSSCG00000005832	MAMDC4	8.34	-1.38	0.66	-2.10	0.04	0.06
ENSSSCG00000026233		595.25	-1.38	0.09	-14.81	0.00	0.00
ENSSSCG00000024084	RF00581	25.73	-1.38	0.38	-3.59	0.00	0.00
ENSSSCG00000017258	FAM20A	248.35	-1.37	0.14	-9.82	0.00	0.00
ENSSSCG00000034738	TRMT61A	853.51	-1.37	0.07	-18.44	0.00	0.00
ENSSSCG00000033720		2016.09	-1.37	0.06	-23.89	0.00	0.00
ENSSSCG00000004342	NDUFAF4	321.38	-1.37	0.11	-12.65	0.00	0.00
ENSSSCG00000017168	SEPT9	3851.88	-1.37	0.05	-29.96	0.00	0.00
ENSSSCG00000012844	SLC25A22	1233.02	-1.37	0.06	-21.25	0.00	0.00
ENSSSCG00000015423	DNAJC2	2992.09	-1.37	0.05	-26.83	0.00	0.00
ENSSSCG00000002875		142.80	-1.36	0.16	-8.62	0.00	0.00
ENSSSCG00000037022	TMEM121	12.97	-1.36	0.52	-2.64	0.01	0.01
ENSSSCG00000031076	MAPK7	2234.69	-1.36	0.06	-24.47	0.00	0.00

ENSSSCG00000010863	AHCTF1	2949.11	-1.36	0.06	-24.37	0.00	0.00
ENSSSCG00000001993	TGM1	9.56	-1.36	0.64	-2.12	0.03	0.05
ENSSSCG00000017429	P3H4	1291.95	-1.36	0.06	-21.42	0.00	0.00
ENSSSCG00000017140	CHMP6	886.94	-1.36	0.07	-18.60	0.00	0.00
ENSSSCG00000015428	PUS7	5005.62	-1.36	0.04	-31.65	0.00	0.00
ENSSSCG00000010478	FFAR4	210.02	-1.36	0.13	-10.24	0.00	0.00
ENSSSCG00000003437	TNFRSF8	662.04	-1.35	0.09	-15.59	0.00	0.00
ENSSSCG00000015090	MPZL3	105.99	-1.35	0.18	-7.38	0.00	0.00
ENSSSCG00000033421		376.11	-1.35	0.10	-13.22	0.00	0.00
ENSSSCG00000028638	UTP4	2713.62	-1.35	0.07	-20.50	0.00	0.00
ENSSSCG00000007039	PRNP	3111.50	-1.35	0.05	-28.07	0.00	0.00
ENSSSCG00000035774	ERRFI1	1169.61	-1.35	0.07	-19.79	0.00	0.00
ENSSSCG00000039094	PLEC	17367.83	-1.34	0.05	-26.30	0.00	0.00
ENSSSCG00000008772	RELL1	2362.16	-1.34	0.05	-26.99	0.00	0.00
ENSSSCG00000007504	RBM38	728.32	-1.34	0.08	-17.33	0.00	0.00
ENSSSCG00000027130	TNFRSF12A	2519.26	-1.34	0.06	-21.99	0.00	0.00
ENSSSCG00000027121	ODC1	9630.39	-1.34	0.04	-33.65	0.00	0.00
ENSSSCG00000017693	AATF	1984.71	-1.34	0.05	-25.11	0.00	0.00
ENSSSCG00000017571	EME1	590.25	-1.33	0.09	-15.66	0.00	0.00
ENSSSCG00000005625	ENG	131.48	-1.33	0.17	-7.87	0.00	0.00
ENSSSCG00000039071		3984.15	-1.33	0.05	-29.35	0.00	0.00
ENSSSCG00000035987	EHD3	2232.77	-1.33	0.06	-23.10	0.00	0.00
ENSSSCG00000032626		317.38	-1.33	0.12	-11.57	0.00	0.00
ENSSSCG00000019228	RF00213	10.96	-1.33	0.58	-2.30	0.02	0.03
ENSSSCG00000017208	SLC25A19	239.11	-1.33	0.12	-10.73	0.00	0.00

ENSSSCG00000033741	NIP7	1545.45	-1.33	0.06	-22.93	0.00	0.00
ENSSSCG00000016698	HOXA11	13.31	-1.33	0.52	-2.57	0.01	0.02
ENSSSCG00000000161	PWP1	1602.34	-1.33	0.06	-21.84	0.00	0.00
ENSSSCG00000000696		2969.82	-1.32	0.06	-23.06	0.00	0.00
ENSSSCG00000008812	ATP10D	245.55	-1.32	0.12	-10.59	0.00	0.00
ENSSSCG00000025067		744.33	-1.32	0.08	-15.82	0.00	0.00
ENSSSCG00000003079		1521.15	-1.32	0.06	-20.69	0.00	0.00
ENSSSCG00000012293	PRICKLE3	465.56	-1.32	0.09	-14.06	0.00	0.00
ENSSSCG00000020754	CD3EAP	731.23	-1.32	0.08	-17.53	0.00	0.00
ENSSSCG00000012649	SMARCA1	8.68	-1.32	0.64	-2.07	0.04	0.06
ENSSSCG00000030510	FBL	1840.99	-1.32	0.06	-20.77	0.00	0.00
ENSSSCG00000016824	RAI14	2149.81	-1.32	0.05	-25.11	0.00	0.00
ENSSSCG00000023177	RRP9	702.34	-1.31	0.08	-16.65	0.00	0.00
ENSSSCG00000001710	RUNX2	182.12	-1.31	0.15	-9.06	0.00	0.00
ENSSSCG00000037099	ANKLE1	195.27	-1.31	0.14	-9.53	0.00	0.00
ENSSSCG00000011604	CHCHD4	695.05	-1.31	0.08	-16.92	0.00	0.00
ENSSSCG00000037749	RF00580	23.40	-1.31	0.41	-3.22	0.00	0.00
ENSSSCG00000000765	IL17RA	1413.19	-1.31	0.06	-20.60	0.00	0.00
ENSSSCG00000002555	JAG2	1421.15	-1.31	0.06	-21.05	0.00	0.00
ENSSSCG00000015955	ITGA6	5860.19	-1.30	0.04	-30.17	0.00	0.00
ENSSSCG00000010576	NOLC1	5757.56	-1.30	0.04	-31.46	0.00	0.00
ENSSSCG00000023599	TIMM8B	1001.93	-1.30	0.07	-18.74	0.00	0.00
ENSSSCG00000012952	TMEM151A	24.42	-1.30	0.39	-3.33	0.00	0.00
ENSSSCG00000000906		82.20	-1.30	0.21	-6.22	0.00	0.00
ENSSSCG00000003718	TAF4B	624.11	-1.30	0.08	-15.76	0.00	0.00

ENSSSCG00000033505		77.56	-1.30	0.21	-6.11	0.00	0.00
ENSSSCG00000003705	CABLES1	136.54	-1.30	0.17	-7.63	0.00	0.00
ENSSSCG00000010915	NAV1	2682.65	-1.29	0.05	-25.45	0.00	0.00
ENSSSCG00000028047	DTNA	195.77	-1.29	0.14	-8.93	0.00	0.00
ENSSSCG00000005935	AGO2	1716.73	-1.29	0.06	-22.69	0.00	0.00
ENSSSCG00000028019	LRRC59	7513.57	-1.29	0.04	-31.84	0.00	0.00
ENSSSCG00000016548		3775.12	-1.29	0.05	-27.80	0.00	0.00
ENSSSCG00000017396	PSMC3IP	550.54	-1.29	0.09	-14.55	0.00	0.00
ENSSSCG00000035403	RFX2	284.80	-1.29	0.12	-10.82	0.00	0.00
ENSSSCG00000007718	CLDN4	1577.17	-1.29	0.06	-19.94	0.00	0.00
ENSSSCG00000015277	SOX13	103.16	-1.29	0.19	-6.79	0.00	0.00
ENSSSCG00000016869		21.44	-1.28	0.42	-3.08	0.00	0.00
ENSSSCG00000015001	SLC35F2	1758.57	-1.28	0.05	-23.53	0.00	0.00
ENSSSCG00000030831	DENND3	463.84	-1.28	0.10	-13.44	0.00	0.00
ENSSSCG00000015222	PUS3	443.32	-1.28	0.10	-12.70	0.00	0.00
ENSSSCG00000001504	WDR46	636.17	-1.28	0.08	-15.60	0.00	0.00
ENSSSCG00000001834	MFGE8	1139.80	-1.28	0.06	-19.78	0.00	0.00
ENSSSCG00000000998	RPP40	890.92	-1.28	0.07	-18.03	0.00	0.00
ENSSSCG00000002235	LPCAT4	751.59	-1.28	0.08	-16.30	0.00	0.00
ENSSSCG00000038366	RAP2B	712.87	-1.28	0.08	-16.67	0.00	0.00
ENSSSCG00000017125	FOXK2	2546.44	-1.28	0.05	-25.50	0.00	0.00
ENSSSCG00000005510	PHF19	1101.95	-1.28	0.07	-19.14	0.00	0.00
ENSSSCG00000004224	HDDC2	328.61	-1.28	0.11	-11.90	0.00	0.00
ENSSSCG00000016745	DDX56	887.68	-1.27	0.07	-17.46	0.00	0.00
ENSSSCG00000015145	ZNF202	248.00	-1.27	0.13	-10.07	0.00	0.00

ENSSSCG00000040183	CDK6	1590.02	-1.27	0.07	-19.53	0.00	0.00
ENSSSCG00000023984	RSL1D1	4447.78	-1.27	0.05	-26.69	0.00	0.00
ENSSSCG00000014970	MTMR2	3562.89	-1.27	0.05	-27.96	0.00	0.00
ENSSSCG00000027365	WNT7B	1439.86	-1.27	0.06	-21.26	0.00	0.00
ENSSSCG00000005130	ELAVL2	405.02	-1.27	0.10	-12.85	0.00	0.00
ENSSSCG00000032903		227.92	-1.27	0.13	-9.85	0.00	0.00
ENSSSCG00000009482	SPRY2	345.53	-1.27	0.11	-11.37	0.00	0.00
ENSSSCG00000016626	CFTR	476.90	-1.27	0.09	-13.91	0.00	0.00
ENSSSCG00000039408	ADCY7	1018.65	-1.27	0.07	-16.89	0.00	0.00
ENSSSCG00000028978	CAD	4197.93	-1.27	0.04	-28.65	0.00	0.00
ENSSSCG00000010514	RRP12	2563.67	-1.27	0.05	-24.90	0.00	0.00
ENSSSCG00000036307	OAF	187.21	-1.26	0.14	-8.98	0.00	0.00
ENSSSCG00000029005	KPNA2	7788.94	-1.26	0.04	-29.81	0.00	0.00
ENSSSCG00000032870		9.01	-1.26	0.62	-2.05	0.04	0.06
ENSSSCG00000019687	RF00403	12.29	-1.26	0.53	-2.37	0.02	0.03
ENSSSCG00000015227	SRPRA	5603.27	-1.26	0.04	-28.84	0.00	0.00
ENSSSCG00000008227	ST3GAL5	119.34	-1.26	0.17	-7.22	0.00	0.00
ENSSSCG00000029627		1532.85	-1.26	0.06	-21.36	0.00	0.00
ENSSSCG00000032655	ALYREF	2192.53	-1.26	0.07	-17.73	0.00	0.00
ENSSSCG00000002746	DHODH	230.11	-1.26	0.13	-9.62	0.00	0.00
ENSSSCG00000029944	FASN	11124.65	-1.25	0.04	-31.20	0.00	0.00
ENSSSCG00000011542	THUMPD3	991.36	-1.25	0.07	-17.67	0.00	0.00
ENSSSCG00000036431	VPS13C	2331.14	-1.25	0.06	-19.79	0.00	0.00
ENSSSCG00000036787	APOLD1	11.15	-1.25	0.55	-2.28	0.02	0.04
ENSSSCG00000035828	ARHGEF4	181.01	-1.25	0.14	-8.81	0.00	0.00

ENSSSCG00000035923	JMJD6	702.92	-1.25	0.08	-15.64	0.00	0.00
ENSSSCG00000000377		5620.49	-1.25	0.04	-30.02	0.00	0.00
ENSSSCG00000009446	PCDH17	46.78	-1.25	0.27	-4.62	0.00	0.00
ENSSSCG00000040425	YRDC	373.07	-1.25	0.10	-12.08	0.00	0.00
ENSSSCG00000004615	WDR72	211.30	-1.24	0.13	-9.32	0.00	0.00
ENSSSCG00000033202		25.69	-1.24	0.37	-3.39	0.00	0.00
ENSSSCG00000035344	UBE2S	1352.19	-1.24	0.06	-20.09	0.00	0.00
ENSSSCG00000034723	PYCR1	2272.77	-1.24	0.05	-23.42	0.00	0.00
ENSSSCG00000017873	CAMKK1	344.46	-1.24	0.11	-11.61	0.00	0.00
ENSSSCG00000025460	SPSB2	81.82	-1.24	0.21	-5.83	0.00	0.00
ENSSSCG00000005533	PTGS1	1460.57	-1.24	0.08	-16.54	0.00	0.00
ENSSSCG00000031600	SYNGR2	1878.94	-1.24	0.07	-17.98	0.00	0.00
ENSSSCG00000012823	DKC1	3075.21	-1.24	0.05	-26.30	0.00	0.00
ENSSSCG00000005965	MYC	5346.72	-1.24	0.10	-11.85	0.00	0.00
ENSSSCG00000033027	CIAO2B	336.17	-1.24	0.11	-10.94	0.00	0.00
ENSSSCG00000016717	MPP6	413.44	-1.24	0.10	-12.44	0.00	0.00
ENSSSCG00000006581		3089.89	-1.24	0.06	-21.30	0.00	0.00
ENSSSCG00000010010	PES1	3373.97	-1.24	0.05	-23.54	0.00	0.00
ENSSSCG00000001990	NOP9	525.54	-1.23	0.09	-13.03	0.00	0.00
ENSSSCG00000015355	DGKB	47.67	-1.23	0.27	-4.52	0.00	0.00
ENSSSCG00000008143	UXS1	1396.67	-1.23	0.07	-18.50	0.00	0.00
ENSSSCG00000011178	CPNE4	161.36	-1.23	0.15	-8.21	0.00	0.00
ENSSSCG00000017207	NUP85	1322.74	-1.23	0.06	-20.52	0.00	0.00
ENSSSCG00000015215		650.17	-1.23	0.08	-15.47	0.00	0.00
ENSSSCG00000015340	ASNS	5413.61	-1.23	0.05	-22.86	0.00	0.00

ENSSSCG00000010198	BMS1	1918.78	-1.23	0.06	-22.01	0.00	0.00
ENSSSCG00000004703		191.19	-1.23	0.14	-8.87	0.00	0.00
ENSSSCG00000002787	E2F4	2163.39	-1.23	0.06	-20.78	0.00	0.00
ENSSSCG00000023775	TFB2M	286.51	-1.23	0.12	-10.11	0.00	0.00
ENSSSCG00000000869	UTP20	1883.12	-1.23	0.05	-22.66	0.00	0.00
ENSSSCG00000023323		1555.32	-1.23	0.06	-20.30	0.00	0.00
ENSSSCG00000025187	TMEM126A	626.92	-1.22	0.09	-13.68	0.00	0.00
ENSSSCG00000021706	KIF21B	265.52	-1.22	0.12	-10.42	0.00	0.00
ENSSSCG00000021997	ALS2CL	540.74	-1.22	0.09	-13.94	0.00	0.00
ENSSSCG00000010575	PPRC1	2842.95	-1.22	0.06	-21.03	0.00	0.00
ENSSSCG00000006496	LMNA	11538.43	-1.22	0.05	-23.61	0.00	0.00
ENSSSCG00000016275	NCL	27593.15	-1.22	0.04	-29.46	0.00	0.00
ENSSSCG00000009759	SCARB1	1669.02	-1.22	0.07	-18.74	0.00	0.00
ENSSSCG00000008429	EPCAM	3319.83	-1.22	0.05	-25.75	0.00	0.00
ENSSSCG00000010145	HEATR1	2590.77	-1.22	0.05	-23.26	0.00	0.00
ENSSSCG00000006892	DNTTIP2	4868.46	-1.22	0.05	-24.18	0.00	0.00
ENSSSCG00000005247		3365.42	-1.22	0.05	-25.75	0.00	0.00
ENSSSCG00000031492	PPP1R18	533.81	-1.22	0.09	-14.23	0.00	0.00
ENSSSCG00000029391	PLSCR3	1039.83	-1.22	0.07	-18.61	0.00	0.00
ENSSSCG00000009692		486.82	-1.22	0.09	-13.26	0.00	0.00
ENSSSCG00000036531		379.69	-1.22	0.10	-11.87	0.00	0.00
ENSSSCG00000017342	KIF18B	975.95	-1.21	0.07	-16.35	0.00	0.00
ENSSSCG00000006727	WDR3	2309.05	-1.21	0.05	-22.74	0.00	0.00
ENSSSCG00000031847	RBM28	811.64	-1.21	0.07	-16.56	0.00	0.00
ENSSSCG00000002469	OTUB2	246.38	-1.21	0.13	-9.23	0.00	0.00

ENSSSCG00000039867	UCK2	2940.29	-1.21	0.05	-25.10	0.00	0.00
ENSSSCG00000028210	NT5C	947.99	-1.21	0.07	-17.63	0.00	0.00
ENSSSCG00000005012	ARF6	6571.17	-1.21	0.04	-27.83	0.00	0.00
ENSSSCG00000011610	NUP210	834.18	-1.21	0.07	-16.49	0.00	0.00
ENSSSCG00000016573	IRF5	97.37	-1.21	0.19	-6.39	0.00	0.00
ENSSSCG00000037061	RRP1	698.18	-1.21	0.08	-15.59	0.00	0.00
ENSSSCG00000011125	GATA3	38.35	-1.21	0.30	-4.00	0.00	0.00
ENSSSCG00000008635	NOL10	1086.02	-1.20	0.07	-17.97	0.00	0.00
ENSSSCG00000028602	GTPBP4	1542.88	-1.20	0.06	-20.91	0.00	0.00
ENSSSCG00000006003	MAL2	2039.99	-1.20	0.05	-22.25	0.00	0.00
ENSSSCG00000019644	RF00281	114.38	-1.20	0.18	-6.73	0.00	0.00
ENSSSCG00000010568	NPM3	2876.74	-1.20	0.05	-22.72	0.00	0.00
ENSSSCG00000000635		514.69	-1.20	0.09	-13.73	0.00	0.00
ENSSSCG00000017072	GALNT10	3267.35	-1.20	0.05	-25.98	0.00	0.00
ENSSSCG00000009531	TEX30	498.50	-1.20	0.09	-13.35	0.00	0.00
ENSSSCG00000021173	BSPRY	389.43	-1.20	0.10	-11.67	0.00	0.00
ENSSSCG00000039592	ZMYND19	469.48	-1.19	0.09	-13.03	0.00	0.00
ENSSSCG00000010163	KCNK1	1349.98	-1.19	0.06	-19.64	0.00	0.00
ENSSSCG00000013662	PPAN	867.06	-1.19	0.07	-16.14	0.00	0.00
ENSSSCG00000012315	SYNJ2	973.80	-1.19	0.07	-16.49	0.00	0.00
ENSSSCG00000008693	NOP14	470.67	-1.19	0.10	-12.13	0.00	0.00
ENSSSCG00000026466	SLC23A2	2888.63	-1.19	0.05	-24.80	0.00	0.00
ENSSSCG00000017957	EIF4A1	23688.81	-1.18	0.03	-34.95	0.00	0.00
ENSSSCG00000004441		895.37	-1.18	0.08	-15.57	0.00	0.00
ENSSSCG00000037982	RF00278	63.35	-1.18	0.24	-4.86	0.00	0.00

ENSSSCG00000004744	TYRO3	242.48	-1.18	0.13	-8.94	0.00	0.00
ENSSSCG00000006643	ANXA9	39.94	-1.18	0.29	-4.02	0.00	0.00
ENSSSCG00000040707		22.63	-1.18	0.39	-3.03	0.00	0.00
ENSSSCG00000036746	RASL10B	1482.45	-1.18	0.06	-19.93	0.00	0.00
ENSSSCG00000010772	ADAM8	22.66	-1.18	0.39	-3.05	0.00	0.00
ENSSSCG00000011599	GRIP2	508.59	-1.18	0.09	-13.39	0.00	0.00
ENSSSCG00000027455	SLC39A6	2778.30	-1.18	0.05	-22.00	0.00	0.00
ENSSSCG00000017251	SOX9	173.50	-1.18	0.15	-7.93	0.00	0.00
ENSSSCG00000032144	ZNF140	86.48	-1.18	0.20	-5.86	0.00	0.00
ENSSSCG00000007754	ITGAM	248.75	-1.18	0.12	-9.65	0.00	0.00
ENSSSCG00000022504	CDON	47.24	-1.18	0.27	-4.33	0.00	0.00
ENSSSCG00000039360	LRFN4	1134.86	-1.17	0.08	-15.58	0.00	0.00
ENSSSCG00000028202	RANGAP1	1921.72	-1.17	0.05	-22.11	0.00	0.00
ENSSSCG00000004048	TCP1	6540.83	-1.17	0.05	-25.92	0.00	0.00
ENSSSCG00000024750	GFOD2	226.67	-1.17	0.13	-9.23	0.00	0.00
ENSSSCG00000035666	C20orf27	2185.19	-1.17	0.06	-19.61	0.00	0.00
ENSSSCG00000023139	METTL23	917.75	-1.17	0.07	-15.92	0.00	0.00
ENSSSCG00000015380	CDCA7L	2655.70	-1.17	0.05	-22.06	0.00	0.00
ENSSSCG00000037606	PSMB3	2033.35	-1.17	0.05	-22.14	0.00	0.00
ENSSSCG00000007487	PFDN4	960.88	-1.17	0.07	-16.39	0.00	0.00
ENSSSCG00000023870	RF00581	54.46	-1.17	0.26	-4.58	0.00	0.00
ENSSSCG00000040904	CLDN1	10136.97	-1.17	0.04	-25.97	0.00	0.00
ENSSSCG00000032203	EPPK1	758.02	-1.17	0.08	-14.77	0.00	0.00
ENSSSCG00000023973	NOC2L	1721.78	-1.17	0.06	-20.15	0.00	0.00
ENSSSCG00000022200	SYT17	51.48	-1.17	0.26	-4.45	0.00	0.00

ENSSSCG00000024235	NPLOC4	2594.47	-1.17	0.05	-21.89	0.00	0.00
ENSSSCG00000008878	PPID	1597.08	-1.16	0.06	-19.73	0.00	0.00
ENSSSCG00000007027	SLC20A2	653.97	-1.16	0.08	-13.85	0.00	0.00
ENSSSCG00000011449	GNL3	2005.21	-1.16	0.06	-20.57	0.00	0.00
ENSSSCG00000017640		306.95	-1.16	0.11	-10.26	0.00	0.00
ENSSSCG00000036814	CLEC11A	959.70	-1.16	0.08	-15.18	0.00	0.00
ENSSSCG00000039443		214.29	-1.16	0.13	-8.77	0.00	0.00
ENSSSCG00000011101	ITGB1	11081.05	-1.16	0.04	-26.39	0.00	0.00
ENSSSCG00000014965	ENDOD1	2005.88	-1.16	0.06	-20.46	0.00	0.00
ENSSSCG00000033015	B3GNT7	75.86	-1.16	0.24	-4.91	0.00	0.00
ENSSSCG00000022405	P2RX1	92.30	-1.16	0.20	-5.72	0.00	0.00
ENSSSCG00000019069	RF00592	95.66	-1.16	0.19	-5.97	0.00	0.00
ENSSSCG00000003807	DNAJC6	666.83	-1.16	0.08	-13.77	0.00	0.00
ENSSSCG00000005925	EXOSC4	330.37	-1.16	0.11	-10.47	0.00	0.00
ENSSSCG00000024418	RHOU	292.05	-1.16	0.12	-9.79	0.00	0.00
ENSSSCG00000015541	IER5	712.27	-1.16	0.08	-13.67	0.00	0.00
ENSSSCG00000025921		351.53	-1.16	0.10	-11.08	0.00	0.00
ENSSSCG00000035020	STK32B	100.78	-1.15	0.19	-6.16	0.00	0.00
ENSSSCG00000033412	B4GALNT3	756.23	-1.15	0.08	-14.58	0.00	0.00
ENSSSCG00000015611	UTP25	2057.91	-1.15	0.05	-22.07	0.00	0.00
ENSSSCG00000006481	GPATCH4	389.44	-1.15	0.11	-10.93	0.00	0.00
ENSSSCG00000005494	TNC	18.12	-1.15	0.46	-2.50	0.01	0.02
ENSSSCG00000029651	SLN	24.42	-1.15	0.38	-3.06	0.00	0.00
ENSSSCG00000038308	RANBP1	5501.43	-1.15	0.05	-23.41	0.00	0.00
ENSSSCG00000026940	CASP10	338.61	-1.15	0.11	-10.94	0.00	0.00

ENSSSCG00000039862	TRIB3	781.66	-1.15	0.09	-13.50	0.00	0.00
ENSSSCG00000035863	PLIN2	3135.06	-1.15	0.05	-21.11	0.00	0.00
ENSSSCG00000038970	BCL2L15	119.39	-1.15	0.17	-6.65	0.00	0.00
ENSSSCG00000026812	EMG1	1771.62	-1.15	0.06	-19.62	0.00	0.00
ENSSSCG00000013747	RAD23A	1997.47	-1.15	0.06	-19.97	0.00	0.00
ENSSSCG00000030386	NUP205	2852.67	-1.15	0.05	-23.94	0.00	0.00
ENSSSCG00000022478	STK10	1942.17	-1.15	0.05	-21.35	0.00	0.00
ENSSSCG00000040352	DUS1L	1520.31	-1.15	0.06	-19.91	0.00	0.00
ENSSSCG00000038196		52.39	-1.14	0.25	-4.51	0.00	0.00
ENSSSCG00000025766	SLC10A3	1595.44	-1.14	0.06	-18.90	0.00	0.00
ENSSSCG00000027676	NOP56	5273.53	-1.14	0.04	-27.03	0.00	0.00
ENSSSCG00000036893	PTHLH	30.09	-1.14	0.34	-3.40	0.00	0.00
ENSSSCG00000001041	PAK1IP1	908.00	-1.14	0.07	-15.89	0.00	0.00
ENSSSCG00000039962	HSPB1	12710.80	-1.14	0.05	-25.24	0.00	0.00
ENSSSCG00000016116	WDR12	1518.79	-1.14	0.06	-19.54	0.00	0.00
ENSSSCG00000036956		64.64	-1.14	0.24	-4.70	0.00	0.00
ENSSSCG00000011034	ST8SIA6	30.18	-1.14	0.34	-3.33	0.00	0.00
ENSSSCG00000019612	RF00069	135.19	-1.14	0.18	-6.42	0.00	0.00
ENSSSCG00000038694	CCND2	2021.37	-1.14	0.07	-17.39	0.00	0.00
ENSSSCG00000002719	EXOSC6	121.50	-1.14	0.17	-6.65	0.00	0.00
ENSSSCG00000005920	BOP1	1809.35	-1.14	0.06	-20.34	0.00	0.00
ENSSSCG00000016441		2141.21	-1.14	0.06	-20.27	0.00	0.00
ENSSSCG00000007465	B4GALT5	3619.75	-1.13	0.05	-24.60	0.00	0.00
ENSSSCG00000009741	PUS1	794.57	-1.13	0.07	-15.16	0.00	0.00
ENSSSCG00000006893	BCAR3	1188.37	-1.13	0.07	-16.28	0.00	0.00

ENSSSCG00000021698	RRP1B	1510.26	-1.13	0.06	-19.20	0.00	0.00
ENSSSCG00000035928	CLDN3	19.46	-1.13	0.42	-2.72	0.01	0.01
ENSSSCG00000013546	DENND1C	43.84	-1.13	0.29	-3.95	0.00	0.00
ENSSSCG00000008550	SLC5A6	3570.82	-1.13	0.05	-24.63	0.00	0.00
ENSSSCG00000005596	ARPC5L	1527.65	-1.13	0.06	-19.18	0.00	0.00
ENSSSCG00000015036	DIXDC1	101.30	-1.13	0.19	-6.02	0.00	0.00
ENSSSCG00000017364	C17orf53	317.56	-1.13	0.11	-10.24	0.00	0.00
ENSSSCG00000017165		1086.37	-1.13	0.07	-16.13	0.00	0.00
ENSSSCG00000006340	UAP1	1086.73	-1.13	0.07	-17.30	0.00	0.00
ENSSSCG00000037900	CCND1	2353.08	-1.13	0.05	-21.48	0.00	0.00
ENSSSCG00000040411	CCDC69	14.13	-1.12	0.50	-2.27	0.02	0.04
ENSSSCG00000007704	NSUN5	458.48	-1.12	0.10	-11.55	0.00	0.00
ENSSSCG00000009083	SPRY1	500.60	-1.12	0.09	-12.39	0.00	0.00
ENSSSCG00000007840	POLR3E	1180.93	-1.12	0.07	-17.24	0.00	0.00
ENSSSCG00000027745	ABCG1	245.17	-1.12	0.13	-8.78	0.00	0.00
ENSSSCG00000004394	RPF2	1041.68	-1.12	0.07	-17.21	0.00	0.00
ENSSSCG00000036675		267.83	-1.12	0.12	-9.44	0.00	0.00
ENSSSCG00000036257		661.68	-1.12	0.08	-14.22	0.00	0.00
ENSSSCG00000033149	DDX47	950.11	-1.12	0.07	-16.66	0.00	0.00
ENSSSCG00000023658	NR2C2AP	150.99	-1.12	0.16	-7.11	0.00	0.00
ENSSSCG00000013304	NAT10	1942.88	-1.12	0.05	-20.79	0.00	0.00
ENSSSCG00000015712	DDX18	4093.29	-1.12	0.04	-25.22	0.00	0.00
ENSSSCG00000039640	CDC34	1961.26	-1.12	0.06	-20.09	0.00	0.00
ENSSSCG00000016461	ZYX	3583.90	-1.12	0.06	-18.43	0.00	0.00
ENSSSCG00000003458	EFHD2	2187.45	-1.12	0.06	-19.96	0.00	0.00

ENSSSCG00000026752	BYSL	1504.00	-1.12	0.06	-19.18	0.00	0.00
ENSSSCG00000010923	UBE2T	862.00	-1.11	0.07	-15.36	0.00	0.00
ENSSSCG00000017202	H3F3B	7472.15	-1.11	0.04	-26.50	0.00	0.00
ENSSSCG00000018229	RF00284	90.55	-1.11	0.20	-5.60	0.00	0.00
ENSSSCG00000025652	CDH1	5734.18	-1.11	0.05	-20.94	0.00	0.00
ENSSSCG00000028996	ALDH1A1	39037.67	-1.11	0.04	-31.73	0.00	0.00
ENSSSCG00000040158	RF00612	37.20	-1.11	0.31	-3.61	0.00	0.00
ENSSSCG00000025796	NLE1	669.65	-1.11	0.09	-12.36	0.00	0.00
ENSSSCG00000017584	PPP1R9B	974.63	-1.11	0.07	-16.13	0.00	0.00
ENSSSCG00000017385	PSME3	4550.83	-1.11	0.05	-22.97	0.00	0.00
ENSSSCG00000013223	NUP160	2044.35	-1.11	0.05	-21.13	0.00	0.00
ENSSSCG00000013723		66.29	-1.11	0.23	-4.84	0.00	0.00
ENSSSCG00000033171	MAPK11	145.12	-1.11	0.16	-7.02	0.00	0.00
ENSSSCG00000017514	KPNB1	18057.02	-1.11	0.04	-30.43	0.00	0.00
ENSSSCG00000001700	SLC29A1	2823.58	-1.11	0.06	-19.44	0.00	0.00
ENSSSCG00000019783	RF00150	20.78	-1.11	0.41	-2.71	0.01	0.01
ENSSSCG00000009868	RBM19	2081.32	-1.10	0.06	-20.01	0.00	0.00
ENSSSCG00000027992	MST1R	1133.08	-1.10	0.07	-16.63	0.00	0.00
ENSSSCG00000034569		529.30	-1.10	0.09	-12.05	0.00	0.00
ENSSSCG00000016758		247.60	-1.10	0.12	-9.02	0.00	0.00
ENSSSCG00000002440	CCDC88C	461.41	-1.10	0.09	-12.01	0.00	0.00
ENSSSCG00000003552	AUNIP	98.05	-1.10	0.20	-5.37	0.00	0.00
ENSSSCG00000038713	CRIP2	2950.76	-1.10	0.06	-17.60	0.00	0.00
ENSSSCG00000016794	MYO10	3997.18	-1.10	0.04	-24.51	0.00	0.00
ENSSSCG00000038663		14.90	-1.10	0.49	-2.25	0.02	0.04

ENSSSCG00000014969		2043.84	-1.10	0.06	-18.57	0.00	0.00
ENSSSCG00000005355	EXOSC3	151.81	-1.10	0.15	-7.12	0.00	0.00
ENSSSCG00000035493	SACS	5368.37	-1.10	0.04	-26.20	0.00	0.00
ENSSSCG00000010599	PDCD11	3381.90	-1.09	0.05	-22.71	0.00	0.00
ENSSSCG00000004847	MPHOSPH10	1203.87	-1.09	0.07	-15.98	0.00	0.00
ENSSSCG00000036947	MAK16	3552.91	-1.09	0.05	-23.03	0.00	0.00
ENSSSCG00000040575	ISG15	34.61	-1.09	0.32	-3.40	0.00	0.00
ENSSSCG00000016970		1227.27	-1.09	0.06	-16.81	0.00	0.00
ENSSSCG00000016459	EPHA1	406.86	-1.09	0.10	-11.23	0.00	0.00
ENSSSCG00000024299	SCN3B	25.75	-1.09	0.36	-3.00	0.00	0.00
ENSSSCG00000001679	XPO5	9074.58	-1.09	0.04	-29.07	0.00	0.00
ENSSSCG00000000433	B4GALNT1	156.95	-1.09	0.16	-6.68	0.00	0.00
ENSSSCG00000028465	ELAC2	2733.56	-1.08	0.05	-21.20	0.00	0.00
ENSSSCG00000011033	VIM	104212.38	-1.08	0.05	-23.82	0.00	0.00
ENSSSCG00000001988	ADCY4	59.74	-1.08	0.24	-4.51	0.00	0.00
ENSSSCG00000006366	PFDN2	536.02	-1.08	0.09	-12.32	0.00	0.00
ENSSSCG00000005227	PUM3	1477.18	-1.08	0.06	-18.36	0.00	0.00
ENSSSCG00000000258	RARG	103.32	-1.08	0.19	-5.59	0.00	0.00
ENSSSCG00000000058	SNU13	2460.34	-1.08	0.05	-21.84	0.00	0.00
ENSSSCG00000028807	RF00190	23.62	-1.08	0.39	-2.78	0.01	0.01
ENSSSCG00000023086	RF00405	23.62	-1.08	0.37	-2.88	0.00	0.01
ENSSSCG00000004503	LOXHD1	58.30	-1.08	0.25	-4.34	0.00	0.00
ENSSSCG00000025447	MID1IP1	508.89	-1.08	0.09	-11.77	0.00	0.00
ENSSSCG00000018352	RF00186	25.63	-1.08	0.37	-2.94	0.00	0.01
ENSSSCG00000010865	EXO1	580.77	-1.08	0.09	-12.27	0.00	0.00

ENSSSCG00000039761	MYCL	35.05	-1.08	0.32	-3.31	0.00	0.00
ENSSSCG00000016827	BRIX1	1155.47	-1.08	0.07	-16.41	0.00	0.00
ENSSSCG00000017788	TP53I13	454.96	-1.07	0.09	-11.71	0.00	0.00
ENSSSCG00000005267	ANXA1	27096.46	-1.07	0.04	-24.12	0.00	0.00
ENSSSCG00000040719	KIAA0040	749.47	-1.07	0.08	-14.10	0.00	0.00
ENSSSCG00000015845	GTF2E2	1266.53	-1.07	0.06	-17.55	0.00	0.00
ENSSSCG00000017204	ITGB4	5173.52	-1.07	0.04	-24.97	0.00	0.00
ENSSSCG00000002559	CDCA4	243.54	-1.07	0.13	-8.56	0.00	0.00
ENSSSCG00000022915	DDX27	1808.53	-1.07	0.06	-19.22	0.00	0.00
ENSSSCG00000034716	CCDC137	548.67	-1.07	0.09	-12.21	0.00	0.00
ENSSSCG00000029249	NAV3	1193.73	-1.07	0.07	-15.93	0.00	0.00
ENSSSCG00000015612	IRF6	435.57	-1.07	0.09	-11.47	0.00	0.00
ENSSSCG00000006500	UBQLN4	1857.69	-1.07	0.06	-19.10	0.00	0.00
ENSSSCG00000009420	NUFIP1	700.16	-1.07	0.09	-12.13	0.00	0.00
ENSSSCG00000036702	ACTN1	10597.43	-1.06	0.04	-24.44	0.00	0.00
ENSSSCG00000006985		30.15	-1.06	0.34	-3.16	0.00	0.00
ENSSSCG00000033586	DBNDD2	745.20	-1.06	0.07	-14.40	0.00	0.00
ENSSSCG00000028182	CDK17	137.94	-1.06	0.16	-6.49	0.00	0.00
ENSSSCG00000033518	LLGL2	1640.87	-1.06	0.06	-17.06	0.00	0.00
ENSSSCG00000021397	PODXL2	43.56	-1.06	0.29	-3.72	0.00	0.00
ENSSSCG00000005344	MELK	1226.39	-1.06	0.07	-15.87	0.00	0.00
ENSSSCG00000014255	SLC12A2	842.19	-1.06	0.07	-14.23	0.00	0.00
ENSSSCG00000016732	TBRG4	1994.71	-1.06	0.06	-19.17	0.00	0.00
ENSSSCG00000003089	TOMM40	2950.87	-1.06	0.05	-22.86	0.00	0.00
ENSSSCG00000037325	UBIAD1	480.19	-1.06	0.09	-11.57	0.00	0.00

ENSSSCG00000032062	WDR55	437.99	-1.05	0.09	-11.32	0.00	0.00
ENSSSCG00000013029	TRMT112	928.64	-1.05	0.07	-15.26	0.00	0.00
ENSSSCG00000033295	EIF2S1	2361.72	-1.05	0.05	-20.52	0.00	0.00
ENSSSCG00000003861	ORC1	1910.90	-1.05	0.06	-18.98	0.00	0.00
ENSSSCG00000000466	XPOT	5327.24	-1.05	0.04	-24.42	0.00	0.00
ENSSSCG00000032106	SURF6	476.38	-1.05	0.10	-10.94	0.00	0.00
ENSSSCG00000023400	ZNF598	860.77	-1.05	0.07	-14.71	0.00	0.00
ENSSSCG00000027135	PPM1G	3464.21	-1.05	0.05	-20.30	0.00	0.00
ENSSSCG00000030045	PRMT5	3779.55	-1.05	0.05	-22.01	0.00	0.00
ENSSSCG00000000682	GNB3	21.31	-1.05	0.41	-2.58	0.01	0.02
ENSSSCG00000011918	USF3	864.58	-1.05	0.07	-14.81	0.00	0.00
ENSSSCG00000024596	NOCT	208.11	-1.05	0.14	-7.71	0.00	0.00
ENSSSCG00000013745	FARSA	2092.75	-1.04	0.05	-19.24	0.00	0.00
ENSSSCG00000021834	ERCC6L	696.48	-1.04	0.08	-12.35	0.00	0.00
ENSSSCG00000008974		1610.02	-1.04	0.06	-17.46	0.00	0.00
ENSSSCG00000033298	SAMD1	633.46	-1.04	0.09	-11.18	0.00	0.00
ENSSSCG00000009775	CDK2AP1	1414.10	-1.04	0.06	-16.50	0.00	0.00
ENSSSCG00000007366	MYBL2	8625.16	-1.04	0.06	-18.46	0.00	0.00
ENSSSCG00000033037		918.26	-1.04	0.07	-14.96	0.00	0.00
ENSSSCG00000001835	ABHD2	2884.96	-1.04	0.05	-21.84	0.00	0.00
ENSSSCG00000009137	GAR1	513.49	-1.04	0.09	-12.05	0.00	0.00
ENSSSCG00000000194		6275.23	-1.04	0.06	-17.34	0.00	0.00
ENSSSCG00000004179	VNN2	2278.07	-1.04	0.05	-19.91	0.00	0.00
ENSSSCG00000015483	EEF1AKNMT	1002.94	-1.04	0.07	-15.83	0.00	0.00
ENSSSCG00000017206	GRB2	2204.94	-1.04	0.05	-20.69	0.00	0.00

ENSSSCG00000007472	BCAS4	255.94	-1.04	0.12	-8.62	0.00	0.00
ENSSSCG00000013352	E2F8	763.85	-1.04	0.08	-13.55	0.00	0.00
ENSSSCG00000022246		397.89	-1.04	0.10	-10.36	0.00	0.00
ENSSSCG00000038987	CCDC59	402.65	-1.04	0.10	-10.79	0.00	0.00
ENSSSCG00000023078	WDR4	532.17	-1.04	0.09	-12.17	0.00	0.00
ENSSSCG00000015600	DTL	1514.20	-1.04	0.06	-17.99	0.00	0.00
ENSSSCG00000026547	SLC45A3	345.08	-1.04	0.11	-9.78	0.00	0.00
ENSSSCG00000029456	SLC7A1	2087.09	-1.04	0.05	-19.44	0.00	0.00
ENSSSCG00000036933	NR1D1	71.09	-1.04	0.22	-4.78	0.00	0.00
ENSSSCG00000016185	ARPC2	7472.49	-1.04	0.04	-24.13	0.00	0.00
ENSSSCG00000021620	STIP1	5192.91	-1.03	0.04	-25.72	0.00	0.00
ENSSSCG00000007737	TPST1	255.64	-1.03	0.12	-8.61	0.00	0.00
ENSSSCG00000017489	PSMD3	3969.70	-1.03	0.05	-21.33	0.00	0.00
ENSSSCG00000038992	TUBB6	1528.05	-1.03	0.06	-17.96	0.00	0.00
ENSSSCG00000004130		1291.62	-1.03	0.06	-17.19	0.00	0.00
ENSSSCG00000040479	NDE1	797.43	-1.03	0.08	-13.67	0.00	0.00
ENSSSCG00000033655	TUBB2B	5345.21	-1.03	0.05	-21.74	0.00	0.00
ENSSSCG00000005043	DDHD1	868.01	-1.03	0.07	-13.97	0.00	0.00
ENSSSCG00000000981	CRELD2	759.89	-1.03	0.07	-13.76	0.00	0.00
ENSSSCG00000024417	ERC2	17.21	-1.03	0.46	-2.22	0.03	0.04
ENSSSCG00000020870		1424.53	-1.03	0.06	-17.47	0.00	0.00
ENSSSCG00000037381	WRB	773.25	-1.03	0.07	-14.14	0.00	0.00
ENSSSCG00000017288	FTSJ3	2485.01	-1.03	0.06	-17.93	0.00	0.00
ENSSSCG00000030843	APLN	29.60	-1.03	0.33	-3.09	0.00	0.00
ENSSSCG00000027646	TIPARP	1164.70	-1.03	0.06	-16.00	0.00	0.00

ENSSSCG00000005729	DDX31	1221.09	-1.03	0.07	-15.70	0.00	0.00
ENSSSCG00000027767	SHLD2	62.82	-1.03	0.23	-4.38	0.00	0.00
ENSSSCG00000004195	ARG1	39.67	-1.02	0.30	-3.42	0.00	0.00
ENSSSCG00000009334	HSPH1	1566.84	-1.02	0.06	-17.82	0.00	0.00
ENSSSCG00000024285	DLG4	19.43	-1.02	0.41	-2.47	0.01	0.02
ENSSSCG00000002516	WARS	3013.98	-1.02	0.05	-19.96	0.00	0.00
ENSSSCG00000009887	NAA25	1728.56	-1.02	0.06	-17.83	0.00	0.00
ENSSSCG00000010186	URB2	1379.14	-1.02	0.06	-17.03	0.00	0.00
ENSSSCG00000039390	EIF3B	7067.24	-1.02	0.04	-26.14	0.00	0.00
ENSSSCG00000037999	PPP1R14B	1289.21	-1.02	0.06	-15.89	0.00	0.00
ENSSSCG00000036584	HAUS8	327.02	-1.02	0.11	-9.56	0.00	0.00
ENSSSCG00000014943	DEUP1	528.99	-1.02	0.09	-11.87	0.00	0.00
ENSSSCG00000032193	NKAIN1	273.06	-1.02	0.13	-8.02	0.00	0.00
ENSSSCG00000012307		1532.54	-1.02	0.06	-17.52	0.00	0.00
ENSSSCG00000037563	RF00002	22.98	-1.02	0.38	-2.69	0.01	0.01
ENSSSCG00000013775	ADGRE5	3238.25	-1.02	0.05	-20.65	0.00	0.00
ENSSSCG00000010479	RBP4	15971.22	-1.02	0.04	-25.89	0.00	0.00
ENSSSCG00000002290	PLEK2	802.32	-1.02	0.07	-13.98	0.00	0.00
ENSSSCG00000014075		2401.35	-1.02	0.05	-19.87	0.00	0.00
ENSSSCG00000032960	MANEAL	156.23	-1.02	0.15	-6.72	0.00	0.00
ENSSSCG00000009408	LRCH1	355.14	-1.02	0.10	-9.86	0.00	0.00
ENSSSCG00000027060	TBX2	184.57	-1.01	0.14	-7.06	0.00	0.00
ENSSSCG00000029577		1055.38	-1.01	0.06	-15.68	0.00	0.00
ENSSSCG00000022784	PLEKHO1	523.98	-1.01	0.09	-10.77	0.00	0.00
ENSSSCG00000015770	VEGFC	81.47	-1.01	0.21	-4.91	0.00	0.00

ENSSSCG00000003755	MCOLN2	130.62	-1.01	0.16	-6.28	0.00	0.00
ENSSSCG00000000721	DYRK4	19.53	-1.01	0.43	-2.38	0.02	0.03
ENSSSCG00000032024	URB1	1209.44	-1.01	0.06	-16.34	0.00	0.00
ENSSSCG00000036872		17.02	-1.01	0.45	-2.27	0.02	0.04
ENSSSCG00000032360	PANX1	425.30	-1.01	0.10	-10.17	0.00	0.00
ENSSSCG00000022864	EIF6	3188.14	-1.01	0.05	-21.82	0.00	0.00
ENSSSCG00000014031		9473.52	-1.01	0.04	-26.46	0.00	0.00
ENSSSCG00000024363		51.24	-1.01	0.26	-3.96	0.00	0.00
ENSSSCG00000008536	WDR43	3422.70	-1.01	0.05	-19.97	0.00	0.00
ENSSSCG00000027777		777.88	-1.01	0.07	-13.64	0.00	0.00
ENSSSCG00000017104	NSUN2	6807.80	-1.01	0.04	-25.84	0.00	0.00
ENSSSCG00000017306	ITGB3	4507.82	-1.01	0.04	-23.57	0.00	0.00
ENSSSCG00000008098	POLR1B	2364.76	-1.01	0.05	-18.51	0.00	0.00
ENSSSCG00000017525	NFE2L1	5352.68	-1.01	0.04	-23.74	0.00	0.00
ENSSSCG00000024018	SLC16A3	2794.07	-1.01	0.05	-20.42	0.00	0.00
ENSSSCG00000025344	PHC2	3067.76	-1.01	0.05	-20.09	0.00	0.00
ENSSSCG00000028949	NUP35	755.51	-1.00	0.07	-13.46	0.00	0.00
ENSSSCG00000039259		197.01	-1.00	0.14	-7.09	0.00	0.00
ENSSSCG00000024174	TGIF1	1405.63	-1.00	0.06	-15.72	0.00	0.00
ENSSSCG00000006524	THBS3	2740.98	-1.00	0.05	-20.54	0.00	0.00
ENSSSCG00000018942	RF00591	95.69	-1.00	0.20	-5.07	0.00	0.00
ENSSSCG00000011113	NUDT5	450.42	-1.00	0.10	-9.89	0.00	0.00
ENSSSCG00000036569	PTP4A3	59.34	-1.00	0.24	-4.20	0.00	0.00
ENSSSCG00000038534	TSR1	3474.29	-1.00	0.05	-22.20	0.00	0.00
ENSSSCG00000017116	LPCAT1	1156.86	-1.00	0.06	-15.43	0.00	0.00

ENSSSCG00000024131	MINDY2	347.01	1.00	0.11	9.08	0.00	0.00
ENSSSCG00000037951	ZNF891	13.50	1.00	0.50	1.99	0.05	0.07
ENSSSCG00000035594	PRSS22	247.25	1.00	0.12	8.03	0.00	0.00
ENSSSCG00000031180		44.16	1.00	0.30	3.34	0.00	0.00
ENSSSCG00000025284	COQ9	1197.90	1.00	0.07	15.24	0.00	0.00
ENSSSCG00000012548	PWWP3B	508.09	1.00	0.09	10.85	0.00	0.00
ENSSSCG00000021664	MAU2	2031.92	1.00	0.05	18.35	0.00	0.00
ENSSSCG00000038858	CFAP61	122.00	1.00	0.17	6.02	0.00	0.00
ENSSSCG00000036575	USHBP1	26.64	1.00	0.36	2.77	0.01	0.01
ENSSSCG00000011882	GOLGB1	3139.24	1.00	0.05	19.04	0.00	0.00
ENSSSCG00000037424	CDK19	350.36	1.00	0.10	9.63	0.00	0.00
ENSSSCG00000001779		1517.19	1.00	0.06	15.60	0.00	0.00
ENSSSCG00000000298	PPP1R1A	91.87	1.00	0.20	5.03	0.00	0.00
ENSSSCG00000008024	CLCN7	516.84	1.01	0.09	11.33	0.00	0.00
ENSSSCG00000031652	RPGRIP1L	887.58	1.01	0.07	13.81	0.00	0.00
ENSSSCG00000011369	WDR6	2186.82	1.01	0.05	19.05	0.00	0.00
ENSSSCG00000004680	TERB2	33.70	1.01	0.32	3.19	0.00	0.00
ENSSSCG00000000475	IRAK3	399.61	1.01	0.10	10.16	0.00	0.00
ENSSSCG00000033054	ROM1	51.80	1.01	0.25	3.95	0.00	0.00
ENSSSCG00000006179		1085.89	1.01	0.06	15.68	0.00	0.00
ENSSSCG00000010316	KAT6B	303.34	1.01	0.11	9.20	0.00	0.00
ENSSSCG00000003090	NECTIN2	547.48	1.01	0.09	11.38	0.00	0.00
ENSSSCG00000012335	FGD1	171.61	1.01	0.14	7.13	0.00	0.00
ENSSSCG00000021573	KCNJ5	6891.76	1.01	0.06	18.30	0.00	0.00
ENSSSCG00000016020	DUSP19	54.83	1.01	0.25	4.03	0.00	0.00

ENSSSCG00000014112	JMY	532.39	1.01	0.09	11.62	0.00	0.00
ENSSSCG00000022521	DPY19L3	386.04	1.01	0.10	10.28	0.00	0.00
ENSSSCG00000017300		40.88	1.02	0.30	3.37	0.00	0.00
ENSSSCG00000015595	ATF3	631.62	1.02	0.08	12.37	0.00	0.00
ENSSSCG00000011629	ACAD11	350.55	1.02	0.10	9.92	0.00	0.00
ENSSSCG00000023258	CLEC4F	30.82	1.02	0.33	3.08	0.00	0.00
ENSSSCG00000006767	MAGI3	1064.66	1.02	0.07	15.25	0.00	0.00
ENSSSCG00000040272	TTC29	215.25	1.02	0.13	7.61	0.00	0.00
ENSSSCG00000011497	MAGI1	482.78	1.02	0.10	10.65	0.00	0.00
ENSSSCG00000038945		19.22	1.02	0.42	2.44	0.01	0.02
ENSSSCG00000030065	ZBTB47	254.46	1.02	0.12	8.54	0.00	0.00
ENSSSCG00000010204	BICC1	10512.63	1.02	0.04	24.11	0.00	0.00
ENSSSCG00000005325	TMEM8B	69.89	1.02	0.22	4.60	0.00	0.00
ENSSSCG00000031744		136.62	1.02	0.16	6.37	0.00	0.00
ENSSSCG00000002427		354.99	1.02	0.11	9.42	0.00	0.00
ENSSSCG00000009932	MMAB	211.11	1.02	0.13	7.82	0.00	0.00
ENSSSCG00000040735	DDAH1	3411.79	1.02	0.05	21.37	0.00	0.00
ENSSSCG00000011746	SKIL	2020.90	1.02	0.05	19.51	0.00	0.00
ENSSSCG00000022292	NPL	27.87	1.02	0.35	2.96	0.00	0.01
ENSSSCG00000031877	ABHD8	105.34	1.03	0.18	5.61	0.00	0.00
ENSSSCG00000009671	PBK	1113.77	1.03	0.07	15.12	0.00	0.00
ENSSSCG00000008867	CTSO	32.44	1.03	0.32	3.16	0.00	0.00
ENSSSCG00000029201	AJUBA	2011.07	1.03	0.05	19.28	0.00	0.00
ENSSSCG00000027459	RNF13	919.50	1.03	0.07	14.36	0.00	0.00
ENSSSCG00000035999	NUDT16L1	53.27	1.03	0.26	3.94	0.00	0.00

ENSSSCG00000005212	PLGRKT	261.46	1.03	0.13	7.97	0.00	0.00
ENSSSCG00000008664	LRATD1	979.35	1.03	0.07	15.10	0.00	0.00
ENSSSCG00000004470	HMGN3	907.13	1.03	0.07	14.68	0.00	0.00
ENSSSCG00000005211	CD274	14.16	1.03	0.51	2.00	0.05	0.07
ENSSSCG00000023325		1422.51	1.03	0.06	16.46	0.00	0.00
ENSSSCG00000021793	SMIM14	670.51	1.03	0.08	12.25	0.00	0.00
ENSSSCG00000028997		1398.00	1.03	0.08	13.30	0.00	0.00
ENSSSCG00000011791	MAP3K13	903.18	1.03	0.07	14.92	0.00	0.00
ENSSSCG00000038954	PLIN3	321.18	1.03	0.11	9.67	0.00	0.00
ENSSSCG00000002988	ZNF546	91.53	1.03	0.19	5.35	0.00	0.00
ENSSSCG00000009505	MBNL2	1312.84	1.03	0.07	14.96	0.00	0.00
ENSSSCG00000003204	VRK3	350.32	1.03	0.10	9.93	0.00	0.00
ENSSSCG00000013351	NAV2	605.18	1.03	0.08	12.37	0.00	0.00
ENSSSCG00000009676	ZNF395	473.11	1.03	0.09	11.28	0.00	0.00
ENSSSCG00000032488	ZADH2	501.72	1.03	0.10	10.72	0.00	0.00
ENSSSCG00000010148	ERO1B	99.80	1.03	0.19	5.56	0.00	0.00
ENSSSCG00000031493	CYBA	342.40	1.04	0.11	9.07	0.00	0.00
ENSSSCG00000003059	ZNF428	105.85	1.04	0.19	5.41	0.00	0.00
ENSSSCG00000002357	ALDH6A1	990.38	1.04	0.07	14.89	0.00	0.00
ENSSSCG00000006588	S100A9	20.43	1.04	0.41	2.52	0.01	0.02
ENSSSCG00000008443	EPAS1	1165.57	1.04	0.07	14.74	0.00	0.00
ENSSSCG00000001070	FAM8A1	320.37	1.04	0.11	9.43	0.00	0.00
ENSSSCG00000004404		503.03	1.04	0.11	9.20	0.00	0.00
ENSSSCG00000040617	TNFAIP8	399.14	1.04	0.10	10.45	0.00	0.00
ENSSSCG00000003809	JAK1	4917.67	1.04	0.04	23.78	0.00	0.00

ENSSSCG00000040922	IP6K2	340.37	1.04	0.12	8.99	0.00	0.00
ENSSSCG00000022288	STK38L	246.68	1.04	0.12	8.61	0.00	0.00
ENSSSCG00000037427	PTDSS2	343.11	1.04	0.11	9.89	0.00	0.00
ENSSSCG00000017502	PGAP3	169.04	1.04	0.15	6.95	0.00	0.00
ENSSSCG00000008961	MTHFD2L	63.88	1.04	0.23	4.44	0.00	0.00
ENSSSCG00000021060	RCBTB1	1246.66	1.04	0.06	17.23	0.00	0.00
ENSSSCG00000016716	GSDME	37.86	1.04	0.31	3.38	0.00	0.00
ENSSSCG00000013664	C19orf66	32.64	1.04	0.34	3.10	0.00	0.00
ENSSSCG00000031913	NDUFB4	785.91	1.04	0.16	6.63	0.00	0.00
ENSSSCG00000010329	ZMIZ1	799.61	1.04	0.08	13.38	0.00	0.00
ENSSSCG00000003741		219.02	1.04	0.13	8.02	0.00	0.00
ENSSSCG00000016949		484.97	1.04	0.09	11.32	0.00	0.00
ENSSSCG00000006779	ST7L	306.09	1.04	0.11	9.12	0.00	0.00
ENSSSCG00000010182	CAPN9	151.50	1.04	0.16	6.59	0.00	0.00
ENSSSCG00000007778	CCDC189	51.20	1.05	0.26	4.03	0.00	0.00
ENSSSCG00000016187	CATIP	95.21	1.05	0.19	5.50	0.00	0.00
ENSSSCG00000013339	ANO3	224.88	1.05	0.13	8.19	0.00	0.00
ENSSSCG00000011082	COMMD3	411.18	1.05	0.10	10.86	0.00	0.00
ENSSSCG00000001975	PRKD1	170.49	1.05	0.15	6.97	0.00	0.00
ENSSSCG00000004684	SPG11	1153.29	1.05	0.07	15.89	0.00	0.00
ENSSSCG00000029392	HUNK	915.77	1.05	0.07	14.97	0.00	0.00
ENSSSCG00000010027	PATZ1	366.74	1.05	0.10	10.40	0.00	0.00
ENSSSCG00000011721	P2RY1	246.89	1.05	0.13	8.31	0.00	0.00
ENSSSCG00000038486	WDR60	123.62	1.05	0.17	6.02	0.00	0.00
ENSSSCG00000004133	FUCA2	1108.79	1.05	0.07	16.10	0.00	0.00

ENSSSCG00000017566	ACSF2	1267.54	1.05	0.06	16.81	0.00	0.00
ENSSSCG00000005305	UNC13B	953.64	1.05	0.07	15.20	0.00	0.00
ENSSSCG00000027525	DHCR24	4799.92	1.05	0.04	23.94	0.00	0.00
ENSSSCG00000009836	ACAD10	764.80	1.05	0.08	13.89	0.00	0.00
ENSSSCG00000023264		3556.40	1.05	0.05	22.64	0.00	0.00
ENSSSCG00000005716	POMT1	449.13	1.05	0.09	11.19	0.00	0.00
ENSSSCG00000004144	HECA	46.25	1.05	0.28	3.82	0.00	0.00
ENSSSCG00000024570	KDM4B	689.05	1.05	0.08	13.46	0.00	0.00
ENSSSCG00000028190	HOMEZ	358.86	1.05	0.10	10.15	0.00	0.00
ENSSSCG00000006369	F11R	1081.91	1.05	0.07	14.75	0.00	0.00
ENSSSCG00000024793	PORCN	131.07	1.05	0.16	6.49	0.00	0.00
ENSSSCG00000021238	STX1B	91.05	1.06	0.21	5.04	0.00	0.00
ENSSSCG00000009831	CUX2	305.10	1.06	0.11	9.33	0.00	0.00
ENSSSCG00000034332		14.92	1.06	0.48	2.21	0.03	0.04
ENSSSCG00000016863	OXCT1	1008.46	1.06	0.08	13.98	0.00	0.00
ENSSSCG00000000728	PARP11	84.42	1.06	0.20	5.20	0.00	0.00
ENSSSCG00000004082		329.51	1.06	0.11	9.88	0.00	0.00
ENSSSCG00000005308	RUSC2	79.40	1.06	0.21	5.05	0.00	0.00
ENSSSCG00000012604	LAMP2	4242.99	1.06	0.05	23.11	0.00	0.00
ENSSSCG00000026863	FARP1	3354.17	1.06	0.05	22.83	0.00	0.00
ENSSSCG00000012875		418.28	1.06	0.10	10.74	0.00	0.00
ENSSSCG00000027857	DMXL1	2403.49	1.06	0.06	18.02	0.00	0.00
ENSSSCG00000004507	SMAD7	329.59	1.06	0.11	9.94	0.00	0.00
ENSSSCG00000012828	STARD8	467.22	1.06	0.09	11.42	0.00	0.00
ENSSSCG00000039368		117.23	1.06	0.18	6.00	0.00	0.00

ENSSSCG00000040838	AGBL2	431.01	1.06	0.09	11.26	0.00	0.00
ENSSSCG00000011014	BAMBI	59.46	1.06	0.24	4.37	0.00	0.00
ENSSSCG00000000838	SLC41A2	439.09	1.06	0.10	10.92	0.00	0.00
ENSSSCG00000008590	MFSD2B	14.96	1.06	0.50	2.14	0.03	0.05
ENSSSCG00000005180	BNC2	407.48	1.07	0.10	10.82	0.00	0.00
ENSSSCG00000012551	RNF128	591.44	1.07	0.08	12.84	0.00	0.00
ENSSSCG00000032501		76.46	1.07	0.21	4.99	0.00	0.00
ENSSSCG00000017152	SGSH	584.47	1.07	0.08	13.02	0.00	0.00
ENSSSCG00000002653		27.94	1.07	0.36	3.00	0.00	0.00
ENSSSCG00000004154	TNFAIP3	767.10	1.07	0.09	11.44	0.00	0.00
ENSSSCG00000003459	CELA2A	146.41	1.07	0.15	6.92	0.00	0.00
ENSSSCG00000025210	ATG10	199.85	1.07	0.14	7.52	0.00	0.00
ENSSSCG00000038530	TMEM240	25.88	1.07	0.36	2.95	0.00	0.01
ENSSSCG00000039034	DNALI1	32.12	1.07	0.33	3.28	0.00	0.00
ENSSSCG00000000006	PPARA	39.46	1.07	0.31	3.47	0.00	0.00
ENSSSCG00000009916	ACADS	1130.73	1.07	0.07	15.79	0.00	0.00
ENSSSCG00000010047	RSPH14	39.93	1.07	0.30	3.60	0.00	0.00
ENSSSCG00000006535	PMVK	123.96	1.07	0.17	6.46	0.00	0.00
ENSSSCG00000038600	PRSS8	36.83	1.07	0.32	3.40	0.00	0.00
ENSSSCG00000026990	FAM162A	347.96	1.08	0.12	8.81	0.00	0.00
ENSSSCG00000016929	PDE4D	718.30	1.08	0.08	13.91	0.00	0.00
ENSSSCG00000033367	PLEKHA2	1458.17	1.08	0.06	18.65	0.00	0.00
ENSSSCG00000006308	CREG1	788.57	1.08	0.07	14.42	0.00	0.00
ENSSSCG00000012519	GPRASP1	116.99	1.08	0.18	5.97	0.00	0.00
ENSSSCG00000008645	ID2	602.42	1.08	0.09	12.08	0.00	0.00

ENSSSCG00000006625	RFX5	740.31	1.08	0.08	14.31	0.00	0.00
ENSSSCG00000028805	PTPRM	910.32	1.08	0.08	14.25	0.00	0.00
ENSSSCG00000003486	ARHGEF10L	1103.78	1.08	0.08	14.31	0.00	0.00
ENSSSCG00000037420	TMEM170B	85.34	1.08	0.20	5.41	0.00	0.00
ENSSSCG00000013039	NUDT22	140.31	1.08	0.16	6.80	0.00	0.00
ENSSSCG00000040450		17.75	1.09	0.44	2.47	0.01	0.02
ENSSSCG00000010219	ARID5B	1721.33	1.09	0.06	18.42	0.00	0.00
ENSSSCG00000007717	METTL27	123.13	1.09	0.17	6.38	0.00	0.00
ENSSSCG00000008003	HAGHL	53.25	1.09	0.26	4.24	0.00	0.00
ENSSSCG00000005221	SPATA6L	87.24	1.09	0.20	5.34	0.00	0.00
ENSSSCG00000026367	IFT172	1073.66	1.09	0.07	16.64	0.00	0.00
ENSSSCG00000008969	RCHY1	467.10	1.09	0.09	11.75	0.00	0.00
ENSSSCG00000034062	SV2C	13.03	1.09	0.51	2.13	0.03	0.05
ENSSSCG00000039907		17.24	1.09	0.45	2.44	0.01	0.02
ENSSSCG00000010854	TMEM63A	1078.48	1.09	0.07	16.22	0.00	0.00
ENSSSCG00000000808	SLC38A2	7208.56	1.09	0.05	22.90	0.00	0.00
ENSSSCG00000022901	KLHDC3	572.43	1.09	0.09	12.84	0.00	0.00
ENSSSCG00000007492	FAM210B	517.66	1.09	0.09	12.18	0.00	0.00
ENSSSCG00000038853	GPR137B	240.28	1.09	0.13	8.45	0.00	0.00
ENSSSCG00000004414		25396.71	1.09	0.04	29.00	0.00	0.00
ENSSSCG00000014141	RFESD	216.91	1.09	0.14	7.91	0.00	0.00
ENSSSCG00000015083	FXYD6	233.13	1.09	0.13	8.13	0.00	0.00
ENSSSCG00000024604	ATAT1	52.36	1.09	0.26	4.27	0.00	0.00
ENSSSCG00000010721	PSTK	56.67	1.10	0.25	4.40	0.00	0.00
ENSSSCG00000013393	SPON1	16.20	1.10	0.48	2.29	0.02	0.03

ENSSSCG00000036326	LATS2	767.47	1.10	0.08	13.63	0.00	0.00
ENSSSCG00000007155	C20orf194	862.16	1.10	0.08	14.05	0.00	0.00
ENSSSCG00000035657	DIP2A	499.02	1.10	0.09	12.42	0.00	0.00
ENSSSCG00000023593	AFF1	869.68	1.10	0.07	15.32	0.00	0.00
ENSSSCG00000026636	ZBTB4	241.28	1.10	0.13	8.57	0.00	0.00
ENSSSCG00000015113	NLRX1	486.23	1.10	0.09	12.06	0.00	0.00
ENSSSCG00000016147	PIKFYVE	1270.40	1.10	0.06	17.41	0.00	0.00
ENSSSCG00000016650		28.35	1.10	0.35	3.17	0.00	0.00
ENSSSCG00000038739	MYPOP	19.44	1.10	0.42	2.62	0.01	0.01
ENSSSCG00000009678	FBXO16	78.24	1.10	0.21	5.25	0.00	0.00
ENSSSCG00000016872	HMGCS1	2679.17	1.10	0.06	17.61	0.00	0.00
ENSSSCG00000016831	SPEF2	177.35	1.10	0.14	7.67	0.00	0.00
ENSSSCG00000038693	RAB19	80.01	1.11	0.21	5.25	0.00	0.00
ENSSSCG00000036294	NUDT19	505.14	1.11	0.09	12.32	0.00	0.00
ENSSSCG00000006635	SEMA6C	13.64	1.11	0.51	2.16	0.03	0.05
ENSSSCG00000001484	TINAG	1109.07	1.11	0.07	15.85	0.00	0.00
ENSSSCG00000006940	CCN1	1177.30	1.11	0.07	15.66	0.00	0.00
ENSSSCG00000028180	DUSP28	292.29	1.11	0.12	9.28	0.00	0.00
ENSSSCG00000005207	ERMP1	2139.28	1.11	0.05	20.44	0.00	0.00
ENSSSCG00000017818	SERPINF1	187.88	1.11	0.15	7.59	0.00	0.00
ENSSSCG00000000148		119.63	1.11	0.18	6.33	0.00	0.00
ENSSSCG00000016836	NADK2	860.77	1.11	0.07	15.04	0.00	0.00
ENSSSCG00000010559		22.64	1.11	0.39	2.82	0.00	0.01
ENSSSCG00000036843		189.85	1.11	0.14	7.88	0.00	0.00
ENSSSCG00000026167		22.17	1.11	0.39	2.84	0.00	0.01

ENSSSCG00000032674	TCEA3	421.39	1.11	0.09	11.79	0.00	0.00
ENSSSCG00000025545	HDAC6	2717.40	1.11	0.05	22.54	0.00	0.00
ENSSSCG00000000003	TTC38	306.53	1.11	0.11	9.90	0.00	0.00
ENSSSCG00000007935		25.94	1.12	0.37	3.05	0.00	0.00
ENSSSCG00000003582	SMPDL3B	149.67	1.12	0.16	7.05	0.00	0.00
ENSSSCG00000013246	C11orf49	94.21	1.12	0.20	5.68	0.00	0.00
ENSSSCG00000015276	ETNK2	13.26	1.12	0.53	2.12	0.03	0.05
ENSSSCG00000009994	MTMR3	1385.59	1.12	0.06	18.94	0.00	0.00
ENSSSCG00000032444	PLXDC2	182.14	1.12	0.14	7.79	0.00	0.00
ENSSSCG00000000111	BAIAP2L2	504.56	1.12	0.09	12.10	0.00	0.00
ENSSSCG00000017343	GFAP	132.27	1.12	0.17	6.60	0.00	0.00
ENSSSCG00000039793	PATJ	3375.23	1.12	0.05	21.47	0.00	0.00
ENSSSCG00000016956	MAST4	210.82	1.12	0.13	8.55	0.00	0.00
ENSSSCG00000034398	C8orf37	212.13	1.12	0.14	8.02	0.00	0.00
ENSSSCG00000034348	NBEA	649.88	1.12	0.08	13.40	0.00	0.00
ENSSSCG00000036169	UMAD1	331.72	1.12	0.11	10.13	0.00	0.00
ENSSSCG00000011643	AMOTL2	1621.54	1.12	0.06	19.98	0.00	0.00
ENSSSCG00000031963	SAXO2	101.40	1.12	0.19	6.02	0.00	0.00
ENSSSCG00000000517	CAPS2	37.71	1.12	0.30	3.72	0.00	0.00
ENSSSCG00000012326	RIBC1	31.87	1.12	0.33	3.46	0.00	0.00
ENSSSCG00000026018	MFSD1	148.25	1.12	0.16	6.99	0.00	0.00
ENSSSCG00000010485	TBC1D12	739.19	1.12	0.08	14.87	0.00	0.00
ENSSSCG00000012318	MAGED1	1774.49	1.13	0.07	17.03	0.00	0.00
ENSSSCG00000013440	ATP8B3	49.39	1.13	0.28	4.02	0.00	0.00
ENSSSCG00000006294	NME7	366.66	1.13	0.10	10.81	0.00	0.00

ENSSSCG00000010798	GGA2	352.48	1.13	0.11	10.74	0.00	0.00
ENSSSCG00000025992	ENPP3	52.13	1.13	0.26	4.36	0.00	0.00
ENSSSCG00000000396	STAT2	302.43	1.13	0.11	10.14	0.00	0.00
ENSSSCG00000032644	HLX	27.12	1.13	0.36	3.17	0.00	0.00
ENSSSCG00000006066	RNF19A	841.38	1.13	0.07	15.69	0.00	0.00
ENSSSCG00000024736	ACP4	155.60	1.13	0.15	7.47	0.00	0.00
ENSSSCG00000028506	YIPF1	647.85	1.13	0.09	12.96	0.00	0.00
ENSSSCG00000001952	NFKBIA	1364.93	1.13	0.08	14.43	0.00	0.00
ENSSSCG00000038943	TOM1	605.48	1.13	0.08	13.33	0.00	0.00
ENSSSCG00000017915	VMO1	104.00	1.13	0.18	6.24	0.00	0.00
ENSSSCG00000025425	FCSK	248.39	1.13	0.13	8.74	0.00	0.00
ENSSSCG00000014380		19.21	1.13	0.42	2.69	0.01	0.01
ENSSSCG00000022773		203.94	1.14	0.14	8.11	0.00	0.00
ENSSSCG00000013572	PNPLA6	811.70	1.14	0.07	15.61	0.00	0.00
ENSSSCG00000035790	BTG1	1499.80	1.14	0.06	19.96	0.00	0.00
ENSSSCG00000017340	DCAKD	584.57	1.14	0.09	12.84	0.00	0.00
ENSSSCG00000030484	AHR	855.31	1.14	0.07	15.62	0.00	0.00
ENSSSCG00000007066	SLX4IP	183.60	1.14	0.14	8.23	0.00	0.00
ENSSSCG00000024346	SLC7A3	52.47	1.14	0.26	4.43	0.00	0.00
ENSSSCG00000005924	MROH1	930.30	1.14	0.07	16.51	0.00	0.00
ENSSSCG00000031102	C1orf174	224.34	1.14	0.13	8.63	0.00	0.00
ENSSSCG00000034997		38.49	1.14	0.33	3.45	0.00	0.00
ENSSSCG00000003402	PGD	5336.63	1.14	0.05	22.93	0.00	0.00
ENSSSCG00000009240	PLAC8	25.17	1.14	0.37	3.12	0.00	0.00
ENSSSCG00000035897		11.23	1.14	0.55	2.09	0.04	0.06

ENSSSCG00000031550		15.02	1.14	0.48	2.36	0.02	0.03
ENSSSCG00000013978	ZNF672	178.66	1.15	0.14	8.03	0.00	0.00
ENSSSCG00000019323	RF00396	22.56	1.15	0.38	2.99	0.00	0.01
ENSSSCG00000011689	PLOD2	2700.99	1.15	0.06	19.47	0.00	0.00
ENSSSCG00000032620	PLCXD2	74.06	1.15	0.23	5.08	0.00	0.00
ENSSSCG00000035249	GADD45G	18.29	1.15	0.46	2.51	0.01	0.02
ENSSSCG00000023539	MBOAT2	100.60	1.15	0.20	5.84	0.00	0.00
ENSSSCG00000004561	HERC1	1559.43	1.15	0.06	20.01	0.00	0.00
ENSSSCG00000009217	PKD2	2595.78	1.15	0.05	21.26	0.00	0.00
ENSSSCG00000038383	DYNC2LI1	1093.33	1.15	0.07	15.83	0.00	0.00
ENSSSCG00000008952	RASSF6	172.51	1.15	0.15	7.58	0.00	0.00
ENSSSCG00000020871	ARSD	400.47	1.15	0.10	11.63	0.00	0.00
ENSSSCG00000016050	INPP1	309.24	1.15	0.11	10.53	0.00	0.00
ENSSSCG00000011386	MST1	32.93	1.16	0.33	3.51	0.00	0.00
ENSSSCG00000009824	RAD9B	24.80	1.16	0.38	3.02	0.00	0.00
ENSSSCG00000031707	CTDSP2	289.09	1.16	0.12	9.90	0.00	0.00
ENSSSCG00000004415		85.18	1.16	0.21	5.61	0.00	0.00
ENSSSCG00000010765	INPP5A	140.67	1.16	0.16	7.23	0.00	0.00
ENSSSCG00000014197	SLC25A46	728.30	1.16	0.08	14.97	0.00	0.00
ENSSSCG00000037391		69.67	1.16	0.22	5.15	0.00	0.00
ENSSSCG00000036274		13391.85	1.16	0.04	27.10	0.00	0.00
ENSSSCG00000017054	CYFIP2	4259.94	1.16	0.05	24.65	0.00	0.00
ENSSSCG00000009801	BCL7A	466.35	1.16	0.09	12.23	0.00	0.00
ENSSSCG00000024587	DNASE1L2	10.84	1.16	0.57	2.04	0.04	0.06
ENSSSCG00000010543	ABCC2	8476.84	1.16	0.04	28.46	0.00	0.00

ENSSSCG00000002712	LDHD	1146.82	1.16	0.07	17.11	0.00	0.00
ENSSSCG00000034474		98.33	1.16	0.19	6.05	0.00	0.00
ENSSSCG00000001720	SLC25A27	414.41	1.16	0.10	11.41	0.00	0.00
ENSSSCG00000002337	ZFYVE1	387.61	1.16	0.10	11.21	0.00	0.00
ENSSSCG00000008796	RBM47	3100.83	1.16	0.05	22.81	0.00	0.00
ENSSSCG00000017393	CNTNAP1	60.09	1.16	0.25	4.69	0.00	0.00
ENSSSCG00000021204	HOXA10	785.51	1.16	0.08	15.45	0.00	0.00
ENSSSCG00000024752	ALDH4A1	3230.19	1.16	0.05	24.73	0.00	0.00
ENSSSCG00000039658		3545.54	1.16	0.05	23.99	0.00	0.00
ENSSSCG00000010036	SLC5A1	55.26	1.17	0.26	4.51	0.00	0.00
ENSSSCG00000039374		20.07	1.17	0.42	2.81	0.00	0.01
ENSSSCG00000014123	FAM151B	27.60	1.17	0.36	3.28	0.00	0.00
ENSSSCG00000009268	CRYL1	159.89	1.17	0.16	7.46	0.00	0.00
ENSSSCG00000022429	KAZALD1	38.52	1.17	0.30	3.88	0.00	0.00
ENSSSCG00000028682		321.69	1.17	0.11	10.73	0.00	0.00
ENSSSCG00000022846	SLC4A3	30.94	1.17	0.34	3.46	0.00	0.00
ENSSSCG00000025106		221.41	1.17	0.14	8.59	0.00	0.00
ENSSSCG00000037781		83.04	1.17	0.22	5.25	0.00	0.00
ENSSSCG00000008857	MSMO1	1746.79	1.17	0.06	18.23	0.00	0.00
ENSSSCG00000008238	ELMOD3	672.89	1.17	0.08	14.90	0.00	0.00
ENSSSCG00000035593		382.57	1.17	0.10	11.50	0.00	0.00
ENSSSCG00000004648	FAM227B	26.08	1.17	0.37	3.14	0.00	0.00
ENSSSCG00000036172	SP9	46.73	1.17	0.28	4.20	0.00	0.00
ENSSSCG00000010534	HPS1	245.05	1.17	0.12	9.55	0.00	0.00
ENSSSCG00000034191	SOX6	912.39	1.17	0.07	16.69	0.00	0.00

ENSSSCG00000000437	ARHGAP9	41.85	1.17	0.29	4.11	0.00	0.00
ENSSSCG00000038539		25.00	1.17	0.37	3.15	0.00	0.00
ENSSSCG00000003747	KIAA1328	58.23	1.17	0.25	4.74	0.00	0.00
ENSSSCG00000003167	FLT3LG	55.55	1.18	0.25	4.68	0.00	0.00
ENSSSCG00000012000	GBE1	1015.22	1.18	0.07	17.24	0.00	0.00
ENSSSCG00000010926	SYT2	55.52	1.18	0.25	4.63	0.00	0.00
ENSSSCG00000007941	CDIP1	256.12	1.18	0.14	8.55	0.00	0.00
ENSSSCG00000040162	NUPR1	197.59	1.18	0.14	8.38	0.00	0.00
ENSSSCG00000011522	CNTN3	140.64	1.18	0.16	7.14	0.00	0.00
ENSSSCG00000040603	SGTB	79.53	1.18	0.21	5.56	0.00	0.00
ENSSSCG00000034879	MAML2	862.24	1.18	0.07	16.01	0.00	0.00
ENSSSCG00000001064	GMPR	194.62	1.18	0.14	8.68	0.00	0.00
ENSSSCG00000003783	FPGT	308.96	1.18	0.11	10.45	0.00	0.00
ENSSSCG00000035941	DSEL	186.04	1.18	0.14	8.47	0.00	0.00
ENSSSCG00000026798		28.40	1.18	0.35	3.39	0.00	0.00
ENSSSCG00000009357	SMAD9	164.60	1.18	0.15	8.08	0.00	0.00
ENSSSCG00000015871	NR4A2	157.18	1.19	0.16	7.56	0.00	0.00
ENSSSCG00000017608		529.21	1.19	0.09	12.67	0.00	0.00
ENSSSCG00000023665	FBXO25	190.13	1.19	0.14	8.53	0.00	0.00
ENSSSCG00000034272	DENND6B	684.67	1.19	0.08	14.52	0.00	0.00
ENSSSCG00000026044	FDFT1	2049.74	1.19	0.06	20.81	0.00	0.00
ENSSSCG00000038918	CTSF	531.86	1.19	0.09	13.22	0.00	0.00
ENSSSCG00000036135	COL1A1	162.77	1.19	0.15	7.79	0.00	0.00
ENSSSCG00000032566	ZHX3	242.78	1.19	0.12	9.75	0.00	0.00
ENSSSCG00000016035	COL5A2	14.25	1.19	0.50	2.39	0.02	0.03

ENSSSCG00000029413	DNMT3A	222.22	1.19	0.13	9.26	0.00	0.00
ENSSSCG00000001582	MDGA1	77.49	1.19	0.22	5.47	0.00	0.00
ENSSSCG00000036790	AKAP7	57.20	1.19	0.25	4.74	0.00	0.00
ENSSSCG00000038558	GOLPH3L	2207.57	1.20	0.06	20.89	0.00	0.00
ENSSSCG00000008722		610.34	1.20	0.08	14.17	0.00	0.00
ENSSSCG00000006194	NCOA2	2670.31	1.20	0.06	20.57	0.00	0.00
ENSSSCG00000008001		96.75	1.20	0.19	6.21	0.00	0.00
ENSSSCG00000015981	HOXD10	11.04	1.20	0.59	2.02	0.04	0.07
ENSSSCG00000040296	EHHADH	4644.47	1.20	0.04	26.81	0.00	0.00
ENSSSCG00000007668	GIGYF1	430.95	1.20	0.10	12.20	0.00	0.00
ENSSSCG00000040890	ABHD14B	293.16	1.20	0.12	10.21	0.00	0.00
ENSSSCG00000005905	SLC39A4	15.44	1.20	0.47	2.53	0.01	0.02
ENSSSCG00000010180	TTC13	59.01	1.20	0.24	4.95	0.00	0.00
ENSSSCG00000027565		70.53	1.20	0.22	5.41	0.00	0.00
ENSSSCG00000013294	LDLRAD3	146.72	1.20	0.17	7.05	0.00	0.00
ENSSSCG00000004859	ZNF516	321.63	1.20	0.11	11.09	0.00	0.00
ENSSSCG00000028461		1023.71	1.21	0.07	17.72	0.00	0.00
ENSSSCG00000005506	MEGF9	278.88	1.21	0.12	10.38	0.00	0.00
ENSSSCG00000029002	PNKD	2530.99	1.21	0.06	20.77	0.00	0.00
ENSSSCG00000005309	FAM166B	13.31	1.21	0.51	2.35	0.02	0.03
ENSSSCG00000022434	ZNF235	9.99	1.21	0.61	2.00	0.05	0.07
ENSSSCG00000031316		54.79	1.21	0.27	4.47	0.00	0.00
ENSSSCG00000002252	ARRDC4	336.70	1.21	0.12	10.44	0.00	0.00
ENSSSCG00000011145	ASB13	521.46	1.21	0.09	13.89	0.00	0.00
ENSSSCG00000004136	AIG1	587.30	1.21	0.09	13.54	0.00	0.00

ENSSSCG00000007456	SULF2	576.02	1.21	0.09	13.85	0.00	0.00
ENSSSCG00000004132	PHACTR2	168.05	1.21	0.15	8.22	0.00	0.00
ENSSSCG00000035281	ZNF292	1000.58	1.21	0.08	16.10	0.00	0.00
ENSSSCG00000023178	BATF2	130.90	1.21	0.17	7.34	0.00	0.00
ENSSSCG00000026113	ZBTB20	237.36	1.21	0.13	9.40	0.00	0.00
ENSSSCG00000011404	HYAL1	319.35	1.22	0.11	10.63	0.00	0.00
ENSSSCG00000025413		42.23	1.22	0.29	4.19	0.00	0.00
ENSSSCG00000013260		1344.21	1.22	0.06	19.01	0.00	0.00
ENSSSCG00000037158	MTURN	118.95	1.22	0.19	6.46	0.00	0.00
ENSSSCG00000038643	KLF11	161.06	1.22	0.15	8.14	0.00	0.00
ENSSSCG00000021270	THAP2	445.28	1.22	0.10	11.69	0.00	0.00
ENSSSCG00000015998	PJVK	16.12	1.22	0.47	2.61	0.01	0.02
ENSSSCG00000010589	SFXN2	1205.30	1.22	0.06	19.00	0.00	0.00
ENSSSCG00000027278	ARL6	473.95	1.22	0.09	12.98	0.00	0.00
ENSSSCG00000034351	B3GALT1	338.99	1.22	0.11	11.42	0.00	0.00
ENSSSCG00000028015	GAL3ST3	71.82	1.22	0.22	5.58	0.00	0.00
ENSSSCG00000016208	ZFAND2B	1472.43	1.22	0.06	19.15	0.00	0.00
ENSSSCG00000007252	DNMT3B	432.82	1.22	0.10	12.21	0.00	0.00
ENSSSCG00000033350		93.00	1.22	0.20	6.05	0.00	0.00
ENSSSCG00000037562	SLC2A4RG	516.41	1.22	0.09	13.39	0.00	0.00
ENSSSCG00000011511	FRMD4B	514.24	1.22	0.09	14.04	0.00	0.00
ENSSSCG00000038940	GNPDA1	674.74	1.22	0.08	15.82	0.00	0.00
ENSSSCG00000011254	XYLB	238.18	1.23	0.13	9.60	0.00	0.00
ENSSSCG00000027169	ENOSF1	62.46	1.23	0.24	5.13	0.00	0.00
ENSSSCG00000030921	APOA1	901.39	1.23	0.09	14.04	0.00	0.00

ENSSSCG00000012452	SH3BGRL	704.79	1.23	0.09	13.49	0.00	0.00
ENSSSCG00000032145	IRX5	564.93	1.23	0.08	14.73	0.00	0.00
ENSSSCG00000033305	PGBD2	10.63	1.23	0.56	2.18	0.03	0.05
ENSSSCG00000037572	EPSTI1	81.16	1.23	0.21	5.77	0.00	0.00
ENSSSCG00000012926	PC	210.20	1.23	0.13	9.33	0.00	0.00
ENSSSCG00000006766	PHTF1	226.51	1.23	0.13	9.69	0.00	0.00
ENSSSCG00000028943	ECH1	240.57	1.23	0.13	9.69	0.00	0.00
ENSSSCG00000019803	RF00019	19.59	1.23	0.42	2.95	0.00	0.01
ENSSSCG00000038580		247.18	1.23	0.12	9.95	0.00	0.00
ENSSSCG00000034198		56.53	1.23	0.25	4.95	0.00	0.00
ENSSSCG00000016261	SP110	375.55	1.23	0.10	12.00	0.00	0.00
ENSSSCG00000027074		491.86	1.23	0.09	13.58	0.00	0.00
ENSSSCG00000012933	CCDC87	41.42	1.23	0.29	4.27	0.00	0.00
ENSSSCG00000003428	MTHFR	198.57	1.24	0.14	8.82	0.00	0.00
ENSSSCG00000016416	SHH	26.38	1.24	0.36	3.41	0.00	0.00
ENSSSCG00000009666	EPHX2	519.07	1.24	0.09	13.31	0.00	0.00
ENSSSCG00000004134	PEX3	387.95	1.24	0.10	12.40	0.00	0.00
ENSSSCG00000023957	CFAP126	11.21	1.24	0.56	2.22	0.03	0.04
ENSSSCG00000009018	SH3D19	997.39	1.24	0.07	17.05	0.00	0.00
ENSSSCG00000035757	SSBP2	362.94	1.24	0.11	11.71	0.00	0.00
ENSSSCG00000039080	TRIB2	669.29	1.24	0.08	15.94	0.00	0.00
ENSSSCG00000030560	IGF1R	5191.71	1.24	0.05	26.47	0.00	0.00
ENSSSCG00000000837	CHST11	421.76	1.24	0.10	12.43	0.00	0.00
ENSSSCG00000016684	SCRN1	1776.46	1.24	0.06	22.36	0.00	0.00
ENSSSCG00000033312		48.53	1.25	0.28	4.48	0.00	0.00

ENSSSCG00000016156	ACADL	517.54	1.25	0.09	13.79	0.00	0.00
ENSSSCG00000000530	FGD4	109.31	1.25	0.18	6.84	0.00	0.00
ENSSSCG00000032684	BOK	368.44	1.25	0.11	11.51	0.00	0.00
ENSSSCG00000009338		29.92	1.25	0.34	3.66	0.00	0.00
ENSSSCG00000035511	C1QTNF1	14.08	1.25	0.53	2.35	0.02	0.03
ENSSSCG00000022797	PPP1R3B	23.19	1.25	0.41	3.07	0.00	0.00
ENSSSCG00000006572	NPR1	11.82	1.25	0.57	2.19	0.03	0.04
ENSSSCG00000029652	TMBIM6	7344.63	1.25	0.05	24.88	0.00	0.00
ENSSSCG00000037065	EMP2	1254.35	1.25	0.07	18.78	0.00	0.00
ENSSSCG00000006997	TRIML2	108.50	1.25	0.18	6.80	0.00	0.00
ENSSSCG00000010968		247.58	1.25	0.12	10.34	0.00	0.00
ENSSSCG00000005514	RAB14	58.29	1.25	0.25	5.08	0.00	0.00
ENSSSCG00000005424	NIPSNAP3B	1431.60	1.25	0.06	21.04	0.00	0.00
ENSSSCG00000008347	FBXO48	13.58	1.25	0.52	2.43	0.02	0.02
ENSSSCG00000012627		33.39	1.25	0.33	3.80	0.00	0.00
ENSSSCG00000012792	RENBP	682.14	1.25	0.08	15.70	0.00	0.00
ENSSSCG00000010571	ARMH3	710.73	1.26	0.08	15.91	0.00	0.00
ENSSSCG00000015135	SORL1	2337.94	1.26	0.06	21.36	0.00	0.00
ENSSSCG00000039756	FOXC1	32.25	1.26	0.34	3.75	0.00	0.00
ENSSSCG00000027198	PDK2	271.38	1.26	0.12	10.70	0.00	0.00
ENSSSCG00000026689		185.98	1.26	0.14	8.85	0.00	0.00
ENSSSCG00000012071		153.73	1.26	0.16	7.92	0.00	0.00
ENSSSCG00000000206	FAIM2	305.06	1.26	0.11	11.33	0.00	0.00
ENSSSCG00000036755	TENT5B	490.40	1.26	0.09	13.57	0.00	0.00
ENSSSCG00000040476		14.18	1.26	0.50	2.54	0.01	0.02

ENSSSCG00000017791	SSH2	1759.71	1.26	0.06	22.80	0.00	0.00
ENSSSCG00000021586	ZHX2	103.97	1.26	0.19	6.77	0.00	0.00
ENSSSCG00000015735	PTPN18	305.93	1.27	0.11	11.38	0.00	0.00
ENSSSCG00000008422		543.65	1.27	0.09	14.05	0.00	0.00
ENSSSCG00000002637		19.95	1.27	0.42	3.05	0.00	0.00
ENSSSCG00000016262		851.93	1.27	0.07	17.24	0.00	0.00
ENSSSCG00000017520	LRRC46	76.75	1.27	0.22	5.68	0.00	0.00
ENSSSCG00000038991	S100A13	173.06	1.27	0.15	8.74	0.00	0.00
ENSSSCG00000035153	TRIM38	31.45	1.27	0.38	3.35	0.00	0.00
ENSSSCG00000011888	GPR156	9.13	1.27	0.62	2.06	0.04	0.06
ENSSSCG00000016725	TNS3	34.28	1.27	0.32	3.93	0.00	0.00
ENSSSCG00000039821	GPRIN3	101.01	1.27	0.19	6.63	0.00	0.00
ENSSSCG00000023498	HSPB6	348.10	1.27	0.13	9.89	0.00	0.00
ENSSSCG00000021414		92.99	1.27	0.21	6.11	0.00	0.00
ENSSSCG00000000091	APOBEC3B	185.06	1.27	0.14	9.01	0.00	0.00
ENSSSCG00000040732	WDR81	503.78	1.28	0.09	13.69	0.00	0.00
ENSSSCG00000028977	DOK6	35.46	1.28	0.31	4.07	0.00	0.00
ENSSSCG00000017128		114.83	1.28	0.18	7.20	0.00	0.00
ENSSSCG00000011895	PLA1A	96.65	1.28	0.19	6.70	0.00	0.00
ENSSSCG00000031787		14.90	1.28	0.49	2.60	0.01	0.02
ENSSSCG00000016223	ACSL3	4616.16	1.28	0.05	27.26	0.00	0.00
ENSSSCG00000029438	SESN2	370.04	1.28	0.10	12.36	0.00	0.00
ENSSSCG00000024517	AKAP6	850.55	1.28	0.07	17.77	0.00	0.00
ENSSSCG00000011087	SKIDA1	62.51	1.28	0.24	5.43	0.00	0.00
ENSSSCG00000039947	KCNJ2	242.50	1.28	0.14	9.41	0.00	0.00

ENSSSCG00000004093	IYD	809.52	1.28	0.08	16.93	0.00	0.00
ENSSSCG00000016207	RETREG2	3602.61	1.29	0.05	27.89	0.00	0.00
ENSSSCG00000006073	OSR2	1047.01	1.29	0.08	16.21	0.00	0.00
ENSSSCG00000002472		45.92	1.29	0.30	4.32	0.00	0.00
ENSSSCG00000007145	ADAM33	134.00	1.29	0.16	7.93	0.00	0.00
ENSSSCG00000021040	SLC26A11	333.16	1.29	0.11	11.97	0.00	0.00
ENSSSCG00000006791	OVGP1	19.56	1.29	0.42	3.06	0.00	0.00
ENSSSCG00000010444	LIPM	44.95	1.29	0.28	4.62	0.00	0.00
ENSSSCG00000008954		43.73	1.29	0.29	4.44	0.00	0.00
ENSSSCG00000006126	DECR1	1076.59	1.29	0.07	19.16	0.00	0.00
ENSSSCG00000015965	GPR155	325.70	1.29	0.11	11.32	0.00	0.00
ENSSSCG00000016611	CADPS2	279.37	1.29	0.12	11.04	0.00	0.00
ENSSSCG00000014055	CDHR2	461.66	1.29	0.10	13.38	0.00	0.00
ENSSSCG00000014213	KCNN2	210.44	1.29	0.14	9.52	0.00	0.00
ENSSSCG00000001762		69.18	1.29	0.25	5.25	0.00	0.00
ENSSSCG00000006995	ASAH1	3299.36	1.30	0.05	24.14	0.00	0.00
ENSSSCG00000023972	DRAM1	8.64	1.30	0.66	1.97	0.05	0.07
ENSSSCG00000013326	DCDC1	15.57	1.30	0.47	2.77	0.01	0.01
ENSSSCG00000040736	EPGN	62.43	1.30	0.25	5.21	0.00	0.00
ENSSSCG00000032660	TMEM35B	78.53	1.30	0.21	6.14	0.00	0.00
ENSSSCG00000015999	FKBP7	87.38	1.30	0.21	6.13	0.00	0.00
ENSSSCG00000028359	BST1	717.53	1.30	0.08	16.36	0.00	0.00
ENSSSCG00000024223	ARHGEF16	1160.05	1.30	0.07	19.30	0.00	0.00
ENSSSCG00000032648	RBL2	377.16	1.30	0.10	12.60	0.00	0.00
ENSSSCG00000008829	OCIAD2	122.15	1.30	0.19	6.92	0.00	0.00

ENSSSCG00000010654	ATRNL1	68.31	1.30	0.23	5.76	0.00	0.00
ENSSSCG00000031489	DUSP22	179.48	1.30	0.14	9.05	0.00	0.00
ENSSSCG00000010450	LIPA	1617.45	1.30	0.06	22.47	0.00	0.00
ENSSSCG00000015772	AGA	130.28	1.30	0.17	7.61	0.00	0.00
ENSSSCG00000008119	KCNIP3	373.19	1.30	0.10	12.78	0.00	0.00
ENSSSCG00000014900	RAB30	64.40	1.31	0.24	5.55	0.00	0.00
ENSSSCG00000021158	MCF2L	23.21	1.31	0.39	3.30	0.00	0.00
ENSSSCG00000031176	GP9	11.60	1.31	0.54	2.40	0.02	0.03
ENSSSCG00000040100	ITPKB	63.82	1.31	0.23	5.61	0.00	0.00
ENSSSCG00000039049	MAP7	3622.41	1.31	0.05	26.09	0.00	0.00
ENSSSCG00000023141	RNLS	27.28	1.31	0.39	3.39	0.00	0.00
ENSSSCG00000003763	IFI44	309.80	1.31	0.11	11.85	0.00	0.00
ENSSSCG00000004237	SERINC1	1380.57	1.31	0.07	19.23	0.00	0.00
ENSSSCG00000009889	ALDH2	1743.00	1.31	0.06	20.40	0.00	0.00
ENSSSCG00000002504	AK7	104.10	1.31	0.19	6.91	0.00	0.00
ENSSSCG00000000700	ACRBP	221.86	1.31	0.13	9.84	0.00	0.00
ENSSSCG00000006648	CTSS	161.74	1.31	0.15	8.66	0.00	0.00
ENSSSCG00000032673	ZFP14	30.19	1.31	0.34	3.81	0.00	0.00
ENSSSCG00000032327	TMEM169	12.20	1.31	0.53	2.45	0.01	0.02
ENSSSCG00000033338		42.48	1.31	0.30	4.35	0.00	0.00
ENSSSCG00000022963		344.01	1.31	0.11	12.05	0.00	0.00
ENSSSCG00000015175	VWA5A	143.97	1.31	0.17	7.82	0.00	0.00
ENSSSCG00000003760	TTLL7	332.67	1.31	0.11	11.92	0.00	0.00
ENSSSCG00000016969		30.33	1.31	0.36	3.70	0.00	0.00
ENSSSCG00000027252	HCFC1R1	153.74	1.32	0.16	8.22	0.00	0.00

ENSSSCG00000033100		9.86	1.32	0.62	2.13	0.03	0.05
ENSSSCG00000000139	IFT27	71.72	1.32	0.23	5.70	0.00	0.00
ENSSSCG00000035392	IGFBP2	4701.36	1.32	0.04	31.04	0.00	0.00
ENSSSCG00000025134	FAM171B	19.82	1.32	0.43	3.05	0.00	0.00
ENSSSCG00000037459	TCTA	279.40	1.32	0.12	11.23	0.00	0.00
ENSSSCG00000038505	MSI2	1169.24	1.32	0.07	20.16	0.00	0.00
ENSSSCG00000028192	DNAJC28	37.41	1.32	0.32	4.17	0.00	0.00
ENSSSCG00000015010	EXPH5	110.42	1.32	0.18	7.19	0.00	0.00
ENSSSCG00000012985		2126.60	1.32	0.05	25.29	0.00	0.00
ENSSSCG00000036248		265.62	1.32	0.12	10.91	0.00	0.00
ENSSSCG00000004535	TCF4	311.21	1.32	0.11	11.76	0.00	0.00
ENSSSCG00000002351		1600.66	1.32	0.07	20.34	0.00	0.00
ENSSSCG00000033462		26.89	1.32	0.37	3.56	0.00	0.00
ENSSSCG00000001612		16.40	1.32	0.46	2.89	0.00	0.01
ENSSSCG00000002385	TGFB3	9.95	1.32	0.59	2.26	0.02	0.04
ENSSSCG00000036091	MORC4	847.26	1.33	0.08	17.21	0.00	0.00
ENSSSCG00000004774	IVD	657.29	1.33	0.08	16.14	0.00	0.00
ENSSSCG00000009228	MAPK10	27.48	1.33	0.37	3.58	0.00	0.00
ENSSSCG00000029649	IFT46	525.83	1.33	0.09	14.48	0.00	0.00
ENSSSCG00000028905	TNIK	1874.65	1.33	0.06	22.86	0.00	0.00
ENSSSCG00000001986	NFATC4	16.98	1.33	0.46	2.90	0.00	0.01
ENSSSCG00000023924	PDE1A	938.06	1.33	0.07	18.80	0.00	0.00
ENSSSCG00000035933	MAPK15	204.42	1.33	0.14	9.58	0.00	0.00
ENSSSCG00000008574	KIF3C	468.90	1.33	0.09	14.19	0.00	0.00
ENSSSCG00000039269	SHLD1	28.29	1.33	0.36	3.73	0.00	0.00

ENSSSCG00000027013		233.01	1.33	0.13	10.61	0.00	0.00
ENSSSCG00000039425	BPGM	3757.66	1.34	0.05	25.32	0.00	0.00
ENSSSCG00000027855	SOCS1	18.82	1.34	0.45	2.99	0.00	0.01
ENSSSCG00000033829	RIMS2	403.42	1.34	0.10	13.55	0.00	0.00
ENSSSCG00000021527	ATP10B	23.57	1.34	0.39	3.46	0.00	0.00
ENSSSCG00000016158	LANCL1	748.55	1.34	0.08	17.19	0.00	0.00
ENSSSCG00000036229		283.97	1.34	0.12	11.58	0.00	0.00
ENSSSCG00000014156	ARRDC3	3273.46	1.34	0.05	27.76	0.00	0.00
ENSSSCG00000023080	TPP1	6319.98	1.34	0.06	23.57	0.00	0.00
ENSSSCG00000038141		121.34	1.34	0.18	7.56	0.00	0.00
ENSSSCG00000011600	SLC6A6	3548.27	1.34	0.05	27.75	0.00	0.00
ENSSSCG00000008686	C4orf48	25.35	1.34	0.38	3.55	0.00	0.00
ENSSSCG00000015906	CSRNP3	29.54	1.34	0.34	3.90	0.00	0.00
ENSSSCG00000000133	TST	753.26	1.34	0.08	16.00	0.00	0.00
ENSSSCG00000023975	CCDC9B	512.43	1.34	0.09	15.05	0.00	0.00
ENSSSCG00000016205	NHEJ1	964.26	1.35	0.07	19.59	0.00	0.00
ENSSSCG00000029267	TIGIT	16.62	1.35	0.46	2.93	0.00	0.01
ENSSSCG00000012825	IKBKG	1286.09	1.35	0.07	18.38	0.00	0.00
ENSSSCG00000008534	TOGARAM2	45.68	1.35	0.28	4.78	0.00	0.00
ENSSSCG00000000082	CACNA1I	11.23	1.35	0.56	2.40	0.02	0.03
ENSSSCG00000010402	ZFAND4	98.60	1.35	0.20	6.75	0.00	0.00
ENSSSCG00000031703		123.32	1.35	0.18	7.54	0.00	0.00
ENSSSCG00000008799	LIMCH1	1325.52	1.35	0.07	19.21	0.00	0.00
ENSSSCG00000036411	ACOT13	152.80	1.35	0.16	8.72	0.00	0.00
ENSSSCG00000009373	SLC25A15	1222.25	1.36	0.07	20.39	0.00	0.00

ENSSSCG00000011892		60.09	1.36	0.25	5.47	0.00	0.00
ENSSSCG00000026996		3739.36	1.36	0.05	25.54	0.00	0.00
ENSSSCG00000013879	SLC27A1	22.04	1.36	0.40	3.40	0.00	0.00
ENSSSCG00000037854	L3HYPDH	144.78	1.36	0.16	8.52	0.00	0.00
ENSSSCG00000035762		1703.47	1.36	0.06	21.88	0.00	0.00
ENSSSCG00000031023		29526.85	1.36	0.05	26.12	0.00	0.00
ENSSSCG00000016093		8.95	1.36	0.66	2.07	0.04	0.06
ENSSSCG00000008486	GALM	1075.28	1.37	0.07	19.16	0.00	0.00
ENSSSCG00000038160	CNGA1	7.76	1.37	0.68	2.02	0.04	0.07
ENSSSCG00000008020	IFT140	218.30	1.37	0.13	10.21	0.00	0.00
ENSSSCG00000036804	TCEA2	101.06	1.37	0.19	7.02	0.00	0.00
ENSSSCG00000033830	CCDC8	92.05	1.37	0.20	6.93	0.00	0.00
ENSSSCG00000030016	PDE9A	30.45	1.37	0.35	3.91	0.00	0.00
ENSSSCG00000000735	TSPAN9	141.94	1.37	0.17	8.28	0.00	0.00
ENSSSCG00000000292	ZNF385A	281.40	1.37	0.13	10.58	0.00	0.00
ENSSSCG00000016842	NIPBL	4139.70	1.37	0.05	25.46	0.00	0.00
ENSSSCG00000028144	EPHX3	10.77	1.37	0.57	2.41	0.02	0.03
ENSSSCG00000024837	SYT12	18.56	1.37	0.43	3.18	0.00	0.00
ENSSSCG00000014920	FZD4	1864.03	1.37	0.06	24.82	0.00	0.00
ENSSSCG00000031199		22.18	1.37	0.40	3.42	0.00	0.00
ENSSSCG00000034418	CREBL2	272.72	1.37	0.12	11.09	0.00	0.00
ENSSSCG00000004586	FAM81A	1249.19	1.37	0.07	19.65	0.00	0.00
ENSSSCG00000023653	GLIS2	770.12	1.37	0.08	17.65	0.00	0.00
ENSSSCG00000032231		30.07	1.38	0.34	4.00	0.00	0.00
ENSSSCG00000029199	SCN4B	31.30	1.38	0.34	4.11	0.00	0.00

ENSSSCG00000032946		451.23	1.38	0.10	13.45	0.00	0.00
ENSSSCG00000012975	SNX32	45.82	1.38	0.28	4.96	0.00	0.00
ENSSSCG00000017137	METRNL	38.55	1.38	0.31	4.44	0.00	0.00
ENSSSCG00000008738	CC2D2A	188.66	1.38	0.14	9.66	0.00	0.00
ENSSSCG00000031531	TSPAN10	37.96	1.38	0.31	4.50	0.00	0.00
ENSSSCG00000008836		63.91	1.38	0.23	5.90	0.00	0.00
ENSSSCG00000037450	SSC5D	166.94	1.38	0.15	9.27	0.00	0.00
ENSSSCG00000007930	MGRN1	616.32	1.38	0.08	16.46	0.00	0.00
ENSSSCG00000002287	MPP5	1573.59	1.39	0.06	21.66	0.00	0.00
ENSSSCG00000027114	SCP2	604.83	1.39	0.09	16.12	0.00	0.00
ENSSSCG00000005110	SYNE2	4328.55	1.39	0.05	30.27	0.00	0.00
ENSSSCG00000005627	AK1	353.40	1.39	0.11	12.48	0.00	0.00
ENSSSCG00000009215	ABCG2	198.51	1.39	0.15	9.18	0.00	0.00
ENSSSCG00000032428	ARSA	182.44	1.39	0.15	9.22	0.00	0.00
ENSSSCG00000007072	SPTLC3	2520.24	1.39	0.05	26.28	0.00	0.00
ENSSSCG00000003839		4511.66	1.39	0.06	24.68	0.00	0.00
ENSSSCG00000015342	COL28A1	50.76	1.39	0.27	5.18	0.00	0.00
ENSSSCG00000005361	ALDH1B1	271.64	1.39	0.12	11.48	0.00	0.00
ENSSSCG00000036206	DIPK2A	165.40	1.39	0.15	9.00	0.00	0.00
ENSSSCG00000009169	SLC39A8	564.45	1.39	0.10	14.54	0.00	0.00
ENSSSCG00000037491		13.26	1.39	0.65	2.14	0.03	0.05
ENSSSCG00000017506	FBXL20	157.62	1.40	0.15	9.21	0.00	0.00
ENSSSCG00000023956	NEDD9	2178.61	1.40	0.06	22.03	0.00	0.00
ENSSSCG00000039860		138.81	1.40	0.19	7.20	0.00	0.00
ENSSSCG00000009046		7.28	1.40	0.70	2.01	0.04	0.07

ENSSSCG00000014988	MMP13	73.44	1.40	0.22	6.24	0.00	0.00
ENSSSCG00000037953	NDUFC1	287.95	1.40	0.12	11.69	0.00	0.00
ENSSSCG00000032160		77.75	1.40	0.22	6.45	0.00	0.00
ENSSSCG00000024389	SIGIRR	144.04	1.40	0.17	8.16	0.00	0.00
ENSSSCG00000015266	FMO4	45.70	1.40	0.29	4.82	0.00	0.00
ENSSSCG00000028827	PARVA	1794.57	1.40	0.06	24.38	0.00	0.00
ENSSSCG00000005529	MORN5	82.11	1.40	0.22	6.50	0.00	0.00
ENSSSCG00000037898	CXXC5	525.05	1.40	0.09	15.01	0.00	0.00
ENSSSCG00000008554	ABHD1	24.37	1.40	0.38	3.65	0.00	0.00
ENSSSCG00000011030	CUBN	254.68	1.41	0.13	11.18	0.00	0.00
ENSSSCG00000008122	ADRA2B	15.25	1.41	0.48	2.91	0.00	0.01
ENSSSCG00000011477	ACOX2	569.01	1.41	0.09	16.03	0.00	0.00
ENSSSCG00000040282		7.32	1.41	0.70	2.01	0.04	0.07
ENSSSCG00000007157	SLC4A11	42.06	1.41	0.30	4.77	0.00	0.00
ENSSSCG00000013270	CRY2	468.51	1.41	0.09	14.99	0.00	0.00
ENSSSCG00000001664	TTBK1	17.11	1.41	0.46	3.08	0.00	0.00
ENSSSCG00000022128	MXI1	105.69	1.41	0.20	7.13	0.00	0.00
ENSSSCG00000011877	CD86	221.18	1.41	0.14	9.81	0.00	0.00
ENSSSCG00000009927	FAM222A	25.07	1.41	0.39	3.66	0.00	0.00
ENSSSCG00000005740	SARDH	53.67	1.41	0.27	5.30	0.00	0.00
ENSSSCG00000040956		17.74	1.41	0.45	3.15	0.00	0.00
ENSSSCG00000000209	NCKAP5L	101.52	1.41	0.19	7.45	0.00	0.00
ENSSSCG00000004813	TTC23	60.58	1.41	0.26	5.53	0.00	0.00
ENSSSCG00000031830		144.44	1.41	0.16	8.80	0.00	0.00
ENSSSCG00000000449	DTX3	109.03	1.41	0.18	7.67	0.00	0.00

ENSSSCG00000010184	AGT	326.74	1.41	0.11	12.50	0.00	0.00
ENSSSCG00000029029	ZNF713	28.75	1.42	0.35	4.04	0.00	0.00
ENSSSCG00000013497	ANKRD24	28.75	1.42	0.35	4.03	0.00	0.00
ENSSSCG00000000260	SOAT2	91.95	1.42	0.20	7.00	0.00	0.00
ENSSSCG00000039695		45.27	1.42	0.29	4.97	0.00	0.00
ENSSSCG00000023273	SH3YL1	93.75	1.42	0.20	7.12	0.00	0.00
ENSSSCG00000040562	C8orf88	10.44	1.42	0.58	2.43	0.02	0.02
ENSSSCG00000025788	ENPP4	941.42	1.42	0.07	19.89	0.00	0.00
ENSSSCG00000017497	ERBB2	551.08	1.42	0.09	15.59	0.00	0.00
ENSSSCG00000013625	CCDC151	205.19	1.42	0.13	10.56	0.00	0.00
ENSSSCG00000011437	ALAS1	2917.46	1.42	0.05	29.44	0.00	0.00
ENSSSCG00000015557	NMNAT2	225.19	1.42	0.13	10.59	0.00	0.00
ENSSSCG00000033669	RDH10	824.71	1.43	0.07	19.08	0.00	0.00
ENSSSCG00000011263	TTC21A	109.09	1.43	0.18	7.85	0.00	0.00
ENSSSCG00000017200	UNC13D	19.77	1.43	0.43	3.31	0.00	0.00
ENSSSCG00000009114	PRSS12	186.19	1.43	0.14	10.12	0.00	0.00
ENSSSCG00000036961	PCDHB7	232.97	1.43	0.13	11.06	0.00	0.00
ENSSSCG00000016877	NNT	3493.72	1.44	0.05	28.70	0.00	0.00
ENSSSCG00000016206	CNPPD1	1559.40	1.44	0.07	21.02	0.00	0.00
ENSSSCG00000017921	ZMYND15	16.76	1.44	0.46	3.14	0.00	0.00
ENSSSCG00000015781	ENPP6	4627.97	1.44	0.05	31.30	0.00	0.00
ENSSSCG00000023026	CLCN5	4994.16	1.44	0.05	31.88	0.00	0.00
ENSSSCG00000009457	DACH1	1243.15	1.44	0.07	21.42	0.00	0.00
ENSSSCG00000037224	DEXI	38.49	1.44	0.31	4.64	0.00	0.00
ENSSSCG00000032740	TMEM106B	1454.34	1.44	0.07	21.36	0.00	0.00

ENSSSCG00000009069	C4orf33	55.92	1.44	0.26	5.59	0.00	0.00
ENSSSCG00000037994		59.13	1.45	0.26	5.55	0.00	0.00
ENSSSCG00000032949		247.56	1.45	0.12	11.58	0.00	0.00
ENSSSCG00000005837	DIPK1B	129.05	1.45	0.17	8.37	0.00	0.00
ENSSSCG00000003722	CDH2	217.42	1.45	0.14	10.34	0.00	0.00
ENSSSCG00000003266	TMC4	11.23	1.45	0.58	2.51	0.01	0.02
ENSSSCG00000010723	ACADSB	2215.82	1.45	0.06	24.82	0.00	0.00
ENSSSCG00000026592	TLR6	37.49	1.45	0.31	4.71	0.00	0.00
ENSSSCG00000029281	PCDHAC2	473.35	1.45	0.09	15.75	0.00	0.00
ENSSSCG00000013767	PALM3	31.88	1.45	0.34	4.31	0.00	0.00
ENSSSCG00000003965		250.42	1.45	0.12	11.85	0.00	0.00
ENSSSCG00000013377	USH1C	461.94	1.45	0.10	15.07	0.00	0.00
ENSSSCG00000035478	RMND5A	692.88	1.46	0.08	17.46	0.00	0.00
ENSSSCG00000033348	ISL2	16.91	1.46	0.46	3.17	0.00	0.00
ENSSSCG00000032814	DNAH7	84.62	1.46	0.21	6.91	0.00	0.00
ENSSSCG00000027669	TNS1	537.83	1.46	0.09	16.25	0.00	0.00
ENSSSCG00000029860	EPB41L4A	2083.39	1.46	0.06	23.39	0.00	0.00
ENSSSCG00000012283	ZNF81	49.67	1.46	0.28	5.23	0.00	0.00
ENSSSCG00000016200	PRKAG3	204.56	1.46	0.14	10.67	0.00	0.00
ENSSSCG00000037262	PTX4	131.20	1.46	0.17	8.69	0.00	0.00
ENSSSCG00000023890	ATG2A	824.39	1.46	0.08	18.87	0.00	0.00
ENSSSCG00000017635	MKS1	380.99	1.46	0.11	13.87	0.00	0.00
ENSSSCG00000008491	QPCT	660.96	1.46	0.08	17.95	0.00	0.00
ENSSSCG00000003777	SLC44A5	173.36	1.47	0.15	9.93	0.00	0.00
ENSSSCG00000024810	PRAM1	20.74	1.47	0.43	3.42	0.00	0.00

ENSSSCG00000008340	ANTXR1	261.08	1.47	0.12	11.79	0.00	0.00
ENSSSCG00000037395	CLIC3	15.70	1.47	0.47	3.09	0.00	0.00
ENSSSCG00000033392	SCML4	249.03	1.47	0.12	11.91	0.00	0.00
ENSSSCG00000031261	RHOQ	190.64	1.47	0.14	10.13	0.00	0.00
ENSSSCG00000003256	PRKCG	90.11	1.47	0.20	7.29	0.00	0.00
ENSSSCG00000002014	JPH4	6.91	1.47	0.73	2.01	0.04	0.07
ENSSSCG00000013894	IL12RB1	27.79	1.47	0.37	3.95	0.00	0.00
ENSSSCG00000003651	RHBDL2	96.49	1.47	0.19	7.55	0.00	0.00
ENSSSCG00000016943	ADAMTS6	118.57	1.47	0.18	8.34	0.00	0.00
ENSSSCG00000007056	PLCB1	3051.72	1.47	0.05	30.82	0.00	0.00
ENSSSCG00000038649		13.85	1.47	0.52	2.85	0.00	0.01
ENSSSCG00000008913	IGFBP7	2522.28	1.47	0.06	26.34	0.00	0.00
ENSSSCG00000004510	ACAA2	2071.54	1.47	0.06	24.79	0.00	0.00
ENSSSCG00000010651	ABLIM1	1205.34	1.48	0.07	21.54	0.00	0.00
ENSSSCG00000022168	APBB2	3604.60	1.48	0.06	24.83	0.00	0.00
ENSSSCG00000028822	UCN3	22.07	1.48	0.41	3.60	0.00	0.00
ENSSSCG00000031940	GAS2	35.44	1.48	0.32	4.67	0.00	0.00
ENSSSCG00000040267	CYS1	722.07	1.48	0.08	18.56	0.00	0.00
ENSSSCG00000008765		530.90	1.48	0.09	16.67	0.00	0.00
ENSSSCG00000035061	WBP1	282.53	1.48	0.12	12.24	0.00	0.00
ENSSSCG00000023074	KLK4	119.21	1.48	0.18	8.47	0.00	0.00
ENSSSCG00000032200		19.01	1.48	0.43	3.43	0.00	0.00
ENSSSCG00000021466	BMT2	242.79	1.48	0.13	11.24	0.00	0.00
ENSSSCG00000038125	NUP210L	107.25	1.48	0.19	7.88	0.00	0.00
ENSSSCG00000035071	DCST1	10.76	1.48	0.58	2.55	0.01	0.02

ENSSSCG00000014909		3944.55	1.48	0.05	28.34	0.00	0.00
ENSSSCG00000014880	AQP11	307.60	1.49	0.12	12.80	0.00	0.00
ENSSSCG00000022305	WDR78	12.69	1.49	0.53	2.79	0.01	0.01
ENSSSCG00000032153	C19orf12	146.81	1.49	0.16	9.32	0.00	0.00
ENSSSCG00000003715	ZNF521	243.63	1.49	0.13	11.12	0.00	0.00
ENSSSCG00000004125	STX11	83.35	1.49	0.21	7.14	0.00	0.00
ENSSSCG00000015144	GRAMD1B	1913.68	1.49	0.06	25.04	0.00	0.00
ENSSSCG00000008029	BAIAP3	35.04	1.49	0.32	4.59	0.00	0.00
ENSSSCG00000015084		170.64	1.49	0.16	9.42	0.00	0.00
ENSSSCG00000028382		36.35	1.49	0.33	4.48	0.00	0.00
ENSSSCG00000026471	PCDHA1	17.19	1.50	0.47	3.21	0.00	0.00
ENSSSCG00000000798	TWF1	10715.45	1.50	0.05	30.99	0.00	0.00
ENSSSCG00000012295	MAGIX	54.33	1.50	0.27	5.58	0.00	0.00
ENSSSCG00000007797	ITGAL	171.16	1.50	0.15	10.01	0.00	0.00
ENSSSCG00000001659	KLC4	588.98	1.50	0.09	16.84	0.00	0.00
ENSSSCG00000002400		234.25	1.50	0.13	11.73	0.00	0.00
ENSSSCG00000015197	SPA17	80.82	1.51	0.22	6.83	0.00	0.00
ENSSSCG00000003543	NIPAL3	496.14	1.51	0.10	15.83	0.00	0.00
ENSSSCG00000016542	LRGUK	25.01	1.51	0.38	3.98	0.00	0.00
ENSSSCG00000014975		19.95	1.51	0.43	3.48	0.00	0.00
ENSSSCG00000007816	IL21R	14.16	1.51	0.51	2.98	0.00	0.01
ENSSSCG00000035371		15.42	1.51	0.48	3.14	0.00	0.00
ENSSSCG00000027467	DZIP1L	500.39	1.51	0.09	16.33	0.00	0.00
ENSSSCG00000002640	DEF8	763.82	1.51	0.08	18.54	0.00	0.00
ENSSSCG00000033467		17.35	1.51	0.45	3.32	0.00	0.00

ENSSSCG00000003917	TESK2	16.71	1.51	0.46	3.25	0.00	0.00
ENSSSCG00000040513	AQP3	16.06	1.51	0.48	3.15	0.00	0.00
ENSSSCG00000032687	CYP4V2	244.21	1.51	0.13	11.34	0.00	0.00
ENSSSCG00000029219	SH3BGR	11.58	1.51	0.56	2.72	0.01	0.01
ENSSSCG00000031957		351.14	1.51	0.11	13.82	0.00	0.00
ENSSSCG00000008980	SCARB2	1326.49	1.51	0.07	22.40	0.00	0.00
ENSSSCG00000016625	CTTNBP2	1878.15	1.51	0.06	26.10	0.00	0.00
ENSSSCG00000026991	LURAP1	12.22	1.52	0.55	2.77	0.01	0.01
ENSSSCG00000021041		264.53	1.52	0.14	11.03	0.00	0.00
ENSSSCG00000016606	ASB15	70.38	1.52	0.23	6.60	0.00	0.00
ENSSSCG00000006720	HAO2	38.12	1.52	0.31	4.91	0.00	0.00
ENSSSCG00000031759	TM6SF2	6.47	1.52	0.75	2.02	0.04	0.07
ENSSSCG00000028185	FGD3	287.61	1.52	0.12	12.92	0.00	0.00
ENSSSCG00000014048	HK3	44.00	1.52	0.30	5.06	0.00	0.00
ENSSSCG00000002425		1064.74	1.52	0.07	21.17	0.00	0.00
ENSSSCG00000014039	RGS14	361.00	1.52	0.11	14.41	0.00	0.00
ENSSSCG00000009542	TNFSF13B	14.88	1.52	0.49	3.11	0.00	0.00
ENSSSCG00000024257	CEMIP2	480.57	1.52	0.09	16.45	0.00	0.00
ENSSSCG00000005484	ZNF618	130.23	1.52	0.17	8.87	0.00	0.00
ENSSSCG00000014217	FEM1C	2062.39	1.52	0.06	26.90	0.00	0.00
ENSSSCG00000000021		65.39	1.53	0.24	6.34	0.00	0.00
ENSSSCG00000026317	SLC10A2	14.28	1.53	0.50	3.05	0.00	0.00
ENSSSCG00000013585	EVI5L	78.98	1.53	0.23	6.67	0.00	0.00
ENSSSCG00000036499	LMO3	38.28	1.53	0.31	5.00	0.00	0.00
ENSSSCG00000010640	NRAP	57.70	1.53	0.26	5.98	0.00	0.00

ENSSSCG00000007313	CNBD2	22.73	1.53	0.40	3.81	0.00	0.00
ENSSSCG00000019941	ssc-mir-30a	7.78	1.53	0.69	2.23	0.03	0.04
ENSSSCG00000004384	NR2E1	19.47	1.53	0.43	3.56	0.00	0.00
ENSSSCG00000000198	DNAJC22	485.66	1.53	0.09	16.49	0.00	0.00
ENSSSCG00000036516		216.43	1.53	0.13	11.61	0.00	0.00
ENSSSCG00000012634	DOCK11	29.31	1.53	0.37	4.18	0.00	0.00
ENSSSCG00000038597	KLHDC9	58.50	1.53	0.26	5.85	0.00	0.00
ENSSSCG00000009197	GRID2	31.88	1.53	0.34	4.46	0.00	0.00
ENSSSCG00000038825		61.15	1.54	0.26	6.01	0.00	0.00
ENSSSCG00000013074	RAB3IL1	57.29	1.54	0.25	6.10	0.00	0.00
ENSSSCG00000004787	GPR176	46.28	1.54	0.28	5.49	0.00	0.00
ENSSSCG00000028004	RIN2	426.93	1.54	0.10	15.50	0.00	0.00
ENSSSCG00000033766		81.51	1.54	0.21	7.18	0.00	0.00
ENSSSCG00000005182	CCDC171	22.15	1.54	0.40	3.81	0.00	0.00
ENSSSCG00000040205	TLCD2	47.00	1.54	0.29	5.38	0.00	0.00
ENSSSCG00000008302	FBXO41	33.39	1.54	0.34	4.57	0.00	0.00
ENSSSCG00000003690	DLGAP1	665.58	1.54	0.09	17.80	0.00	0.00
ENSSSCG00000013461	PEAK3	57.52	1.54	0.25	6.10	0.00	0.00
ENSSSCG00000011755	NCEH1	569.32	1.55	0.09	16.85	0.00	0.00
ENSSSCG00000029571	AVIL	41.32	1.55	0.30	5.22	0.00	0.00
ENSSSCG00000005659	ZER1	259.01	1.55	0.12	12.68	0.00	0.00
ENSSSCG00000011076	OTUD1	136.64	1.55	0.18	8.68	0.00	0.00
ENSSSCG00000038190	MPST	103.87	1.55	0.20	7.80	0.00	0.00
ENSSSCG00000032637	EVC	636.99	1.55	0.09	17.80	0.00	0.00
ENSSSCG00000003472	ARHGEF19	27.58	1.55	0.38	4.05	0.00	0.00

ENSSSCG00000015795		104.62	1.55	0.19	8.21	0.00	0.00
ENSSSCG00000005916		60.42	1.55	0.26	6.06	0.00	0.00
ENSSSCG00000011559	IL17RC	82.81	1.56	0.22	7.14	0.00	0.00
ENSSSCG00000003429	CLCN6	543.36	1.56	0.09	17.67	0.00	0.00
ENSSSCG00000010277	SLC29A3	303.81	1.56	0.12	12.91	0.00	0.00
ENSSSCG00000008771	C4orf19	381.23	1.56	0.10	15.25	0.00	0.00
ENSSSCG00000013320	PAX6	23.12	1.56	0.40	3.92	0.00	0.00
ENSSSCG00000030345	INPP5J	31.68	1.56	0.34	4.62	0.00	0.00
ENSSSCG00000011106	CREM	347.72	1.56	0.11	13.91	0.00	0.00
ENSSSCG00000020706		2423.52	1.56	0.06	27.26	0.00	0.00
ENSSSCG00000038772	IKBIP	101.13	1.56	0.20	7.95	0.00	0.00
ENSSSCG00000012309	SHROOM4	1430.17	1.57	0.06	24.18	0.00	0.00
ENSSSCG00000016159	CPS1	227.09	1.57	0.13	11.98	0.00	0.00
ENSSSCG00000010298	CFAP70	6.62	1.57	0.74	2.12	0.03	0.05
ENSSSCG00000006069	RGS22	25.14	1.57	0.38	4.14	0.00	0.00
ENSSSCG00000003228	VSIG10L	7.30	1.57	0.74	2.12	0.03	0.05
ENSSSCG00000029347		202.29	1.57	0.14	11.44	0.00	0.00
ENSSSCG00000027091	OAZ3	15.26	1.57	0.48	3.25	0.00	0.00
ENSSSCG00000023710	REEP1	11.94	1.57	0.55	2.84	0.00	0.01
ENSSSCG00000039651	SLC2A5	18.66	1.57	0.47	3.35	0.00	0.00
ENSSSCG00000017904	ENO3	40.54	1.57	0.31	5.11	0.00	0.00
ENSSSCG00000008742	CD38	16.65	1.57	0.47	3.34	0.00	0.00
ENSSSCG00000009293		673.43	1.58	0.08	18.74	0.00	0.00
ENSSSCG00000030585	HOXC6	13.98	1.58	0.52	3.03	0.00	0.00
ENSSSCG00000006082	MATN2	34.55	1.58	0.35	4.53	0.00	0.00

ENSSSCG00000000695	IFFO1	86.52	1.58	0.21	7.54	0.00	0.00
ENSSSCG00000010581	PSD	21.96	1.58	0.41	3.84	0.00	0.00
ENSSSCG00000016033	GULP1	98.65	1.58	0.21	7.56	0.00	0.00
ENSSSCG00000000455	LRIG3	470.12	1.58	0.09	16.97	0.00	0.00
ENSSSCG00000004875	CYB5A	1757.25	1.58	0.07	24.19	0.00	0.00
ENSSSCG00000033790		8.04	1.58	0.70	2.28	0.02	0.04
ENSSSCG00000016005	SESTD1	890.85	1.58	0.08	20.01	0.00	0.00
ENSSSCG00000025306	PAK3	185.66	1.58	0.14	11.08	0.00	0.00
ENSSSCG00000016976	ZNF366	17.38	1.59	0.45	3.49	0.00	0.00
ENSSSCG00000002866	CEBPA	11.32	1.59	0.59	2.68	0.01	0.01
ENSSSCG00000031594		275.59	1.59	0.12	13.28	0.00	0.00
ENSSSCG00000015491		276.29	1.59	0.12	12.72	0.00	0.00
ENSSSCG00000006479		14.73	1.59	0.50	3.16	0.00	0.00
ENSSSCG00000014908	CCDC89	12.69	1.59	0.53	2.97	0.00	0.01
ENSSSCG00000024485	RUNDC3B	17.48	1.59	0.47	3.41	0.00	0.00
ENSSSCG00000021208	SELENOP	14025.72	1.59	0.06	28.29	0.00	0.00
ENSSSCG00000009875	CFAP73	31.58	1.59	0.35	4.58	0.00	0.00
ENSSSCG00000011496	ADAMTS9	945.92	1.60	0.07	22.20	0.00	0.00
ENSSSCG00000038038	SBK1	10.78	1.60	0.59	2.70	0.01	0.01
ENSSSCG00000039998	CLIC2	32.31	1.60	0.34	4.68	0.00	0.00
ENSSSCG00000017952	SAT2	238.61	1.60	0.13	12.35	0.00	0.00
ENSSSCG00000006052	FZD6	7690.41	1.60	0.04	38.76	0.00	0.00
ENSSSCG00000008689	ZFYVE28	49.17	1.60	0.30	5.26	0.00	0.00
ENSSSCG00000028108	ASAP3	32.43	1.60	0.34	4.68	0.00	0.00
ENSSSCG00000004971	TLE3	1710.65	1.60	0.06	27.43	0.00	0.00

ENSSSCG00000009778	RILPL2	300.02	1.60	0.13	12.58	0.00	0.00
ENSSSCG00000006267	PCMTD1	1816.28	1.61	0.06	26.99	0.00	0.00
ENSSSCG00000003374	ESPN	25.05	1.61	0.38	4.19	0.00	0.00
ENSSSCG00000035372	PSCA	72.32	1.61	0.23	7.05	0.00	0.00
ENSSSCG00000035934	TXNDC16	86.30	1.62	0.21	7.76	0.00	0.00
ENSSSCG00000037270	TCTEX1D4	23.73	1.62	0.40	4.03	0.00	0.00
ENSSSCG00000030938		137.99	1.62	0.17	9.48	0.00	0.00
ENSSSCG00000009151	CYP2U1	168.08	1.62	0.16	10.41	0.00	0.00
ENSSSCG00000015607	HHAT	130.83	1.62	0.17	9.57	0.00	0.00
ENSSSCG00000006878	DPYD	527.99	1.63	0.10	17.10	0.00	0.00
ENSSSCG00000023585	SERINC2	376.23	1.63	0.11	14.67	0.00	0.00
ENSSSCG00000007864	GPRC5B	4367.99	1.63	0.05	33.72	0.00	0.00
ENSSSCG00000007435		168.25	1.63	0.15	10.82	0.00	0.00
ENSSSCG00000001755	HYKK	475.47	1.63	0.10	17.04	0.00	0.00
ENSSSCG00000031450	MLYCD	70.60	1.63	0.24	6.84	0.00	0.00
ENSSSCG00000037808		103.61	1.63	0.20	8.30	0.00	0.00
ENSSSCG00000031452	C14orf93	11.69	1.64	0.58	2.81	0.00	0.01
ENSSSCG00000008488		43.92	1.64	0.30	5.50	0.00	0.00
ENSSSCG00000023520	PCSK5	475.97	1.64	0.10	16.73	0.00	0.00
ENSSSCG00000010664	ENO4	105.97	1.64	0.19	8.61	0.00	0.00
ENSSSCG00000015802	FAM149A	1119.10	1.64	0.07	24.43	0.00	0.00
ENSSSCG00000004012	THBS2	18.65	1.64	0.46	3.57	0.00	0.00
ENSSSCG00000007901	CIITA	54.37	1.64	0.28	5.86	0.00	0.00
ENSSSCG00000013490	PIP5K1C	857.28	1.64	0.08	20.67	0.00	0.00
ENSSSCG00000030696	SLC19A1	206.39	1.65	0.14	11.62	0.00	0.00

ENSSSCG00000031384	PCDHGA2	7.58	1.65	0.71	2.33	0.02	0.03
ENSSSCG00000024633	JAKMIP3	7.59	1.65	0.71	2.33	0.02	0.03
ENSSSCG00000013568		992.17	1.65	0.07	23.41	0.00	0.00
ENSSSCG00000013335	LGR4	2613.83	1.65	0.05	32.66	0.00	0.00
ENSSSCG00000024341	USP20	113.87	1.66	0.19	8.83	0.00	0.00
ENSSSCG00000032098		15.99	1.66	0.49	3.39	0.00	0.00
ENSSSCG00000009852	VSIG10	300.44	1.66	0.12	14.36	0.00	0.00
ENSSSCG00000006587	PGLYRP4	18.04	1.66	0.45	3.69	0.00	0.00
ENSSSCG00000012585	DCX	110.47	1.66	0.19	8.70	0.00	0.00
ENSSSCG00000039433		7.00	1.66	0.77	2.16	0.03	0.05
ENSSSCG00000015331	PON2	427.62	1.66	0.10	16.27	0.00	0.00
ENSSSCG00000039327	DAPP1	79.46	1.66	0.22	7.44	0.00	0.00
ENSSSCG00000001422	C2	9.76	1.67	0.61	2.71	0.01	0.01
ENSSSCG00000013145	DTX4	443.99	1.67	0.11	15.80	0.00	0.00
ENSSSCG00000031255		16.72	1.67	0.47	3.52	0.00	0.00
ENSSSCG00000036356	ZSCAN2	130.59	1.67	0.17	9.81	0.00	0.00
ENSSSCG00000003455	FHAD1	886.53	1.67	0.07	22.94	0.00	0.00
ENSSSCG00000033997		6.30	1.67	0.76	2.20	0.03	0.04
ENSSSCG00000013569	PEX11G	37.76	1.67	0.32	5.29	0.00	0.00
ENSSSCG00000001629	USP49	18.20	1.68	0.45	3.72	0.00	0.00
ENSSSCG00000004752	EXD1	25.95	1.68	0.38	4.39	0.00	0.00
ENSSSCG00000006717	PHGDH	4146.30	1.68	0.05	34.48	0.00	0.00
ENSSSCG00000006053	BAALC	194.92	1.68	0.14	11.89	0.00	0.00
ENSSSCG00000005232	SMARCA2	1693.16	1.68	0.06	28.73	0.00	0.00
ENSSSCG00000029442		14.08	1.68	0.51	3.27	0.00	0.00

ENSSSCG00000037566		108.38	1.68	0.19	8.80	0.00	0.00
ENSSSCG00000007286	ACSS2	526.67	1.68	0.09	18.78	0.00	0.00
ENSSSCG00000031648	CCN5	52.82	1.69	0.27	6.35	0.00	0.00
ENSSSCG00000007814	KIAA0556	83.13	1.69	0.21	7.90	0.00	0.00
ENSSSCG00000030513	CFAP74	163.67	1.69	0.16	10.63	0.00	0.00
ENSSSCG00000021867	HPN	67.80	1.69	0.24	6.90	0.00	0.00
ENSSSCG00000026605	BPI	5.66	1.69	0.81	2.10	0.04	0.06
ENSSSCG00000001715	ENPP5	809.75	1.69	0.08	22.46	0.00	0.00
ENSSSCG00000015540	MR1	24.11	1.70	0.40	4.26	0.00	0.00
ENSSSCG00000030544	TMEM143	148.02	1.70	0.17	9.95	0.00	0.00
ENSSSCG00000032536	B3GNT8	7.81	1.70	0.69	2.48	0.01	0.02
ENSSSCG00000037997		12.76	1.70	0.54	3.15	0.00	0.00
ENSSSCG00000004180		10.65	1.70	0.59	2.88	0.00	0.01
ENSSSCG00000007451	SLC2A10	58.78	1.70	0.26	6.58	0.00	0.00
ENSSSCG00000010106		4.97	1.70	0.85	1.99	0.05	0.07
ENSSSCG00000010532	LOXL4	475.87	1.70	0.10	17.14	0.00	0.00
ENSSSCG00000022310	VPS9D1	178.41	1.70	0.15	11.06	0.00	0.00
ENSSSCG00000031518	STARD4	980.88	1.70	0.09	19.95	0.00	0.00
ENSSSCG00000037015	SESN3	757.90	1.70	0.08	20.50	0.00	0.00
ENSSSCG00000011504	EOGT	654.58	1.70	0.08	20.18	0.00	0.00
ENSSSCG00000008456	PLEKHH2	266.94	1.71	0.13	13.34	0.00	0.00
ENSSSCG00000005512	C5	15.68	1.71	0.50	3.43	0.00	0.00
ENSSSCG00000006080	ERICH5	351.40	1.71	0.11	15.86	0.00	0.00
ENSSSCG00000039395	GAS2L2	8.55	1.71	0.66	2.58	0.01	0.02
ENSSSCG00000004861	TSHZ1	181.93	1.72	0.15	11.25	0.00	0.00

ENSSSCG00000017446		37.22	1.72	0.33	5.19	0.00	0.00
ENSSSCG00000016420	INSIG1	1244.42	1.72	0.14	12.65	0.00	0.00
ENSSSCG00000032262	IDUA	314.73	1.72	0.12	14.48	0.00	0.00
ENSSSCG00000029131	KCNU1	39.51	1.72	0.32	5.31	0.00	0.00
ENSSSCG00000017914	GLTPD2	13.63	1.72	0.53	3.25	0.00	0.00
ENSSSCG00000005020	ATL1	32.28	1.72	0.34	5.02	0.00	0.00
ENSSSCG00000028452	ERAS	9.39	1.73	0.67	2.59	0.01	0.02
ENSSSCG00000015984	HOXD4	69.84	1.73	0.23	7.40	0.00	0.00
ENSSSCG00000039245	RIMS3	56.91	1.73	0.26	6.66	0.00	0.00
ENSSSCG00000009658	BNIP3L	1284.17	1.73	0.07	25.64	0.00	0.00
ENSSSCG00000002349	ACOT4	2775.15	1.73	0.05	33.17	0.00	0.00
ENSSSCG00000011664	RBP1	571.98	1.73	0.11	15.45	0.00	0.00
ENSSSCG00000035673	HBP1	1171.32	1.73	0.07	26.58	0.00	0.00
ENSSSCG00000022689	GADD45B	130.17	1.73	0.17	10.01	0.00	0.00
ENSSSCG00000002931	ZNF565	5.77	1.73	0.80	2.16	0.03	0.05
ENSSSCG00000002348		261.94	1.74	0.13	13.87	0.00	0.00
ENSSSCG00000037614	RF01957	484.31	1.74	0.11	16.45	0.00	0.00
ENSSSCG00000005498	PAPPA	73.13	1.74	0.23	7.59	0.00	0.00
ENSSSCG00000010076	ZNF70	28.95	1.74	0.37	4.66	0.00	0.00
ENSSSCG00000036383	LGALS3BP	374.76	1.74	0.11	16.53	0.00	0.00
ENSSSCG00000013566	INSR	873.20	1.74	0.08	22.93	0.00	0.00
ENSSSCG00000002736	TAT	5.80	1.74	0.80	2.17	0.03	0.05
ENSSSCG00000005194	PTPRD	1659.03	1.74	0.06	29.10	0.00	0.00
ENSSSCG00000029815	SRGAP1	664.43	1.74	0.08	21.28	0.00	0.00
ENSSSCG00000038904		25.40	1.74	0.39	4.46	0.00	0.00

ENSSSCG00000022045	PTCHD3	5.81	1.74	0.80	2.18	0.03	0.05
ENSSSCG00000009426	CCDC122	13.11	1.74	0.55	3.16	0.00	0.00
ENSSSCG00000026425	ADAMTSL2	14.56	1.74	0.52	3.34	0.00	0.00
ENSSSCG00000029257		29.79	1.74	0.35	4.95	0.00	0.00
ENSSSCG00000014982	MMP7	22.61	1.75	0.42	4.21	0.00	0.00
ENSSSCG00000007998	RHBDL1	42.30	1.75	0.30	5.78	0.00	0.00
ENSSSCG00000036520	EFNA5	416.90	1.75	0.10	17.00	0.00	0.00
ENSSSCG00000000197	C1QL4	13.92	1.76	0.52	3.39	0.00	0.00
ENSSSCG00000025631		38.22	1.76	0.32	5.49	0.00	0.00
ENSSSCG00000014565		84.51	1.76	0.22	8.21	0.00	0.00
ENSSSCG00000013114	SLC15A3	16.91	1.77	0.48	3.66	0.00	0.00
ENSSSCG00000010831	DUSP10	221.96	1.77	0.14	12.56	0.00	0.00
ENSSSCG00000009048	GAB1	702.96	1.77	0.09	20.43	0.00	0.00
ENSSSCG00000017296	ACE	66.34	1.77	0.25	7.20	0.00	0.00
ENSSSCG00000015045	NCAM1	79.50	1.77	0.23	7.76	0.00	0.00
ENSSSCG00000036721	DEPDC4	6.66	1.77	0.75	2.37	0.02	0.03
ENSSSCG00000030358	SARM1	286.45	1.78	0.13	14.20	0.00	0.00
ENSSSCG00000008552	PRR30	9.59	1.78	0.64	2.78	0.01	0.01
ENSSSCG00000017237	CD300LB	14.05	1.78	0.52	3.45	0.00	0.00
ENSSSCG00000016782	OTULINL	34.03	1.78	0.34	5.23	0.00	0.00
ENSSSCG00000009317	CDX2	8.16	1.78	0.68	2.61	0.01	0.02
ENSSSCG00000034407	AMN1	136.19	1.78	0.18	9.97	0.00	0.00
ENSSSCG00000039953	PNRC1	640.70	1.78	0.09	20.70	0.00	0.00
ENSSSCG00000035596		47.35	1.78	0.30	5.94	0.00	0.00
ENSSSCG00000013506	STAP2	39.98	1.78	0.31	5.80	0.00	0.00

ENSSSCG00000015876	CCDC148	83.86	1.78	0.21	8.35	0.00	0.00
ENSSSCG00000011291		8.19	1.78	0.69	2.58	0.01	0.02
ENSSSCG00000008966	PARM1	1376.84	1.79	0.14	12.54	0.00	0.00
ENSSSCG00000000062	CSDC2	156.29	1.79	0.16	10.94	0.00	0.00
ENSSSCG00000025590		13.40	1.79	0.55	3.25	0.00	0.00
ENSSSCG00000009742	ULK1	731.68	1.79	0.08	21.81	0.00	0.00
ENSSSCG00000010432		224.18	1.79	0.14	12.51	0.00	0.00
ENSSSCG00000034491	PRICKLE1	231.81	1.79	0.13	13.37	0.00	0.00
ENSSSCG00000034261	TMEM59	1336.23	1.79	0.07	25.73	0.00	0.00
ENSSSCG00000034497		5.96	1.80	0.82	2.18	0.03	0.05
ENSSSCG00000002697		162.86	1.80	0.16	11.37	0.00	0.00
ENSSSCG00000012137	BMX	227.83	1.80	0.13	13.36	0.00	0.00
ENSSSCG00000003731	GAREM1	902.99	1.80	0.07	24.31	0.00	0.00
ENSSSCG00000015801	TLR3	2980.40	1.80	0.06	30.72	0.00	0.00
ENSSSCG00000014378		56.82	1.80	0.27	6.78	0.00	0.00
ENSSSCG00000021271		19.50	1.80	0.44	4.09	0.00	0.00
ENSSSCG00000003371	GPR153	18.02	1.81	0.47	3.85	0.00	0.00
ENSSSCG00000026196		899.92	1.81	0.07	24.19	0.00	0.00
ENSSSCG00000015882	BAZ2B	1159.71	1.81	0.08	23.18	0.00	0.00
ENSSSCG00000009179	MTTP	21.10	1.81	0.44	4.07	0.00	0.00
ENSSSCG00000021911	NDRG4	725.89	1.81	0.08	22.96	0.00	0.00
ENSSSCG00000014016	SQSTM1	8530.49	1.81	0.05	36.60	0.00	0.00
ENSSSCG00000011915	GRAMD1C	145.52	1.81	0.17	10.47	0.00	0.00
ENSSSCG00000009798	B3GNT4	6.77	1.81	0.74	2.44	0.01	0.02
ENSSSCG00000035689	NEXMIF	89.01	1.81	0.21	8.70	0.00	0.00

ENSSSCG00000033613	FOXS1	68.63	1.81	0.24	7.66	0.00	0.00
ENSSSCG00000022417	HNF1B	6745.24	1.82	0.06	31.53	0.00	0.00
ENSSSCG00000008973	NAAA	38.48	1.82	0.32	5.74	0.00	0.00
ENSSSCG00000037893		5.30	1.82	0.88	2.07	0.04	0.06
ENSSSCG00000003712	OSBPL1A	766.48	1.82	0.08	22.02	0.00	0.00
ENSSSCG00000034653	DLX4	5.28	1.82	0.88	2.08	0.04	0.06
ENSSSCG00000013252	F2	50.66	1.82	0.28	6.54	0.00	0.00
ENSSSCG00000006495	SEMA4A	12.88	1.82	0.54	3.36	0.00	0.00
ENSSSCG00000023280	SULT2B1	17.48	1.83	0.48	3.78	0.00	0.00
ENSSSCG00000036191	SLC17A2	104.05	1.83	0.19	9.47	0.00	0.00
ENSSSCG00000029888	CAMK2N2	33.56	1.83	0.35	5.24	0.00	0.00
ENSSSCG00000002013	DHRS4	6.09	1.83	0.79	2.32	0.02	0.03
ENSSSCG00000009872	PLBD2	3295.29	1.84	0.06	32.32	0.00	0.00
ENSSSCG00000036201	NPR3	203.37	1.84	0.15	12.30	0.00	0.00
ENSSSCG00000039089		247.02	1.84	0.13	14.33	0.00	0.00
ENSSSCG00000020725	ERBB3	1880.82	1.84	0.06	32.01	0.00	0.00
ENSSSCG00000005997	COL14A1	325.60	1.84	0.12	15.77	0.00	0.00
ENSSSCG00000032517	DMXL2	1459.26	1.84	0.06	28.38	0.00	0.00
ENSSSCG00000010064		85.11	1.84	0.21	8.63	0.00	0.00
ENSSSCG00000007508	ZBP1	22.23	1.84	0.42	4.41	0.00	0.00
ENSSSCG00000024635	SPART	190.56	1.85	0.15	12.29	0.00	0.00
ENSSSCG00000015589	VASH2	36.97	1.85	0.33	5.61	0.00	0.00
ENSSSCG00000035634	PLSCR1	1023.94	1.85	0.07	25.22	0.00	0.00
ENSSSCG00000026724	PLBD1	10.80	1.85	0.64	2.91	0.00	0.01
ENSSSCG00000027275	HHLA2	9.26	1.85	0.66	2.82	0.00	0.01

ENSSSCG00000010816	TGFB2	81.06	1.86	0.22	8.46	0.00	0.00
ENSSSCG00000000614	GRIN2B	9.29	1.86	0.64	2.88	0.00	0.01
ENSSSCG00000031348		38.63	1.86	0.32	5.74	0.00	0.00
ENSSSCG00000011587	EFCAB12	152.94	1.86	0.16	11.35	0.00	0.00
ENSSSCG00000008266	LOXL3	17.84	1.86	0.49	3.83	0.00	0.00
ENSSSCG00000033834	NDST4	36.40	1.86	0.33	5.68	0.00	0.00
ENSSSCG00000036022		14.75	1.87	0.51	3.65	0.00	0.00
ENSSSCG00000037234	CLDN2	548.52	1.87	0.09	20.37	0.00	0.00
ENSSSCG00000006289	F5	554.96	1.87	0.09	20.03	0.00	0.00
ENSSSCG00000012532	TCEAL1	74.74	1.87	0.24	7.95	0.00	0.00
ENSSSCG00000033585	DUSP23	83.23	1.87	0.22	8.63	0.00	0.00
ENSSSCG00000034036		15.54	1.87	0.50	3.76	0.00	0.00
ENSSSCG00000035849	PTPRF	766.81	1.87	0.08	23.26	0.00	0.00
ENSSSCG00000026564		23.41	1.87	0.41	4.61	0.00	0.00
ENSSSCG00000010533	PYROXD2	123.14	1.88	0.18	10.26	0.00	0.00
ENSSSCG00000005607	RALGPS1	29.65	1.88	0.37	5.06	0.00	0.00
ENSSSCG00000005486	KIF12	1133.55	1.88	0.08	24.10	0.00	0.00
ENSSSCG00000008959	CXCL2	324.80	1.88	0.13	13.95	0.00	0.00
ENSSSCG00000014540		59939.60	1.88	0.04	44.43	0.00	0.00
ENSSSCG00000011951	NFKBIZ	1222.61	1.88	0.07	27.52	0.00	0.00
ENSSSCG00000024388	BNIP3	1425.52	1.89	0.07	25.47	0.00	0.00
ENSSSCG00000005943	ST3GAL1	11.74	1.89	0.59	3.22	0.00	0.00
ENSSSCG00000009503		92.65	1.89	0.21	9.13	0.00	0.00
ENSSSCG00000003257	CACNG6	34.59	1.89	0.34	5.57	0.00	0.00
ENSSSCG00000020872		236.35	1.89	0.14	13.88	0.00	0.00

ENSSSCG00000035227	ESRP2	19.72	1.89	0.45	4.20	0.00	0.00
ENSSSCG00000017592	MBTD1	1827.29	1.89	0.06	33.70	0.00	0.00
ENSSSCG00000026164		7.86	1.90	0.72	2.65	0.01	0.01
ENSSSCG00000034625	PXMP4	558.51	1.90	0.09	20.46	0.00	0.00
ENSSSCG00000016691	JAZF1	10.26	1.90	0.61	3.10	0.00	0.00
ENSSSCG00000024109	BDH1	105.17	1.90	0.20	9.68	0.00	0.00
ENSSSCG00000012569	ATG4A	241.96	1.90	0.13	14.49	0.00	0.00
ENSSSCG00000031943		309.41	1.90	0.13	14.40	0.00	0.00
ENSSSCG00000032367	CEBPD	3183.63	1.90	0.12	15.87	0.00	0.00
ENSSSCG00000015559	NCF2	20.63	1.90	0.44	4.32	0.00	0.00
ENSSSCG00000010753	CLRN3	30.05	1.90	0.37	5.21	0.00	0.00
ENSSSCG00000004614	UNC13C	73.07	1.91	0.23	8.18	0.00	0.00
ENSSSCG00000021683		27.79	1.91	0.37	5.13	0.00	0.00
ENSSSCG00000000862	GNPTAB	4231.33	1.91	0.05	36.95	0.00	0.00
ENSSSCG00000025463	PROS1	909.05	1.91	0.08	24.07	0.00	0.00
ENSSSCG00000032053	ST8SIA5	4.77	1.91	0.91	2.11	0.04	0.05
ENSSSCG00000003146	NTN5	9.57	1.92	0.64	2.97	0.00	0.01
ENSSSCG00000013248	LRP4	19.17	1.92	0.46	4.21	0.00	0.00
ENSSSCG00000022672	TRPM4	145.33	1.92	0.17	11.09	0.00	0.00
ENSSSCG00000011782	ABCC5	1561.38	1.92	0.06	31.64	0.00	0.00
ENSSSCG00000016211		2376.92	1.93	0.06	32.98	0.00	0.00
ENSSSCG00000001916	INSYN1	4.81	1.93	0.92	2.09	0.04	0.06
ENSSSCG00000036396	RF01956	459.55	1.93	0.21	9.15	0.00	0.00
ENSSSCG00000040566		4.83	1.93	0.90	2.14	0.03	0.05
ENSSSCG00000007564	AMZ1	6.44	1.93	0.81	2.40	0.02	0.03

ENSSSCG00000001914	LOXL1	71.60	1.93	0.24	8.05	0.00	0.00
ENSSSCG00000024439	PTGER4	432.21	1.94	0.10	18.99	0.00	0.00
ENSSSCG00000040728	EYA1	133.26	1.94	0.18	10.75	0.00	0.00
ENSSSCG00000034633	CFAP99	6.45	1.94	0.78	2.48	0.01	0.02
ENSSSCG00000021067	BLVRB	2128.12	1.94	0.07	29.83	0.00	0.00
ENSSSCG00000040013	MTUS1	1569.23	1.94	0.07	29.17	0.00	0.00
ENSSSCG00000035267		18.63	1.94	0.46	4.19	0.00	0.00
ENSSSCG00000005902	FOXH1	15.35	1.94	0.50	3.87	0.00	0.00
ENSSSCG00000000997	PPP1R3G	210.16	1.95	0.14	13.61	0.00	0.00
ENSSSCG00000007094	DZANK1	845.61	1.95	0.08	24.93	0.00	0.00
ENSSSCG00000023434	PPM1L	275.94	1.95	0.13	14.54	0.00	0.00
ENSSSCG00000035668	C9orf135	31.01	1.96	0.37	5.36	0.00	0.00
ENSSSCG00000026517	CALML4	596.18	1.96	0.09	21.29	0.00	0.00
ENSSSCG00000037682	HFE	282.85	1.96	0.13	15.55	0.00	0.00
ENSSSCG00000010292	P4HA1	2870.39	1.96	0.07	27.32	0.00	0.00
ENSSSCG00000016057	STAT1	2894.10	1.97	0.06	32.39	0.00	0.00
ENSSSCG00000038912	IFITM3	553.48	1.97	0.09	21.29	0.00	0.00
ENSSSCG00000005269	TRPM6	63.31	1.97	0.25	7.85	0.00	0.00
ENSSSCG00000009784	ABCB9	51.80	1.97	0.29	6.92	0.00	0.00
ENSSSCG00000012523		36.29	1.98	0.33	5.96	0.00	0.00
ENSSSCG00000012298	CACNA1F	5.76	1.98	0.83	2.37	0.02	0.03
ENSSSCG00000038429		73.51	1.98	0.24	8.24	0.00	0.00
ENSSSCG00000008511		5.78	1.98	0.83	2.39	0.02	0.03
ENSSSCG00000038257	SGCZ	114.90	1.98	0.20	9.76	0.00	0.00
ENSSSCG00000023848	VXN	4.12	1.98	0.99	2.01	0.04	0.07

ENSSSCG00000016203	CFAP65	7.44	1.98	0.73	2.72	0.01	0.01
ENSSSCG00000005229	VLDLR	951.13	1.98	0.08	24.04	0.00	0.00
ENSSSCG00000024518		7.46	1.98	0.74	2.69	0.01	0.01
ENSSSCG00000003908	TSPAN1	353.37	1.98	0.12	15.89	0.00	0.00
ENSSSCG00000033266	C7orf57	21.54	1.99	0.43	4.65	0.00	0.00
ENSSSCG00000004896	PHLPP1	18.25	1.99	0.47	4.26	0.00	0.00
ENSSSCG00000037473		4.16	1.99	0.99	2.01	0.04	0.07
ENSSSCG00000010829		238.70	1.99	0.14	14.48	0.00	0.00
ENSSSCG00000002669	CRISPLD2	4.15	1.99	0.99	2.01	0.04	0.07
ENSSSCG00000030460	ARHGEF6	37.33	1.99	0.33	6.04	0.00	0.00
ENSSSCG00000004570	TPM1	12389.12	1.99	0.04	49.72	0.00	0.00
ENSSSCG00000022341	TMEM207	37.39	1.99	0.33	6.10	0.00	0.00
ENSSSCG00000012257	MAOA	281.82	1.99	0.13	15.65	0.00	0.00
ENSSSCG00000010433	SGMS1	1332.12	1.99	0.06	31.39	0.00	0.00
ENSSSCG00000032392	MYO15A	46.59	1.99	0.30	6.66	0.00	0.00
ENSSSCG00000006088	SDC2	902.96	2.00	0.08	25.68	0.00	0.00
ENSSSCG00000016885	ITGA1	2282.29	2.00	0.06	33.27	0.00	0.00
ENSSSCG00000036125		243.74	2.00	0.14	14.14	0.00	0.00
ENSSSCG00000036000		40.16	2.00	0.32	6.17	0.00	0.00
ENSSSCG00000009613	DMTN	179.01	2.00	0.16	12.91	0.00	0.00
ENSSSCG00000015375	ITGB8	900.18	2.00	0.08	25.12	0.00	0.00
ENSSSCG00000008615	VSNL1	250.46	2.01	0.13	15.51	0.00	0.00
ENSSSCG00000003740	ZSCAN30	7.50	2.01	0.77	2.61	0.01	0.02
ENSSSCG00000024392	THEMIS	22.72	2.01	0.43	4.68	0.00	0.00
ENSSSCG00000001931		21.00	2.01	0.43	4.63	0.00	0.00

ENSSSCG00000034746	CAPN8	86.48	2.01	0.22	9.21	0.00	0.00
ENSSSCG00000016567	STRIP2	65.59	2.01	0.25	8.09	0.00	0.00
ENSSSCG00000032841	RF01955	413.36	2.01	0.11	17.61	0.00	0.00
ENSSSCG00000032301		493.25	2.02	0.10	19.78	0.00	0.00
ENSSSCG00000011880	EAF2	8.41	2.02	0.71	2.83	0.00	0.01
ENSSSCG00000004902	RNF152	15.18	2.02	0.51	3.93	0.00	0.00
ENSSSCG00000022162	RAB11FIP5	1456.94	2.02	0.06	31.23	0.00	0.00
ENSSSCG00000008311	CYP26B1	61.78	2.02	0.26	7.92	0.00	0.00
ENSSSCG00000007171	EBF4	87.95	2.02	0.22	9.37	0.00	0.00
ENSSSCG00000004390	SESN1	162.81	2.03	0.16	12.79	0.00	0.00
ENSSSCG00000022719	RAB20	7.66	2.03	0.72	2.81	0.00	0.01
ENSSSCG00000001498	BEND6	76.42	2.03	0.23	8.70	0.00	0.00
ENSSSCG00000006702	FMO5	301.05	2.03	0.13	15.69	0.00	0.00
ENSSSCG00000002648	CBFA2T3	23.93	2.04	0.44	4.65	0.00	0.00
ENSSSCG00000028481	CALCOCO1	541.17	2.04	0.10	21.44	0.00	0.00
ENSSSCG00000038452	ADAMTS17	280.07	2.04	0.13	16.23	0.00	0.00
ENSSSCG00000002140		6.00	2.04	0.82	2.48	0.01	0.02
ENSSSCG00000026423	SLC23A1	797.84	2.04	0.08	24.73	0.00	0.00
ENSSSCG00000036076	SAPCD1	5.14	2.04	0.89	2.28	0.02	0.04
ENSSSCG00000010745	FANK1	11.17	2.05	0.61	3.34	0.00	0.00
ENSSSCG00000016527	SLC13A4	4.28	2.05	0.97	2.12	0.03	0.05
ENSSSCG00000005586	LHX2	5.17	2.05	0.88	2.32	0.02	0.03
ENSSSCG00000010456	PANK1	667.14	2.05	0.09	23.56	0.00	0.00
ENSSSCG00000000645	GABARAPL1	472.32	2.05	0.10	20.36	0.00	0.00
ENSSSCG00000017349	ADAM11	68.17	2.06	0.25	8.36	0.00	0.00

ENSSSCG00000018056	SRCIN1	12.98	2.06	0.56	3.65	0.00	0.00
ENSSSCG00000007899		86.49	2.06	0.22	9.22	0.00	0.00
ENSSSCG00000007642		4.32	2.06	0.97	2.12	0.03	0.05
ENSSSCG00000016986	CREBRF	179.23	2.06	0.16	12.90	0.00	0.00
ENSSSCG00000011102	NRP1	160.96	2.06	0.17	12.47	0.00	0.00
ENSSSCG00000015105	SLC37A4	1701.61	2.06	0.07	29.61	0.00	0.00
ENSSSCG00000032509	LRRC26	7.80	2.07	0.72	2.86	0.00	0.01
ENSSSCG00000037365		14.67	2.07	0.59	3.51	0.00	0.00
ENSSSCG00000023294	KRCC1	110.25	2.07	0.19	10.65	0.00	0.00
ENSSSCG00000017801	TRARG1	10.43	2.07	0.62	3.36	0.00	0.00
ENSSSCG00000020657	BCAM	900.39	2.07	0.08	26.43	0.00	0.00
ENSSSCG00000012202		601.06	2.07	0.09	22.76	0.00	0.00
ENSSSCG00000030325	C1QTNF6	1081.58	2.07	0.07	28.72	0.00	0.00
ENSSSCG00000004869	CNDP1	180.93	2.07	0.15	13.51	0.00	0.00
ENSSSCG00000005472	SLC46A2	10.43	2.07	0.63	3.30	0.00	0.00
ENSSSCG00000033739		8.68	2.07	0.70	2.94	0.00	0.01
ENSSSCG00000006049		1911.10	2.08	0.06	32.21	0.00	0.00
ENSSSCG00000009100	TNIP3	9.58	2.08	0.65	3.21	0.00	0.00
ENSSSCG00000011443	STAB1	12.20	2.08	0.57	3.62	0.00	0.00
ENSSSCG00000035707		82.89	2.08	0.23	9.13	0.00	0.00
ENSSSCG00000033843	ETFRF1	101.24	2.08	0.21	9.75	0.00	0.00
ENSSSCG00000022347	WNT11	230.55	2.08	0.14	15.26	0.00	0.00
ENSSSCG00000023192		35.86	2.08	0.34	6.21	0.00	0.00
ENSSSCG00000001718	CYP39A1	406.19	2.09	0.11	19.38	0.00	0.00
ENSSSCG00000032857	S100A12	259.92	2.09	0.14	15.37	0.00	0.00

ENSSSCG00000038843	ST3GAL2	597.18	2.09	0.10	21.68	0.00	0.00
ENSSSCG00000000040		17.62	2.09	0.49	4.31	0.00	0.00
ENSSSCG00000011456	CHDH	143.58	2.10	0.17	12.12	0.00	0.00
ENSSSCG00000003225		101.53	2.10	0.21	10.06	0.00	0.00
ENSSSCG00000031193		15.87	2.10	0.51	4.13	0.00	0.00
ENSSSCG00000022280	DACT3	10.59	2.10	0.62	3.39	0.00	0.00
ENSSSCG00000039512	RBP5	185.53	2.10	0.16	13.51	0.00	0.00
ENSSSCG00000021343	ZEB2	701.30	2.10	0.09	24.41	0.00	0.00
ENSSSCG00000008150	TMEM182	5.32	2.10	0.87	2.41	0.02	0.03
ENSSSCG00000038535	ARSB	783.35	2.11	0.08	25.67	0.00	0.00
ENSSSCG00000038283	KLK11	24.79	2.11	0.40	5.23	0.00	0.00
ENSSSCG00000005699	HMCN2	32.02	2.11	0.37	5.73	0.00	0.00
ENSSSCG00000033660	FIGNL2	8.88	2.11	0.69	3.06	0.00	0.00
ENSSSCG00000036417		55.06	2.11	0.27	7.72	0.00	0.00
ENSSSCG00000027266	PNPLA3	179.57	2.11	0.15	13.76	0.00	0.00
ENSSSCG00000034181	NKX3-2	18.62	2.11	0.48	4.38	0.00	0.00
ENSSSCG00000010570	KCNIP2	129.08	2.12	0.19	11.37	0.00	0.00
ENSSSCG00000033762	NATD1	21.46	2.12	0.45	4.75	0.00	0.00
ENSSSCG00000012121	EGFL6	76.71	2.12	0.23	9.04	0.00	0.00
ENSSSCG00000009829	CCDC63	4.46	2.12	0.98	2.17	0.03	0.05
ENSSSCG00000004439	TSPYL4	93.07	2.12	0.22	9.87	0.00	0.00
ENSSSCG00000038471	NUAK2	163.83	2.12	0.17	12.25	0.00	0.00
ENSSSCG00000015462	TPK1	569.00	2.13	0.09	22.47	0.00	0.00
ENSSSCG00000018357	RF00413	8.98	2.13	0.68	3.13	0.00	0.00
ENSSSCG00000017604	HLF	10.80	2.13	0.62	3.43	0.00	0.00

ENSSSCG00000015211	PKNOX2	6.28	2.13	0.80	2.67	0.01	0.01
ENSSSCG00000025561	VASN	209.39	2.13	0.15	14.62	0.00	0.00
ENSSSCG00000016981	CPEB4	2198.89	2.13	0.06	38.18	0.00	0.00
ENSSSCG00000016210	ABCB6	3240.10	2.13	0.06	36.74	0.00	0.00
ENSSSCG00000017885	SMTNL2	420.23	2.13	0.11	19.29	0.00	0.00
ENSSSCG00000023904	CFAP57	9.94	2.14	0.64	3.33	0.00	0.00
ENSSSCG00000005272	NMRK1	23.44	2.14	0.42	5.04	0.00	0.00
ENSSSCG00000024103	ADPRHL1	6.33	2.14	0.84	2.56	0.01	0.02
ENSSSCG00000013042		8.13	2.14	0.73	2.92	0.00	0.01
ENSSSCG00000007115	THBD	695.79	2.14	0.08	25.48	0.00	0.00
ENSSSCG00000027660	IFI44L	275.41	2.14	0.13	16.68	0.00	0.00
ENSSSCG00000033880		244.66	2.15	0.13	16.02	0.00	0.00
ENSSSCG00000033358	VPS37D	48.05	2.15	0.30	7.16	0.00	0.00
ENSSSCG00000000117	C22orf23	58.05	2.15	0.28	7.58	0.00	0.00
ENSSSCG00000031267	NIPAL2	257.00	2.15	0.13	16.29	0.00	0.00
ENSSSCG00000011911	DRD3	74.53	2.15	0.25	8.77	0.00	0.00
ENSSSCG00000037288	YPEL1	38.19	2.15	0.33	6.54	0.00	0.00
ENSSSCG00000015616	HSD11B1	10.95	2.16	0.61	3.54	0.00	0.00
ENSSSCG00000015806		26.41	2.16	0.39	5.47	0.00	0.00
ENSSSCG00000034376		5.49	2.16	0.87	2.47	0.01	0.02
ENSSSCG00000011147		9792.56	2.16	0.05	47.91	0.00	0.00
ENSSSCG00000008529	YPEL5	279.48	2.16	0.13	16.07	0.00	0.00
ENSSSCG00000016825	TTC23L	24.70	2.16	0.41	5.27	0.00	0.00
ENSSSCG00000029838	FZD2	46.65	2.16	0.30	7.27	0.00	0.00
ENSSSCG00000032078	ZNF362	261.97	2.16	0.14	15.90	0.00	0.00

ENSSSCG00000015200	ESAM	18.35	2.17	0.48	4.51	0.00	0.00
ENSSSCG00000000061	PMM1	302.16	2.17	0.12	17.85	0.00	0.00
ENSSSCG00000003439	DHRS3	621.49	2.17	0.09	23.24	0.00	0.00
ENSSSCG00000028327	RHOBTB1	323.81	2.17	0.12	18.03	0.00	0.00
ENSSSCG00000008307	SFXN5	5.52	2.17	0.91	2.39	0.02	0.03
ENSSSCG00000028536	LHFPL2	944.50	2.18	0.08	26.62	0.00	0.00
ENSSSCG00000025345	GUCY2D	16.63	2.18	0.50	4.35	0.00	0.00
ENSSSCG00000023933	CRACR2B	143.07	2.18	0.18	12.09	0.00	0.00
ENSSSCG00000000435		18.45	2.18	0.48	4.56	0.00	0.00
ENSSSCG00000023714	SLC31A2	32.43	2.18	0.37	5.88	0.00	0.00
ENSSSCG00000021479	RGS9BP	30.62	2.19	0.40	5.51	0.00	0.00
ENSSSCG00000024569	ANO9	112.23	2.19	0.20	11.06	0.00	0.00
ENSSSCG00000008943	SLC4A4	4695.65	2.19	0.05	43.31	0.00	0.00
ENSSSCG00000032137	PCDHGA9	7.44	2.19	0.78	2.81	0.00	0.01
ENSSSCG00000040093		27.88	2.19	0.40	5.45	0.00	0.00
ENSSSCG00000010449	CH25H	4.64	2.19	0.95	2.29	0.02	0.03
ENSSSCG00000027688	RNF183	156.90	2.19	0.18	12.46	0.00	0.00
ENSSSCG00000027045	LRRC56	125.49	2.19	0.20	11.23	0.00	0.00
ENSSSCG00000001846	WDR93	15.75	2.19	0.54	4.08	0.00	0.00
ENSSSCG00000040095	GPR35	30.74	2.19	0.38	5.82	0.00	0.00
ENSSSCG00000004024		203.24	2.20	0.15	14.58	0.00	0.00
ENSSSCG00000003951	C1orf210	574.44	2.20	0.09	23.33	0.00	0.00
ENSSSCG00000015401		1969.56	2.20	0.06	35.75	0.00	0.00
ENSSSCG00000022830	KANSL1L	287.65	2.20	0.13	16.65	0.00	0.00
ENSSSCG00000009504	HS6ST3	112.56	2.20	0.20	10.85	0.00	0.00

ENSSSCG00000030076	SLC6A13	14.04	2.21	0.54	4.05	0.00	0.00
ENSSSCG00000001723	PLA2G7	54.54	2.21	0.28	7.88	0.00	0.00
ENSSSCG00000000110	PLA2G6	310.93	2.21	0.12	18.11	0.00	0.00
ENSSSCG00000006506	SYT11	92.26	2.21	0.22	10.15	0.00	0.00
ENSSSCG00000039322	NRGN	130.14	2.22	0.19	11.80	0.00	0.00
ENSSSCG00000003854	ECHDC2	720.21	2.22	0.09	25.72	0.00	0.00
ENSSSCG00000009152	SGMS2	888.99	2.22	0.08	27.53	0.00	0.00
ENSSSCG00000013010	NAALADL1	15.16	2.22	0.53	4.15	0.00	0.00
ENSSSCG00000005423	ABCA1	377.74	2.22	0.11	19.92	0.00	0.00
ENSSSCG00000033196	MYORG	675.42	2.22	0.09	24.00	0.00	0.00
ENSSSCG00000003572	SYTL1	77.79	2.23	0.24	9.44	0.00	0.00
ENSSSCG00000013004	TM7SF2	37.98	2.23	0.34	6.59	0.00	0.00
ENSSSCG00000025523	COL2A1	7.60	2.23	0.74	3.03	0.00	0.00
ENSSSCG00000007281	MAP1LC3A	143.64	2.23	0.18	12.54	0.00	0.00
ENSSSCG00000013302	CAT	4025.06	2.24	0.06	39.39	0.00	0.00
ENSSSCG00000013276	PRDM11	11.50	2.24	0.61	3.64	0.00	0.00
ENSSSCG00000035854	RAB11FIP3	1357.51	2.24	0.07	34.10	0.00	0.00
ENSSSCG00000004729		60.23	2.24	0.27	8.42	0.00	0.00
ENSSSCG00000036946		12.44	2.24	0.60	3.75	0.00	0.00
ENSSSCG00000009316		10.52	2.24	0.64	3.49	0.00	0.00
ENSSSCG00000033009		136.11	2.24	0.19	12.11	0.00	0.00
ENSSSCG00000028148	DMD	651.51	2.25	0.09	25.02	0.00	0.00
ENSSSCG00000003909	PIK3R3	438.30	2.25	0.11	19.90	0.00	0.00
ENSSSCG00000024678	MEGF6	678.29	2.25	0.09	24.40	0.00	0.00
ENSSSCG00000004109	ZC3H12D	42.38	2.25	0.32	7.01	0.00	0.00

ENSSSCG00000023932	PCDHGB7	6.73	2.25	0.80	2.82	0.00	0.01
ENSSSCG00000037549	GCLM	5130.98	2.25	0.04	51.58	0.00	0.00
ENSSSCG00000015069	APOC3	18.29	2.25	0.48	4.66	0.00	0.00
ENSSSCG00000021646	KLF9	457.35	2.26	0.11	20.48	0.00	0.00
ENSSSCG00000006726	SPAG17	438.33	2.26	0.11	20.37	0.00	0.00
ENSSSCG00000010853	EPHX1	1458.09	2.26	0.06	35.43	0.00	0.00
ENSSSCG00000035331		144.83	2.26	0.17	12.96	0.00	0.00
ENSSSCG00000028706	MORN1	13.48	2.26	0.57	3.98	0.00	0.00
ENSSSCG00000004234	FABP7	16.49	2.26	0.51	4.40	0.00	0.00
ENSSSCG00000035038	CGNL1	2654.19	2.27	0.06	38.67	0.00	0.00
ENSSSCG00000010403	MARCH8	678.91	2.27	0.09	24.19	0.00	0.00
ENSSSCG00000027860	ERAP2	114.32	2.28	0.20	11.27	0.00	0.00
ENSSSCG00000040910	APOH	9.78	2.28	0.66	3.47	0.00	0.00
ENSSSCG00000017186	RNF157	8.81	2.28	0.69	3.28	0.00	0.00
ENSSSCG00000005045	BMP4	604.71	2.29	0.10	22.05	0.00	0.00
ENSSSCG00000026614	RF01164	39.32	2.29	0.34	6.67	0.00	0.00
ENSSSCG00000031329	ST8SIA1	28.61	2.30	0.39	5.92	0.00	0.00
ENSSSCG00000032197	GNG3	69.28	2.30	0.25	9.04	0.00	0.00
ENSSSCG00000035125		7.90	2.30	0.73	3.14	0.00	0.00
ENSSSCG00000031959	CAPSL	43.59	2.30	0.32	7.29	0.00	0.00
ENSSSCG00000026129		204.02	2.30	0.15	15.45	0.00	0.00
ENSSSCG00000012572	COL4A5	124.16	2.30	0.19	11.88	0.00	0.00
ENSSSCG00000012038	C21orf62	35.74	2.31	0.35	6.64	0.00	0.00
ENSSSCG00000015345	GLCCI1	1205.08	2.31	0.07	33.56	0.00	0.00
ENSSSCG00000040288	ARNT2	783.29	2.31	0.08	27.94	0.00	0.00

ENSSSCG00000010655	GFRA1	3.99	2.31	1.04	2.22	0.03	0.04
ENSSSCG00000011972	FILIP1L	267.38	2.31	0.14	17.11	0.00	0.00
ENSSSCG00000031474	PTK6	9.98	2.32	0.71	3.28	0.00	0.00
ENSSSCG00000010142	RYR2	28.05	2.32	0.40	5.78	0.00	0.00
ENSSSCG00000017886	FBXO39	40.12	2.32	0.33	7.08	0.00	0.00
ENSSSCG00000031761		12.03	2.32	0.62	3.74	0.00	0.00
ENSSSCG00000004475		608.86	2.32	0.09	24.75	0.00	0.00
ENSSSCG00000033913	C1RL	82.36	2.33	0.23	10.08	0.00	0.00
ENSSSCG00000010060	UPB1	52.40	2.33	0.29	7.99	0.00	0.00
ENSSSCG00000027609	GC	28.25	2.33	0.39	5.95	0.00	0.00
ENSSSCG00000036454	TMEM220	48.44	2.33	0.30	7.84	0.00	0.00
ENSSSCG00000029460	RND2	33.44	2.34	0.38	6.17	0.00	0.00
ENSSSCG00000009229	ARHGAP24	510.10	2.34	0.10	23.49	0.00	0.00
ENSSSCG00000012132	ASB9	44.72	2.35	0.31	7.50	0.00	0.00
ENSSSCG00000034763	IRS2	107.80	2.35	0.20	11.61	0.00	0.00
ENSSSCG00000025133	ITGB2	7.13	2.35	0.78	3.02	0.00	0.00
ENSSSCG00000004923	SLC51B	111.98	2.35	0.20	11.78	0.00	0.00
ENSSSCG00000002815	ADGRG1	8956.39	2.36	0.06	42.84	0.00	0.00
ENSSSCG00000013455	IZUMO4	17.41	2.36	0.50	4.73	0.00	0.00
ENSSSCG00000000687	CD4	5.11	2.36	0.92	2.55	0.01	0.02
ENSSSCG00000036541		5.14	2.36	0.93	2.54	0.01	0.02
ENSSSCG00000012027	ADAMTS5	4.10	2.36	1.03	2.30	0.02	0.03
ENSSSCG00000004233	SMPDL3A	66.80	2.37	0.26	9.10	0.00	0.00
ENSSSCG00000006935	CLCA2	16.45	2.37	0.53	4.48	0.00	0.00
ENSSSCG00000032601	RASSF2	23.76	2.37	0.43	5.48	0.00	0.00

ENSSSCG00000036854		4.11	2.37	1.08	2.20	0.03	0.04
ENSSSCG00000033735	PERM1	8.26	2.37	0.73	3.25	0.00	0.00
ENSSSCG00000003133	GRIN2D	3.09	2.38	1.20	1.98	0.05	0.07
ENSSSCG00000010621	ADD3	4218.37	2.38	0.05	46.63	0.00	0.00
ENSSSCG00000002675	DNAAF1	13.47	2.38	0.59	4.05	0.00	0.00
ENSSSCG00000039413	SLC9A3	216.96	2.38	0.15	16.38	0.00	0.00
ENSSSCG00000010452	IFIT1	14.59	2.39	0.55	4.35	0.00	0.00
ENSSSCG00000015196	SIAE	216.10	2.39	0.15	16.01	0.00	0.00
ENSSSCG00000011119	ECHDC3	500.26	2.39	0.10	23.88	0.00	0.00
ENSSSCG00000040260		207.90	2.39	0.15	15.96	0.00	0.00
ENSSSCG00000011867	MYLK	6.28	2.40	0.83	2.88	0.00	0.01
ENSSSCG00000010468	CPEB3	95.64	2.40	0.22	11.02	0.00	0.00
ENSSSCG00000001457	SLA-DQB1	26.30	2.40	0.42	5.71	0.00	0.00
ENSSSCG00000014575		3.14	2.40	1.19	2.03	0.04	0.07
ENSSSCG00000022296	CDK15	6.32	2.40	0.83	2.88	0.00	0.01
ENSSSCG00000021588	DAPK2	18.94	2.40	0.49	4.94	0.00	0.00
ENSSSCG00000003732	MEP1B	3.18	2.41	1.22	1.97	0.05	0.07
ENSSSCG00000006213		120.15	2.41	0.20	12.33	0.00	0.00
ENSSSCG00000012399	FOXO4	87.76	2.42	0.23	10.45	0.00	0.00
ENSSSCG00000031086		5.30	2.42	0.93	2.59	0.01	0.02
ENSSSCG00000036305		142.87	2.42	0.18	13.08	0.00	0.00
ENSSSCG00000032538	GCM1	5.29	2.42	0.91	2.66	0.01	0.01
ENSSSCG00000031112	TM4SF5	28.62	2.42	0.40	6.05	0.00	0.00
ENSSSCG00000028204	TREH	48.93	2.43	0.31	7.94	0.00	0.00
ENSSSCG00000004678	DUOX2	42.53	2.43	0.35	7.01	0.00	0.00

ENSSSCG00000021811	GDPD3	4.25	2.43	1.02	2.37	0.02	0.03
ENSSSCG00000034358	KCNJ16	421.17	2.43	0.11	22.02	0.00	0.00
ENSSSCG00000008988	CCNG2	125.15	2.43	0.19	12.52	0.00	0.00
ENSSSCG00000001657	CUL7	123.01	2.43	0.20	12.26	0.00	0.00
ENSSSCG00000004702	STRC	41.82	2.44	0.33	7.34	0.00	0.00
ENSSSCG00000017084	FAT2	165.18	2.44	0.17	14.10	0.00	0.00
ENSSSCG00000033765	PCDHGA3	12.89	2.44	0.59	4.14	0.00	0.00
ENSSSCG00000038993	HOXC4	8.62	2.44	0.72	3.38	0.00	0.00
ENSSSCG00000017638	TSPOAP1	294.07	2.45	0.13	18.74	0.00	0.00
ENSSSCG00000015563	RGL1	1151.02	2.45	0.08	32.18	0.00	0.00
ENSSSCG00000008034	NOXO1	35.60	2.45	0.36	6.81	0.00	0.00
ENSSSCG00000011965	TMEM45A	144.30	2.46	0.19	12.99	0.00	0.00
ENSSSCG00000038184	ACBD7	21.70	2.46	0.46	5.39	0.00	0.00
ENSSSCG00000012076	MX2	32.58	2.46	0.37	6.59	0.00	0.00
ENSSSCG00000024476	CES3	634.22	2.47	0.09	26.82	0.00	0.00
ENSSSCG00000039161	MEIS1	113.68	2.47	0.20	12.33	0.00	0.00
ENSSSCG00000018015	DNAH9	35.02	2.47	0.36	6.83	0.00	0.00
ENSSSCG00000004760		5.46	2.47	0.91	2.73	0.01	0.01
ENSSSCG00000031287		10.98	2.47	0.65	3.82	0.00	0.00
ENSSSCG00000026499	NMT2	394.31	2.47	0.11	22.15	0.00	0.00
ENSSSCG00000004452	PRSS35	174.48	2.48	0.17	14.30	0.00	0.00
ENSSSCG00000004576	RORA	41.76	2.48	0.33	7.46	0.00	0.00
ENSSSCG00000033736		11.03	2.48	0.66	3.75	0.00	0.00
ENSSSCG00000001473	COL11A2	3.29	2.48	1.17	2.12	0.03	0.05
ENSSSCG00000025686	KMO	3.31	2.48	1.17	2.13	0.03	0.05

ENSSSCG00000008118	PROM2	388.83	2.49	0.12	20.81	0.00	0.00
ENSSSCG00000003784	LRRIQ3	3.32	2.49	1.17	2.13	0.03	0.05
ENSSSCG00000022758	PECR	816.75	2.49	0.09	28.91	0.00	0.00
ENSSSCG00000010241	TET1	280.90	2.49	0.13	18.69	0.00	0.00
ENSSSCG00000023187		2959.80	2.49	0.05	45.78	0.00	0.00
ENSSSCG00000031207	PLAC1	17.75	2.49	0.50	4.94	0.00	0.00
ENSSSCG00000033800	PELI2	37.74	2.50	0.36	6.98	0.00	0.00
ENSSSCG00000026486		23.34	2.50	0.44	5.68	0.00	0.00
ENSSSCG00000015035	C11orf52	24.45	2.50	0.46	5.48	0.00	0.00
ENSSSCG00000016083	PLCL1	7.81	2.50	0.76	3.30	0.00	0.00
ENSSSCG00000003129	ZSWIM9	16.75	2.50	0.52	4.81	0.00	0.00
ENSSSCG00000001780	FAH	978.55	2.51	0.08	31.75	0.00	0.00
ENSSSCG00000034345	SPRY3	26.83	2.51	0.41	6.06	0.00	0.00
ENSSSCG00000000259	CSAD	175.76	2.51	0.17	15.17	0.00	0.00
ENSSSCG00000035218	ADA2	119.98	2.51	0.20	12.52	0.00	0.00
ENSSSCG00000031976	ANKRD53	4.50	2.51	1.00	2.51	0.01	0.02
ENSSSCG00000012136	PIR	160.74	2.53	0.17	14.76	0.00	0.00
ENSSSCG00000011884	HCLS1	5.67	2.53	0.91	2.79	0.01	0.01
ENSSSCG00000006834	MYBPHL	14.77	2.53	0.58	4.37	0.00	0.00
ENSSSCG00000016334	SCLY	316.11	2.53	0.13	19.77	0.00	0.00
ENSSSCG00000018052	NT5M	13.60	2.53	0.58	4.34	0.00	0.00
ENSSSCG00000017676	BCAS3	112.39	2.54	0.20	12.42	0.00	0.00
ENSSSCG00000035424		72.75	2.54	0.26	9.85	0.00	0.00
ENSSSCG00000006035	ANGPT1	37.52	2.54	0.35	7.15	0.00	0.00
ENSSSCG00000036436	FZD1	925.35	2.54	0.08	31.38	0.00	0.00

ENSSSCG00000036340	ZBTB5	88.96	2.54	0.23	10.93	0.00	0.00
ENSSSCG00000010009	GAL3ST1	80.99	2.54	0.25	10.36	0.00	0.00
ENSSSCG00000016295	NGEF	219.69	2.55	0.15	16.66	0.00	0.00
ENSSSCG00000039573	SLPI	126.29	2.55	0.20	13.04	0.00	0.00
ENSSSCG00000025598	COBLL1	841.97	2.55	0.09	29.39	0.00	0.00
ENSSSCG00000027882	C1orf115	155.93	2.56	0.17	14.66	0.00	0.00
ENSSSCG00000015825	ADGRA2	5.78	2.57	0.90	2.84	0.00	0.01
ENSSSCG00000039841	SHC3	3.47	2.57	1.14	2.24	0.02	0.04
ENSSSCG00000005723	NTNG2	6.97	2.57	0.81	3.16	0.00	0.00
ENSSSCG00000031423	UPK3A	6.95	2.57	0.84	3.06	0.00	0.00
ENSSSCG00000037251		22.14	2.58	0.48	5.42	0.00	0.00
ENSSSCG00000012300	FOXP3	3.51	2.58	1.19	2.18	0.03	0.05
ENSSSCG00000004413	PPIL6	38.53	2.58	0.36	7.26	0.00	0.00
ENSSSCG00000032580	MGST1	767.99	2.59	0.10	26.18	0.00	0.00
ENSSSCG00000002811		14.11	2.59	0.61	4.26	0.00	0.00
ENSSSCG00000026618	CAVIN2	35.22	2.59	0.37	6.91	0.00	0.00
ENSSSCG00000028699	NEIL2	108.06	2.59	0.22	11.69	0.00	0.00
ENSSSCG00000028124	SNRPN	686.65	2.60	0.09	29.06	0.00	0.00
ENSSSCG00000016213	GLB1L	582.72	2.60	0.10	26.50	0.00	0.00
ENSSSCG00000012238		419.97	2.60	0.11	23.46	0.00	0.00
ENSSSCG00000022676	OPHN1	172.41	2.60	0.17	15.12	0.00	0.00
ENSSSCG00000012138	ACE2	131.07	2.60	0.19	13.54	0.00	0.00
ENSSSCG00000030137	LMNTD2	10.66	2.60	0.67	3.91	0.00	0.00
ENSSSCG00000001847	MESP1	10.65	2.60	0.69	3.79	0.00	0.00
ENSSSCG00000001456		47.53	2.61	0.33	7.85	0.00	0.00

ENSSSCG00000031477	GLOD5	162.93	2.61	0.18	14.59	0.00	0.00
ENSSSCG00000004791	RASGRP1	215.81	2.62	0.15	17.35	0.00	0.00
ENSSSCG00000034133		52.58	2.62	0.30	8.71	0.00	0.00
ENSSSCG00000012971	EFEMP2	41.87	2.62	0.34	7.76	0.00	0.00
ENSSSCG00000004401	DDO	178.41	2.62	0.17	15.71	0.00	0.00
ENSSSCG00000008314	ATP6V1B1	129.50	2.63	0.20	13.20	0.00	0.00
ENSSSCG00000006979	MSR1	4.81	2.63	1.00	2.64	0.01	0.01
ENSSSCG00000003780	CRYZ	1255.40	2.63	0.08	34.00	0.00	0.00
ENSSSCG00000017416	DHX58	12.00	2.63	0.65	4.06	0.00	0.00
ENSSSCG00000026842	CDH18	3.58	2.63	1.17	2.25	0.02	0.04
ENSSSCG00000021968	SLC19A3	2415.57	2.63	0.06	42.36	0.00	0.00
ENSSSCG00000027053	PACSIN1	8.44	2.64	0.74	3.55	0.00	0.00
ENSSSCG00000008449	SLC3A1	7207.97	2.64	0.05	55.53	0.00	0.00
ENSSSCG00000023479		7.24	2.64	0.82	3.23	0.00	0.00
ENSSSCG00000005240	DOCK8	2418.40	2.64	0.07	40.13	0.00	0.00
ENSSSCG00000017717	UNC45B	12.08	2.64	0.63	4.19	0.00	0.00
ENSSSCG00000003834	C8B	3.64	2.65	1.15	2.31	0.02	0.03
ENSSSCG00000037539	SORCS2	7.27	2.65	0.85	3.13	0.00	0.00
ENSSSCG00000000801	TMEM117	651.35	2.66	0.09	28.44	0.00	0.00
ENSSSCG00000011799	AHSG	17.08	2.66	0.54	4.94	0.00	0.00
ENSSSCG00000001236	TRIM40	4.89	2.66	1.07	2.48	0.01	0.02
ENSSSCG00000012993	SLC25A45	30.64	2.66	0.40	6.58	0.00	0.00
ENSSSCG00000023090	BEST1	15.94	2.67	0.58	4.60	0.00	0.00
ENSSSCG00000035915	ABHD16B	8.61	2.67	0.74	3.59	0.00	0.00
ENSSSCG00000006862	VCAM1	82.58	2.67	0.24	10.93	0.00	0.00

ENSSSCG00000016855	FYB1	4.93	2.68	0.99	2.71	0.01	0.01
ENSSSCG00000021490	PRDM16	9.85	2.68	0.72	3.74	0.00	0.00
ENSSSCG00000010814	ESRRG	142.47	2.68	0.19	14.12	0.00	0.00
ENSSSCG00000035859	WNT5A	243.18	2.68	0.15	18.26	0.00	0.00
ENSSSCG00000038207	ssc-mir-503	91.97	2.69	0.24	11.10	0.00	0.00
ENSSSCG00000016746	NPC1L1	14.92	2.69	0.58	4.60	0.00	0.00
ENSSSCG00000030821		8.72	2.70	0.74	3.62	0.00	0.00
ENSSSCG00000029570	PXDN	28.85	2.70	0.42	6.45	0.00	0.00
ENSSSCG00000037706	PRKAR2B	50.08	2.70	0.32	8.56	0.00	0.00
ENSSSCG00000001469	SLA-DMB	56.53	2.70	0.30	9.10	0.00	0.00
ENSSSCG00000000802	NELL2	8.82	2.71	0.74	3.65	0.00	0.00
ENSSSCG00000010026	PIK3IP1	190.28	2.71	0.17	15.82	0.00	0.00
ENSSSCG00000009245	SCD5	13.87	2.71	0.59	4.57	0.00	0.00
ENSSSCG00000004434	FRK	229.49	2.71	0.15	18.18	0.00	0.00
ENSSSCG00000003590	PTPRU	67.18	2.72	0.27	9.89	0.00	0.00
ENSSSCG00000038811	MOB3B	369.43	2.72	0.13	21.30	0.00	0.00
ENSSSCG00000031533	PCDHGB6	11.39	2.72	0.65	4.19	0.00	0.00
ENSSSCG00000025836	SULT1C4	107.65	2.72	0.21	12.69	0.00	0.00
ENSSSCG00000030305	SLC15A2	69.71	2.72	0.26	10.26	0.00	0.00
ENSSSCG00000009565	GAS6	30.56	2.73	0.40	6.75	0.00	0.00
ENSSSCG00000011704	WWTR1	427.63	2.73	0.12	22.85	0.00	0.00
ENSSSCG00000036033	THRB	38.25	2.73	0.37	7.48	0.00	0.00
ENSSSCG00000029305	FNDC4	15.29	2.73	0.57	4.82	0.00	0.00
ENSSSCG00000014851	SLCO2B1	100.89	2.73	0.23	12.03	0.00	0.00
ENSSSCG00000038639	TOM1L2	603.03	2.73	0.10	28.19	0.00	0.00

ENSSSCG00000024926	ASGR1	10.26	2.74	0.69	3.96	0.00	0.00
ENSSSCG00000013181	SERPING1	194.18	2.74	0.17	16.03	0.00	0.00
ENSSSCG00000010201	RASGEF1A	130.08	2.76	0.20	13.83	0.00	0.00
ENSSSCG00000021880	MXRA8	252.64	2.76	0.15	18.82	0.00	0.00
ENSSSCG00000028115	ALDH8A1	3.90	2.77	1.16	2.39	0.02	0.03
ENSSSCG00000027197	DYRK1B	216.63	2.77	0.16	17.78	0.00	0.00
ENSSSCG00000014672		53.61	2.77	0.31	9.02	0.00	0.00
ENSSSCG00000032436		2.63	2.79	1.39	2.01	0.04	0.07
ENSSSCG00000011400	SEMA3B	552.91	2.79	0.10	26.86	0.00	0.00
ENSSSCG00000013604	MYO1F	3.97	2.79	1.12	2.49	0.01	0.02
ENSSSCG00000006950	WDR63	752.39	2.79	0.10	29.31	0.00	0.00
ENSSSCG00000013762	PODNL1	3.98	2.79	1.12	2.49	0.01	0.02
ENSSSCG00000010404	ALOX5	17.27	2.79	0.54	5.19	0.00	0.00
ENSSSCG00000029576	ZNF239	2.66	2.80	1.39	2.02	0.04	0.07
ENSSSCG00000009827	HVCN1	227.35	2.80	0.16	17.70	0.00	0.00
ENSSSCG00000016892	FST	16.03	2.81	0.56	4.97	0.00	0.00
ENSSSCG00000008510	LTBP1	1496.80	2.81	0.08	37.41	0.00	0.00
ENSSSCG00000008687	MXD4	209.38	2.81	0.16	17.49	0.00	0.00
ENSSSCG00000027621	NDUFA4L2	6.69	2.82	0.94	2.99	0.00	0.01
ENSSSCG00000000151		17.53	2.82	0.54	5.25	0.00	0.00
ENSSSCG00000015079	BACE1	628.08	2.82	0.10	27.90	0.00	0.00
ENSSSCG00000010861	COQ8A	68.92	2.83	0.28	10.26	0.00	0.00
ENSSSCG00000011906	IGSF11	1476.08	2.83	0.07	38.44	0.00	0.00
ENSSSCG00000034119	MEIG1	27.12	2.83	0.43	6.59	0.00	0.00
ENSSSCG00000039009		33.93	2.83	0.39	7.18	0.00	0.00

ENSSSCG00000029248		1581.82	2.84	0.08	35.68	0.00	0.00
ENSSSCG00000028672	GCKR	165.81	2.85	0.19	15.34	0.00	0.00
ENSSSCG00000006163	PKIA	209.67	2.85	0.16	17.44	0.00	0.00
ENSSSCG00000021920	SOX12	4.13	2.86	1.10	2.59	0.01	0.02
ENSSSCG00000015985	HOXD3	164.58	2.86	0.18	15.87	0.00	0.00
ENSSSCG00000039272	IP6K3	52.60	2.87	0.31	9.14	0.00	0.00
ENSSSCG00000034993	NREP	17830.19	2.87	0.05	55.59	0.00	0.00
ENSSSCG00000023627	LYPD6	252.06	2.88	0.15	19.81	0.00	0.00
ENSSSCG00000001453	HLA-DRA	6.95	2.88	0.87	3.31	0.00	0.00
ENSSSCG00000003218	MYBPC2	5.57	2.88	0.98	2.94	0.00	0.01
ENSSSCG00000028969	SLC26A4	6.99	2.88	0.86	3.34	0.00	0.00
ENSSSCG00000039081		2.79	2.88	1.35	2.13	0.03	0.05
ENSSSCG00000035152	TEF	51.59	2.88	0.32	8.96	0.00	0.00
ENSSSCG00000010086		19.56	2.88	0.52	5.52	0.00	0.00
ENSSSCG00000025629	COLEC11	2.81	2.89	1.34	2.15	0.03	0.05
ENSSSCG00000027157	SLC40A1	77.33	2.89	0.27	10.78	0.00	0.00
ENSSSCG00000040622	HIST3H2A	2.82	2.90	1.35	2.15	0.03	0.05
ENSSSCG00000010879	KIF26B	14.14	2.90	0.61	4.78	0.00	0.00
ENSSSCG00000024881	TCP11L2	62.38	2.90	0.29	10.07	0.00	0.00
ENSSSCG00000025266	VWA3A	2.85	2.91	1.37	2.11	0.03	0.05
ENSSSCG00000016442	AOC1	18.50	2.91	0.55	5.33	0.00	0.00
ENSSSCG00000011556		7.15	2.91	0.85	3.42	0.00	0.00
ENSSSCG00000035400	YPEL2	1734.81	2.93	0.08	36.81	0.00	0.00
ENSSSCG00000009684	MSRA	148.69	2.93	0.19	15.33	0.00	0.00
ENSSSCG00000039535	CAMK2B	5.76	2.93	0.95	3.08	0.00	0.00

ENSSSCG00000016290	EFHD1	69.38	2.94	0.28	10.31	0.00	0.00
ENSSSCG00000035893		258.95	2.94	0.16	18.71	0.00	0.00
ENSSSCG00000010523	HOGA1	313.97	2.94	0.14	21.52	0.00	0.00
ENSSSCG00000022099	TP53INP2	650.46	2.94	0.10	28.44	0.00	0.00
ENSSSCG00000007133	ACSS1	11.59	2.94	0.67	4.39	0.00	0.00
ENSSSCG00000000131	ELFN2	27.47	2.94	0.44	6.68	0.00	0.00
ENSSSCG00000016141	PLEKHM3	20.37	2.95	0.51	5.77	0.00	0.00
ENSSSCG00000016244	COL4A4	1303.59	2.95	0.08	36.39	0.00	0.00
ENSSSCG00000017738	ADAP2	5.85	2.95	0.96	3.08	0.00	0.00
ENSSSCG00000010089		11.69	2.96	0.67	4.40	0.00	0.00
ENSSSCG00000035121	RHBG	221.17	2.96	0.16	18.45	0.00	0.00
ENSSSCG00000025870	HEPACAM2	10.30	2.96	0.72	4.11	0.00	0.00
ENSSSCG00000009893	TMEM116	42.67	2.97	0.35	8.42	0.00	0.00
ENSSSCG00000014368		2.93	2.97	1.37	2.17	0.03	0.05
ENSSSCG00000021654	ZNF641	25.10	2.97	0.46	6.48	0.00	0.00
ENSSSCG00000017003	KCNIP1	2.97	2.98	1.33	2.25	0.02	0.04
ENSSSCG00000017583	SGCA	7.42	2.98	0.85	3.52	0.00	0.00
ENSSSCG00000022975	AMT	129.49	2.98	0.21	14.35	0.00	0.00
ENSSSCG00000017144		2.99	2.98	1.38	2.16	0.03	0.05
ENSSSCG00000004673	SLC28A2	4.47	2.98	1.08	2.77	0.01	0.01
ENSSSCG00000029521	CD82	47.72	2.98	0.34	8.65	0.00	0.00
ENSSSCG00000037206		17.89	2.99	0.55	5.42	0.00	0.00
ENSSSCG00000017117	SLC6A18	2.98	2.99	1.33	2.25	0.02	0.04
ENSSSCG00000003967	ZMYND12	3.00	2.99	1.35	2.21	0.03	0.04
ENSSSCG00000012911	CARNS1	42.08	3.00	0.38	7.95	0.00	0.00

ENSSSCG00000004616	ONECUT1	10.55	3.01	0.71	4.25	0.00	0.00
ENSSSCG00000010593	CNNM2	98.10	3.01	0.23	12.80	0.00	0.00
ENSSSCG00000007674	ZAN	9.04	3.01	0.80	3.75	0.00	0.00
ENSSSCG00000001909	STRA6	300.21	3.01	0.14	21.65	0.00	0.00
ENSSSCG00000005858	RNF208	12.19	3.02	0.66	4.55	0.00	0.00
ENSSSCG00000024061	TNNI1	62.74	3.03	0.30	10.03	0.00	0.00
ENSSSCG00000039332	SEC16B	4.61	3.04	1.09	2.80	0.01	0.01
ENSSSCG00000010627	PDCD4	250.80	3.05	0.15	20.30	0.00	0.00
ENSSSCG00000027796	SLC22A7	17.06	3.05	0.57	5.34	0.00	0.00
ENSSSCG00000002309	PLEKHD1	27.94	3.05	0.44	6.90	0.00	0.00
ENSSSCG00000015270	FMOD	1183.75	3.05	0.09	34.62	0.00	0.00
ENSSSCG00000009490	DCT	4.67	3.05	1.10	2.79	0.01	0.01
ENSSSCG00000023693	PROC	91.72	3.05	0.25	12.22	0.00	0.00
ENSSSCG00000026559	EML6	34.30	3.06	0.41	7.43	0.00	0.00
ENSSSCG00000010272	ADAMTS14	352.82	3.07	0.13	22.89	0.00	0.00
ENSSSCG00000033707		3.14	3.07	1.33	2.31	0.02	0.03
ENSSSCG00000014878	PAK1	3.18	3.08	1.35	2.29	0.02	0.04
ENSSSCG00000002632	SLC28A1	7.97	3.09	0.83	3.71	0.00	0.00
ENSSSCG00000012521	GPRASP2	65.22	3.09	0.30	10.29	0.00	0.00
ENSSSCG00000009870	SDSL	38.28	3.09	0.39	8.03	0.00	0.00
ENSSSCG00000012852	CDHR5	12.77	3.09	0.68	4.58	0.00	0.00
ENSSSCG00000000634	STYK1	47.86	3.10	0.34	9.08	0.00	0.00
ENSSSCG00000002957	GGN	11.31	3.11	0.71	4.40	0.00	0.00
ENSSSCG00000034321	TP53INP1	554.69	3.12	0.11	27.52	0.00	0.00
ENSSSCG00000001910	ISLR	453.88	3.13	0.12	26.72	0.00	0.00

ENSSSCG00000029458	SLC16A2	11.39	3.13	0.70	4.49	0.00	0.00
ENSSSCG00000016613	AASS	1437.99	3.14	0.08	41.33	0.00	0.00
ENSSSCG00000040607	MAF	273.65	3.14	0.15	21.38	0.00	0.00
ENSSSCG00000024495		3.29	3.15	1.31	2.41	0.02	0.03
ENSSSCG00000037358		158.54	3.15	0.20	15.70	0.00	0.00
ENSSSCG00000030882		3.31	3.15	1.32	2.39	0.02	0.03
ENSSSCG00000000623	BCL2L14	8.29	3.16	0.83	3.80	0.00	0.00
ENSSSCG00000035284	BMF	535.00	3.16	0.11	28.48	0.00	0.00
ENSSSCG00000001470	SLA-DMA	56.85	3.17	0.32	9.91	0.00	0.00
ENSSSCG00000032490	LEAP2	13.38	3.17	0.65	4.87	0.00	0.00
ENSSSCG00000013408	ADM	496.94	3.18	0.12	25.78	0.00	0.00
ENSSSCG00000027710	PDZD3	209.85	3.18	0.17	18.60	0.00	0.00
ENSSSCG00000010992		59.14	3.19	0.32	9.85	0.00	0.00
ENSSSCG00000034068		108.20	3.19	0.24	13.54	0.00	0.00
ENSSSCG00000025393	SUSD2	570.04	3.19	0.11	28.78	0.00	0.00
ENSSSCG00000022804	RHOJ	1.81	3.20	1.61	1.99	0.05	0.07
ENSSSCG00000034532		1.81	3.20	1.61	1.99	0.05	0.07
ENSSSCG00000031367		277.72	3.20	0.15	21.75	0.00	0.00
ENSSSCG00000010210	SLC16A9	70.33	3.21	0.29	11.05	0.00	0.00
ENSSSCG00000023403		18.89	3.21	0.55	5.80	0.00	0.00
ENSSSCG00000012030	CLDN8	927.36	3.21	0.09	35.67	0.00	0.00
ENSSSCG00000016204	IHH	1.84	3.21	1.62	1.99	0.05	0.07
ENSSSCG00000015267	FMO2	5.15	3.22	1.08	2.98	0.00	0.01
ENSSSCG00000010426	PCDH15	5.18	3.22	1.08	2.99	0.00	0.01
ENSSSCG00000010431	A1CF	18.96	3.22	0.59	5.50	0.00	0.00

ENSSSCG00000010504	BLNK	1.85	3.22	1.64	1.97	0.05	0.07
ENSSSCG00000010056		708.27	3.22	0.10	31.64	0.00	0.00
ENSSSCG00000015894	DPP4	816.41	3.23	0.17	19.19	0.00	0.00
ENSSSCG00000003147	CA11	3.45	3.23	1.29	2.50	0.01	0.02
ENSSSCG00000030561	LMTK3	24.26	3.23	0.49	6.58	0.00	0.00
ENSSSCG00000013025	SLC22A12	3.46	3.23	1.32	2.45	0.01	0.02
ENSSSCG00000011397	SLC38A3	124.89	3.23	0.23	14.32	0.00	0.00
ENSSSCG00000005398		15.69	3.23	0.63	5.13	0.00	0.00
ENSSSCG00000009138		35.01	3.24	0.41	7.86	0.00	0.00
ENSSSCG00000031421	DYNLRB2	14.17	3.27	0.65	5.04	0.00	0.00
ENSSSCG00000027417	LDLRAD4	454.72	3.27	0.12	27.44	0.00	0.00
ENSSSCG00000004961	ITGA11	8.92	3.28	0.81	4.03	0.00	0.00
ENSSSCG00000029541	PSD2	8.93	3.28	0.81	4.04	0.00	0.00
ENSSSCG00000001040	C6orf52	21.57	3.28	0.53	6.15	0.00	0.00
ENSSSCG00000029275	PPARGC1A	1599.73	3.29	0.07	44.91	0.00	0.00
ENSSSCG00000014224	SEMA6A	2611.91	3.30	0.07	49.39	0.00	0.00
ENSSSCG00000022261		10.81	3.30	0.76	4.31	0.00	0.00
ENSSSCG00000006161	IL7	5.46	3.30	1.06	3.12	0.00	0.00
ENSSSCG00000006719		120.19	3.31	0.23	14.55	0.00	0.00
ENSSSCG00000017321	LYZL6	9.13	3.31	0.82	4.03	0.00	0.00
ENSSSCG00000013360	TMEM86A	23.73	3.31	0.51	6.54	0.00	0.00
ENSSSCG00000012520		12.84	3.31	0.71	4.63	0.00	0.00
ENSSSCG00000016857	DAB2	2684.32	3.32	0.06	51.38	0.00	0.00
ENSSSCG00000004402	METTL24	208.87	3.32	0.17	18.96	0.00	0.00
ENSSSCG00000028030	PCDHA13	3.66	3.32	1.30	2.56	0.01	0.02

ENSSSCG00000033193	TPO	3.69	3.32	1.31	2.54	0.01	0.02
ENSSSCG00000008819	TXK	1.97	3.33	1.59	2.10	0.04	0.06
ENSSSCG00000036732		1.97	3.33	1.59	2.10	0.04	0.06
ENSSSCG00000038300	ALDOB	170.30	3.33	0.20	16.83	0.00	0.00
ENSSSCG00000005852	ENTPD8	7.39	3.33	0.92	3.61	0.00	0.00
ENSSSCG00000037892	YPEL4	1.98	3.34	1.58	2.12	0.03	0.05
ENSSSCG00000013337	BBOX1	52.05	3.34	0.35	9.68	0.00	0.00
ENSSSCG00000002341	PAPLN	452.02	3.34	0.13	25.53	0.00	0.00
ENSSSCG00000034921	CLDN19	22.36	3.34	0.53	6.36	0.00	0.00
ENSSSCG00000009499		1.99	3.35	1.58	2.12	0.03	0.05
ENSSSCG00000035430	YPEL3	132.63	3.35	0.22	15.33	0.00	0.00
ENSSSCG00000003603	COL16A1	41.11	3.35	0.39	8.56	0.00	0.00
ENSSSCG00000023686	TTR	2.00	3.35	1.59	2.10	0.04	0.05
ENSSSCG00000017356	FAM171A2	151.62	3.35	0.21	16.13	0.00	0.00
ENSSSCG00000005278		97.51	3.35	0.26	13.11	0.00	0.00
ENSSSCG00000003619	AZIN2	5.63	3.35	1.04	3.22	0.00	0.00
ENSSSCG00000033264		2.01	3.35	1.59	2.11	0.04	0.05
ENSSSCG00000017129	RAB40B	24.43	3.36	0.53	6.36	0.00	0.00
ENSSSCG00000040771		3.75	3.36	1.30	2.59	0.01	0.02
ENSSSCG00000029558	EXTL1	37.88	3.37	0.41	8.30	0.00	0.00
ENSSSCG00000036572	BCO1	41.78	3.38	0.39	8.59	0.00	0.00
ENSSSCG00000017068	FAXDC2	17.33	3.40	0.60	5.68	0.00	0.00
ENSSSCG00000026718	PLCH1	25.08	3.40	0.50	6.74	0.00	0.00
ENSSSCG00000013296	SLC1A2	96.42	3.40	0.26	13.13	0.00	0.00
ENSSSCG00000028488	LTC4S	5.81	3.41	1.04	3.28	0.00	0.00

ENSSSCG00000005457		331.39	3.41	0.15	22.78	0.00	0.00
ENSSSCG00000010212		1359.31	3.41	0.08	41.43	0.00	0.00
ENSSSCG00000026387	REEP6	54.80	3.42	0.35	9.84	0.00	0.00
ENSSSCG00000016574	KCP	1413.22	3.43	0.07	45.75	0.00	0.00
ENSSSCG00000006836	KIAA1324	3.94	3.43	1.26	2.72	0.01	0.01
ENSSSCG00000015412	CCDC146	9.85	3.44	0.80	4.29	0.00	0.00
ENSSSCG00000033394	VWA3B	39.65	3.44	0.40	8.62	0.00	0.00
ENSSSCG00000003578	FGR	13.93	3.45	0.68	5.09	0.00	0.00
ENSSSCG00000003699	GREB1L	89.52	3.45	0.27	12.69	0.00	0.00
ENSSSCG00000022728	HOXC10	3.98	3.45	1.26	2.73	0.01	0.01
ENSSSCG00000002834		9.95	3.45	0.80	4.29	0.00	0.00
ENSSSCG00000025834	FNDC5	29.98	3.45	0.46	7.44	0.00	0.00
ENSSSCG00000029635	GRIN3B	2.16	3.47	1.56	2.22	0.03	0.04
ENSSSCG00000025294	AIRE	2.16	3.47	1.55	2.24	0.03	0.04
ENSSSCG00000009517	GPR183	42.60	3.48	0.40	8.75	0.00	0.00
ENSSSCG00000004617	FAM214A	367.37	3.48	0.14	24.67	0.00	0.00
ENSSSCG00000012637	KLHL13	111.03	3.50	0.24	14.34	0.00	0.00
ENSSSCG00000026412	PLXNA2	49.37	3.50	0.37	9.50	0.00	0.00
ENSSSCG00000022009	DDC	1056.41	3.51	0.09	38.39	0.00	0.00
ENSSSCG00000014561	NLRP6	4.19	3.52	1.26	2.79	0.01	0.01
ENSSSCG00000000875	NR1H4	234.40	3.53	0.17	20.53	0.00	0.00
ENSSSCG00000013864	TMEM38A	8.42	3.53	0.89	3.98	0.00	0.00
ENSSSCG00000003386	TNFRSF9	14.74	3.54	0.67	5.28	0.00	0.00
ENSSSCG00000024914		10.60	3.55	0.80	4.46	0.00	0.00
ENSSSCG00000000675	C1R	119.65	3.56	0.24	14.74	0.00	0.00

ENSSSCG00000036438	GPX3	1475.46	3.56	0.08	44.93	0.00	0.00
ENSSSCG00000006704		2.28	3.56	1.57	2.27	0.02	0.04
ENSSSCG00000021874	UNC5C	15.04	3.56	0.69	5.19	0.00	0.00
ENSSSCG00000007873	ABCC6	86.34	3.57	0.29	12.37	0.00	0.00
ENSSSCG00000015632		299.59	3.59	0.16	22.80	0.00	0.00
ENSSSCG00000032791		32.60	3.59	0.46	7.79	0.00	0.00
ENSSSCG00000008953	CXCL8	432.14	3.59	0.13	26.61	0.00	0.00
ENSSSCG00000017260	WIPI1	130.66	3.59	0.24	15.05	0.00	0.00
ENSSSCG00000016401		4.46	3.63	1.24	2.92	0.00	0.01
ENSSSCG00000023732	KLHDC7A	277.80	3.63	0.16	22.33	0.00	0.00
ENSSSCG00000003506	PINK1	368.54	3.64	0.15	24.71	0.00	0.00
ENSSSCG00000004332	BACH2	11.24	3.64	0.78	4.64	0.00	0.00
ENSSSCG00000003632		1.15	3.64	1.85	1.97	0.05	0.07
ENSSSCG00000024058	SLC5A2	256.81	3.64	0.18	20.76	0.00	0.00
ENSSSCG00000000760	LRTM2	1.15	3.64	1.83	1.99	0.05	0.07
ENSSSCG00000037735		15.80	3.64	0.67	5.47	0.00	0.00
ENSSSCG00000022948	SLC7A9	103.71	3.64	0.26	14.03	0.00	0.00
ENSSSCG00000004032	PRKN	1.15	3.64	1.85	1.97	0.05	0.07
ENSSSCG00000010177	TRIM67	1.15	3.64	1.85	1.97	0.05	0.07
ENSSSCG00000002919	TYROBP	1.16	3.65	1.86	1.97	0.05	0.07
ENSSSCG00000011801	HRG	1.16	3.65	1.86	1.97	0.05	0.07
ENSSSCG00000001523	GRM4	1.16	3.65	1.83	2.00	0.05	0.07
ENSSSCG00000010304	MYOZ1	1.16	3.65	1.83	2.00	0.05	0.07
ENSSSCG00000029516	SLC22A8	1.16	3.65	1.83	2.00	0.05	0.07
ENSSSCG00000036095	ACOT12	1.16	3.65	1.83	2.00	0.05	0.07

ENSSSCG00000030621		18.18	3.66	0.62	5.88	0.00	0.00
ENSSSCG00000023834	RSPH4A	11.40	3.66	0.80	4.60	0.00	0.00
ENSSSCG00000035396		22.78	3.66	0.55	6.62	0.00	0.00
ENSSSCG00000015119	MFRP	2.42	3.66	1.55	2.37	0.02	0.03
ENSSSCG00000003212	NAPSA	4.62	3.68	1.24	2.97	0.00	0.01
ENSSSCG00000000892	HAL	4.64	3.68	1.24	2.98	0.00	0.01
ENSSSCG00000015250	ADAMTS15	281.17	3.69	0.18	20.77	0.00	0.00
ENSSSCG00000004898	TNFRSF11A	2.49	3.69	1.51	2.45	0.01	0.02
ENSSSCG00000001107	SLC17A1	69.75	3.69	0.32	11.42	0.00	0.00
ENSSSCG00000022721	CDH16	579.59	3.70	0.13	28.64	0.00	0.00
ENSSSCG00000022989	ZNF704	39.92	3.71	0.43	8.71	0.00	0.00
ENSSSCG00000031141	ABCA13	21.11	3.71	0.58	6.37	0.00	0.00
ENSSSCG00000022390	RGN	11.79	3.71	0.78	4.75	0.00	0.00
ENSSSCG00000036645	PDE6B	30.68	3.71	0.48	7.66	0.00	0.00
ENSSSCG00000038296	SH3RF3	7.08	3.72	1.00	3.71	0.00	0.00
ENSSSCG00000027801	VTN	18.93	3.72	0.61	6.06	0.00	0.00
ENSSSCG00000033941	C2orf88	11.99	3.74	0.79	4.72	0.00	0.00
ENSSSCG00000009543	MYO16	4.78	3.74	1.23	3.04	0.00	0.00
ENSSSCG00000036011	ISLR2	21.64	3.74	0.58	6.49	0.00	0.00
ENSSSCG00000023279	SH3TC2	12.00	3.74	0.80	4.70	0.00	0.00
ENSSSCG00000002412	STON2	252.73	3.75	0.18	21.10	0.00	0.00
ENSSSCG00000002515	SLC25A47	4.83	3.75	1.24	3.03	0.00	0.00
ENSSSCG00000036158	TRAM1L1	4.85	3.75	1.24	3.02	0.00	0.00
ENSSSCG00000039921		4.83	3.75	1.24	3.04	0.00	0.00
ENSSSCG00000039626	DERL3	12.14	3.76	0.79	4.75	0.00	0.00

ENSSSCG00000028635		111.65	3.76	0.26	14.49	0.00	0.00
ENSSSCG00000024621	KAT2B	143.07	3.76	0.24	15.90	0.00	0.00
ENSSSCG00000028322	BTG2	97.17	3.76	0.28	13.41	0.00	0.00
ENSSSCG00000017261	ARSG	21.86	3.76	0.58	6.50	0.00	0.00
ENSSSCG00000012394	GJB1	1159.14	3.76	0.09	42.28	0.00	0.00
ENSSSCG00000020967		12.22	3.77	0.77	4.87	0.00	0.00
ENSSSCG00000027926	FTCD	213.80	3.78	0.19	19.88	0.00	0.00
ENSSSCG00000011201	SATB1	2.64	3.78	1.49	2.54	0.01	0.02
ENSSSCG00000028537		126.18	3.79	0.25	14.88	0.00	0.00
ENSSSCG00000022692	RINL	2.65	3.79	1.49	2.55	0.01	0.02
ENSSSCG00000013236	MYBPC3	4.98	3.79	1.23	3.08	0.00	0.00
ENSSSCG00000011239	TRANK1	146.81	3.80	0.23	16.34	0.00	0.00
ENSSSCG00000034524		2.67	3.80	1.49	2.54	0.01	0.02
ENSSSCG00000007346	ADIG	2.68	3.80	1.50	2.53	0.01	0.02
ENSSSCG00000037125	TMEM252	109.69	3.80	0.49	7.76	0.00	0.00
ENSSSCG00000037547	SLC17A3	180.18	3.80	0.21	18.10	0.00	0.00
ENSSSCG00000010546		105.16	3.80	0.28	13.68	0.00	0.00
ENSSSCG00000021227	HGD	15.14	3.81	0.72	5.33	0.00	0.00
ENSSSCG00000035911	P2RY8	1.31	3.83	1.77	2.16	0.03	0.05
ENSSSCG00000002921	CLIP3	12.70	3.83	0.77	4.95	0.00	0.00
ENSSSCG00000006693	PDZK1	4381.33	3.83	0.07	57.77	0.00	0.00
ENSSSCG00000002188		1.32	3.84	1.76	2.18	0.03	0.05
ENSSSCG00000017888	SLC13A5	1.32	3.84	1.76	2.18	0.03	0.05
ENSSSCG00000001045	ELOVL2	1.32	3.84	1.76	2.18	0.03	0.05
ENSSSCG00000003650	GJA9	1.33	3.85	1.76	2.19	0.03	0.05

ENSSSCG00000035912	ABCG5	1.33	3.85	1.76	2.19	0.03	0.05
ENSSSCG00000011160	ADARB2	1.33	3.85	1.77	2.18	0.03	0.05
ENSSSCG00000015653	IL19	1.34	3.85	1.80	2.15	0.03	0.05
ENSSSCG00000011495	PRICKLE2	95.84	3.86	0.28	13.54	0.00	0.00
ENSSSCG00000014812	FOLR2	196.98	3.86	0.20	19.16	0.00	0.00
ENSSSCG00000038476		28.56	3.86	0.52	7.45	0.00	0.00
ENSSSCG00000015293		2.77	3.86	1.52	2.54	0.01	0.02
ENSSSCG00000016829	AGXT2	2.78	3.87	1.50	2.59	0.01	0.02
ENSSSCG00000037446	INHBC	2.79	3.87	1.48	2.62	0.01	0.01
ENSSSCG00000031985		2.79	3.87	1.48	2.62	0.01	0.01
ENSSSCG00000039200		2.81	3.88	1.48	2.62	0.01	0.01
ENSSSCG00000029875	ZNF461	2.81	3.88	1.48	2.63	0.01	0.01
ENSSSCG00000006001	ENPP2	738.83	3.90	0.11	35.59	0.00	0.00
ENSSSCG00000001848	MESP2	124.18	3.92	0.25	15.51	0.00	0.00
ENSSSCG00000027320	SLC4A5	5.45	3.94	1.20	3.27	0.00	0.00
ENSSSCG00000024743	PRR15L	27.23	3.94	0.55	7.21	0.00	0.00
ENSSSCG00000031579	PCP4L1	35.44	3.94	0.47	8.30	0.00	0.00
ENSSSCG00000014251		24.86	3.96	0.57	6.97	0.00	0.00
ENSSSCG00000005250	APBA1	19.56	3.97	0.65	6.10	0.00	0.00
ENSSSCG00000039426	FSTL1	2.99	3.97	1.48	2.68	0.01	0.01
ENSSSCG00000033777	GPR152	3.00	3.98	1.46	2.72	0.01	0.01
ENSSSCG00000007678	COL26A1	3.02	3.98	1.47	2.71	0.01	0.01
ENSSSCG00000010068		177.50	3.99	0.22	18.14	0.00	0.00
ENSSSCG00000016010	CERKL	1.46	3.99	1.74	2.29	0.02	0.04
ENSSSCG00000009196	ATOH1	1.47	4.00	1.74	2.30	0.02	0.03

ENSSSCG00000007859	UMOD	1.47	4.00	1.71	2.34	0.02	0.03
ENSSSCG00000004502	RNF165	1.49	4.01	1.70	2.36	0.02	0.03
ENSSSCG00000037235		1.49	4.01	1.70	2.36	0.02	0.03
ENSSSCG00000012571		1.49	4.02	1.74	2.31	0.02	0.03
ENSSSCG00000023612		1.50	4.02	1.71	2.36	0.02	0.03
ENSSSCG00000021579		204.69	4.02	0.20	19.77	0.00	0.00
ENSSSCG00000001099	CMAH	1.50	4.02	1.72	2.34	0.02	0.03
ENSSSCG00000029513	TEX28	1.51	4.03	1.73	2.32	0.02	0.03
ENSSSCG00000016018	FRZB	1.52	4.03	1.73	2.33	0.02	0.03
ENSSSCG00000035739	SLC26A1	199.56	4.05	0.21	19.60	0.00	0.00
ENSSSCG00000009281	SGCG	11.78	4.05	0.84	4.82	0.00	0.00
ENSSSCG00000034623	ADAM2	3.29	4.12	1.46	2.82	0.00	0.01
ENSSSCG00000007507	PCK1	359.16	4.13	0.16	25.99	0.00	0.00
ENSSSCG00000034242	HNF4G	863.64	4.14	0.11	36.54	0.00	0.00
ENSSSCG00000003463	AGMAT	199.51	4.14	0.21	19.45	0.00	0.00
ENSSSCG00000009667		1.63	4.15	1.67	2.48	0.01	0.02
ENSSSCG00000011212	RARB	1.64	4.16	1.67	2.49	0.01	0.02
ENSSSCG00000040010	BCL2A1	1.65	4.16	1.67	2.50	0.01	0.02
ENSSSCG00000031610		1.66	4.17	1.66	2.51	0.01	0.02
ENSSSCG00000036820		1.66	4.17	1.66	2.51	0.01	0.02
ENSSSCG00000015926	ABCB11	19.04	4.17	0.69	6.05	0.00	0.00
ENSSSCG00000023322		1.67	4.18	1.66	2.51	0.01	0.02
ENSSSCG00000039945		1.68	4.19	1.69	2.48	0.01	0.02
ENSSSCG00000001753	CHRNB4	9.65	4.19	0.97	4.32	0.00	0.00
ENSSSCG00000004736	SPTBN5	25.71	4.19	0.59	7.10	0.00	0.00

ENSSSCG00000001411	APOM	29.02	4.19	0.56	7.53	0.00	0.00
ENSSSCG00000010639	HABP2	2385.53	4.20	0.17	24.39	0.00	0.00
ENSSSCG00000008124	NEURL3	210.23	4.20	0.21	19.87	0.00	0.00
ENSSSCG00000026067	TMPRSS6	22.71	4.20	0.63	6.62	0.00	0.00
ENSSSCG00000016652	LRRN3	181.80	4.20	0.22	18.74	0.00	0.00
ENSSSCG00000039858		3.48	4.21	1.42	2.96	0.00	0.01
ENSSSCG00000013150		199.10	4.24	0.22	19.57	0.00	0.00
ENSSSCG00000010948	CTSL	26.89	4.26	0.59	7.23	0.00	0.00
ENSSSCG00000040746	LRP2	23497.59	4.26	0.11	38.05	0.00	0.00
ENSSSCG00000015656	FCMR	6.73	4.26	1.17	3.63	0.00	0.00
ENSSSCG00000037416	CLIC5	47.32	4.27	0.44	9.62	0.00	0.00
ENSSSCG00000038171		10.23	4.28	0.95	4.49	0.00	0.00
ENSSSCG00000008214	FABP1	6.83	4.28	1.18	3.63	0.00	0.00
ENSSSCG00000010054	ADORA2A	13.76	4.29	0.83	5.18	0.00	0.00
ENSSSCG00000027849	CNR2	1.80	4.29	1.63	2.63	0.01	0.01
ENSSSCG00000026695	TNFSF14	1.82	4.30	1.63	2.63	0.01	0.01
ENSSSCG00000033073		1.82	4.30	1.64	2.63	0.01	0.01
ENSSSCG00000008121		20.84	4.31	0.67	6.40	0.00	0.00
ENSSSCG00000010332	PLAC9	1.83	4.31	1.64	2.63	0.01	0.01
ENSSSCG00000010600	CALHM2	1.84	4.31	1.63	2.65	0.01	0.01
ENSSSCG00000001746	PKHD1	2356.19	4.32	0.08	56.06	0.00	0.00
ENSSSCG00000002041	SLC7A7	985.17	4.33	0.11	40.60	0.00	0.00
ENSSSCG00000016270	C2orf72	119.98	4.33	0.29	14.98	0.00	0.00
ENSSSCG00000040554		21.21	4.33	0.67	6.44	0.00	0.00
ENSSSCG00000011561	PRRT3	3.82	4.34	1.41	3.08	0.00	0.00

ENSSSCG00000004291	NT5E	14.26	4.34	0.82	5.27	0.00	0.00
ENSSSCG00000037796		3.84	4.35	1.41	3.09	0.00	0.00
ENSSSCG00000024325	SGK2	234.58	4.37	0.21	20.92	0.00	0.00
ENSSSCG00000007710	MLXIPL	7.26	4.37	1.16	3.78	0.00	0.00
ENSSSCG00000009322	SLC46A3	145.24	4.37	0.26	16.65	0.00	0.00
ENSSSCG00000032282	ACP5	120.66	4.38	0.29	15.24	0.00	0.00
ENSSSCG00000015657	PIGR	594.17	4.39	0.14	32.30	0.00	0.00
ENSSSCG00000006148		14.74	4.39	0.82	5.33	0.00	0.00
ENSSSCG00000036122		1.95	4.41	1.63	2.70	0.01	0.01
ENSSSCG00000039027	TRO	1.96	4.41	1.61	2.74	0.01	0.01
ENSSSCG00000033532	SBK2	1.97	4.42	1.60	2.76	0.01	0.01
ENSSSCG00000014067	TMEM174	1.98	4.42	1.64	2.70	0.01	0.01
ENSSSCG00000004621	MYO5C	97.72	4.43	0.32	13.76	0.00	0.00
ENSSSCG00000001550	ARMC12	1.98	4.43	1.59	2.79	0.01	0.01
ENSSSCG00000034223		1.99	4.43	1.59	2.78	0.01	0.01
ENSSSCG00000024791		2.01	4.44	1.60	2.78	0.01	0.01
ENSSSCG00000025924	IGFBP5	285.15	4.46	0.20	22.78	0.00	0.00
ENSSSCG00000023936	MYRFL	4.18	4.48	1.40	3.19	0.00	0.00
ENSSSCG00000037740	RF02160	7.80	4.48	1.16	3.88	0.00	0.00
ENSSSCG00000032877		133.29	4.49	0.29	15.51	0.00	0.00
ENSSSCG00000011663	RBP2	1362.04	4.49	0.18	24.43	0.00	0.00
ENSSSCG00000002524	AMN	23.81	4.51	0.66	6.78	0.00	0.00
ENSSSCG00000011775	KLHL24	540.75	4.51	0.14	31.18	0.00	0.00
ENSSSCG00000033031		4.30	4.52	1.39	3.26	0.00	0.00
ENSSSCG00000027404	UNC93A	32.20	4.53	0.58	7.82	0.00	0.00

ENSSSCG00000004646	ATP8B4	145.79	4.54	0.27	16.69	0.00	0.00
ENSSSCG00000020884	GPR179	2.14	4.54	1.56	2.90	0.00	0.01
ENSSSCG00000015746	DLGAP2	2.15	4.54	1.61	2.81	0.00	0.01
ENSSSCG00000017131	FN3K	20.33	4.54	0.73	6.25	0.00	0.00
ENSSSCG00000033089		12.22	4.55	0.94	4.82	0.00	0.00
ENSSSCG00000012607		2.16	4.55	1.57	2.91	0.00	0.01
ENSSSCG00000034686	TAFA2	2.18	4.56	1.58	2.89	0.00	0.01
ENSSSCG00000031865	AQP8	12.56	4.59	0.93	4.93	0.00	0.00
ENSSSCG00000002333	DPF3	4.50	4.59	1.38	3.34	0.00	0.00
ENSSSCG00000033509	SAMD11	188.52	4.59	0.24	18.82	0.00	0.00
ENSSSCG00000003963		8.40	4.59	1.14	4.03	0.00	0.00
ENSSSCG00000008754	GBA3	277.73	4.62	0.21	22.52	0.00	0.00
ENSSSCG00000037535	SLC34A1	290.74	4.62	0.20	22.76	0.00	0.00
ENSSSCG00000011598	FGD5	12.83	4.62	0.93	4.97	0.00	0.00
ENSSSCG00000016473	EPHB6	2.31	4.65	1.54	3.01	0.00	0.00
ENSSSCG00000011107	CCNY	2.32	4.65	1.54	3.03	0.00	0.00
ENSSSCG00000011685	PAQR9	13.18	4.66	0.94	4.96	0.00	0.00
ENSSSCG00000016153	UNC80	2.34	4.66	1.54	3.02	0.00	0.00
ENSSSCG00000039548	PTGFR	128.83	4.68	0.31	15.23	0.00	0.00
ENSSSCG00000036649	FAM151A	13.44	4.69	0.93	5.05	0.00	0.00
ENSSSCG00000021938	UPP2	9.07	4.71	1.14	4.15	0.00	0.00
ENSSSCG00000033538	SMLR1	9.17	4.73	1.14	4.14	0.00	0.00
ENSSSCG00000004252		2.47	4.74	1.52	3.13	0.00	0.00
ENSSSCG00000024307	PRM2	2.48	4.75	1.52	3.13	0.00	0.00
ENSSSCG00000001863	TMEM266	2.49	4.76	1.52	3.14	0.00	0.00

ENSSSCG00000034087	TNFSF15	14.16	4.77	0.93	5.14	0.00	0.00
ENSSSCG00000036348	PCDHB15	5.12	4.79	1.35	3.55	0.00	0.00
ENSSSCG00000028896	DIO1	3471.16	4.83	0.07	65.45	0.00	0.00
ENSSSCG00000001698	CAPN11	2.61	4.83	1.51	3.20	0.00	0.00
ENSSSCG00000014438	PDE6A	2.62	4.83	1.51	3.21	0.00	0.00
ENSSSCG00000016247	TM4SF20	64.20	4.84	0.44	10.88	0.00	0.00
ENSSSCG00000015332	PON1	5.30	4.84	1.34	3.60	0.00	0.00
ENSSSCG00000039661	SSPO	2.64	4.84	1.52	3.18	0.00	0.00
ENSSSCG00000006141	CA3	2.65	4.84	1.49	3.24	0.00	0.00
ENSSSCG00000021483	NPBWR1	2.66	4.85	1.52	3.20	0.00	0.00
ENSSSCG00000037588		2.67	4.85	1.50	3.24	0.00	0.00
ENSSSCG00000028203		75.42	4.86	0.41	11.78	0.00	0.00
ENSSSCG00000015854	OCA2	25.18	4.86	0.71	6.84	0.00	0.00
ENSSSCG00000006238	CYP7A1	46.26	4.90	0.53	9.18	0.00	0.00
ENSSSCG00000016328	RAB17	20.61	4.90	0.80	6.14	0.00	0.00
ENSSSCG00000022592	FIBIN	103.33	4.90	0.36	13.72	0.00	0.00
ENSSSCG00000002033	CEBPE	2.75	4.91	1.52	3.23	0.00	0.00
ENSSSCG00000027300	TNP2	2.78	4.92	1.49	3.31	0.00	0.00
ENSSSCG00000029419	SCN11A	20.95	4.92	0.80	6.13	0.00	0.00
ENSSSCG00000035607	PRM3	2.81	4.93	1.48	3.33	0.00	0.00
ENSSSCG00000014108	BHMT	2.84	4.94	1.49	3.33	0.00	0.00
ENSSSCG00000006624	SELENBP1	344.50	4.94	0.20	24.61	0.00	0.00
ENSSSCG00000011640	TF	2760.75	4.96	0.18	28.33	0.00	0.00
ENSSSCG00000026453	ACSM5	70.00	4.97	0.44	11.31	0.00	0.00
ENSSSCG00000003006	CYP2B22	66.03	5.00	0.46	10.95	0.00	0.00

ENSSSCG00000031640		11.07	5.00	1.12	4.49	0.00	0.00
ENSSSCG00000040673	TMEM140	38.90	5.01	0.60	8.40	0.00	0.00
ENSSSCG00000016245		1486.33	5.01	0.11	47.14	0.00	0.00
ENSSSCG00000021656		3.12	5.08	1.45	3.50	0.00	0.00
ENSSSCG00000031174	FBP1	1143.45	5.13	0.13	39.79	0.00	0.00
ENSSSCG00000017797	SLC6A4	175.49	5.14	0.29	17.59	0.00	0.00
ENSSSCG00000016397	NEB	6.58	5.16	1.31	3.93	0.00	0.00
ENSSSCG00000031142		19.05	5.21	0.90	5.77	0.00	0.00
ENSSSCG00000015445	NRCAM	6.86	5.22	1.34	3.89	0.00	0.00
ENSSSCG00000016402	AGXT	25.63	5.22	0.78	6.69	0.00	0.00
ENSSSCG00000025273	CYP11A1	3.53	5.25	1.45	3.62	0.00	0.00
ENSSSCG00000039025	FREM1	101.27	5.30	0.41	13.02	0.00	0.00
ENSSSCG00000004672	GATM	872.92	5.30	0.15	34.96	0.00	0.00
ENSSSCG00000040088		122.14	5.31	0.37	14.43	0.00	0.00
ENSSSCG00000040465	KLRK1	3.76	5.36	1.42	3.77	0.00	0.00
ENSSSCG00000024051		3.78	5.36	1.41	3.81	0.00	0.00
ENSSSCG00000002012	CARMIL3	3.80	5.36	1.43	3.75	0.00	0.00
ENSSSCG00000017637	MPO	49.73	5.38	0.59	9.16	0.00	0.00
ENSSSCG00000029694	SLC23A3	35.94	5.39	0.69	7.80	0.00	0.00
ENSSSCG00000034639	MGAM	3.94	5.42	1.40	3.87	0.00	0.00
ENSSSCG00000039847	C1S	29.46	5.43	0.78	6.97	0.00	0.00
ENSSSCG00000025560	PGLYRP2	7.93	5.43	1.29	4.21	0.00	0.00
ENSSSCG00000013665		4.01	5.44	1.40	3.87	0.00	0.00
ENSSSCG00000002029	MYH7	8.12	5.47	1.29	4.25	0.00	0.00
ENSSSCG00000017254	MAP2K6	45.48	5.47	0.63	8.66	0.00	0.00

ENSSSCG00000009500	CLDN10	8.16	5.47	1.29	4.23	0.00	0.00
ENSSSCG00000004644	HDC	2749.72	5.48	0.09	58.99	0.00	0.00
ENSSSCG00000017763	SLC13A2	62.20	5.51	0.54	10.11	0.00	0.00
ENSSSCG00000023745	MCOLN3	15.89	5.54	1.09	5.09	0.00	0.00
ENSSSCG00000011973	COL8A1	40.09	5.55	0.69	8.04	0.00	0.00
ENSSSCG00000032607	ALPL	120.79	5.56	0.40	13.98	0.00	0.00
ENSSSCG00000024379	PCSK4	8.66	5.56	1.29	4.32	0.00	0.00
ENSSSCG00000020906	TNFSF10	477.30	5.57	0.21	26.91	0.00	0.00
ENSSSCG00000004835	MAGEL2	8.71	5.57	1.28	4.34	0.00	0.00
ENSSSCG00000010549		139.34	5.59	0.38	14.58	0.00	0.00
ENSSSCG00000013551	C3	4079.65	5.61	0.09	63.88	0.00	0.00
ENSSSCG00000002913	PRODH2	4.68	5.66	1.39	4.08	0.00	0.00
ENSSSCG00000032591		4.74	5.69	1.38	4.14	0.00	0.00
ENSSSCG00000014083	ANKDD1B	63.33	5.73	0.58	9.88	0.00	0.00
ENSSSCG00000009413	CPB2	103.89	5.79	0.46	12.53	0.00	0.00
ENSSSCG00000001849	ANPEP	1511.66	5.81	0.13	44.16	0.00	0.00
ENSSSCG00000039644	TRPM5	68.47	5.85	0.58	10.13	0.00	0.00
ENSSSCG00000038188		10.54	5.85	1.26	4.63	0.00	0.00
ENSSSCG00000000212	AQP6	5.43	5.88	1.34	4.38	0.00	0.00
ENSSSCG00000016609	SLC13A1	120.58	5.89	0.44	13.39	0.00	0.00
ENSSSCG00000003882	SLC5A9	5.60	5.93	1.34	4.44	0.00	0.00
ENSSSCG00000040105	SLC34A3	43.53	6.00	0.76	7.93	0.00	0.00
ENSSSCG00000010947	FBP2	11.72	6.01	1.26	4.77	0.00	0.00
ENSSSCG00000009942	DAO	746.94	6.02	0.19	31.76	0.00	0.00
ENSSSCG00000001908	CCDC33	6.11	6.05	1.33	4.54	0.00	0.00

ENSSSCG00000007858		6095.80	6.07	0.12	49.05	0.00	0.00
ENSSSCG00000039745	HMOX1	19784.22	6.09	0.05	116.77	0.00	0.00
ENSSSCG00000004057	SYTL3	6.44	6.13	1.32	4.65	0.00	0.00
ENSSSCG00000004643	SLC27A2	143.81	6.14	0.44	14.04	0.00	0.00
ENSSSCG00000037579	PPBP	13.11	6.17	1.25	4.94	0.00	0.00
ENSSSCG00000031111	CBR1	6.65	6.17	1.32	4.69	0.00	0.00
ENSSSCG00000011802	KNG1	6.66	6.17	1.33	4.65	0.00	0.00
ENSSSCG00000007909	ABAT	515.77	6.18	0.24	25.57	0.00	0.00
ENSSSCG00000017636	LPO	77.79	6.26	0.61	10.20	0.00	0.00
ENSSSCG00000026537	CACNA2D4	14.86	6.35	1.24	5.13	0.00	0.00
ENSSSCG00000029990	DEFB1	27.90	6.37	1.06	6.02	0.00	0.00
ENSSSCG00000035557		7.63	6.37	1.71	3.73	0.00	0.00
ENSSSCG00000001485	MLIP	15.10	6.38	1.24	5.14	0.00	0.00
ENSSSCG00000006147		86.75	6.42	0.61	10.44	0.00	0.00
ENSSSCG00000017762	FOXN1	7.93	6.43	1.29	4.96	0.00	0.00
ENSSSCG00000008312	DYSF	92.76	6.52	0.62	10.55	0.00	0.00
ENSSSCG00000002032	SLC7A8	369.21	6.70	0.33	20.52	0.00	0.00
ENSSSCG00000038221	HSD17B2	9.84	6.73	1.28	5.26	0.00	0.00
ENSSSCG00000007860	PDILT	19.36	6.74	1.23	5.50	0.00	0.00
ENSSSCG00000003846	GLIS1	54.16	6.74	0.86	7.86	0.00	0.00
ENSSSCG00000005488		19.56	6.75	1.23	5.51	0.00	0.00
ENSSSCG00000025141	PCDHGB1	10.50	6.83	1.27	5.36	0.00	0.00
ENSSSCG00000010055	GGT5	38.50	6.84	1.05	6.52	0.00	0.00
ENSSSCG00000040397	COLEC12	662.94	6.85	0.26	26.45	0.00	0.00
ENSSSCG00000018042	SLC47A2	40.19	6.90	1.05	6.58	0.00	0.00

ENSSSCG00000009805	HPD	21.97	6.92	1.22	5.67	0.00	0.00
ENSSSCG00000005125	LRRC19	41.35	6.94	1.05	6.62	0.00	0.00
ENSSSCG00000017781	PIPOX	12.05	7.03	1.25	5.61	0.00	0.00
ENSSSCG00000007857	ACSM3	626.62	7.28	0.30	23.95	0.00	0.00
ENSSSCG00000032923	PRND	15.36	7.38	1.24	5.96	0.00	0.00
ENSSSCG00000004040	SLC22A2	236.69	7.46	0.52	14.34	0.00	0.00
ENSSSCG00000009134	EGF	384.88	7.70	0.51	15.13	0.00	0.00
ENSSSCG00000011641	SLCO2A1	1293.30	7.78	0.25	31.16	0.00	0.00
ENSSSCG00000035602	GPR88	21.40	7.86	1.22	6.43	0.00	0.00
ENSSSCG00000014726	HBE1	26.58	8.17	1.22	6.71	0.00	0.00
ENSSSCG00000004017	FRMD1	106.63	8.31	1.03	8.05	0.00	0.00
ENSSSCG00000002720		31.40	8.41	1.21	6.94	0.00	0.00
ENSSSCG00000009486	SLITRK6	42.28	8.84	1.20	7.37	0.00	0.00
ENSSSCG00000010826	MARC2	47.26	9.00	1.20	7.51	0.00	0.00

Table S8. Differential gene expression analysis between untreated PK-15 cells and AFB_1-treated PK-15 cells by RNA-Seq.

**Gene names**	**Gene ID**	**baseMean**	**log2FoldChange**	**lfcSE**	**stat**	**pvalue**	**padj**
ENSSSCG00000034282	ABCA12	28.76	-7.35	1.22	-6.03	0.00	0.00
ENSSSCG00000012034	TIAM1	25.68	-7.18	1.22	-5.88	0.00	0.00
ENSSSCG00000038257	SGCZ	23.68	-7.06	1.23	-5.76	0.00	0.00
ENSSSCG00000000478	GRIP1	128.31	-7.02	0.61	-11.55	0.00	0.00
ENSSSCG00000009197	GRID2	8.27	-6.52	1.31	-4.96	0.00	0.00
ENSSSCG00000009228	MAPK10	7.94	-6.46	1.32	-4.90	0.00	0.00
ENSSSCG00000021132	PLD5	7.62	-6.40	1.32	-4.84	0.00	0.00

ENSSSCG00000010429	PRKG1	6.59	-6.19	1.34	-4.61	0.00	0.00
ENSSSCG00000029865	GRM5	12.16	-6.10	1.27	-4.81	0.00	0.00
ENSSSCG00000035058	PID1	5.92	-6.04	1.36	-4.44	0.00	0.00
ENSSSCG00000023181	ZNF385B	5.92	-6.04	1.36	-4.42	0.00	0.00
ENSSSCG00000032877		5.76	-6.00	1.37	-4.37	0.00	0.00
ENSSSCG00000039907		5.58	-5.95	1.37	-4.33	0.00	0.00
ENSSSCG00000011497	MAGI1	164.00	-5.95	0.38	-15.47	0.00	0.00
ENSSSCG00000024777	HDAC4	122.28	-5.94	0.44	-13.41	0.00	0.00
ENSSSCG00000033202		18.58	-5.81	1.09	-5.33	0.00	0.00
ENSSSCG00000004281	KCNQ5	138.97	-5.80	0.40	-14.52	0.00	0.00
ENSSSCG00000026129		35.48	-5.74	0.77	-7.41	0.00	0.00
ENSSSCG00000000854		4.73	-5.71	1.40	-4.08	0.00	0.00
ENSSSCG00000016123	PARD3B	210.94	-5.66	0.31	-18.10	0.00	0.00
ENSSSCG00000011821	ATP13A4	57.42	-5.62	0.59	-9.54	0.00	0.00
ENSSSCG00000031957		94.13	-5.55	0.45	-12.24	0.00	0.00
ENSSSCG00000033834	NDST4	8.10	-5.50	1.32	-4.18	0.00	0.00
ENSSSCG00000009537		4.07	-5.49	1.44	-3.80	0.00	0.00
ENSSSCG00000034351	B3GALT1	105.24	-5.49	0.42	-13.12	0.00	0.00
ENSSSCG00000016543	EXOC4	256.60	-5.46	0.27	-20.39	0.00	0.00
ENSSSCG00000023243	NFIA	338.69	-5.43	0.23	-23.31	0.00	0.00
ENSSSCG00000036181		3.88	-5.43	1.46	-3.73	0.00	0.00
ENSSSCG00000008758	SLC34A2	3.88	-5.43	1.50	-3.62	0.00	0.00
ENSSSCG00000034133		7.58	-5.40	1.32	-4.08	0.00	0.00
ENSSSCG00000012585	DCX	27.48	-5.36	0.79	-6.78	0.00	0.00
ENSSSCG00000037232	SNX29	54.01	-5.33	0.56	-9.48	0.00	0.00

ENSSSCG00000005194	PTPRD	396.11	-5.33	0.21	-25.48	0.00	0.00
ENSSSCG00000012709		13.20	-5.30	1.12	-4.73	0.00	0.00
ENSSSCG00000038766	MAD1L1	289.17	-5.26	0.24	-22.07	0.00	0.00
ENSSSCG00000015701	MGAT5	492.73	-5.20	0.18	-28.85	0.00	0.00
ENSSSCG00000011199	TBC1D5	473.10	-5.19	0.19	-28.06	0.00	0.00
ENSSSCG00000038505	MSI2	347.53	-5.18	0.21	-24.36	0.00	0.00
ENSSSCG00000009125	ANK2	556.54	-5.17	0.17	-30.43	0.00	0.00
ENSSSCG00000009693	XKR6	3.22	-5.16	1.51	-3.42	0.00	0.00
ENSSSCG00000005498	PAPPA	17.54	-5.12	0.92	-5.54	0.00	0.00
ENSSSCG00000004646	ATP8B4	6.25	-5.12	1.35	-3.79	0.00	0.00
ENSSSCG00000028805	PTPRM	304.54	-5.09	0.22	-22.82	0.00	0.00
ENSSSCG00000036553	SAMD12	80.34	-5.09	0.43	-11.89	0.00	0.00
ENSSSCG00000031193		3.04	-5.07	1.53	-3.31	0.00	0.00
ENSSSCG00000002684	CDH13	10.96	-5.03	1.13	-4.43	0.00	0.00
ENSSSCG00000013403	GALNT18	202.26	-5.02	0.27	-18.69	0.00	0.00
ENSSSCG00000005240	DOCK8	348.60	-5.01	0.21	-24.41	0.00	0.00
ENSSSCG00000011259	SCN5A	502.91	-5.01	0.17	-29.25	0.00	0.00
ENSSSCG00000028977	DOK6	10.83	-5.00	1.14	-4.39	0.00	0.00
ENSSSCG00000003777	SLC44A5	48.11	-4.99	0.54	-9.28	0.00	0.00
ENSSSCG00000037365		2.85	-4.99	1.65	-3.02	0.00	0.00
ENSSSCG00000035456	WWC1	2601.27	-4.98	0.08	-61.05	0.00	0.00
ENSSSCG00000009504	HS6ST3	20.94	-4.96	0.81	-6.12	0.00	0.00
ENSSSCG00000000837	CHST11	130.77	-4.96	0.33	-15.17	0.00	0.00
ENSSSCG00000016639	FOXP2	61.79	-4.94	0.47	-10.52	0.00	0.00
ENSSSCG00000001080	CDKAL1	112.21	-4.92	0.35	-14.04	0.00	0.00

ENSSSCG00000039334		15.00	-4.89	0.94	-5.18	0.00	0.00
ENSSSCG00000027417	LDLRAD4	44.71	-4.88	0.54	-9.01	0.00	0.00
ENSSSCG00000023924	PDE1A	279.14	-4.86	0.22	-22.21	0.00	0.00
ENSSSCG00000040093		5.23	-4.85	1.38	-3.52	0.00	0.00
ENSSSCG00000032200		5.23	-4.85	1.38	-3.52	0.00	0.00
ENSSSCG00000008444	PRKCE	150.36	-4.84	0.30	-16.41	0.00	0.00
ENSSSCG00000039025	FREM1	2.55	-4.82	1.63	-2.96	0.00	0.00
ENSSSCG00000027232		14.36	-4.82	0.94	-5.13	0.00	0.00
ENSSSCG00000016611	CADPS2	84.87	-4.80	0.39	-12.37	0.00	0.00
ENSSSCG00000011489		543.25	-4.80	0.16	-30.74	0.00	0.00
ENSSSCG00000011811	LPP	512.22	-4.80	0.16	-30.04	0.00	0.00
ENSSSCG00000008593	KLHL29	464.08	-4.79	0.17	-28.42	0.00	0.00
ENSSSCG00000027556		398.62	-4.79	0.18	-26.61	0.00	0.00
ENSSSCG00000006235	TOX	216.24	-4.77	0.39	-12.31	0.00	0.00
ENSSSCG00000011744		4.90	-4.75	1.40	-3.40	0.00	0.00
ENSSSCG00000035020	STK32B	72.96	-4.75	0.41	-11.45	0.00	0.00
ENSSSCG00000037754	SLC39A11	471.16	-4.74	0.16	-28.97	0.00	0.00
ENSSSCG00000030560	IGF1R	1619.52	-4.73	0.09	-50.58	0.00	0.00
ENSSSCG00000027074		154.38	-4.66	0.28	-16.78	0.00	0.00
ENSSSCG00000015519	RASAL2	440.21	-4.65	0.17	-28.04	0.00	0.00
ENSSSCG00000016656	ELMO1	190.31	-4.64	0.64	-7.25	0.00	0.00
ENSSSCG00000007056	PLCB1	850.55	-4.64	0.12	-38.19	0.00	0.00
ENSSSCG00000010329	ZMIZ1	274.88	-4.64	0.21	-22.35	0.00	0.00
ENSSSCG00000004490	SETBP1	338.73	-4.62	0.19	-24.60	0.00	0.00
ENSSSCG00000010204	BICC1	3654.13	-4.62	0.07	-70.61	0.00	0.00

ENSSSCG00000003773	AK5	124.38	-4.61	0.30	-15.21	0.00	0.00
ENSSSCG00000012474	DIAPH2	255.25	-4.60	0.22	-21.18	0.00	0.00
ENSSSCG00000009457	DACH1	351.97	-4.59	0.18	-25.10	0.00	0.00
ENSSSCG00000001083		4.39	-4.59	1.41	-3.25	0.00	0.00
ENSSSCG00000010814	ESRRG	20.25	-4.57	0.74	-6.18	0.00	0.00
ENSSSCG00000028759	ASAP1	612.23	-4.56	0.14	-32.90	0.00	0.00
ENSSSCG00000016317	AGAP1	472.33	-4.56	0.16	-29.08	0.00	0.00
ENSSSCG00000033118		4.24	-4.53	1.44	-3.15	0.00	0.00
ENSSSCG00000004064	ARID1B	528.81	-4.52	0.15	-30.67	0.00	0.00
ENSSSCG00000013294	LDLRAD3	46.92	-4.52	0.49	-9.27	0.00	0.00
ENSSSCG00000015462	TPK1	112.15	-4.50	0.31	-14.38	0.00	0.00
ENSSSCG00000016609	SLC13A1	2.04	-4.50	1.69	-2.66	0.01	0.01
ENSSSCG00000040746	LRP2	1234.35	-4.49	0.11	-41.80	0.00	0.00
ENSSSCG00000033650		2.03	-4.49	1.67	-2.69	0.01	0.01
ENSSSCG00000033765	PCDHGA3	2.03	-4.49	1.66	-2.71	0.01	0.01
ENSSSCG00000031830		41.68	-4.47	0.50	-8.88	0.00	0.00
ENSSSCG00000005457		30.18	-4.47	0.60	-7.50	0.00	0.00
ENSSSCG00000000801	TMEM117	94.34	-4.47	0.34	-13.26	0.00	0.00
ENSSSCG00000005585	DENND1A	493.49	-4.47	0.15	-29.77	0.00	0.00
ENSSSCG00000035424		11.31	-4.46	0.97	-4.60	0.00	0.00
ENSSSCG00000009060	MAML3	233.77	-4.44	0.21	-20.71	0.00	0.00
ENSSSCG00000038226		396.21	-4.44	0.33	-13.39	0.00	0.00
ENSSSCG00000006194	NCOA2	859.46	-4.41	0.11	-38.60	0.00	0.00
ENSSSCG00000033829	RIMS2	121.32	-4.38	0.29	-15.07	0.00	0.00
ENSSSCG00000034879	MAML2	280.32	-4.37	0.19	-22.50	0.00	0.00

ENSSSCG00000014434	IL17B	1.86	-4.37	1.70	-2.57	0.01	0.01
ENSSSCG00000008606	OSR1	17.71	-4.37	0.76	-5.73	0.00	0.00
ENSSSCG00000012724	SLITRK2	1.85	-4.36	1.74	-2.50	0.01	0.02
ENSSSCG00000037507	BTBD9	83.92	-4.35	0.35	-12.55	0.00	0.00
ENSSSCG00000033012		3.71	-4.33	1.46	-2.97	0.00	0.00
ENSSSCG00000033043	SHANK2	113.54	-4.33	0.30	-14.60	0.00	0.00
ENSSSCG00000014224	SEMA6A	256.38	-4.32	0.20	-21.51	0.00	0.00
ENSSSCG00000015307	CDK14	776.86	-4.31	0.12	-35.64	0.00	0.00
ENSSSCG00000039783		37.43	-4.31	0.51	-8.41	0.00	0.00
ENSSSCG00000033763	GNAQ	545.83	-4.30	0.14	-30.92	0.00	0.00
ENSSSCG00000022168	APBB2	1012.99	-4.30	0.10	-41.20	0.00	0.00
ENSSSCG00000010949	DAPK1	174.74	-4.29	0.24	-18.01	0.00	0.00
ENSSSCG00000029621	BMPR1B	339.14	-4.29	0.17	-25.03	0.00	0.00
ENSSSCG00000006071	VPS13B	747.43	-4.29	0.12	-36.03	0.00	0.00
ENSSSCG00000017274	PITPNC1	3.55	-4.27	1.46	-2.91	0.00	0.01
ENSSSCG00000037670	TMEM164	264.60	-4.27	0.19	-21.93	0.00	0.00
ENSSSCG00000010962	FRMD3	363.71	-4.25	0.17	-25.58	0.00	0.00
ENSSSCG00000004614	UNC13C	16.40	-4.25	0.76	-5.56	0.00	0.00
ENSSSCG00000026465	BABAM2	296.45	-4.24	0.18	-23.12	0.00	0.00
ENSSSCG00000021586	ZHX2	32.57	-4.24	0.54	-7.87	0.00	0.00
ENSSSCG00000039541	ANKRD11	1529.96	-4.24	0.23	-18.12	0.00	0.00
ENSSSCG00000023041	PCBP3	230.50	-4.24	0.20	-20.69	0.00	0.00
ENSSSCG00000031287		1.70	-4.23	1.75	-2.41	0.02	0.02
ENSSSCG00000022044		1.69	-4.23	1.75	-2.41	0.02	0.02
ENSSSCG00000035396		1.69	-4.23	1.75	-2.41	0.02	0.02

ENSSSCG00000006009	EXT1	3905.52	-4.23	0.06	-69.20	0.00	0.00
ENSSSCG00000023796		1.68	-4.23	1.76	-2.41	0.02	0.02
ENSSSCG00000011880	EAF2	1.68	-4.23	1.79	-2.36	0.02	0.03
ENSSSCG00000011485	PTPRG	2115.93	-4.22	0.07	-56.53	0.00	0.00
ENSSSCG00000008241	TCF7L1	105.43	-4.21	0.30	-14.07	0.00	0.00
ENSSSCG00000025598	COBLL1	130.61	-4.21	0.27	-15.60	0.00	0.00
ENSSSCG00000003747	KIAA1328	19.05	-4.21	0.71	-5.96	0.00	0.00
ENSSSCG00000005743	VAV2	814.55	-4.21	0.11	-37.50	0.00	0.00
ENSSSCG00000018681	ssc-mir-92a-1	3.38	-4.20	1.49	-2.81	0.00	0.01
ENSSSCG00000017694	ACACA	3107.26	-4.18	0.06	-65.20	0.00	0.00
ENSSSCG00000024152	ETV6	307.28	-4.17	0.18	-23.64	0.00	0.00
ENSSSCG00000021683		6.26	-4.17	1.22	-3.43	0.00	0.00
ENSSSCG00000011652	STAG1	589.28	-4.17	0.13	-31.32	0.00	0.00
ENSSSCG00000035414	XXYLT1	173.23	-4.17	0.23	-17.95	0.00	0.00
ENSSSCG00000024417	ERC2	12.33	-4.16	0.86	-4.85	0.00	0.00
ENSSSCG00000011416	DOCK3	9.11	-4.14	1.00	-4.16	0.00	0.00
ENSSSCG00000011103	PARD3	359.21	-4.14	0.16	-25.56	0.00	0.00
ENSSSCG00000000955	TBC1D22A	162.86	-4.13	0.24	-17.25	0.00	0.00
ENSSSCG00000012367	ZC3H12B	3.21	-4.11	1.50	-2.75	0.01	0.01
ENSSSCG00000027735		8.93	-4.11	1.00	-4.09	0.00	0.00
ENSSSCG00000006035	ANGPT1	5.89	-4.10	1.22	-3.35	0.00	0.00
ENSSSCG00000017268	PRKCA	980.49	-4.10	0.10	-40.90	0.00	0.00
ENSSSCG00000015329	PPP1R9A	324.36	-4.09	0.18	-23.30	0.00	0.00
ENSSSCG00000005180	BNC2	141.15	-4.09	0.25	-16.25	0.00	0.00
ENSSSCG00000015670	GTDC1	182.05	-4.09	0.22	-18.32	0.00	0.00

ENSSSCG00000031290	GRIN2A	283.90	-4.08	0.18	-22.80	0.00	0.00
ENSSSCG00000008903	KIAA1211	435.52	-4.08	0.15	-27.58	0.00	0.00
ENSSSCG00000038811	MOB3B	52.23	-4.07	0.43	-9.56	0.00	0.00
ENSSSCG00000016781	TRIO	3173.89	-4.06	0.06	-64.29	0.00	0.00
ENSSSCG00000036000		8.61	-4.05	1.00	-4.04	0.00	0.00
ENSSSCG00000025602	DOCK4	415.46	-4.05	0.15	-27.29	0.00	0.00
ENSSSCG00000011752	FNDC3B	1996.45	-4.04	0.08	-50.47	0.00	0.00
ENSSSCG00000001101	SCGN	212.35	-4.03	0.21	-19.34	0.00	0.00
ENSSSCG00000039317	SLC25A21	90.54	-4.03	0.31	-12.83	0.00	0.00
ENSSSCG00000035314		14.02	-4.01	0.78	-5.14	0.00	0.00
ENSSSCG00000008459	THADA	454.20	-4.01	0.14	-28.06	0.00	0.00
ENSSSCG00000015144	GRAMD1B	539.78	-4.00	0.13	-30.49	0.00	0.00
ENSSSCG00000017139	RPTOR	791.88	-4.00	0.11	-36.76	0.00	0.00
ENSSSCG00000017072	GALNT10	2447.32	-3.99	0.07	-59.30	0.00	0.00
ENSSSCG00000035949	FTO	192.46	-3.99	0.21	-18.79	0.00	0.00
ENSSSCG00000000555	ITPR2	579.07	-3.98	0.24	-16.88	0.00	0.00
ENSSSCG00000022177	DIS3L2	254.63	-3.98	0.18	-21.53	0.00	0.00
ENSSSCG00000028974	UST	84.16	-3.97	0.32	-12.55	0.00	0.00
ENSSSCG00000008310	EXOC6B	263.55	-3.97	0.18	-21.75	0.00	0.00
ENSSSCG00000002260		43.39	-3.96	0.44	-8.99	0.00	0.00
ENSSSCG00000006038	ZFPM2	78.12	-3.95	0.33	-12.07	0.00	0.00
ENSSSCG00000032532	CHRM2	85.88	-3.95	0.32	-12.50	0.00	0.00
ENSSSCG00000027161	PIP5K1B	2.88	-3.95	1.53	-2.58	0.01	0.01
ENSSSCG00000003828	FGGY	115.19	-3.94	0.28	-14.32	0.00	0.00
ENSSSCG00000002285	GPHN	275.62	-3.94	0.18	-22.37	0.00	0.00

ENSSSCG00000012104	SHROOM2	262.05	-3.94	0.18	-21.84	0.00	0.00
ENSSSCG00000016068	HECW2	187.04	-3.94	0.22	-18.14	0.00	0.00
ENSSSCG00000024146	MIR1249-1	2.86	-3.94	1.58	-2.50	0.01	0.02
ENSSSCG00000032321	MGAT4C	106.27	-3.93	0.29	-13.79	0.00	0.00
ENSSSCG00000024062		355.94	-3.92	0.16	-25.23	0.00	0.00
ENSSSCG00000011163	DIP2C	276.59	-3.92	0.18	-22.12	0.00	0.00
ENSSSCG00000038717	FARS2	99.86	-3.92	0.29	-13.65	0.00	0.00
ENSSSCG00000035621	GABRB1	81.29	-3.91	0.32	-12.22	0.00	0.00
ENSSSCG00000036751	PPM1H	1638.85	-3.91	0.08	-50.12	0.00	0.00
ENSSSCG00000016006		1.35	-3.91	1.86	-2.10	0.04	0.05
ENSSSCG00000029064		1.35	-3.91	1.87	-2.09	0.04	0.05
ENSSSCG00000030621		1.35	-3.91	1.90	-2.06	0.04	0.05
ENSSSCG00000035125		1.35	-3.91	1.86	-2.10	0.04	0.05
ENSSSCG00000003721	CHST9	1.35	-3.91	1.88	-2.07	0.04	0.05
ENSSSCG00000006911	TGFBR3	202.30	-3.90	0.20	-19.07	0.00	0.00
ENSSSCG00000005484	ZNF618	36.30	-3.89	0.48	-8.19	0.00	0.00
ENSSSCG00000004551	ZNF609	1023.68	-3.89	0.10	-40.32	0.00	0.00
ENSSSCG00000018084	ND3	4788.36	-3.88	0.18	-21.12	0.00	0.00
ENSSSCG00000002950	SIPA1L3	657.10	-3.87	0.12	-31.30	0.00	0.00
ENSSSCG00000035300		17.85	-3.87	0.68	-5.66	0.00	0.00
ENSSSCG00000015866	FMNL2	441.79	-3.86	0.14	-27.76	0.00	0.00
ENSSSCG00000007080		447.37	-3.86	0.14	-27.99	0.00	0.00
ENSSSCG00000028976	PPP2R2B	272.97	-3.85	0.18	-21.85	0.00	0.00
ENSSSCG00000012100		2.69	-3.85	1.58	-2.44	0.01	0.02
ENSSSCG00000015426	RELN	2.68	-3.85	1.58	-2.44	0.01	0.02

ENSSSCG00000008803	ATP8A1	102.53	-3.84	0.28	-13.75	0.00	0.00
ENSSSCG00000013339	ANO3	79.46	-3.83	0.32	-12.08	0.00	0.00
ENSSSCG00000003715	ZNF521	69.40	-3.82	0.35	-10.87	0.00	0.00
ENSSSCG00000012059	HLCS	645.18	-3.82	0.12	-32.18	0.00	0.00
ENSSSCG00000003877	FAF1	842.97	-3.81	0.10	-37.57	0.00	0.00
ENSSSCG00000009029	ARHGAP10	326.51	-3.81	0.16	-23.87	0.00	0.00
ENSSSCG00000002653		9.75	-3.81	0.90	-4.22	0.00	0.00
ENSSSCG00000011496	ADAMTS9	254.97	-3.79	0.18	-21.14	0.00	0.00
ENSSSCG00000036236	ELOVL6	1189.40	-3.78	0.09	-43.32	0.00	0.00
ENSSSCG00000010604	SH3PXD2A	571.82	-3.78	0.12	-30.22	0.00	0.00
ENSSSCG00000027669	TNS1	155.62	-3.77	0.22	-16.83	0.00	0.00
ENSSSCG00000017993	NTN1	546.55	-3.76	0.13	-29.06	0.00	0.00
ENSSSCG00000005278		9.44	-3.75	0.91	-4.14	0.00	0.00
ENSSSCG00000003848	LRP8	493.61	-3.75	0.13	-28.99	0.00	0.00
ENSSSCG00000039056		680.04	-3.75	0.11	-33.80	0.00	0.00
ENSSSCG00000036078		2.52	-3.75	1.58	-2.37	0.02	0.03
ENSSSCG00000008615	VSNL1	54.34	-3.75	0.38	-9.96	0.00	0.00
ENSSSCG00000030947	ZFAT	74.84	-3.74	0.32	-11.55	0.00	0.00
ENSSSCG00000010638	TCF7L2	343.10	-3.73	0.15	-24.74	0.00	0.00
ENSSSCG00000009953		277.92	-3.73	0.17	-22.14	0.00	0.00
ENSSSCG00000009364	FREM2	32.78	-3.73	0.50	-7.41	0.00	0.00
ENSSSCG00000036531		289.03	-3.73	0.16	-22.76	0.00	0.00
ENSSSCG00000001004	SLC22A23	482.00	-3.73	0.13	-28.92	0.00	0.00
ENSSSCG00000035794		11.64	-3.72	0.82	-4.54	0.00	0.00
ENSSSCG00000028905	TNIK	581.77	-3.70	0.12	-31.17	0.00	0.00

ENSSSCG00000002440	CCDC88C	343.10	-3.70	0.15	-24.31	0.00	0.00
ENSSSCG00000011230	OSBPL10	481.48	-3.70	0.13	-28.50	0.00	0.00
ENSSSCG00000007034	PSD3	139.25	-3.69	0.24	-15.43	0.00	0.00
ENSSSCG00000010915	NAV1	2078.11	-3.69	0.07	-53.26	0.00	0.00
ENSSSCG00000038958	DNM3	1670.70	-3.68	0.07	-49.74	0.00	0.00
ENSSSCG00000001100	CARMIL1	776.91	-3.67	0.10	-35.54	0.00	0.00
ENSSSCG00000031361	CELSR1	1758.73	-3.67	0.08	-48.64	0.00	0.00
ENSSSCG00000016784	ANKH	160.65	-3.66	0.22	-16.95	0.00	0.00
ENSSSCG00000002259		522.24	-3.66	0.13	-28.94	0.00	0.00
ENSSSCG00000008943	SLC4A4	923.12	-3.65	0.10	-36.99	0.00	0.00
ENSSSCG00000004980	THSD4	86.07	-3.65	0.30	-12.33	0.00	0.00
ENSSSCG00000011178	CPNE4	123.73	-3.65	0.25	-14.74	0.00	0.00
ENSSSCG00000026718	PLCH1	2.37	-3.65	1.59	-2.29	0.02	0.03
ENSSSCG00000036739	RF01225	4.37	-3.64	1.29	-2.81	0.00	0.01
ENSSSCG00000036926	MVB12B	193.61	-3.64	0.20	-18.51	0.00	0.00
ENSSSCG00000016034	COL3A1	258.59	-3.64	0.18	-20.40	0.00	0.00
ENSSSCG00000004506	CTIF	96.46	-3.63	0.28	-13.21	0.00	0.00
ENSSSCG00000031776	TULP4	111.81	-3.63	0.26	-14.21	0.00	0.00
ENSSSCG00000008642	ASAP2	927.94	-3.62	0.09	-38.32	0.00	0.00
ENSSSCG00000001975	PRKD1	60.91	-3.62	0.36	-10.19	0.00	0.00
ENSSSCG00000010272	ADAMTS14	41.18	-3.61	0.43	-8.41	0.00	0.00
ENSSSCG00000011056	FRMD4A	721.72	-3.61	0.10	-34.46	0.00	0.00
ENSSSCG00000015499		482.93	-3.61	0.13	-28.57	0.00	0.00
ENSSSCG00000015355	DGKB	36.61	-3.61	0.45	-7.93	0.00	0.00
ENSSSCG00000003733	KLHL14	641.93	-3.61	0.12	-31.28	0.00	0.00

ENSSSCG00000014223	COMMD10	491.23	-3.60	0.13	-28.17	0.00	0.00
ENSSSCG00000030359	ARHGEF3	169.17	-3.60	0.21	-17.25	0.00	0.00
ENSSSCG00000020736	ADD2	8.61	-3.60	0.92	-3.92	0.00	0.00
ENSSSCG00000006726	SPAG17	83.13	-3.59	0.29	-12.17	0.00	0.00
ENSSSCG00000002283	FUT8	407.02	-3.58	0.14	-26.21	0.00	0.00
ENSSSCG00000006978		186.20	-3.58	0.20	-18.04	0.00	0.00
ENSSSCG00000002257	MCTP2	188.14	-3.58	0.20	-18.15	0.00	0.00
ENSSSCG00000006321	FAM78B	10.65	-3.58	0.83	-4.33	0.00	0.00
ENSSSCG00000003663	HPCAL1	118.39	-3.58	0.25	-14.32	0.00	0.00
ENSSSCG00000028922	GABRA2	12.67	-3.57	0.75	-4.75	0.00	0.00
ENSSSCG00000009172	PPP3CA	351.40	-3.57	0.15	-24.16	0.00	0.00
ENSSSCG00000037535	SLC34A1	12.48	-3.55	0.75	-4.75	0.00	0.00
ENSSSCG00000005589	NR6A1	147.29	-3.55	0.22	-15.96	0.00	0.00
ENSSSCG00000027002	KLHL32	6.23	-3.55	1.07	-3.33	0.00	0.00
ENSSSCG00000026900	C15orf41	389.50	-3.55	0.14	-24.96	0.00	0.00
ENSSSCG00000016590	SND1	1598.36	-3.55	0.07	-47.34	0.00	0.00
ENSSSCG00000002457	ITPK1	506.77	-3.54	0.12	-28.87	0.00	0.00
ENSSSCG00000029811	PLCL2	6.24	-3.54	1.08	-3.28	0.00	0.00
ENSSSCG00000015645		477.24	-3.54	0.13	-28.24	0.00	0.00
ENSSSCG00000012026	ADAMTS1	2075.29	-3.54	0.07	-50.36	0.00	0.00
ENSSSCG00000008826	FRYL	492.02	-3.54	0.12	-28.33	0.00	0.00
ENSSSCG00000000614	GRIN2B	2.21	-3.53	1.63	-2.17	0.03	0.04
ENSSSCG00000039364		2.20	-3.53	1.61	-2.19	0.03	0.04
ENSSSCG00000004209	PTPRK	973.17	-3.53	0.09	-38.02	0.00	0.00
ENSSSCG00000009449	TDRD3	206.31	-3.52	0.19	-18.56	0.00	0.00

ENSSSCG00000005305	UNC13B	341.49	-3.52	0.15	-23.78	0.00	0.00
ENSSSCG00000038437	EHMT1	1029.60	-3.52	0.09	-39.99	0.00	0.00
ENSSSCG00000035153	TRIM38	10.16	-3.51	0.88	-3.97	0.00	0.00
ENSSSCG00000037125	TMEM252	8.07	-3.51	0.95	-3.69	0.00	0.00
ENSSSCG00000037119		14.19	-3.50	0.71	-4.92	0.00	0.00
ENSSSCG00000002752	ZFHX3	199.73	-3.50	0.19	-18.60	0.00	0.00
ENSSSCG00000034262	FIGN	320.59	-3.50	0.15	-23.01	0.00	0.00
ENSSSCG00000023133	OSBPL6	226.31	-3.50	0.18	-19.42	0.00	0.00
ENSSSCG00000016687		405.75	-3.49	0.14	-25.78	0.00	0.00
ENSSSCG00000010739	CTBP2	1042.37	-3.49	0.09	-39.72	0.00	0.00
ENSSSCG00000024232	CCDC91	245.97	-3.49	0.18	-19.75	0.00	0.00
ENSSSCG00000023437	ITPR1	903.76	-3.49	0.10	-36.47	0.00	0.00
ENSSSCG00000027935	FHOD3	35.69	-3.48	0.44	-7.87	0.00	0.00
ENSSSCG00000012121	EGFL6	15.82	-3.48	0.66	-5.27	0.00	0.00
ENSSSCG00000038397	PTK2	1381.98	-3.48	0.08	-43.93	0.00	0.00
ENSSSCG00000027415	WWOX	27.67	-3.47	0.50	-6.96	0.00	0.00
ENSSSCG00000005358		820.35	-3.46	0.10	-35.41	0.00	0.00
ENSSSCG00000006277	SPIDR	556.04	-3.46	0.12	-29.89	0.00	0.00
ENSSSCG00000026863	FARP1	1200.09	-3.46	0.08	-41.89	0.00	0.00
ENSSSCG00000039793	PATJ	1175.06	-3.46	0.08	-41.93	0.00	0.00
ENSSSCG00000016033	GULP1	27.31	-3.46	0.51	-6.74	0.00	0.00
ENSSSCG00000009831	CUX2	109.37	-3.45	0.25	-13.81	0.00	0.00
ENSSSCG00000015694	ZRANB3	286.13	-3.45	0.16	-21.51	0.00	0.00
ENSSSCG00000039868		3.88	-3.44	1.32	-2.61	0.01	0.01
ENSSSCG00000012663	ENOX2	291.55	-3.44	0.15	-22.25	0.00	0.00

ENSSSCG00000039594	SSBP3	396.77	-3.42	0.14	-25.08	0.00	0.00
ENSSSCG00000004829	CACHD1	752.39	-3.42	0.10	-34.40	0.00	0.00
ENSSSCG00000017168	SEPT9	3073.43	-3.42	0.06	-57.04	0.00	0.00
ENSSSCG00000018087	ND4	55032.34	-3.42	0.15	-22.46	0.00	0.00
ENSSSCG00000015846	RBPMS	1423.68	-3.42	0.08	-41.46	0.00	0.00
ENSSSCG00000000588	PLEKHA5	695.39	-3.42	0.10	-33.29	0.00	0.00
ENSSSCG00000031273	IMPG2	2.04	-3.41	1.68	-2.03	0.04	0.06
ENSSSCG00000016074	ANKRD44	94.85	-3.41	0.27	-12.65	0.00	0.00
ENSSSCG00000020173	RF00427	2.02	-3.40	1.65	-2.06	0.04	0.05
ENSSSCG00000022361		2.01	-3.40	1.66	-2.05	0.04	0.06
ENSSSCG00000002689	CMIP	600.94	-3.40	0.11	-30.01	0.00	0.00
ENSSSCG00000015326	COL1A2	835.78	-3.39	0.10	-34.85	0.00	0.00
ENSSSCG00000012164	CNKSR2	217.46	-3.39	0.18	-18.85	0.00	0.00
ENSSSCG00000023709	PTPRJ	3229.96	-3.39	0.06	-56.05	0.00	0.00
ENSSSCG00000011226	TGFBR2	7904.45	-3.38	0.04	-75.53	0.00	0.00
ENSSSCG00000023627	LYPD6	33.52	-3.38	0.44	-7.65	0.00	0.00
ENSSSCG00000013409	SBF2	750.83	-3.38	0.10	-33.25	0.00	0.00
ENSSSCG00000038138	TFEC	5.54	-3.38	1.09	-3.09	0.00	0.00
ENSSSCG00000015707	GPR39	195.14	-3.38	0.19	-18.04	0.00	0.00
ENSSSCG00000039144		81.64	-3.38	0.29	-11.68	0.00	0.00
ENSSSCG00000010732	FAM53B	126.34	-3.37	0.23	-14.60	0.00	0.00
ENSSSCG00000011760	TBL1XR1	2539.95	-3.37	0.08	-44.63	0.00	0.00
ENSSSCG00000012102		96.04	-3.36	0.47	-7.10	0.00	0.00
ENSSSCG00000017826	SMG6	537.78	-3.36	0.12	-29.16	0.00	0.00
ENSSSCG00000038452	ADAMTS17	60.93	-3.36	0.34	-9.90	0.00	0.00

ENSSSCG00000015878	PKP4	915.08	-3.35	0.09	-37.24	0.00	0.00
ENSSSCG00000011619	EEFSEC	199.13	-3.35	0.19	-17.79	0.00	0.00
ENSSSCG00000036499	LMO3	10.95	-3.35	0.76	-4.38	0.00	0.00
ENSSSCG00000039597	RF02168	20.06	-3.35	0.57	-5.90	0.00	0.00
ENSSSCG00000038164	RAI1	705.86	-3.34	0.10	-33.07	0.00	0.00
ENSSSCG00000002332	SIPA1L1	464.11	-3.34	0.12	-26.85	0.00	0.00
ENSSSCG00000000755	ERC1	1090.34	-3.33	0.08	-40.05	0.00	0.00
ENSSSCG00000040272	TTC29	79.18	-3.33	0.29	-11.43	0.00	0.00
ENSSSCG00000024136	AMPH	44.80	-3.32	0.38	-8.75	0.00	0.00
ENSSSCG00000011079	PIP4K2A	437.00	-3.32	0.13	-26.24	0.00	0.00
ENSSSCG00000035027	ZDHHC14	390.22	-3.32	0.14	-24.30	0.00	0.00
ENSSSCG00000009101	PRDM5	189.46	-3.31	0.19	-17.52	0.00	0.00
ENSSSCG00000008504	CRIM1	2745.42	-3.31	0.06	-53.97	0.00	0.00
ENSSSCG00000003486	ARHGEF10L	394.91	-3.31	0.14	-23.59	0.00	0.00
ENSSSCG00000024754	BBS9	74.61	-3.30	0.29	-11.25	0.00	0.00
ENSSSCG00000006878	DPYD	144.04	-3.30	0.22	-15.34	0.00	0.00
ENSSSCG00000005934	TRAPPC9	399.27	-3.30	0.13	-25.18	0.00	0.00
ENSSSCG00000024960	PDGFC	431.07	-3.30	0.13	-25.78	0.00	0.00
ENSSSCG00000009348	STARD13	453.78	-3.30	0.12	-26.64	0.00	0.00
ENSSSCG00000027608	FAM172A	74.46	-3.30	0.29	-11.21	0.00	0.00
ENSSSCG00000014203	MCC	180.88	-3.30	0.19	-16.94	0.00	0.00
ENSSSCG00000040742		7.06	-3.30	0.95	-3.49	0.00	0.00
ENSSSCG00000009584	SEMA4D	412.31	-3.29	0.13	-25.72	0.00	0.00
ENSSSCG00000033521		105.48	-3.29	0.25	-13.26	0.00	0.00
ENSSSCG00000018086	ND4L	2035.18	-3.27	0.08	-39.40	0.00	0.00

ENSSSCG00000036168	PDSS2	341.61	-3.27	0.14	-22.62	0.00	0.00
ENSSSCG00000005751	COL5A1	2500.46	-3.26	0.06	-54.58	0.00	0.00
ENSSSCG00000016513	KIAA1549	218.34	-3.25	0.18	-18.55	0.00	0.00
ENSSSCG00000034348	NBEA	228.79	-3.25	0.17	-19.22	0.00	0.00
ENSSSCG00000009112	SEC24D	563.19	-3.25	0.11	-28.73	0.00	0.00
ENSSSCG00000010900	DENND1B	373.97	-3.25	0.13	-24.37	0.00	0.00
ENSSSCG00000023001	CCDC149	402.97	-3.25	0.13	-24.98	0.00	0.00
ENSSSCG00000004600	TCF12	838.41	-3.24	0.09	-35.19	0.00	0.00
ENSSSCG00000008447	CAMKMT	186.33	-3.24	0.19	-17.42	0.00	0.00
ENSSSCG00000009864	MED13L	1424.30	-3.24	0.08	-41.94	0.00	0.00
ENSSSCG00000006018	TRPS1	266.37	-3.24	0.16	-20.47	0.00	0.00
ENSSSCG00000003839		1394.69	-3.24	0.09	-37.95	0.00	0.00
ENSSSCG00000013331	METTL15	100.49	-3.24	0.25	-12.80	0.00	0.00
ENSSSCG00000015631	GRB10	1141.53	-3.24	0.09	-37.83	0.00	0.00
ENSSSCG00000010755	PTPRE	312.84	-3.23	0.14	-22.44	0.00	0.00
ENSSSCG00000015880	TANC1	971.75	-3.23	0.09	-37.38	0.00	0.00
ENSSSCG00000039780	RTN4RL1	1302.12	-3.22	0.08	-41.17	0.00	0.00
ENSSSCG00000014213	KCNN2	68.32	-3.21	0.30	-10.56	0.00	0.00
ENSSSCG00000015399	SEMA3E	1427.61	-3.21	0.08	-42.57	0.00	0.00
ENSSSCG00000024392	THEMIS	5.09	-3.20	1.15	-2.78	0.01	0.01
ENSSSCG00000009230	WDFY3	1367.30	-3.20	0.08	-41.65	0.00	0.00
ENSSSCG00000011030	CUBN	78.36	-3.20	0.29	-10.95	0.00	0.00
ENSSSCG00000003736	ASXL3	214.01	-3.19	0.17	-18.46	0.00	0.00
ENSSSCG00000022240	POLA1	519.32	-3.19	0.12	-27.41	0.00	0.00
ENSSSCG00000008512	TTC27	571.59	-3.18	0.11	-29.13	0.00	0.00

ENSSSCG00000032968	VTI1A	123.34	-3.18	0.23	-13.89	0.00	0.00
ENSSSCG00000026733	HIPK2	781.16	-3.18	0.10	-33.41	0.00	0.00
ENSSSCG00000025130	KLF12	112.77	-3.17	0.24	-13.45	0.00	0.00
ENSSSCG00000015607	HHAT	36.06	-3.17	0.41	-7.65	0.00	0.00
ENSSSCG00000009007	TMEM131L	447.74	-3.17	0.13	-25.34	0.00	0.00
ENSSSCG00000022034	SMYD3	53.92	-3.17	0.34	-9.44	0.00	0.00
ENSSSCG00000011970	CMSS1	451.79	-3.17	0.12	-26.40	0.00	0.00
ENSSSCG00000039157	CLASP1	1066.49	-3.17	0.09	-35.13	0.00	0.00
ENSSSCG00000018092	ND6	745.92	-3.17	0.27	-11.88	0.00	0.00
ENSSSCG00000005178	CNTLN	374.75	-3.17	0.14	-22.81	0.00	0.00
ENSSSCG00000016976	ZNF366	4.89	-3.17	1.10	-2.87	0.00	0.01
ENSSSCG00000031118	PREX1	693.52	-3.16	0.10	-31.42	0.00	0.00
ENSSSCG00000000075	MRTFA	661.15	-3.16	0.10	-31.28	0.00	0.00
ENSSSCG00000009781	PITPNM2	746.99	-3.15	0.10	-32.76	0.00	0.00
ENSSSCG00000015770	VEGFC	61.31	-3.15	0.31	-10.02	0.00	0.00
ENSSSCG00000032549	GNA12	467.70	-3.15	0.12	-26.53	0.00	0.00
ENSSSCG00000009448	DIAPH3	398.36	-3.15	0.13	-24.06	0.00	0.00
ENSSSCG00000034191	SOX6	315.67	-3.15	0.14	-21.86	0.00	0.00
ENSSSCG00000034059	ST3GAL3	74.16	-3.15	0.29	-10.93	0.00	0.00
ENSSSCG00000011209		246.98	-3.14	0.16	-19.72	0.00	0.00
ENSSSCG00000018067		45.03	-3.14	0.37	-8.50	0.00	0.00
ENSSSCG00000015780	STOX2	339.83	-3.13	0.14	-22.57	0.00	0.00
ENSSSCG00000005182	CCDC171	6.41	-3.13	0.97	-3.23	0.00	0.00
ENSSSCG00000002241	NUTM1	3.20	-3.13	1.36	-2.30	0.02	0.03
ENSSSCG00000022989	ZNF704	3.20	-3.13	1.36	-2.30	0.02	0.03

ENSSSCG00000009503		22.22	-3.13	0.52	-5.96	0.00	0.00
ENSSSCG00000012309	SHROOM4	407.29	-3.13	0.13	-24.09	0.00	0.00
ENSSSCG00000004948	SMAD6	128.18	-3.12	0.22	-13.89	0.00	0.00
ENSSSCG00000011070	MPP7	68.41	-3.12	0.30	-10.26	0.00	0.00
ENSSSCG00000039952	ZFAND3	510.83	-3.12	0.11	-27.53	0.00	0.00
ENSSSCG00000004511		1789.91	-3.12	0.07	-47.16	0.00	0.00
ENSSSCG00000008171	NPAS2	256.92	-3.12	0.16	-19.49	0.00	0.00
ENSSSCG00000004250	SLC35F1	69.82	-3.12	0.30	-10.37	0.00	0.00
ENSSSCG00000036008	TACR1	118.57	-3.12	0.23	-13.71	0.00	0.00
ENSSSCG00000008621	NBAS	465.01	-3.11	0.12	-26.67	0.00	0.00
ENSSSCG00000011906	IGSF11	205.95	-3.11	0.18	-17.30	0.00	0.00
ENSSSCG00000033537		350.71	-3.11	0.14	-22.70	0.00	0.00
ENSSSCG00000023520	PCSK5	130.73	-3.11	0.22	-14.01	0.00	0.00
ENSSSCG00000038196		40.74	-3.11	0.38	-8.15	0.00	0.00
ENSSSCG00000004538	WDR7	436.26	-3.11	0.12	-25.53	0.00	0.00
ENSSSCG00000040208	PAG1	595.12	-3.10	0.11	-29.33	0.00	0.00
ENSSSCG00000029474		256.14	-3.10	0.16	-19.38	0.00	0.00
ENSSSCG00000023434	PPM1L	64.02	-3.10	0.31	-10.08	0.00	0.00
ENSSSCG00000030102	RF00568	18.68	-3.10	0.57	-5.45	0.00	0.00
ENSSSCG00000040875	ZFPM1	148.22	-3.10	0.20	-15.19	0.00	0.00
ENSSSCG00000033136		24.88	-3.10	0.49	-6.32	0.00	0.00
ENSSSCG00000009489		12.47	-3.09	0.68	-4.52	0.00	0.00
ENSSSCG00000022496	STK39	540.33	-3.09	0.11	-27.30	0.00	0.00
ENSSSCG00000000941	METTL25	35.69	-3.09	0.41	-7.55	0.00	0.00
ENSSSCG00000016665	BMPER	297.05	-3.08	0.15	-21.03	0.00	0.00

ENSSSCG00000034993	NREP	2420.28	-3.08	0.07	-43.78	0.00	0.00
ENSSSCG00000008698	RGS12	81.64	-3.08	0.27	-11.34	0.00	0.00
ENSSSCG00000012001	ROBO1	1259.43	-3.07	0.08	-38.71	0.00	0.00
ENSSSCG00000005177	SH3GL2	438.23	-3.07	0.12	-25.70	0.00	0.00
ENSSSCG00000014437	PPARGC1B	230.83	-3.06	0.16	-18.87	0.00	0.00
ENSSSCG00000002494	CLMN	202.62	-3.06	0.17	-17.66	0.00	0.00
ENSSSCG00000010650	AFAP1L2	144.66	-3.05	0.21	-14.59	0.00	0.00
ENSSSCG00000026146	GALNT5	7.58	-3.05	0.87	-3.50	0.00	0.00
ENSSSCG00000023078	WDR4	405.81	-3.05	0.12	-24.67	0.00	0.00
ENSSSCG00000006231	CHD7	1079.89	-3.05	0.09	-33.72	0.00	0.00
ENSSSCG00000014891	GAB2	3.04	-3.05	1.38	-2.21	0.03	0.04
ENSSSCG00000015368	HDAC9	1774.60	-3.03	0.07	-45.63	0.00	0.00
ENSSSCG00000001709	SUPT3H	500.14	-3.03	0.11	-26.54	0.00	0.00
ENSSSCG00000022112	DOCK9	1427.07	-3.03	0.07	-41.70	0.00	0.00
ENSSSCG00000005997	COL14A1	80.52	-3.03	0.28	-10.95	0.00	0.00
ENSSSCG00000033185	PPP2R3A	340.60	-3.03	0.14	-22.10	0.00	0.00
ENSSSCG00000029392	HUNK	339.55	-3.01	0.14	-21.84	0.00	0.00
ENSSSCG00000013399	TEAD1	1298.56	-3.01	0.08	-39.39	0.00	0.00
ENSSSCG00000005587	NEK6	1090.45	-3.01	0.08	-37.54	0.00	0.00
ENSSSCG00000008372	EHBP1	396.70	-3.01	0.13	-23.86	0.00	0.00
ENSSSCG00000000783	SLC2A13	236.87	-3.01	0.16	-18.63	0.00	0.00
ENSSSCG00000031875	ZNF469	339.76	-3.01	0.14	-22.20	0.00	0.00
ENSSSCG00000011499	LRIG1	107.76	-3.01	0.23	-12.90	0.00	0.00
ENSSSCG00000023784	SEMA3C	3356.60	-3.00	0.06	-50.13	0.00	0.00
ENSSSCG00000015667	MBD5	61.64	-3.00	0.31	-9.74	0.00	0.00

ENSSSCG00000015396	SEMA3D	2156.69	-3.00	0.07	-43.75	0.00	0.00
ENSSSCG00000001065	ATXN1	246.44	-2.99	0.16	-19.00	0.00	0.00
ENSSSCG00000038058	RAD51AP2	5.91	-2.99	1.00	-3.00	0.00	0.00
ENSSSCG00000016650		10.29	-2.99	0.75	-3.97	0.00	0.00
ENSSSCG00000011599	GRIP2	401.78	-2.99	0.13	-23.68	0.00	0.00
ENSSSCG00000003690	DLGAP1	193.65	-2.99	0.18	-16.82	0.00	0.00
ENSSSCG00000028979	UVRAG	234.13	-2.98	0.17	-17.80	0.00	0.00
ENSSSCG00000005487	COL27A1	107.54	-2.98	0.24	-12.64	0.00	0.00
ENSSSCG00000002429	FOXN3	148.21	-2.98	0.20	-14.71	0.00	0.00
ENSSSCG00000035757	SSBP2	122.94	-2.98	0.23	-12.77	0.00	0.00
ENSSSCG00000008434	TTC7A	827.64	-2.98	0.09	-32.24	0.00	0.00
ENSSSCG00000003731	GAREM1	230.24	-2.98	0.16	-18.44	0.00	0.00
ENSSSCG00000015011		944.53	-2.97	0.09	-33.60	0.00	0.00
ENSSSCG00000036872		12.95	-2.97	0.68	-4.40	0.00	0.00
ENSSSCG00000038684		14.46	-2.97	0.63	-4.71	0.00	0.00
ENSSSCG00000004037	MAP3K4	480.90	-2.97	0.12	-25.66	0.00	0.00
ENSSSCG00000010494	SORBS1	947.14	-2.97	0.09	-32.87	0.00	0.00
ENSSSCG00000028877	RAD51B	63.26	-2.97	0.30	-9.84	0.00	0.00
ENSSSCG00000037556	COG5	534.43	-2.96	0.11	-27.23	0.00	0.00
ENSSSCG00000005481		97.59	-2.96	0.25	-12.04	0.00	0.00
ENSSSCG00000004415		30.16	-2.96	0.44	-6.78	0.00	0.00
ENSSSCG00000005465	SUSD1	170.35	-2.96	0.18	-16.11	0.00	0.00
ENSSSCG00000020717	FAM160A1	138.73	-2.96	0.21	-14.29	0.00	0.00
ENSSSCG00000023408	SAMD4A	908.85	-2.96	0.09	-34.28	0.00	0.00
ENSSSCG00000036033	THRB	5.71	-2.96	1.02	-2.91	0.00	0.01

ENSSSCG00000010765	INPP5A	49.76	-2.95	0.34	-8.64	0.00	0.00
ENSSSCG00000016894	ARL15	19.85	-2.95	0.53	-5.54	0.00	0.00
ENSSSCG00000013335	LGR4	720.73	-2.94	0.10	-29.65	0.00	0.00
ENSSSCG00000005095		363.91	-2.94	0.13	-22.34	0.00	0.00
ENSSSCG00000014792	STIM1	376.40	-2.94	0.13	-21.86	0.00	0.00
ENSSSCG00000006900	DIPK1A	407.39	-2.94	0.13	-23.44	0.00	0.00
ENSSSCG00000014136	VCAN	99.99	-2.93	0.24	-12.32	0.00	0.00
ENSSSCG00000010185	GALNT2	1132.75	-2.93	0.08	-36.49	0.00	0.00
ENSSSCG00000020984	LXN	12.61	-2.93	0.67	-4.35	0.00	0.00
ENSSSCG00000004123	UTRN	1653.37	-2.92	0.08	-38.78	0.00	0.00
ENSSSCG00000005720	MED27	174.34	-2.91	0.19	-15.66	0.00	0.00
ENSSSCG00000008881	RAPGEF2	483.33	-2.91	0.11	-25.70	0.00	0.00
ENSSSCG00000014580	RIC3	52.63	-2.91	0.33	-8.81	0.00	0.00
ENSSSCG00000030274	PPEF1	113.91	-2.90	0.23	-12.76	0.00	0.00
ENSSSCG00000007098		1036.28	-2.90	0.08	-35.23	0.00	0.00
ENSSSCG00000029169	MAPKAP1	733.89	-2.90	0.10	-29.78	0.00	0.00
ENSSSCG00000009021	LRBA	856.49	-2.90	0.09	-32.29	0.00	0.00
ENSSSCG00000003684	MTCL1	822.42	-2.90	0.09	-33.15	0.00	0.00
ENSSSCG00000007477	NFATC2	911.80	-2.89	0.09	-32.79	0.00	0.00
ENSSSCG00000022636	DENND5B	374.87	-2.89	0.14	-21.10	0.00	0.00
ENSSSCG00000040944	SKI	1303.73	-2.89	0.08	-36.73	0.00	0.00
ENSSSCG00000007066	SLX4IP	65.79	-2.89	0.29	-9.93	0.00	0.00
ENSSSCG00000026506	RALGAPA2	489.52	-2.89	0.11	-25.53	0.00	0.00
ENSSSCG00000039084		75.23	-2.88	0.27	-10.59	0.00	0.00
ENSSSCG00000023084	ATP2B2	10.94	-2.88	0.71	-4.08	0.00	0.00

ENSSSCG00000015402	CACNA2D1	2048.25	-2.88	0.06	-46.04	0.00	0.00
ENSSSCG00000028720	NXN	783.89	-2.88	0.09	-31.88	0.00	0.00
ENSSSCG00000029745	FAM193A	302.89	-2.88	0.15	-19.80	0.00	0.00
ENSSSCG00000014976	ARHGAP42	761.45	-2.87	0.09	-30.90	0.00	0.00
ENSSSCG00000010556	PAX2	868.44	-2.87	0.09	-32.13	0.00	0.00
ENSSSCG00000026655	PTPN13	2500.61	-2.87	0.06	-47.32	0.00	0.00
ENSSSCG00000037321		4.02	-2.87	1.23	-2.33	0.02	0.03
ENSSSCG00000015565	C1orf21	398.55	-2.87	0.12	-23.13	0.00	0.00
ENSSSCG00000022126	EGFR	2782.70	-2.86	0.06	-48.54	0.00	0.00
ENSSSCG00000032271		6.74	-2.86	0.90	-3.17	0.00	0.00
ENSSSCG00000040466	FMN1	122.96	-2.86	0.22	-13.22	0.00	0.00
ENSSSCG00000040538	WDFY2	144.69	-2.86	0.20	-13.97	0.00	0.00
ENSSSCG00000025210	ATG10	74.30	-2.86	0.29	-9.92	0.00	0.00
ENSSSCG00000003044	LYPD4	2.70	-2.85	1.45	-1.96	0.05	0.07
ENSSSCG00000002436	TTC7B	303.14	-2.85	0.14	-20.62	0.00	0.00
ENSSSCG00000006115	RUNX1T1	290.57	-2.85	0.14	-20.10	0.00	0.00
ENSSSCG00000016520	CREB3L2	1295.01	-2.85	0.08	-37.94	0.00	0.00
ENSSSCG00000018091	ND5	69875.13	-2.85	0.11	-25.29	0.00	0.00
ENSSSCG00000028148	DMD	130.90	-2.84	0.21	-13.36	0.00	0.00
ENSSSCG00000012434	ATRX	1526.41	-2.84	0.07	-38.62	0.00	0.00
ENSSSCG00000024088	TLN2	708.31	-2.84	0.09	-30.09	0.00	0.00
ENSSSCG00000005225	RFX3	37.14	-2.84	0.39	-7.29	0.00	0.00
ENSSSCG00000012253		366.82	-2.83	0.13	-22.02	0.00	0.00
ENSSSCG00000010735	LHPP	147.82	-2.82	0.20	-13.85	0.00	0.00
ENSSSCG00000036695	IGF2BP3	2334.68	-2.82	0.06	-47.96	0.00	0.00

ENSSSCG00000010571	ARMH3	242.19	-2.82	0.16	-17.95	0.00	0.00
ENSSSCG00000033800	PELI2	6.56	-2.81	0.90	-3.12	0.00	0.00
ENSSSCG00000040397	COLEC12	6.56	-2.81	0.90	-3.11	0.00	0.00
ENSSSCG00000004509	LIPG	10140.75	-2.81	0.06	-46.56	0.00	0.00
ENSSSCG00000039867	UCK2	2374.74	-2.81	0.06	-43.46	0.00	0.00
ENSSSCG00000008753	ADGRA3	701.83	-2.80	0.10	-28.96	0.00	0.00
ENSSSCG00000024517	AKAP6	286.89	-2.80	0.14	-19.51	0.00	0.00
ENSSSCG00000005110	SYNE2	1385.27	-2.80	0.07	-39.09	0.00	0.00
ENSSSCG00000004508	DYM	584.72	-2.79	0.10	-27.88	0.00	0.00
ENSSSCG00000005136	IFNE	56.85	-2.79	0.31	-9.03	0.00	0.00
ENSSSCG00000009084	SPATA5	207.41	-2.78	0.17	-16.86	0.00	0.00
ENSSSCG00000005166	MLLT3	98.99	-2.78	0.25	-11.16	0.00	0.00
ENSSSCG00000009868	RBM19	1647.09	-2.78	0.07	-40.79	0.00	0.00
ENSSSCG00000021527	ATP10B	7.75	-2.78	0.83	-3.35	0.00	0.00
ENSSSCG00000040896		3.89	-2.78	1.18	-2.36	0.02	0.03
ENSSSCG00000004207		14.17	-2.78	0.62	-4.48	0.00	0.00
ENSSSCG00000004018	AFDN	1571.70	-2.77	0.07	-41.06	0.00	0.00
ENSSSCG00000010651	ABLIM1	369.67	-2.77	0.12	-22.42	0.00	0.00
ENSSSCG00000018069	ND2	30330.28	-2.77	0.19	-14.35	0.00	0.00
ENSSSCG00000040728	EYA1	32.01	-2.77	0.43	-6.51	0.00	0.00
ENSSSCG00000036702	ACTN1	8319.81	-2.77	0.04	-67.06	0.00	0.00
ENSSSCG00000013351	NAV2	230.68	-2.77	0.16	-17.76	0.00	0.00
ENSSSCG00000011714	MED12L	97.90	-2.77	0.24	-11.75	0.00	0.00
ENSSSCG00000010683	GRK5	177.26	-2.76	0.18	-15.08	0.00	0.00
ENSSSCG00000007858		103.90	-2.76	0.23	-11.74	0.00	0.00

ENSSSCG00000012110	MID1	226.44	-2.74	0.16	-16.67	0.00	0.00
ENSSSCG00000001639	TRERF1	292.86	-2.74	0.29	-9.40	0.00	0.00
ENSSSCG00000017578	ITGA3	37156.03	-2.74	0.04	-72.91	0.00	0.00
ENSSSCG00000024206	CPPED1	90.09	-2.74	0.25	-10.89	0.00	0.00
ENSSSCG00000020858	KIF13A	490.33	-2.74	0.12	-23.75	0.00	0.00
ENSSSCG00000002526	RCOR1	1332.26	-2.74	0.08	-34.53	0.00	0.00
ENSSSCG00000016035	COL5A2	5.06	-2.74	1.05	-2.60	0.01	0.01
ENSSSCG00000035690	SFPQ	6181.10	-2.73	0.04	-63.70	0.00	0.00
ENSSSCG00000031159	BEND3	688.88	-2.73	0.10	-28.68	0.00	0.00
ENSSSCG00000029251		4807.15	-2.73	0.05	-59.23	0.00	0.00
ENSSSCG00000011085	MLLT10	407.38	-2.73	0.12	-22.88	0.00	0.00
ENSSSCG00000035198		6.23	-2.73	0.93	-2.94	0.00	0.01
ENSSSCG00000004379	SOBP	55.66	-2.72	0.32	-8.49	0.00	0.00
ENSSSCG00000040183	CDK6	1310.71	-2.72	0.08	-35.74	0.00	0.00
ENSSSCG00000025784	CDH4	3.69	-2.72	1.19	-2.28	0.02	0.03
ENSSSCG00000009200		3.68	-2.72	1.24	-2.19	0.03	0.04
ENSSSCG00000033654	FANCC	39.72	-2.72	0.36	-7.47	0.00	0.00
ENSSSCG00000015336	SLC25A13	1418.12	-2.72	0.07	-39.39	0.00	0.00
ENSSSCG00000039476		3.71	-2.72	1.26	-2.15	0.03	0.04
ENSSSCG00000016090	SPATS2L	1026.25	-2.71	0.08	-34.55	0.00	0.00
ENSSSCG00000013400	MICAL2	1283.20	-2.71	0.07	-36.60	0.00	0.00
ENSSSCG00000022649	SLC7A11	245.85	-2.71	0.15	-17.68	0.00	0.00
ENSSSCG00000038194	RF02166	4.86	-2.70	1.05	-2.58	0.01	0.01
ENSSSCG00000004349		370.23	-2.70	0.12	-21.82	0.00	0.00
ENSSSCG00000011720	MBNL1	3796.51	-2.70	0.06	-43.61	0.00	0.00

ENSSSCG00000007456	SULF2	202.83	-2.70	0.17	-15.93	0.00	0.00
ENSSSCG00000000770	MICAL3	343.52	-2.69	0.13	-20.65	0.00	0.00
ENSSSCG00000000766	CECR2	206.14	-2.69	0.17	-16.03	0.00	0.00
ENSSSCG00000015839	NRG1	84.09	-2.69	0.25	-10.77	0.00	0.00
ENSSSCG00000035243	RAB27B	65.82	-2.69	0.28	-9.50	0.00	0.00
ENSSSCG00000037723		14.64	-2.69	0.59	-4.53	0.00	0.00
ENSSSCG00000014225	DTWD2	36.53	-2.69	0.38	-7.11	0.00	0.00
ENSSSCG00000015582	PTPN14	833.05	-2.69	0.09	-30.15	0.00	0.00
ENSSSCG00000006453	KIRREL1	1151.50	-2.69	0.08	-35.80	0.00	0.00
ENSSSCG00000001061	JARID2	159.07	-2.69	0.19	-14.53	0.00	0.00
ENSSSCG00000010315	ADK	1127.03	-2.69	0.07	-36.02	0.00	0.00
ENSSSCG00000031940	GAS2	10.94	-2.69	0.70	-3.86	0.00	0.00
ENSSSCG00000034610		213.49	-2.68	0.16	-16.87	0.00	0.00
ENSSSCG00000022417	HNF1B	1744.77	-2.68	0.07	-37.56	0.00	0.00
ENSSSCG00000014975		6.08	-2.68	0.93	-2.88	0.00	0.01
ENSSSCG00000026113	ZBTB20	83.62	-2.68	0.25	-10.68	0.00	0.00
ENSSSCG00000039009		4.91	-2.68	1.08	-2.49	0.01	0.02
ENSSSCG00000039374		7.24	-2.68	0.85	-3.15	0.00	0.00
ENSSSCG00000033971	ZNF407	277.00	-2.68	0.14	-18.79	0.00	0.00
ENSSSCG00000035479	DISP1	160.82	-2.68	0.18	-14.71	0.00	0.00
ENSSSCG00000003586	EPB41	2782.13	-2.68	0.06	-48.51	0.00	0.00
ENSSSCG00000014820	FCHSD2	471.98	-2.67	0.11	-23.68	0.00	0.00
ENSSSCG00000013292	PRR5L	736.44	-2.67	0.09	-28.50	0.00	0.00
ENSSSCG00000002688		739.80	-2.67	0.10	-27.92	0.00	0.00
ENSSSCG00000040166	FLRT2	1416.45	-2.67	0.07	-37.29	0.00	0.00

ENSSSCG00000010862	CDC42BPA	994.62	-2.67	0.08	-33.54	0.00	0.00
ENSSSCG00000011498	SLC25A26	189.27	-2.67	0.17	-15.35	0.00	0.00
ENSSSCG00000016857	DAB2	286.79	-2.67	0.14	-19.20	0.00	0.00
ENSSSCG00000036148		8.41	-2.66	0.79	-3.36	0.00	0.00
ENSSSCG00000018352	RF00186	20.38	-2.66	0.51	-5.25	0.00	0.00
ENSSSCG00000001534	ANKS1A	416.67	-2.66	0.12	-22.58	0.00	0.00
ENSSSCG00000002517	WDR25	112.70	-2.66	0.22	-11.91	0.00	0.00
ENSSSCG00000016941	RNF180	96.66	-2.66	0.23	-11.44	0.00	0.00
ENSSSCG00000017753	KSR1	119.38	-2.66	0.21	-12.56	0.00	0.00
ENSSSCG00000009037	ZNF827	226.10	-2.66	0.16	-16.23	0.00	0.00
ENSSSCG00000012885		681.17	-2.66	0.10	-27.52	0.00	0.00
ENSSSCG00000005604	PBX3	636.88	-2.65	0.10	-27.29	0.00	0.00
ENSSSCG00000025408	ULK4	148.83	-2.65	0.20	-13.39	0.00	0.00
ENSSSCG00000007878	PARN	221.69	-2.65	0.16	-16.65	0.00	0.00
ENSSSCG00000008581	NCOA1	297.05	-2.65	0.14	-19.51	0.00	0.00
ENSSSCG00000014339	CTNNA1	5528.35	-2.65	0.05	-58.03	0.00	0.00
ENSSSCG00000005186	TTC39B	75.71	-2.65	0.27	-9.84	0.00	0.00
ENSSSCG00000021200		167.35	-2.64	0.18	-14.72	0.00	0.00
ENSSSCG00000029860	EPB41L4A	651.84	-2.64	0.10	-25.44	0.00	0.00
ENSSSCG00000018333	RF00152	42.63	-2.64	0.35	-7.44	0.00	0.00
ENSSSCG00000015335	DYNC1I1	5.89	-2.63	0.93	-2.84	0.00	0.01
ENSSSCG00000029815	SRGAP1	179.67	-2.63	0.17	-15.33	0.00	0.00
ENSSSCG00000020663	KMT2C	775.85	-2.63	0.10	-26.69	0.00	0.00
ENSSSCG00000012956	PACS1	410.96	-2.63	0.12	-22.17	0.00	0.00
ENSSSCG00000001107	SLC17A1	5.90	-2.63	0.93	-2.83	0.00	0.01

ENSSSCG00000006956	ZC3H3	302.06	-2.63	0.14	-18.70	0.00	0.00
ENSSSCG00000005198	KDM4C	134.72	-2.63	0.20	-13.38	0.00	0.00
ENSSSCG00000024694	TAF4	804.87	-2.62	0.09	-30.28	0.00	0.00
ENSSSCG00000000020	PHF21B	58.72	-2.62	0.32	-8.23	0.00	0.00
ENSSSCG00000005719	RAPGEF1	651.88	-2.62	0.10	-26.97	0.00	0.00
ENSSSCG00000001021	RREB1	423.67	-2.61	0.12	-22.30	0.00	0.00
ENSSSCG00000014430	ABLIM3	824.43	-2.61	0.10	-25.51	0.00	0.00
ENSSSCG00000039171	MRTFB	1091.50	-2.61	0.08	-32.54	0.00	0.00
ENSSSCG00000010267	LRRC20	180.57	-2.61	0.18	-14.82	0.00	0.00
ENSSSCG00000035045	DOCK1	1024.92	-2.60	0.08	-31.74	0.00	0.00
ENSSSCG00000036679	SORBS2	3036.93	-2.60	0.05	-49.48	0.00	0.00
ENSSSCG00000031831	CADM1	315.52	-2.60	0.13	-19.64	0.00	0.00
ENSSSCG00000036505		15008.26	-2.60	0.04	-63.25	0.00	0.00
ENSSSCG00000009408	LRCH1	280.20	-2.60	0.14	-18.47	0.00	0.00
ENSSSCG00000005130	ELAVL2	337.87	-2.60	0.13	-19.80	0.00	0.00
ENSSSCG00000008513	BIRC6	2323.36	-2.59	0.06	-44.59	0.00	0.00
ENSSSCG00000021791	SENP7	138.72	-2.59	0.20	-13.03	0.00	0.00
ENSSSCG00000038128	CDYL2	140.29	-2.59	0.20	-12.92	0.00	0.00
ENSSSCG00000003761	ADGRL2	1671.53	-2.59	0.07	-37.47	0.00	0.00
ENSSSCG00000012089	ADARB1	107.78	-2.59	0.22	-11.55	0.00	0.00
ENSSSCG00000014195	MAN2A1	869.07	-2.59	0.08	-31.33	0.00	0.00
ENSSSCG00000016164	IKZF2	199.97	-2.59	0.16	-15.93	0.00	0.00
ENSSSCG00000013915	CRTC1	262.81	-2.59	0.14	-18.32	0.00	0.00
ENSSSCG00000018082	COX3	78129.72	-2.59	0.14	-19.11	0.00	0.00
ENSSSCG00000013321	ELP4	275.45	-2.58	0.14	-18.57	0.00	0.00

ENSSSCG00000004225	TPD52L1	5.75	-2.57	0.97	-2.66	0.01	0.01
ENSSSCG00000036812	MPRIP	1605.82	-2.57	0.06	-40.12	0.00	0.00
ENSSSCG00000039215	PHYHIPL	1309.43	-2.57	0.07	-37.00	0.00	0.00
ENSSSCG00000016545	CHCHD3	785.11	-2.57	0.09	-29.27	0.00	0.00
ENSSSCG00000009684	MSRA	20.36	-2.56	0.50	-5.14	0.00	0.00
ENSSSCG00000035136	SDCCAG8	32.66	-2.56	0.40	-6.47	0.00	0.00
ENSSSCG00000033657	GREM1	5364.38	-2.56	0.06	-43.23	0.00	0.00
ENSSSCG00000035254	DPH6	316.90	-2.55	0.14	-18.78	0.00	0.00
ENSSSCG00000035520		11959.57	-2.55	0.24	-10.73	0.00	0.00
ENSSSCG00000022194		73.84	-2.55	0.26	-9.63	0.00	0.00
ENSSSCG00000018094	CYTB	56021.52	-2.55	0.22	-11.70	0.00	0.00
ENSSSCG00000038081	AGPAT3	1158.36	-2.55	0.08	-33.76	0.00	0.00
ENSSSCG00000002427		138.87	-2.54	0.19	-13.16	0.00	0.00
ENSSSCG00000014267		641.45	-2.54	0.10	-25.16	0.00	0.00
ENSSSCG00000000999		286.90	-2.54	0.14	-18.62	0.00	0.00
ENSSSCG00000015136	UBASH3B	1122.33	-2.53	0.08	-32.03	0.00	0.00
ENSSSCG00000031174	FBP1	37.70	-2.53	0.37	-6.86	0.00	0.00
ENSSSCG00000007547	ADAP1	265.85	-2.53	0.14	-18.16	0.00	0.00
ENSSSCG00000024722	PIBF1	385.33	-2.53	0.12	-21.18	0.00	0.00
ENSSSCG00000032049	ZZEF1	1426.31	-2.53	0.07	-37.72	0.00	0.00
ENSSSCG00000008836		21.05	-2.53	0.49	-5.18	0.00	0.00
ENSSSCG00000030480	DYRK1A	689.24	-2.53	0.10	-26.11	0.00	0.00
ENSSSCG00000004753	INO80	828.26	-2.52	0.08	-29.98	0.00	0.00
ENSSSCG00000015398	SEMA3A	7.77	-2.52	0.81	-3.12	0.00	0.00
ENSSSCG00000028827	PARVA	585.55	-2.52	0.10	-25.81	0.00	0.00

ENSSSCG00000001486	LRRC1	203.37	-2.52	0.16	-15.65	0.00	0.00
ENSSSCG00000022553	TNRC18	811.16	-2.52	0.08	-29.70	0.00	0.00
ENSSSCG00000015401		419.07	-2.52	0.11	-22.12	0.00	0.00
ENSSSCG00000029813	TSPAN5	592.63	-2.52	0.10	-26.10	0.00	0.00
ENSSSCG00000000145	MYH9	12523.72	-2.51	0.04	-63.13	0.00	0.00
ENSSSCG00000026110	SRPK2	859.42	-2.51	0.09	-28.63	0.00	0.00
ENSSSCG00000034598	HIST2H2AC	6.57	-2.51	0.87	-2.88	0.00	0.01
ENSSSCG00000001716	RCAN2	689.38	-2.51	0.09	-27.78	0.00	0.00
ENSSSCG00000005269	TRPM6	15.31	-2.51	0.57	-4.42	0.00	0.00
ENSSSCG00000031462	ZNRF1	358.49	-2.51	0.12	-20.27	0.00	0.00
ENSSSCG00000025901	LCLAT1	471.35	-2.51	0.11	-23.51	0.00	0.00
ENSSSCG00000003705	CABLES1	115.57	-2.50	0.21	-11.75	0.00	0.00
ENSSSCG00000001746	PKHD1	133.64	-2.50	0.20	-12.71	0.00	0.00
ENSSSCG00000024614	ITSN1	1060.92	-2.50	0.08	-32.63	0.00	0.00
ENSSSCG00000015346	ICA1	196.78	-2.50	0.16	-15.46	0.00	0.00
ENSSSCG00000029413	DNMT3A	80.61	-2.50	0.26	-9.78	0.00	0.00
ENSSSCG00000021203	PIK3CB	676.22	-2.50	0.09	-26.90	0.00	0.00
ENSSSCG00000016885	ITGA1	544.62	-2.50	0.10	-24.08	0.00	0.00
ENSSSCG00000014088	IQGAP2	1635.83	-2.50	0.06	-39.45	0.00	0.00
ENSSSCG00000003722	CDH2	69.28	-2.50	0.58	-4.28	0.00	0.00
ENSSSCG00000000288	HNRNPA1	10367.47	-2.49	0.04	-63.44	0.00	0.00
ENSSSCG00000039373	SNX24	283.80	-2.49	0.14	-18.04	0.00	0.00
ENSSSCG00000039770	SLC6A9	594.29	-2.49	0.10	-23.75	0.00	0.00
ENSSSCG00000013382	PLEKHA7	757.92	-2.49	0.09	-27.50	0.00	0.00
ENSSSCG00000014190	FBXL17	86.22	-2.49	0.24	-10.29	0.00	0.00

ENSSSCG00000000735	TSPAN9	47.20	-2.49	0.34	-7.37	0.00	0.00
ENSSSCG00000014904	DLG2	139.83	-2.48	0.19	-12.79	0.00	0.00
ENSSSCG00000028117		209.12	-2.48	0.16	-15.65	0.00	0.00
ENSSSCG00000011023	ZNF438	11.74	-2.48	0.65	-3.80	0.00	0.00
ENSSSCG00000006250	LYN	1413.10	-2.48	0.07	-35.53	0.00	0.00
ENSSSCG00000009850	TAOK3	370.96	-2.48	0.12	-20.73	0.00	0.00
ENSSSCG00000026996		1253.11	-2.48	0.08	-32.02	0.00	0.00
ENSSSCG00000032444	PLXDC2	68.59	-2.48	0.27	-9.27	0.00	0.00
ENSSSCG00000005480		140.09	-2.47	0.19	-12.78	0.00	0.00
ENSSSCG00000036169	UMAD1	124.79	-2.47	0.20	-12.33	0.00	0.00
ENSSSCG00000038698	RF00614	6.39	-2.46	0.89	-2.76	0.01	0.01
ENSSSCG00000038220	RXRA	298.27	-2.46	0.14	-18.11	0.00	0.00
ENSSSCG00000013043	MACROD1	50.91	-2.46	0.32	-7.72	0.00	0.00
ENSSSCG00000009035	SLC10A7	107.45	-2.46	0.21	-11.45	0.00	0.00
ENSSSCG00000015563	RGL1	213.12	-2.46	0.16	-15.31	0.00	0.00
ENSSSCG00000014126	MSH3	188.61	-2.46	0.18	-13.89	0.00	0.00
ENSSSCG00000028063	TACC2	174.99	-2.45	0.18	-13.61	0.00	0.00
ENSSSCG00000034390	CARD11	275.25	-2.45	0.14	-17.99	0.00	0.00
ENSSSCG00000002410	CEP128	239.10	-2.45	0.15	-16.57	0.00	0.00
ENSSSCG00000015882	BAZ2B	308.82	-2.45	0.14	-17.79	0.00	0.00
ENSSSCG00000005503	TLR4	465.49	-2.45	0.11	-22.95	0.00	0.00
ENSSSCG00000009192	PDLIM5	1456.83	-2.44	0.07	-35.02	0.00	0.00
ENSSSCG00000004358	ASCC3	1507.91	-2.44	0.07	-36.69	0.00	0.00
ENSSSCG00000014161		39.94	-2.44	0.36	-6.70	0.00	0.00
ENSSSCG00000011610	NUP210	698.49	-2.43	0.09	-26.45	0.00	0.00

ENSSSCG00000034853		8586.47	-2.43	0.06	-39.63	0.00	0.00
ENSSSCG00000038417	LRRC8D	1352.79	-2.43	0.07	-32.75	0.00	0.00
ENSSSCG00000030153	SMURF1	945.73	-2.43	0.08	-28.90	0.00	0.00
ENSSSCG00000029744	PLCH2	114.50	-2.43	0.21	-11.64	0.00	0.00
ENSSSCG00000034357	DAG1	7649.32	-2.42	0.04	-55.25	0.00	0.00
ENSSSCG00000004955	MAP2K5	262.76	-2.42	0.14	-17.29	0.00	0.00
ENSSSCG00000013347	PRMT3	559.02	-2.42	0.10	-24.57	0.00	0.00
ENSSSCG00000027941	LSAMP	8.23	-2.42	0.78	-3.11	0.00	0.00
ENSSSCG00000010302	USP54	331.17	-2.42	0.13	-19.29	0.00	0.00
ENSSSCG00000005346	ZCCHC7	113.60	-2.41	0.21	-11.41	0.00	0.00
ENSSSCG00000009511	STK24	2055.25	-2.41	0.06	-41.08	0.00	0.00
ENSSSCG00000025984	TTLL5	661.44	-2.41	0.10	-24.17	0.00	0.00
ENSSSCG00000031767	TANGO6	219.87	-2.41	0.15	-15.85	0.00	0.00
ENSSSCG00000035867	GFOD1	139.96	-2.41	0.19	-12.67	0.00	0.00
ENSSSCG00000008468	PKDCC	666.28	-2.40	0.09	-25.55	0.00	0.00
ENSSSCG00000037424	CDK19	140.34	-2.40	0.19	-12.76	0.00	0.00
ENSSSCG00000018078	COX2	50859.18	-2.40	0.13	-18.06	0.00	0.00
ENSSSCG00000030110		380.70	-2.40	0.12	-20.17	0.00	0.00
ENSSSCG00000030016	PDE9A	10.24	-2.39	0.70	-3.43	0.00	0.00
ENSSSCG00000016988	ERGIC1	1908.40	-2.39	0.07	-34.23	0.00	0.00
ENSSSCG00000013281	EXT2	850.86	-2.39	0.08	-29.15	0.00	0.00
ENSSSCG00000034738	TRMT61A	742.16	-2.39	0.09	-27.51	0.00	0.00
ENSSSCG00000031592	ZNF462	420.63	-2.39	0.11	-20.82	0.00	0.00
ENSSSCG00000012315	SYNJ2	816.07	-2.39	0.09	-27.43	0.00	0.00
ENSSSCG00000039714	MIPOL1	146.07	-2.38	0.19	-12.72	0.00	0.00

ENSSSCG00000003512	EIF4G3	2753.21	-2.38	0.05	-43.66	0.00	0.00
ENSSSCG00000004919	NEDD4L	467.75	-2.38	0.11	-21.29	0.00	0.00
ENSSSCG00000034062	SV2C	5.03	-2.37	0.96	-2.46	0.01	0.02
ENSSSCG00000032778	PLEKHG1	63.72	-2.37	0.28	-8.58	0.00	0.00
ENSSSCG00000011033	VIM	85526.61	-2.37	0.04	-67.04	0.00	0.00
ENSSSCG00000019803	RF00019	7.07	-2.37	0.82	-2.90	0.00	0.01
ENSSSCG00000036520	EFNA5	115.40	-2.36	0.21	-11.38	0.00	0.00
ENSSSCG00000010943	AOPEP	364.89	-2.36	0.12	-19.54	0.00	0.00
ENSSSCG00000003797	DIRAS3	391.44	-2.36	0.12	-19.50	0.00	0.00
ENSSSCG00000011576	HRH1	124.82	-2.36	0.20	-11.53	0.00	0.00
ENSSSCG00000016245		53.90	-2.36	0.30	-7.81	0.00	0.00
ENSSSCG00000002835	TOX3	333.70	-2.35	0.12	-18.82	0.00	0.00
ENSSSCG00000012857	CARS	1026.83	-2.35	0.08	-30.92	0.00	0.00
ENSSSCG00000040392		40.89	-2.35	0.35	-6.71	0.00	0.00
ENSSSCG00000016806		811.51	-2.35	0.09	-26.75	0.00	0.00
ENSSSCG00000001865	SCAPER	138.91	-2.34	0.20	-11.85	0.00	0.00
ENSSSCG00000007720	GTF2IRD1	299.96	-2.34	0.13	-17.99	0.00	0.00
ENSSSCG00000004979	MYO9A	763.18	-2.34	0.09	-25.61	0.00	0.00
ENSSSCG00000029249	NAV3	979.70	-2.34	0.08	-29.11	0.00	0.00
ENSSSCG00000029627		1310.31	-2.34	0.07	-33.09	0.00	0.00
ENSSSCG00000007681	CUX1	451.75	-2.34	0.11	-21.77	0.00	0.00
ENSSSCG00000006743	SLC22A15	211.84	-2.34	0.16	-14.97	0.00	0.00
ENSSSCG00000004615	WDR72	180.14	-2.34	0.17	-13.94	0.00	0.00
ENSSSCG00000032229	WDPCP	43.55	-2.33	0.34	-6.92	0.00	0.00
ENSSSCG00000005170	DENND4C	1604.67	-2.33	0.07	-33.17	0.00	0.00

ENSSSCG00000000875	NR1H4	22.68	-2.33	0.45	-5.15	0.00	0.00
ENSSSCG00000008799	LIMCH1	452.09	-2.33	0.11	-21.20	0.00	0.00
ENSSSCG00000011943	BBX	500.07	-2.33	0.11	-21.97	0.00	0.00
ENSSSCG00000005190	NFIB	255.13	-2.33	0.14	-16.25	0.00	0.00
ENSSSCG00000011022	SVIL	500.09	-2.33	0.10	-22.50	0.00	0.00
ENSSSCG00000008617	FAM49A	313.82	-2.32	0.13	-18.05	0.00	0.00
ENSSSCG00000017054	CYFIP2	1600.21	-2.32	0.06	-35.90	0.00	0.00
ENSSSCG00000016824	RAI14	1862.67	-2.32	0.06	-36.30	0.00	0.00
ENSSSCG00000038066	USH2A	53.80	-2.32	0.31	-7.48	0.00	0.00
ENSSSCG00000009716	SH3RF1	916.44	-2.32	0.09	-27.11	0.00	0.00
ENSSSCG00000011296	ANO10	187.84	-2.32	0.16	-14.21	0.00	0.00
ENSSSCG00000012713	ATP11C	2481.68	-2.32	0.06	-39.15	0.00	0.00
ENSSSCG00000007606	TRRAP	3371.68	-2.32	0.05	-47.28	0.00	0.00
ENSSSCG00000028327	RHOBTB1	71.34	-2.32	0.26	-8.85	0.00	0.00
ENSSSCG00000011454	SFMBT1	200.38	-2.32	0.16	-14.28	0.00	0.00
ENSSSCG00000035395		4.87	-2.32	0.97	-2.39	0.02	0.02
ENSSSCG00000009446	PCDH17	39.95	-2.32	0.35	-6.56	0.00	0.00
ENSSSCG00000035371		4.86	-2.32	0.98	-2.35	0.02	0.03
ENSSSCG00000009023	DCLK2	48.72	-2.31	0.31	-7.39	0.00	0.00
ENSSSCG00000028406	FOCAD	1225.44	-2.31	0.07	-33.05	0.00	0.00
ENSSSCG00000026346	UBE3D	270.21	-2.31	0.14	-16.99	0.00	0.00
ENSSSCG00000011193	BTD	893.19	-2.30	0.08	-29.49	0.00	0.00
ENSSSCG00000002408	ADCK1	308.36	-2.30	0.13	-18.12	0.00	0.00
ENSSSCG00000038421		27.04	-2.30	0.42	-5.42	0.00	0.00
ENSSSCG00000027153	WARS2	345.32	-2.30	0.12	-19.09	0.00	0.00

ENSSSCG00000018065	ND1	18289.03	-2.29	0.23	-10.03	0.00	0.00
ENSSSCG00000029808	EIF2B3	299.69	-2.29	0.13	-17.77	0.00	0.00
ENSSSCG00000007000	FAT1	32871.28	-2.29	0.04	-61.17	0.00	0.00
ENSSSCG00000015888	RBMS1	1370.72	-2.29	0.07	-32.73	0.00	0.00
ENSSSCG00000015432	ATXN7L1	574.84	-2.29	0.10	-23.21	0.00	0.00
ENSSSCG00000031539		9.55	-2.29	0.70	-3.26	0.00	0.00
ENSSSCG00000036022		3.87	-2.29	1.11	-2.06	0.04	0.05
ENSSSCG00000019194	RF00289	6.72	-2.29	0.86	-2.67	0.01	0.01
ENSSSCG00000018081	ATP6	68011.28	-2.29	0.15	-15.61	0.00	0.00
ENSSSCG00000033186	RF00153	5.70	-2.29	0.93	-2.47	0.01	0.02
ENSSSCG00000037755	NSMCE2	108.39	-2.29	0.21	-10.75	0.00	0.00
ENSSSCG00000016942	CWC27	387.82	-2.28	0.12	-19.45	0.00	0.00
ENSSSCG00000022676	OPHN1	29.78	-2.28	0.42	-5.48	0.00	0.00
ENSSSCG00000015537	XPR1	1445.97	-2.28	0.08	-29.53	0.00	0.00
ENSSSCG00000038663		12.42	-2.28	0.62	-3.69	0.00	0.00
ENSSSCG00000015238	ARHGAP32	531.50	-2.27	0.10	-22.89	0.00	0.00
ENSSSCG00000004952	SMAD3	872.28	-2.27	0.08	-27.91	0.00	0.00
ENSSSCG00000009256	ANTXR2	1291.43	-2.27	0.07	-31.70	0.00	0.00
ENSSSCG00000008382	USP34	1675.98	-2.26	0.07	-33.47	0.00	0.00
ENSSSCG00000022081	PHLDB2	3310.19	-2.26	0.05	-43.14	0.00	0.00
ENSSSCG00000002664	GSE1	755.80	-2.26	0.09	-25.43	0.00	0.00
ENSSSCG00000026569	CLEC16A	210.93	-2.26	0.15	-14.93	0.00	0.00
ENSSSCG00000004659	SLC12A1	37.00	-2.26	0.36	-6.32	0.00	0.00
ENSSSCG00000024351	MET	9437.04	-2.26	0.05	-48.89	0.00	0.00
ENSSSCG00000009276	XPO4	478.34	-2.26	0.10	-21.86	0.00	0.00

ENSSSCG00000025423	KCNK5	1321.50	-2.25	0.07	-31.00	0.00	0.00
ENSSSCG00000022486	CBLB	162.30	-2.25	0.18	-12.80	0.00	0.00
ENSSSCG00000005609	GARNL3	124.09	-2.25	0.20	-11.43	0.00	0.00
ENSSSCG00000006893	BCAR3	999.42	-2.25	0.08	-27.14	0.00	0.00
ENSSSCG00000004311	RNGTT	437.53	-2.25	0.11	-20.64	0.00	0.00
ENSSSCG00000022478	STK10	1638.72	-2.25	0.06	-35.46	0.00	0.00
ENSSSCG00000015632		28.20	-2.25	0.42	-5.40	0.00	0.00
ENSSSCG00000007896	TXNDC11	768.65	-2.25	0.08	-26.87	0.00	0.00
ENSSSCG00000022032		1433.75	-2.25	0.07	-30.06	0.00	0.00
ENSSSCG00000006901	EVI5	1039.55	-2.25	0.08	-28.19	0.00	0.00
ENSSSCG00000001972	AP4S1	48.71	-2.24	0.31	-7.24	0.00	0.00
ENSSSCG00000021309	TRDMT1	436.45	-2.24	0.11	-20.46	0.00	0.00
ENSSSCG00000040967	NACC2	518.86	-2.24	0.10	-22.04	0.00	0.00
ENSSSCG00000004082		131.01	-2.24	0.19	-11.75	0.00	0.00
ENSSSCG00000010468	CPEB3	18.63	-2.24	0.50	-4.48	0.00	0.00
ENSSSCG00000014440	HMGXB3	1065.21	-2.24	0.08	-29.46	0.00	0.00
ENSSSCG00000029837	VWA8	850.54	-2.24	0.08	-28.10	0.00	0.00
ENSSSCG00000015944	TLK1	823.40	-2.24	0.09	-25.27	0.00	0.00
ENSSSCG00000016674	MINDY4	494.37	-2.24	0.10	-22.28	0.00	0.00
ENSSSCG00000003405	PEX14	207.33	-2.24	0.15	-14.53	0.00	0.00
ENSSSCG00000010360	BMPR1A	475.70	-2.24	0.12	-18.62	0.00	0.00
ENSSSCG00000007074	TASP1	190.28	-2.24	0.16	-13.97	0.00	0.00
ENSSSCG00000011075	KIAA1217	1353.64	-2.23	0.07	-32.83	0.00	0.00
ENSSSCG00000014079		36.30	-2.23	0.36	-6.26	0.00	0.00
ENSSSCG00000009123	CAMK2D	456.95	-2.23	0.10	-21.41	0.00	0.00

ENSSSCG00000015876	CCDC148	23.17	-2.23	0.45	-5.01	0.00	0.00
ENSSSCG00000026579		6.55	-2.23	0.86	-2.58	0.01	0.01
ENSSSCG00000008809	GABRG1	117.52	-2.23	0.20	-10.94	0.00	0.00
ENSSSCG00000015692	R3HDM1	1650.03	-2.23	0.06	-34.58	0.00	0.00
ENSSSCG00000008147	FHL2	3087.03	-2.22	0.05	-42.99	0.00	0.00
ENSSSCG00000029196	DIP2B	490.54	-2.22	0.11	-21.09	0.00	0.00
ENSSSCG00000025685	TP73	833.45	-2.22	0.08	-26.61	0.00	0.00
ENSSSCG00000038731	STX18	273.57	-2.22	0.14	-15.79	0.00	0.00
ENSSSCG00000036560	PAWR	1023.14	-2.22	0.08	-27.64	0.00	0.00
ENSSSCG00000004242	TBC1D32	78.91	-2.22	0.25	-8.86	0.00	0.00
ENSSSCG00000003438	VPS13D	1374.84	-2.22	0.07	-33.21	0.00	0.00
ENSSSCG00000032024	URB1	994.08	-2.22	0.08	-27.53	0.00	0.00
ENSSSCG00000006735	PTGFRN	4322.56	-2.22	0.05	-44.77	0.00	0.00
ENSSSCG00000008475	MAP4K3	869.82	-2.22	0.09	-25.88	0.00	0.00
ENSSSCG00000038973	PPP1R12A	910.42	-2.22	0.08	-27.22	0.00	0.00
ENSSSCG00000026498		1331.01	-2.22	0.07	-33.29	0.00	0.00
ENSSSCG00000010699	ATE1	472.92	-2.22	0.10	-21.42	0.00	0.00
ENSSSCG00000037418		12.96	-2.21	0.60	-3.70	0.00	0.00
ENSSSCG00000033949	GNG7	15.59	-2.21	0.54	-4.06	0.00	0.00
ENSSSCG00000003514	HSPG2	22225.73	-2.21	0.04	-58.51	0.00	0.00
ENSSSCG00000011514	MITF	372.10	-2.21	0.12	-18.42	0.00	0.00
ENSSSCG00000014817	ARAP1	927.72	-2.21	0.08	-27.92	0.00	0.00
ENSSSCG00000000920	POC1B	467.48	-2.21	0.11	-20.84	0.00	0.00
ENSSSCG00000007337	CTNNBL1	704.50	-2.21	0.09	-25.02	0.00	0.00
ENSSSCG00000005224	GLIS3	219.47	-2.21	0.16	-14.13	0.00	0.00

ENSSSCG00000016244	COL4A4	184.13	-2.20	0.16	-13.69	0.00	0.00
ENSSSCG00000002656	ZCCHC14	1106.28	-2.20	0.08	-29.31	0.00	0.00
ENSSSCG00000003493	CAPZB	1700.17	-2.20	0.07	-33.47	0.00	0.00
ENSSSCG00000039306		73.91	-2.20	0.26	-8.55	0.00	0.00
ENSSSCG00000005935	AGO2	1501.81	-2.20	0.07	-32.64	0.00	0.00
ENSSSCG00000008635	NOL10	933.38	-2.20	0.08	-28.24	0.00	0.00
ENSSSCG00000024253	CTCF	1537.17	-2.20	0.06	-34.42	0.00	0.00
ENSSSCG00000006074	STK3	489.95	-2.20	0.11	-20.82	0.00	0.00
ENSSSCG00000010483	PLCE1	189.44	-2.20	0.16	-13.94	0.00	0.00
ENSSSCG00000025703	REV3L	639.24	-2.20	0.10	-23.07	0.00	0.00
ENSSSCG00000009682	HMBOX1	411.42	-2.19	0.11	-20.08	0.00	0.00
ENSSSCG00000018023	COX10	510.57	-2.19	0.10	-21.61	0.00	0.00
ENSSSCG00000035720	HRCT1	4.53	-2.19	0.99	-2.21	0.03	0.04
ENSSSCG00000014927	NOX4	328.73	-2.19	0.12	-17.71	0.00	0.00
ENSSSCG00000026382	PPP2R5E	844.70	-2.19	0.08	-26.57	0.00	0.00
ENSSSCG00000040100	ITPKB	22.68	-2.19	0.44	-4.92	0.00	0.00
ENSSSCG00000004092	MTHFD1L	1525.36	-2.18	0.07	-30.63	0.00	0.00
ENSSSCG00000038649		4.53	-2.18	1.00	-2.19	0.03	0.04
ENSSSCG00000016113	BMPR2	1105.26	-2.18	0.07	-29.17	0.00	0.00
ENSSSCG00000027763	NDUFAF2	131.00	-2.18	0.20	-10.98	0.00	0.00
ENSSSCG00000005471	SNX30	174.57	-2.18	0.17	-13.05	0.00	0.00
ENSSSCG00000028225	DICER1	2969.30	-2.18	0.05	-41.88	0.00	0.00
ENSSSCG00000014113	HOMER1	746.50	-2.18	0.09	-25.17	0.00	0.00
ENSSSCG00000029855	LHFPL6	303.05	-2.18	0.13	-17.25	0.00	0.00
ENSSSCG00000007454		526.21	-2.18	0.10	-21.22	0.00	0.00

ENSSSCG00000037676		80.25	-2.18	0.25	-8.64	0.00	0.00
ENSSSCG00000006767	MAGI3	434.96	-2.18	0.11	-19.63	0.00	0.00
ENSSSCG00000000804	ANO6	1355.96	-2.17	0.07	-32.73	0.00	0.00
ENSSSCG00000020813	FAM20C	1115.38	-2.17	0.07	-30.71	0.00	0.00
ENSSSCG00000006087	CPQ	341.75	-2.17	0.13	-17.12	0.00	0.00
ENSSSCG00000028814	SOD3	5303.18	-2.17	0.05	-44.12	0.00	0.00
ENSSSCG00000037910	DUSP6	3450.55	-2.17	0.05	-45.96	0.00	0.00
ENSSSCG00000040013	MTUS1	400.99	-2.17	0.11	-19.34	0.00	0.00
ENSSSCG00000008754	GBA3	13.41	-2.17	0.59	-3.70	0.00	0.00
ENSSSCG00000011579	PPARG	1434.86	-2.17	0.07	-31.38	0.00	0.00
ENSSSCG00000001950		186.52	-2.17	0.16	-13.50	0.00	0.00
ENSSSCG00000007951	CREBBP	1270.41	-2.16	0.07	-31.89	0.00	0.00
ENSSSCG00000014909		1285.86	-2.16	0.07	-29.35	0.00	0.00
ENSSSCG00000040199	AFG1L	116.65	-2.16	0.21	-10.26	0.00	0.00
ENSSSCG00000016412	LMBR1	438.59	-2.16	0.11	-19.57	0.00	0.00
ENSSSCG00000012741	MAMLD1	381.20	-2.16	0.12	-18.48	0.00	0.00
ENSSSCG00000033312		17.82	-2.16	0.51	-4.24	0.00	0.00
ENSSSCG00000006294	NME7	142.60	-2.16	0.18	-11.91	0.00	0.00
ENSSSCG00000037782	MRPS28	401.60	-2.16	0.11	-19.28	0.00	0.00
ENSSSCG00000034692	SH3BP4	3018.62	-2.16	0.06	-38.59	0.00	0.00
ENSSSCG00000011729		286.70	-2.16	0.13	-16.12	0.00	0.00
ENSSSCG00000009650	DOCK5	951.60	-2.15	0.08	-28.65	0.00	0.00
ENSSSCG00000011278	TRAK1	486.86	-2.15	0.10	-20.77	0.00	0.00
ENSSSCG00000039053	VGF	4881.86	-2.15	0.06	-34.28	0.00	0.00
ENSSSCG00000029456	SLC7A1	1740.17	-2.15	0.06	-33.18	0.00	0.00

ENSSSCG00000021027	PGBD5	60.30	-2.15	0.28	-7.67	0.00	0.00
ENSSSCG00000002729		984.48	-2.15	0.08	-28.06	0.00	0.00
ENSSSCG00000010433	SGMS1	331.80	-2.15	0.12	-17.87	0.00	0.00
ENSSSCG00000016061	MYO1B	778.93	-2.15	0.08	-26.10	0.00	0.00
ENSSSCG00000016629	ST7	398.52	-2.15	0.12	-18.28	0.00	0.00
ENSSSCG00000036887	CLPB	432.72	-2.14	0.11	-19.63	0.00	0.00
ENSSSCG00000011750	PLD1	273.82	-2.14	0.14	-15.10	0.00	0.00
ENSSSCG00000012862	OSBPL5	169.47	-2.14	0.17	-12.24	0.00	0.00
ENSSSCG00000037781		31.72	-2.14	0.39	-5.54	0.00	0.00
ENSSSCG00000015140	HSPA8	45027.41	-2.14	0.05	-45.44	0.00	0.00
ENSSSCG00000037846	ACOXL	36.08	-2.14	0.35	-6.07	0.00	0.00
ENSSSCG00000007360	CHD6	241.55	-2.14	0.15	-14.65	0.00	0.00
ENSSSCG00000039723		11.39	-2.14	0.63	-3.38	0.00	0.00
ENSSSCG00000032029	PPP2R3B	173.21	-2.14	0.17	-12.90	0.00	0.00
ENSSSCG00000011870	PDIA5	405.14	-2.13	0.11	-19.33	0.00	0.00
ENSSSCG00000004328	MDN1	2375.33	-2.13	0.06	-38.32	0.00	0.00
ENSSSCG00000005449	PTPN3	918.96	-2.13	0.08	-26.42	0.00	0.00
ENSSSCG00000007072	SPTLC3	864.99	-2.13	0.08	-27.38	0.00	0.00
ENSSSCG00000010211	CCDC6	1207.16	-2.13	0.08	-28.32	0.00	0.00
ENSSSCG00000009705	GALNT7	1150.22	-2.13	0.07	-29.63	0.00	0.00
ENSSSCG00000014248	LMNB1	3771.66	-2.13	0.05	-46.42	0.00	0.00
ENSSSCG00000012156	CDKL5	14.94	-2.13	0.55	-3.90	0.00	0.00
ENSSSCG00000015394	KIAA1324L	84.03	-2.13	0.24	-8.92	0.00	0.00
ENSSSCG00000009765	DNAH10	220.20	-2.13	0.15	-14.29	0.00	0.00
ENSSSCG00000009545	COL4A2	3300.15	-2.13	0.05	-42.39	0.00	0.00

ENSSSCG00000027777		646.50	-2.13	0.09	-23.42	0.00	0.00
ENSSSCG00000008467	EML4	2032.42	-2.12	0.06	-35.07	0.00	0.00
ENSSSCG00000019059	RF00026	4.36	-2.12	1.01	-2.11	0.03	0.05
ENSSSCG00000011819	MB21D2	79.38	-2.12	0.24	-8.66	0.00	0.00
ENSSSCG00000006725	TBX15	426.22	-2.12	0.11	-18.81	0.00	0.00
ENSSSCG00000001062	DTNBP1	43.43	-2.12	0.34	-6.18	0.00	0.00
ENSSSCG00000000778	CPNE8	83.88	-2.12	0.24	-8.78	0.00	0.00
ENSSSCG00000035593		146.40	-2.12	0.18	-11.86	0.00	0.00
ENSSSCG00000006734	CD101	8.74	-2.12	0.73	-2.92	0.00	0.01
ENSSSCG00000026981	MRPS6	1073.04	-2.11	0.08	-27.49	0.00	0.00
ENSSSCG00000005785	PCSK6	803.41	-2.11	0.08	-25.45	0.00	0.00
ENSSSCG00000011593	TMCC1	318.99	-2.11	0.12	-17.28	0.00	0.00
ENSSSCG00000036705	RPH3AL	165.01	-2.11	0.17	-12.23	0.00	0.00
ENSSSCG00000010878	EFCAB2	20.00	-2.11	0.47	-4.47	0.00	0.00
ENSSSCG00000027898	ATP2B1	1950.12	-2.11	0.06	-34.68	0.00	0.00
ENSSSCG00000016248	AGFG1	3744.67	-2.11	0.05	-45.25	0.00	0.00
ENSSSCG00000003818	DOCK7	3882.04	-2.11	0.05	-43.66	0.00	0.00
ENSSSCG00000005425	SLC44A1	2239.52	-2.11	0.06	-35.51	0.00	0.00
ENSSSCG00000031329	ST8SIA1	6.02	-2.10	0.87	-2.43	0.02	0.02
ENSSSCG00000012000	GBE1	388.74	-2.10	0.11	-18.52	0.00	0.00
ENSSSCG00000007572	LFNG	530.24	-2.10	0.10	-21.69	0.00	0.00
ENSSSCG00000038767	MBP	159.82	-2.10	0.17	-12.09	0.00	0.00
ENSSSCG00000011833	DLG1	1018.67	-2.10	0.08	-26.46	0.00	0.00
ENSSSCG00000016243	RHBDD1	224.98	-2.10	0.16	-13.36	0.00	0.00
ENSSSCG00000030798	FUS	3992.31	-2.10	0.05	-41.42	0.00	0.00

ENSSSCG00000019894	RF00091	54.23	-2.09	0.29	-7.30	0.00	0.00
ENSSSCG00000003627	ZMYM4	587.46	-2.09	0.10	-20.96	0.00	0.00
ENSSSCG00000027557		666.26	-2.09	0.09	-23.54	0.00	0.00
ENSSSCG00000039703	EEPD1	28.31	-2.09	0.41	-5.09	0.00	0.00
ENSSSCG00000027667	EML1	223.45	-2.09	0.14	-14.46	0.00	0.00
ENSSSCG00000017783		4208.62	-2.09	0.05	-44.93	0.00	0.00
ENSSSCG00000039094	PLEC	15577.26	-2.09	0.04	-52.54	0.00	0.00
ENSSSCG00000011384	BSN	14.61	-2.09	0.55	-3.77	0.00	0.00
ENSSSCG00000011522	CNTN3	53.91	-2.09	0.29	-7.17	0.00	0.00
ENSSSCG00000015071	SIK3	898.24	-2.08	0.08	-26.78	0.00	0.00
ENSSSCG00000030424	USP31	957.60	-2.08	0.08	-25.98	0.00	0.00
ENSSSCG00000003478	CROCC	63.30	-2.08	0.27	-7.86	0.00	0.00
ENSSSCG00000008187	KIAA1211L	117.15	-2.08	0.21	-10.15	0.00	0.00
ENSSSCG00000039769		552.40	-2.08	0.10	-20.96	0.00	0.00
ENSSSCG00000011813	P3H2	1349.86	-2.08	0.07	-31.53	0.00	0.00
ENSSSCG00000011194	ANKRD28	1301.44	-2.08	0.07	-29.54	0.00	0.00
ENSSSCG00000017310	KANSL1	735.06	-2.08	0.09	-23.65	0.00	0.00
ENSSSCG00000000612	ATF7IP	810.68	-2.07	0.08	-25.11	0.00	0.00
ENSSSCG00000005020	ATL1	9.42	-2.07	0.71	-2.92	0.00	0.01
ENSSSCG00000021702	XRRA1	18.60	-2.07	0.49	-4.22	0.00	0.00
ENSSSCG00000033894		23.61	-2.07	0.45	-4.60	0.00	0.00
ENSSSCG00000013246	C11orf49	37.29	-2.07	0.36	-5.77	0.00	0.00
ENSSSCG00000011127	TAF3	236.18	-2.07	0.16	-13.22	0.00	0.00
ENSSSCG00000006927	PKN2	1873.40	-2.06	0.07	-29.62	0.00	0.00
ENSSSCG00000015853	HERC2	1375.91	-2.06	0.07	-31.07	0.00	0.00

ENSSSCG00000011765	USP13	402.30	-2.06	0.11	-18.87	0.00	0.00
ENSSSCG00000008080	PHF2	434.14	-2.06	0.11	-19.40	0.00	0.00
ENSSSCG00000012146	CTPS2	508.22	-2.06	0.11	-19.51	0.00	0.00
ENSSSCG00000039393	SPRED1	619.80	-2.06	0.10	-20.91	0.00	0.00
ENSSSCG00000035798	PRTG	9.21	-2.06	0.71	-2.90	0.00	0.01
ENSSSCG00000020366	RF00406	8.37	-2.06	0.75	-2.75	0.01	0.01
ENSSSCG00000040426		87.55	-2.06	0.24	-8.55	0.00	0.00
ENSSSCG00000004678	DUOX2	8.35	-2.06	0.78	-2.62	0.01	0.01
ENSSSCG00000008576		136.08	-2.06	0.20	-10.10	0.00	0.00
ENSSSCG00000000924	C12orf50	5.02	-2.05	0.94	-2.18	0.03	0.04
ENSSSCG00000000994	GMDS	157.10	-2.05	0.18	-11.50	0.00	0.00
ENSSSCG00000009839	CIT	1196.50	-2.05	0.07	-29.06	0.00	0.00
ENSSSCG00000039408	ADCY7	903.91	-2.05	0.08	-24.43	0.00	0.00
ENSSSCG00000031960		12.62	-2.05	0.60	-3.39	0.00	0.00
ENSSSCG00000004246		83.78	-2.05	0.23	-8.72	0.00	0.00
ENSSSCG00000035887	C8orf34	16.76	-2.05	0.53	-3.89	0.00	0.00
ENSSSCG00000010277	SLC29A3	96.89	-2.04	0.22	-9.10	0.00	0.00
ENSSSCG00000019687	RF00403	10.93	-2.04	0.64	-3.22	0.00	0.00
ENSSSCG00000032775		6.73	-2.04	0.81	-2.54	0.01	0.02
ENSSSCG00000008772	RELL1	2131.21	-2.04	0.06	-35.77	0.00	0.00
ENSSSCG00000027093	FOLH1B	40.09	-2.04	0.34	-6.09	0.00	0.00
ENSSSCG00000024570	KDM4B	281.82	-2.04	0.14	-15.09	0.00	0.00
ENSSSCG00000021784	TBCK	555.91	-2.04	0.10	-20.32	0.00	0.00
ENSSSCG00000037748		24.17	-2.04	0.43	-4.71	0.00	0.00
ENSSSCG00000029843	NPHP4	546.33	-2.04	0.10	-20.65	0.00	0.00

ENSSSCG00000017812	VPS53	653.72	-2.04	0.09	-23.23	0.00	0.00
ENSSSCG00000009249	HNRNPD	2412.81	-2.04	0.07	-31.11	0.00	0.00
ENSSSCG00000014893		305.19	-2.03	0.12	-16.44	0.00	0.00
ENSSSCG00000011074	ARHGAP21	998.88	-2.03	0.07	-27.50	0.00	0.00
ENSSSCG00000032931		4132.96	-2.03	0.05	-42.14	0.00	0.00
ENSSSCG00000005930	SLC45A4	221.96	-2.03	0.15	-13.98	0.00	0.00
ENSSSCG00000007585	ACTB	61149.09	-2.03	0.04	-52.81	0.00	0.00
ENSSSCG00000003811	ROR1	11.55	-2.03	0.62	-3.26	0.00	0.00
ENSSSCG00000010473	EXOC6	317.28	-2.03	0.13	-15.97	0.00	0.00
ENSSSCG00000034569		455.26	-2.03	0.10	-19.59	0.00	0.00
ENSSSCG00000004402	METTL24	24.05	-2.02	0.44	-4.63	0.00	0.00
ENSSSCG00000017527	SKAP1	679.99	-2.02	0.09	-22.67	0.00	0.00
ENSSSCG00000004802	AQR	1097.83	-2.02	0.07	-28.52	0.00	0.00
ENSSSCG00000010563	BTRC	326.01	-2.02	0.12	-16.82	0.00	0.00
ENSSSCG00000031269		94.23	-2.02	0.22	-9.11	0.00	0.00
ENSSSCG00000017298	TANC2	167.39	-2.02	0.17	-12.18	0.00	0.00
ENSSSCG00000021731	WWC2	802.77	-2.01	0.08	-24.78	0.00	0.00
ENSSSCG00000021038	NRIP1	1978.54	-2.01	0.06	-34.20	0.00	0.00
ENSSSCG00000023187		563.65	-2.01	0.12	-17.43	0.00	0.00
ENSSSCG00000023992	CEP112	210.06	-2.01	0.15	-13.34	0.00	0.00
ENSSSCG00000011518	SHQ1	248.29	-2.01	0.14	-14.48	0.00	0.00
ENSSSCG00000009432	DGKD	644.33	-2.01	0.09	-21.38	0.00	0.00
ENSSSCG00000005282		920.31	-2.01	0.09	-21.91	0.00	0.00
ENSSSCG00000003772		1092.40	-2.01	0.07	-28.38	0.00	0.00
ENSSSCG00000011340	SMARCC1	2093.57	-2.01	0.06	-34.87	0.00	0.00

ENSSSCG00000004344	MMS22L	515.14	-2.01	0.10	-19.43	0.00	0.00
ENSSSCG00000005135		3546.11	-2.01	0.05	-39.56	0.00	0.00
ENSSSCG00000038963		652.29	-2.01	0.09	-22.74	0.00	0.00
ENSSSCG00000017000	RANBP17	517.63	-2.01	0.10	-20.43	0.00	0.00
ENSSSCG00000008510	LTBP1	235.82	-2.00	0.14	-14.22	0.00	0.00
ENSSSCG00000033412	B4GALNT3	659.84	-2.00	0.09	-22.13	0.00	0.00
ENSSSCG00000005232	SMARCA2	509.43	-2.00	0.10	-20.48	0.00	0.00
ENSSSCG00000015901	GRB14	106.26	-2.00	0.22	-9.19	0.00	0.00
ENSSSCG00000004029	QKI	1758.92	-2.00	0.06	-31.23	0.00	0.00
ENSSSCG00000014568	ST5	711.78	-2.00	0.09	-21.87	0.00	0.00
ENSSSCG00000001499	DST	5612.78	-2.00	0.05	-43.57	0.00	0.00
ENSSSCG00000005965	MYC	4753.10	-2.00	0.06	-35.99	0.00	0.00
ENSSSCG00000031716	PTPRQ	10.56	-2.00	0.66	-3.03	0.00	0.00
ENSSSCG00000010276	UNC5B	1051.81	-2.00	0.08	-26.54	0.00	0.00
ENSSSCG00000009896	BICDL1	204.64	-2.00	0.15	-12.99	0.00	0.00
ENSSSCG00000028512	LDLR	3070.89	-2.00	0.05	-38.13	0.00	0.00
ENSSSCG00000014249	MARCH3	111.37	-1.99	0.20	-9.80	0.00	0.00
ENSSSCG00000008464	MTA3	110.58	-1.99	0.20	-9.99	0.00	0.00
ENSSSCG00000016330		1106.46	-1.99	0.08	-24.99	0.00	0.00
ENSSSCG00000038401	TRAM2	1710.58	-1.99	0.06	-32.10	0.00	0.00
ENSSSCG00000034156	GTF2F2	816.79	-1.99	0.08	-24.72	0.00	0.00
ENSSSCG00000034491	PRICKLE1	65.79	-1.99	0.26	-7.74	0.00	0.00
ENSSSCG00000006864	CDC14A	294.41	-1.99	0.14	-14.74	0.00	0.00
ENSSSCG00000014387		784.97	-1.99	0.09	-22.09	0.00	0.00
ENSSSCG00000008682	NSD2	2349.29	-1.99	0.05	-37.21	0.00	0.00

ENSSSCG00000015906	CSRNP3	10.60	-1.99	0.65	-3.07	0.00	0.00
ENSSSCG00000016005	SESTD1	282.37	-1.99	0.14	-13.95	0.00	0.00
ENSSSCG00000027812	IQSEC1	319.09	-1.99	0.12	-15.93	0.00	0.00
ENSSSCG00000007153	ATRN	2515.94	-1.99	0.05	-37.10	0.00	0.00
ENSSSCG00000027365	WNT7B	1290.58	-1.99	0.07	-29.94	0.00	0.00
ENSSSCG00000034964	STX8	391.51	-1.98	0.11	-17.85	0.00	0.00
ENSSSCG00000004149	NHSL1	2929.95	-1.98	0.06	-35.49	0.00	0.00
ENSSSCG00000003110		682.03	-1.98	0.09	-23.27	0.00	0.00
ENSSSCG00000010497	ENTPD1	8.05	-1.98	0.75	-2.66	0.01	0.01
ENSSSCG00000024399	EVC2	148.53	-1.98	0.18	-11.05	0.00	0.00
ENSSSCG00000037322		3077.82	-1.98	0.05	-38.01	0.00	0.00
ENSSSCG00000013262	AMBRA1	441.86	-1.98	0.11	-18.39	0.00	0.00
ENSSSCG00000004823	LRRK1	406.13	-1.98	0.11	-17.48	0.00	0.00
ENSSSCG00000009473	MYCBP2	1407.85	-1.98	0.07	-29.66	0.00	0.00
ENSSSCG00000007602	BAIAP2L1	1703.46	-1.98	0.06	-32.67	0.00	0.00
ENSSSCG00000016045	PMS1	614.74	-1.98	0.09	-21.82	0.00	0.00
ENSSSCG00000016898		225.07	-1.98	0.15	-13.27	0.00	0.00
ENSSSCG00000017693	AATF	1805.22	-1.97	0.06	-30.66	0.00	0.00
ENSSSCG00000003107	ARHGAP35	3025.52	-1.97	0.05	-37.59	0.00	0.00
ENSSSCG00000024189	SIL1	298.56	-1.97	0.13	-15.60	0.00	0.00
ENSSSCG00000008326	TGFA	19.27	-1.97	0.48	-4.11	0.00	0.00
ENSSSCG00000004435	NT5DC1	704.95	-1.97	0.09	-22.73	0.00	0.00
ENSSSCG00000012166		1878.88	-1.97	0.06	-31.45	0.00	0.00
ENSSSCG00000020749	KCNAB2	246.54	-1.97	0.14	-14.35	0.00	0.00
ENSSSCG00000016643	TMEM168	305.63	-1.97	0.12	-15.83	0.00	0.00

ENSSSCG00000010052	BCR	998.25	-1.97	0.07	-26.85	0.00	0.00
ENSSSCG00000021285	RNF216	339.32	-1.97	0.12	-16.57	0.00	0.00
ENSSSCG00000019699	RF00279	11.27	-1.97	0.62	-3.18	0.00	0.00
ENSSSCG00000011025	ZEB1	75.67	-1.97	0.25	-7.76	0.00	0.00
ENSSSCG00000033120	PALM2	1359.98	-1.97	0.06	-30.30	0.00	0.00
ENSSSCG00000034871		102.83	-1.97	0.21	-9.18	0.00	0.00
ENSSSCG00000037284	CCDC85C	557.21	-1.96	0.10	-19.90	0.00	0.00
ENSSSCG00000032193	NKAIN1	232.48	-1.96	0.14	-13.81	0.00	0.00
ENSSSCG00000004070	SCAF8	1083.47	-1.96	0.08	-25.42	0.00	0.00
ENSSSCG00000013566	INSR	255.68	-1.96	0.14	-14.20	0.00	0.00
ENSSSCG00000037561	ATP11A	2650.28	-1.96	0.05	-37.93	0.00	0.00
ENSSSCG00000016846	WDR70	278.31	-1.96	0.13	-14.93	0.00	0.00
ENSSSCG00000038202		671.53	-1.96	0.09	-22.02	0.00	0.00
ENSSSCG00000009698	GLRA3	167.72	-1.96	0.17	-11.64	0.00	0.00
ENSSSCG00000019270	MIR10A	11.28	-1.96	0.65	-3.04	0.00	0.00
ENSSSCG00000003699	GREB1L	9.56	-1.96	0.68	-2.90	0.00	0.01
ENSSSCG00000028883	SETD4	15.92	-1.96	0.53	-3.73	0.00	0.00
ENSSSCG00000027447	TMTC1	250.01	-1.96	0.14	-14.14	0.00	0.00
ENSSSCG00000034259	PMEPA1	1619.00	-1.96	0.07	-29.89	0.00	0.00
ENSSSCG00000015960	MAP3K20	788.92	-1.95	0.08	-23.54	0.00	0.00
ENSSSCG00000009071	JADE1	1446.35	-1.95	0.07	-29.08	0.00	0.00
ENSSSCG00000011600	SLC6A6	1278.94	-1.95	0.07	-29.31	0.00	0.00
ENSSSCG00000039049	MAP7	1328.74	-1.95	0.07	-28.19	0.00	0.00
ENSSSCG00000007721	GTF2I	3289.25	-1.95	0.05	-37.93	0.00	0.00
ENSSSCG00000000169	POLR3B	633.84	-1.95	0.09	-20.78	0.00	0.00

ENSSSCG00000037929	HIST1H2BJ	5.54	-1.94	0.88	-2.21	0.03	0.04
ENSSSCG00000018016	MAP2K4	1255.90	-1.94	0.07	-27.46	0.00	0.00
ENSSSCG00000009683	KIF13B	357.23	-1.94	0.11	-16.92	0.00	0.00
ENSSSCG00000004058	EZR	15673.50	-1.94	0.04	-48.22	0.00	0.00
ENSSSCG00000016614	PTPRZ1	1630.51	-1.94	0.06	-30.95	0.00	0.00
ENSSSCG00000003719		234.57	-1.94	0.15	-13.33	0.00	0.00
ENSSSCG00000035823		7.02	-1.94	0.83	-2.33	0.02	0.03
ENSSSCG00000014076	FAM169A	517.16	-1.94	0.10	-19.92	0.00	0.00
ENSSSCG00000012833		24.77	-1.94	0.47	-4.12	0.00	0.00
ENSSSCG00000033469		8.74	-1.94	0.72	-2.68	0.01	0.01
ENSSSCG00000038535	ARSB	188.58	-1.94	0.16	-12.25	0.00	0.00
ENSSSCG00000021026	TMEM51	437.47	-1.94	0.11	-17.75	0.00	0.00
ENSSSCG00000029281	PCDHAC2	161.63	-1.94	0.17	-11.60	0.00	0.00
ENSSSCG00000005090	MNAT1	449.89	-1.93	0.12	-16.57	0.00	0.00
ENSSSCG00000011795	IGF2BP2	1629.44	-1.93	0.06	-31.72	0.00	0.00
ENSSSCG00000009345	PDS5B	1150.37	-1.93	0.08	-24.91	0.00	0.00
ENSSSCG00000039996	FAM168A	456.64	-1.93	0.13	-15.42	0.00	0.00
ENSSSCG00000036124		437.54	-1.93	0.11	-18.18	0.00	0.00
ENSSSCG00000024864	ATXN10	489.60	-1.93	0.10	-19.67	0.00	0.00
ENSSSCG00000008710	JAKMIP1	94.37	-1.93	0.22	-8.71	0.00	0.00
ENSSSCG00000028317	ARHGEF28	604.22	-1.93	0.09	-21.25	0.00	0.00
ENSSSCG00000031175	FNIP2	325.20	-1.93	0.12	-16.09	0.00	0.00
ENSSSCG00000017136	TBCD	1241.25	-1.93	0.07	-27.48	0.00	0.00
ENSSSCG00000004535	TCF4	113.52	-1.93	0.20	-9.61	0.00	0.00
ENSSSCG00000004405	FIG4	376.22	-1.93	0.12	-16.63	0.00	0.00

ENSSSCG00000027280	RF00608	21.99	-1.92	0.45	-4.27	0.00	0.00
ENSSSCG00000021343	ZEB2	169.39	-1.92	0.16	-11.69	0.00	0.00
ENSSSCG00000039514	ID3	495.21	-1.92	0.11	-17.09	0.00	0.00
ENSSSCG00000004896	PHLPP1	4.71	-1.92	0.96	-2.01	0.04	0.06
ENSSSCG00000038447	RF01295	6.23	-1.92	0.95	-2.01	0.04	0.06
ENSSSCG00000000024	PARVB	467.35	-1.92	0.10	-18.63	0.00	0.00
ENSSSCG00000031392		45.07	-1.92	0.32	-6.02	0.00	0.00
ENSSSCG00000007352	DHX35	444.49	-1.92	0.12	-16.23	0.00	0.00
ENSSSCG00000005098	SYT16	121.66	-1.92	0.19	-10.10	0.00	0.00
ENSSSCG00000007719		1006.04	-1.92	0.07	-25.83	0.00	0.00
ENSSSCG00000015379	DNAH11	527.80	-1.91	0.10	-19.18	0.00	0.00
ENSSSCG00000039468	SERPINH1	4110.48	-1.91	0.05	-37.94	0.00	0.00
ENSSSCG00000007027	SLC20A2	579.08	-1.91	0.10	-19.82	0.00	0.00
ENSSSCG00000009595		30.35	-1.91	0.38	-5.00	0.00	0.00
ENSSSCG00000015754	MCPH1	279.62	-1.91	0.13	-14.43	0.00	0.00
ENSSSCG00000034434		6.21	-1.91	0.83	-2.31	0.02	0.03
ENSSSCG00000005466	PTBP3	6063.45	-1.91	0.06	-32.30	0.00	0.00
ENSSSCG00000026722	PHF14	387.96	-1.91	0.11	-16.96	0.00	0.00
ENSSSCG00000025344	PHC2	2624.08	-1.91	0.05	-37.09	0.00	0.00
ENSSSCG00000008712	PPP2R2C	980.84	-1.91	0.09	-20.77	0.00	0.00
ENSSSCG00000014800	RNF121	205.61	-1.91	0.15	-12.84	0.00	0.00
ENSSSCG00000009929	TRPV4	974.38	-1.90	0.07	-26.21	0.00	0.00
ENSSSCG00000019224	RF00606	16.29	-1.90	0.53	-3.58	0.00	0.00
ENSSSCG00000008357	SPRED2	1065.39	-1.90	0.07	-25.57	0.00	0.00
ENSSSCG00000017102	TENT4A	1493.11	-1.90	0.06	-30.04	0.00	0.00

ENSSSCG00000039010	WNT7A	131.55	-1.90	0.18	-10.39	0.00	0.00
ENSSSCG00000015273	ATP2B4	3480.14	-1.90	0.05	-39.43	0.00	0.00
ENSSSCG00000011575	ATG7	305.62	-1.90	0.12	-15.55	0.00	0.00
ENSSSCG00000026078	GLCE	787.41	-1.90	0.08	-23.56	0.00	0.00
ENSSSCG00000037105	EVL	71.96	-1.90	0.25	-7.47	0.00	0.00
ENSSSCG00000013876	MYO9B	1548.68	-1.90	0.07	-28.96	0.00	0.00
ENSSSCG00000015940	UBR3	684.21	-1.90	0.09	-21.93	0.00	0.00
ENSSSCG00000001621	TFEB	133.54	-1.90	0.19	-10.17	0.00	0.00
ENSSSCG00000016794	MYO10	3498.13	-1.90	0.05	-37.29	0.00	0.00
ENSSSCG00000006746	VANGL1	717.53	-1.89	0.09	-21.68	0.00	0.00
ENSSSCG00000005388	INVS	204.33	-1.89	0.15	-12.70	0.00	0.00
ENSSSCG00000033919	DCLK1	94.03	-1.89	0.22	-8.62	0.00	0.00
ENSSSCG00000040457	EPN2	339.41	-1.88	0.12	-16.11	0.00	0.00
ENSSSCG00000004603	RFX7	738.73	-1.88	0.09	-21.29	0.00	0.00
ENSSSCG00000017748	NF1	4014.22	-1.88	0.05	-36.75	0.00	0.00
ENSSSCG00000005191	MPDZ	841.68	-1.88	0.08	-23.49	0.00	0.00
ENSSSCG00000009298	CDK8	857.42	-1.88	0.08	-22.86	0.00	0.00
ENSSSCG00000008820	TEC	201.87	-1.88	0.15	-12.28	0.00	0.00
ENSSSCG00000008756	DHX15	4677.93	-1.88	0.04	-42.78	0.00	0.00
ENSSSCG00000004587	MYO1E	1432.36	-1.88	0.06	-29.28	0.00	0.00
ENSSSCG00000009885	HECTD4	420.92	-1.88	0.11	-17.54	0.00	0.00
ENSSSCG00000003863	ZFYVE9	332.48	-1.88	0.12	-15.12	0.00	0.00
ENSSSCG00000036192	C7orf26	6.89	-1.88	0.79	-2.37	0.02	0.02
ENSSSCG00000016938	IPO11	846.68	-1.88	0.08	-22.70	0.00	0.00
ENSSSCG00000000934	LRRIQ1	139.41	-1.88	0.18	-10.47	0.00	0.00

ENSSSCG00000001421	ZBTB12	35.08	-1.87	0.35	-5.30	0.00	0.00
ENSSSCG00000035967	ZC3H18	1351.38	-1.87	0.07	-28.58	0.00	0.00
ENSSSCG00000026044	FDFT1	805.15	-1.87	0.08	-23.75	0.00	0.00
ENSSSCG00000009681	INTS9	308.85	-1.87	0.13	-14.66	0.00	0.00
ENSSSCG00000017791	SSH2	667.39	-1.87	0.09	-20.91	0.00	0.00
ENSSSCG00000022073	ZBTB38	1469.65	-1.87	0.07	-28.66	0.00	0.00
ENSSSCG00000009300	WASF3	116.89	-1.87	0.19	-9.66	0.00	0.00
ENSSSCG00000017470	TNS4	2027.41	-1.87	0.07	-26.16	0.00	0.00
ENSSSCG00000021495	C3orf33	68.09	-1.86	0.25	-7.36	0.00	0.00
ENSSSCG00000010509	PIK3AP1	4258.07	-1.86	0.05	-34.44	0.00	0.00
ENSSSCG00000015411	PTPN12	5330.83	-1.86	0.05	-36.70	0.00	0.00
ENSSSCG00000008144	NCK2	979.06	-1.86	0.07	-24.93	0.00	0.00
ENSSSCG00000022096	POU3F3	249.44	-1.86	0.14	-13.62	0.00	0.00
ENSSSCG00000010437	PAPSS2	381.83	-1.86	0.11	-16.46	0.00	0.00
ENSSSCG00000017301	TLK2	449.49	-1.85	0.10	-18.24	0.00	0.00
ENSSSCG00000018972	RF00438	14.20	-1.85	0.57	-3.24	0.00	0.00
ENSSSCG00000006240	FAM110B	34.77	-1.85	0.35	-5.27	0.00	0.00
ENSSSCG00000017879		1076.64	-1.85	0.07	-25.89	0.00	0.00
ENSSSCG00000035400	YPEL2	259.57	-1.85	0.16	-11.50	0.00	0.00
ENSSSCG00000038187		1734.10	-1.85	0.07	-26.41	0.00	0.00
ENSSSCG00000023533	ZNF236	319.76	-1.85	0.12	-15.46	0.00	0.00
ENSSSCG00000022197	HS6ST1	1903.27	-1.85	0.06	-29.85	0.00	0.00
ENSSSCG00000036826		10.65	-1.85	0.70	-2.64	0.01	0.01
ENSSSCG00000028755	EIPR1	487.64	-1.85	0.10	-18.26	0.00	0.00
ENSSSCG00000008337	AAK1	466.14	-1.84	0.11	-16.10	0.00	0.00

ENSSSCG00000017804	ABR	3078.54	-1.84	0.05	-36.81	0.00	0.00
ENSSSCG00000011312	LARS2	661.71	-1.84	0.09	-21.14	0.00	0.00
ENSSSCG00000015874	ACVR1	371.98	-1.84	0.11	-16.02	0.00	0.00
ENSSSCG00000036401		480.90	-1.84	0.10	-18.26	0.00	0.00
ENSSSCG00000038928		467.57	-1.84	0.10	-17.77	0.00	0.00
ENSSSCG00000031943		84.61	-1.84	0.23	-7.92	0.00	0.00
ENSSSCG00000001906	CYP1A1	163.16	-1.84	0.18	-10.47	0.00	0.00
ENSSSCG00000040388	ZBTB46	220.77	-1.84	0.15	-12.32	0.00	0.00
ENSSSCG00000005729	DDX31	1061.47	-1.84	0.07	-25.90	0.00	0.00
ENSSSCG00000006496	LMNA	10467.52	-1.83	0.04	-44.46	0.00	0.00
ENSSSCG00000008984	SHROOM3	1923.09	-1.83	0.06	-32.68	0.00	0.00
ENSSSCG00000015931		138.64	-1.83	0.19	-9.62	0.00	0.00
ENSSSCG00000032731	NDST1	700.21	-1.83	0.08	-21.94	0.00	0.00
ENSSSCG00000009250	PRKG2	1577.38	-1.83	0.06	-30.48	0.00	0.00
ENSSSCG00000012463	CHM	672.47	-1.83	0.09	-19.52	0.00	0.00
ENSSSCG00000013079	DAGLA	152.16	-1.83	0.18	-10.07	0.00	0.00
ENSSSCG00000025504	RF00431	8.23	-1.83	0.74	-2.47	0.01	0.02
ENSSSCG00000028355	ACTG1	139958.39	-1.83	0.03	-54.83	0.00	0.00
ENSSSCG00000000549	PPFIBP1	709.16	-1.83	0.09	-20.00	0.00	0.00
ENSSSCG00000014598	PPFIBP2	109.74	-1.82	0.20	-8.99	0.00	0.00
ENSSSCG00000003913	MAST2	763.62	-1.82	0.08	-22.34	0.00	0.00
ENSSSCG00000008445	SRBD1	715.61	-1.82	0.09	-20.96	0.00	0.00
ENSSSCG00000012703	RBMX	4679.78	-1.82	0.05	-39.95	0.00	0.00
ENSSSCG00000009761	NCOR2	2831.80	-1.82	0.05	-34.46	0.00	0.00
ENSSSCG00000006201	ARFGEF1	2666.43	-1.82	0.05	-35.01	0.00	0.00

ENSSSCG00000029598	FAM124A	59.27	-1.82	0.27	-6.76	0.00	0.00
ENSSSCG00000029778	SPATA17	22.12	-1.82	0.44	-4.13	0.00	0.00
ENSSSCG00000029998	KLF7	464.69	-1.82	0.10	-17.38	0.00	0.00
ENSSSCG00000022227	BRD4	1745.03	-1.82	0.07	-27.63	0.00	0.00
ENSSSCG00000040184	LMO7	6678.40	-1.82	0.04	-46.38	0.00	0.00
ENSSSCG00000007541	PDGFA	873.36	-1.81	0.08	-23.68	0.00	0.00
ENSSSCG00000011788	VPS8	810.12	-1.81	0.08	-21.59	0.00	0.00
ENSSSCG00000035987	EHD3	2078.31	-1.81	0.06	-30.13	0.00	0.00
ENSSSCG00000031593	NUDT3	754.34	-1.81	0.08	-22.27	0.00	0.00
ENSSSCG00000023526	RAPGEF3	168.62	-1.81	0.17	-10.68	0.00	0.00
ENSSSCG00000002670	USP10	1974.13	-1.81	0.06	-29.21	0.00	0.00
ENSSSCG00000024084	RF00581	24.09	-1.81	0.43	-4.21	0.00	0.00
ENSSSCG00000000456	SLC16A7	396.03	-1.81	0.11	-16.75	0.00	0.00
ENSSSCG00000005379	GALNT12	273.76	-1.81	0.13	-14.00	0.00	0.00
ENSSSCG00000031744		58.64	-1.81	0.27	-6.57	0.00	0.00
ENSSSCG00000032814	DNAH7	29.32	-1.80	0.39	-4.67	0.00	0.00
ENSSSCG00000007950	ADCY9	155.49	-1.80	0.17	-10.43	0.00	0.00
ENSSSCG00000039085		1466.11	-1.80	0.06	-28.78	0.00	0.00
ENSSSCG00000007733		571.15	-1.80	0.09	-19.03	0.00	0.00
ENSSSCG00000023548	GSTCD	409.76	-1.80	0.11	-17.08	0.00	0.00
ENSSSCG00000016566	NRF1	464.14	-1.80	0.10	-17.77	0.00	0.00
ENSSSCG00000021748	PRR5	439.75	-1.80	0.11	-17.08	0.00	0.00
ENSSSCG00000017624	CUEDC1	677.60	-1.80	0.09	-19.42	0.00	0.00
ENSSSCG00000012884	PPP6R3	1813.81	-1.80	0.06	-28.83	0.00	0.00
ENSSSCG00000023862	MTMR8	123.56	-1.80	0.19	-9.47	0.00	0.00

ENSSSCG00000016497	DENND2A	79.24	-1.80	0.24	-7.38	0.00	0.00
ENSSSCG00000025224	ACYP2	21.09	-1.80	0.46	-3.89	0.00	0.00
ENSSSCG00000002523	CDC42BPB	1624.36	-1.80	0.06	-30.08	0.00	0.00
ENSSSCG00000004460	IBTK	1297.00	-1.80	0.08	-22.15	0.00	0.00
ENSSSCG00000032827	DNAH5	326.86	-1.80	0.12	-15.39	0.00	0.00
ENSSSCG00000009229	ARHGAP24	109.66	-1.80	0.20	-8.94	0.00	0.00
ENSSSCG00000038934		57.06	-1.80	0.27	-6.63	0.00	0.00
ENSSSCG00000015557	NMNAT2	79.63	-1.80	0.24	-7.58	0.00	0.00
ENSSSCG00000005981	FBXO32	872.39	-1.80	0.08	-21.36	0.00	0.00
ENSSSCG00000035581	SUGCT	84.69	-1.79	0.23	-7.93	0.00	0.00
ENSSSCG00000016690	CREB5	133.84	-1.79	0.20	-8.89	0.00	0.00
ENSSSCG00000008340	ANTXR1	90.44	-1.79	0.22	-8.21	0.00	0.00
ENSSSCG00000020702	SENP6	1547.13	-1.79	0.07	-27.03	0.00	0.00
ENSSSCG00000017069	LARP1	5257.90	-1.79	0.05	-39.81	0.00	0.00
ENSSSCG00000020188	RF00090	8.72	-1.79	0.70	-2.55	0.01	0.02
ENSSSCG00000016842	NIPBL	1508.04	-1.79	0.07	-27.49	0.00	0.00
ENSSSCG00000010245	STOX1	126.34	-1.79	0.20	-9.15	0.00	0.00
ENSSSCG00000027211	EPC2	445.25	-1.79	0.11	-15.92	0.00	0.00
ENSSSCG00000002281		250.31	-1.79	0.13	-13.44	0.00	0.00
ENSSSCG00000030130	DPYSL2	1503.14	-1.79	0.07	-27.39	0.00	0.00
ENSSSCG00000040847	AP1S3	965.46	-1.79	0.08	-22.14	0.00	0.00
ENSSSCG00000034074		58.89	-1.79	0.28	-6.48	0.00	0.00
ENSSSCG00000031367		35.56	-1.78	0.35	-5.10	0.00	0.00
ENSSSCG00000002965	ACTN4	8161.94	-1.78	0.04	-42.26	0.00	0.00
ENSSSCG00000006346	ATF6	3467.34	-1.78	0.05	-37.25	0.00	0.00

ENSSSCG00000026006	KLF13	194.35	-1.78	0.15	-11.71	0.00	0.00
ENSSSCG00000032728	EFNB1	1837.75	-1.78	0.06	-29.13	0.00	0.00
ENSSSCG00000026433	CCDC73	20.33	-1.78	0.46	-3.87	0.00	0.00
ENSSSCG00000015523	RALGPS2	425.20	-1.78	0.11	-16.36	0.00	0.00
ENSSSCG00000000570	C2CD5	699.29	-1.78	0.09	-19.86	0.00	0.00
ENSSSCG00000013938	GATAD2A	3398.73	-1.78	0.05	-35.90	0.00	0.00
ENSSSCG00000023141	RNLS	10.27	-1.78	0.71	-2.50	0.01	0.02
ENSSSCG00000035854	RAB11FIP3	309.76	-1.78	0.13	-13.43	0.00	0.00
ENSSSCG00000001968	NUBPL	179.21	-1.78	0.16	-11.21	0.00	0.00
ENSSSCG00000002330	PCNX1	1009.74	-1.78	0.08	-22.56	0.00	0.00
ENSSSCG00000015507	TNN	83.86	-1.78	0.22	-7.91	0.00	0.00
ENSSSCG00000017116	LPCAT1	1008.48	-1.78	0.08	-21.88	0.00	0.00
ENSSSCG00000023894	AKNA	96.87	-1.77	0.21	-8.37	0.00	0.00
ENSSSCG00000010316	KAT6B	131.85	-1.77	0.18	-9.74	0.00	0.00
ENSSSCG00000037451	PPFIA1	1235.95	-1.77	0.07	-25.81	0.00	0.00
ENSSSCG00000012103		454.89	-1.77	0.80	-2.22	0.03	0.04
ENSSSCG00000025260	CARD10	1442.65	-1.77	0.06	-27.36	0.00	0.00
ENSSSCG00000018039	NCOR1	2577.46	-1.77	0.05	-32.92	0.00	0.00
ENSSSCG00000032996	SLC7A5	6220.67	-1.76	0.05	-32.51	0.00	0.00
ENSSSCG00000011471	FLNB	10522.74	-1.76	0.04	-41.48	0.00	0.00
ENSSSCG00000015509	COP1	370.87	-1.76	0.11	-15.72	0.00	0.00
ENSSSCG00000037547	SLC17A3	15.75	-1.76	0.51	-3.44	0.00	0.00
ENSSSCG00000000587	AEBP2	411.89	-1.76	0.11	-15.90	0.00	0.00
ENSSSCG00000011394	RBM6	861.71	-1.76	0.08	-23.25	0.00	0.00
ENSSSCG00000009370	FOXO1	434.20	-1.76	0.11	-16.47	0.00	0.00

ENSSSCG00000033574	CFAP54	92.66	-1.76	0.22	-8.06	0.00	0.00
ENSSSCG00000022998	PKIG	396.29	-1.76	0.12	-14.78	0.00	0.00
ENSSSCG00000010432		65.86	-1.76	0.26	-6.67	0.00	0.00
ENSSSCG00000011260	SCN10A	10.71	-1.76	0.63	-2.80	0.01	0.01
ENSSSCG00000013534	MLLT1	1596.50	-1.75	0.07	-25.92	0.00	0.00
ENSSSCG00000010544	DNMBP	223.02	-1.75	0.14	-12.13	0.00	0.00
ENSSSCG00000008190	INPP4A	336.70	-1.75	0.12	-15.06	0.00	0.00
ENSSSCG00000040418	FSIP1	47.73	-1.75	0.30	-5.84	0.00	0.00
ENSSSCG00000001710	RUNX2	170.51	-1.75	0.16	-11.06	0.00	0.00
ENSSSCG00000030198		599.55	-1.75	0.09	-19.37	0.00	0.00
ENSSSCG00000005386	STX17	259.99	-1.75	0.13	-13.41	0.00	0.00
ENSSSCG00000004832	UBE3A	1671.24	-1.75	0.06	-27.45	0.00	0.00
ENSSSCG00000038178	FUT10	250.84	-1.75	0.14	-12.84	0.00	0.00
ENSSSCG00000013303	ABTB2	199.02	-1.75	0.16	-11.12	0.00	0.00
ENSSSCG00000040350		1914.81	-1.75	0.06	-28.59	0.00	0.00
ENSSSCG00000006025	PKHD1L1	14.91	-1.75	0.53	-3.29	0.00	0.00
ENSSSCG00000029633	USP24	2294.31	-1.75	0.05	-32.36	0.00	0.00
ENSSSCG00000038177	SPATA16	17.16	-1.75	0.51	-3.45	0.00	0.00
ENSSSCG00000002134	RNASE10	6.35	-1.75	0.81	-2.15	0.03	0.04
ENSSSCG00000004857	ATP9B	138.07	-1.75	0.18	-9.90	0.00	0.00
ENSSSCG00000021657	SATB2	254.87	-1.74	0.14	-12.59	0.00	0.00
ENSSSCG00000037608	LRRC70	12.08	-1.74	0.58	-2.98	0.00	0.00
ENSSSCG00000034364	SPECC1	859.63	-1.74	0.08	-22.54	0.00	0.00
ENSSSCG00000006885	ALG14	195.57	-1.74	0.15	-11.75	0.00	0.00
ENSSSCG00000010396	PARG	658.24	-1.74	0.09	-19.75	0.00	0.00

ENSSSCG00000007512		120.99	-1.74	0.19	-9.26	0.00	0.00
ENSSSCG00000011120	USP6NL	544.41	-1.74	0.10	-17.93	0.00	0.00
ENSSSCG00000016531		114.63	-1.74	0.20	-8.74	0.00	0.00
ENSSSCG00000035230	PIEZO1	2019.26	-1.74	0.05	-31.70	0.00	0.00
ENSSSCG00000002451	RIN3	18.48	-1.73	0.48	-3.59	0.00	0.00
ENSSSCG00000028420	EIF4E	1752.71	-1.73	0.07	-25.50	0.00	0.00
ENSSSCG00000014794	NUP98	3883.76	-1.73	0.05	-37.40	0.00	0.00
ENSSSCG00000007700	HIP1	159.87	-1.73	0.17	-10.30	0.00	0.00
ENSSSCG00000023112	PDS5A	3838.91	-1.73	0.06	-30.87	0.00	0.00
ENSSSCG00000015961	CDCA7	1976.10	-1.73	0.06	-29.20	0.00	0.00
ENSSSCG00000010186	URB2	1215.77	-1.73	0.07	-25.94	0.00	0.00
ENSSSCG00000037826	RF00593	10.54	-1.73	0.68	-2.56	0.01	0.02
ENSSSCG00000006730	MAN1A2	1599.28	-1.73	0.06	-27.82	0.00	0.00
ENSSSCG00000006053	BAALC	61.13	-1.73	0.26	-6.52	0.00	0.00
ENSSSCG00000009018	SH3D19	390.70	-1.73	0.11	-15.96	0.00	0.00
ENSSSCG00000008771	C4orf19	127.50	-1.73	0.18	-9.46	0.00	0.00
ENSSSCG00000034493	ST3GAL6	425.12	-1.73	0.11	-16.34	0.00	0.00
ENSSSCG00000004558	CSNK1G1	506.35	-1.72	0.10	-17.79	0.00	0.00
ENSSSCG00000038646		2090.82	-1.72	0.06	-28.44	0.00	0.00
ENSSSCG00000015963	OLA1	2102.44	-1.72	0.05	-31.38	0.00	0.00
ENSSSCG00000004033	AGPAT4	99.59	-1.72	0.21	-8.32	0.00	0.00
ENSSSCG00000016291	GIGYF2	1312.41	-1.72	0.06	-26.77	0.00	0.00
ENSSSCG00000001810	PDE8A	1116.16	-1.72	0.07	-24.18	0.00	0.00
ENSSSCG00000020695		347.70	-1.72	0.11	-14.96	0.00	0.00
ENSSSCG00000035900	CENPB	1795.45	-1.72	0.06	-29.60	0.00	0.00

ENSSSCG00000021612	ARHGAP28	23.82	-1.72	0.41	-4.15	0.00	0.00
ENSSSCG00000011162	LARP4B	789.61	-1.71	0.09	-20.01	0.00	0.00
ENSSSCG00000010289	MICU1	2102.79	-1.71	0.06	-28.82	0.00	0.00
ENSSSCG00000029943	SCLT1	185.32	-1.71	0.15	-11.11	0.00	0.00
ENSSSCG00000007135	NINL	374.84	-1.71	0.11	-15.26	0.00	0.00
ENSSSCG00000016148	PTH2R	25.15	-1.71	0.41	-4.20	0.00	0.00
ENSSSCG00000027859	HDAC7	1239.39	-1.71	0.07	-25.41	0.00	0.00
ENSSSCG00000026041	MAP3K5	378.53	-1.71	0.13	-13.54	0.00	0.00
ENSSSCG00000010941		191.61	-1.71	0.16	-10.76	0.00	0.00
ENSSSCG00000001549	FKBP5	2405.51	-1.71	0.05	-33.26	0.00	0.00
ENSSSCG00000015037	IL18	225.79	-1.71	0.17	-9.83	0.00	0.00
ENSSSCG00000029573	NASP	3170.88	-1.71	0.05	-35.83	0.00	0.00
ENSSSCG00000001792	EFL1	1392.43	-1.70	0.06	-26.52	0.00	0.00
ENSSSCG00000000036	PACSIN2	1659.24	-1.70	0.06	-28.00	0.00	0.00
ENSSSCG00000004368	PREP	1053.71	-1.70	0.07	-23.51	0.00	0.00
ENSSSCG00000004465	BCKDHB	145.41	-1.70	0.17	-9.80	0.00	0.00
ENSSSCG00000016725	TNS3	13.27	-1.70	0.56	-3.04	0.00	0.00
ENSSSCG00000040476		5.53	-1.70	0.86	-1.97	0.05	0.06
ENSSSCG00000027207	GPATCH2	187.94	-1.70	0.15	-11.21	0.00	0.00
ENSSSCG00000025993	SGPP2	801.14	-1.70	0.10	-17.79	0.00	0.00
ENSSSCG00000022353	RAP1GDS1	1648.46	-1.70	0.06	-28.11	0.00	0.00
ENSSSCG00000016747	NUDCD3	1012.52	-1.69	0.07	-22.78	0.00	0.00
ENSSSCG00000035273		239.55	-1.69	0.14	-11.68	0.00	0.00
ENSSSCG00000010430	CSTF2T	1226.23	-1.69	0.07	-22.87	0.00	0.00
ENSSSCG00000033602	UBE2K	1265.14	-1.69	0.07	-24.40	0.00	0.00

ENSSSCG00000023204	AXIN1	574.61	-1.69	0.10	-17.80	0.00	0.00
ENSSSCG00000007282	PIGU	756.49	-1.69	0.08	-20.12	0.00	0.00
ENSSSCG00000010872	AKT3	221.36	-1.69	0.14	-12.01	0.00	0.00
ENSSSCG00000009551	ARHGEF7	691.31	-1.69	0.09	-18.36	0.00	0.00
ENSSSCG00000037671	PACS2	1425.63	-1.69	0.08	-21.92	0.00	0.00
ENSSSCG00000024158	ANO1	53.17	-1.69	0.28	-6.03	0.00	0.00
ENSSSCG00000029275	PPARGC1A	197.36	-1.69	0.15	-11.01	0.00	0.00
ENSSSCG00000008768	ARAP2	210.80	-1.69	0.15	-11.41	0.00	0.00
ENSSSCG00000028740	CTDSPL	161.91	-1.69	0.16	-10.34	0.00	0.00
ENSSSCG00000035357	BCL2L1	1675.55	-1.69	0.06	-27.60	0.00	0.00
ENSSSCG00000005446	EPB41L4B	194.39	-1.69	0.15	-11.14	0.00	0.00
ENSSSCG00000005235	KANK1	1786.52	-1.68	0.07	-25.15	0.00	0.00
ENSSSCG00000031232	RF00271	15.72	-1.68	0.52	-3.22	0.00	0.00
ENSSSCG00000007576	FOXK1	890.08	-1.68	0.08	-21.14	0.00	0.00
ENSSSCG00000015586	RPS6KC1	1126.51	-1.68	0.07	-24.05	0.00	0.00
ENSSSCG00000001646	BICRAL	271.65	-1.68	0.13	-13.05	0.00	0.00
ENSSSCG00000005373	NANS	6464.67	-1.68	0.04	-41.43	0.00	0.00
ENSSSCG00000005389	TEX10	846.81	-1.68	0.08	-21.64	0.00	0.00
ENSSSCG00000010290	MCU	5245.36	-1.68	0.04	-39.66	0.00	0.00
ENSSSCG00000030685	ARID2	982.52	-1.68	0.08	-21.17	0.00	0.00
ENSSSCG00000018450	RF00609	6.89	-1.68	0.80	-2.11	0.04	0.05
ENSSSCG00000011042	RSU1	680.12	-1.68	0.09	-18.10	0.00	0.00
ENSSSCG00000016295	NGEF	42.56	-1.68	0.31	-5.42	0.00	0.00
ENSSSCG00000003134	GRWD1	1665.55	-1.68	0.06	-28.56	0.00	0.00
ENSSSCG00000014876	MYO7A	607.57	-1.68	0.09	-18.44	0.00	0.00

ENSSSCG00000033032	ZBTB14	183.69	-1.68	0.15	-11.08	0.00	0.00
ENSSSCG00000014450	TCOF1	2250.05	-1.68	0.05	-30.99	0.00	0.00
ENSSSCG00000016943	ADAMTS6	41.73	-1.68	0.32	-5.30	0.00	0.00
ENSSSCG00000006214	RRS1	677.84	-1.67	0.09	-19.39	0.00	0.00
ENSSSCG00000017043	RNF145	1605.57	-1.67	0.07	-25.43	0.00	0.00
ENSSSCG00000024998	PCDHA11	112.18	-1.67	0.20	-8.41	0.00	0.00
ENSSSCG00000037470	RF00156	9.53	-1.67	0.66	-2.53	0.01	0.02
ENSSSCG00000028855	GMPS	2212.97	-1.67	0.06	-28.47	0.00	0.00
ENSSSCG00000000277	NPFF	9.55	-1.67	0.65	-2.55	0.01	0.02
ENSSSCG00000029578	NFATC3	461.68	-1.67	0.10	-16.57	0.00	0.00
ENSSSCG00000007874	ABCC1	513.05	-1.67	0.10	-17.22	0.00	0.00
ENSSSCG00000012856	NAP1L4	3386.12	-1.67	0.05	-34.81	0.00	0.00
ENSSSCG00000005715	PRRC2B	3152.79	-1.67	0.06	-26.45	0.00	0.00
ENSSSCG00000016769	CDK13	663.46	-1.67	0.09	-19.54	0.00	0.00
ENSSSCG00000014303	JADE2	801.85	-1.67	0.08	-20.81	0.00	0.00
ENSSSCG00000015569	SWT1	95.21	-1.66	0.21	-8.01	0.00	0.00
ENSSSCG00000001078	MBOAT1	572.27	-1.66	0.09	-18.25	0.00	0.00
ENSSSCG00000012602		623.96	-1.66	0.09	-18.56	0.00	0.00
ENSSSCG00000008515	MEMO1	899.54	-1.66	0.08	-21.61	0.00	0.00
ENSSSCG00000002135	PNP	6302.82	-1.66	0.04	-36.85	0.00	0.00
ENSSSCG00000011911	DRD3	18.20	-1.66	0.50	-3.34	0.00	0.00
ENSSSCG00000000869	UTP20	1758.89	-1.66	0.06	-26.07	0.00	0.00
ENSSSCG00000002623	GSTA2	783.54	-1.65	0.27	-6.11	0.00	0.00
ENSSSCG00000039473		897.99	-1.65	0.08	-21.36	0.00	0.00
ENSSSCG00000024134	MGLL	908.66	-1.65	0.08	-20.48	0.00	0.00

ENSSSCG00000010850		219.97	-1.65	0.15	-11.13	0.00	0.00
ENSSSCG00000023907	AFAP1	226.23	-1.65	0.14	-11.82	0.00	0.00
ENSSSCG00000032804		54.84	-1.65	0.28	-5.93	0.00	0.00
ENSSSCG00000012328	HUWE1	13009.27	-1.65	0.04	-41.84	0.00	0.00
ENSSSCG00000025610	AGAP3	392.44	-1.65	0.11	-14.63	0.00	0.00
ENSSSCG00000028018	EP400	2091.37	-1.65	0.06	-29.61	0.00	0.00
ENSSSCG00000004294	SYNCRIP	12180.83	-1.65	0.04	-39.99	0.00	0.00
ENSSSCG00000014242	ZNF608	157.65	-1.65	0.16	-10.08	0.00	0.00
ENSSSCG00000005078	DAAM1	373.36	-1.64	0.12	-14.24	0.00	0.00
ENSSSCG00000002815	ADGRG1	1953.87	-1.64	0.07	-23.28	0.00	0.00
ENSSSCG00000037847	MOB2	251.51	-1.64	0.13	-12.37	0.00	0.00
ENSSSCG00000004861	TSHZ1	56.65	-1.64	0.27	-6.02	0.00	0.00
ENSSSCG00000023266	FOXJ3	1194.83	-1.64	0.07	-23.30	0.00	0.00
ENSSSCG00000021669	MARK3	1224.28	-1.64	0.07	-23.58	0.00	0.00
ENSSSCG00000012634	DOCK11	10.02	-1.64	0.64	-2.55	0.01	0.02
ENSSSCG00000001966	ARHGAP5	1061.55	-1.64	0.08	-20.33	0.00	0.00
ENSSSCG00000002654	FBXO31	696.92	-1.64	0.08	-19.76	0.00	0.00
ENSSSCG00000036379		38.91	-1.64	0.35	-4.70	0.00	0.00
ENSSSCG00000028878	BCAR1	3048.11	-1.64	0.05	-30.21	0.00	0.00
ENSSSCG00000039311	NCS1	684.28	-1.64	0.08	-19.25	0.00	0.00
ENSSSCG00000010053		626.14	-1.64	0.09	-18.09	0.00	0.00
ENSSSCG00000007909	ABAT	9.37	-1.63	0.69	-2.38	0.02	0.02
ENSSSCG00000011102	NRP1	41.55	-1.63	0.31	-5.21	0.00	0.00
ENSSSCG00000017756	NLK	799.42	-1.63	0.08	-20.50	0.00	0.00
ENSSSCG00000008179	REV1	416.40	-1.63	0.10	-15.67	0.00	0.00

ENSSSCG00000015345	GLCCI1	270.94	-1.63	0.13	-12.35	0.00	0.00
ENSSSCG00000009238	COQ2	740.41	-1.63	0.08	-20.20	0.00	0.00
ENSSSCG00000011741	GOLIM4	1351.31	-1.63	0.06	-25.66	0.00	0.00
ENSSSCG00000036537	NFIC	1236.20	-1.63	0.07	-24.52	0.00	0.00
ENSSSCG00000022312	RHPN2	2392.32	-1.63	0.05	-30.36	0.00	0.00
ENSSSCG00000016956	MAST4	89.01	-1.63	0.22	-7.52	0.00	0.00
ENSSSCG00000033893		6.70	-1.63	0.78	-2.08	0.04	0.05
ENSSSCG00000005524	DAB2IP	914.11	-1.63	0.08	-21.05	0.00	0.00
ENSSSCG00000028047	DTNA	186.23	-1.63	0.16	-10.19	0.00	0.00
ENSSSCG00000036156	RBM12	618.79	-1.63	0.10	-16.92	0.00	0.00
ENSSSCG00000004370	ATG5	872.18	-1.62	0.08	-21.50	0.00	0.00
ENSSSCG00000025067		712.71	-1.62	0.08	-19.57	0.00	0.00
ENSSSCG00000014884		656.54	-1.62	0.09	-19.01	0.00	0.00
ENSSSCG00000004113	STXBP5	476.51	-1.62	0.10	-15.72	0.00	0.00
ENSSSCG00000005205	KIAA2026	297.30	-1.62	0.12	-13.24	0.00	0.00
ENSSSCG00000031488	ARHGAP17	547.29	-1.62	0.10	-16.57	0.00	0.00
ENSSSCG00000009956	GRK3	197.07	-1.62	0.15	-11.07	0.00	0.00
ENSSSCG00000026268	DLG5	1383.82	-1.62	0.07	-24.89	0.00	0.00
ENSSSCG00000005975	MTSS1	102.34	-1.62	0.22	-7.47	0.00	0.00
ENSSSCG00000040215	TFAP4	214.44	-1.62	0.14	-11.26	0.00	0.00
ENSSSCG00000005979	ANXA13	1993.75	-1.62	0.06	-26.30	0.00	0.00
ENSSSCG00000032176	SMURF2	1035.75	-1.62	0.09	-18.55	0.00	0.00
ENSSSCG00000008319	CD207	77.13	-1.62	0.24	-6.70	0.00	0.00
ENSSSCG00000017957	EIF4A1	22071.72	-1.62	0.04	-39.49	0.00	0.00
ENSSSCG00000012262	KDM6A	155.58	-1.62	0.16	-9.85	0.00	0.00

ENSSSCG00000002305	EXD2	254.14	-1.62	0.14	-11.80	0.00	0.00
ENSSSCG00000009293		227.23	-1.61	0.16	-10.32	0.00	0.00
ENSSSCG00000034786	HACD2	1173.45	-1.61	0.07	-21.53	0.00	0.00
ENSSSCG00000037101	LRRC8B	151.92	-1.61	0.17	-9.37	0.00	0.00
ENSSSCG00000040288	ARNT2	176.79	-1.61	0.17	-9.43	0.00	0.00
ENSSSCG00000006137	WWP1	661.31	-1.61	0.09	-18.48	0.00	0.00
ENSSSCG00000000423	LRP1	3241.18	-1.61	0.05	-34.28	0.00	0.00
ENSSSCG00000001532	UHRF1BP1	594.23	-1.61	0.09	-17.95	0.00	0.00
ENSSSCG00000009106	PDE5A	693.53	-1.61	0.09	-18.58	0.00	0.00
ENSSSCG00000022609	WWP2	676.51	-1.61	0.09	-18.38	0.00	0.00
ENSSSCG00000008989	CNOT6L	505.80	-1.60	0.10	-16.11	0.00	0.00
ENSSSCG00000010476	MYOF	16447.18	-1.60	0.04	-42.62	0.00	0.00
ENSSSCG00000016494	BRAF	576.96	-1.60	0.09	-17.80	0.00	0.00
ENSSSCG00000005711	NUP214	1872.29	-1.60	0.06	-26.71	0.00	0.00
ENSSSCG00000013522	SAFB	2697.67	-1.60	0.05	-30.72	0.00	0.00
ENSSSCG00000038989		9654.40	-1.60	0.05	-31.97	0.00	0.00
ENSSSCG00000006069	RGS22	8.55	-1.59	0.69	-2.32	0.02	0.03
ENSSSCG00000011858	ZNF148	675.82	-1.59	0.09	-17.99	0.00	0.00
ENSSSCG00000027489	TMCO4	143.70	-1.59	0.18	-8.91	0.00	0.00
ENSSSCG00000032422		232.90	-1.59	0.14	-11.37	0.00	0.00
ENSSSCG00000034725	UBE2R2	591.76	-1.59	0.09	-18.03	0.00	0.00
ENSSSCG00000014410	SH3RF2	329.98	-1.59	0.11	-13.91	0.00	0.00
ENSSSCG00000011896	ADPRH	141.73	-1.59	0.18	-8.98	0.00	0.00
ENSSSCG00000037381	WRB	699.60	-1.59	0.08	-19.16	0.00	0.00
ENSSSCG00000006062	YWHAZ	13689.13	-1.59	0.03	-45.50	0.00	0.00

ENSSSCG00000007812	XPO6	3022.34	-1.59	0.05	-29.89	0.00	0.00
ENSSSCG00000024623	USP25	756.55	-1.59	0.08	-18.78	0.00	0.00
ENSSSCG00000032673	ZFP14	11.69	-1.59	0.62	-2.56	0.01	0.02
ENSSSCG00000002525	TRAF3	860.75	-1.59	0.08	-20.72	0.00	0.00
ENSSSCG00000031121		879.89	-1.58	0.08	-20.93	0.00	0.00
ENSSSCG00000012257	MAOA	76.36	-1.58	0.24	-6.71	0.00	0.00
ENSSSCG00000003413	MTOR	1875.47	-1.58	0.06	-26.77	0.00	0.00
ENSSSCG00000031557	CHCHD6	171.15	-1.58	0.16	-10.05	0.00	0.00
ENSSSCG00000003743	GALNT1	2125.18	-1.58	0.06	-26.47	0.00	0.00
ENSSSCG00000029125	FAM13B	412.13	-1.58	0.10	-15.25	0.00	0.00
ENSSSCG00000009516	UBAC2	338.43	-1.58	0.12	-13.45	0.00	0.00
ENSSSCG00000022479	UBR5	4605.64	-1.58	0.04	-35.89	0.00	0.00
ENSSSCG00000002508	SETD3	978.71	-1.58	0.07	-21.25	0.00	0.00
ENSSSCG00000017561	ABCC3	5215.44	-1.58	0.04	-36.82	0.00	0.00
ENSSSCG00000007814	KIAA0556	26.61	-1.58	0.40	-3.99	0.00	0.00
ENSSSCG00000016887	ITGA2	4370.54	-1.58	0.05	-29.45	0.00	0.00
ENSSSCG00000012054	DOP1B	475.75	-1.58	0.10	-15.66	0.00	0.00
ENSSSCG00000004472	IRAK1BP1	86.89	-1.57	0.22	-7.22	0.00	0.00
ENSSSCG00000027722	BMP2K	898.94	-1.57	0.08	-20.41	0.00	0.00
ENSSSCG00000037898	CXXC5	194.83	-1.57	0.15	-10.37	0.00	0.00
ENSSSCG00000023032	SCG2	9.71	-1.57	0.64	-2.45	0.01	0.02
ENSSSCG00000006850	FAM102B	4054.34	-1.57	0.05	-34.25	0.00	0.00
ENSSSCG00000015955	ITGA6	5642.01	-1.57	0.04	-35.77	0.00	0.00
ENSSSCG00000015555	LAMC1	10602.94	-1.57	0.04	-41.41	0.00	0.00
ENSSSCG00000037482		122.97	-1.57	0.19	-8.06	0.00	0.00

ENSSSCG00000024373	TRIP12	3263.00	-1.57	0.05	-33.28	0.00	0.00
ENSSSCG00000006889	ARHGAP29	3927.94	-1.57	0.05	-33.47	0.00	0.00
ENSSSCG00000013984	SNAP47	128.09	-1.57	0.18	-8.54	0.00	0.00
ENSSSCG00000017125	FOXK2	2440.50	-1.57	0.05	-30.98	0.00	0.00
ENSSSCG00000031441	GRAMD4	118.16	-1.57	0.19	-8.10	0.00	0.00
ENSSSCG00000007839	EEF2K	1003.25	-1.57	0.07	-21.68	0.00	0.00
ENSSSCG00000014829	MRPL48	433.67	-1.56	0.11	-14.57	0.00	0.00
ENSSSCG00000015099	BCL9L	4594.47	-1.56	0.05	-30.38	0.00	0.00
ENSSSCG00000034356		424.28	-1.56	0.10	-15.03	0.00	0.00
ENSSSCG00000003384	DNAJC11	1128.78	-1.56	0.07	-22.64	0.00	0.00
ENSSSCG00000013629	SMARCA4	3543.72	-1.56	0.05	-33.69	0.00	0.00
ENSSSCG00000000811	PCED1B	47.09	-1.56	0.29	-5.28	0.00	0.00
ENSSSCG00000009714	NEK1	389.88	-1.56	0.11	-14.44	0.00	0.00
ENSSSCG00000020152	RF00554	12.23	-1.56	0.57	-2.75	0.01	0.01
ENSSSCG00000000078	TNRC6B	639.67	-1.55	0.09	-17.20	0.00	0.00
ENSSSCG00000006344	NOS1AP	440.41	-1.55	0.10	-15.31	0.00	0.00
ENSSSCG00000010521	UBTD1	296.40	-1.55	0.12	-12.80	0.00	0.00
ENSSSCG00000037621	KIAA1522	1374.97	-1.55	0.07	-21.84	0.00	0.00
ENSSSCG00000010566	FBXW4	130.37	-1.55	0.22	-7.19	0.00	0.00
ENSSSCG00000027675		736.11	-1.55	0.09	-18.10	0.00	0.00
ENSSSCG00000023188	ARIH1	1288.30	-1.55	0.07	-23.50	0.00	0.00
ENSSSCG00000038089	COL18A1	1443.73	-1.55	0.06	-25.63	0.00	0.00
ENSSSCG00000031077	KPNA4	1568.12	-1.55	0.07	-23.48	0.00	0.00
ENSSSCG00000027348		357.88	-1.55	0.11	-14.03	0.00	0.00
ENSSSCG00000029783	MKX	15.42	-1.55	0.51	-3.06	0.00	0.00

ENSSSCG00000039688		177.13	-1.55	0.15	-10.00	0.00	0.00
ENSSSCG00000010440		1206.19	-1.55	0.08	-19.74	0.00	0.00
ENSSSCG00000012676	MBNL3	102.09	-1.55	0.20	-7.57	0.00	0.00
ENSSSCG00000011594	NR2C2	386.08	-1.55	0.12	-13.14	0.00	0.00
ENSSSCG00000035062		399.21	-1.55	0.11	-14.56	0.00	0.00
ENSSSCG00000035086		337.68	-1.55	0.12	-13.18	0.00	0.00
ENSSSCG00000003859	TUT4	593.58	-1.55	0.09	-16.93	0.00	0.00
ENSSSCG00000027846	RRP15	668.12	-1.54	0.08	-18.55	0.00	0.00
ENSSSCG00000004795	MEIS2	38.99	-1.54	0.34	-4.51	0.00	0.00
ENSSSCG00000004941	DIS3L	78.12	-1.54	0.24	-6.46	0.00	0.00
ENSSSCG00000018698	ssc-mir-221	10.90	-1.54	0.61	-2.54	0.01	0.02
ENSSSCG00000038483		70.40	-1.54	0.26	-5.97	0.00	0.00
ENSSSCG00000014578	DENND5A	1293.59	-1.54	0.07	-20.69	0.00	0.00
ENSSSCG00000022039		44.74	-1.54	0.30	-5.09	0.00	0.00
ENSSSCG00000032734	VOPP1	1017.47	-1.54	0.07	-22.08	0.00	0.00
ENSSSCG00000024372	RF00151	9.55	-1.54	0.65	-2.37	0.02	0.03
ENSSSCG00000014919	ME3	263.46	-1.54	0.13	-11.71	0.00	0.00
ENSSSCG00000003881	SPATA6	357.05	-1.54	0.11	-13.53	0.00	0.00
ENSSSCG00000040629	IL34	72.93	-1.53	0.24	-6.37	0.00	0.00
ENSSSCG00000017728	MYO1D	749.27	-1.53	0.08	-19.01	0.00	0.00
ENSSSCG00000020879	FBXW7	428.64	-1.53	0.10	-15.01	0.00	0.00
ENSSSCG00000021068	TRAF5	426.91	-1.53	0.10	-14.87	0.00	0.00
ENSSSCG00000015534	ACBD6	739.93	-1.53	0.08	-19.31	0.00	0.00
ENSSSCG00000012584	CAPN6	743.78	-1.53	0.09	-17.27	0.00	0.00
ENSSSCG00000035403	RFX2	275.41	-1.53	0.13	-12.05	0.00	0.00

ENSSSCG00000032590		20.54	-1.53	0.48	-3.20	0.00	0.00
ENSSSCG00000014965	ENDOD1	1888.27	-1.53	0.06	-25.72	0.00	0.00
ENSSSCG00000004136	AIG1	241.52	-1.52	0.14	-10.80	0.00	0.00
ENSSSCG00000000152	RBFOX2	852.58	-1.52	0.08	-19.18	0.00	0.00
ENSSSCG00000008169	TBC1D8	644.54	-1.52	0.11	-13.46	0.00	0.00
ENSSSCG00000010313	VCL	4366.72	-1.52	0.04	-35.69	0.00	0.00
ENSSSCG00000009148	LEF1	1068.65	-1.52	0.08	-19.00	0.00	0.00
ENSSSCG00000000981	CRELD2	696.01	-1.52	0.08	-18.42	0.00	0.00
ENSSSCG00000010894	TP53BP2	2559.36	-1.52	0.05	-29.08	0.00	0.00
ENSSSCG00000002412	STON2	23.89	-1.52	0.43	-3.54	0.00	0.00
ENSSSCG00000009887	NAA25	1580.27	-1.52	0.06	-24.57	0.00	0.00
ENSSSCG00000013421	PTBP1	11545.58	-1.52	0.04	-37.44	0.00	0.00
ENSSSCG00000024125	KPNA3	2098.14	-1.52	0.06	-25.54	0.00	0.00
ENSSSCG00000005459	ECPAS	2822.65	-1.52	0.05	-29.02	0.00	0.00
ENSSSCG00000015892	PSMD14	2194.51	-1.52	0.05	-27.98	0.00	0.00
ENSSSCG00000038640	RF00026	12.56	-1.51	0.57	-2.66	0.01	0.01
ENSSSCG00000027525	DHCR24	2134.21	-1.51	0.06	-24.62	0.00	0.00
ENSSSCG00000040178	E2F3	484.89	-1.51	0.10	-15.59	0.00	0.00
ENSSSCG00000021893	ROCK1	1834.18	-1.51	0.07	-23.06	0.00	0.00
ENSSSCG00000007981	NPRL3	276.79	-1.51	0.13	-11.97	0.00	0.00
ENSSSCG00000004762	DNAJC17	213.73	-1.51	0.14	-10.65	0.00	0.00
ENSSSCG00000009994	MTMR3	597.91	-1.51	0.09	-17.19	0.00	0.00
ENSSSCG00000001869	PEAK1	207.52	-1.51	0.14	-10.56	0.00	0.00
ENSSSCG00000002787	E2F4	2073.19	-1.51	0.06	-27.27	0.00	0.00
ENSSSCG00000015283	PIK3C2B	342.79	-1.51	0.12	-13.09	0.00	0.00

ENSSSCG00000016717	MPP6	397.12	-1.51	0.11	-14.09	0.00	0.00
ENSSSCG00000007166	PTPRA	1433.76	-1.51	0.06	-24.57	0.00	0.00
ENSSSCG00000008449	SLC3A1	1363.09	-1.51	0.06	-23.84	0.00	0.00
ENSSSCG00000009674	ELP3	616.66	-1.51	0.09	-16.77	0.00	0.00
ENSSSCG00000000860	NUP37	185.11	-1.51	0.15	-9.86	0.00	0.00
ENSSSCG00000000985	EXOC2	691.40	-1.51	0.08	-18.18	0.00	0.00
ENSSSCG00000021890	GNB1	4853.37	-1.51	0.04	-35.34	0.00	0.00
ENSSSCG00000003790	ANKRD13C	601.52	-1.51	0.09	-15.94	0.00	0.00
ENSSSCG00000006508	ASH1L	2358.07	-1.50	0.06	-24.21	0.00	0.00
ENSSSCG00000009522	PCCA	262.46	-1.50	0.13	-11.74	0.00	0.00
ENSSSCG00000003973	CTPS1	2002.92	-1.50	0.06	-26.53	0.00	0.00
ENSSSCG00000014993		194.39	-1.50	0.15	-10.13	0.00	0.00
ENSSSCG00000021738		810.04	-1.50	0.08	-18.97	0.00	0.00
ENSSSCG00000003749	PIK3C3	382.63	-1.50	0.11	-13.43	0.00	0.00
ENSSSCG00000022302	BICRA	97.96	-1.50	0.22	-6.73	0.00	0.00
ENSSSCG00000015988	HNRNPA3	3729.02	-1.50	0.05	-30.26	0.00	0.00
ENSSSCG00000016772	VPS41	891.95	-1.50	0.07	-20.33	0.00	0.00
ENSSSCG00000006790	WDR77	2766.40	-1.50	0.05	-27.57	0.00	0.00
ENSSSCG00000002386	IFT43	49.43	-1.50	0.30	-5.08	0.00	0.00
ENSSSCG00000010801	CDC73	1226.81	-1.50	0.06	-23.29	0.00	0.00
ENSSSCG00000005012	ARF6	6286.54	-1.50	0.05	-32.40	0.00	0.00
ENSSSCG00000025413		17.37	-1.50	0.49	-3.08	0.00	0.00
ENSSSCG00000014567	TRIM66	207.62	-1.50	0.14	-10.51	0.00	0.00
ENSSSCG00000008738	CC2D2A	71.69	-1.49	0.24	-6.21	0.00	0.00
ENSSSCG00000017602	STXBP4	138.13	-1.49	0.17	-8.59	0.00	0.00

ENSSSCG00000000736	TEAD4	521.42	-1.49	0.09	-15.80	0.00	0.00
ENSSSCG00000003718	TAF4B	608.60	-1.49	0.09	-16.67	0.00	0.00
ENSSSCG00000023983	AGPS	2167.36	-1.49	0.06	-26.91	0.00	0.00
ENSSSCG00000003712	OSBPL1A	232.20	-1.49	0.14	-10.97	0.00	0.00
ENSSSCG00000038763	CERK	416.40	-1.49	0.10	-14.62	0.00	0.00
ENSSSCG00000002718	FA2H	446.14	-1.49	0.12	-12.49	0.00	0.00
ENSSSCG00000011217	NEK10	94.01	-1.49	0.21	-6.95	0.00	0.00
ENSSSCG00000036909	ERI3	182.82	-1.49	0.15	-9.68	0.00	0.00
ENSSSCG00000026962	A4GALT	1139.96	-1.49	0.08	-18.99	0.00	0.00
ENSSSCG00000004814	LRRC28	758.11	-1.49	0.08	-18.30	0.00	0.00
ENSSSCG00000016174	FN1	185464.38	-1.49	0.03	-46.92	0.00	0.00
ENSSSCG00000004199	EPB41L2	1189.11	-1.49	0.07	-22.36	0.00	0.00
ENSSSCG00000004854	TJP1	3834.78	-1.49	0.04	-33.18	0.00	0.00
ENSSSCG00000004203	L3MBTL3	258.18	-1.49	0.14	-10.88	0.00	0.00
ENSSSCG00000010579	GBF1	1519.41	-1.49	0.06	-24.66	0.00	0.00
ENSSSCG00000012637	KLHL13	12.38	-1.48	0.57	-2.58	0.01	0.01
ENSSSCG00000032340		711.05	-1.48	0.08	-17.47	0.00	0.00
ENSSSCG00000014970	MTMR2	3464.23	-1.48	0.05	-32.08	0.00	0.00
ENSSSCG00000000779	KIF21A	688.57	-1.48	0.09	-17.23	0.00	0.00
ENSSSCG00000031429	YAP1	1447.44	-1.48	0.07	-21.98	0.00	0.00
ENSSSCG00000029668	IL2RB	952.22	-1.48	0.07	-19.72	0.00	0.00
ENSSSCG00000022778	SGF29	370.41	-1.48	0.11	-13.47	0.00	0.00
ENSSSCG00000033528	BPTF	1886.84	-1.48	0.06	-25.75	0.00	0.00
ENSSSCG00000009519	CLYBL	137.88	-1.47	0.18	-8.25	0.00	0.00
ENSSSCG00000038557		120.48	-1.47	0.19	-7.81	0.00	0.00

ENSSSCG00000004205	ARHGAP18	1232.69	-1.47	0.09	-17.07	0.00	0.00
ENSSSCG00000021354	CNOT4	646.00	-1.47	0.09	-17.06	0.00	0.00
ENSSSCG00000017877	ANKFY1	1566.97	-1.47	0.06	-25.04	0.00	0.00
ENSSSCG00000001493	PRIM2	295.26	-1.47	0.12	-12.37	0.00	0.00
ENSSSCG00000038977	RSBN1L	1189.85	-1.47	0.07	-20.00	0.00	0.00
ENSSSCG00000009664	PTK2B	583.44	-1.47	0.09	-15.63	0.00	0.00
ENSSSCG00000015455	CUL1	2672.25	-1.47	0.05	-29.46	0.00	0.00
ENSSSCG00000035657	DIP2A	218.87	-1.47	0.14	-10.30	0.00	0.00
ENSSSCG00000008629	ROCK2	1551.33	-1.47	0.07	-21.96	0.00	0.00
ENSSSCG00000014132		96.65	-1.47	0.21	-7.10	0.00	0.00
ENSSSCG00000033983	RRP7A	873.95	-1.47	0.07	-19.81	0.00	0.00
ENSSSCG00000032007	RTN4	4790.80	-1.47	0.04	-34.45	0.00	0.00
ENSSSCG00000036155	FAT4	170.85	-1.47	0.16	-9.38	0.00	0.00
ENSSSCG00000015861	WDR33	2339.16	-1.47	0.05	-28.26	0.00	0.00
ENSSSCG00000007311	PHF20	1130.17	-1.47	0.08	-19.13	0.00	0.00
ENSSSCG00000040528		1064.05	-1.47	0.07	-20.60	0.00	0.00
ENSSSCG00000012197		29.48	-1.46	0.36	-4.03	0.00	0.00
ENSSSCG00000016608	IQUB	74.95	-1.46	0.24	-6.11	0.00	0.00
ENSSSCG00000004138	HIVEP2	1363.94	-1.46	0.07	-21.82	0.00	0.00
ENSSSCG00000016809	DROSHA	1106.98	-1.46	0.07	-19.56	0.00	0.00
ENSSSCG00000009657	PPP2R2A	1875.45	-1.46	0.06	-25.40	0.00	0.00
ENSSSCG00000030420	GLG1	1493.61	-1.46	0.06	-23.98	0.00	0.00
ENSSSCG00000026092	SREBF2	2723.01	-1.46	0.05	-27.14	0.00	0.00
ENSSSCG00000000753	WNK1	3315.85	-1.46	0.05	-30.62	0.00	0.00
ENSSSCG00000025821	C2CD2	794.79	-1.46	0.08	-18.52	0.00	0.00

ENSSSCG00000039577	HEATR3	1100.53	-1.46	0.07	-19.63	0.00	0.00
ENSSSCG00000008192	TMEM131	3624.06	-1.45	0.05	-29.19	0.00	0.00
ENSSSCG00000009061	NAA15	2993.82	-1.45	0.06	-25.22	0.00	0.00
ENSSSCG00000025992	ENPP3	22.61	-1.45	0.43	-3.37	0.00	0.00
ENSSSCG00000032552		1860.76	-1.45	0.06	-23.15	0.00	0.00
ENSSSCG00000016119		619.31	-1.45	0.09	-16.74	0.00	0.00
ENSSSCG00000009692		470.55	-1.45	0.10	-14.94	0.00	0.00
ENSSSCG00000014031		8753.03	-1.45	0.04	-32.63	0.00	0.00
ENSSSCG00000008740	FBXL5	1626.57	-1.45	0.06	-24.58	0.00	0.00
ENSSSCG00000011540	SETD5	2256.78	-1.45	0.07	-22.04	0.00	0.00
ENSSSCG00000009319	PAN3	556.03	-1.45	0.09	-15.97	0.00	0.00
ENSSSCG00000014049	UNC5A	389.12	-1.45	0.11	-13.23	0.00	0.00
ENSSSCG00000016409	UBE3C	1239.21	-1.45	0.07	-21.98	0.00	0.00
ENSSSCG00000005124	IFT74	440.93	-1.45	0.10	-14.24	0.00	0.00
ENSSSCG00000006274	PRKDC	1281.59	-1.45	0.07	-21.93	0.00	0.00
ENSSSCG00000009137	GAR1	478.06	-1.45	0.10	-15.04	0.00	0.00
ENSSSCG00000005894	ARHGAP39	256.09	-1.44	0.13	-11.05	0.00	0.00
ENSSSCG00000002682	MBTPS1	1724.03	-1.44	0.06	-24.27	0.00	0.00
ENSSSCG00000032171	TCF20	1052.32	-1.44	0.08	-18.05	0.00	0.00
ENSSSCG00000014364	ANKHD1	2287.72	-1.44	0.05	-26.45	0.00	0.00
ENSSSCG00000010987	UBAP2	1349.48	-1.44	0.06	-22.24	0.00	0.00
ENSSSCG00000033786		1145.83	-1.44	0.07	-20.63	0.00	0.00
ENSSSCG00000022317	SLC38A10	1400.92	-1.44	0.07	-21.70	0.00	0.00
ENSSSCG00000008713	KIAA0232	362.96	-1.44	0.11	-13.03	0.00	0.00
ENSSSCG00000023130	ASCC1	580.99	-1.44	0.09	-15.86	0.00	0.00

ENSSSCG00000034501	RIOX2	1364.04	-1.44	0.06	-22.33	0.00	0.00
ENSSSCG00000004044	IGF2R	4634.54	-1.44	0.05	-30.68	0.00	0.00
ENSSSCG00000016625	CTTNBP2	674.56	-1.44	0.08	-17.36	0.00	0.00
ENSSSCG00000024149	ELOVL5	1927.47	-1.44	0.06	-22.49	0.00	0.00
ENSSSCG00000022178		822.69	-1.44	0.08	-18.54	0.00	0.00
ENSSSCG00000008119	KCNIP3	149.01	-1.44	0.17	-8.55	0.00	0.00
ENSSSCG00000016882	PARP8	603.49	-1.44	0.09	-16.22	0.00	0.00
ENSSSCG00000000896	NTN4	923.87	-1.44	0.08	-18.87	0.00	0.00
ENSSSCG00000032761	LAMA5	16599.58	-1.44	0.04	-37.50	0.00	0.00
ENSSSCG00000038594	SDC4	7203.73	-1.44	0.05	-28.27	0.00	0.00
ENSSSCG00000023593	AFF1	383.88	-1.43	0.12	-11.75	0.00	0.00
ENSSSCG00000009505	MBNL2	597.80	-1.43	0.09	-15.45	0.00	0.00
ENSSSCG00000030305	SLC15A2	12.75	-1.43	0.56	-2.57	0.01	0.01
ENSSSCG00000037530		64.43	-1.43	0.27	-5.40	0.00	0.00
ENSSSCG00000026004	MSRB3	2708.55	-1.43	0.05	-29.21	0.00	0.00
ENSSSCG00000030957	NFKB1	1019.17	-1.43	0.07	-19.52	0.00	0.00
ENSSSCG00000029030	SMCHD1	949.33	-1.43	0.08	-17.30	0.00	0.00
ENSSSCG00000014081	COL4A3BP	314.58	-1.43	0.12	-12.10	0.00	0.00
ENSSSCG00000003679	PPP4R1	1603.08	-1.43	0.06	-23.12	0.00	0.00
ENSSSCG00000012157	PHKA2	92.72	-1.43	0.21	-6.75	0.00	0.00
ENSSSCG00000005510	PHF19	1083.07	-1.43	0.07	-20.80	0.00	0.00
ENSSSCG00000015859	SAP130	1148.69	-1.43	0.07	-21.49	0.00	0.00
ENSSSCG00000008789	WDR19	284.92	-1.43	0.13	-11.26	0.00	0.00
ENSSSCG00000022534	ZNF644	920.81	-1.43	0.07	-19.71	0.00	0.00
ENSSSCG00000003591	PUM1	1885.67	-1.43	0.06	-25.02	0.00	0.00

ENSSSCG00000008762	STIM2	103.38	-1.43	0.21	-6.65	0.00	0.00
ENSSSCG00000010957	AGTPBP1	133.18	-1.42	0.18	-8.11	0.00	0.00
ENSSSCG00000006179		500.87	-1.42	0.10	-14.99	0.00	0.00
ENSSSCG00000003235		20.43	-1.42	0.45	-3.18	0.00	0.00
ENSSSCG00000012084	PRDM15	182.79	-1.42	0.17	-8.52	0.00	0.00
ENSSSCG00000010589	SFXN2	502.96	-1.42	0.09	-14.98	0.00	0.00
ENSSSCG00000017142	BAIAP2	598.89	-1.42	0.09	-15.45	0.00	0.00
ENSSSCG00000013602	HNRNPM	5144.09	-1.42	0.04	-32.42	0.00	0.00
ENSSSCG00000014831	PAAF1	196.83	-1.42	0.15	-9.80	0.00	0.00
ENSSSCG00000037558	YY1	1380.10	-1.42	0.06	-23.08	0.00	0.00
ENSSSCG00000011236		820.95	-1.42	0.08	-17.58	0.00	0.00
ENSSSCG00000030626	ALDH1L1	83.38	-1.42	0.22	-6.50	0.00	0.00
ENSSSCG00000013630	DNM2	2244.95	-1.42	0.05	-26.57	0.00	0.00
ENSSSCG00000009917	SPPL3	264.55	-1.42	0.13	-11.09	0.00	0.00
ENSSSCG00000038870	TNRC6A	1666.41	-1.41	0.06	-24.16	0.00	0.00
ENSSSCG00000016810	PDZD2	95.25	-1.41	0.22	-6.47	0.00	0.00
ENSSSCG00000038539		10.66	-1.41	0.63	-2.23	0.03	0.04
ENSSSCG00000008831	DCUN1D4	497.61	-1.41	0.09	-14.90	0.00	0.00
ENSSSCG00000040056		3245.91	-1.41	0.05	-30.53	0.00	0.00
ENSSSCG00000006571	INTS3	1519.70	-1.41	0.06	-21.91	0.00	0.00
ENSSSCG00000010160	TARBP1	1047.05	-1.41	0.07	-20.62	0.00	0.00
ENSSSCG00000037354	MMACHC	143.31	-1.41	0.17	-8.35	0.00	0.00
ENSSSCG00000022575	PRMT2	232.84	-1.41	0.13	-10.58	0.00	0.00
ENSSSCG00000001925	ADPGK	697.92	-1.41	0.08	-17.09	0.00	0.00
ENSSSCG00000029553		57.21	-1.41	0.26	-5.35	0.00	0.00

ENSSSCG00000003377	ACOT7	660.62	-1.41	0.09	-15.52	0.00	0.00
ENSSSCG00000008796	RBM47	1333.33	-1.41	0.07	-21.36	0.00	0.00
ENSSSCG00000015023		930.50	-1.41	0.07	-19.70	0.00	0.00
ENSSSCG00000032377	RALGAPA1	563.56	-1.41	0.09	-15.44	0.00	0.00
ENSSSCG00000036686	CDC42SE2	1094.22	-1.41	0.07	-20.96	0.00	0.00
ENSSSCG00000039062		16.81	-1.41	0.50	-2.80	0.01	0.01
ENSSSCG00000009759	SCARB1	1628.94	-1.40	0.06	-22.72	0.00	0.00
ENSSSCG00000037977	ALMS1	986.33	-1.40	0.07	-19.19	0.00	0.00
ENSSSCG00000006310	POU2F1	169.26	-1.40	0.16	-8.81	0.00	0.00
ENSSSCG00000037252		17.95	-1.40	0.61	-2.32	0.02	0.03
ENSSSCG00000016626	CFTR	469.89	-1.40	0.10	-14.33	0.00	0.00
ENSSSCG00000011663	RBP2	80.86	-1.40	0.23	-5.98	0.00	0.00
ENSSSCG00000038207	ssc-mir-503	17.26	-1.40	0.48	-2.92	0.00	0.01
ENSSSCG00000038777	C7orf50	189.32	-1.40	0.15	-9.29	0.00	0.00
ENSSSCG00000000784	LRRK2	410.49	-1.40	0.10	-13.60	0.00	0.00
ENSSSCG00000012569	ATG4A	71.37	-1.40	0.24	-5.89	0.00	0.00
ENSSSCG00000003168	ALDH16A1	578.56	-1.40	0.09	-15.55	0.00	0.00
ENSSSCG00000038856	TRIM8	1234.87	-1.40	0.07	-19.92	0.00	0.00
ENSSSCG00000038300	ALDOB	21.45	-1.40	0.46	-3.06	0.00	0.00
ENSSSCG00000023788	TBC1D30	402.82	-1.40	0.11	-13.10	0.00	0.00
ENSSSCG00000011889	GSK3B	1903.16	-1.40	0.05	-25.42	0.00	0.00
ENSSSCG00000003795	WLS	1755.80	-1.40	0.06	-24.81	0.00	0.00
ENSSSCG00000017790	TAOK1	1205.74	-1.40	0.08	-18.04	0.00	0.00
ENSSSCG00000008376	COMMD1	89.36	-1.39	0.21	-6.61	0.00	0.00
ENSSSCG00000026063	DPP9	1371.45	-1.39	0.06	-22.75	0.00	0.00

ENSSSCG00000031997		206.86	-1.39	0.15	-9.10	0.00	0.00
ENSSSCG00000014822	ARHGEF17	378.68	-1.39	0.11	-12.93	0.00	0.00
ENSSSCG00000000502	CNOT2	1409.46	-1.39	0.06	-22.04	0.00	0.00
ENSSSCG00000034768	CYTH3	225.00	-1.39	0.14	-9.96	0.00	0.00
ENSSSCG00000011166	ZMYND11	1003.78	-1.39	0.07	-19.65	0.00	0.00
ENSSSCG00000012996	CDC42EP2	888.44	-1.39	0.08	-18.14	0.00	0.00
ENSSSCG00000015908	TTC21B	460.06	-1.39	0.10	-13.84	0.00	0.00
ENSSSCG00000036801	C6orf132	1283.67	-1.39	0.07	-20.11	0.00	0.00
ENSSSCG00000035824	PBRM1	1412.71	-1.39	0.06	-22.92	0.00	0.00
ENSSSCG00000017201	UNK	546.34	-1.39	0.10	-14.52	0.00	0.00
ENSSSCG00000012160	SH3KBP1	519.22	-1.39	0.09	-15.01	0.00	0.00
ENSSSCG00000005598	SCAI	132.70	-1.39	0.18	-7.81	0.00	0.00
ENSSSCG00000008041	PKD1	905.59	-1.39	0.08	-18.27	0.00	0.00
ENSSSCG00000014435	CSNK1A1	3673.54	-1.39	0.05	-29.71	0.00	0.00
ENSSSCG00000004661	MYEF2	1889.48	-1.39	0.06	-22.27	0.00	0.00
ENSSSCG00000022164		259.65	-1.39	0.13	-10.63	0.00	0.00
ENSSSCG00000037274	PTMA	26075.47	-1.39	0.04	-39.27	0.00	0.00
ENSSSCG00000017504	CDK12	3350.83	-1.38	0.05	-29.81	0.00	0.00
ENSSSCG00000016134	FASTKD2	682.78	-1.38	0.09	-15.67	0.00	0.00
ENSSSCG00000013155		2959.94	-1.38	0.05	-27.15	0.00	0.00
ENSSSCG00000016548		3751.15	-1.38	0.05	-28.28	0.00	0.00
ENSSSCG00000009860	FBXW8	264.52	-1.38	0.13	-10.33	0.00	0.00
ENSSSCG00000022159	FNDC3A	782.32	-1.38	0.09	-15.54	0.00	0.00
ENSSSCG00000009971	ZNRF3	164.04	-1.38	0.16	-8.73	0.00	0.00
ENSSSCG00000014401	NR3C1	366.57	-1.38	0.12	-11.81	0.00	0.00

ENSSSCG00000005992	SHAS2	37.01	-1.38	0.34	-4.10	0.00	0.00
ENSSSCG00000000262	SPRYD3	1174.88	-1.38	0.07	-18.75	0.00	0.00
ENSSSCG00000034736		14.05	-1.38	0.55	-2.50	0.01	0.02
ENSSSCG00000016417	RBM33	592.93	-1.38	0.10	-14.38	0.00	0.00
ENSSSCG00000009567	RASA3	54.49	-1.38	0.28	-4.99	0.00	0.00
ENSSSCG00000023045	BAHCC1	344.47	-1.38	0.11	-12.28	0.00	0.00
ENSSSCG00000004739	MGA	489.06	-1.38	0.10	-13.69	0.00	0.00
ENSSSCG00000015045	NCAM1	25.26	-1.38	0.41	-3.38	0.00	0.00
ENSSSCG00000029593	FOXN2	341.26	-1.37	0.12	-11.54	0.00	0.00
ENSSSCG00000003807	DNAJC6	645.85	-1.37	0.09	-14.87	0.00	0.00
ENSSSCG00000007907	USP7	3478.34	-1.37	0.05	-28.89	0.00	0.00
ENSSSCG00000022830	KANSL1L	71.89	-1.37	0.26	-5.29	0.00	0.00
ENSSSCG00000014882	RSF1	560.37	-1.37	0.09	-15.16	0.00	0.00
ENSSSCG00000014307	H2AFY	2892.41	-1.37	0.05	-26.86	0.00	0.00
ENSSSCG00000035676	G3BP1	6581.37	-1.37	0.04	-32.21	0.00	0.00
ENSSSCG00000017836	PAFAH1B1	3286.13	-1.37	0.05	-25.23	0.00	0.00
ENSSSCG00000014331	KDM3B	121.28	-1.37	0.19	-7.39	0.00	0.00
ENSSSCG00000003760	TTLL7	133.79	-1.37	0.17	-7.87	0.00	0.00
ENSSSCG00000008575	ASXL2	1123.18	-1.37	0.07	-20.08	0.00	0.00
ENSSSCG00000009114	PRSS12	70.82	-1.37	0.24	-5.78	0.00	0.00
ENSSSCG00000025910	ZNF277	573.18	-1.37	0.10	-14.15	0.00	0.00
ENSSSCG00000000793	PPHLN1	753.57	-1.37	0.08	-17.24	0.00	0.00
ENSSSCG00000009308	LNX2	240.90	-1.37	0.14	-10.12	0.00	0.00
ENSSSCG00000009208		388.45	-1.37	0.11	-12.04	0.00	0.00
ENSSSCG00000035733	PLXNB2	20295.45	-1.36	0.04	-33.36	0.00	0.00

ENSSSCG00000015314	ANKIB1	2011.61	-1.36	0.06	-24.45	0.00	0.00
ENSSSCG00000006468	HDGF	5532.72	-1.36	0.05	-25.81	0.00	0.00
ENSSSCG00000007818	KDM8	138.64	-1.36	0.18	-7.77	0.00	0.00
ENSSSCG00000034742	MECP2	281.39	-1.36	0.13	-10.86	0.00	0.00
ENSSSCG00000003771		2057.88	-1.36	0.05	-24.96	0.00	0.00
ENSSSCG00000019069	RF00592	92.85	-1.36	0.21	-6.41	0.00	0.00
ENSSSCG00000011336	ELP6	183.02	-1.36	0.16	-8.74	0.00	0.00
ENSSSCG00000022618	CMTM8	221.76	-1.36	0.14	-10.02	0.00	0.00
ENSSSCG00000026466	SLC23A2	2821.60	-1.36	0.05	-27.92	0.00	0.00
ENSSSCG00000001921	NEO1	959.46	-1.36	0.08	-17.17	0.00	0.00
ENSSSCG00000016234	CUL3	464.80	-1.36	0.10	-13.33	0.00	0.00
ENSSSCG00000029869	RF00325	8.69	-1.36	0.67	-2.03	0.04	0.06
ENSSSCG00000020743	RABGAP1	763.19	-1.36	0.08	-16.30	0.00	0.00
ENSSSCG00000020864	VDR	835.08	-1.36	0.08	-17.26	0.00	0.00
ENSSSCG00000013468	THOP1	1288.98	-1.36	0.06	-21.28	0.00	0.00
ENSSSCG00000021322		845.67	-1.36	0.08	-17.44	0.00	0.00
ENSSSCG00000008697	HTT	2756.65	-1.35	0.05	-26.78	0.00	0.00
ENSSSCG00000010023	PLA2G3	113.96	-1.35	0.19	-6.96	0.00	0.00
ENSSSCG00000011615	RAB7A	5772.94	-1.35	0.05	-27.44	0.00	0.00
ENSSSCG00000008143	UXS1	1380.32	-1.35	0.07	-20.07	0.00	0.00
ENSSSCG00000029163	BCAT1	2535.96	-1.35	0.06	-21.61	0.00	0.00
ENSSSCG00000037413	KBTBD7	81.63	-1.35	0.22	-6.09	0.00	0.00
ENSSSCG00000022656	SRFBP1	486.95	-1.35	0.10	-13.91	0.00	0.00
ENSSSCG00000005287	PSAT1	1533.49	-1.35	0.06	-21.35	0.00	0.00
ENSSSCG00000024621	KAT2B	13.85	-1.35	0.55	-2.45	0.01	0.02

ENSSSCG00000013233	CELF1	2775.17	-1.35	0.06	-24.38	0.00	0.00
ENSSSCG00000040452	TMEM241	171.70	-1.35	0.16	-8.56	0.00	0.00
ENSSSCG00000003485	RCC2	2446.61	-1.35	0.05	-25.26	0.00	0.00
ENSSSCG00000009974	EWSR1	5455.39	-1.35	0.05	-29.33	0.00	0.00
ENSSSCG00000022536	SLC37A2	19343.17	-1.35	0.04	-31.53	0.00	0.00
ENSSSCG00000037905	ITGB5	374.10	-1.35	0.11	-12.16	0.00	0.00
ENSSSCG00000033987		595.48	-1.35	0.09	-15.19	0.00	0.00
ENSSSCG00000033946	DIDO1	1216.07	-1.34	0.06	-20.89	0.00	0.00
ENSSSCG00000027723		465.93	-1.34	0.10	-13.69	0.00	0.00
ENSSSCG00000010118	HIRA	1714.39	-1.34	0.06	-23.42	0.00	0.00
ENSSSCG00000022528	DAZAP1	2475.05	-1.34	0.05	-26.06	0.00	0.00
ENSSSCG00000005291	TLE1	1043.79	-1.34	0.07	-18.96	0.00	0.00
ENSSSCG00000008858	KLHL2	429.10	-1.34	0.10	-13.17	0.00	0.00
ENSSSCG00000004608	DNAAF4	38.67	-1.34	0.33	-4.13	0.00	0.00
ENSSSCG00000003847	DMRTB1	9.92	-1.34	0.65	-2.07	0.04	0.05
ENSSSCG00000010357	WAPL	2346.63	-1.34	0.05	-25.64	0.00	0.00
ENSSSCG00000022015	RF00604	36.96	-1.34	0.35	-3.88	0.00	0.00
ENSSSCG00000028513	VKORC1L1	946.83	-1.34	0.07	-17.86	0.00	0.00
ENSSSCG00000000600	EPS8	5572.12	-1.34	0.04	-31.67	0.00	0.00
ENSSSCG00000000862	GNPTAB	1254.58	-1.34	0.07	-18.70	0.00	0.00
ENSSSCG00000000625	LRP6	705.10	-1.34	0.09	-15.43	0.00	0.00
ENSSSCG00000035730	ZDHHC21	709.19	-1.34	0.09	-15.56	0.00	0.00
ENSSSCG00000025514	RUFY3	362.90	-1.34	0.11	-12.33	0.00	0.00
ENSSSCG00000024018	SLC16A3	2634.56	-1.34	0.07	-19.53	0.00	0.00
ENSSSCG00000023880	RRP8	1256.25	-1.34	0.07	-18.82	0.00	0.00

ENSSSCG00000002245	KATNBL1	499.35	-1.34	0.09	-14.12	0.00	0.00
ENSSSCG00000016974	MAP1B	5650.87	-1.34	0.04	-32.78	0.00	0.00
ENSSSCG00000020835	EIF4EBP3	22.01	-1.33	0.44	-3.04	0.00	0.00
ENSSSCG00000012332	WNK3	435.41	-1.33	0.10	-13.13	0.00	0.00
ENSSSCG00000038156	TMED7	2639.76	-1.33	0.05	-24.47	0.00	0.00
ENSSSCG00000014101	AP3B1	1402.15	-1.33	0.07	-19.77	0.00	0.00
ENSSSCG00000023307	FBXW11	2893.62	-1.33	0.05	-26.38	0.00	0.00
ENSSSCG00000040494	RPL3	11977.16	-1.33	0.04	-30.90	0.00	0.00
ENSSSCG00000035852	CSNK1D	3647.02	-1.33	0.05	-25.83	0.00	0.00
ENSSSCG00000036610	ARMC8	439.45	-1.33	0.10	-13.32	0.00	0.00
ENSSSCG00000038873	SLMAP	1456.72	-1.33	0.07	-20.23	0.00	0.00
ENSSSCG00000011069	ARMC4	141.14	-1.33	0.17	-7.68	0.00	0.00
ENSSSCG00000021975		71.84	-1.33	0.24	-5.61	0.00	0.00
ENSSSCG00000015864	MYO7B	3453.11	-1.33	0.05	-27.17	0.00	0.00
ENSSSCG00000013883	COLGALT1	2169.30	-1.33	0.05	-24.39	0.00	0.00
ENSSSCG00000010543	ABCC2	3706.44	-1.33	0.05	-27.46	0.00	0.00
ENSSSCG00000014958	FUT4	186.49	-1.33	0.15	-8.96	0.00	0.00
ENSSSCG00000005217	RCL1	786.67	-1.33	0.08	-15.89	0.00	0.00
ENSSSCG00000026383	NRP2	1334.24	-1.33	0.06	-21.11	0.00	0.00
ENSSSCG00000011850		6513.89	-1.33	0.05	-26.41	0.00	0.00
ENSSSCG00000016272	PSMD1	3253.99	-1.33	0.05	-26.82	0.00	0.00
ENSSSCG00000016420	INSIG1	410.51	-1.33	0.13	-10.57	0.00	0.00
ENSSSCG00000004075	RGS17	56.12	-1.33	0.29	-4.63	0.00	0.00
ENSSSCG00000017165		1055.19	-1.33	0.07	-18.47	0.00	0.00
ENSSSCG00000011843	PAK2	4502.53	-1.33	0.05	-24.30	0.00	0.00

ENSSSCG00000017376	MEOX1	601.64	-1.32	0.09	-14.93	0.00	0.00
ENSSSCG00000034242	HNF4G	65.75	-1.32	0.24	-5.46	0.00	0.00
ENSSSCG00000009793	CLIP1	2113.21	-1.32	0.06	-23.32	0.00	0.00
ENSSSCG00000004357	SIM1	177.03	-1.32	0.15	-8.59	0.00	0.00
ENSSSCG00000005753	CAMSAP1	966.36	-1.32	0.07	-18.93	0.00	0.00
ENSSSCG00000025209		10.88	-1.32	0.61	-2.17	0.03	0.04
ENSSSCG00000002755	NFAT5	1857.23	-1.32	0.06	-21.72	0.00	0.00
ENSSSCG00000027063	DPY19L1	1835.87	-1.32	0.06	-21.27	0.00	0.00
ENSSSCG00000040089	TEDC1	270.31	-1.32	0.13	-10.37	0.00	0.00
ENSSSCG00000004394	RPF2	1011.71	-1.32	0.07	-18.28	0.00	0.00
ENSSSCG00000017084	FAT2	36.44	-1.32	0.33	-3.98	0.00	0.00
ENSSSCG00000006793		314.23	-1.32	0.13	-10.16	0.00	0.00
ENSSSCG00000028983	TBC1D1	967.19	-1.32	0.08	-17.48	0.00	0.00
ENSSSCG00000004578	ANXA2	36299.71	-1.32	0.04	-36.98	0.00	0.00
ENSSSCG00000029226	UBC	3399.94	-1.32	0.29	-4.52	0.00	0.00
ENSSSCG00000010904	NEK7	407.08	-1.32	0.10	-12.86	0.00	0.00
ENSSSCG00000010485	TBC1D12	329.91	-1.32	0.12	-11.21	0.00	0.00
ENSSSCG00000033144	YTHDF3	3313.88	-1.31	0.06	-22.76	0.00	0.00
ENSSSCG00000035493	SACS	5188.05	-1.31	0.04	-30.87	0.00	0.00
ENSSSCG00000013312	HIPK3	2835.44	-1.31	0.05	-24.05	0.00	0.00
ENSSSCG00000001009	RIPK1	1059.55	-1.31	0.07	-18.69	0.00	0.00
ENSSSCG00000014148	TMEM161B	514.16	-1.31	0.10	-13.09	0.00	0.00
ENSSSCG00000019612	RF00069	131.87	-1.31	0.18	-7.24	0.00	0.00
ENSSSCG00000004090	ZBTB2	304.74	-1.31	0.13	-10.22	0.00	0.00
ENSSSCG00000022004	SH3BP2	338.18	-1.31	0.12	-11.38	0.00	0.00

ENSSSCG00000004408	SMPD2	1324.26	-1.31	0.07	-19.46	0.00	0.00
ENSSSCG00000016159	CPS1	81.35	-1.31	0.22	-5.99	0.00	0.00
ENSSSCG00000008395	VRK2	379.61	-1.31	0.11	-12.08	0.00	0.00
ENSSSCG00000023935	GMPPA	389.94	-1.31	0.11	-12.30	0.00	0.00
ENSSSCG00000012564	MID2	91.45	-1.31	0.21	-6.18	0.00	0.00
ENSSSCG00000004789	THBS1	22307.74	-1.31	0.05	-29.01	0.00	0.00
ENSSSCG00000007465	B4GALT5	3530.87	-1.31	0.05	-28.69	0.00	0.00
ENSSSCG00000033673	SLC25A6	18100.07	-1.31	0.04	-34.18	0.00	0.00
ENSSSCG00000013857	EPS15L1	525.28	-1.31	0.09	-14.10	0.00	0.00
ENSSSCG00000034090	CRTC3	622.62	-1.31	0.09	-14.09	0.00	0.00
ENSSSCG00000013638	ILF3	5219.66	-1.31	0.04	-32.22	0.00	0.00
ENSSSCG00000001518	ITPR3	3804.00	-1.31	0.05	-27.78	0.00	0.00
ENSSSCG00000001564	SRSF3	3164.90	-1.31	0.05	-26.70	0.00	0.00
ENSSSCG00000035822	FAM120C	747.02	-1.31	0.08	-16.74	0.00	0.00
ENSSSCG00000000838	SLC41A2	201.74	-1.31	0.15	-8.95	0.00	0.00
ENSSSCG00000015720	BIN1	700.59	-1.31	0.09	-14.57	0.00	0.00
ENSSSCG00000018076		62.49	-1.31	0.26	-5.06	0.00	0.00
ENSSSCG00000007334	SRC	1155.84	-1.31	0.07	-18.26	0.00	0.00
ENSSSCG00000033527	ITFG1	881.87	-1.31	0.07	-17.64	0.00	0.00
ENSSSCG00000009186	METAP1	2506.93	-1.31	0.05	-25.26	0.00	0.00
ENSSSCG00000004120	SHPRH	450.47	-1.30	0.10	-12.63	0.00	0.00
ENSSSCG00000016708	SKAP2	647.35	-1.30	0.08	-15.64	0.00	0.00
ENSSSCG00000011933	NECTIN3	731.51	-1.30	0.08	-16.03	0.00	0.00
ENSSSCG00000011578	TAMM41	595.55	-1.30	0.10	-13.45	0.00	0.00
ENSSSCG00000012742	MTM1	361.63	-1.30	0.11	-12.00	0.00	0.00

ENSSSCG00000023870	RF00581	53.69	-1.30	0.27	-4.80	0.00	0.00
ENSSSCG00000003710	TTC39C	158.60	-1.30	0.16	-7.96	0.00	0.00
ENSSSCG00000003700	ESCO1	391.31	-1.30	0.12	-10.97	0.00	0.00
ENSSSCG00000017601	TOM1L1	782.54	-1.30	0.08	-16.49	0.00	0.00
ENSSSCG00000010046	GNAZ	648.72	-1.30	0.10	-13.31	0.00	0.00
ENSSSCG00000016504	TBXAS1	263.50	-1.30	0.13	-10.13	0.00	0.00
ENSSSCG00000006052	FZD6	2712.81	-1.30	0.05	-26.11	0.00	0.00
ENSSSCG00000008226	POLR1A	2267.20	-1.30	0.06	-23.20	0.00	0.00
ENSSSCG00000025323	SF3B3	6482.65	-1.30	0.04	-30.53	0.00	0.00
ENSSSCG00000006709	NOTCH2	2375.83	-1.30	0.06	-23.43	0.00	0.00
ENSSSCG00000009569	PSPC1	748.31	-1.30	0.08	-16.47	0.00	0.00
ENSSSCG00000021443	SGK1	3911.50	-1.29	0.06	-23.17	0.00	0.00
ENSSSCG00000034835	SLC7A6	1501.15	-1.29	0.06	-21.70	0.00	0.00
ENSSSCG00000034167	SLC5A3	1435.94	-1.29	0.07	-18.06	0.00	0.00
ENSSSCG00000002431	TDP1	272.50	-1.29	0.13	-10.11	0.00	0.00
ENSSSCG00000016734	CCM2	1129.89	-1.29	0.07	-18.77	0.00	0.00
ENSSSCG00000015951	METAP1D	95.43	-1.29	0.21	-6.22	0.00	0.00
ENSSSCG00000025830	NUP93	1207.70	-1.29	0.07	-19.71	0.00	0.00
ENSSSCG00000016406	ESYT2	1081.46	-1.29	0.07	-19.08	0.00	0.00
ENSSSCG00000011956	TRMT10C	339.18	-1.29	0.12	-10.85	0.00	0.00
ENSSSCG00000009741	PUS1	778.68	-1.29	0.08	-16.69	0.00	0.00
ENSSSCG00000010688	MCMBP	3086.51	-1.29	0.05	-27.03	0.00	0.00
ENSSSCG00000035454	B4GALT1	608.08	-1.29	0.09	-14.99	0.00	0.00
ENSSSCG00000010148	ERO1B	46.73	-1.29	0.29	-4.47	0.00	0.00
ENSSSCG00000011677	GK5	319.61	-1.29	0.12	-11.11	0.00	0.00

ENSSSCG00000028159		274.37	-1.29	0.12	-10.32	0.00	0.00
ENSSSCG00000024385	RNASEH2B	730.37	-1.29	0.08	-15.88	0.00	0.00
ENSSSCG00000036067	PDP2	1129.62	-1.29	0.07	-18.20	0.00	0.00
ENSSSCG00000029803	KDM2B	693.56	-1.28	0.08	-15.82	0.00	0.00
ENSSSCG00000024001	PLXNA1	2785.24	-1.28	0.05	-25.42	0.00	0.00
ENSSSCG00000024361	TFB1M	240.14	-1.28	0.13	-9.86	0.00	0.00
ENSSSCG00000002555	JAG2	1445.88	-1.28	0.06	-20.04	0.00	0.00
ENSSSCG00000001974	G2E3	663.37	-1.28	0.11	-11.64	0.00	0.00
ENSSSCG00000022925		334.45	-1.28	0.12	-11.09	0.00	0.00
ENSSSCG00000024592	ANKRD17	2413.86	-1.28	0.05	-23.44	0.00	0.00
ENSSSCG00000032078	ZNF362	68.16	-1.28	0.24	-5.23	0.00	0.00
ENSSSCG00000011892		24.09	-1.28	0.40	-3.20	0.00	0.00
ENSSSCG00000003730	RNF138	523.21	-1.28	0.10	-12.27	0.00	0.00
ENSSSCG00000006877	SNX7	518.74	-1.28	0.09	-13.76	0.00	0.00
ENSSSCG00000026793	B4GALT2	381.99	-1.28	0.11	-11.45	0.00	0.00
ENSSSCG00000023994	ARMC9	117.96	-1.28	0.18	-6.99	0.00	0.00
ENSSSCG00000010145	HEATR1	2591.12	-1.28	0.06	-22.73	0.00	0.00
ENSSSCG00000004339	UFL1	790.30	-1.28	0.08	-16.07	0.00	0.00
ENSSSCG00000009431	DGKH	261.33	-1.28	0.13	-9.87	0.00	0.00
ENSSSCG00000038913	HERC4	763.93	-1.27	0.08	-15.92	0.00	0.00
ENSSSCG00000012642	STAG2	2190.92	-1.27	0.06	-20.27	0.00	0.00
ENSSSCG00000022345		230.82	-1.27	0.14	-9.38	0.00	0.00
ENSSSCG00000030361	PRKCZ	370.22	-1.27	0.11	-11.13	0.00	0.00
ENSSSCG00000040123		40.92	-1.27	0.32	-4.01	0.00	0.00
ENSSSCG00000023974	PHF21A	182.79	-1.27	0.16	-7.98	0.00	0.00

ENSSSCG00000009862	RNFT2	113.83	-1.27	0.19	-6.79	0.00	0.00
ENSSSCG00000006853	PRMT6	25.57	-1.27	0.39	-3.26	0.00	0.00
ENSSSCG00000015789	SNX25	263.43	-1.27	0.13	-9.60	0.00	0.00
ENSSSCG00000007565	TTYH3	2387.40	-1.27	0.05	-23.59	0.00	0.00
ENSSSCG00000011417	DCAF1	1434.35	-1.27	0.06	-20.94	0.00	0.00
ENSSSCG00000011731	SMC4	5642.73	-1.27	0.05	-26.37	0.00	0.00
ENSSSCG00000040616	RF00412	10.52	-1.27	0.61	-2.07	0.04	0.05
ENSSSCG00000010470	IDE	2638.23	-1.27	0.05	-24.41	0.00	0.00
ENSSSCG00000005103		269.63	-1.27	0.12	-10.32	0.00	0.00
ENSSSCG00000003755	MCOLN2	125.13	-1.27	0.18	-7.13	0.00	0.00
ENSSSCG00000004561	HERC1	693.89	-1.27	0.08	-15.41	0.00	0.00
ENSSSCG00000005614	FAM129B	2322.43	-1.26	0.06	-21.95	0.00	0.00
ENSSSCG00000036431	VPS13C	2352.78	-1.26	0.06	-19.81	0.00	0.00
ENSSSCG00000009320	FLT1	1171.00	-1.26	0.07	-17.67	0.00	0.00
ENSSSCG00000004241	GJA1	280.79	-1.26	0.13	-9.92	0.00	0.00
ENSSSCG00000010241	TET1	60.88	-1.26	0.28	-4.59	0.00	0.00
ENSSSCG00000035901	SPIN1	1086.27	-1.26	0.08	-16.70	0.00	0.00
ENSSSCG00000036961	PCDHB7	90.23	-1.26	0.21	-5.98	0.00	0.00
ENSSSCG00000016378	PASK	468.51	-1.26	0.10	-13.04	0.00	0.00
ENSSSCG00000009524	TMTC4	336.58	-1.26	0.11	-11.42	0.00	0.00
ENSSSCG00000017907	PFN1	18604.73	-1.26	0.05	-25.49	0.00	0.00
ENSSSCG00000001431		45.75	-1.26	0.30	-4.20	0.00	0.00
ENSSSCG00000027130	TNFRSF12A	2593.18	-1.26	0.05	-24.40	0.00	0.00
ENSSSCG00000008578	ADCY3	778.22	-1.26	0.08	-15.56	0.00	0.00
ENSSSCG00000011181	NEK11	41.44	-1.26	0.31	-3.99	0.00	0.00

ENSSSCG00000032558	EMX2	1413.34	-1.25	0.06	-20.35	0.00	0.00
ENSSSCG00000039255	RF00287	152.06	-1.25	0.16	-7.62	0.00	0.00
ENSSSCG00000039549	PLEKHF2	895.97	-1.25	0.07	-17.18	0.00	0.00
ENSSSCG00000032820	BRD1	600.57	-1.25	0.09	-14.06	0.00	0.00
ENSSSCG00000037647	TFDP2	415.79	-1.25	0.10	-12.45	0.00	0.00
ENSSSCG00000035366	MFHAS1	363.54	-1.25	0.11	-11.29	0.00	0.00
ENSSSCG00000011620	RUVBL1	3524.75	-1.25	0.05	-26.68	0.00	0.00
ENSSSCG00000005436		3483.10	-1.25	0.05	-27.10	0.00	0.00
ENSSSCG00000035582	GIPC2	200.61	-1.25	0.16	-7.94	0.00	0.00
ENSSSCG00000029741	RALA	888.24	-1.25	0.07	-16.84	0.00	0.00
ENSSSCG00000002900		179.34	-1.25	0.16	-7.84	0.00	0.00
ENSSSCG00000016114	FAM117B	113.93	-1.25	0.19	-6.52	0.00	0.00
ENSSSCG00000016509		1564.34	-1.25	0.06	-20.27	0.00	0.00
ENSSSCG00000010817	LYPLAL1	395.28	-1.25	0.11	-11.44	0.00	0.00
ENSSSCG00000013490	PIP5K1C	298.56	-1.25	0.12	-10.25	0.00	0.00
ENSSSCG00000016367	RNPEPL1	716.51	-1.25	0.08	-15.71	0.00	0.00
ENSSSCG00000013314	QSER1	2844.16	-1.25	0.05	-22.82	0.00	0.00
ENSSSCG00000037245	HNRNPDL	4352.55	-1.25	0.04	-29.38	0.00	0.00
ENSSSCG00000023086	RF00405	23.04	-1.25	0.41	-3.04	0.00	0.00
ENSSSCG00000028771	MEF2A	1082.23	-1.25	0.07	-17.54	0.00	0.00
ENSSSCG00000004139	ADGRG6	2119.12	-1.25	0.06	-21.48	0.00	0.00
ENSSSCG00000015743	PTPN4	360.41	-1.25	0.11	-10.94	0.00	0.00
ENSSSCG00000040130		20.93	-1.24	0.43	-2.91	0.00	0.01
ENSSSCG00000011158	PFKP	2815.34	-1.24	0.05	-24.41	0.00	0.00
ENSSSCG00000016049	HIBCH	151.33	-1.24	0.17	-7.43	0.00	0.00

ENSSSCG00000007748	PSPH	412.45	-1.24	0.10	-11.98	0.00	0.00
ENSSSCG00000025156	BRWD3	436.87	-1.24	0.11	-11.64	0.00	0.00
ENSSSCG00000016008	CWC22	819.42	-1.24	0.08	-15.78	0.00	0.00
ENSSSCG00000027476	RF00581	178.45	-1.24	0.15	-8.30	0.00	0.00
ENSSSCG00000026001	DNAJC1	169.40	-1.24	0.15	-8.04	0.00	0.00
ENSSSCG00000007275	RALY	2681.77	-1.24	0.05	-23.82	0.00	0.00
ENSSSCG00000016778	NLN	1478.09	-1.24	0.06	-19.90	0.00	0.00
ENSSSCG00000011672	RASA2	570.94	-1.24	0.09	-13.36	0.00	0.00
ENSSSCG00000010143	MTR	242.83	-1.24	0.14	-9.08	0.00	0.00
ENSSSCG00000037645	COTL1	4521.78	-1.24	0.05	-26.34	0.00	0.00
ENSSSCG00000021149	RNF217	227.22	-1.24	0.14	-8.86	0.00	0.00
ENSSSCG00000029094	PIK3R4	934.04	-1.24	0.07	-16.61	0.00	0.00
ENSSSCG00000005518		1965.72	-1.24	0.07	-18.85	0.00	0.00
ENSSSCG00000006029	NUDCD1	1645.30	-1.24	0.07	-18.36	0.00	0.00
ENSSSCG00000012163	RPS6KA3	675.98	-1.24	0.09	-14.07	0.00	0.00
ENSSSCG00000024904	SEC22A	199.46	-1.23	0.14	-8.57	0.00	0.00
ENSSSCG00000010242		60.30	-1.23	0.26	-4.75	0.00	0.00
ENSSSCG00000038494	LPIN2	2983.55	-1.23	0.05	-24.73	0.00	0.00
ENSSSCG00000010744	DHX32	561.16	-1.23	0.09	-13.92	0.00	0.00
ENSSSCG00000006296	ATP1B1	5384.89	-1.23	0.05	-26.27	0.00	0.00
ENSSSCG00000021173	BSPRY	391.19	-1.23	0.11	-11.49	0.00	0.00
ENSSSCG00000025305		555.88	-1.23	0.09	-13.97	0.00	0.00
ENSSSCG00000038549	ZFP36L2	4398.91	-1.23	0.06	-21.77	0.00	0.00
ENSSSCG00000011776	YEATS2	1149.43	-1.23	0.07	-18.11	0.00	0.00
ENSSSCG00000005601	HSPA5	20472.94	-1.23	0.05	-26.98	0.00	0.00

ENSSSCG00000025194	ZSWIM6	227.21	-1.23	0.14	-8.78	0.00	0.00
ENSSSCG00000011013	WAC	1622.31	-1.23	0.06	-19.95	0.00	0.00
ENSSSCG00000016553	COPG2	625.71	-1.23	0.09	-13.75	0.00	0.00
ENSSSCG00000004958	PIAS1	549.66	-1.23	0.10	-12.72	0.00	0.00
ENSSSCG00000005355	EXOSC3	149.28	-1.23	0.17	-7.30	0.00	0.00
ENSSSCG00000027738	LRRFIP2	714.33	-1.23	0.08	-14.56	0.00	0.00
ENSSSCG00000037953	NDUFC1	114.35	-1.23	0.22	-5.50	0.00	0.00
ENSSSCG00000038632	ADNP2	616.45	-1.23	0.08	-14.54	0.00	0.00
ENSSSCG00000031249	PTTG1IP	1942.24	-1.23	0.05	-22.46	0.00	0.00
ENSSSCG00000005895	C8orf82	580.85	-1.23	0.09	-13.42	0.00	0.00
ENSSSCG00000035123	COMMD5	37.20	-1.23	0.33	-3.75	0.00	0.00
ENSSSCG00000022786	MOGS	621.00	-1.23	0.08	-14.53	0.00	0.00
ENSSSCG00000032517	DMXL2	459.42	-1.23	0.10	-12.77	0.00	0.00
ENSSSCG00000030451	LRRC14	374.41	-1.23	0.11	-11.57	0.00	0.00
ENSSSCG00000021059	ADORA1	452.04	-1.23	0.10	-12.68	0.00	0.00
ENSSSCG00000008242	KCMF1	1309.15	-1.23	0.07	-18.57	0.00	0.00
ENSSSCG00000003909	PIK3R3	110.01	-1.23	0.19	-6.53	0.00	0.00
ENSSSCG00000008887	NAF1	977.97	-1.22	0.07	-17.10	0.00	0.00
ENSSSCG00000021906	SYT7	511.46	-1.22	0.09	-13.18	0.00	0.00
ENSSSCG00000040735	DDAH1	1626.97	-1.22	0.06	-21.01	0.00	0.00
ENSSSCG00000011772		881.28	-1.22	0.08	-15.51	0.00	0.00
ENSSSCG00000026158	RAD18	670.47	-1.22	0.09	-14.12	0.00	0.00
ENSSSCG00000039360	LRFN4	1137.61	-1.22	0.08	-14.86	0.00	0.00
ENSSSCG00000024578	PHLDB1	2255.87	-1.22	0.05	-23.62	0.00	0.00
ENSSSCG00000014177	CHD1	1875.28	-1.22	0.07	-18.58	0.00	0.00

ENSSSCG00000027179	SIM2	129.07	-1.22	0.18	-6.70	0.00	0.00
ENSSSCG00000012136	PIR	34.37	-1.22	0.34	-3.58	0.00	0.00
ENSSSCG00000015984	HOXD4	23.48	-1.22	0.42	-2.93	0.00	0.01
ENSSSCG00000003401	KIF1B	2406.35	-1.22	0.05	-22.79	0.00	0.00
ENSSSCG00000033591	CHD9	974.60	-1.22	0.08	-15.64	0.00	0.00
ENSSSCG00000029756	ADGRG2	99.82	-1.22	0.20	-6.20	0.00	0.00
ENSSSCG00000013508	SH3GL1	2329.77	-1.22	0.05	-22.65	0.00	0.00
ENSSSCG00000016715	OSBPL3	327.92	-1.22	0.12	-10.45	0.00	0.00
ENSSSCG00000011482	C3orf67	148.76	-1.21	0.16	-7.40	0.00	0.00
ENSSSCG00000013745	FARSA	2041.48	-1.21	0.05	-22.44	0.00	0.00
ENSSSCG00000007331	RBL1	2642.81	-1.21	0.07	-18.19	0.00	0.00
ENSSSCG00000010863	AHCTF1	3074.12	-1.21	0.06	-21.81	0.00	0.00
ENSSSCG00000022222	MTX2	647.83	-1.21	0.09	-14.17	0.00	0.00
ENSSSCG00000015945	METTL8	308.89	-1.21	0.12	-10.15	0.00	0.00
ENSSSCG00000027410	CBFA2T2	238.69	-1.21	0.13	-9.13	0.00	0.00
ENSSSCG00000001527	ILRUN	1562.25	-1.21	0.06	-19.75	0.00	0.00
ENSSSCG00000033010	AHNAK	22235.14	-1.21	0.04	-27.43	0.00	0.00
ENSSSCG00000018053	MED9	593.82	-1.21	0.09	-13.13	0.00	0.00
ENSSSCG00000031620		18.91	-1.21	0.46	-2.62	0.01	0.01
ENSSSCG00000002838	ZNF423	13.57	-1.21	0.53	-2.29	0.02	0.03
ENSSSCG00000008404	MTIF2	814.77	-1.21	0.07	-16.27	0.00	0.00
ENSSSCG00000007488	DOK5	339.67	-1.21	0.11	-10.67	0.00	0.00
ENSSSCG00000022083		139.85	-1.21	0.17	-7.11	0.00	0.00
ENSSSCG00000013886	B3GNT3	733.18	-1.21	0.09	-13.97	0.00	0.00
ENSSSCG00000011136	TASOR2	1728.09	-1.21	0.06	-21.29	0.00	0.00

ENSSSCG00000024697	TMEM125	122.78	-1.21	0.18	-6.63	0.00	0.00
ENSSSCG00000025357	LDAH	199.79	-1.21	0.16	-7.73	0.00	0.00
ENSSSCG00000000528	PKP2	836.12	-1.21	0.07	-16.19	0.00	0.00
ENSSSCG00000008079	FAM120A	4770.84	-1.20	0.05	-25.50	0.00	0.00
ENSSSCG00000024663		194.42	-1.20	0.14	-8.34	0.00	0.00
ENSSSCG00000032544	MTA1	1558.73	-1.20	0.06	-19.65	0.00	0.00
ENSSSCG00000036451		222.21	-1.20	0.14	-8.60	0.00	0.00
ENSSSCG00000001871	HMG20A	1104.99	-1.20	0.07	-17.52	0.00	0.00
ENSSSCG00000006912	HFM1	25.99	-1.20	0.40	-3.00	0.00	0.00
ENSSSCG00000011826	TMEM44	423.06	-1.20	0.10	-11.49	0.00	0.00
ENSSSCG00000004503	LOXHD1	57.50	-1.20	0.26	-4.58	0.00	0.00
ENSSSCG00000018456	RF00277	46.84	-1.20	0.29	-4.19	0.00	0.00
ENSSSCG00000024222	MLH1	510.37	-1.20	0.09	-12.80	0.00	0.00
ENSSSCG00000015497	KLHL20	530.64	-1.20	0.10	-12.15	0.00	0.00
ENSSSCG00000006580	S100A2	8770.27	-1.20	0.12	-9.67	0.00	0.00
ENSSSCG00000011640	TF	124.45	-1.20	0.20	-6.05	0.00	0.00
ENSSSCG00000030413	PIK3CA	782.53	-1.20	0.09	-13.68	0.00	0.00
ENSSSCG00000033608	LOXL2	5340.64	-1.20	0.04	-27.67	0.00	0.00
ENSSSCG00000033944	CYB5B	4001.21	-1.20	0.05	-23.18	0.00	0.00
ENSSSCG00000013875	USE1	59.98	-1.19	0.26	-4.60	0.00	0.00
ENSSSCG00000010062	CABIN1	2118.13	-1.19	0.06	-21.18	0.00	0.00
ENSSSCG00000027628	IL6R	217.22	-1.19	0.14	-8.41	0.00	0.00
ENSSSCG00000000927	TMTC3	1139.64	-1.19	0.07	-15.94	0.00	0.00
ENSSSCG00000000908		1194.05	-1.19	0.07	-17.09	0.00	0.00
ENSSSCG00000035028	GRK6	1272.16	-1.19	0.06	-18.39	0.00	0.00

ENSSSCG00000035432	HDGFL3	2033.48	-1.19	0.08	-15.70	0.00	0.00
ENSSSCG00000008729	LYAR	2184.13	-1.19	0.06	-20.66	0.00	0.00
ENSSSCG00000024938	SH3BP5	428.56	-1.19	0.10	-12.03	0.00	0.00
ENSSSCG00000030225	TRA2B	2855.77	-1.19	0.05	-22.05	0.00	0.00
ENSSSCG00000027169	ENOSF1	27.23	-1.19	0.38	-3.11	0.00	0.00
ENSSSCG00000003592	SDC3	1175.22	-1.19	0.08	-14.89	0.00	0.00
ENSSSCG00000009302	USP12	371.35	-1.19	0.11	-11.13	0.00	0.00
ENSSSCG00000028552	BHLHE41	3672.23	-1.19	0.06	-19.17	0.00	0.00
ENSSSCG00000021885	MDFIC	851.94	-1.19	0.08	-15.66	0.00	0.00
ENSSSCG00000010825	MARK1	248.30	-1.19	0.13	-9.11	0.00	0.00
ENSSSCG00000011743	MECOM	745.29	-1.19	0.08	-15.01	0.00	0.00
ENSSSCG00000013751	NACC1	2785.80	-1.19	0.06	-21.21	0.00	0.00
ENSSSCG00000007993	CAPN15	1132.31	-1.18	0.07	-18.07	0.00	0.00
ENSSSCG00000003513	ECE1	830.40	-1.18	0.08	-15.34	0.00	0.00
ENSSSCG00000007514	VAPB	3107.11	-1.18	0.05	-23.00	0.00	0.00
ENSSSCG00000021591		4880.21	-1.18	0.05	-25.06	0.00	0.00
ENSSSCG00000032145	IRX5	246.21	-1.18	0.13	-9.05	0.00	0.00
ENSSSCG00000038135		69.36	-1.18	0.28	-4.22	0.00	0.00
ENSSSCG00000024424	FAM91A1	3515.56	-1.18	0.06	-18.85	0.00	0.00
ENSSSCG00000007944		185.15	-1.18	0.15	-8.00	0.00	0.00
ENSSSCG00000040845	AGPAT2	742.97	-1.18	0.08	-13.93	0.00	0.00
ENSSSCG00000028806	RPS6KA4	589.84	-1.18	0.09	-12.59	0.00	0.00
ENSSSCG00000001379	TUBB	25950.76	-1.18	0.04	-27.12	0.00	0.00
ENSSSCG00000004963		1490.46	-1.18	0.07	-17.38	0.00	0.00
ENSSSCG00000001137	HIST1H1D	735.84	-1.18	0.08	-14.12	0.00	0.00

ENSSSCG00000017140	CHMP6	930.64	-1.18	0.08	-15.65	0.00	0.00
ENSSSCG00000004595	ADAM10	1757.58	-1.18	0.06	-20.75	0.00	0.00
ENSSSCG00000029339	SPATA2L	57.05	-1.18	0.27	-4.37	0.00	0.00
ENSSSCG00000010654	ATRNL1	28.81	-1.17	0.37	-3.18	0.00	0.00
ENSSSCG00000008044	MLST8	311.90	-1.17	0.12	-10.15	0.00	0.00
ENSSSCG00000012793	HCFC1	4105.21	-1.17	0.06	-21.22	0.00	0.00
ENSSSCG00000004899	RELCH	1079.39	-1.17	0.07	-17.12	0.00	0.00
ENSSSCG00000028135	PRTFDC1	1470.76	-1.17	0.06	-19.09	0.00	0.00
ENSSSCG00000005469	KIAA1958	128.57	-1.17	0.17	-6.75	0.00	0.00
ENSSSCG00000000453	ATP23	323.86	-1.17	0.11	-10.47	0.00	0.00
ENSSSCG00000003976	NFYC	855.94	-1.17	0.08	-14.16	0.00	0.00
ENSSSCG00000031547		804.05	-1.17	0.08	-15.49	0.00	0.00
ENSSSCG00000014871	TSKU	1025.22	-1.17	0.07	-16.12	0.00	0.00
ENSSSCG00000022722	CARMIL2	48.42	-1.17	0.29	-4.06	0.00	0.00
ENSSSCG00000035259	PHF3	2505.98	-1.17	0.06	-21.10	0.00	0.00
ENSSSCG00000009142	SEC24B	1000.27	-1.17	0.07	-16.75	0.00	0.00
ENSSSCG00000009040	SMAD1	434.19	-1.17	0.10	-11.96	0.00	0.00
ENSSSCG00000005222	SLC1A1	3815.53	-1.17	0.05	-22.71	0.00	0.00
ENSSSCG00000022508	UIMC1	106.59	-1.17	0.19	-6.07	0.00	0.00
ENSSSCG00000040386	GINS2	664.00	-1.16	0.09	-13.33	0.00	0.00
ENSSSCG00000002528	TECPR2	187.82	-1.16	0.16	-7.42	0.00	0.00
ENSSSCG00000008398	PPP4R3B	2893.33	-1.16	0.05	-21.42	0.00	0.00
ENSSSCG00000035489	C11orf74	124.97	-1.16	0.18	-6.57	0.00	0.00
ENSSSCG00000037655		609.44	-1.16	0.09	-12.42	0.00	0.00
ENSSSCG00000003437	TNFRSF8	697.28	-1.16	0.09	-13.17	0.00	0.00

ENSSSCG00000000466	XPOT	5261.22	-1.16	0.04	-27.41	0.00	0.00
ENSSSCG00000010764		82.93	-1.16	0.22	-5.37	0.00	0.00
ENSSSCG00000013633	CARM1	2653.00	-1.16	0.05	-22.54	0.00	0.00
ENSSSCG00000040267	CYS1	279.04	-1.16	0.12	-9.33	0.00	0.00
ENSSSCG00000012572	COL4A5	30.60	-1.16	0.35	-3.31	0.00	0.00
ENSSSCG00000009879	TPCN1	549.58	-1.16	0.09	-12.67	0.00	0.00
ENSSSCG00000028886	IPO7	10077.37	-1.16	0.04	-28.89	0.00	0.00
ENSSSCG00000022080	RHOA	7950.43	-1.16	0.04	-27.93	0.00	0.00
ENSSSCG00000012854	RASSF7	331.84	-1.16	0.12	-9.78	0.00	0.00
ENSSSCG00000008722		271.94	-1.15	0.12	-9.37	0.00	0.00
ENSSSCG00000001878	PTPN9	467.98	-1.15	0.10	-12.06	0.00	0.00
ENSSSCG00000031302	CTBP1	1890.80	-1.15	0.05	-21.20	0.00	0.00
ENSSSCG00000004592	SLTM	1503.89	-1.15	0.06	-18.02	0.00	0.00
ENSSSCG00000011426	POC1A	636.10	-1.15	0.09	-13.02	0.00	0.00
ENSSSCG00000023929	TXNRD3	203.12	-1.15	0.15	-7.56	0.00	0.00
ENSSSCG00000008536	WDR43	3355.04	-1.15	0.05	-22.17	0.00	0.00
ENSSSCG00000011881	IQCB1	187.65	-1.15	0.15	-7.65	0.00	0.00
ENSSSCG00000017104	NSUN2	6671.37	-1.15	0.04	-28.26	0.00	0.00
ENSSSCG00000001544	TEAD3	441.21	-1.15	0.10	-11.74	0.00	0.00
ENSSSCG00000037719	PDPK1	1423.68	-1.15	0.06	-18.72	0.00	0.00
ENSSSCG00000005582	STRBP	1039.23	-1.15	0.07	-16.49	0.00	0.00
ENSSSCG00000006016	EIF3H	2814.67	-1.15	0.06	-20.49	0.00	0.00
ENSSSCG00000002652	KLHDC4	576.39	-1.15	0.09	-13.04	0.00	0.00
ENSSSCG00000040642	LASP1	9940.96	-1.15	0.04	-29.39	0.00	0.00
ENSSSCG00000008675	LETM1	3478.49	-1.15	0.05	-24.95	0.00	0.00

ENSSSCG00000010672	RAB11FIP2	428.43	-1.15	0.10	-11.39	0.00	0.00
ENSSSCG00000008909	CLOCK	1356.35	-1.15	0.06	-17.85	0.00	0.00
ENSSSCG00000009090	KIAA1109	611.69	-1.15	0.09	-12.63	0.00	0.00
ENSSSCG00000007366	MYBL2	8532.10	-1.15	0.05	-24.22	0.00	0.00
ENSSSCG00000026488		813.01	-1.15	0.08	-13.58	0.00	0.00
ENSSSCG00000010311	CAMK2G	781.98	-1.14	0.08	-15.08	0.00	0.00
ENSSSCG00000031417		12.02	-1.14	0.57	-2.01	0.04	0.06
ENSSSCG00000035739	SLC26A1	16.67	-1.14	0.49	-2.31	0.02	0.03
ENSSSCG00000005364	TDRD7	131.63	-1.14	0.18	-6.42	0.00	0.00
ENSSSCG00000012880	CPT1A	2727.37	-1.14	0.05	-22.91	0.00	0.00
ENSSSCG00000011710	EIF2A	2594.18	-1.14	0.05	-22.13	0.00	0.00
ENSSSCG00000012774	DUSP9	1473.40	-1.14	0.07	-16.49	0.00	0.00
ENSSSCG00000008427	KCNK12	110.58	-1.14	0.19	-5.95	0.00	0.00
ENSSSCG00000006879	PTBP2	569.70	-1.14	0.10	-11.42	0.00	0.00
ENSSSCG00000023727	TRIM37	1408.82	-1.14	0.07	-17.02	0.00	0.00
ENSSSCG00000039963	RF00334	18.90	-1.14	0.45	-2.52	0.01	0.02
ENSSSCG00000004146	REPS1	1071.66	-1.14	0.07	-16.17	0.00	0.00
ENSSSCG00000003128	LIG1	1205.97	-1.14	0.07	-16.56	0.00	0.00
ENSSSCG00000001539	PPARD	395.84	-1.14	0.12	-9.42	0.00	0.00
ENSSSCG00000015872	GPD2	2790.68	-1.14	0.05	-21.79	0.00	0.00
ENSSSCG00000001880	SIN3A	1432.00	-1.14	0.07	-16.67	0.00	0.00
ENSSSCG00000000493	FRS2	291.18	-1.14	0.12	-9.19	0.00	0.00
ENSSSCG00000013432	MIDN	1088.45	-1.14	0.07	-15.67	0.00	0.00
ENSSSCG00000022823	MCM5	2693.13	-1.14	0.06	-20.61	0.00	0.00
ENSSSCG00000010164		621.96	-1.14	0.09	-12.79	0.00	0.00

ENSSSCG00000017316		873.08	-1.14	0.07	-15.53	0.00	0.00
ENSSSCG00000008136	RANBP2	6994.72	-1.14	0.04	-27.02	0.00	0.00
ENSSSCG00000006033	EIF3E	4826.49	-1.14	0.05	-23.29	0.00	0.00
ENSSSCG00000008385	PUS10	560.94	-1.13	0.09	-12.67	0.00	0.00
ENSSSCG00000009015	GATB	312.39	-1.13	0.12	-9.58	0.00	0.00
ENSSSCG00000004968	PAQR5	568.75	-1.13	0.09	-12.77	0.00	0.00
ENSSSCG00000008591	ATAD2B	330.69	-1.13	0.11	-10.26	0.00	0.00
ENSSSCG00000021289	GAK	1677.54	-1.13	0.06	-19.10	0.00	0.00
ENSSSCG00000036075	WASHC3	263.86	-1.13	0.13	-8.71	0.00	0.00
ENSSSCG00000011420	RBM15B	605.30	-1.13	0.09	-13.14	0.00	0.00
ENSSSCG00000003965		98.72	-1.13	0.20	-5.65	0.00	0.00
ENSSSCG00000006939	ZNHIT6	941.79	-1.13	0.07	-15.87	0.00	0.00
ENSSSCG00000010435	MINPP1	921.37	-1.13	0.08	-14.21	0.00	0.00
ENSSSCG00000021536	CLDN9	62.54	-1.13	0.25	-4.59	0.00	0.00
ENSSSCG00000040617	TNFAIP8	192.51	-1.13	0.15	-7.78	0.00	0.00
ENSSSCG00000007109	XRN2	4573.47	-1.13	0.04	-26.32	0.00	0.00
ENSSSCG00000014379	PCDHB2	26.12	-1.13	0.38	-2.96	0.00	0.00
ENSSSCG00000004276	SMAP1	1185.44	-1.13	0.07	-17.29	0.00	0.00
ENSSSCG00000026710	CARHSP1	1392.09	-1.13	0.06	-18.80	0.00	0.00
ENSSSCG00000040167		102.51	-1.13	0.19	-5.80	0.00	0.00
ENSSSCG00000011330	NBEAL2	756.23	-1.13	0.08	-13.93	0.00	0.00
ENSSSCG00000036284	USP46	519.34	-1.13	0.09	-12.53	0.00	0.00
ENSSSCG00000010865	EXO1	581.39	-1.13	0.09	-12.11	0.00	0.00
ENSSSCG00000010238	HNRNPH3	2350.67	-1.13	0.06	-19.50	0.00	0.00
ENSSSCG00000009611	XPO7	2687.28	-1.13	0.05	-22.89	0.00	0.00

ENSSSCG00000003111	SAE1	1813.09	-1.13	0.06	-20.17	0.00	0.00
ENSSSCG00000001024	RIOK1	959.87	-1.13	0.07	-16.12	0.00	0.00
ENSSSCG00000023818	MRPS27	536.85	-1.13	0.09	-12.33	0.00	0.00
ENSSSCG00000019644	RF00281	117.62	-1.12	0.19	-5.88	0.00	0.00
ENSSSCG00000022212	SNRNP35	266.11	-1.12	0.13	-8.82	0.00	0.00
ENSSSCG00000001782	ABHD17C	386.84	-1.12	0.10	-10.93	0.00	0.00
ENSSSCG00000000877	SCYL2	777.02	-1.12	0.09	-12.19	0.00	0.00
ENSSSCG00000027205	LPCAT3	1438.47	-1.12	0.07	-16.61	0.00	0.00
ENSSSCG00000029602	WDR37	590.05	-1.12	0.09	-12.55	0.00	0.00
ENSSSCG00000006461	ARHGEF11	647.22	-1.12	0.10	-10.81	0.00	0.00
ENSSSCG00000015689	DARS	1949.34	-1.12	0.06	-17.44	0.00	0.00
ENSSSCG00000040337	AK4	2218.08	-1.12	0.06	-17.82	0.00	0.00
ENSSSCG00000004392		3500.87	-1.12	0.06	-20.31	0.00	0.00
ENSSSCG00000038008	RBM14	474.52	-1.12	0.10	-11.34	0.00	0.00
ENSSSCG00000020868	TMEM192	384.61	-1.12	0.11	-10.14	0.00	0.00
ENSSSCG00000021749	MCF2L2	46.60	-1.12	0.29	-3.93	0.00	0.00
ENSSSCG00000011425	RAD54L2	649.83	-1.12	0.09	-12.21	0.00	0.00
ENSSSCG00000032574	RTTN	326.79	-1.12	0.12	-9.61	0.00	0.00
ENSSSCG00000007252	DNMT3B	191.90	-1.12	0.15	-7.34	0.00	0.00
ENSSSCG00000033902	ZNF414	175.19	-1.12	0.15	-7.29	0.00	0.00
ENSSSCG00000014170	CAST	4364.74	-1.12	0.05	-24.75	0.00	0.00
ENSSSCG00000001814	IQGAP1	11662.93	-1.12	0.04	-29.33	0.00	0.00
ENSSSCG00000007840	POLR3E	1196.43	-1.12	0.06	-17.26	0.00	0.00
ENSSSCG00000006338	DDR2	827.46	-1.12	0.08	-14.70	0.00	0.00
ENSSSCG00000027503	OSBPL8	956.40	-1.12	0.08	-13.95	0.00	0.00

ENSSSCG00000012639	THOC2	2478.34	-1.12	0.06	-19.08	0.00	0.00
ENSSSCG00000012202		170.93	-1.12	0.16	-6.92	0.00	0.00
ENSSSCG00000033497	RERE	1235.10	-1.12	0.08	-14.39	0.00	0.00
ENSSSCG00000026705	NVL	944.51	-1.11	0.07	-15.62	0.00	0.00
ENSSSCG00000027371	PCDHGC4	193.01	-1.11	0.14	-7.80	0.00	0.00
ENSSSCG00000007664	AGFG2	1165.33	-1.11	0.07	-15.60	0.00	0.00
ENSSSCG00000001483	FAM83B	584.66	-1.11	0.09	-12.64	0.00	0.00
ENSSSCG00000016036	WDR75	1464.38	-1.11	0.06	-18.29	0.00	0.00
ENSSSCG00000011645	CEP63	189.52	-1.11	0.15	-7.21	0.00	0.00
ENSSSCG00000006888	SLC44A3	241.86	-1.11	0.13	-8.55	0.00	0.00
ENSSSCG00000008175	CHST10	1219.75	-1.11	0.06	-17.55	0.00	0.00
ENSSSCG00000012551	RNF128	282.99	-1.11	0.12	-9.13	0.00	0.00
ENSSSCG00000005689	FNBP1	381.69	-1.11	0.11	-10.45	0.00	0.00
ENSSSCG00000009944	CORO1C	2281.51	-1.11	0.05	-21.43	0.00	0.00
ENSSSCG00000029113	RNF219	617.19	-1.11	0.09	-12.38	0.00	0.00
ENSSSCG00000014903	CCDC90B	1557.65	-1.11	0.06	-19.07	0.00	0.00
ENSSSCG00000015129	ARHGEF12	2550.02	-1.11	0.05	-20.92	0.00	0.00
ENSSSCG00000016266	CAB39	1692.21	-1.11	0.06	-17.48	0.00	0.00
ENSSSCG00000031954	SLC39A10	951.40	-1.11	0.08	-14.41	0.00	0.00
ENSSSCG00000017448	KRT14	8683.55	-1.11	0.04	-24.92	0.00	0.00
ENSSSCG00000038542	NCBP2AS2	112.56	-1.11	0.20	-5.48	0.00	0.00
ENSSSCG00000012591	AMOT	1458.68	-1.11	0.06	-18.43	0.00	0.00
ENSSSCG00000038287	RDH13	395.63	-1.11	0.10	-10.73	0.00	0.00
ENSSSCG00000027997	NME5	32.42	-1.11	0.35	-3.20	0.00	0.00
ENSSSCG00000032334		23.28	-1.11	0.41	-2.67	0.01	0.01

ENSSSCG00000006306	MPZL1	2125.53	-1.11	0.06	-18.74	0.00	0.00
ENSSSCG00000040373	BCL2L13	704.00	-1.11	0.08	-13.96	0.00	0.00
ENSSSCG00000000118	MICALL1	1141.43	-1.11	0.07	-16.68	0.00	0.00
ENSSSCG00000030820	STK4	1434.19	-1.10	0.06	-18.70	0.00	0.00
ENSSSCG00000024692	RRBP1	4557.07	-1.10	0.05	-22.48	0.00	0.00
ENSSSCG00000005959	FAM49B	989.83	-1.10	0.07	-15.51	0.00	0.00
ENSSSCG00000035622	UBE2D3	6002.74	-1.10	0.04	-26.02	0.00	0.00
ENSSSCG00000015270	FMOD	188.72	-1.10	0.16	-6.89	0.00	0.00
ENSSSCG00000036785		281.97	-1.10	0.13	-8.54	0.00	0.00
ENSSSCG00000005724	SETX	1526.81	-1.10	0.06	-17.06	0.00	0.00
ENSSSCG00000015947	DCAF17	246.48	-1.10	0.13	-8.31	0.00	0.00
ENSSSCG00000012123	RAB9A	855.57	-1.10	0.08	-13.67	0.00	0.00
ENSSSCG00000015583	SMYD2	597.46	-1.10	0.09	-12.88	0.00	0.00
ENSSSCG00000013397	ARNTL	84.76	-1.10	0.21	-5.19	0.00	0.00
ENSSSCG00000032275	MRPS30	729.55	-1.10	0.09	-12.48	0.00	0.00
ENSSSCG00000009629	BIN3	215.36	-1.10	0.14	-8.04	0.00	0.00
ENSSSCG00000033653		1926.76	-1.10	0.06	-17.71	0.00	0.00
ENSSSCG00000038509	NOTCH1	602.78	-1.10	0.08	-12.95	0.00	0.00
ENSSSCG00000013305	CAPRIN1	8383.52	-1.10	0.04	-25.73	0.00	0.00
ENSSSCG00000011028	EPC1	295.95	-1.10	0.12	-8.96	0.00	0.00
ENSSSCG00000019783	RF00150	21.08	-1.10	0.42	-2.59	0.01	0.01
ENSSSCG00000026663	DTD2	372.60	-1.10	0.11	-10.27	0.00	0.00
ENSSSCG00000031706		122.86	-1.10	0.18	-5.99	0.00	0.00
ENSSSCG00000035883	GPR160	389.82	-1.10	0.11	-10.04	0.00	0.00
ENSSSCG00000023747	TTC19	733.40	-1.10	0.08	-13.01	0.00	0.00

ENSSSCG00000029082	KMT2E	5433.41	-1.10	0.05	-23.00	0.00	0.00
ENSSSCG00000011112	CDC123	798.91	-1.10	0.08	-14.03	0.00	0.00
ENSSSCG00000025672		547.84	-1.09	0.09	-12.09	0.00	0.00
ENSSSCG00000016554	MEST	1578.70	-1.09	0.06	-17.04	0.00	0.00
ENSSSCG00000032702		2963.29	-1.09	0.05	-21.22	0.00	0.00
ENSSSCG00000018229	RF00284	92.09	-1.09	0.20	-5.34	0.00	0.00
ENSSSCG00000010176	C1orf131	692.51	-1.09	0.08	-13.38	0.00	0.00
ENSSSCG00000023334	BCLAF3	85.89	-1.09	0.22	-5.05	0.00	0.00
ENSSSCG00000028019	LRRC59	7926.53	-1.09	0.04	-27.01	0.00	0.00
ENSSSCG00000015714	CCDC93	482.04	-1.09	0.09	-11.55	0.00	0.00
ENSSSCG00000029248		288.82	-1.09	0.12	-8.77	0.00	0.00
ENSSSCG00000015603	LPGAT1	4312.23	-1.09	0.05	-21.22	0.00	0.00
ENSSSCG00000006757	TRIM33	1100.69	-1.09	0.07	-14.75	0.00	0.00
ENSSSCG00000012251	USP9X	2012.52	-1.09	0.06	-18.85	0.00	0.00
ENSSSCG00000036145	PRKAA2	675.74	-1.09	0.08	-13.22	0.00	0.00
ENSSSCG00000000202	MCRS1	767.35	-1.09	0.08	-13.58	0.00	0.00
ENSSSCG00000037982	RF00278	65.38	-1.09	0.24	-4.54	0.00	0.00
ENSSSCG00000002506	VRK1	622.50	-1.09	0.09	-12.39	0.00	0.00
ENSSSCG00000005247		3503.97	-1.09	0.05	-23.70	0.00	0.00
ENSSSCG00000005948	TG	21.36	-1.09	0.44	-2.47	0.01	0.02
ENSSSCG00000014184	PAM	1023.40	-1.09	0.07	-15.61	0.00	0.00
ENSSSCG00000024665	TRAPPC12	643.84	-1.09	0.09	-12.74	0.00	0.00
ENSSSCG00000015710		5955.94	-1.09	0.04	-24.62	0.00	0.00
ENSSSCG00000004132	PHACTR2	75.37	-1.09	0.23	-4.78	0.00	0.00
ENSSSCG00000012267	JADE3	619.63	-1.08	0.09	-12.44	0.00	0.00

ENSSSCG00000012540	SLC25A53	199.41	-1.08	0.15	-7.40	0.00	0.00
ENSSSCG00000011516	EIF4E3	319.05	-1.08	0.11	-9.56	0.00	0.00
ENSSSCG00000012131	MOSPD2	791.47	-1.08	0.08	-13.89	0.00	0.00
ENSSSCG00000002540	PPP2R5C	1229.55	-1.08	0.06	-16.70	0.00	0.00
ENSSSCG00000010573	HPS6	296.90	-1.08	0.12	-8.84	0.00	0.00
ENSSSCG00000012770	CCNQ	281.25	-1.08	0.12	-8.85	0.00	0.00
ENSSSCG00000025775	TADA2A	1294.24	-1.08	0.06	-17.04	0.00	0.00
ENSSSCG00000025108	G6PD	3775.27	-1.08	0.06	-18.76	0.00	0.00
ENSSSCG00000035585		379.76	-1.08	0.11	-9.90	0.00	0.00
ENSSSCG00000031123	PPIG	1741.89	-1.08	0.07	-16.45	0.00	0.00
ENSSSCG00000001488	GCLC	1031.16	-1.08	0.07	-14.81	0.00	0.00
ENSSSCG00000005022	NIN	1089.65	-1.08	0.07	-14.55	0.00	0.00
ENSSSCG00000008498	HEATR5B	883.50	-1.08	0.07	-14.84	0.00	0.00
ENSSSCG00000008009	LMF1	393.02	-1.08	0.11	-9.76	0.00	0.00
ENSSSCG00000038410	CPEB2	739.06	-1.08	0.09	-11.74	0.00	0.00
ENSSSCG00000039821	GPRIN3	44.10	-1.08	0.30	-3.55	0.00	0.00
ENSSSCG00000028737	USP3	693.48	-1.08	0.08	-13.48	0.00	0.00
ENSSSCG00000002414	SEL1L	3379.62	-1.07	0.05	-22.14	0.00	0.00
ENSSSCG00000034373	TNRC6C	186.71	-1.07	0.15	-7.03	0.00	0.00
ENSSSCG00000015949	SLC25A12	693.71	-1.07	0.09	-12.37	0.00	0.00
ENSSSCG00000000975	PANX2	348.92	-1.07	0.12	-9.23	0.00	0.00
ENSSSCG00000038125	NUP210L	42.15	-1.07	0.30	-3.54	0.00	0.00
ENSSSCG00000009270	IFT88	595.01	-1.07	0.09	-12.49	0.00	0.00
ENSSSCG00000032723	ACER3	1218.01	-1.07	0.07	-15.67	0.00	0.00
ENSSSCG00000000599	DERA	710.39	-1.07	0.08	-13.71	0.00	0.00

ENSSSCG00000010163	KCNK1	1401.26	-1.07	0.06	-16.95	0.00	0.00
ENSSSCG00000006873	FRRS1	8129.75	-1.07	0.04	-26.52	0.00	0.00
ENSSSCG00000012386	FAM155B	1413.25	-1.07	0.06	-17.63	0.00	0.00
ENSSSCG00000011229	STT3B	6686.68	-1.07	0.05	-22.38	0.00	0.00
ENSSSCG00000005678	NTMT1	520.46	-1.07	0.09	-11.77	0.00	0.00
ENSSSCG00000027509	VPS54	617.06	-1.07	0.09	-11.73	0.00	0.00
ENSSSCG00000015235	ETS1	2761.33	-1.07	0.05	-20.36	0.00	0.00
ENSSSCG00000017230	TMEM104	403.55	-1.07	0.10	-10.41	0.00	0.00
ENSSSCG00000032367	CEBPD	1004.06	-1.07	0.08	-13.04	0.00	0.00
ENSSSCG00000016677	GARS	5688.47	-1.07	0.04	-25.79	0.00	0.00
ENSSSCG00000030255	EIF4G1	20973.46	-1.07	0.04	-28.48	0.00	0.00
ENSSSCG00000000887		135.95	-1.07	0.40	-2.65	0.01	0.01
ENSSSCG00000029485	FBXO11	1106.64	-1.07	0.08	-13.50	0.00	0.00
ENSSSCG00000009787	CCDC62	443.35	-1.07	0.10	-10.28	0.00	0.00
ENSSSCG00000000807	SLC38A1	1272.33	-1.07	0.06	-16.87	0.00	0.00
ENSSSCG00000010203		7803.68	-1.07	0.04	-28.03	0.00	0.00
ENSSSCG00000030415	DPP3	1470.61	-1.07	0.06	-17.58	0.00	0.00
ENSSSCG00000018942	RF00591	95.42	-1.07	0.21	-5.01	0.00	0.00
ENSSSCG00000010508	TM9SF3	2546.73	-1.07	0.07	-15.77	0.00	0.00
ENSSSCG00000030291	RPRD2	1355.13	-1.07	0.06	-17.04	0.00	0.00
ENSSSCG00000007019	GPAT4	3527.29	-1.06	0.05	-23.17	0.00	0.00
ENSSSCG00000011363	PRKAR2A	2600.23	-1.06	0.05	-21.44	0.00	0.00
ENSSSCG00000005706	ABL1	1582.44	-1.06	0.06	-17.57	0.00	0.00
ENSSSCG00000008415	ASB3	255.63	-1.06	0.13	-8.20	0.00	0.00
ENSSSCG00000005477	FKBP15	594.94	-1.06	0.09	-12.48	0.00	0.00

ENSSSCG00000013402	USP47	1639.34	-1.06	0.07	-15.76	0.00	0.00
ENSSSCG00000021206	IL1RAP	516.27	-1.06	0.10	-10.80	0.00	0.00
ENSSSCG00000006146	LRRCC1	989.37	-1.06	0.08	-13.55	0.00	0.00
ENSSSCG00000023362	RHBDF2	1188.85	-1.06	0.07	-16.21	0.00	0.00
ENSSSCG00000039419	SLCO4A1	748.75	-1.06	0.11	-9.77	0.00	0.00
ENSSSCG00000007516	STX16	1284.10	-1.06	0.07	-16.21	0.00	0.00
ENSSSCG00000029388	PDE2A	351.43	-1.06	0.11	-9.37	0.00	0.00
ENSSSCG00000031710	BRF1	637.22	-1.06	0.09	-12.20	0.00	0.00
ENSSSCG00000034064	RF00056	128.87	-1.06	0.18	-5.98	0.00	0.00
ENSSSCG00000016488	SSBP1	888.78	-1.06	0.07	-14.35	0.00	0.00
ENSSSCG00000037559	ACOT11	754.16	-1.06	0.09	-11.76	0.00	0.00
ENSSSCG00000029624	TADA2B	1329.51	-1.06	0.06	-17.08	0.00	0.00
ENSSSCG00000009083	SPRY1	514.18	-1.06	0.10	-10.85	0.00	0.00
ENSSSCG00000030535	SNX9	1226.64	-1.06	0.07	-15.48	0.00	0.00
ENSSSCG00000012377	KIF4A	2271.75	-1.05	0.05	-19.88	0.00	0.00
ENSSSCG00000037355	RNF115	1819.30	-1.05	0.06	-18.71	0.00	0.00
ENSSSCG00000012656	ELF4	822.99	-1.05	0.08	-13.81	0.00	0.00
ENSSSCG00000011235	UBP1	2996.99	-1.05	0.05	-22.20	0.00	0.00
ENSSSCG00000010536	CNNM1	501.53	-1.05	0.09	-11.26	0.00	0.00
ENSSSCG00000014240	CSNK1G3	320.31	-1.05	0.12	-8.92	0.00	0.00
ENSSSCG00000037061	RRP1	730.75	-1.05	0.08	-13.02	0.00	0.00
ENSSSCG00000011639	SRPRB	986.76	-1.05	0.07	-15.09	0.00	0.00
ENSSSCG00000016686	PRR15	148.06	-1.05	0.16	-6.41	0.00	0.00
ENSSSCG00000014902	ANKRD42	390.32	-1.05	0.11	-9.85	0.00	0.00
ENSSSCG00000004222	NCOA7	119.83	-1.05	0.19	-5.67	0.00	0.00

ENSSSCG00000039348	H1F0	6919.67	-1.05	0.05	-22.53	0.00	0.00
ENSSSCG00000006872	AGL	882.58	-1.05	0.08	-13.17	0.00	0.00
ENSSSCG00000040974		577.50	-1.05	0.09	-11.32	0.00	0.00
ENSSSCG00000025455	RALB	1661.56	-1.05	0.06	-18.04	0.00	0.00
ENSSSCG00000038801	NPNT	7381.59	-1.05	0.04	-25.00	0.00	0.00
ENSSSCG00000008026	CRAMP1	488.01	-1.05	0.10	-10.73	0.00	0.00
ENSSSCG00000036113		445.94	-1.05	0.10	-10.31	0.00	0.00
ENSSSCG00000000278		1155.50	-1.05	0.07	-15.43	0.00	0.00
ENSSSCG00000012375	DLG3	458.33	-1.05	0.10	-10.90	0.00	0.00
ENSSSCG00000022982	VPS50	902.91	-1.05	0.08	-13.60	0.00	0.00
ENSSSCG00000016911	MTREX	2437.29	-1.05	0.05	-19.35	0.00	0.00
ENSSSCG00000002868	PEPD	794.19	-1.05	0.09	-12.27	0.00	0.00
ENSSSCG00000003559	ARID1A	2897.53	-1.05	0.05	-21.18	0.00	0.00
ENSSSCG00000006101		377.63	-1.05	0.11	-9.90	0.00	0.00
ENSSSCG00000003831	OMA1	404.42	-1.05	0.10	-10.41	0.00	0.00
ENSSSCG00000015857	UGGT1	7235.40	-1.05	0.04	-23.36	0.00	0.00
ENSSSCG00000017108		232.95	-1.05	0.13	-7.77	0.00	0.00
ENSSSCG00000027349	TBC1D14	345.08	-1.05	0.11	-9.45	0.00	0.00
ENSSSCG00000023569		165.58	-1.04	0.16	-6.48	0.00	0.00
ENSSSCG00000006553	UBAP2L	7828.15	-1.04	0.04	-24.85	0.00	0.00
ENSSSCG00000015231	DCPS	781.61	-1.04	0.08	-13.15	0.00	0.00
ENSSSCG00000004708	TUBGCP4	346.28	-1.04	0.11	-9.43	0.00	0.00
ENSSSCG00000033530	RF02124	15.11	-1.04	0.51	-2.03	0.04	0.06
ENSSSCG00000005056	DLGAP5	1787.13	-1.04	0.07	-15.23	0.00	0.00
ENSSSCG00000007767	ZNF668	309.25	-1.04	0.12	-8.79	0.00	0.00

ENSSSCG00000002276	PLEKHG3	2960.07	-1.04	0.05	-20.63	0.00	0.00
ENSSSCG00000005207	ERMP1	1020.26	-1.04	0.07	-14.85	0.00	0.00
ENSSSCG00000032894		104.02	-1.04	0.20	-5.32	0.00	0.00
ENSSSCG00000014989	DCUN1D5	1087.23	-1.04	0.07	-15.51	0.00	0.00
ENSSSCG00000026034	NUP153	1861.44	-1.04	0.05	-19.03	0.00	0.00
ENSSSCG00000008728	ZBTB49	156.80	-1.04	0.17	-6.24	0.00	0.00
ENSSSCG00000015232	ST3GAL4	928.06	-1.04	0.07	-14.35	0.00	0.00
ENSSSCG00000004387	FOXO3	566.06	-1.04	0.10	-9.88	0.00	0.00
ENSSSCG00000023186	CA4	22.39	-1.04	0.43	-2.43	0.01	0.02
ENSSSCG00000004486	SLC17A5	621.44	-1.04	0.09	-11.83	0.00	0.00
ENSSSCG00000023118	BZW2	1729.46	-1.04	0.06	-17.98	0.00	0.00
ENSSSCG00000021273		20.86	-1.04	0.42	-2.44	0.01	0.02
ENSSSCG00000025343	ZNF628	282.14	-1.04	0.12	-8.57	0.00	0.00
ENSSSCG00000000199		744.30	-1.04	0.08	-13.37	0.00	0.00
ENSSSCG00000000274	PCBP2	7387.47	-1.04	0.04	-26.07	0.00	0.00
ENSSSCG00000034614		134.84	-1.03	0.18	-5.81	0.00	0.00
ENSSSCG00000037234	CLDN2	177.49	-1.03	0.15	-6.83	0.00	0.00
ENSSSCG00000014416	TCERG1	2054.49	-1.03	0.05	-18.88	0.00	0.00
ENSSSCG00000013072	FADS2	2737.39	-1.03	0.05	-20.63	0.00	0.00
ENSSSCG00000001641	UBR2	844.89	-1.03	0.08	-13.59	0.00	0.00
ENSSSCG00000033299	FLNA	62681.71	-1.03	0.03	-31.21	0.00	0.00
ENSSSCG00000008573	RAB10	4619.30	-1.03	0.04	-23.75	0.00	0.00
ENSSSCG00000010568	NPM3	3019.79	-1.03	0.07	-15.64	0.00	0.00
ENSSSCG00000004404		248.22	-1.03	0.14	-7.13	0.00	0.00
ENSSSCG00000029944	FASN	11805.32	-1.03	0.04	-23.39	0.00	0.00

ENSSSCG00000036157	BARX2	175.42	-1.03	0.15	-6.90	0.00	0.00
ENSSSCG00000031532	ABO	563.73	-1.03	0.09	-11.48	0.00	0.00
ENSSSCG00000034730	PIAS2	898.76	-1.03	0.08	-13.59	0.00	0.00
ENSSSCG00000003189	PRMT1	5295.47	-1.03	0.04	-23.22	0.00	0.00
ENSSSCG00000001660	PTK7	692.74	-1.03	0.08	-12.20	0.00	0.00
ENSSSCG00000031105	C9orf85	118.65	-1.03	0.19	-5.46	0.00	0.00
ENSSSCG00000033937		280.45	-1.03	0.13	-7.91	0.00	0.00
ENSSSCG00000036257		683.44	-1.03	0.08	-12.49	0.00	0.00
ENSSSCG00000005630	NAIF1	19.88	-1.03	0.44	-2.33	0.02	0.03
ENSSSCG00000005201		798.39	-1.03	0.08	-13.17	0.00	0.00
ENSSSCG00000016205	NHEJ1	410.17	-1.03	0.10	-9.93	0.00	0.00
ENSSSCG00000008748	LCORL	286.89	-1.03	0.12	-8.23	0.00	0.00
ENSSSCG00000030197		5312.03	-1.03	0.05	-18.81	0.00	0.00
ENSSSCG00000000843	TXNRD1	1908.56	-1.03	0.06	-17.08	0.00	0.00
ENSSSCG00000029697	FAM120B	790.21	-1.03	0.08	-13.34	0.00	0.00
ENSSSCG00000026590	HGH1	1105.49	-1.03	0.07	-14.55	0.00	0.00
ENSSSCG00000009943	SSH1	572.82	-1.02	0.09	-11.26	0.00	0.00
ENSSSCG00000009794	MLXIP	905.87	-1.02	0.08	-12.66	0.00	0.00
ENSSSCG00000040713	TXLNG	694.96	-1.02	0.08	-12.52	0.00	0.00
ENSSSCG00000021436	DFFB	115.76	-1.02	0.18	-5.57	0.00	0.00
ENSSSCG00000014146	RASA1	284.03	-1.02	0.12	-8.48	0.00	0.00
ENSSSCG00000026719		899.56	-1.02	0.08	-13.52	0.00	0.00
ENSSSCG00000040904	CLDN1	10596.37	-1.02	0.04	-23.69	0.00	0.00
ENSSSCG00000033892	EVA1B	191.67	-1.02	0.14	-7.13	0.00	0.00
ENSSSCG00000004307		1380.56	-1.02	0.06	-16.40	0.00	0.00

ENSSSCG00000030289	PTPN11	6505.62	-1.02	0.04	-24.17	0.00	0.00
ENSSSCG00000000034	TTLL12	8928.16	-1.02	0.05	-19.03	0.00	0.00
ENSSSCG00000004657	CEP152	389.28	-1.02	0.11	-9.30	0.00	0.00
ENSSSCG00000011650	MSL2	442.97	-1.02	0.10	-10.27	0.00	0.00
ENSSSCG00000031719	SEMA4B	1227.26	-1.02	0.07	-13.79	0.00	0.00
ENSSSCG00000027165	UBE2G2	753.08	-1.02	0.08	-12.64	0.00	0.00
ENSSSCG00000006562	GATAD2B	1078.85	-1.02	0.07	-14.85	0.00	0.00
ENSSSCG00000036501	WDR1	7938.09	-1.02	0.04	-26.29	0.00	0.00
ENSSSCG00000005208	RIC1	529.32	-1.02	0.10	-9.72	0.00	0.00
ENSSSCG00000022131	KCTD6	219.86	-1.02	0.14	-7.54	0.00	0.00
ENSSSCG00000015524	FAM20B	3046.45	-1.02	0.05	-21.86	0.00	0.00
ENSSSCG00000011901	TMEM39A	332.22	-1.02	0.11	-9.19	0.00	0.00
ENSSSCG00000015065	BUD13	548.43	-1.02	0.09	-11.36	0.00	0.00
ENSSSCG00000028475	KPNA1	1157.77	-1.02	0.07	-14.72	0.00	0.00
ENSSSCG00000036274		6263.93	-1.02	0.06	-17.43	0.00	0.00
ENSSSCG00000006717	PHGDH	1492.61	-1.02	0.07	-13.59	0.00	0.00
ENSSSCG00000004303	RARS2	445.00	-1.02	0.10	-9.97	0.00	0.00
ENSSSCG00000000068	EP300	3034.15	-1.02	0.05	-20.29	0.00	0.00
ENSSSCG00000002516	WARS	3052.87	-1.02	0.05	-19.32	0.00	0.00
ENSSSCG00000015480	PRRC2C	15400.03	-1.02	0.04	-25.38	0.00	0.00
ENSSSCG00000004845	FAN1	553.11	-1.02	0.09	-11.10	0.00	0.00
ENSSSCG00000031769	CTTN	5239.93	-1.01	0.04	-22.86	0.00	0.00
ENSSSCG00000003738	MAPRE2	680.45	-1.01	0.08	-12.22	0.00	0.00
ENSSSCG00000011543	LHFPL4	193.78	-1.01	0.14	-7.05	0.00	0.00
ENSSSCG00000037598	SNX10	412.31	-1.01	0.10	-10.14	0.00	0.00

ENSSSCG00000008164	MAP4K4	3321.53	-1.01	0.05	-20.02	0.00	0.00
ENSSSCG00000011580	TSEN2	535.75	-1.01	0.09	-11.41	0.00	0.00
ENSSSCG00000016556	CEP41	539.84	-1.01	0.09	-10.81	0.00	0.00
ENSSSCG00000009458		17.18	-1.01	0.46	-2.18	0.03	0.04
ENSSSCG00000028228	XPO1	3811.27	-1.01	0.06	-18.15	0.00	0.00
ENSSSCG00000025770	ST6GAL1	97.93	-1.01	0.20	-5.14	0.00	0.00
ENSSSCG00000027827	GALE	1844.60	-1.01	0.06	-17.02	0.00	0.00
ENSSSCG00000017205	SAP30BP	928.74	-1.01	0.08	-13.39	0.00	0.00
ENSSSCG00000009746	RAN	9814.87	-1.01	0.04	-24.01	0.00	0.00
ENSSSCG00000006970	DLC1	997.66	-1.01	0.07	-13.50	0.00	0.00
ENSSSCG00000000110	PLA2G6	83.60	-1.01	0.22	-4.65	0.00	0.00
ENSSSCG00000028465	ELAC2	2816.81	-1.00	0.05	-18.34	0.00	0.00
ENSSSCG00000011036	STAM	682.32	-1.00	0.09	-11.58	0.00	0.00
ENSSSCG00000028202	RANGAP1	2019.51	-1.00	0.05	-19.01	0.00	0.00
ENSSSCG00000006985		30.92	-1.00	0.36	-2.82	0.00	0.01
ENSSSCG00000006969	TRMT9B	170.92	-1.00	0.17	-6.05	0.00	0.00
ENSSSCG00000013297	CD44	3011.72	-1.00	0.06	-18.12	0.00	0.00
ENSSSCG00000004224	HDDC2	352.98	-1.00	0.11	-9.21	0.00	0.00
ENSSSCG00000025447	MID1IP1	523.63	-1.00	0.09	-10.67	0.00	0.00
ENSSSCG00000027926	FTCD	22.01	-1.00	0.41	-2.42	0.02	0.02
ENSSSCG00000037100	OPA3	279.55	-1.00	0.12	-8.29	0.00	0.00
ENSSSCG00000013848	RAB8A	1317.64	-1.00	0.07	-14.73	0.00	0.00
ENSSSCG00000010107	MED15	968.14	-1.00	0.08	-12.14	0.00	0.00
ENSSSCG00000004275	FAM135A	901.08	-1.00	0.08	-12.86	0.00	0.00
ENSSSCG00000038144		495.41	-1.00	0.09	-10.83	0.00	0.00

ENSSSCG00000035161	TMEM237	747.15	1.00	0.08	12.79	0.00	0.00
ENSSSCG00000006533	ADAM15	1348.76	1.00	0.06	15.70	0.00	0.00
ENSSSCG00000038618	RFLNB	214.17	1.00	0.14	7.29	0.00	0.00
ENSSSCG00000032475	CEP44	259.29	1.00	0.13	7.93	0.00	0.00
ENSSSCG00000002367	ISCA2	174.20	1.00	0.15	6.47	0.00	0.00
ENSSSCG00000035364		976.97	1.00	0.08	12.51	0.00	0.00
ENSSSCG00000013645	ATG4D	521.31	1.00	0.09	11.14	0.00	0.00
ENSSSCG00000040682	MAPK3	905.79	1.00	0.09	11.73	0.00	0.00
ENSSSCG00000013721		1437.94	1.00	0.06	16.52	0.00	0.00
ENSSSCG00000003037	ZNF574	787.21	1.00	0.09	11.55	0.00	0.00
ENSSSCG00000008389	PAPOLG	650.77	1.00	0.08	12.00	0.00	0.00
ENSSSCG00000035199	PTGES3L	194.58	1.00	0.15	6.86	0.00	0.00
ENSSSCG00000035728		1014.15	1.01	0.07	14.54	0.00	0.00
ENSSSCG00000009011	FHDC1	443.16	1.01	0.10	10.15	0.00	0.00
ENSSSCG00000011562	EMC3	2226.57	1.01	0.06	17.18	0.00	0.00
ENSSSCG00000027778		1933.97	1.01	0.06	17.99	0.00	0.00
ENSSSCG00000013736	PRDX2	5931.24	1.01	0.04	25.42	0.00	0.00
ENSSSCG00000000095	GTPBP1	1131.17	1.01	0.08	13.13	0.00	0.00
ENSSSCG00000028293	RCOR3	629.69	1.01	0.08	11.99	0.00	0.00
ENSSSCG00000031970	RASSF5	82.52	1.01	0.22	4.57	0.00	0.00
ENSSSCG00000026931	SERTAD1	624.21	1.01	0.09	11.32	0.00	0.00
ENSSSCG00000015014	ZC3H12C	967.46	1.01	0.08	12.97	0.00	0.00
ENSSSCG00000015816	LETM2	84.66	1.01	0.22	4.51	0.00	0.00
ENSSSCG00000013905	FKBP8	3461.83	1.01	0.05	20.70	0.00	0.00
ENSSSCG00000009774	C12orf65	336.84	1.01	0.11	8.93	0.00	0.00

ENSSSCG00000017367	MPP2	232.11	1.01	0.13	7.60	0.00	0.00
ENSSSCG00000001513	SYNGAP1	235.81	1.01	0.14	7.44	0.00	0.00
ENSSSCG00000031349	ZNF580	270.49	1.01	0.12	8.38	0.00	0.00
ENSSSCG00000009778	RILPL2	227.01	1.01	0.15	6.69	0.00	0.00
ENSSSCG00000006742	MAB21L3	245.66	1.02	0.13	7.55	0.00	0.00
ENSSSCG00000007167	VPS16	2147.15	1.02	0.05	19.63	0.00	0.00
ENSSSCG00000027072	ATP5IF1	1176.33	1.02	0.07	15.33	0.00	0.00
ENSSSCG00000007849	CRYM	824.13	1.02	0.07	13.60	0.00	0.00
ENSSSCG00000013242	ACP2	340.77	1.02	0.11	8.96	0.00	0.00
ENSSSCG00000003652	AKIRIN1	956.93	1.02	0.07	13.93	0.00	0.00
ENSSSCG00000003119	ZNF541	16.89	1.02	0.47	2.15	0.03	0.04
ENSSSCG00000016095	CLK1	833.85	1.02	0.08	13.12	0.00	0.00
ENSSSCG00000026890	GALNT6	298.79	1.02	0.12	8.65	0.00	0.00
ENSSSCG00000024019	GTF2H5	261.47	1.02	0.12	8.23	0.00	0.00
ENSSSCG00000003350	MIB2	458.75	1.02	0.10	9.95	0.00	0.00
ENSSSCG00000025106		208.87	1.02	0.15	6.79	0.00	0.00
ENSSSCG00000040125		194.59	1.02	0.15	6.99	0.00	0.00
ENSSSCG00000014905	TMEM126B	665.48	1.02	0.08	12.07	0.00	0.00
ENSSSCG00000003051	CD177	15.39	1.02	0.50	2.03	0.04	0.06
ENSSSCG00000003106	AP2S1	1378.25	1.02	0.07	15.54	0.00	0.00
ENSSSCG00000021891	ZDHHC24	447.61	1.02	0.10	9.99	0.00	0.00
ENSSSCG00000009074	ABHD18	201.47	1.02	0.15	6.88	0.00	0.00
ENSSSCG00000031893		1165.10	1.02	0.07	14.35	0.00	0.00
ENSSSCG00000010003	CCDC157	159.42	1.02	0.16	6.35	0.00	0.00
ENSSSCG00000013720		931.25	1.02	0.07	14.24	0.00	0.00

ENSSSCG00000003038	GRIK5	66.19	1.02	0.24	4.23	0.00	0.00
ENSSSCG00000017338	PLCD3	1041.64	1.02	0.07	14.68	0.00	0.00
ENSSSCG00000002446	ATXN3	1193.83	1.02	0.07	14.47	0.00	0.00
ENSSSCG00000012625	PGRMC1	5282.22	1.03	0.04	24.92	0.00	0.00
ENSSSCG00000015315	RBM48	233.00	1.03	0.13	7.80	0.00	0.00
ENSSSCG00000003491	AKR7A2	1046.23	1.03	0.07	14.05	0.00	0.00
ENSSSCG00000007089		2965.01	1.03	0.05	20.72	0.00	0.00
ENSSSCG00000024476	CES3	298.30	1.03	0.12	8.70	0.00	0.00
ENSSSCG00000010537	GOT1	1950.30	1.03	0.06	17.85	0.00	0.00
ENSSSCG00000023377	EMP3	656.71	1.03	0.08	12.30	0.00	0.00
ENSSSCG00000025284	COQ9	1225.97	1.03	0.06	15.96	0.00	0.00
ENSSSCG00000040769		96.51	1.03	0.21	4.93	0.00	0.00
ENSSSCG00000022395	SLC25A35	93.07	1.03	0.21	4.87	0.00	0.00
ENSSSCG00000037661	INAFM2	27.73	1.03	0.39	2.66	0.01	0.01
ENSSSCG00000003439	DHRS3	347.41	1.03	0.12	8.82	0.00	0.00
ENSSSCG00000016919	SETD9	167.07	1.03	0.15	6.68	0.00	0.00
ENSSSCG00000037964	GNG10	2648.41	1.03	0.06	18.46	0.00	0.00
ENSSSCG00000030362		46.28	1.03	0.30	3.41	0.00	0.00
ENSSSCG00000007753	C16orf58	370.08	1.03	0.11	9.19	0.00	0.00
ENSSSCG00000022429	KAZALD1	36.52	1.03	0.32	3.21	0.00	0.00
ENSSSCG00000009405	SUCLA2	1558.62	1.03	0.06	16.71	0.00	0.00
ENSSSCG00000003753		760.13	1.03	0.08	12.46	0.00	0.00
ENSSSCG00000002380	ZC2HC1C	80.21	1.03	0.22	4.64	0.00	0.00
ENSSSCG00000040421	HMG20B	1180.43	1.03	0.07	14.46	0.00	0.00
ENSSSCG00000038919	RP9	245.21	1.03	0.13	7.89	0.00	0.00

ENSSSCG00000038662	ZBED8	166.43	1.03	0.16	6.34	0.00	0.00
ENSSSCG00000009807	RHOF	678.77	1.03	0.08	12.41	0.00	0.00
ENSSSCG00000027147	CENPH	612.12	1.03	0.09	12.06	0.00	0.00
ENSSSCG00000003553	MTFR1L	903.48	1.03	0.07	14.53	0.00	0.00
ENSSSCG00000008569	HADHB	2858.60	1.03	0.05	20.66	0.00	0.00
ENSSSCG00000017407	GHDC	280.44	1.04	0.12	8.65	0.00	0.00
ENSSSCG00000001821	UNC45A	925.58	1.04	0.07	14.59	0.00	0.00
ENSSSCG00000013444	BTBD2	1288.94	1.04	0.06	16.66	0.00	0.00
ENSSSCG00000017761	UNC119	843.36	1.04	0.07	14.00	0.00	0.00
ENSSSCG00000006544	UBE2Q1	2564.99	1.04	0.05	19.66	0.00	0.00
ENSSSCG00000024254	ALKBH7	58.80	1.04	0.26	3.99	0.00	0.00
ENSSSCG00000002341	PAPLN	125.56	1.04	0.18	5.64	0.00	0.00
ENSSSCG00000037536	SLC25A28	630.61	1.04	0.09	11.88	0.00	0.00
ENSSSCG00000008987	CCNI	2336.12	1.04	0.06	16.49	0.00	0.00
ENSSSCG00000030677	GART	2816.79	1.04	0.05	19.63	0.00	0.00
ENSSSCG00000023312	PIN4	344.93	1.04	0.11	9.18	0.00	0.00
ENSSSCG00000035523	FUT11	2494.76	1.04	0.05	20.85	0.00	0.00
ENSSSCG00000035390	C2orf49	968.42	1.04	0.07	14.40	0.00	0.00
ENSSSCG00000034625	PXMP4	365.84	1.04	0.11	9.61	0.00	0.00
ENSSSCG00000008309		1883.12	1.04	0.06	17.91	0.00	0.00
ENSSSCG00000010853	EPHX1	780.92	1.04	0.08	12.87	0.00	0.00
ENSSSCG00000033442	HCN2	435.59	1.04	0.10	10.25	0.00	0.00
ENSSSCG00000030857	LENG8	2932.84	1.04	0.05	20.95	0.00	0.00
ENSSSCG00000004547		187.32	1.04	0.15	6.99	0.00	0.00
ENSSSCG00000027374		1093.21	1.04	0.07	15.03	0.00	0.00

ENSSSCG00000027331	COL6A3	30.58	1.04	0.37	2.83	0.00	0.01
ENSSSCG00000003214	KCNC3	67.35	1.05	0.24	4.33	0.00	0.00
ENSSSCG00000008579	CENPO	963.64	1.05	0.07	14.77	0.00	0.00
ENSSSCG00000014054	GPRIN1	139.30	1.05	0.17	6.11	0.00	0.00
ENSSSCG00000014901	PCF11	2253.63	1.05	0.06	18.42	0.00	0.00
ENSSSCG00000034752	SUPT4H1	1859.87	1.05	0.06	17.91	0.00	0.00
ENSSSCG00000010831	DUSP10	156.61	1.05	0.16	6.38	0.00	0.00
ENSSSCG00000038895	DCTN6	739.10	1.05	0.09	11.52	0.00	0.00
ENSSSCG00000029600		42.51	1.05	0.31	3.39	0.00	0.00
ENSSSCG00000006079	RIDA	1941.03	1.05	0.06	18.28	0.00	0.00
ENSSSCG00000016662	TBX20	53.48	1.05	0.27	3.85	0.00	0.00
ENSSSCG00000002793		80.92	1.05	0.22	4.80	0.00	0.00
ENSSSCG00000040714	RSPH6A	29.59	1.05	0.36	2.95	0.00	0.00
ENSSSCG00000036673	PPT1	2998.54	1.05	0.05	21.41	0.00	0.00
ENSSSCG00000020725	ERBB3	1276.27	1.05	0.07	15.02	0.00	0.00
ENSSSCG00000016050	INPP1	298.19	1.05	0.12	8.85	0.00	0.00
ENSSSCG00000028587	PRSS54	63.39	1.05	0.25	4.15	0.00	0.00
ENSSSCG00000029652	TMBIM6	6754.74	1.05	0.05	22.48	0.00	0.00
ENSSSCG00000039265	COX6A1	3597.22	1.05	0.05	20.66	0.00	0.00
ENSSSCG00000022341	TMEM207	23.37	1.05	0.41	2.55	0.01	0.02
ENSSSCG00000040586		398.32	1.05	0.10	10.04	0.00	0.00
ENSSSCG00000036747	WAS	16.12	1.05	0.49	2.13	0.03	0.05
ENSSSCG00000010584	MFSD13A	95.12	1.05	0.21	5.14	0.00	0.00
ENSSSCG00000017912	PLD2	2076.54	1.05	0.06	19.07	0.00	0.00
ENSSSCG00000038644		20.28	1.05	0.43	2.43	0.02	0.02

ENSSSCG00000011375	USP19	1420.60	1.06	0.06	17.15	0.00	0.00
ENSSSCG00000036178	CCDC167	710.03	1.06	0.08	13.43	0.00	0.00
ENSSSCG00000036568	MVB12A	780.74	1.06	0.08	13.04	0.00	0.00
ENSSSCG00000006470	RRNAD1	355.01	1.06	0.11	9.80	0.00	0.00
ENSSSCG00000005918	DGAT1	424.77	1.06	0.11	9.59	0.00	0.00
ENSSSCG00000033833		979.48	1.06	0.07	15.42	0.00	0.00
ENSSSCG00000034487	NDUFB11	1479.13	1.06	0.06	17.65	0.00	0.00
ENSSSCG00000010102	SLC7A4	35.98	1.06	0.32	3.29	0.00	0.00
ENSSSCG00000026818	CTC1	1250.23	1.06	0.07	15.48	0.00	0.00
ENSSSCG00000026547	SLC45A3	724.44	1.06	0.09	12.22	0.00	0.00
ENSSSCG00000005058	ATG14	281.92	1.06	0.12	8.86	0.00	0.00
ENSSSCG00000000634	STYK1	15.65	1.06	0.49	2.17	0.03	0.04
ENSSSCG00000034272	DENND6B	652.76	1.06	0.09	12.21	0.00	0.00
ENSSSCG00000038189	SMCR8	2945.48	1.06	0.05	20.31	0.00	0.00
ENSSSCG00000038888	C1orf122	219.40	1.06	0.13	7.89	0.00	0.00
ENSSSCG00000037499	NEDD4	391.04	1.06	0.11	9.95	0.00	0.00
ENSSSCG00000005037	ERO1A	2638.96	1.07	0.14	7.54	0.00	0.00
ENSSSCG00000034049		364.52	1.07	0.11	9.50	0.00	0.00
ENSSSCG00000036893	PTHLH	64.81	1.07	0.25	4.33	0.00	0.00
ENSSSCG00000007492	FAM210B	517.56	1.07	0.09	11.41	0.00	0.00
ENSSSCG00000006748	TSPAN2	245.28	1.07	0.13	8.18	0.00	0.00
ENSSSCG00000033390		810.56	1.07	0.08	12.59	0.00	0.00
ENSSSCG00000003451		4402.89	1.07	0.05	21.20	0.00	0.00
ENSSSCG00000011810	BCL6	1000.53	1.07	0.07	15.39	0.00	0.00
ENSSSCG00000027051	EMC7	1353.62	1.07	0.06	17.28	0.00	0.00

ENSSSCG00000010737	ABRAXAS2	728.17	1.07	0.08	12.77	0.00	0.00
ENSSSCG00000009434	RGCC	227.86	1.07	0.14	7.84	0.00	0.00
ENSSSCG00000016259	FBXO36	28.77	1.07	0.37	2.87	0.00	0.01
ENSSSCG00000035741	TOR1AIP2	4521.87	1.07	0.04	25.49	0.00	0.00
ENSSSCG00000011192	HACL1	205.43	1.07	0.14	7.70	0.00	0.00
ENSSSCG00000013861	SLC35E1	1467.76	1.07	0.06	17.53	0.00	0.00
ENSSSCG00000012290	WDR45	1012.41	1.07	0.08	13.43	0.00	0.00
ENSSSCG00000036402	MYL9	3498.86	1.07	0.05	22.64	0.00	0.00
ENSSSCG00000038229	EML2	676.23	1.07	0.09	12.30	0.00	0.00
ENSSSCG00000031493	CYBA	352.34	1.07	0.11	9.58	0.00	0.00
ENSSSCG00000039767	TH	104.86	1.07	0.20	5.44	0.00	0.00
ENSSSCG00000029626	NECAB3	210.88	1.07	0.14	7.59	0.00	0.00
ENSSSCG00000016261	SP110	352.60	1.08	0.11	9.46	0.00	0.00
ENSSSCG00000008298	DUSP11	1136.08	1.08	0.07	16.37	0.00	0.00
ENSSSCG00000006957	RHPN1	233.18	1.08	0.13	8.12	0.00	0.00
ENSSSCG00000038467		70.32	1.08	0.25	4.39	0.00	0.00
ENSSSCG00000007962	SNRNP25	546.69	1.08	0.09	11.59	0.00	0.00
ENSSSCG00000028015	GAL3ST3	67.83	1.08	0.24	4.54	0.00	0.00
ENSSSCG00000011010	C9orf72	189.89	1.08	0.15	7.28	0.00	0.00
ENSSSCG00000015823		1915.27	1.08	0.05	19.89	0.00	0.00
ENSSSCG00000006858	OLFM3	69.39	1.08	0.25	4.40	0.00	0.00
ENSSSCG00000030421	GDI1	4740.31	1.08	0.05	23.31	0.00	0.00
ENSSSCG00000020823	BSCL2	1155.79	1.08	0.08	14.33	0.00	0.00
ENSSSCG00000017962	KDM6B	1531.76	1.08	0.07	16.12	0.00	0.00
ENSSSCG00000001511	PHF1	186.42	1.08	0.16	6.95	0.00	0.00

ENSSSCG00000001457	SLA-DQB1	13.16	1.08	0.54	1.99	0.05	0.06
ENSSSCG00000017265	AXIN2	180.71	1.08	0.15	7.18	0.00	0.00
ENSSSCG00000033627	YIPF3	902.82	1.08	0.07	15.18	0.00	0.00
ENSSSCG00000003741		225.84	1.09	0.13	8.11	0.00	0.00
ENSSSCG00000039307	H2AFJ	177.97	1.09	0.15	7.18	0.00	0.00
ENSSSCG00000008961	MTHFD2L	65.99	1.09	0.24	4.48	0.00	0.00
ENSSSCG00000001092	TDP2	641.93	1.09	0.09	12.67	0.00	0.00
ENSSSCG00000009671	PBK	1160.57	1.09	0.07	15.42	0.00	0.00
ENSSSCG00000035331		79.25	1.09	0.23	4.70	0.00	0.00
ENSSSCG00000034566	NPDC1	649.00	1.09	0.08	13.01	0.00	0.00
ENSSSCG00000006940	CCN1	1181.83	1.09	0.07	15.98	0.00	0.00
ENSSSCG00000008388	REL	744.31	1.09	0.08	13.62	0.00	0.00
ENSSSCG00000030849	RPL39	4206.30	1.09	0.06	18.72	0.00	0.00
ENSSSCG00000025134	FAM171B	17.97	1.09	0.48	2.30	0.02	0.03
ENSSSCG00000030108	ZNFX1	3676.63	1.09	0.05	23.84	0.00	0.00
ENSSSCG00000034632	PDXP	622.52	1.09	0.09	12.53	0.00	0.00
ENSSSCG00000002697		115.40	1.09	0.19	5.84	0.00	0.00
ENSSSCG00000040087	SPRTN	842.65	1.09	0.08	13.74	0.00	0.00
ENSSSCG00000000997	PPP1R3G	137.22	1.09	0.18	6.05	0.00	0.00
ENSSSCG00000032328	MAP6D1	307.66	1.09	0.12	9.32	0.00	0.00
ENSSSCG00000003481	PADI1	885.19	1.09	0.08	13.49	0.00	0.00
ENSSSCG00000012890	TCIRG1	500.63	1.09	0.09	11.92	0.00	0.00
ENSSSCG00000037347		110.27	1.09	0.19	5.79	0.00	0.00
ENSSSCG00000033278	ATP6V1D	2411.43	1.09	0.06	19.64	0.00	0.00
ENSSSCG00000008117	ZNF2	185.05	1.09	0.15	7.45	0.00	0.00

ENSSSCG00000007965	ZNF200	554.53	1.10	0.09	11.92	0.00	0.00
ENSSSCG00000002378	MLH3	432.76	1.10	0.10	10.96	0.00	0.00
ENSSSCG00000031871		2790.16	1.10	0.05	21.04	0.00	0.00
ENSSSCG00000012792	RENBP	640.02	1.10	0.08	13.10	0.00	0.00
ENSSSCG00000007470	RIPOR3	198.25	1.10	0.15	7.23	0.00	0.00
ENSSSCG00000016577	ATP6V1F	942.27	1.10	0.07	14.95	0.00	0.00
ENSSSCG00000005659	ZER1	209.81	1.10	0.14	8.05	0.00	0.00
ENSSSCG00000036665	ZNF394	184.99	1.10	0.16	7.04	0.00	0.00
ENSSSCG00000013622	TMEM205	674.16	1.10	0.08	13.50	0.00	0.00
ENSSSCG00000027057	SYVN1	260.58	1.10	0.13	8.56	0.00	0.00
ENSSSCG00000032129	COX7B	3941.27	1.10	0.06	19.61	0.00	0.00
ENSSSCG00000008051	ABCA3	235.36	1.10	0.14	7.98	0.00	0.00
ENSSSCG00000008130	CIAO1	3229.99	1.10	0.05	21.17	0.00	0.00
ENSSSCG00000017164	TIMP2	678.65	1.10	0.09	12.99	0.00	0.00
ENSSSCG00000013601	MARCH2	306.52	1.10	0.12	9.27	0.00	0.00
ENSSSCG00000003379	KLHL21	1117.63	1.10	0.07	16.83	0.00	0.00
ENSSSCG00000017337	ACBD4	186.97	1.11	0.16	6.90	0.00	0.00
ENSSSCG00000035253	MAFG	626.81	1.11	0.09	12.56	0.00	0.00
ENSSSCG00000007970	POLR3K	673.35	1.11	0.08	13.17	0.00	0.00
ENSSSCG00000040286	CYLD	464.39	1.11	0.10	11.24	0.00	0.00
ENSSSCG00000034954	C5orf24	1200.13	1.11	0.07	15.89	0.00	0.00
ENSSSCG00000014048	HK3	36.27	1.11	0.33	3.33	0.00	0.00
ENSSSCG00000002460	UBR7	1971.82	1.11	0.06	19.23	0.00	0.00
ENSSSCG00000010026	PIK3IP1	80.58	1.11	0.22	5.10	0.00	0.00
ENSSSCG00000006237	SDCBP	5363.09	1.11	0.04	27.16	0.00	0.00

ENSSSCG00000034916	GORASP1	774.66	1.11	0.08	14.45	0.00	0.00
ENSSSCG00000002383	FOS	966.80	1.11	0.08	14.55	0.00	0.00
ENSSSCG00000027100	MED31	540.92	1.11	0.09	12.25	0.00	0.00
ENSSSCG00000037850		55.59	1.11	0.26	4.28	0.00	0.00
ENSSSCG00000027970	PRRT2	60.98	1.11	0.26	4.31	0.00	0.00
ENSSSCG00000017605	MMD	547.38	1.11	0.09	12.63	0.00	0.00
ENSSSCG00000001042	MAK	413.60	1.12	0.10	11.06	0.00	0.00
ENSSSCG00000033054	ROM1	55.13	1.12	0.26	4.24	0.00	0.00
ENSSSCG00000007529	SYCP2	307.05	1.12	0.13	8.31	0.00	0.00
ENSSSCG00000009379	NEK3	165.01	1.12	0.16	7.13	0.00	0.00
ENSSSCG00000029179	HIGD2A	1158.39	1.12	0.07	16.15	0.00	0.00
ENSSSCG00000010084		69.12	1.12	0.24	4.67	0.00	0.00
ENSSSCG00000016755	POLM	294.99	1.12	0.12	9.35	0.00	0.00
ENSSSCG00000004053	TAGAP	54.21	1.12	0.27	4.10	0.00	0.00
ENSSSCG00000007160	DDRGK1	1150.23	1.12	0.06	17.30	0.00	0.00
ENSSSCG00000037015	SESN3	572.23	1.12	0.10	11.62	0.00	0.00
ENSSSCG00000032123	SPX	144.98	1.12	0.17	6.80	0.00	0.00
ENSSSCG00000040186	C12orf57	64.92	1.12	0.25	4.42	0.00	0.00
ENSSSCG00000010798	GGA2	356.09	1.13	0.11	10.10	0.00	0.00
ENSSSCG00000015111	C2CD2L	341.78	1.13	0.12	9.78	0.00	0.00
ENSSSCG00000029752	C16orf54	197.94	1.13	0.15	7.27	0.00	0.00
ENSSSCG00000032967	CACNB3	883.92	1.13	0.07	15.62	0.00	0.00
ENSSSCG00000031958	KCTD2	1675.84	1.13	0.06	17.63	0.00	0.00
ENSSSCG00000005021	SAV1	1329.35	1.13	0.06	17.37	0.00	0.00
ENSSSCG00000026919	RF01294	14.52	1.13	0.52	2.17	0.03	0.04

ENSSSCG00000008092	NT5DC4	15.57	1.13	0.53	2.14	0.03	0.04
ENSSSCG00000027701	RP2	311.41	1.13	0.13	8.46	0.00	0.00
ENSSSCG00000004672	GATM	69.45	1.13	0.24	4.78	0.00	0.00
ENSSSCG00000002041	SLC7A7	150.80	1.13	0.17	6.82	0.00	0.00
ENSSSCG00000001984	KHNYN	863.58	1.13	0.08	14.63	0.00	0.00
ENSSSCG00000028065	USPL1	810.71	1.13	0.08	14.36	0.00	0.00
ENSSSCG00000020912	HECTD2	54.55	1.13	0.29	3.91	0.00	0.00
ENSSSCG00000031738		36.15	1.14	0.35	3.24	0.00	0.00
ENSSSCG00000032626		734.80	1.14	0.08	13.51	0.00	0.00
ENSSSCG00000021255	ADA	84.93	1.14	0.21	5.30	0.00	0.00
ENSSSCG00000010849	WDR26	1867.37	1.14	0.06	20.09	0.00	0.00
ENSSSCG00000007743		3961.96	1.14	0.04	26.09	0.00	0.00
ENSSSCG00000025190	COQ10A	180.72	1.14	0.15	7.70	0.00	0.00
ENSSSCG00000013012	SNX15	147.80	1.14	0.16	7.01	0.00	0.00
ENSSSCG00000040296	EHHADH	4572.28	1.14	0.05	23.52	0.00	0.00
ENSSSCG00000006841	CLCC1	1138.30	1.14	0.07	17.14	0.00	0.00
ENSSSCG00000007797	ITGAL	145.12	1.14	0.16	6.92	0.00	0.00
ENSSSCG00000016969		28.23	1.14	0.38	3.01	0.00	0.00
ENSSSCG00000007770	ORAI3	1045.41	1.14	0.08	14.68	0.00	0.00
ENSSSCG00000030284	TRNAU1AP	697.70	1.14	0.08	13.77	0.00	0.00
ENSSSCG00000032165	ATP5MD	1215.19	1.14	0.07	15.76	0.00	0.00
ENSSSCG00000005309	FAM166B	13.05	1.15	0.55	2.08	0.04	0.05
ENSSSCG00000006371	USF1	900.79	1.15	0.08	15.21	0.00	0.00
ENSSSCG00000010719		274.16	1.15	0.12	9.28	0.00	0.00
ENSSSCG00000035675		1707.31	1.15	0.06	17.82	0.00	0.00

ENSSSCG00000012940	B4GAT1	628.94	1.15	0.10	11.81	0.00	0.00
ENSSSCG00000033732	ANKRD10	812.71	1.15	0.08	14.73	0.00	0.00
ENSSSCG00000026990	FAM162A	364.59	1.15	0.12	9.52	0.00	0.00
ENSSSCG00000010682	PRDX3	8217.68	1.15	0.04	30.39	0.00	0.00
ENSSSCG00000007648	GAL3ST4	86.53	1.15	0.22	5.12	0.00	0.00
ENSSSCG00000021258		12.49	1.15	0.56	2.05	0.04	0.05
ENSSSCG00000030182	DEDD2	114.95	1.15	0.19	6.17	0.00	0.00
ENSSSCG00000011437	ALAS1	2582.08	1.15	0.05	21.24	0.00	0.00
ENSSSCG00000013507	MPND	129.74	1.15	0.18	6.54	0.00	0.00
ENSSSCG00000002035	C14orf119	412.90	1.15	0.10	11.13	0.00	0.00
ENSSSCG00000017226		1886.80	1.15	0.05	21.03	0.00	0.00
ENSSSCG00000037238	DBF4B	468.71	1.15	0.10	11.60	0.00	0.00
ENSSSCG00000039860		124.82	1.15	0.19	6.05	0.00	0.00
ENSSSCG00000014959	PIWIL4	104.72	1.16	0.20	5.87	0.00	0.00
ENSSSCG00000009395	SETDB2	467.66	1.16	0.10	11.71	0.00	0.00
ENSSSCG00000001720	SLC25A27	417.96	1.16	0.10	11.36	0.00	0.00
ENSSSCG00000001573	PIM1	652.72	1.16	0.09	13.47	0.00	0.00
ENSSSCG00000024070	CDK5	205.14	1.16	0.14	8.08	0.00	0.00
ENSSSCG00000033883		32.77	1.16	0.37	3.15	0.00	0.00
ENSSSCG00000037254	RNPC3	747.65	1.16	0.08	14.34	0.00	0.00
ENSSSCG00000023829	CCP110	1432.48	1.16	0.06	18.62	0.00	0.00
ENSSSCG00000000298	PPP1R1A	100.04	1.16	0.20	5.84	0.00	0.00
ENSSSCG00000004114	RAB32	599.60	1.16	0.09	13.66	0.00	0.00
ENSSSCG00000006361	PPOX	736.53	1.16	0.09	13.56	0.00	0.00
ENSSSCG00000001826	CFAP100	182.84	1.16	0.15	7.68	0.00	0.00

ENSSSCG00000004314	PM20D2	1266.91	1.16	0.07	16.83	0.00	0.00
ENSSSCG00000004480	COX7A2	1085.45	1.16	0.08	14.99	0.00	0.00
ENSSSCG00000037971		19.23	1.16	0.47	2.50	0.01	0.02
ENSSSCG00000010607	COL17A1	5559.89	1.16	0.04	28.14	0.00	0.00
ENSSSCG00000011444	NT5DC2	1288.91	1.16	0.07	17.17	0.00	0.00
ENSSSCG00000016991	DUSP1	1042.07	1.16	0.08	14.36	0.00	0.00
ENSSSCG00000026939	LMAN2L	319.59	1.16	0.12	9.89	0.00	0.00
ENSSSCG00000033392	SCML4	217.14	1.17	0.14	8.16	0.00	0.00
ENSSSCG00000034378	IFNGR2	1095.87	1.17	0.07	16.64	0.00	0.00
ENSSSCG00000004875	CYB5A	1443.32	1.17	0.07	17.88	0.00	0.00
ENSSSCG00000031680	GGPS1	1798.00	1.17	0.06	20.51	0.00	0.00
ENSSSCG00000036081	TBC1D20	2648.42	1.17	0.05	22.78	0.00	0.00
ENSSSCG00000034963	IQCH	52.16	1.17	0.27	4.27	0.00	0.00
ENSSSCG00000035147	NAGLU	511.85	1.17	0.10	11.48	0.00	0.00
ENSSSCG00000008571	HADHA	4226.27	1.17	0.05	25.82	0.00	0.00
ENSSSCG00000016057	STAT1	1939.60	1.17	0.05	21.61	0.00	0.00
ENSSSCG00000014899	PRCP	1239.27	1.17	0.07	17.81	0.00	0.00
ENSSSCG00000014832	DNAJB13	172.55	1.17	0.16	7.52	0.00	0.00
ENSSSCG00000040961	LIF	955.01	1.17	0.08	15.57	0.00	0.00
ENSSSCG00000011364	NCKIPSD	451.33	1.17	0.10	12.00	0.00	0.00
ENSSSCG00000025698	SERPINE1	3637.78	1.17	0.12	9.88	0.00	0.00
ENSSSCG00000007236	TTLL9	125.95	1.17	0.18	6.61	0.00	0.00
ENSSSCG00000015241	TMEM45B	1038.85	1.17	0.07	16.62	0.00	0.00
ENSSSCG00000008833	SGCB	1163.61	1.17	0.06	18.07	0.00	0.00
ENSSSCG00000010972	DCTN3	568.03	1.17	0.09	13.09	0.00	0.00

ENSSSCG00000009164	CISD2	1027.79	1.17	0.07	15.99	0.00	0.00
ENSSSCG00000015604	NEK2	3485.25	1.17	0.05	23.00	0.00	0.00
ENSSSCG00000007958	ZNF174	681.99	1.18	0.08	14.60	0.00	0.00
ENSSSCG00000037228	RRAGB	1060.01	1.18	0.07	16.79	0.00	0.00
ENSSSCG00000037002	SERTAD2	1158.19	1.18	0.07	17.10	0.00	0.00
ENSSSCG00000037158	MTURN	118.06	1.18	0.18	6.46	0.00	0.00
ENSSSCG00000026819	NID1	60.62	1.18	0.28	4.15	0.00	0.00
ENSSSCG00000007687	LRWD1	557.02	1.18	0.09	13.43	0.00	0.00
ENSSSCG00000030309	NDRG2	445.00	1.18	0.10	11.85	0.00	0.00
ENSSSCG00000036135	COL1A1	164.11	1.18	0.16	7.37	0.00	0.00
ENSSSCG00000011570	IRAK2	390.48	1.18	0.10	11.29	0.00	0.00
ENSSSCG00000003832	TACSTD2	791.04	1.18	0.08	14.25	0.00	0.00
ENSSSCG00000026290	BBS12	139.89	1.18	0.17	6.84	0.00	0.00
ENSSSCG00000030278	MLLT11	317.25	1.18	0.12	9.86	0.00	0.00
ENSSSCG00000012014		1194.39	1.18	0.07	17.61	0.00	0.00
ENSSSCG00000015073	TAGLN	341.39	1.18	0.12	9.88	0.00	0.00
ENSSSCG00000024313		2576.71	1.18	0.05	22.43	0.00	0.00
ENSSSCG00000037617	ZBTB39	1017.59	1.19	0.07	16.11	0.00	0.00
ENSSSCG00000010235	SIRT1	705.37	1.19	0.08	14.56	0.00	0.00
ENSSSCG00000021307	USP44	274.02	1.19	0.13	9.14	0.00	0.00
ENSSSCG00000034213	ACER2	59.22	1.19	0.27	4.34	0.00	0.00
ENSSSCG00000025287	GIN1	103.58	1.19	0.20	6.05	0.00	0.00
ENSSSCG00000020953		80.34	1.19	0.22	5.37	0.00	0.00
ENSSSCG00000025691	BCL10	1507.14	1.19	0.06	19.04	0.00	0.00
ENSSSCG00000023054	IDO2	17.26	1.19	0.54	2.22	0.03	0.04

ENSSSCG00000016920	MIER3	622.80	1.19	0.09	12.67	0.00	0.00
ENSSSCG00000006493	PMF1	45.59	1.20	0.32	3.76	0.00	0.00
ENSSSCG00000000189	LMBR1L	162.32	1.20	0.17	7.24	0.00	0.00
ENSSSCG00000039545		243.08	1.20	0.13	9.00	0.00	0.00
ENSSSCG00000024773	EID2	98.49	1.20	0.20	5.84	0.00	0.00
ENSSSCG00000005902	FOXH1	10.58	1.20	0.60	2.00	0.05	0.06
ENSSSCG00000002707	GABARAPL2	1095.69	1.20	0.08	15.89	0.00	0.00
ENSSSCG00000003928	PLK3	441.93	1.20	0.10	11.92	0.00	0.00
ENSSSCG00000031442	CLP1	638.77	1.20	0.09	13.18	0.00	0.00
ENSSSCG00000023277	NDUFA1	470.82	1.20	0.10	12.01	0.00	0.00
ENSSSCG00000003572	SYTL1	45.68	1.20	0.30	4.05	0.00	0.00
ENSSSCG00000034220		43.47	1.20	0.31	3.90	0.00	0.00
ENSSSCG00000032541	TIGD2	625.59	1.20	0.09	13.18	0.00	0.00
ENSSSCG00000016886		107.77	1.20	0.19	6.18	0.00	0.00
ENSSSCG00000026794	SIRT2	753.36	1.21	0.08	14.78	0.00	0.00
ENSSSCG00000012621	UBE2A	2912.13	1.21	0.05	22.95	0.00	0.00
ENSSSCG00000038805		48.68	1.21	0.28	4.26	0.00	0.00
ENSSSCG00000012409	CITED1	72.84	1.21	0.24	4.99	0.00	0.00
ENSSSCG00000020927	SLC2A2	2038.41	1.21	0.07	16.99	0.00	0.00
ENSSSCG00000001683	POLH	1585.45	1.21	0.06	20.78	0.00	0.00
ENSSSCG00000007899		56.04	1.21	0.27	4.54	0.00	0.00
ENSSSCG00000007556	PSMG3	465.59	1.21	0.10	11.86	0.00	0.00
ENSSSCG00000001875	SNX33	282.31	1.21	0.12	9.89	0.00	0.00
ENSSSCG00000027806	SAMHD1	3341.69	1.21	0.05	22.39	0.00	0.00
ENSSSCG00000014016	SQSTM1	6363.72	1.22	0.05	26.16	0.00	0.00

ENSSSCG00000003651	RHBDL2	85.99	1.22	0.21	5.69	0.00	0.00
ENSSSCG00000033462		25.85	1.22	0.39	3.12	0.00	0.00
ENSSSCG00000003473	CPLANE2	80.34	1.22	0.22	5.46	0.00	0.00
ENSSSCG00000007985	TMEM8A	28.69	1.22	0.37	3.30	0.00	0.00
ENSSSCG00000028723	CCDC102A	116.52	1.22	0.19	6.46	0.00	0.00
ENSSSCG00000012816	MTCP1	250.90	1.22	0.13	9.23	0.00	0.00
ENSSSCG00000005083	DHRS7	747.06	1.22	0.08	14.37	0.00	0.00
ENSSSCG00000033766		70.39	1.22	0.24	5.10	0.00	0.00
ENSSSCG00000026175	FAM81B	18.02	1.22	0.47	2.60	0.01	0.01
ENSSSCG00000040550	CASC1	42.24	1.22	0.31	3.89	0.00	0.00
ENSSSCG00000010995	CHMP5	1265.65	1.22	0.06	19.10	0.00	0.00
ENSSSCG00000001927		2269.62	1.22	0.05	23.70	0.00	0.00
ENSSSCG00000022364	CPT1B	207.39	1.22	0.14	8.65	0.00	0.00
ENSSSCG00000014214	TRIM36	255.06	1.22	0.13	9.07	0.00	0.00
ENSSSCG00000012124	TRAPPC2	203.39	1.22	0.15	8.40	0.00	0.00
ENSSSCG00000003951	C1orf210	347.48	1.22	0.11	10.78	0.00	0.00
ENSSSCG00000013514	LRG1	41.76	1.23	0.30	4.04	0.00	0.00
ENSSSCG00000033787		178.87	1.23	0.15	8.10	0.00	0.00
ENSSSCG00000016705	HOXA3	125.90	1.23	0.18	6.91	0.00	0.00
ENSSSCG00000022901	KLHDC3	617.91	1.23	0.09	14.15	0.00	0.00
ENSSSCG00000031255		13.53	1.23	0.54	2.27	0.02	0.03
ENSSSCG00000040332	LBH	602.55	1.23	0.09	13.10	0.00	0.00
ENSSSCG00000023912	ORAI2	369.27	1.23	0.11	11.05	0.00	0.00
ENSSSCG00000006342	UHMK1	5259.16	1.23	0.13	9.75	0.00	0.00
ENSSSCG00000002812	KIFC3	474.69	1.23	0.10	12.46	0.00	0.00

ENSSSCG00000024267	TMEM50A	1023.34	1.23	0.07	16.46	0.00	0.00
ENSSSCG00000039802	FBXL2	119.24	1.23	0.18	6.77	0.00	0.00
ENSSSCG00000037912	FITM2	1560.35	1.23	0.06	19.70	0.00	0.00
ENSSSCG00000013046	COX8A	3110.91	1.23	0.05	22.64	0.00	0.00
ENSSSCG00000000259	CSAD	88.79	1.23	0.22	5.72	0.00	0.00
ENSSSCG00000029991	SNIP1	665.56	1.23	0.09	13.44	0.00	0.00
ENSSSCG00000027550	PLCD1	341.50	1.23	0.11	10.92	0.00	0.00
ENSSSCG00000035262	TMEM74B	43.66	1.23	0.30	4.07	0.00	0.00
ENSSSCG00000001834	MFGE8	2738.10	1.23	0.05	24.83	0.00	0.00
ENSSSCG00000015113	NLRX1	525.19	1.23	0.09	13.39	0.00	0.00
ENSSSCG00000007738	ASL	470.20	1.23	0.10	12.40	0.00	0.00
ENSSSCG00000006192	LACTB2	1340.50	1.23	0.06	19.38	0.00	0.00
ENSSSCG00000006175	LY96	202.39	1.24	0.15	8.40	0.00	0.00
ENSSSCG00000017543	CALCOCO2	3807.91	1.24	0.07	18.75	0.00	0.00
ENSSSCG00000020837		4669.39	1.24	0.05	26.90	0.00	0.00
ENSSSCG00000017566	ACSF2	1403.63	1.24	0.07	18.20	0.00	0.00
ENSSSCG00000022693	LBX1	34.62	1.24	0.34	3.69	0.00	0.00
ENSSSCG00000012055	MORC3	420.92	1.24	0.10	12.05	0.00	0.00
ENSSSCG00000032369	HSD3B7	116.42	1.24	0.19	6.55	0.00	0.00
ENSSSCG00000033167	SLC7A6OS	1229.51	1.24	0.07	18.55	0.00	0.00
ENSSSCG00000035583	PKDREJ	16.45	1.24	0.48	2.56	0.01	0.02
ENSSSCG00000038436	HYPK	1379.29	1.24	0.07	17.79	0.00	0.00
ENSSSCG00000024296	NKIRAS1	115.37	1.24	0.19	6.61	0.00	0.00
ENSSSCG00000037168	TMEM92	382.36	1.24	0.11	11.53	0.00	0.00
ENSSSCG00000004142	CITED2	832.19	1.24	0.08	15.83	0.00	0.00

ENSSSCG00000017869	ITGAE	105.80	1.24	0.20	6.37	0.00	0.00
ENSSSCG00000014389	RELL2	203.66	1.24	0.15	8.46	0.00	0.00
ENSSSCG00000016593	ARF5	376.07	1.24	0.11	11.31	0.00	0.00
ENSSSCG00000009717	CBR4	428.14	1.24	0.10	11.86	0.00	0.00
ENSSSCG00000036062	RNF122	80.33	1.24	0.23	5.52	0.00	0.00
ENSSSCG00000031809	ZKSCAN5	189.63	1.24	0.15	8.50	0.00	0.00
ENSSSCG00000025881	PDHB	3712.78	1.24	0.05	23.88	0.00	0.00
ENSSSCG00000015331	PON2	349.79	1.25	0.11	11.38	0.00	0.00
ENSSSCG00000006392	IGSF8	188.78	1.25	0.17	7.51	0.00	0.00
ENSSSCG00000011567		459.20	1.25	0.10	12.24	0.00	0.00
ENSSSCG00000014029	COL23A1	62.85	1.25	0.26	4.76	0.00	0.00
ENSSSCG00000004133	FUCA2	1233.96	1.25	0.07	18.91	0.00	0.00
ENSSSCG00000024635	SPART	141.66	1.25	0.18	6.82	0.00	0.00
ENSSSCG00000002803	CCDC113	61.05	1.25	0.27	4.70	0.00	0.00
ENSSSCG00000009877	IQCD	122.71	1.25	0.18	6.75	0.00	0.00
ENSSSCG00000030263	CHRNB1	937.88	1.25	0.07	17.38	0.00	0.00
ENSSSCG00000009412	LCP1	274.72	1.25	0.13	9.77	0.00	0.00
ENSSSCG00000036172	SP9	49.16	1.25	0.30	4.14	0.00	0.00
ENSSSCG00000001242	GABBR1	204.02	1.25	0.14	8.82	0.00	0.00
ENSSSCG00000035790	BTG1	1604.42	1.25	0.06	20.10	0.00	0.00
ENSSSCG00000037360	CST3	10551.65	1.25	0.04	29.39	0.00	0.00
ENSSSCG00000033843	ETFRF1	66.21	1.25	0.26	4.90	0.00	0.00
ENSSSCG00000000728	PARP11	93.78	1.25	0.20	6.12	0.00	0.00
ENSSSCG00000017251	SOX9	412.12	1.25	0.11	11.77	0.00	0.00
ENSSSCG00000003421		632.28	1.25	0.09	14.68	0.00	0.00

ENSSSCG00000006800	CD53	123.63	1.26	0.19	6.65	0.00	0.00
ENSSSCG00000029129	RWDD2A	261.71	1.26	0.13	9.54	0.00	0.00
ENSSSCG00000037457	HSPB7	58.37	1.26	0.27	4.74	0.00	0.00
ENSSSCG00000027361	OVOL1	313.44	1.26	0.12	10.81	0.00	0.00
ENSSSCG00000037201	MPV17	305.44	1.26	0.12	10.72	0.00	0.00
ENSSSCG00000000156	FBXO7	804.77	1.26	0.08	16.31	0.00	0.00
ENSSSCG00000029352	CCNL1	2904.06	1.26	0.07	19.21	0.00	0.00
ENSSSCG00000008929		29.80	1.26	0.36	3.51	0.00	0.00
ENSSSCG00000001398		1312.64	1.26	0.06	19.46	0.00	0.00
ENSSSCG00000006820	EPS8L3	2658.49	1.26	0.05	24.41	0.00	0.00
ENSSSCG00000016782	OTULINL	26.38	1.26	0.39	3.27	0.00	0.00
ENSSSCG00000014558	SIRT3	572.69	1.26	0.09	13.67	0.00	0.00
ENSSSCG00000036772	CAMTA1	53.52	1.26	0.27	4.64	0.00	0.00
ENSSSCG00000010340	PRXL2A	14586.46	1.27	0.04	36.01	0.00	0.00
ENSSSCG00000008314	ATP6V1B1	62.20	1.27	0.25	5.08	0.00	0.00
ENSSSCG00000024626	TNFAIP1	1998.11	1.27	0.05	23.41	0.00	0.00
ENSSSCG00000010201	RASGEF1A	57.53	1.27	0.27	4.76	0.00	0.00
ENSSSCG00000031666	C11orf95	42.62	1.27	0.32	3.97	0.00	0.00
ENSSSCG00000033816		359.49	1.27	0.11	11.27	0.00	0.00
ENSSSCG00000014948	C11orf54	749.46	1.27	0.09	14.53	0.00	0.00
ENSSSCG00000038403		232.04	1.27	0.14	9.24	0.00	0.00
ENSSSCG00000026706	PIGS	2897.68	1.27	0.05	24.66	0.00	0.00
ENSSSCG00000006995	ASAH1	3296.73	1.27	0.05	26.18	0.00	0.00
ENSSSCG00000035628	CCDC115	794.26	1.27	0.08	15.14	0.00	0.00
ENSSSCG00000038626	ZNF513	116.49	1.27	0.19	6.83	0.00	0.00

ENSSSCG00000017118	TERT	186.36	1.27	0.15	8.57	0.00	0.00
ENSSSCG00000008480	MORN2	387.37	1.27	0.11	11.92	0.00	0.00
ENSSSCG00000031122	HIST1H2BB	14.45	1.27	0.56	2.25	0.02	0.03
ENSSSCG00000010248	KIF1BP	994.32	1.27	0.07	17.45	0.00	0.00
ENSSSCG00000032181	C17orf58	75.11	1.27	0.23	5.54	0.00	0.00
ENSSSCG00000040411	CCDC69	33.54	1.27	0.34	3.70	0.00	0.00
ENSSSCG00000014898	DDIAS	2202.18	1.27	0.06	22.16	0.00	0.00
ENSSSCG00000014806	ANAPC15	446.64	1.27	0.10	12.86	0.00	0.00
ENSSSCG00000038161	ARL16	965.37	1.28	0.07	17.77	0.00	0.00
ENSSSCG00000035473		9.24	1.28	0.65	1.96	0.05	0.07
ENSSSCG00000038423	GLA	1591.87	1.28	0.06	21.72	0.00	0.00
ENSSSCG00000037513	GINS4	1335.63	1.28	0.06	20.07	0.00	0.00
ENSSSCG00000039267	ATP6V0B	2202.93	1.28	0.06	23.12	0.00	0.00
ENSSSCG00000035374	TRAPPC6A	460.51	1.28	0.10	13.16	0.00	0.00
ENSSSCG00000038772	IKBIP	88.58	1.28	0.21	5.97	0.00	0.00
ENSSSCG00000007149	SPEF1	343.61	1.28	0.11	11.29	0.00	0.00
ENSSSCG00000002988	ZNF546	104.18	1.28	0.20	6.35	0.00	0.00
ENSSSCG00000034407	AMN1	106.38	1.28	0.21	6.21	0.00	0.00
ENSSSCG00000013732	MAST1	37.08	1.28	0.33	3.82	0.00	0.00
ENSSSCG00000006252	TMEM68	648.53	1.28	0.08	15.30	0.00	0.00
ENSSSCG00000033880		156.47	1.28	0.17	7.70	0.00	0.00
ENSSSCG00000016140	FZD5	492.18	1.28	0.10	13.00	0.00	0.00
ENSSSCG00000017427	HAP1	1214.59	1.28	0.07	19.30	0.00	0.00
ENSSSCG00000023498	HSPB6	353.25	1.28	0.12	10.57	0.00	0.00
ENSSSCG00000037832	PMP22	37.70	1.28	0.32	3.95	0.00	0.00

ENSSSCG00000013586	LRRC8E	48.72	1.28	0.29	4.49	0.00	0.00
ENSSSCG00000032404	KLHL11	857.12	1.28	0.08	17.08	0.00	0.00
ENSSSCG00000010011	TCN2	1259.38	1.28	0.07	19.02	0.00	0.00
ENSSSCG00000000209	NCKAP5L	96.48	1.28	0.21	6.25	0.00	0.00
ENSSSCG00000022401	AGTRAP	1064.44	1.29	0.21	6.05	0.00	0.00
ENSSSCG00000037066	GADD45A	1963.03	1.29	0.06	21.58	0.00	0.00
ENSSSCG00000033991	DPP7	1047.90	1.29	0.07	18.04	0.00	0.00
ENSSSCG00000035600	DNAJC12	444.58	1.29	0.10	12.73	0.00	0.00
ENSSSCG00000015089	JAML	20.34	1.29	0.45	2.88	0.00	0.01
ENSSSCG00000040582		9.87	1.29	0.62	2.07	0.04	0.05
ENSSSCG00000015196	SIAE	120.43	1.29	0.19	6.79	0.00	0.00
ENSSSCG00000006504	KHDC4	2136.86	1.29	0.06	21.47	0.00	0.00
ENSSSCG00000029185	MAPRE3	287.32	1.29	0.12	10.44	0.00	0.00
ENSSSCG00000036314	HHEX	589.67	1.29	0.09	14.03	0.00	0.00
ENSSSCG00000002933	ZNF382	42.01	1.29	0.31	4.13	0.00	0.00
ENSSSCG00000006095	CCNE2	1315.66	1.29	0.06	20.01	0.00	0.00
ENSSSCG00000002781	KCTD19	16.88	1.29	0.51	2.55	0.01	0.02
ENSSSCG00000034045	ERGIC3	2088.07	1.29	0.05	23.53	0.00	0.00
ENSSSCG00000017518		624.09	1.29	0.09	14.91	0.00	0.00
ENSSSCG00000017541	HOXB13	249.47	1.29	0.13	9.87	0.00	0.00
ENSSSCG00000030358	SARM1	226.00	1.29	0.14	9.15	0.00	0.00
ENSSSCG00000004761	ZFYVE19	216.25	1.29	0.14	9.37	0.00	0.00
ENSSSCG00000004931	IGDCC4	19.26	1.29	0.46	2.84	0.00	0.01
ENSSSCG00000036582	MTLN	175.52	1.29	0.16	8.32	0.00	0.00
ENSSSCG00000001624	FRS3	68.26	1.29	0.24	5.38	0.00	0.00

ENSSSCG00000024555	ING2	326.51	1.29	0.12	10.79	0.00	0.00
ENSSSCG00000012523		25.72	1.30	0.39	3.31	0.00	0.00
ENSSSCG00000027401	OPRD1	12.28	1.30	0.58	2.22	0.03	0.04
ENSSSCG00000013978	ZNF672	194.65	1.30	0.15	8.67	0.00	0.00
ENSSSCG00000040377	FCER2	87.81	1.30	0.21	6.10	0.00	0.00
ENSSSCG00000008545	ZNF512	262.91	1.30	0.13	10.35	0.00	0.00
ENSSSCG00000001418	NEU1	661.46	1.30	0.09	14.74	0.00	0.00
ENSSSCG00000008197	SEMA4C	141.84	1.30	0.20	6.57	0.00	0.00
ENSSSCG00000032946		439.20	1.30	0.10	12.84	0.00	0.00
ENSSSCG00000007431	CTSA	2563.47	1.30	0.05	24.57	0.00	0.00
ENSSSCG00000017398	COASY	1098.61	1.30	0.07	19.44	0.00	0.00
ENSSSCG00000017766		324.44	1.31	0.12	11.32	0.00	0.00
ENSSSCG00000013570	MCOLN1	717.15	1.31	0.08	16.16	0.00	0.00
ENSSSCG00000026425	ADAMTSL2	11.78	1.31	0.60	2.18	0.03	0.04
ENSSSCG00000021053	GIPR	37.53	1.31	0.33	3.93	0.00	0.00
ENSSSCG00000008218	RNF103	286.03	1.31	0.13	10.25	0.00	0.00
ENSSSCG00000036709	OSCP1	220.89	1.31	0.14	9.31	0.00	0.00
ENSSSCG00000038038	SBK1	9.42	1.31	0.65	2.00	0.05	0.06
ENSSSCG00000029781	SELENOM	454.56	1.31	0.10	13.38	0.00	0.00
ENSSSCG00000036454	TMEM220	28.18	1.31	0.37	3.51	0.00	0.00
ENSSSCG00000016443	TMEM176A	412.08	1.31	0.11	11.69	0.00	0.00
ENSSSCG00000022347	WNT11	155.02	1.31	0.16	8.04	0.00	0.00
ENSSSCG00000024061	TNNI1	24.11	1.31	0.40	3.24	0.00	0.00
ENSSSCG00000040603	SGTB	85.72	1.31	0.22	5.89	0.00	0.00
ENSSSCG00000009633		3142.75	1.31	0.05	25.46	0.00	0.00

ENSSSCG00000029347		179.22	1.31	0.15	8.63	0.00	0.00
ENSSSCG00000005716	POMT1	514.23	1.31	0.10	13.76	0.00	0.00
ENSSSCG00000015958	PDK1	1253.26	1.31	0.09	14.67	0.00	0.00
ENSSSCG00000012911	CARNS1	16.42	1.31	0.51	2.57	0.01	0.01
ENSSSCG00000037459	TCTA	281.11	1.31	0.12	10.49	0.00	0.00
ENSSSCG00000014288	GDF9	297.63	1.31	0.12	11.06	0.00	0.00
ENSSSCG00000029666	HOXA13	147.81	1.31	0.17	7.68	0.00	0.00
ENSSSCG00000008240		693.05	1.31	0.09	14.73	0.00	0.00
ENSSSCG00000003582	SMPDL3B	166.84	1.31	0.17	7.94	0.00	0.00
ENSSSCG00000026167		24.79	1.32	0.43	3.09	0.00	0.00
ENSSSCG00000009491	TGDS	532.59	1.32	0.09	14.27	0.00	0.00
ENSSSCG00000026455	TMEM54	439.40	1.32	0.10	13.29	0.00	0.00
ENSSSCG00000005301	FAM214B	385.55	1.32	0.11	11.55	0.00	0.00
ENSSSCG00000004774	IVD	662.54	1.32	0.09	15.08	0.00	0.00
ENSSSCG00000015545	GLUL	4564.96	1.32	0.05	28.51	0.00	0.00
ENSSSCG00000038991	S100A13	179.75	1.32	0.15	8.77	0.00	0.00
ENSSSCG00000021741	ZNF527	18.93	1.32	0.45	2.91	0.00	0.01
ENSSSCG00000036136	BHLHE40	3832.94	1.32	0.06	22.14	0.00	0.00
ENSSSCG00000005650	CERCAM	628.52	1.32	0.09	14.90	0.00	0.00
ENSSSCG00000027076	CYHR1	858.21	1.32	0.07	17.81	0.00	0.00
ENSSSCG00000022672	TRPM4	107.79	1.32	0.20	6.49	0.00	0.00
ENSSSCG00000003198	FUZ	139.82	1.33	0.17	7.75	0.00	0.00
ENSSSCG00000000437	ARHGAP9	45.62	1.33	0.29	4.52	0.00	0.00
ENSSSCG00000039413	SLC9A3	123.90	1.33	0.18	7.25	0.00	0.00
ENSSSCG00000034727	CCDC24	207.07	1.33	0.16	8.56	0.00	0.00

ENSSSCG00000028204	TREH	27.21	1.33	0.43	3.06	0.00	0.00
ENSSSCG00000009875	CFAP73	27.87	1.33	0.38	3.52	0.00	0.00
ENSSSCG00000001952	NFKBIA	1519.14	1.33	0.08	16.21	0.00	0.00
ENSSSCG00000003077		11.88	1.33	0.57	2.32	0.02	0.03
ENSSSCG00000009769	GTF2H3	1360.53	1.33	0.07	19.08	0.00	0.00
ENSSSCG00000021232	SYNC	38.05	1.33	0.33	4.06	0.00	0.00
ENSSSCG00000001095	GMNN	799.48	1.33	0.08	16.49	0.00	0.00
ENSSSCG00000031913	NDUFB4	913.16	1.33	0.08	17.64	0.00	0.00
ENSSSCG00000038394	PAIP2B	444.34	1.33	0.10	12.78	0.00	0.00
ENSSSCG00000006080	ERICH5	292.36	1.33	0.12	10.92	0.00	0.00
ENSSSCG00000016290	EFHD1	28.50	1.33	0.38	3.47	0.00	0.00
ENSSSCG00000010182	CAPN9	176.11	1.33	0.16	8.49	0.00	0.00
ENSSSCG00000004180		8.93	1.33	0.66	2.03	0.04	0.06
ENSSSCG00000008602	LAPTM4A	5322.98	1.33	0.04	30.81	0.00	0.00
ENSSSCG00000006308	CREG1	903.56	1.33	0.07	17.97	0.00	0.00
ENSSSCG00000021259	CDA	1157.10	1.34	0.07	18.58	0.00	0.00
ENSSSCG00000000398	APOF	27.98	1.34	0.37	3.58	0.00	0.00
ENSSSCG00000000749	SLC6A12	477.52	1.34	0.11	12.17	0.00	0.00
ENSSSCG00000015559	NCF2	15.53	1.34	0.51	2.62	0.01	0.01
ENSSSCG00000009916	ACADS	1301.72	1.34	0.06	21.24	0.00	0.00
ENSSSCG00000007243	PLAGL2	1801.61	1.34	0.06	23.10	0.00	0.00
ENSSSCG00000002831	IRX3	174.39	1.34	0.16	8.58	0.00	0.00
ENSSSCG00000025842	SPAG8	503.95	1.34	0.10	12.83	0.00	0.00
ENSSSCG00000008828	OCIAD1	2073.71	1.34	0.06	24.34	0.00	0.00
ENSSSCG00000034868		539.26	1.34	0.11	12.01	0.00	0.00

ENSSSCG00000038700	FMC1	250.85	1.34	0.14	9.58	0.00	0.00
ENSSSCG00000033636	AFMID	1344.66	1.34	0.06	21.49	0.00	0.00
ENSSSCG00000025041		211.47	1.34	0.15	9.07	0.00	0.00
ENSSSCG00000033292	C1orf194	342.71	1.34	0.11	11.74	0.00	0.00
ENSSSCG00000000738	RHNO1	2456.76	1.35	0.05	25.39	0.00	0.00
ENSSSCG00000012276	SYN1	44.36	1.35	0.32	4.18	0.00	0.00
ENSSSCG00000011565	BRK1	2324.63	1.35	0.05	24.72	0.00	0.00
ENSSSCG00000034774	TBX6	127.04	1.35	0.18	7.54	0.00	0.00
ENSSSCG00000010179	ARV1	325.93	1.35	0.12	11.64	0.00	0.00
ENSSSCG00000038260		12.01	1.35	0.60	2.23	0.03	0.04
ENSSSCG00000031452	C14orf93	10.20	1.35	0.64	2.10	0.04	0.05
ENSSSCG00000038508	SPTBN2	785.08	1.35	0.08	16.79	0.00	0.00
ENSSSCG00000007308	ROMO1	807.34	1.35	0.08	17.27	0.00	0.00
ENSSSCG00000030513	CFAP74	139.66	1.35	0.18	7.42	0.00	0.00
ENSSSCG00000032949		239.47	1.36	0.14	9.85	0.00	0.00
ENSSSCG00000009945	FICD	255.05	1.36	0.13	10.32	0.00	0.00
ENSSSCG00000006632	SCNM1	768.34	1.36	0.08	17.42	0.00	0.00
ENSSSCG00000008727	MSX1	441.02	1.36	0.10	13.29	0.00	0.00
ENSSSCG00000032380	C1orf53	55.34	1.36	0.28	4.82	0.00	0.00
ENSSSCG00000032160		77.14	1.36	0.23	5.88	0.00	0.00
ENSSSCG00000040745	ZNF771	109.60	1.36	0.19	7.11	0.00	0.00
ENSSSCG00000035355	F2R	1803.67	1.36	0.06	23.86	0.00	0.00
ENSSSCG00000009824	RAD9B	27.74	1.36	0.39	3.46	0.00	0.00
ENSSSCG00000007427	ZSWIM3	89.30	1.36	0.22	6.22	0.00	0.00
ENSSSCG00000001342	TRIM39	117.10	1.36	0.19	7.22	0.00	0.00

ENSSSCG00000000139	IFT27	74.18	1.36	0.24	5.77	0.00	0.00
ENSSSCG00000012766	BGN	191.45	1.36	0.15	8.98	0.00	0.00
ENSSSCG00000039864	KLC3	80.37	1.36	0.23	6.05	0.00	0.00
ENSSSCG00000011264	CSRNP1	517.09	1.36	0.10	13.72	0.00	0.00
ENSSSCG00000012481	TSPAN6	1391.60	1.37	0.06	21.09	0.00	0.00
ENSSSCG00000000411	NAB2	346.86	1.37	0.12	11.62	0.00	0.00
ENSSSCG00000038753	RF00002	45.33	1.37	0.30	4.57	0.00	0.00
ENSSSCG00000001561	ETV7	14.52	1.37	0.53	2.60	0.01	0.01
ENSSSCG00000012678		9.08	1.37	0.66	2.08	0.04	0.05
ENSSSCG00000006842		461.39	1.37	0.11	12.81	0.00	0.00
ENSSSCG00000023636	TMEM222	643.50	1.37	0.09	15.91	0.00	0.00
ENSSSCG00000037395	CLIC3	15.17	1.37	0.51	2.70	0.01	0.01
ENSSSCG00000001582	MDGA1	85.61	1.37	0.22	6.17	0.00	0.00
ENSSSCG00000012960	CST6	256.15	1.37	0.13	10.66	0.00	0.00
ENSSSCG00000005512	C5	13.36	1.38	0.57	2.42	0.02	0.02
ENSSSCG00000033003	WFDC2	1548.69	1.38	0.06	22.16	0.00	0.00
ENSSSCG00000017947	ACADVL	2933.65	1.38	0.05	28.10	0.00	0.00
ENSSSCG00000016262		910.83	1.38	0.08	17.94	0.00	0.00
ENSSSCG00000005688	PTGES	1897.17	1.38	0.06	24.32	0.00	0.00
ENSSSCG00000036608		31.07	1.38	0.38	3.65	0.00	0.00
ENSSSCG00000026042	ULBP1	455.46	1.38	0.10	13.56	0.00	0.00
ENSSSCG00000009542	TNFSF13B	14.02	1.38	0.53	2.61	0.01	0.01
ENSSSCG00000004530	STARD6	48.21	1.38	0.29	4.77	0.00	0.00
ENSSSCG00000039327	DAPP1	69.56	1.38	0.24	5.65	0.00	0.00
ENSSSCG00000013008	CDCA5	2278.65	1.39	0.06	22.96	0.00	0.00

ENSSSCG00000002637		21.37	1.39	0.43	3.23	0.00	0.00
ENSSSCG00000003257	CACNG6	26.87	1.39	0.39	3.60	0.00	0.00
ENSSSCG00000022980	TBX4	53.84	1.39	0.28	5.03	0.00	0.00
ENSSSCG00000001849	ANPEP	96.62	1.39	0.21	6.63	0.00	0.00
ENSSSCG00000012933	CCDC87	45.33	1.39	0.30	4.58	0.00	0.00
ENSSSCG00000023160		104.73	1.39	0.20	7.10	0.00	0.00
ENSSSCG00000038964	COX19	268.34	1.39	0.13	10.75	0.00	0.00
ENSSSCG00000008474		36.73	1.39	0.33	4.24	0.00	0.00
ENSSSCG00000017953	FXR2	1551.31	1.39	0.06	21.88	0.00	0.00
ENSSSCG00000017918	ARRB2	1314.70	1.39	0.06	21.56	0.00	0.00
ENSSSCG00000000045	NDUFA6	986.93	1.39	0.08	18.11	0.00	0.00
ENSSSCG00000007754	ITGAM	632.78	1.39	0.09	15.18	0.00	0.00
ENSSSCG00000021638	NEU3	329.83	1.39	0.12	11.73	0.00	0.00
ENSSSCG00000007079	FLRT3	1362.29	1.39	0.08	17.74	0.00	0.00
ENSSSCG00000011404	HYAL1	352.83	1.39	0.12	11.99	0.00	0.00
ENSSSCG00000001629	USP49	15.92	1.39	0.51	2.73	0.01	0.01
ENSSSCG00000007987	DECR2	761.17	1.39	0.08	17.77	0.00	0.00
ENSSSCG00000013593		21.52	1.40	0.44	3.21	0.00	0.00
ENSSSCG00000008096	ZC3H6	301.34	1.40	0.13	10.74	0.00	0.00
ENSSSCG00000036684	TTPAL	908.55	1.40	0.07	19.23	0.00	0.00
ENSSSCG00000010518	EXOSC1	1346.50	1.40	0.06	22.37	0.00	0.00
ENSSSCG00000022784	PLEKHO1	1288.54	1.40	0.07	20.51	0.00	0.00
ENSSSCG00000000182	WNT10B	173.87	1.40	0.15	9.07	0.00	0.00
ENSSSCG00000009216	SPP1	36243.65	1.40	0.04	36.33	0.00	0.00
ENSSSCG00000004390	SESN1	117.96	1.40	0.19	7.45	0.00	0.00

ENSSSCG00000021270	THAP2	492.61	1.40	0.11	12.94	0.00	0.00
ENSSSCG00000009378	CKAP2	3448.24	1.40	0.06	24.39	0.00	0.00
ENSSSCG00000007249	NOL4L	691.62	1.40	0.09	15.45	0.00	0.00
ENSSSCG00000005575	RC3H2	1629.54	1.40	0.06	22.22	0.00	0.00
ENSSSCG00000013760		48.08	1.40	0.29	4.77	0.00	0.00
ENSSSCG00000017087	GM2A	482.72	1.40	0.10	14.54	0.00	0.00
ENSSSCG00000001801	BTBD1	2077.08	1.40	0.06	22.69	0.00	0.00
ENSSSCG00000005627	AK1	360.30	1.40	0.12	11.64	0.00	0.00
ENSSSCG00000036941	AKR1E2	505.77	1.40	0.09	14.95	0.00	0.00
ENSSSCG00000036063	LPAR6	274.26	1.40	0.13	11.00	0.00	0.00
ENSSSCG00000011767	TTC14	1317.15	1.40	0.07	19.14	0.00	0.00
ENSSSCG00000031352	TCTEX1D1	12.37	1.41	0.61	2.31	0.02	0.03
ENSSSCG00000034189	LDOC1	696.46	1.41	0.09	15.10	0.00	0.00
ENSSSCG00000037461	MLF1	672.64	1.41	0.08	17.11	0.00	0.00
ENSSSCG00000014287	SHROOM1	67.87	1.41	0.24	5.77	0.00	0.00
ENSSSCG00000033303	MBD4	857.72	1.41	0.08	18.02	0.00	0.00
ENSSSCG00000036933	NR1D1	176.92	1.41	0.16	8.84	0.00	0.00
ENSSSCG00000001507	TAPBP	2967.34	1.41	0.05	26.77	0.00	0.00
ENSSSCG00000023195		379.01	1.41	0.11	13.10	0.00	0.00
ENSSSCG00000021581	CLCN2	248.10	1.41	0.14	10.14	0.00	0.00
ENSSSCG00000017617	SCPEP1	2096.07	1.41	0.06	25.59	0.00	0.00
ENSSSCG00000011673		512.27	1.41	0.10	14.49	0.00	0.00
ENSSSCG00000023890	ATG2A	812.80	1.41	0.08	17.49	0.00	0.00
ENSSSCG00000007237	PDRG1	1342.90	1.41	0.07	20.46	0.00	0.00
ENSSSCG00000028381	FLYWCH1	301.65	1.41	0.12	11.55	0.00	0.00

ENSSSCG00000006529	SLC50A1	862.50	1.41	0.07	19.06	0.00	0.00
ENSSSCG00000000261	IGFBP6	306.69	1.41	0.12	11.69	0.00	0.00
ENSSSCG00000038550		1196.91	1.42	0.09	15.66	0.00	0.00
ENSSSCG00000014035	B4GALT7	119.06	1.42	0.19	7.55	0.00	0.00
ENSSSCG00000014628	APBB1	219.74	1.42	0.14	10.25	0.00	0.00
ENSSSCG00000026392	BSDC1	1057.12	1.42	0.07	20.42	0.00	0.00
ENSSSCG00000032157	GVQW3	408.25	1.42	0.11	13.23	0.00	0.00
ENSSSCG00000035615	GSTK1	588.32	1.42	0.09	16.05	0.00	0.00
ENSSSCG00000020803	BRPF3	917.43	1.42	0.07	19.15	0.00	0.00
ENSSSCG00000009881	OAS2	1054.47	1.42	0.07	19.69	0.00	0.00
ENSSSCG00000035928	CLDN3	49.74	1.42	0.28	5.04	0.00	0.00
ENSSSCG00000031612	ZNF570	113.05	1.42	0.20	7.03	0.00	0.00
ENSSSCG00000017542	TTLL6	28.01	1.42	0.38	3.78	0.00	0.00
ENSSSCG00000014625	TRIM3	29.91	1.42	0.37	3.82	0.00	0.00
ENSSSCG00000038650	TLCD1	868.78	1.42	0.10	14.73	0.00	0.00
ENSSSCG00000003063	PHLDB3	122.06	1.42	0.20	7.24	0.00	0.00
ENSSSCG00000040071		107.79	1.42	0.19	7.35	0.00	0.00
ENSSSCG00000017789	ABHD15	61.05	1.43	0.26	5.45	0.00	0.00
ENSSSCG00000023684	MT1A	203.69	1.43	0.15	9.83	0.00	0.00
ENSSSCG00000009309		120.25	1.43	0.19	7.69	0.00	0.00
ENSSSCG00000004917	MALT1	505.15	1.43	0.10	13.71	0.00	0.00
ENSSSCG00000005316	TPM2	84.20	1.43	0.22	6.40	0.00	0.00
ENSSSCG00000032098		14.35	1.43	0.52	2.72	0.01	0.01
ENSSSCG00000000197	C1QL4	11.85	1.43	0.58	2.46	0.01	0.02
ENSSSCG00000017343	GFAP	155.97	1.43	0.17	8.51	0.00	0.00

ENSSSCG00000004954	C15orf61	83.66	1.43	0.22	6.39	0.00	0.00
ENSSSCG00000028943	ECH1	268.57	1.43	0.13	11.00	0.00	0.00
ENSSSCG00000002449		359.70	1.43	0.11	12.85	0.00	0.00
ENSSSCG00000033539	CRYBG2	307.52	1.43	0.12	11.90	0.00	0.00
ENSSSCG00000026731	CEP85	576.66	1.43	0.09	15.82	0.00	0.00
ENSSSCG00000003354	CCNL2	3020.40	1.44	0.05	27.39	0.00	0.00
ENSSSCG00000032392	MYO15A	35.02	1.44	0.35	4.12	0.00	0.00
ENSSSCG00000013308	FBXO3	442.82	1.44	0.11	13.56	0.00	0.00
ENSSSCG00000009625		807.85	1.44	0.08	17.76	0.00	0.00
ENSSSCG00000008237	RETSAT	2391.55	1.44	0.05	27.75	0.00	0.00
ENSSSCG00000001131	BTN2A2	63.39	1.44	0.26	5.43	0.00	0.00
ENSSSCG00000039405		34.45	1.44	0.36	3.96	0.00	0.00
ENSSSCG00000031660	MAPK8IP2	245.88	1.44	0.13	10.99	0.00	0.00
ENSSSCG00000013753	IER2	596.82	1.44	0.11	13.00	0.00	0.00
ENSSSCG00000035037		667.29	1.44	0.09	15.72	0.00	0.00
ENSSSCG00000013767	PALM3	32.06	1.44	0.36	4.02	0.00	0.00
ENSSSCG00000000370	DGKA	1567.75	1.44	0.06	24.52	0.00	0.00
ENSSSCG00000033505		207.37	1.44	0.14	10.25	0.00	0.00
ENSSSCG00000015779	CDKN2AIP	493.16	1.44	0.10	14.08	0.00	0.00
ENSSSCG00000039731		2638.16	1.45	0.06	25.34	0.00	0.00
ENSSSCG00000029029	ZNF713	29.59	1.45	0.38	3.84	0.00	0.00
ENSSSCG00000007866	TMC7	219.58	1.45	0.14	10.38	0.00	0.00
ENSSSCG00000032977	PPP1R3E	330.64	1.45	0.12	12.50	0.00	0.00
ENSSSCG00000006528	DPM3	300.48	1.45	0.12	12.21	0.00	0.00
ENSSSCG00000025281	ARL6IP1	8361.55	1.45	0.05	32.08	0.00	0.00

ENSSSCG00000040461	CDKN1C	11.37	1.45	0.59	2.46	0.01	0.02
ENSSSCG00000037184	GPR4	191.46	1.45	0.15	9.79	0.00	0.00
ENSSSCG00000013894	IL12RB1	27.82	1.45	0.39	3.68	0.00	0.00
ENSSSCG00000027711	SS18L2	575.83	1.45	0.09	15.89	0.00	0.00
ENSSSCG00000012038	C21orf62	22.77	1.45	0.42	3.50	0.00	0.00
ENSSSCG00000031786	NCDN	1934.35	1.45	0.06	24.62	0.00	0.00
ENSSSCG00000015079	BACE1	294.09	1.46	0.13	11.27	0.00	0.00
ENSSSCG00000005910	TMEM249	15.82	1.46	0.50	2.91	0.00	0.01
ENSSSCG00000002805	CFAP20	964.33	1.46	0.07	19.87	0.00	0.00
ENSSSCG00000023228	NUMBL	79.24	1.46	0.23	6.30	0.00	0.00
ENSSSCG00000000103	DMC1	8.26	1.46	0.70	2.08	0.04	0.05
ENSSSCG00000014168	ELL2	388.20	1.46	0.11	12.70	0.00	0.00
ENSSSCG00000034990		71.69	1.46	0.24	6.17	0.00	0.00
ENSSSCG00000039591	SERINC3	3080.31	1.46	0.05	28.07	0.00	0.00
ENSSSCG00000034743		218.29	1.46	0.15	9.92	0.00	0.00
ENSSSCG00000024990	MPI	1152.69	1.46	0.08	19.49	0.00	0.00
ENSSSCG00000021712	HERC6	211.48	1.46	0.14	10.23	0.00	0.00
ENSSSCG00000012790	ARHGAP4	24.85	1.46	0.44	3.35	0.00	0.00
ENSSSCG00000006370	TSTD1	455.95	1.47	0.10	14.55	0.00	0.00
ENSSSCG00000000707	SCNN1A	284.90	1.47	0.13	11.26	0.00	0.00
ENSSSCG00000002366	NPC2	3344.91	1.47	0.05	30.45	0.00	0.00
ENSSSCG00000006525		178.20	1.47	0.15	9.55	0.00	0.00
ENSSSCG00000016267	ITM2C	2229.87	1.47	0.05	27.22	0.00	0.00
ENSSSCG00000001070	FAM8A1	399.95	1.47	0.11	13.07	0.00	0.00
ENSSSCG00000008613	GEN1	752.81	1.47	0.09	16.44	0.00	0.00

ENSSSCG00000007778	CCDC189	63.80	1.47	0.25	5.79	0.00	0.00
ENSSSCG00000022322	BCL2L11	178.62	1.47	0.16	9.19	0.00	0.00
ENSSSCG00000008029	BAIAP3	35.10	1.47	0.34	4.32	0.00	0.00
ENSSSCG00000039183	NDUFAF8	212.13	1.48	0.14	10.49	0.00	0.00
ENSSSCG00000032003		9434.01	1.48	0.05	29.99	0.00	0.00
ENSSSCG00000017340	DCAKD	698.98	1.48	0.09	16.78	0.00	0.00
ENSSSCG00000033578		24.30	1.48	0.43	3.43	0.00	0.00
ENSSSCG00000013030	PRDX5	634.12	1.48	0.08	17.50	0.00	0.00
ENSSSCG00000000700	ACRBP	244.22	1.48	0.14	10.78	0.00	0.00
ENSSSCG00000040351		12.78	1.48	0.56	2.62	0.01	0.01
ENSSSCG00000027890		119.90	1.48	0.19	7.92	0.00	0.00
ENSSSCG00000000133	TST	817.67	1.48	0.09	17.09	0.00	0.00
ENSSSCG00000005437		1667.29	1.48	0.07	21.81	0.00	0.00
ENSSSCG00000035755	SHISA8	29.53	1.48	0.38	3.95	0.00	0.00
ENSSSCG00000006543	ADAR	1338.06	1.48	0.06	23.08	0.00	0.00
ENSSSCG00000006917	LRRC8C	19.22	1.48	0.47	3.18	0.00	0.00
ENSSSCG00000023737	CSF2	80.98	1.49	0.25	5.87	0.00	0.00
ENSSSCG00000013018	CDC42BPG	823.38	1.49	0.08	19.02	0.00	0.00
ENSSSCG00000030300		773.30	1.49	0.09	15.78	0.00	0.00
ENSSSCG00000033788	OXLD1	353.92	1.49	0.12	12.28	0.00	0.00
ENSSSCG00000008202	CNNM4	828.40	1.49	0.08	19.65	0.00	0.00
ENSSSCG00000017565	SPATA20	773.98	1.49	0.08	19.05	0.00	0.00
ENSSSCG00000029649	IFT46	577.65	1.49	0.09	16.39	0.00	0.00
ENSSSCG00000014073	HEXB	1101.23	1.49	0.07	21.31	0.00	0.00
ENSSSCG00000010128		394.42	1.49	0.11	13.73	0.00	0.00

ENSSSCG00000008985	SOWAHB	41.85	1.49	0.33	4.55	0.00	0.00
ENSSSCG00000006396	IGSF9	228.23	1.49	0.16	9.29	0.00	0.00
ENSSSCG00000033824		7.10	1.50	0.74	2.01	0.04	0.06
ENSSSCG00000040412		152.22	1.50	0.17	8.57	0.00	0.00
ENSSSCG00000003459	CELA2A	182.62	1.50	0.16	9.46	0.00	0.00
ENSSSCG00000023691		16.15	1.50	0.52	2.87	0.00	0.01
ENSSSCG00000028452	ERAS	8.40	1.50	0.70	2.13	0.03	0.05
ENSSSCG00000002353	FAM161B	345.60	1.50	0.11	13.05	0.00	0.00
ENSSSCG00000017134	FN3KRP	409.90	1.50	0.11	13.82	0.00	0.00
ENSSSCG00000040136	RF00614	16.16	1.50	0.53	2.81	0.01	0.01
ENSSSCG00000010060	UPB1	33.66	1.50	0.35	4.29	0.00	0.00
ENSSSCG00000027786	ZBTB21	126.69	1.50	0.19	7.85	0.00	0.00
ENSSSCG00000036575	USHBP1	34.29	1.50	0.35	4.32	0.00	0.00
ENSSSCG00000038867	PPM1K	283.95	1.50	0.13	12.00	0.00	0.00
ENSSSCG00000002938		20.11	1.50	0.45	3.31	0.00	0.00
ENSSSCG00000020591	RF00100	8.43	1.50	0.71	2.12	0.03	0.05
ENSSSCG00000006391	ATP1A2	105.02	1.50	0.21	7.29	0.00	0.00
ENSSSCG00000006518	HCN3	121.21	1.50	0.19	7.80	0.00	0.00
ENSSSCG00000011440	SEMA3G	339.92	1.50	0.12	13.03	0.00	0.00
ENSSSCG00000022058		960.39	1.50	0.08	20.04	0.00	0.00
ENSSSCG00000024587	DNASE1L2	12.98	1.50	0.56	2.69	0.01	0.01
ENSSSCG00000040088		11.67	1.51	0.59	2.57	0.01	0.01
ENSSSCG00000027428	ENHO	127.95	1.51	0.18	8.36	0.00	0.00
ENSSSCG00000004807	SCG5	15.61	1.51	0.54	2.81	0.00	0.01
ENSSSCG00000006560	CREB3L4	46.17	1.51	0.30	5.04	0.00	0.00

ENSSSCG00000017231	NAT9	220.39	1.51	0.14	10.59	0.00	0.00
ENSSSCG00000022370	TNFSF9	623.37	1.51	0.09	16.45	0.00	0.00
ENSSSCG00000036905		11.70	1.51	0.61	2.47	0.01	0.02
ENSSSCG00000005943	ST3GAL1	9.73	1.51	0.66	2.29	0.02	0.03
ENSSSCG00000036183	MCIDAS	12.34	1.51	0.58	2.62	0.01	0.01
ENSSSCG00000005219	CDC37L1	438.42	1.51	0.11	14.01	0.00	0.00
ENSSSCG00000036603		357.09	1.51	0.11	13.41	0.00	0.00
ENSSSCG00000031594		268.02	1.51	0.13	12.08	0.00	0.00
ENSSSCG00000005576	ZBTB6	191.43	1.51	0.16	9.59	0.00	0.00
ENSSSCG00000028190	HOMEZ	455.66	1.51	0.10	14.76	0.00	0.00
ENSSSCG00000017904	ENO3	39.76	1.52	0.33	4.55	0.00	0.00
ENSSSCG00000010491		36.52	1.52	0.33	4.57	0.00	0.00
ENSSSCG00000033913	C1RL	53.49	1.52	0.28	5.51	0.00	0.00
ENSSSCG00000001659	KLC4	601.23	1.52	0.09	16.29	0.00	0.00
ENSSSCG00000021946	ZNF383	20.91	1.52	0.44	3.43	0.00	0.00
ENSSSCG00000008590	MFSD2B	18.90	1.52	0.50	3.06	0.00	0.00
ENSSSCG00000034461		17.64	1.52	0.48	3.20	0.00	0.00
ENSSSCG00000009668	CLU	14560.81	1.52	0.04	40.70	0.00	0.00
ENSSSCG00000039469	DNAL1	742.66	1.52	0.08	18.13	0.00	0.00
ENSSSCG00000005016	L2HGDH	15.04	1.52	0.56	2.74	0.01	0.01
ENSSSCG00000030209	MFNG	28.80	1.52	0.40	3.82	0.00	0.00
ENSSSCG00000024827	ABTB1	353.70	1.52	0.11	13.35	0.00	0.00
ENSSSCG00000004989	FBXO33	438.39	1.53	0.11	14.33	0.00	0.00
ENSSSCG00000001396		355.28	1.53	0.11	13.70	0.00	0.00
ENSSSCG00000006810	KCNC4	7.86	1.53	0.73	2.10	0.04	0.05

ENSSSCG00000017296	ACE	59.15	1.53	0.26	5.83	0.00	0.00
ENSSSCG00000026585	PROCR	161.05	1.53	0.16	9.43	0.00	0.00
ENSSSCG00000010076	ZNF70	26.27	1.53	0.41	3.71	0.00	0.00
ENSSSCG00000026940	CASP10	920.26	1.53	0.08	18.52	0.00	0.00
ENSSSCG00000016826	RAD1	462.81	1.53	0.10	15.59	0.00	0.00
ENSSSCG00000035218	ADA2	70.52	1.53	0.24	6.30	0.00	0.00
ENSSSCG00000021588	DAPK2	11.86	1.54	0.58	2.64	0.01	0.01
ENSSSCG00000007305	SPAG4	17.77	1.54	0.49	3.17	0.00	0.00
ENSSSCG00000035057	RUNDC1	817.88	1.54	0.08	19.95	0.00	0.00
ENSSSCG00000040778	NIPA1	971.13	1.54	0.08	18.52	0.00	0.00
ENSSSCG00000007312	SCAND1	1363.38	1.54	0.08	19.96	0.00	0.00
ENSSSCG00000002675	DNAAF1	8.55	1.54	0.71	2.15	0.03	0.04
ENSSSCG00000008981		223.04	1.54	0.14	11.26	0.00	0.00
ENSSSCG00000020696	HMGN4	128.06	1.54	0.19	8.30	0.00	0.00
ENSSSCG00000025109	PLD3	1404.12	1.54	0.07	23.62	0.00	0.00
ENSSSCG00000000893	AMDHD1	102.97	1.54	0.20	7.51	0.00	0.00
ENSSSCG00000013505	FSD1	111.70	1.54	0.20	7.72	0.00	0.00
ENSSSCG00000005713	PLPP7	44.90	1.54	0.30	5.09	0.00	0.00
ENSSSCG00000010309	ZSWIM8	2494.45	1.54	0.05	29.74	0.00	0.00
ENSSSCG00000004554	PCLAF	3262.62	1.54	0.07	21.24	0.00	0.00
ENSSSCG00000034843		485.02	1.54	0.10	15.73	0.00	0.00
ENSSSCG00000008036	SYNGR3	54.20	1.54	0.28	5.58	0.00	0.00
ENSSSCG00000032446	C1orf116	1112.28	1.54	0.07	21.57	0.00	0.00
ENSSSCG00000007200	FAM110A	274.37	1.54	0.13	12.12	0.00	0.00
ENSSSCG00000037420	TMEM170B	108.44	1.54	0.21	7.39	0.00	0.00

ENSSSCG00000012693		45.02	1.54	0.30	5.07	0.00	0.00
ENSSSCG00000031531	TSPAN10	41.65	1.54	0.31	4.91	0.00	0.00
ENSSSCG00000010006		503.85	1.54	0.10	16.04	0.00	0.00
ENSSSCG00000036868	CTNNBIP1	84.11	1.54	0.23	6.74	0.00	0.00
ENSSSCG00000016652	LRRN3	37.07	1.54	0.33	4.70	0.00	0.00
ENSSSCG00000005089	SIX4	492.75	1.55	0.10	15.60	0.00	0.00
ENSSSCG00000001463	PSMB9	124.83	1.55	0.19	8.29	0.00	0.00
ENSSSCG00000005740	SARDH	58.39	1.55	0.27	5.64	0.00	0.00
ENSSSCG00000026177	SLC25A40	1515.39	1.55	0.07	23.85	0.00	0.00
ENSSSCG00000012944	PELI3	57.06	1.55	0.28	5.51	0.00	0.00
ENSSSCG00000014060		1728.14	1.55	0.06	27.04	0.00	0.00
ENSSSCG00000034207	CEBPB	1571.99	1.55	0.06	24.66	0.00	0.00
ENSSSCG00000016286	PRSS56	23.96	1.55	0.42	3.69	0.00	0.00
ENSSSCG00000014093	CRHBP	65.80	1.55	0.25	6.19	0.00	0.00
ENSSSCG00000000464	C12orf56	17.31	1.55	0.49	3.17	0.00	0.00
ENSSSCG00000036383	LGALS3BP	344.11	1.55	0.11	13.67	0.00	0.00
ENSSSCG00000018057	SOCS7	1059.23	1.55	0.07	21.98	0.00	0.00
ENSSSCG00000011377	LAMB2	2120.56	1.56	0.05	29.28	0.00	0.00
ENSSSCG00000026924	KIFC2	365.04	1.56	0.12	12.83	0.00	0.00
ENSSSCG00000003428	MTHFR	235.81	1.56	0.13	11.57	0.00	0.00
ENSSSCG00000031844		14.72	1.56	0.55	2.84	0.00	0.01
ENSSSCG00000039211		12.03	1.56	0.58	2.70	0.01	0.01
ENSSSCG00000039370		839.02	1.56	0.08	19.36	0.00	0.00
ENSSSCG00000003968	RIMKLA	147.81	1.56	0.17	8.99	0.00	0.00
ENSSSCG00000025561	VASN	155.72	1.56	0.17	9.25	0.00	0.00

ENSSSCG00000010577	ELOVL3	36.16	1.56	0.34	4.63	0.00	0.00
ENSSSCG00000036509	RWDD3	269.33	1.57	0.15	10.63	0.00	0.00
ENSSSCG00000014362	HBEGF	1440.27	1.57	0.07	24.07	0.00	0.00
ENSSSCG00000002783	SLC9A5	463.64	1.57	0.10	15.68	0.00	0.00
ENSSSCG00000001689	GTPBP2	481.05	1.57	0.10	15.94	0.00	0.00
ENSSSCG00000034450	ABHD10	414.88	1.57	0.11	14.24	0.00	0.00
ENSSSCG00000005297	DNAJB5	215.22	1.57	0.14	10.89	0.00	0.00
ENSSSCG00000027565		85.87	1.57	0.22	7.12	0.00	0.00
ENSSSCG00000034012	CASP3	2071.87	1.57	0.07	22.85	0.00	0.00
ENSSSCG00000010664	ENO4	103.48	1.57	0.21	7.65	0.00	0.00
ENSSSCG00000033207	SULT2A1	34.96	1.57	0.35	4.54	0.00	0.00
ENSSSCG00000030175	RF00377	9.38	1.57	0.67	2.34	0.02	0.03
ENSSSCG00000032063	THEM6	35.63	1.57	0.34	4.62	0.00	0.00
ENSSSCG00000011463	IL17RD	8.08	1.57	0.73	2.16	0.03	0.04
ENSSSCG00000026453	ACSM5	8.72	1.57	0.69	2.30	0.02	0.03
ENSSSCG00000013433	ADAMTSL5	365.75	1.57	0.11	14.36	0.00	0.00
ENSSSCG00000003981	ZFP69B	580.51	1.58	0.09	17.03	0.00	0.00
ENSSSCG00000002014	JPH4	7.40	1.58	0.74	2.15	0.03	0.04
ENSSSCG00000005368	XPA	341.54	1.58	0.11	13.77	0.00	0.00
ENSSSCG00000012845	CEND1	169.93	1.58	0.17	9.38	0.00	0.00
ENSSSCG00000012317	GSPT2	319.92	1.58	0.12	13.09	0.00	0.00
ENSSSCG00000013393	SPON1	20.85	1.58	0.47	3.39	0.00	0.00
ENSSSCG00000002955	CATSPERG	806.44	1.58	0.08	19.80	0.00	0.00
ENSSSCG00000010929	CYB5R1	83.60	1.58	0.24	6.69	0.00	0.00
ENSSSCG00000037371	TMPPE	108.85	1.58	0.20	7.90	0.00	0.00

ENSSSCG00000032752	ZBTB43	509.79	1.58	0.10	15.90	0.00	0.00
ENSSSCG00000008334	MXD1	848.70	1.58	0.08	20.38	0.00	0.00
ENSSSCG00000033348	ISL2	18.22	1.58	0.49	3.26	0.00	0.00
ENSSSCG00000005617	STXBP1	224.01	1.59	0.14	11.27	0.00	0.00
ENSSSCG00000006729	TENT5C	497.34	1.59	0.10	16.06	0.00	0.00
ENSSSCG00000038597	KLHDC9	60.92	1.59	0.27	5.87	0.00	0.00
ENSSSCG00000021815		94.18	1.59	0.21	7.46	0.00	0.00
ENSSSCG00000026502	LIX1L	1368.98	1.59	0.07	24.00	0.00	0.00
ENSSSCG00000006861	EXTL2	214.81	1.59	0.14	10.99	0.00	0.00
ENSSSCG00000012273	USP11	1033.88	1.59	0.07	21.41	0.00	0.00
ENSSSCG00000038003	ITK	11.53	1.59	0.60	2.65	0.01	0.01
ENSSSCG00000017402	STAT5A	471.87	1.59	0.10	15.92	0.00	0.00
ENSSSCG00000016550	KLF14	23.05	1.59	0.43	3.74	0.00	0.00
ENSSSCG00000008534	TOGARAM2	52.26	1.59	0.28	5.67	0.00	0.00
ENSSSCG00000003167	FLT3LG	69.38	1.59	0.25	6.31	0.00	0.00
ENSSSCG00000016873	NIM1K	14.94	1.60	0.52	3.05	0.00	0.00
ENSSSCG00000003922	UROD	690.78	1.60	0.08	18.96	0.00	0.00
ENSSSCG00000024669	NBL1	637.28	1.60	0.09	18.71	0.00	0.00
ENSSSCG00000037324		253.81	1.60	0.13	12.13	0.00	0.00
ENSSSCG00000009129	TIFA	250.47	1.60	0.15	10.46	0.00	0.00
ENSSSCG00000002337	ZFYVE1	487.63	1.60	0.10	16.30	0.00	0.00
ENSSSCG00000006531		67.47	1.60	0.25	6.28	0.00	0.00
ENSSSCG00000003219		85.82	1.60	0.22	7.15	0.00	0.00
ENSSSCG00000028706	MORN1	9.52	1.60	0.69	2.32	0.02	0.03
ENSSSCG00000024058	SLC5A2	77.69	1.60	0.23	6.87	0.00	0.00

ENSSSCG00000007145	ADAM33	159.07	1.60	0.17	9.61	0.00	0.00
ENSSSCG00000001427	C4A	28.66	1.60	0.39	4.12	0.00	0.00
ENSSSCG00000017884	TEKT1	115.41	1.60	0.20	8.13	0.00	0.00
ENSSSCG00000016218	PTPRN	12.29	1.61	0.59	2.74	0.01	0.01
ENSSSCG00000034036		13.66	1.61	0.55	2.91	0.00	0.01
ENSSSCG00000030388	UPP1	1481.69	1.61	0.08	21.16	0.00	0.00
ENSSSCG00000000010	FBLN1	33.58	1.61	0.37	4.39	0.00	0.00
ENSSSCG00000011407	RASSF1	523.27	1.61	0.10	15.62	0.00	0.00
ENSSSCG00000017300		55.47	1.61	0.27	5.88	0.00	0.00
ENSSSCG00000008384	C2orf74	8.92	1.61	0.67	2.40	0.02	0.02
ENSSSCG00000029760	CIPC	1505.52	1.61	0.06	26.41	0.00	0.00
ENSSSCG00000009209	NAP1L5	235.01	1.61	0.14	11.46	0.00	0.00
ENSSSCG00000021579		48.69	1.61	0.29	5.53	0.00	0.00
ENSSSCG00000003083	CBLC	96.64	1.61	0.21	7.56	0.00	0.00
ENSSSCG00000011014	BAMBI	78.94	1.61	0.23	6.89	0.00	0.00
ENSSSCG00000010104		236.95	1.61	0.14	11.75	0.00	0.00
ENSSSCG00000002817	DRC7	115.96	1.62	0.19	8.33	0.00	0.00
ENSSSCG00000013625	CCDC151	229.43	1.62	0.14	11.70	0.00	0.00
ENSSSCG00000002879	GRAMD1A	852.82	1.62	0.08	20.34	0.00	0.00
ENSSSCG00000014070	ANKRA2	454.17	1.62	0.10	15.88	0.00	0.00
ENSSSCG00000007426	ACOT8	267.74	1.62	0.13	12.64	0.00	0.00
ENSSSCG00000018056	SRCIN1	10.33	1.62	0.65	2.47	0.01	0.02
ENSSSCG00000017563	MYCBPAP	93.03	1.62	0.22	7.36	0.00	0.00
ENSSSCG00000026360	CLGN	1940.03	1.62	0.07	22.43	0.00	0.00
ENSSSCG00000026018	MFSD1	192.48	1.62	0.16	10.37	0.00	0.00

ENSSSCG00000011397	SLC38A3	49.59	1.62	0.30	5.38	0.00	0.00
ENSSSCG00000024418	RHOU	831.96	1.62	0.08	19.85	0.00	0.00
ENSSSCG00000011855	IQCG	12.40	1.62	0.61	2.67	0.01	0.01
ENSSSCG00000037154	SFTA2	36.60	1.62	0.34	4.76	0.00	0.00
ENSSSCG00000000292	ZNF385A	325.59	1.63	0.13	12.93	0.00	0.00
ENSSSCG00000016093		10.39	1.63	0.63	2.60	0.01	0.01
ENSSSCG00000002004		1395.93	1.63	0.07	23.81	0.00	0.00
ENSSSCG00000012485	NOX1	17.34	1.63	0.51	3.23	0.00	0.00
ENSSSCG00000017500	TCAP	6.23	1.63	0.82	2.00	0.05	0.06
ENSSSCG00000007382	PABPC1L	10.41	1.63	0.62	2.61	0.01	0.01
ENSSSCG00000013658	S1PR2	150.48	1.63	0.17	9.42	0.00	0.00
ENSSSCG00000035933	MAPK15	241.24	1.63	0.14	11.87	0.00	0.00
ENSSSCG00000035596		44.37	1.64	0.32	5.05	0.00	0.00
ENSSSCG00000015664		1428.34	1.64	0.07	24.35	0.00	0.00
ENSSSCG00000030548	HERC5	79.21	1.64	0.24	6.89	0.00	0.00
ENSSSCG00000015074	SIDT2	1093.41	1.64	0.07	22.51	0.00	0.00
ENSSSCG00000038848	IL22RA1	655.23	1.64	0.10	16.76	0.00	0.00
ENSSSCG00000038643	KLF11	201.98	1.64	0.15	11.27	0.00	0.00
ENSSSCG00000029763	IFI35	173.35	1.64	0.16	10.33	0.00	0.00
ENSSSCG00000011082	COMMD3	559.52	1.64	0.09	18.01	0.00	0.00
ENSSSCG00000032599		417.18	1.64	0.14	11.77	0.00	0.00
ENSSSCG00000004572		170.79	1.64	0.17	9.50	0.00	0.00
ENSSSCG00000005636	SLC25A25	560.60	1.64	0.10	16.92	0.00	0.00
ENSSSCG00000003395	PIK3CD	101.16	1.65	0.20	8.12	0.00	0.00
ENSSSCG00000038970	BCL2L15	344.14	1.65	0.12	14.05	0.00	0.00

ENSSSCG00000026517	CALML4	509.70	1.65	0.10	17.10	0.00	0.00
ENSSSCG00000008742	CD38	17.47	1.65	0.49	3.39	0.00	0.00
ENSSSCG00000013252	F2	46.80	1.65	0.30	5.52	0.00	0.00
ENSSSCG00000012482	SRPX2	30.07	1.65	0.38	4.32	0.00	0.00
ENSSSCG00000039953	PNRC1	604.57	1.65	0.10	16.64	0.00	0.00
ENSSSCG00000012910	RPS6KB2	321.58	1.65	0.12	13.57	0.00	0.00
ENSSSCG00000005638	LCN2	21.03	1.65	0.47	3.48	0.00	0.00
ENSSSCG00000002472		56.05	1.65	0.31	5.39	0.00	0.00
ENSSSCG00000040581	CISH	305.58	1.65	0.12	13.60	0.00	0.00
ENSSSCG00000004687	B2M	3416.16	1.65	0.07	24.48	0.00	0.00
ENSSSCG00000000089		14.05	1.66	0.55	3.01	0.00	0.00
ENSSSCG00000028964	PIM2	1376.73	1.66	0.06	25.75	0.00	0.00
ENSSSCG00000001011	SERPINB1	2642.49	1.66	0.05	30.87	0.00	0.00
ENSSSCG00000005643		1639.70	1.66	0.06	27.42	0.00	0.00
ENSSSCG00000021584	CDK5R2	7.76	1.66	0.74	2.24	0.02	0.03
ENSSSCG00000031274	F12	214.58	1.66	0.15	11.39	0.00	0.00
ENSSSCG00000030427	MIR590	7.71	1.66	0.79	2.11	0.03	0.05
ENSSSCG00000004237	SERINC1	1676.64	1.66	0.06	25.80	0.00	0.00
ENSSSCG00000003709	LAMA3	4060.51	1.67	0.05	36.56	0.00	0.00
ENSSSCG00000008668	RBAK	214.48	1.67	0.14	11.66	0.00	0.00
ENSSSCG00000025655		20.46	1.67	0.47	3.56	0.00	0.00
ENSSSCG00000022714	OSER1	1483.51	1.67	0.07	25.45	0.00	0.00
ENSSSCG00000015316		16.25	1.67	0.51	3.30	0.00	0.00
ENSSSCG00000027677		36.01	1.67	0.35	4.82	0.00	0.00
ENSSSCG00000020783	SLC41A1	1341.15	1.67	0.07	25.01	0.00	0.00

ENSSSCG00000009540	LIG4	764.56	1.67	0.08	20.77	0.00	0.00
ENSSSCG00000009627		36.18	1.68	0.35	4.83	0.00	0.00
ENSSSCG00000009410	RUBCNL	45.39	1.68	0.31	5.43	0.00	0.00
ENSSSCG00000017415	TTC25	212.69	1.68	0.14	11.66	0.00	0.00
ENSSSCG00000029503	F7	67.44	1.68	0.25	6.64	0.00	0.00
ENSSSCG00000000033	TSPO	1275.46	1.68	0.07	25.11	0.00	0.00
ENSSSCG00000015083	FXYD6	317.06	1.68	0.12	13.79	0.00	0.00
ENSSSCG00000016111	FZD7	272.94	1.69	0.13	12.56	0.00	0.00
ENSSSCG00000005494	TNC	53.38	1.69	0.30	5.57	0.00	0.00
ENSSSCG00000028699	NEIL2	65.58	1.69	0.27	6.16	0.00	0.00
ENSSSCG00000009742	ULK1	700.78	1.69	0.10	17.71	0.00	0.00
ENSSSCG00000015930	DHRS9	36.35	1.69	0.34	4.97	0.00	0.00
ENSSSCG00000010523	HOGA1	154.83	1.69	0.17	10.03	0.00	0.00
ENSSSCG00000004856	NFATC1	12.85	1.69	0.57	2.95	0.00	0.00
ENSSSCG00000008741	FGFBP1	20.70	1.69	0.45	3.76	0.00	0.00
ENSSSCG00000014072	ENC1	1043.62	1.69	0.08	20.33	0.00	0.00
ENSSSCG00000013440	ATP8B3	66.44	1.69	0.27	6.22	0.00	0.00
ENSSSCG00000034798	JKAMP	737.06	1.69	0.08	19.94	0.00	0.00
ENSSSCG00000037270	TCTEX1D4	25.02	1.69	0.42	4.01	0.00	0.00
ENSSSCG00000010580	NFKB2	3430.86	1.69	0.05	31.79	0.00	0.00
ENSSSCG00000007713	BUD23	1991.36	1.70	0.06	28.64	0.00	0.00
ENSSSCG00000032536	B3GNT8	7.88	1.70	0.72	2.35	0.02	0.03
ENSSSCG00000031456	ARL5B	565.08	1.70	0.12	13.86	0.00	0.00
ENSSSCG00000034778	ARTN	7.88	1.70	0.72	2.35	0.02	0.03
ENSSSCG00000038948	ETS2	1162.20	1.70	0.07	24.71	0.00	0.00

ENSSSCG00000007304		34.44	1.70	0.36	4.78	0.00	0.00
ENSSSCG00000012277	TIMP1	2760.86	1.70	0.05	31.62	0.00	0.00
ENSSSCG00000003766	DNAJB4	527.86	1.70	0.10	17.42	0.00	0.00
ENSSSCG00000012895		65.36	1.70	0.26	6.53	0.00	0.00
ENSSSCG00000030561	LMTK3	10.06	1.70	0.66	2.58	0.01	0.01
ENSSSCG00000021284	GPR20	17.99	1.70	0.49	3.49	0.00	0.00
ENSSSCG00000040922	IP6K2	479.77	1.70	0.10	17.14	0.00	0.00
ENSSSCG00000038829		15.09	1.70	0.52	3.25	0.00	0.00
ENSSSCG00000013569	PEX11G	38.85	1.70	0.33	5.17	0.00	0.00
ENSSSCG00000032249	GBX1	62.64	1.70	0.26	6.53	0.00	0.00
ENSSSCG00000002852	PLEKHF1	263.47	1.70	0.13	13.11	0.00	0.00
ENSSSCG00000007963	ZNF263	756.40	1.71	0.08	21.16	0.00	0.00
ENSSSCG00000031199		26.68	1.71	0.39	4.33	0.00	0.00
ENSSSCG00000007919		424.96	1.71	0.11	16.02	0.00	0.00
ENSSSCG00000040047	TMEM151B	33.93	1.71	0.36	4.79	0.00	0.00
ENSSSCG00000036096		7.94	1.71	0.73	2.34	0.02	0.03
ENSSSCG00000010839		5.79	1.71	0.84	2.03	0.04	0.06
ENSSSCG00000006474	NES	970.21	1.71	0.09	19.76	0.00	0.00
ENSSSCG00000034239	ZNF189	607.19	1.71	0.10	16.74	0.00	0.00
ENSSSCG00000010103	TUBA3D	7.94	1.71	0.79	2.17	0.03	0.04
ENSSSCG00000021569	MMP25	27.51	1.71	0.39	4.37	0.00	0.00
ENSSSCG00000012399	FOXO4	60.01	1.71	0.28	6.20	0.00	0.00
ENSSSCG00000006390	CASQ1	19.51	1.71	0.47	3.63	0.00	0.00
ENSSSCG00000010893		58.67	1.71	0.27	6.34	0.00	0.00
ENSSSCG00000032693	MAPK12	403.64	1.72	0.11	15.51	0.00	0.00

ENSSSCG00000038677	GJB3	203.21	1.72	0.15	11.22	0.00	0.00
ENSSSCG00000036129	RNF114	3473.07	1.72	0.05	35.24	0.00	0.00
ENSSSCG00000000773	TUBA8	14.54	1.72	0.54	3.20	0.00	0.00
ENSSSCG00000031011	CCDC160	205.46	1.72	0.15	11.75	0.00	0.00
ENSSSCG00000014286	SOWAHA	83.59	1.72	0.23	7.63	0.00	0.00
ENSSSCG00000031380	MARCKSL1	1573.93	1.72	0.07	25.90	0.00	0.00
ENSSSCG00000037566		111.98	1.72	0.20	8.48	0.00	0.00
ENSSSCG00000009477	EDNRB	13.09	1.72	0.58	2.96	0.00	0.00
ENSSSCG00000032395		16.04	1.72	0.56	3.10	0.00	0.00
ENSSSCG00000020178		6.55	1.73	0.81	2.15	0.03	0.04
ENSSSCG00000014390	FCHSD1	258.07	1.73	0.14	12.74	0.00	0.00
ENSSSCG00000013497	ANKRD24	34.25	1.73	0.36	4.81	0.00	0.00
ENSSSCG00000036839	ZNF34	145.99	1.73	0.17	10.14	0.00	0.00
ENSSSCG00000015732	TFCP2L1	12.41	1.73	0.60	2.86	0.00	0.01
ENSSSCG00000022384		8.80	1.73	0.71	2.43	0.01	0.02
ENSSSCG00000021328	PCSK1N	229.32	1.73	0.14	12.20	0.00	0.00
ENSSSCG00000006698	BCL9	910.78	1.73	0.08	20.80	0.00	0.00
ENSSSCG00000028192	DNAJC28	46.85	1.73	0.31	5.52	0.00	0.00
ENSSSCG00000001703	NFKBIE	1246.53	1.74	0.07	25.03	0.00	0.00
ENSSSCG00000034660	CCR4	24.16	1.74	0.42	4.13	0.00	0.00
ENSSSCG00000011171	CTSV	3815.42	1.74	0.05	37.65	0.00	0.00
ENSSSCG00000033703		1053.59	1.74	0.08	20.51	0.00	0.00
ENSSSCG00000001752	CHRNA3	14.68	1.74	0.53	3.27	0.00	0.00
ENSSSCG00000014349	TMEM173	281.83	1.74	0.13	13.74	0.00	0.00
ENSSSCG00000013147	FAM111B	1298.71	1.74	0.07	23.88	0.00	0.00

ENSSSCG00000023423	UBALD2	893.37	1.74	0.08	22.30	0.00	0.00
ENSSSCG00000013042		6.61	1.74	0.84	2.08	0.04	0.05
ENSSSCG00000003407	CASZ1	56.77	1.75	0.27	6.41	0.00	0.00
ENSSSCG00000027710	PDZD3	92.13	1.75	0.24	7.19	0.00	0.00
ENSSSCG00000040905	FAAP24	335.38	1.75	0.12	14.68	0.00	0.00
ENSSSCG00000000138	PVALB	5.92	1.75	0.88	2.00	0.05	0.06
ENSSSCG00000017615	DGKE	452.90	1.75	0.11	16.65	0.00	0.00
ENSSSCG00000009937	ACACB	22.94	1.76	0.43	4.08	0.00	0.00
ENSSSCG00000005828	EGFL7	109.55	1.76	0.20	8.67	0.00	0.00
ENSSSCG00000028481	CALCOCO1	469.42	1.76	0.10	17.36	0.00	0.00
ENSSSCG00000022500	IL20RB	7.40	1.76	0.76	2.31	0.02	0.03
ENSSSCG00000036387	ATPSCKMT	231.17	1.76	0.15	12.08	0.00	0.00
ENSSSCG00000014900	RAB30	82.38	1.76	0.24	7.45	0.00	0.00
ENSSSCG00000009592	NFIL3	844.17	1.76	0.08	20.97	0.00	0.00
ENSSSCG00000000396	STAT2	421.92	1.76	0.11	15.87	0.00	0.00
ENSSSCG00000022031	CHST2	5.94	1.76	0.85	2.08	0.04	0.05
ENSSSCG00000022866	NXF1	3535.29	1.76	0.06	31.18	0.00	0.00
ENSSSCG00000021867	HPN	71.35	1.76	0.26	6.80	0.00	0.00
ENSSSCG00000007212	C20orf96	8.18	1.77	0.75	2.35	0.02	0.03
ENSSSCG00000029571	AVIL	46.94	1.77	0.30	5.87	0.00	0.00
ENSSSCG00000014041	MXD3	375.07	1.77	0.11	16.03	0.00	0.00
ENSSSCG00000000443	GLI1	57.46	1.77	0.28	6.37	0.00	0.00
ENSSSCG00000037808		112.68	1.77	0.20	8.75	0.00	0.00
ENSSSCG00000023904	CFAP57	8.23	1.77	0.71	2.50	0.01	0.02
ENSSSCG00000024588		130.73	1.77	0.19	9.56	0.00	0.00

ENSSSCG00000012286	ZNF630	99.59	1.78	0.21	8.41	0.00	0.00
ENSSSCG00000038429		66.59	1.78	0.26	6.93	0.00	0.00
ENSSSCG00000017865	CTNS	595.55	1.78	0.09	19.43	0.00	0.00
ENSSSCG00000013599	ANGPTL4	458.01	1.78	0.11	16.53	0.00	0.00
ENSSSCG00000006196		8.27	1.78	0.72	2.46	0.01	0.02
ENSSSCG00000016754	AEBP1	24.81	1.78	0.42	4.20	0.00	0.00
ENSSSCG00000027013		297.37	1.78	0.13	14.06	0.00	0.00
ENSSSCG00000017261	ARSG	6.77	1.78	0.80	2.24	0.02	0.03
ENSSSCG00000017237	CD300LB	14.30	1.79	0.56	3.20	0.00	0.00
ENSSSCG00000031793	ZNF331	102.41	1.79	0.21	8.35	0.00	0.00
ENSSSCG00000029882	SPATA1	30.18	1.79	0.39	4.62	0.00	0.00
ENSSSCG00000017476	MSL1	1799.34	1.79	0.06	27.67	0.00	0.00
ENSSSCG00000040973	HYDIN	9.04	1.79	0.68	2.64	0.01	0.01
ENSSSCG00000006369	F11R	1586.39	1.79	0.07	27.45	0.00	0.00
ENSSSCG00000011423	GRM2	80.64	1.79	0.24	7.46	0.00	0.00
ENSSSCG00000032937		259.55	1.79	0.13	13.54	0.00	0.00
ENSSSCG00000029458	SLC16A2	5.29	1.79	0.89	2.02	0.04	0.06
ENSSSCG00000038918	CTSF	733.21	1.79	0.09	20.54	0.00	0.00
ENSSSCG00000012071		204.74	1.80	0.15	11.92	0.00	0.00
ENSSSCG00000024736	ACP4	220.70	1.80	0.14	12.58	0.00	0.00
ENSSSCG00000004746	ITPKA	97.61	1.80	0.21	8.41	0.00	0.00
ENSSSCG00000036865		10.59	1.80	0.66	2.74	0.01	0.01
ENSSSCG00000004956	SKOR1	28.80	1.80	0.40	4.51	0.00	0.00
ENSSSCG00000014274	PDLIM4	9.10	1.80	0.71	2.55	0.01	0.02
ENSSSCG00000023847	RF00156	6.06	1.80	0.86	2.09	0.04	0.05

ENSSSCG00000015175	VWA5A	187.49	1.80	0.17	10.77	0.00	0.00
ENSSSCG00000026257	STMN1	3245.84	1.80	0.05	34.20	0.00	0.00
ENSSSCG00000002792	HSF4	114.73	1.81	0.20	9.21	0.00	0.00
ENSSSCG00000023200		6.12	1.81	0.86	2.09	0.04	0.05
ENSSSCG00000028635		35.05	1.81	0.35	5.22	0.00	0.00
ENSSSCG00000008529	YPEL5	233.13	1.81	0.16	11.60	0.00	0.00
ENSSSCG00000024810	PRAM1	25.17	1.81	0.45	4.03	0.00	0.00
ENSSSCG00000004413	PPIL6	25.14	1.81	0.43	4.23	0.00	0.00
ENSSSCG00000001823	UROC1	6.12	1.81	0.86	2.12	0.03	0.05
ENSSSCG00000039135	ARHGAP33	64.08	1.81	0.26	7.01	0.00	0.00
ENSSSCG00000036787	APOLD1	35.85	1.81	0.35	5.18	0.00	0.00
ENSSSCG00000012618	SOWAHD	10.71	1.81	0.64	2.84	0.00	0.01
ENSSSCG00000016438	NUB1	2058.51	1.81	0.05	33.07	0.00	0.00
ENSSSCG00000012882		10.70	1.82	0.63	2.89	0.00	0.01
ENSSSCG00000037597		16.07	1.82	0.53	3.41	0.00	0.00
ENSSSCG00000029533	SEMA4G	104.83	1.82	0.22	8.44	0.00	0.00
ENSSSCG00000016196	VIL1	2765.44	1.82	0.06	33.07	0.00	0.00
ENSSSCG00000039587		6.88	1.82	0.79	2.30	0.02	0.03
ENSSSCG00000016808	C5orf22	264.85	1.82	0.15	12.23	0.00	0.00
ENSSSCG00000014336	EGR1	570.87	1.82	0.10	18.95	0.00	0.00
ENSSSCG00000005620	SH2D3C	69.88	1.82	0.25	7.29	0.00	0.00
ENSSSCG00000018052	NT5M	9.19	1.83	0.68	2.68	0.01	0.01
ENSSSCG00000003740	ZSCAN30	6.88	1.83	0.81	2.25	0.02	0.03
ENSSSCG00000008723	HTRA3	13.11	1.83	0.62	2.97	0.00	0.00
ENSSSCG00000040503	LRRC45	821.10	1.83	0.08	23.29	0.00	0.00

ENSSSCG00000028810		23.81	1.83	0.44	4.17	0.00	0.00
ENSSSCG00000002737	CHST4	158.68	1.83	0.17	10.59	0.00	0.00
ENSSSCG00000011874	PARP14	399.64	1.83	0.11	16.07	0.00	0.00
ENSSSCG00000007718	CLDN4	5162.65	1.83	0.05	38.16	0.00	0.00
ENSSSCG00000015550	RGS16	27.71	1.83	0.40	4.55	0.00	0.00
ENSSSCG00000007941	CDIP1	362.81	1.83	0.13	14.04	0.00	0.00
ENSSSCG00000009055	ELMOD2	519.33	1.83	0.11	15.95	0.00	0.00
ENSSSCG00000019154	ssc-mir-155	5.38	1.83	0.92	1.99	0.05	0.06
ENSSSCG00000023976	DCP1B	23.16	1.83	0.43	4.24	0.00	0.00
ENSSSCG00000031787		20.12	1.84	0.47	3.90	0.00	0.00
ENSSSCG00000021918	CEP19	369.96	1.84	0.13	14.64	0.00	0.00
ENSSSCG00000006530	EFNA1	1053.83	1.84	0.07	24.64	0.00	0.00
ENSSSCG00000010071	MMP11	1028.68	1.84	0.08	23.81	0.00	0.00
ENSSSCG00000021220	CKB	3526.23	1.84	0.05	34.34	0.00	0.00
ENSSSCG00000013735	JUNB	1511.06	1.84	0.07	25.01	0.00	0.00
ENSSSCG00000035249	GADD45G	26.35	1.84	0.41	4.52	0.00	0.00
ENSSSCG00000015489	SLC9C2	5.44	1.84	0.89	2.07	0.04	0.05
ENSSSCG00000032644	HLX	39.48	1.84	0.35	5.25	0.00	0.00
ENSSSCG00000031201	LMOD1	6.22	1.85	0.85	2.17	0.03	0.04
ENSSSCG00000031773		1380.36	1.85	0.07	25.82	0.00	0.00
ENSSSCG00000003908	TSPAN1	331.57	1.85	0.13	14.05	0.00	0.00
ENSSSCG00000007668	GIGYF1	608.77	1.85	0.10	19.11	0.00	0.00
ENSSSCG00000021040	SLC26A11	451.20	1.85	0.10	17.95	0.00	0.00
ENSSSCG00000025834	FNDC5	11.71	1.85	0.62	3.01	0.00	0.00
ENSSSCG00000001715	ENPP5	893.13	1.86	0.08	23.79	0.00	0.00

ENSSSCG00000017925	SLC16A11	242.02	1.86	0.14	13.26	0.00	0.00
ENSSSCG00000037479		5.46	1.86	0.93	2.00	0.05	0.06
ENSSSCG00000040282		9.37	1.86	0.68	2.72	0.01	0.01
ENSSSCG00000005916		71.93	1.86	0.25	7.52	0.00	0.00
ENSSSCG00000015965	GPR155	441.85	1.86	0.11	17.46	0.00	0.00
ENSSSCG00000036030	INKA2	38.35	1.86	0.34	5.54	0.00	0.00
ENSSSCG00000011263	TTC21A	138.64	1.86	0.18	10.47	0.00	0.00
ENSSSCG00000023166	RNF227	10.18	1.86	0.65	2.89	0.00	0.01
ENSSSCG00000013074	RAB3IL1	68.99	1.86	0.25	7.38	0.00	0.00
ENSSSCG00000036948		16.48	1.86	0.51	3.68	0.00	0.00
ENSSSCG00000011090	NEBL	10.19	1.86	0.64	2.90	0.00	0.01
ENSSSCG00000010559		33.71	1.87	0.36	5.11	0.00	0.00
ENSSSCG00000023693	PROC	46.30	1.87	0.31	6.11	0.00	0.00
ENSSSCG00000010772	ADAM8	73.81	1.87	0.25	7.47	0.00	0.00
ENSSSCG00000029570	PXDN	18.10	1.87	0.52	3.59	0.00	0.00
ENSSSCG00000024793	PORCN	200.45	1.87	0.15	12.53	0.00	0.00
ENSSSCG00000027555	DEFB1	10.22	1.87	0.67	2.78	0.01	0.01
ENSSSCG00000004737	PLA2G4B	159.56	1.87	0.18	10.51	0.00	0.00
ENSSSCG00000010292	P4HA1	2760.89	1.87	0.06	29.78	0.00	0.00
ENSSSCG00000034146	CBX8	311.43	1.87	0.12	14.98	0.00	0.00
ENSSSCG00000039506		217.95	1.87	0.15	12.29	0.00	0.00
ENSSSCG00000011119	ECHDC3	377.39	1.87	0.11	16.29	0.00	0.00
ENSSSCG00000032228	C8orf89	14.22	1.87	0.56	3.34	0.00	0.00
ENSSSCG00000010861	COQ8A	40.17	1.87	0.34	5.54	0.00	0.00
ENSSSCG00000017798	TMIGD1	7.88	1.87	0.76	2.46	0.01	0.02

ENSSSCG00000013715		343.87	1.87	0.12	15.64	0.00	0.00
ENSSSCG00000035859	WNT5A	154.60	1.88	0.18	10.43	0.00	0.00
ENSSSCG00000029151	RASIP1	130.99	1.88	0.19	10.00	0.00	0.00
ENSSSCG00000012050	RCAN1	727.02	1.88	0.09	21.02	0.00	0.00
ENSSSCG00000014967	FAM76B	780.72	1.88	0.09	22.05	0.00	0.00
ENSSSCG00000023156		573.97	1.88	0.09	19.96	0.00	0.00
ENSSSCG00000008123	ARID5A	112.25	1.88	0.20	9.28	0.00	0.00
ENSSSCG00000025717	HIC1	110.83	1.88	0.20	9.37	0.00	0.00
ENSSSCG00000030271	GSTO2	13.46	1.88	0.57	3.32	0.00	0.00
ENSSSCG00000001762		95.13	1.88	0.22	8.69	0.00	0.00
ENSSSCG00000008413	GPR75	21.40	1.88	0.45	4.21	0.00	0.00
ENSSSCG00000016825	TTC23L	21.45	1.88	0.45	4.15	0.00	0.00
ENSSSCG00000017371	TMEM101	395.22	1.89	0.11	16.78	0.00	0.00
ENSSSCG00000038460	FOXF1	154.24	1.89	0.18	10.42	0.00	0.00
ENSSSCG00000034332		23.07	1.89	0.44	4.30	0.00	0.00
ENSSSCG00000014581	TUB	24.64	1.89	0.42	4.46	0.00	0.00
ENSSSCG00000002037	CDH24	763.14	1.89	0.08	23.01	0.00	0.00
ENSSSCG00000037334	ADSSL1	434.64	1.89	0.11	17.15	0.00	0.00
ENSSSCG00000017120	SLC6A19	4.77	1.89	0.96	1.96	0.05	0.07
ENSSSCG00000004144	HECA	71.76	1.89	0.26	7.29	0.00	0.00
ENSSSCG00000035181	RNF24	96.55	1.89	0.22	8.51	0.00	0.00
ENSSSCG00000032156		4.79	1.89	0.95	2.00	0.05	0.06
ENSSSCG00000038357		5.59	1.90	0.90	2.10	0.04	0.05
ENSSSCG00000004729		50.27	1.90	0.29	6.48	0.00	0.00
ENSSSCG00000008607		4.80	1.90	0.94	2.02	0.04	0.06

ENSSSCG00000024759	CX3CL1	182.08	1.90	0.17	10.85	0.00	0.00
ENSSSCG00000013270	CRY2	613.19	1.90	0.09	21.21	0.00	0.00
ENSSSCG00000000026	SULT4A1	4.81	1.90	0.96	1.98	0.05	0.06
ENSSSCG00000004703		641.64	1.90	0.09	21.77	0.00	0.00
ENSSSCG00000005857	CYSRT1	34.46	1.90	0.36	5.29	0.00	0.00
ENSSSCG00000014957	C11orf97	398.53	1.90	0.11	17.40	0.00	0.00
ENSSSCG00000035071	DCST1	13.61	1.91	0.57	3.33	0.00	0.00
ENSSSCG00000007717	METTL27	189.44	1.91	0.16	12.17	0.00	0.00
ENSSSCG00000035833	NKPD1	7.24	1.91	0.77	2.47	0.01	0.02
ENSSSCG00000009822	FAM216A	195.40	1.91	0.15	12.46	0.00	0.00
ENSSSCG00000008829	OCIAD2	169.69	1.91	0.17	11.19	0.00	0.00
ENSSSCG00000007285	GGT7	566.22	1.91	0.10	19.92	0.00	0.00
ENSSSCG00000000943	OTOGL	7.22	1.91	0.80	2.40	0.02	0.02
ENSSSCG00000024596	NOCT	676.84	1.91	0.10	18.72	0.00	0.00
ENSSSCG00000032254	ssc-mir-7144	14.51	1.91	0.55	3.49	0.00	0.00
ENSSSCG00000004464	TENT5A	1505.09	1.91	0.07	28.08	0.00	0.00
ENSSSCG00000026605	BPI	6.46	1.91	0.84	2.29	0.02	0.03
ENSSSCG00000033386		16.14	1.92	0.51	3.72	0.00	0.00
ENSSSCG00000039990		9.67	1.92	0.72	2.66	0.01	0.01
ENSSSCG00000022175		9.68	1.92	0.67	2.86	0.00	0.01
ENSSSCG00000016200	PRKAG3	263.10	1.92	0.14	13.89	0.00	0.00
ENSSSCG00000003256	PRKCG	115.45	1.92	0.20	9.73	0.00	0.00
ENSSSCG00000001498	BEND6	72.62	1.92	0.25	7.68	0.00	0.00
ENSSSCG00000005511	TRAF1	41.15	1.92	0.33	5.74	0.00	0.00
ENSSSCG00000040883	TMEM199	1321.91	1.92	0.07	27.49	0.00	0.00

ENSSSCG00000003069	KCNN4	16.22	1.92	0.53	3.61	0.00	0.00
ENSSSCG00000036639	STOM	4302.20	1.93	0.04	43.24	0.00	0.00
ENSSSCG00000027197	DYRK1B	134.65	1.93	0.19	10.18	0.00	0.00
ENSSSCG00000009658	BNIP3L	1447.37	1.93	0.08	24.57	0.00	0.00
ENSSSCG00000009827	HVCN1	138.66	1.93	0.19	9.99	0.00	0.00
ENSSSCG00000008034	NOXO1	26.76	1.93	0.42	4.56	0.00	0.00
ENSSSCG00000014015	MRNIP	189.38	1.93	0.16	12.36	0.00	0.00
ENSSSCG00000024221	SMUG1	392.43	1.93	0.11	17.13	0.00	0.00
ENSSSCG00000003140	FGF21	25.20	1.93	0.44	4.37	0.00	0.00
ENSSSCG00000034345	SPRY3	19.51	1.93	0.47	4.09	0.00	0.00
ENSSSCG00000000692	PIANP	30.99	1.93	0.39	4.97	0.00	0.00
ENSSSCG00000024067	KRT23	381.67	1.93	0.12	16.80	0.00	0.00
ENSSSCG00000013260		1972.88	1.93	0.06	33.73	0.00	0.00
ENSSSCG00000028682		483.26	1.93	0.10	19.29	0.00	0.00
ENSSSCG00000010922	ELF3	1515.22	1.94	0.07	29.47	0.00	0.00
ENSSSCG00000024867	ISG20	172.84	1.94	0.16	11.80	0.00	0.00
ENSSSCG00000015393	DMTF1	1138.54	1.94	0.08	25.30	0.00	0.00
ENSSSCG00000029838	FZD2	41.61	1.94	0.32	5.99	0.00	0.00
ENSSSCG00000032709	ARL4A	347.07	1.94	0.12	16.42	0.00	0.00
ENSSSCG00000008584		152.82	1.94	0.18	10.94	0.00	0.00
ENSSSCG00000034474		148.74	1.94	0.18	10.75	0.00	0.00
ENSSSCG00000026724	PLBD1	11.45	1.94	0.68	2.87	0.00	0.01
ENSSSCG00000011353	PFKFB4	144.14	1.94	0.18	10.77	0.00	0.00
ENSSSCG00000013258	HARBI1	113.18	1.94	0.20	9.63	0.00	0.00
ENSSSCG00000021606	FLVCR2	11.46	1.95	0.62	3.14	0.00	0.00

ENSSSCG00000003266	TMC4	14.77	1.95	0.55	3.55	0.00	0.00
ENSSSCG00000031154	ACKR2	5.76	1.95	0.87	2.24	0.02	0.03
ENSSSCG00000010106		5.76	1.95	0.86	2.27	0.02	0.03
ENSSSCG00000034471	PSORS1C2	15.62	1.95	0.53	3.68	0.00	0.00
ENSSSCG00000017998	GLP2R	5.77	1.95	0.87	2.25	0.02	0.03
ENSSSCG00000033739		8.23	1.95	0.74	2.65	0.01	0.01
ENSSSCG00000013620	PLPPR2	172.20	1.95	0.17	11.70	0.00	0.00
ENSSSCG00000004573		4.95	1.95	0.94	2.08	0.04	0.05
ENSSSCG00000030076	SLC6A13	12.37	1.96	0.60	3.27	0.00	0.00
ENSSSCG00000006764	PTPN22	125.45	1.96	0.20	9.57	0.00	0.00
ENSSSCG00000038693	RAB19	125.36	1.96	0.19	10.29	0.00	0.00
ENSSSCG00000026067	TMPRSS6	5.78	1.96	0.91	2.14	0.03	0.04
ENSSSCG00000028995	TLE2	220.44	1.96	0.14	13.58	0.00	0.00
ENSSSCG00000002906	ETV2	21.53	1.96	0.45	4.35	0.00	0.00
ENSSSCG00000006477	BCAN	49.81	1.96	0.32	6.19	0.00	0.00
ENSSSCG00000007094	DZANK1	863.36	1.96	0.08	24.91	0.00	0.00
ENSSSCG00000005452	C9orf152	99.53	1.97	0.21	9.26	0.00	0.00
ENSSSCG00000026583	TLR1	37.39	1.97	0.35	5.57	0.00	0.00
ENSSSCG00000008899		101.25	1.97	0.21	9.31	0.00	0.00
ENSSSCG00000000523	BBS10	83.88	1.97	0.23	8.43	0.00	0.00
ENSSSCG00000010926	SYT2	84.83	1.97	0.23	8.49	0.00	0.00
ENSSSCG00000038148	PROKR1	13.27	1.97	0.59	3.34	0.00	0.00
ENSSSCG00000026689		273.11	1.97	0.14	14.39	0.00	0.00
ENSSSCG00000021092	FAM53C	2068.08	1.97	0.06	33.87	0.00	0.00
ENSSSCG00000027196		10.02	1.97	0.70	2.81	0.00	0.01

ENSSSCG00000007369		10.82	1.97	0.64	3.10	0.00	0.00
ENSSSCG00000022721	CDH16	206.64	1.97	0.15	13.28	0.00	0.00
ENSSSCG00000000371	PMEL	201.89	1.98	0.15	13.11	0.00	0.00
ENSSSCG00000005216		20.86	1.98	0.46	4.33	0.00	0.00
ENSSSCG00000013758	ZSWIM4	410.66	1.98	0.12	16.38	0.00	0.00
ENSSSCG00000002444	FBLN5	20.88	1.98	0.46	4.34	0.00	0.00
ENSSSCG00000006579	S100A3	299.22	1.98	0.13	15.55	0.00	0.00
ENSSSCG00000023732	KLHDC7A	103.76	1.98	0.21	9.30	0.00	0.00
ENSSSCG00000011876	DTX3L	381.65	1.98	0.11	17.33	0.00	0.00
ENSSSCG00000038945		31.79	1.98	0.38	5.18	0.00	0.00
ENSSSCG00000020876	RTL5	133.12	1.98	0.18	10.79	0.00	0.00
ENSSSCG00000035673	HBP1	1359.27	1.98	0.07	28.46	0.00	0.00
ENSSSCG00000022292	NPL	46.08	1.99	0.31	6.32	0.00	0.00
ENSSSCG00000037577	CHAC2	37.69	1.99	0.35	5.70	0.00	0.00
ENSSSCG00000000483		367.33	1.99	0.11	17.40	0.00	0.00
ENSSSCG00000032887	EFCAB1	7.51	1.99	0.81	2.45	0.01	0.02
ENSSSCG00000030262		14.22	1.99	0.61	3.23	0.00	0.00
ENSSSCG00000000091	APOBEC3B	272.00	1.99	0.13	15.24	0.00	0.00
ENSSSCG00000031856	DACT1	102.34	1.99	0.22	8.95	0.00	0.00
ENSSSCG00000007703	TRIM50	55.40	1.99	0.30	6.72	0.00	0.00
ENSSSCG00000035284	BMF	270.46	1.99	0.13	14.88	0.00	0.00
ENSSSCG00000010687	INPP5F	472.29	1.99	0.10	19.56	0.00	0.00
ENSSSCG00000033358	VPS37D	44.57	1.99	0.33	6.02	0.00	0.00
ENSSSCG00000037955		13.46	1.99	0.57	3.48	0.00	0.00
ENSSSCG00000010444	LIPM	65.70	1.99	0.27	7.44	0.00	0.00

ENSSSCG00000001411	APOM	7.60	2.00	0.76	2.62	0.01	0.01
ENSSSCG00000034753	FAM177A1	402.91	2.00	0.12	17.04	0.00	0.00
ENSSSCG00000036363		236.35	2.00	0.14	13.78	0.00	0.00
ENSSSCG00000031110	TMEM150B	60.82	2.00	0.27	7.31	0.00	0.00
ENSSSCG00000001470	SLA-DMA	28.73	2.00	0.40	5.04	0.00	0.00
ENSSSCG00000023165	SEMA7A	147.02	2.00	0.18	10.96	0.00	0.00
ENSSSCG00000017511	PLXDC1	12.68	2.00	0.59	3.40	0.00	0.00
ENSSSCG00000017901	INCA1	17.77	2.00	0.50	4.03	0.00	0.00
ENSSSCG00000006640	BNIPL	29.64	2.00	0.39	5.10	0.00	0.00
ENSSSCG00000000703	VAMP1	15.24	2.01	0.56	3.57	0.00	0.00
ENSSSCG00000035014		14.44	2.01	0.56	3.57	0.00	0.00
ENSSSCG00000009730	ZNF26	9.33	2.01	0.69	2.92	0.00	0.01
ENSSSCG00000006458	FCRL4	5.11	2.01	0.92	2.18	0.03	0.04
ENSSSCG00000029694	SLC23A3	4.26	2.01	1.02	1.98	0.05	0.06
ENSSSCG00000014034	N4BP3	615.17	2.02	0.09	21.71	0.00	0.00
ENSSSCG00000023991	RIT1	1292.37	2.02	0.07	29.08	0.00	0.00
ENSSSCG00000028056	ZFP36	1215.12	2.02	0.07	27.29	0.00	0.00
ENSSSCG00000027091	OAZ3	19.64	2.02	0.48	4.21	0.00	0.00
ENSSSCG00000025836	SULT1C4	72.59	2.02	0.25	8.07	0.00	0.00
ENSSSCG00000003693	MYOM1	8.53	2.02	0.72	2.81	0.00	0.01
ENSSSCG00000030247	EPM2AIP1	919.09	2.02	0.08	25.48	0.00	0.00
ENSSSCG00000035955		7.68	2.02	0.83	2.45	0.01	0.02
ENSSSCG00000017490	GSDMA	7.69	2.02	0.76	2.65	0.01	0.01
ENSSSCG00000024666		16.23	2.02	0.53	3.84	0.00	0.00
ENSSSCG00000007897	SNN	25.71	2.03	0.42	4.81	0.00	0.00

ENSSSCG00000010338	DYDC1	111.54	2.03	0.20	10.07	0.00	0.00
ENSSSCG00000021041		352.27	2.03	0.12	17.07	0.00	0.00
ENSSSCG00000014277	IRF1	867.67	2.03	0.08	24.70	0.00	0.00
ENSSSCG00000004675	DUOX1	6.89	2.03	0.81	2.52	0.01	0.02
ENSSSCG00000005657	PKN3	76.54	2.03	0.25	8.18	0.00	0.00
ENSSSCG00000013664	C19orf66	55.05	2.03	0.30	6.83	0.00	0.00
ENSSSCG00000005272	NMRK1	22.36	2.03	0.45	4.48	0.00	0.00
ENSSSCG00000023585	SERINC2	474.64	2.04	0.10	19.69	0.00	0.00
ENSSSCG00000017359	RUNDC3A	129.28	2.04	0.19	10.46	0.00	0.00
ENSSSCG00000036534	MYOM3	95.58	2.04	0.23	8.67	0.00	0.00
ENSSSCG00000007800	SEPT1	9.48	2.04	0.70	2.93	0.00	0.01
ENSSSCG00000038167		6.91	2.04	0.80	2.55	0.01	0.02
ENSSSCG00000020809	B3GAT3	956.48	2.04	0.08	26.65	0.00	0.00
ENSSSCG00000023975	CCDC9B	749.65	2.04	0.08	24.13	0.00	0.00
ENSSSCG00000025523	COL2A1	6.93	2.04	0.80	2.56	0.01	0.02
ENSSSCG00000002309	PLEKHD1	15.58	2.04	0.54	3.80	0.00	0.00
ENSSSCG00000007451	SLC2A10	71.92	2.04	0.25	8.04	0.00	0.00
ENSSSCG00000030211	NBR1	3031.66	2.04	0.05	40.73	0.00	0.00
ENSSSCG00000026417	EPS8L1	31.19	2.05	0.38	5.42	0.00	0.00
ENSSSCG00000025667	FBXO2	725.80	2.05	0.08	24.36	0.00	0.00
ENSSSCG00000024970	ETV3	2363.94	2.05	0.06	36.15	0.00	0.00
ENSSSCG00000037029	CSRNP2	471.32	2.05	0.11	19.51	0.00	0.00
ENSSSCG00000032301		508.58	2.05	0.24	8.70	0.00	0.00
ENSSSCG00000013361	SPTY2D1	2494.71	2.05	0.05	37.35	0.00	0.00
ENSSSCG00000016187	CATIP	161.78	2.05	0.17	12.06	0.00	0.00

ENSSSCG00000039433		8.74	2.05	0.76	2.70	0.01	0.01
ENSSSCG00000025924	IGFBP5	64.35	2.05	0.27	7.48	0.00	0.00
ENSSSCG00000028076	ZBTB7C	22.70	2.06	0.48	4.31	0.00	0.00
ENSSSCG00000015871	NR4A2	250.53	2.06	0.14	14.63	0.00	0.00
ENSSSCG00000033479	NEUROG2	34.05	2.06	0.36	5.65	0.00	0.00
ENSSSCG00000017963	DNAH2	273.23	2.06	0.13	15.32	0.00	0.00
ENSSSCG00000009565	GAS6	20.93	2.06	0.47	4.35	0.00	0.00
ENSSSCG00000011271	ZNF619	12.24	2.06	0.67	3.09	0.00	0.00
ENSSSCG00000022128	MXI1	151.70	2.07	0.19	10.91	0.00	0.00
ENSSSCG00000029305	FNDC4	10.53	2.07	0.65	3.20	0.00	0.00
ENSSSCG00000006625	RFX5	1250.33	2.07	0.07	29.16	0.00	0.00
ENSSSCG00000036469		5.26	2.07	0.92	2.26	0.02	0.03
ENSSSCG00000008491	QPCT	925.43	2.07	0.08	27.03	0.00	0.00
ENSSSCG00000031661	C12orf75	181.35	2.07	0.16	12.92	0.00	0.00
ENSSSCG00000025353	TNNT1	369.58	2.07	0.12	17.62	0.00	0.00
ENSSSCG00000006400	SLAMF9	5.30	2.07	0.95	2.18	0.03	0.04
ENSSSCG00000034418	CREBL2	401.35	2.08	0.12	17.03	0.00	0.00
ENSSSCG00000014964		4.40	2.08	1.01	2.06	0.04	0.05
ENSSSCG00000038419		114.01	2.08	0.21	9.94	0.00	0.00
ENSSSCG00000037569		4.40	2.08	1.04	2.01	0.04	0.06
ENSSSCG00000029460	RND2	29.14	2.08	0.40	5.17	0.00	0.00
ENSSSCG00000017923	ALOX15	9.72	2.08	0.69	3.04	0.00	0.00
ENSSSCG00000008684	POLN	53.16	2.08	0.30	7.05	0.00	0.00
ENSSSCG00000027045	LRRC56	119.65	2.08	0.20	10.31	0.00	0.00
ENSSSCG00000017943	ACAP1	1753.80	2.08	0.15	13.80	0.00	0.00

ENSSSCG00000017277	PECAM1	45.18	2.09	0.32	6.61	0.00	0.00
ENSSSCG00000023848	VXN	4.43	2.09	1.02	2.05	0.04	0.05
ENSSSCG00000013455	IZUMO4	15.11	2.09	0.55	3.81	0.00	0.00
ENSSSCG00000003662	NT5C1A	4.45	2.09	1.00	2.10	0.04	0.05
ENSSSCG00000002811		10.70	2.09	0.68	3.08	0.00	0.00
ENSSSCG00000001789	TMC3	6.24	2.09	0.88	2.37	0.02	0.03
ENSSSCG00000025393	SUSD2	298.92	2.09	0.13	16.06	0.00	0.00
ENSSSCG00000012544		8.90	2.09	0.73	2.87	0.00	0.01
ENSSSCG00000001565	CDKN1A	687.60	2.09	0.09	22.93	0.00	0.00
ENSSSCG00000036265		12.45	2.10	0.60	3.48	0.00	0.00
ENSSSCG00000037951	ZNF891	24.08	2.10	0.44	4.77	0.00	0.00
ENSSSCG00000028460	S1PR5	57.93	2.10	0.29	7.20	0.00	0.00
ENSSSCG00000037001	PPM1N	16.94	2.10	0.54	3.89	0.00	0.00
ENSSSCG00000006495	SEMA4A	15.16	2.10	0.55	3.84	0.00	0.00
ENSSSCG00000013506	STAP2	48.19	2.10	0.31	6.85	0.00	0.00
ENSSSCG00000015035	C11orf52	19.64	2.10	0.50	4.17	0.00	0.00
ENSSSCG00000018061		3575.71	2.10	0.13	15.94	0.00	0.00
ENSSSCG00000032509	LRRC26	8.07	2.10	0.74	2.84	0.00	0.01
ENSSSCG00000033059	CASKIN1	81.63	2.11	0.24	8.74	0.00	0.00
ENSSSCG00000025529	FAM3D	15.28	2.11	0.56	3.77	0.00	0.00
ENSSSCG00000034570	IFI6	35.82	2.11	0.37	5.69	0.00	0.00
ENSSSCG00000040497	RF00026	8.08	2.11	0.75	2.82	0.00	0.01
ENSSSCG00000020838		80.87	2.11	0.24	8.87	0.00	0.00
ENSSSCG00000017522	SP6	246.39	2.11	0.14	15.12	0.00	0.00
ENSSSCG00000011439	PHF7	55.80	2.11	0.29	7.38	0.00	0.00

ENSSSCG00000003170	SLC17A7	9.92	2.11	0.69	3.08	0.00	0.00
ENSSSCG00000029796	KBTBD11	18.88	2.11	0.49	4.32	0.00	0.00
ENSSSCG00000038186	TIGD4	191.72	2.11	0.16	13.49	0.00	0.00
ENSSSCG00000034858	RAP1GAP2	7.19	2.11	0.80	2.66	0.01	0.01
ENSSSCG00000027198	PDK2	432.13	2.12	0.11	19.33	0.00	0.00
ENSSSCG00000033652		9.01	2.12	0.71	3.00	0.00	0.00
ENSSSCG00000032966		7.24	2.12	0.80	2.63	0.01	0.01
ENSSSCG00000016217	DNAJB2	701.81	2.12	0.09	24.42	0.00	0.00
ENSSSCG00000006935	CLCA2	14.50	2.12	0.57	3.71	0.00	0.00
ENSSSCG00000038979	ARHGDIG	10.87	2.13	0.66	3.22	0.00	0.00
ENSSSCG00000025618	TAP1	1183.80	2.13	0.07	30.88	0.00	0.00
ENSSSCG00000012959	CATSPER1	19.99	2.13	0.48	4.43	0.00	0.00
ENSSSCG00000009366	STOML3	127.46	2.13	0.20	10.92	0.00	0.00
ENSSSCG00000034181	NKX3-2	19.10	2.13	0.51	4.21	0.00	0.00
ENSSSCG00000038739	MYPOP	33.71	2.13	0.37	5.83	0.00	0.00
ENSSSCG00000028372	FAM131A	66.51	2.13	0.26	8.11	0.00	0.00
ENSSSCG00000027434		8.21	2.13	0.75	2.84	0.00	0.01
ENSSSCG00000002866	CEBPA	15.50	2.14	0.55	3.86	0.00	0.00
ENSSSCG00000006309	CD247	23.77	2.14	0.44	4.83	0.00	0.00
ENSSSCG00000014803	LRRC51	76.67	2.14	0.25	8.50	0.00	0.00
ENSSSCG00000033726		10.06	2.14	0.68	3.15	0.00	0.00
ENSSSCG00000001469	SLA-DMB	41.18	2.14	0.34	6.38	0.00	0.00
ENSSSCG00000008631	PQLC3	4.58	2.14	1.01	2.12	0.03	0.05
ENSSSCG00000013740	SYCE2	77.73	2.14	0.25	8.62	0.00	0.00
ENSSSCG00000003024	PRR19	24.71	2.14	0.44	4.88	0.00	0.00

ENSSSCG00000038527		20.21	2.14	0.50	4.33	0.00	0.00
ENSSSCG00000011538	LMCD1	14.64	2.14	0.57	3.78	0.00	0.00
ENSSSCG00000012520		6.45	2.14	0.90	2.39	0.02	0.02
ENSSSCG00000014335	REEP2	469.29	2.15	0.10	20.88	0.00	0.00
ENSSSCG00000017282	SCN4A	23.85	2.15	0.45	4.82	0.00	0.00
ENSSSCG00000006678	FCGR1A	9.17	2.15	0.71	3.02	0.00	0.00
ENSSSCG00000017792	CORO6	536.38	2.15	0.10	21.69	0.00	0.00
ENSSSCG00000026333	GTSF1	4.60	2.15	0.98	2.19	0.03	0.04
ENSSSCG00000002013	DHRS4	7.35	2.15	0.79	2.72	0.01	0.01
ENSSSCG00000008001		162.01	2.15	0.17	12.35	0.00	0.00
ENSSSCG00000011951	NFKBIZ	1438.96	2.16	0.07	28.81	0.00	0.00
ENSSSCG00000033762	NATD1	22.25	2.16	0.46	4.73	0.00	0.00
ENSSSCG00000006183	SBSPON	7.42	2.16	0.78	2.76	0.01	0.01
ENSSSCG00000040504		4.63	2.16	1.02	2.13	0.03	0.05
ENSSSCG00000011587	EFCAB12	183.24	2.16	0.16	13.17	0.00	0.00
ENSSSCG00000039272	IP6K3	35.21	2.16	0.37	5.88	0.00	0.00
ENSSSCG00000014997		342.58	2.17	0.12	17.41	0.00	0.00
ENSSSCG00000027357	CSTB	1128.97	2.17	0.07	29.80	0.00	0.00
ENSSSCG00000039626	DERL3	4.66	2.17	1.06	2.06	0.04	0.05
ENSSSCG00000023294	KRCC1	118.15	2.17	0.20	10.84	0.00	0.00
ENSSSCG00000037791	NKD2	89.37	2.17	0.23	9.54	0.00	0.00
ENSSSCG00000028492	C4orf46	205.83	2.17	0.15	14.34	0.00	0.00
ENSSSCG00000001207	PGBD1	131.41	2.17	0.19	11.25	0.00	0.00
ENSSSCG00000021911	NDRG4	899.33	2.17	0.08	28.00	0.00	0.00
ENSSSCG00000016435	SMARCD3	198.53	2.17	0.16	13.70	0.00	0.00

ENSSSCG00000011400	SEMA3B	391.00	2.18	0.11	19.05	0.00	0.00
ENSSSCG00000008054	NTN3	17.70	2.18	0.52	4.18	0.00	0.00
ENSSSCG00000011559	IL17RC	117.71	2.18	0.21	10.26	0.00	0.00
ENSSSCG00000040260		186.01	2.18	0.16	13.62	0.00	0.00
ENSSSCG00000012517		331.87	2.18	0.12	17.62	0.00	0.00
ENSSSCG00000001040	C6orf52	11.25	2.18	0.66	3.31	0.00	0.00
ENSSSCG00000030921	APOA1	1511.82	2.18	0.06	34.59	0.00	0.00
ENSSSCG00000038184	ACBD7	18.71	2.18	0.50	4.41	0.00	0.00
ENSSSCG00000006834	MYBPHL	12.25	2.18	0.68	3.23	0.00	0.00
ENSSSCG00000034102	DGAT2	29.05	2.18	0.40	5.52	0.00	0.00
ENSSSCG00000003590	PTPRU	49.78	2.19	0.33	6.67	0.00	0.00
ENSSSCG00000039947	KCNJ2	396.79	2.19	0.13	16.84	0.00	0.00
ENSSSCG00000007171	EBF4	97.76	2.19	0.22	9.81	0.00	0.00
ENSSSCG00000000062	CSDC2	197.51	2.19	0.16	13.93	0.00	0.00
ENSSSCG00000000117	C22orf23	60.14	2.19	0.28	7.74	0.00	0.00
ENSSSCG00000006542	KCNN3	7.51	2.19	0.79	2.77	0.01	0.01
ENSSSCG00000035104	TERB1	15.04	2.19	0.60	3.67	0.00	0.00
ENSSSCG00000002980		18.86	2.19	0.51	4.28	0.00	0.00
ENSSSCG00000001439	GPSM3	3.77	2.20	1.10	2.00	0.05	0.06
ENSSSCG00000006587	PGLYRP4	24.53	2.20	0.44	4.96	0.00	0.00
ENSSSCG00000014812	FOLR2	71.73	2.20	0.26	8.54	0.00	0.00
ENSSSCG00000006023	SYBU	10.44	2.20	0.68	3.23	0.00	0.00
ENSSSCG00000010581	PSD	31.28	2.20	0.39	5.65	0.00	0.00
ENSSSCG00000017321	LYZL6	4.74	2.20	1.01	2.19	0.03	0.04
ENSSSCG00000001846	WDR93	16.11	2.21	0.54	4.09	0.00	0.00

ENSSSCG00000003278		48.45	2.21	0.32	6.89	0.00	0.00
ENSSSCG00000006082	MATN2	49.33	2.21	0.31	7.03	0.00	0.00
ENSSSCG00000017954	SOX15	16.14	2.21	0.54	4.11	0.00	0.00
ENSSSCG00000006153	FABP5	4.75	2.21	0.98	2.26	0.02	0.03
ENSSSCG00000016110	KIAA2012	55.09	2.21	0.29	7.50	0.00	0.00
ENSSSCG00000014157	NR2F1	69.42	2.21	0.27	8.22	0.00	0.00
ENSSSCG00000003232	LIM2	4.74	2.21	0.99	2.24	0.03	0.03
ENSSSCG00000003410	MASP2	15.22	2.21	0.56	3.96	0.00	0.00
ENSSSCG00000010096	AIFM3	9.52	2.22	0.72	3.08	0.00	0.00
ENSSSCG00000031759	TM6SF2	9.56	2.22	0.70	3.19	0.00	0.00
ENSSSCG00000017244		15.28	2.22	0.55	4.06	0.00	0.00
ENSSSCG00000033637		40.18	2.22	0.34	6.52	0.00	0.00
ENSSSCG00000009012	TMEM154	45.97	2.22	0.33	6.80	0.00	0.00
ENSSSCG00000027206	PARD6B	2246.62	2.23	0.06	36.49	0.00	0.00
ENSSSCG00000031877	ABHD8	199.54	2.23	0.15	14.38	0.00	0.00
ENSSSCG00000033190		89.26	2.23	0.23	9.61	0.00	0.00
ENSSSCG00000022741	PDGFRB	54.68	2.23	0.30	7.47	0.00	0.00
ENSSSCG00000001657	CUL7	110.48	2.23	0.21	10.52	0.00	0.00
ENSSSCG00000000092	NPTXR	130.73	2.23	0.19	11.51	0.00	0.00
ENSSSCG00000021880	MXRA8	186.46	2.23	0.16	13.72	0.00	0.00
ENSSSCG00000033819		25.01	2.23	0.44	5.11	0.00	0.00
ENSSSCG00000031799	TMEM139	92.42	2.23	0.23	9.57	0.00	0.00
ENSSSCG00000040062	KRTCAP3	6.75	2.23	0.85	2.63	0.01	0.01
ENSSSCG00000013378	ABCC8	37.58	2.23	0.35	6.30	0.00	0.00
ENSSSCG00000007816	IL21R	21.23	2.23	0.47	4.75	0.00	0.00

ENSSSCG00000036586		4.85	2.24	1.07	2.10	0.04	0.05
ENSSSCG00000017585	SAMD14	18.32	2.24	0.53	4.24	0.00	0.00
ENSSSCG00000002639		634.37	2.24	0.10	22.93	0.00	0.00
ENSSSCG00000001440	NOTCH4	27.04	2.24	0.42	5.29	0.00	0.00
ENSSSCG00000007967	ZNF213	143.14	2.24	0.19	12.03	0.00	0.00
ENSSSCG00000012773	PNCK	113.29	2.24	0.21	10.51	0.00	0.00
ENSSSCG00000011047	FAM171A1	6.79	2.24	0.85	2.65	0.01	0.01
ENSSSCG00000038491	MEX3B	135.81	2.24	0.19	11.72	0.00	0.00
ENSSSCG00000040182	RHBDD2	1531.12	2.25	0.06	34.81	0.00	0.00
ENSSSCG00000034113		9.69	2.25	0.70	3.20	0.00	0.00
ENSSSCG00000005097	SNAPC1	358.50	2.25	0.12	18.26	0.00	0.00
ENSSSCG00000024604	ATAT1	97.10	2.25	0.22	10.17	0.00	0.00
ENSSSCG00000015098	CXCR5	13.58	2.25	0.59	3.78	0.00	0.00
ENSSSCG00000003142	IZUMO1	64.25	2.25	0.28	8.14	0.00	0.00
ENSSSCG00000032687	CYP4V2	368.74	2.25	0.12	18.63	0.00	0.00
ENSSSCG00000037600	C1orf216	19.49	2.25	0.49	4.59	0.00	0.00
ENSSSCG00000038283	KLK11	27.29	2.25	0.42	5.35	0.00	0.00
ENSSSCG00000024388	BNIP3	1773.66	2.26	0.16	14.31	0.00	0.00
ENSSSCG00000005707	FIBCD1	22.44	2.26	0.46	4.94	0.00	0.00
ENSSSCG00000034449	RSRP1	1499.33	2.26	0.07	31.21	0.00	0.00
ENSSSCG00000016416	SHH	45.94	2.26	0.32	7.04	0.00	0.00
ENSSSCG00000029412	RF00319	24.47	2.26	0.44	5.10	0.00	0.00
ENSSSCG00000023280	SULT2B1	22.46	2.26	0.46	4.86	0.00	0.00
ENSSSCG00000036076	SAPCD1	5.87	2.26	0.99	2.28	0.02	0.03
ENSSSCG00000002962	MAP4K1	134.54	2.26	0.20	11.55	0.00	0.00

ENSSSCG00000014920	FZD4	3056.03	2.27	0.05	43.06	0.00	0.00
ENSSSCG00000029285		190.50	2.27	0.16	13.90	0.00	0.00
ENSSSCG00000020872		295.28	2.27	0.13	17.23	0.00	0.00
ENSSSCG00000009138		19.66	2.27	0.50	4.56	0.00	0.00
ENSSSCG00000011239	TRANK1	58.14	2.27	0.30	7.68	0.00	0.00
ENSSSCG00000003228	VSIG10L	10.84	2.27	0.69	3.28	0.00	0.00
ENSSSCG00000013643	CDKN2D	168.39	2.27	0.17	13.25	0.00	0.00
ENSSSCG00000038904		34.50	2.27	0.38	6.01	0.00	0.00
ENSSSCG00000000982	TTLL8	3.94	2.27	1.12	2.02	0.04	0.06
ENSSSCG00000017515	TBKBP1	29.57	2.27	0.41	5.54	0.00	0.00
ENSSSCG00000027529	BIRC3	6289.88	2.27	0.06	39.03	0.00	0.00
ENSSSCG00000037879		14.79	2.27	0.57	3.97	0.00	0.00
ENSSSCG00000011361	SLC26A6	471.74	2.27	0.11	21.16	0.00	0.00
ENSSSCG00000014952	IZUMO1R	15.80	2.28	0.54	4.18	0.00	0.00
ENSSSCG00000017006	LCP2	3.96	2.28	1.09	2.09	0.04	0.05
ENSSSCG00000011740	SERPINI1	151.18	2.28	0.18	12.77	0.00	0.00
ENSSSCG00000016437	WDR86	3.94	2.28	1.13	2.01	0.04	0.06
ENSSSCG00000027157	SLC40A1	54.50	2.28	0.30	7.72	0.00	0.00
ENSSSCG00000002478	SERPINA12	3.95	2.28	1.11	2.06	0.04	0.05
ENSSSCG00000013482	C19orf71	3.99	2.28	1.11	2.05	0.04	0.05
ENSSSCG00000006958	TOP1MT	5.96	2.28	0.90	2.53	0.01	0.02
ENSSSCG00000015436	CCDC71L	22.86	2.28	0.46	4.94	0.00	0.00
ENSSSCG00000017260	WIPI1	59.53	2.28	0.28	8.04	0.00	0.00
ENSSSCG00000004698	SERINC4	4.97	2.28	1.00	2.28	0.02	0.03
ENSSSCG00000037070	GRID2IP	25.81	2.29	0.45	5.11	0.00	0.00

ENSSSCG00000006774	PPM1J	114.37	2.29	0.21	10.71	0.00	0.00
ENSSSCG00000013011	SAC3D1	65.71	2.29	0.28	8.17	0.00	0.00
ENSSSCG00000024591	ETV1	4.01	2.29	1.15	2.00	0.05	0.06
ENSSSCG00000013879	SLC27A1	37.02	2.29	0.38	6.08	0.00	0.00
ENSSSCG00000035078	CD40	942.47	2.30	0.09	26.35	0.00	0.00
ENSSSCG00000016501	KDM7A	275.84	2.30	0.16	14.64	0.00	0.00
ENSSSCG00000034921	CLDN19	11.99	2.30	0.63	3.64	0.00	0.00
ENSSSCG00000021843	SYT5	63.98	2.30	0.28	8.35	0.00	0.00
ENSSSCG00000006497	MEX3A	14.04	2.30	0.60	3.85	0.00	0.00
ENSSSCG00000014091	F2RL1	635.75	2.30	0.10	23.09	0.00	0.00
ENSSSCG00000031219	NAPB	524.65	2.30	0.10	22.89	0.00	0.00
ENSSSCG00000013425	MISP	627.61	2.31	0.10	23.91	0.00	0.00
ENSSSCG00000006238	CYP7A1	9.08	2.31	0.72	3.19	0.00	0.00
ENSSSCG00000039526	HIST3H2BB	31.23	2.31	0.39	5.91	0.00	0.00
ENSSSCG00000033340		7.06	2.31	0.82	2.83	0.00	0.01
ENSSSCG00000008282	WDR54	470.17	2.32	0.11	20.91	0.00	0.00
ENSSSCG00000008867	CTSO	64.74	2.32	0.28	8.35	0.00	0.00
ENSSSCG00000034441	MRGPRF	6.07	2.32	0.91	2.54	0.01	0.02
ENSSSCG00000003157	LIN7B	46.55	2.32	0.32	7.17	0.00	0.00
ENSSSCG00000013385	INSC	11.15	2.32	0.67	3.48	0.00	0.00
ENSSSCG00000021342	QRICH2	20.25	2.32	0.49	4.69	0.00	0.00
ENSSSCG00000036437	NOG	5.05	2.32	0.99	2.34	0.02	0.03
ENSSSCG00000005586	LHX2	6.10	2.32	0.88	2.63	0.01	0.01
ENSSSCG00000008908	PDCL2	6.12	2.32	0.92	2.52	0.01	0.02
ENSSSCG00000017754		2760.96	2.32	0.07	34.59	0.00	0.00

ENSSSCG00000035506	UBAP1L	6.08	2.32	0.89	2.61	0.01	0.01
ENSSSCG00000005699	HMCN2	36.62	2.32	0.37	6.26	0.00	0.00
ENSSSCG00000011355		32.52	2.33	0.39	5.94	0.00	0.00
ENSSSCG00000032261	CCDC117	1846.12	2.33	0.06	35.85	0.00	0.00
ENSSSCG00000033453	BST2	608.34	2.33	0.10	23.70	0.00	0.00
ENSSSCG00000013092	PGA5	74.29	2.33	0.26	9.02	0.00	0.00
ENSSSCG00000007033	AP3M2	195.71	2.33	0.16	14.70	0.00	0.00
ENSSSCG00000002908	ZBTB32	34.70	2.33	0.38	6.18	0.00	0.00
ENSSSCG00000038857	TMIE	4.07	2.33	1.10	2.13	0.03	0.05
ENSSSCG00000039101	C1QL1	37.83	2.33	0.38	6.09	0.00	0.00
ENSSSCG00000040308		4.10	2.34	1.09	2.14	0.03	0.04
ENSSSCG00000036248		465.64	2.34	0.11	21.93	0.00	0.00
ENSSSCG00000017983		1256.82	2.34	0.07	32.16	0.00	0.00
ENSSSCG00000038954	PLIN3	649.01	2.34	0.09	25.11	0.00	0.00
ENSSSCG00000001848	MESP2	47.26	2.34	0.32	7.22	0.00	0.00
ENSSSCG00000012658	RAB33A	18.51	2.35	0.51	4.61	0.00	0.00
ENSSSCG00000029199	SCN4B	53.49	2.35	0.32	7.40	0.00	0.00
ENSSSCG00000040707		96.72	2.35	0.23	10.33	0.00	0.00
ENSSSCG00000027726	DLL1	4.12	2.35	1.09	2.15	0.03	0.04
ENSSSCG00000005472	SLC46A2	12.36	2.35	0.64	3.70	0.00	0.00
ENSSSCG00000003236		10.30	2.35	0.69	3.40	0.00	0.00
ENSSSCG00000023537	SYT8	15.50	2.35	0.57	4.15	0.00	0.00
ENSSSCG00000019602	RF00154	6.17	2.35	0.94	2.49	0.01	0.02
ENSSSCG00000022405	P2RX1	394.32	2.35	0.13	18.00	0.00	0.00
ENSSSCG00000021069		53.68	2.35	0.30	7.74	0.00	0.00

ENSSSCG00000033090		51.63	2.36	0.32	7.36	0.00	0.00
ENSSSCG00000017982	HES7	20.72	2.36	0.49	4.80	0.00	0.00
ENSSSCG00000014055	CDHR2	829.78	2.36	0.09	26.60	0.00	0.00
ENSSSCG00000034543	RF00026	6.20	2.36	0.89	2.66	0.01	0.01
ENSSSCG00000014278	IL5	42.55	2.36	0.35	6.81	0.00	0.00
ENSSSCG00000012789	AVPR2	13.50	2.36	0.61	3.87	0.00	0.00
ENSSSCG00000009612	DOK2	41.50	2.36	0.36	6.60	0.00	0.00
ENSSSCG00000029761		19.69	2.36	0.50	4.73	0.00	0.00
ENSSSCG00000015612	IRF6	1834.17	2.36	0.07	35.37	0.00	0.00
ENSSSCG00000010627	PDCD4	168.40	2.36	0.17	13.71	0.00	0.00
ENSSSCG00000038126	MGAT3	21.87	2.37	0.48	4.95	0.00	0.00
ENSSSCG00000008997	FGB	27.07	2.37	0.42	5.63	0.00	0.00
ENSSSCG00000036208	SHMT1	6.26	2.37	0.89	2.67	0.01	0.01
ENSSSCG00000038530	TMEM240	52.07	2.37	0.31	7.64	0.00	0.00
ENSSSCG00000040453		29.21	2.37	0.43	5.47	0.00	0.00
ENSSSCG00000039660		5.22	2.37	1.00	2.37	0.02	0.02
ENSSSCG00000000687	CD4	5.20	2.37	0.98	2.43	0.02	0.02
ENSSSCG00000002632	SLC28A1	5.22	2.37	0.96	2.46	0.01	0.02
ENSSSCG00000010009	GAL3ST1	74.08	2.37	0.26	9.04	0.00	0.00
ENSSSCG00000000148		237.17	2.37	0.15	15.86	0.00	0.00
ENSSSCG00000034198		105.78	2.38	0.22	11.04	0.00	0.00
ENSSSCG00000008124	NEURL3	67.99	2.38	0.29	8.18	0.00	0.00
ENSSSCG00000036892		42.86	2.38	0.36	6.61	0.00	0.00
ENSSSCG00000016578	FLNC	8.36	2.38	0.77	3.10	0.00	0.00
ENSSSCG00000017948	EFNB3	130.02	2.38	0.20	12.04	0.00	0.00

ENSSSCG00000033420	ZNF227	5.23	2.38	0.96	2.48	0.01	0.02
ENSSSCG00000033413	NRTN	12.59	2.38	0.63	3.80	0.00	0.00
ENSSSCG00000031526		23.10	2.38	0.47	5.08	0.00	0.00
ENSSSCG00000017131	FN3K	5.28	2.38	0.98	2.42	0.02	0.02
ENSSSCG00000037563	RF00002	96.63	2.38	0.23	10.56	0.00	0.00
ENSSSCG00000009239		331.96	2.38	0.13	19.03	0.00	0.00
ENSSSCG00000017994	CFAP52	136.77	2.39	0.19	12.45	0.00	0.00
ENSSSCG00000000645	GABARAPL1	579.08	2.39	0.11	21.63	0.00	0.00
ENSSSCG00000026454	PMAIP1	1011.18	2.39	0.08	29.11	0.00	0.00
ENSSSCG00000037432	PLPPR3	5.28	2.39	0.96	2.49	0.01	0.02
ENSSSCG00000028274	FAM131B	10.54	2.39	0.69	3.45	0.00	0.00
ENSSSCG00000006635	SEMA6C	27.47	2.39	0.42	5.65	0.00	0.00
ENSSSCG00000015595	ATF3	1321.42	2.39	0.07	34.84	0.00	0.00
ENSSSCG00000033790		12.70	2.39	0.62	3.86	0.00	0.00
ENSSSCG00000038269		77.29	2.40	0.62	3.88	0.00	0.00
ENSSSCG00000000894	CCDC38	9.54	2.40	0.71	3.37	0.00	0.00
ENSSSCG00000040690	LRRC61	111.33	2.40	0.21	11.37	0.00	0.00
ENSSSCG00000035176	SEMA6B	208.19	2.40	0.16	14.77	0.00	0.00
ENSSSCG00000015798	ANKRD37	215.15	2.40	0.18	13.04	0.00	0.00
ENSSSCG00000036804	TCEA2	179.74	2.40	0.17	14.16	0.00	0.00
ENSSSCG00000008686	C4orf48	45.76	2.41	0.33	7.22	0.00	0.00
ENSSSCG00000017186	RNF157	9.59	2.41	0.72	3.34	0.00	0.00
ENSSSCG00000013476	CELF5	4.27	2.41	1.11	2.18	0.03	0.04
ENSSSCG00000034942	STAR	17.09	2.41	0.54	4.48	0.00	0.00
ENSSSCG00000011246	VILL	787.52	2.42	0.09	28.35	0.00	0.00

ENSSSCG00000031899	JSRP1	67.53	2.42	0.28	8.70	0.00	0.00
ENSSSCG00000030303	ACHE	81.59	2.42	0.26	9.34	0.00	0.00
ENSSSCG00000012323	TSPYL2	453.64	2.43	0.11	21.59	0.00	0.00
ENSSSCG00000037674		26.97	2.43	0.46	5.23	0.00	0.00
ENSSSCG00000015412	CCDC146	5.38	2.43	0.96	2.53	0.01	0.02
ENSSSCG00000010832	HHIPL2	7.57	2.43	0.82	2.96	0.00	0.00
ENSSSCG00000030694	PKP1	10.83	2.44	0.67	3.63	0.00	0.00
ENSSSCG00000011324		22.82	2.44	0.47	5.23	0.00	0.00
ENSSSCG00000011519	GXYLT2	5.44	2.44	0.95	2.56	0.01	0.02
ENSSSCG00000022390	RGN	5.44	2.44	0.95	2.58	0.01	0.01
ENSSSCG00000010303	SYNPO2L	47.90	2.44	0.33	7.33	0.00	0.00
ENSSSCG00000033286		11.98	2.44	0.66	3.68	0.00	0.00
ENSSSCG00000037132	POU2F2	17.44	2.44	0.53	4.60	0.00	0.00
ENSSSCG00000015202	ROBO3	43.61	2.45	0.34	7.19	0.00	0.00
ENSSSCG00000021585	OXTR	15.25	2.45	0.57	4.27	0.00	0.00
ENSSSCG00000019512	RF00026	5.47	2.45	0.96	2.54	0.01	0.02
ENSSSCG00000007373	GDAP1L1	13.13	2.45	0.62	3.98	0.00	0.00
ENSSSCG00000029558	EXTL1	21.86	2.45	0.48	5.08	0.00	0.00
ENSSSCG00000031925	SHANK3	3.29	2.45	1.24	1.98	0.05	0.06
ENSSSCG00000017730	CDK5R1	130.03	2.45	0.21	11.87	0.00	0.00
ENSSSCG00000031959	CAPSL	48.15	2.45	0.32	7.65	0.00	0.00
ENSSSCG00000034472	RF00614	10.91	2.45	0.69	3.54	0.00	0.00
ENSSSCG00000024043	ADAMTS2	7.66	2.46	0.81	3.05	0.00	0.00
ENSSSCG00000000436	PIP4K2C	20.86	2.46	0.48	5.08	0.00	0.00
ENSSSCG00000020657	BCAM	1138.15	2.46	0.08	32.33	0.00	0.00

ENSSSCG00000001988	ADCY4	267.31	2.46	0.14	17.17	0.00	0.00
ENSSSCG00000017569	CHAD	3.31	2.46	1.21	2.03	0.04	0.06
ENSSSCG00000013296	SLC1A2	55.01	2.46	0.30	8.08	0.00	0.00
ENSSSCG00000038946	SLC17A8	4.41	2.47	1.06	2.34	0.02	0.03
ENSSSCG00000013313	PRRG4	954.16	2.47	0.09	28.52	0.00	0.00
ENSSSCG00000023441	KLHDC8A	25.38	2.47	0.46	5.34	0.00	0.00
ENSSSCG00000020705	MAP3K8	193.39	2.47	0.17	14.58	0.00	0.00
ENSSSCG00000012173	SAT1	3251.84	2.47	0.05	45.12	0.00	0.00
ENSSSCG00000015234	KIRREL3	12.17	2.47	0.64	3.87	0.00	0.00
ENSSSCG00000032428	ARSA	335.07	2.48	0.13	19.21	0.00	0.00
ENSSSCG00000005905	SLC39A4	31.11	2.48	0.41	6.02	0.00	0.00
ENSSSCG00000034013	TMEM88B	33.29	2.48	0.40	6.25	0.00	0.00
ENSSSCG00000029186	SEZ6L2	28.89	2.48	0.42	5.96	0.00	0.00
ENSSSCG00000035224		15.55	2.48	0.58	4.29	0.00	0.00
ENSSSCG00000024622	ZNF667	7.78	2.48	0.83	2.99	0.00	0.00
ENSSSCG00000024569	ANO9	134.64	2.48	0.20	12.44	0.00	0.00
ENSSSCG00000006372	ARHGAP30	132.49	2.48	0.21	11.68	0.00	0.00
ENSSSCG00000012733		10.04	2.48	0.72	3.45	0.00	0.00
ENSSSCG00000038289	ZNF234	10.01	2.48	0.72	3.46	0.00	0.00
ENSSSCG00000038073	NOD2	43.57	2.49	0.34	7.26	0.00	0.00
ENSSSCG00000033533		5.59	2.49	0.94	2.64	0.01	0.01
ENSSSCG00000027030	BDKRB2	53.66	2.49	0.31	8.15	0.00	0.00
ENSSSCG00000032601	RASSF2	25.81	2.49	0.44	5.67	0.00	0.00
ENSSSCG00000033015	B3GNT7	351.87	2.49	0.14	17.44	0.00	0.00
ENSSSCG00000032434	PLAUR	1551.62	2.50	0.07	34.93	0.00	0.00

ENSSSCG00000034261	TMEM59	2011.84	2.50	0.06	39.73	0.00	0.00
ENSSSCG00000037987		5.62	2.50	1.00	2.49	0.01	0.02
ENSSSCG00000017562	CACNA1G	63.09	2.50	0.30	8.39	0.00	0.00
ENSSSCG00000027894	FAM76A	765.41	2.50	0.10	26.13	0.00	0.00
ENSSSCG00000031977	PTCH2	10.16	2.50	0.72	3.49	0.00	0.00
ENSSSCG00000032197	GNG3	79.11	2.50	0.27	9.43	0.00	0.00
ENSSSCG00000008294	ACTG2	38.43	2.51	0.37	6.86	0.00	0.00
ENSSSCG00000002297	RDH12	11.34	2.51	0.71	3.53	0.00	0.00
ENSSSCG00000027325	TRNP1	139.16	2.51	0.19	12.90	0.00	0.00
ENSSSCG00000040140	CD3E	177.78	2.51	0.18	13.78	0.00	0.00
ENSSSCG00000000137	NCF4	43.08	2.51	0.35	7.25	0.00	0.00
ENSSSCG00000016567	STRIP2	88.43	2.51	0.24	10.47	0.00	0.00
ENSSSCG00000001618		11.36	2.51	0.68	3.71	0.00	0.00
ENSSSCG00000026991	LURAP1	21.56	2.52	0.50	5.01	0.00	0.00
ENSSSCG00000004752	EXD1	42.09	2.52	0.35	7.19	0.00	0.00
ENSSSCG00000034260	GDF15	254.16	2.52	0.15	17.31	0.00	0.00
ENSSSCG00000035594	PRSS22	562.24	2.52	0.10	25.09	0.00	0.00
ENSSSCG00000010461	ANKRD1	95.77	2.52	0.23	10.75	0.00	0.00
ENSSSCG00000028802		5.71	2.52	0.97	2.62	0.01	0.01
ENSSSCG00000035175		9.13	2.53	0.76	3.31	0.00	0.00
ENSSSCG00000028522	KRT10	36.63	2.53	0.38	6.65	0.00	0.00
ENSSSCG00000027875	CA14	6.87	2.53	0.86	2.95	0.00	0.00
ENSSSCG00000001882	NEIL1	177.59	2.53	0.17	14.70	0.00	0.00
ENSSSCG00000017630	EPX	4.57	2.53	1.06	2.39	0.02	0.02
ENSSSCG00000025870	HEPACAM2	8.02	2.53	0.79	3.19	0.00	0.00

ENSSSCG00000006582	S100A14	37.91	2.53	0.36	6.96	0.00	0.00
ENSSSCG00000007805	ATP2A1	40.24	2.53	0.36	7.09	0.00	0.00
ENSSSCG00000024671	WNT9A	6.90	2.54	0.87	2.92	0.00	0.01
ENSSSCG00000033142	ENDOU	4.61	2.54	1.05	2.41	0.02	0.02
ENSSSCG00000017373	CFAP97D1	10.36	2.54	0.70	3.61	0.00	0.00
ENSSSCG00000028203		17.24	2.54	0.55	4.60	0.00	0.00
ENSSSCG00000010162	SLC35F3	5.78	2.54	0.95	2.66	0.01	0.01
ENSSSCG00000015548	RGSL1	5.76	2.54	0.94	2.70	0.01	0.01
ENSSSCG00000018427	RF00100	4.61	2.54	1.06	2.39	0.02	0.02
ENSSSCG00000020764	CABP2	65.67	2.54	0.29	8.82	0.00	0.00
ENSSSCG00000012558	PIH1D3	383.25	2.54	0.12	20.43	0.00	0.00
ENSSSCG00000007656	PVRIG	3.45	2.54	1.21	2.10	0.04	0.05
ENSSSCG00000002917	NFKBID	536.15	2.54	0.10	24.62	0.00	0.00
ENSSSCG00000002669	CRISPLD2	5.77	2.54	0.95	2.67	0.01	0.01
ENSSSCG00000001625	PGC	3.47	2.54	1.21	2.11	0.04	0.05
ENSSSCG00000015792	LRP2BP	79.79	2.55	0.26	9.96	0.00	0.00
ENSSSCG00000035663		244.52	2.55	0.15	17.12	0.00	0.00
ENSSSCG00000028184	DNAH1	3.48	2.55	1.22	2.08	0.04	0.05
ENSSSCG00000031963	SAXO2	222.33	2.56	0.15	16.61	0.00	0.00
ENSSSCG00000015413	FGL2	33.75	2.56	0.39	6.61	0.00	0.00
ENSSSCG00000009784	ABCB9	73.39	2.56	0.27	9.53	0.00	0.00
ENSSSCG00000034056		9.31	2.56	0.74	3.44	0.00	0.00
ENSSSCG00000039918	SRR	735.20	2.56	0.09	27.37	0.00	0.00
ENSSSCG00000038190	MPST	184.50	2.56	0.17	14.92	0.00	0.00
ENSSSCG00000005250	APBA1	8.18	2.56	0.83	3.08	0.00	0.00

ENSSSCG00000040925		11.70	2.56	0.69	3.73	0.00	0.00
ENSSSCG00000016986	CREBRF	242.07	2.56	0.15	16.70	0.00	0.00
ENSSSCG00000032860	IFNLR1	116.87	2.56	0.22	11.63	0.00	0.00
ENSSSCG00000010992		40.97	2.57	0.36	7.10	0.00	0.00
ENSSSCG00000014328	GFRA3	16.38	2.57	0.57	4.48	0.00	0.00
ENSSSCG00000005858	RNF208	9.36	2.57	0.74	3.48	0.00	0.00
ENSSSCG00000010271	PRF1	5.87	2.57	0.96	2.67	0.01	0.01
ENSSSCG00000024103	ADPRHL1	8.20	2.57	0.82	3.13	0.00	0.00
ENSSSCG00000001986	NFATC4	34.16	2.58	0.39	6.65	0.00	0.00
ENSSSCG00000007384		7.08	2.58	0.85	3.04	0.00	0.00
ENSSSCG00000016742	MYO1G	28.30	2.58	0.43	6.05	0.00	0.00
ENSSSCG00000025410	PRSS16	80.08	2.58	0.26	10.10	0.00	0.00
ENSSSCG00000018734	MIR1282	40.13	2.58	0.39	6.68	0.00	0.00
ENSSSCG00000036626		5.93	2.58	0.98	2.63	0.01	0.01
ENSSSCG00000015200	ESAM	23.65	2.58	0.48	5.44	0.00	0.00
ENSSSCG00000023426		412.22	2.58	0.12	21.28	0.00	0.00
ENSSSCG00000000223	BIN2	5.92	2.59	0.92	2.81	0.01	0.01
ENSSSCG00000003000	ITPKC	329.99	2.59	0.14	18.26	0.00	0.00
ENSSSCG00000039206	UCN	28.37	2.59	0.44	5.93	0.00	0.00
ENSSSCG00000027178	MAN2B2	4.71	2.59	1.11	2.33	0.02	0.03
ENSSSCG00000033451	HIST1H2AG	29.59	2.59	0.43	6.04	0.00	0.00
ENSSSCG00000006572	NPR1	24.89	2.59	0.45	5.71	0.00	0.00
ENSSSCG00000035707		112.99	2.59	0.24	10.72	0.00	0.00
ENSSSCG00000015120	USP2	311.55	2.59	0.14	19.06	0.00	0.00
ENSSSCG00000023957	CFAP126	23.79	2.59	0.47	5.57	0.00	0.00

ENSSSCG00000006506	SYT11	116.72	2.60	0.22	12.02	0.00	0.00
ENSSSCG00000033074	ZCCHC24	4.75	2.60	1.04	2.49	0.01	0.02
ENSSSCG00000003192	IL4I1	40.52	2.60	0.36	7.23	0.00	0.00
ENSSSCG00000028108	ASAP3	57.16	2.60	0.30	8.64	0.00	0.00
ENSSSCG00000039966	NXPH4	4.75	2.60	1.07	2.44	0.01	0.02
ENSSSCG00000016557	CPA1	37.08	2.60	0.39	6.62	0.00	0.00
ENSSSCG00000027372	SAMD9	3937.46	2.60	0.06	45.31	0.00	0.00
ENSSSCG00000032327	TMEM169	25.08	2.60	0.46	5.72	0.00	0.00
ENSSSCG00000009844	HSPB8	1020.51	2.60	0.08	31.29	0.00	0.00
ENSSSCG00000007117	GZF1	1064.43	2.61	0.09	30.43	0.00	0.00
ENSSSCG00000003604	ADGRB2	15.57	2.61	0.58	4.47	0.00	0.00
ENSSSCG00000017233	RAB37	101.80	2.61	0.24	11.08	0.00	0.00
ENSSSCG00000013114	SLC15A3	27.58	2.61	0.43	6.03	0.00	0.00
ENSSSCG00000034848		7.21	2.61	0.85	3.07	0.00	0.00
ENSSSCG00000015205	HEPACAM	3.58	2.61	1.24	2.10	0.04	0.05
ENSSSCG00000037025	PLVAP	7.22	2.61	0.84	3.10	0.00	0.00
ENSSSCG00000033173	SPINK14	3.61	2.61	1.18	2.22	0.03	0.04
ENSSSCG00000036335		7.22	2.61	0.84	3.11	0.00	0.00
ENSSSCG00000012300	FOXP3	3.62	2.62	1.20	2.17	0.03	0.04
ENSSSCG00000001847	MESP1	10.87	2.62	0.72	3.66	0.00	0.00
ENSSSCG00000006051	CTHRC1	41.05	2.62	0.37	7.18	0.00	0.00
ENSSSCG00000012853	IRF7	713.09	2.62	0.09	27.72	0.00	0.00
ENSSSCG00000036956		322.23	2.62	0.14	19.21	0.00	0.00
ENSSSCG00000040224	RSPH1	44.83	2.63	0.35	7.57	0.00	0.00
ENSSSCG00000016999	TLX3	135.93	2.63	0.20	13.31	0.00	0.00

ENSSSCG00000013016	PPP2R5B	567.84	2.63	0.10	26.16	0.00	0.00
ENSSSCG00000015897	IFIH1	346.51	2.63	0.13	19.60	0.00	0.00
ENSSSCG00000038009		8.49	2.63	0.80	3.29	0.00	0.00
ENSSSCG00000004658	FBN1	13.38	2.63	0.63	4.16	0.00	0.00
ENSSSCG00000003078	CEACAM19	111.04	2.63	0.23	11.65	0.00	0.00
ENSSSCG00000027855	SOCS1	39.00	2.64	0.37	7.04	0.00	0.00
ENSSSCG00000037543		15.93	2.64	0.58	4.53	0.00	0.00
ENSSSCG00000012652	SASH3	60.13	2.65	0.30	8.91	0.00	0.00
ENSSSCG00000007935		60.18	2.65	0.30	8.87	0.00	0.00
ENSSSCG00000004705	MAP1A	362.42	2.65	0.13	20.90	0.00	0.00
ENSSSCG00000015474	PPFIA4	4.89	2.65	1.05	2.53	0.01	0.02
ENSSSCG00000025206	RNF19B	815.11	2.65	0.09	30.18	0.00	0.00
ENSSSCG00000014447	SLC6A7	4.92	2.65	1.03	2.57	0.01	0.01
ENSSSCG00000013632	C19orf38	7.38	2.65	0.84	3.16	0.00	0.00
ENSSSCG00000015659	YOD1	1913.12	2.65	0.08	34.32	0.00	0.00
ENSSSCG00000036340	ZBTB5	96.08	2.65	0.24	10.86	0.00	0.00
ENSSSCG00000017358	SLC4A1	4.93	2.65	1.02	2.60	0.01	0.01
ENSSSCG00000034763	IRS2	131.13	2.66	0.20	13.19	0.00	0.00
ENSSSCG00000039761	MYCL	175.98	2.66	0.19	14.18	0.00	0.00
ENSSSCG00000001804	HOMER2	6.22	2.67	0.93	2.88	0.00	0.01
ENSSSCG00000011904	UPK1B	474.66	2.67	0.12	22.57	0.00	0.00
ENSSSCG00000017236	CD300C	164.28	2.67	0.19	14.08	0.00	0.00
ENSSSCG00000035144	RF02138	6.24	2.67	0.92	2.89	0.00	0.01
ENSSSCG00000034119	MEIG1	24.97	2.67	0.46	5.84	0.00	0.00
ENSSSCG00000033256		71.17	2.67	0.28	9.67	0.00	0.00

ENSSSCG00000002828	LPCAT2	15.03	2.68	0.63	4.28	0.00	0.00
ENSSSCG00000013248	LRP4	30.07	2.68	0.42	6.38	0.00	0.00
ENSSSCG00000004617	FAM214A	226.56	2.68	0.16	17.00	0.00	0.00
ENSSSCG00000026323	FAM50B	3.76	2.68	1.22	2.20	0.03	0.04
ENSSSCG00000006161	IL7	3.76	2.68	1.19	2.26	0.02	0.03
ENSSSCG00000011256	SLC22A13	3.77	2.68	1.18	2.27	0.02	0.03
ENSSSCG00000033702	SBSN	30.14	2.68	0.43	6.26	0.00	0.00
ENSSSCG00000003371	GPR153	30.15	2.69	0.43	6.29	0.00	0.00
ENSSSCG00000025621	CXXC4	3.78	2.69	1.19	2.25	0.02	0.03
ENSSSCG00000017501	PNMT	3.75	2.69	1.22	2.21	0.03	0.04
ENSSSCG00000036305		169.84	2.69	0.19	14.52	0.00	0.00
ENSSSCG00000013911		1444.18	2.69	0.07	37.89	0.00	0.00
ENSSSCG00000035337		5.04	2.70	1.05	2.56	0.01	0.02
ENSSSCG00000034757	FGF11	53.13	2.70	0.32	8.37	0.00	0.00
ENSSSCG00000027486	TRIP6	16.48	2.70	0.58	4.69	0.00	0.00
ENSSSCG00000016579	CCDC136	45.60	2.70	0.36	7.58	0.00	0.00
ENSSSCG00000032620	PLCXD2	174.56	2.70	0.22	12.25	0.00	0.00
ENSSSCG00000031615	SERPINB9	69.72	2.70	0.28	9.63	0.00	0.00
ENSSSCG00000013604	MYO1F	3.81	2.70	1.20	2.26	0.02	0.03
ENSSSCG00000012975	SNX32	96.46	2.70	0.24	11.42	0.00	0.00
ENSSSCG00000012190		5.10	2.71	1.03	2.62	0.01	0.01
ENSSSCG00000024568		168.12	2.71	0.20	13.88	0.00	0.00
ENSSSCG00000001592	KCNK17	31.84	2.71	0.41	6.57	0.00	0.00
ENSSSCG00000017864		246.21	2.71	0.15	17.70	0.00	0.00
ENSSSCG00000012053	CBR3	34.46	2.71	0.40	6.79	0.00	0.00

ENSSSCG00000009865	TBX3	15.33	2.71	0.61	4.46	0.00	0.00
ENSSSCG00000003506	PINK1	209.94	2.72	0.17	16.15	0.00	0.00
ENSSSCG00000021576	CD83	121.96	2.72	0.21	12.89	0.00	0.00
ENSSSCG00000015981	HOXD10	25.81	2.73	0.46	5.99	0.00	0.00
ENSSSCG00000008062	PRSS27	12.90	2.73	0.65	4.21	0.00	0.00
ENSSSCG00000035577		74.96	2.73	0.27	10.03	0.00	0.00
ENSSSCG00000029960	LRRC4B	11.68	2.73	0.69	3.94	0.00	0.00
ENSSSCG00000036932	WNT6	19.44	2.74	0.53	5.14	0.00	0.00
ENSSSCG00000003146	NTN5	15.57	2.74	0.59	4.65	0.00	0.00
ENSSSCG00000030437	TMEM253	3.91	2.74	1.21	2.26	0.02	0.03
ENSSSCG00000015657	PIGR	211.36	2.75	0.17	16.59	0.00	0.00
ENSSSCG00000011700	CP	7.84	2.75	0.83	3.31	0.00	0.00
ENSSSCG00000039218	SLC38A8	17.00	2.75	0.58	4.77	0.00	0.00
ENSSSCG00000040019		31.42	2.75	0.42	6.60	0.00	0.00
ENSSSCG00000015126	TRIM29	22.23	2.76	0.51	5.44	0.00	0.00
ENSSSCG00000033217		6.57	2.76	0.95	2.90	0.00	0.01
ENSSSCG00000006105	GEM	1185.86	2.76	0.08	34.34	0.00	0.00
ENSSSCG00000031379	RF02137	3.95	2.76	1.17	2.36	0.02	0.03
ENSSSCG00000003993		5.26	2.76	1.04	2.65	0.01	0.01
ENSSSCG00000023972	DRAM1	19.72	2.76	0.54	5.11	0.00	0.00
ENSSSCG00000038067		143.32	2.76	0.22	12.85	0.00	0.00
ENSSSCG00000005296	PHF24	3.96	2.77	1.20	2.30	0.02	0.03
ENSSSCG00000017349	ADAM11	104.37	2.77	0.23	11.95	0.00	0.00
ENSSSCG00000026387	REEP6	36.98	2.77	0.38	7.24	0.00	0.00
ENSSSCG00000015094	CD3G	22.54	2.77	0.49	5.63	0.00	0.00

ENSSSCG00000011972	FILIP1L	354.99	2.77	0.13	20.89	0.00	0.00
ENSSSCG00000032303	ENTPD2	29.24	2.78	0.44	6.38	0.00	0.00
ENSSSCG00000027826		58.66	2.78	0.34	8.27	0.00	0.00
ENSSSCG00000017137	METRNL	85.29	2.78	0.26	10.64	0.00	0.00
ENSSSCG00000007463	PTGIS	12.00	2.78	0.69	4.05	0.00	0.00
ENSSSCG00000004012	THBS2	36.06	2.78	0.40	7.00	0.00	0.00
ENSSSCG00000008538	SPDYA	20.00	2.79	0.52	5.37	0.00	0.00
ENSSSCG00000007774	CTF1	32.00	2.79	0.42	6.59	0.00	0.00
ENSSSCG00000029037	DRD1	54.76	2.79	0.32	8.79	0.00	0.00
ENSSSCG00000028671	CEL	16.03	2.79	0.59	4.77	0.00	0.00
ENSSSCG00000039758		100.35	2.79	0.24	11.46	0.00	0.00
ENSSSCG00000003011		40.46	2.80	0.44	6.35	0.00	0.00
ENSSSCG00000000449	DTX3	240.50	2.81	0.15	18.20	0.00	0.00
ENSSSCG00000039322	NRGN	186.49	2.81	0.19	15.15	0.00	0.00
ENSSSCG00000030585	HOXC6	28.36	2.81	0.44	6.37	0.00	0.00
ENSSSCG00000013655	ICAM1	5481.09	2.81	0.05	57.55	0.00	0.00
ENSSSCG00000004760		6.77	2.81	0.91	3.08	0.00	0.00
ENSSSCG00000009979	NEFH	5.42	2.81	1.00	2.81	0.00	0.01
ENSSSCG00000008599		56.86	2.81	0.31	9.01	0.00	0.00
ENSSSCG00000016192	PLCD4	16.30	2.81	0.59	4.77	0.00	0.00
ENSSSCG00000023162	CDH3	9.50	2.81	0.76	3.71	0.00	0.00
ENSSSCG00000008266	LOXL3	31.37	2.82	0.43	6.61	0.00	0.00
ENSSSCG00000020521	RF00411	4.09	2.82	1.16	2.43	0.01	0.02
ENSSSCG00000020785	DES	46.47	2.82	0.35	8.15	0.00	0.00
ENSSSCG00000004040	SLC22A2	10.93	2.83	0.71	3.96	0.00	0.00

ENSSSCG00000008595	APOB	323.00	2.83	0.13	21.10	0.00	0.00
ENSSSCG00000036989	RF02271	19.21	2.83	0.56	5.06	0.00	0.00
ENSSSCG00000016462	CLCN1	10.97	2.83	0.73	3.87	0.00	0.00
ENSSSCG00000038969		71.37	2.83	0.28	10.10	0.00	0.00
ENSSSCG00000017295	KCNH6	10.99	2.83	0.71	3.96	0.00	0.00
ENSSSCG00000003584	THEMIS2	26.12	2.83	0.46	6.18	0.00	0.00
ENSSSCG00000036867	C11orf71	6.88	2.84	0.89	3.19	0.00	0.00
ENSSSCG00000007507	PCK1	159.80	2.84	0.22	13.20	0.00	0.00
ENSSSCG00000040906		15.18	2.84	0.61	4.68	0.00	0.00
ENSSSCG00000038600	PRSS8	98.08	2.84	0.24	11.82	0.00	0.00
ENSSSCG00000024809	CLCN4	4.17	2.84	1.18	2.41	0.02	0.02
ENSSSCG00000030368	HSPA1L	13.87	2.85	0.68	4.21	0.00	0.00
ENSSSCG00000022473	A4GNT	9.70	2.85	0.77	3.68	0.00	0.00
ENSSSCG00000003819	ANGPTL3	2.77	2.85	1.41	2.02	0.04	0.06
ENSSSCG00000001993	TGM1	56.92	2.85	0.32	9.04	0.00	0.00
ENSSSCG00000040771		2.78	2.85	1.43	2.00	0.05	0.06
ENSSSCG00000003472	ARHGEF19	58.38	2.85	0.32	8.96	0.00	0.00
ENSSSCG00000007508	ZBP1	40.32	2.85	0.37	7.64	0.00	0.00
ENSSSCG00000038912	IFITM3	938.03	2.85	0.08	33.75	0.00	0.00
ENSSSCG00000008648	RSAD2	8.36	2.86	0.81	3.52	0.00	0.00
ENSSSCG00000036007	MFAP4	2.79	2.86	1.41	2.02	0.04	0.06
ENSSSCG00000023004	FZD9	8.37	2.86	0.81	3.51	0.00	0.00
ENSSSCG00000024881	TCP11L2	61.38	2.86	0.31	9.13	0.00	0.00
ENSSSCG00000023178	BATF2	329.49	2.86	0.13	21.19	0.00	0.00
ENSSSCG00000040169		2.79	2.86	1.43	2.00	0.05	0.06

ENSSSCG00000033849	CD37	8.39	2.86	0.81	3.52	0.00	0.00
ENSSSCG00000033070		40.50	2.86	0.40	7.22	0.00	0.00
ENSSSCG00000025176	NOTCH3	8.42	2.86	0.83	3.46	0.00	0.00
ENSSSCG00000002700	SYCE1L	2.80	2.86	1.40	2.04	0.04	0.06
ENSSSCG00000008973	NAAA	71.33	2.86	0.28	10.22	0.00	0.00
ENSSSCG00000011040	CACNB2	2.80	2.86	1.45	1.97	0.05	0.06
ENSSSCG00000011412	CACNA2D2	11.21	2.87	0.75	3.83	0.00	0.00
ENSSSCG00000000668	APOBEC1	316.03	2.87	0.14	20.81	0.00	0.00
ENSSSCG00000040393	DCAF16	16.81	2.87	0.59	4.83	0.00	0.00
ENSSSCG00000033952	CITED4	2.81	2.87	1.42	2.01	0.04	0.06
ENSSSCG00000032656	BRSK1	68.87	2.87	0.29	9.95	0.00	0.00
ENSSSCG00000004109	ZC3H12D	61.94	2.87	0.32	8.95	0.00	0.00
ENSSSCG00000003155	PPP1R15A	1443.67	2.87	0.07	40.35	0.00	0.00
ENSSSCG00000008533	ALK	2.81	2.87	1.44	2.00	0.05	0.06
ENSSSCG00000032803	CYP2S1	542.08	2.87	0.12	24.93	0.00	0.00
ENSSSCG00000024914		7.03	2.88	0.90	3.20	0.00	0.00
ENSSSCG00000024299	SCN3B	148.12	2.88	0.20	14.56	0.00	0.00
ENSSSCG00000031112	TM4SF5	38.09	2.88	0.39	7.37	0.00	0.00
ENSSSCG00000036029	SLC26A10	4.23	2.88	1.16	2.48	0.01	0.02
ENSSSCG00000001753	CHRNB4	4.24	2.88	1.16	2.48	0.01	0.02
ENSSSCG00000000183	WNT1	4.24	2.88	1.16	2.48	0.01	0.02
ENSSSCG00000006173	GDAP1	4.25	2.88	1.18	2.45	0.01	0.02
ENSSSCG00000010339	DYDC2	168.23	2.88	0.19	15.48	0.00	0.00
ENSSSCG00000000492	LYZ	38.24	2.88	0.39	7.44	0.00	0.00
ENSSSCG00000004702	STRC	55.35	2.89	0.33	8.69	0.00	0.00

ENSSSCG00000024072	P2RY11	38.41	2.89	0.40	7.18	0.00	0.00
ENSSSCG00000010770	KNDC1	4.26	2.89	1.13	2.55	0.01	0.02
ENSSSCG00000032938	MFSD6L	68.24	2.89	0.30	9.77	0.00	0.00
ENSSSCG00000012077	MX1	338.60	2.89	0.14	21.28	0.00	0.00
ENSSSCG00000007421		29.87	2.89	0.44	6.64	0.00	0.00
ENSSSCG00000034765		4.28	2.89	1.16	2.49	0.01	0.02
ENSSSCG00000017956	CD68	48.46	2.89	0.36	8.08	0.00	0.00
ENSSSCG00000006213		162.31	2.89	0.19	14.96	0.00	0.00
ENSSSCG00000000040		28.51	2.89	0.45	6.46	0.00	0.00
ENSSSCG00000013731	DNASE2	262.96	2.90	0.15	18.81	0.00	0.00
ENSSSCG00000004046	PNLDC1	5.74	2.90	1.00	2.90	0.00	0.01
ENSSSCG00000032902	KLK7	15.81	2.90	0.61	4.77	0.00	0.00
ENSSSCG00000006988	PDGFRL	89.21	2.91	0.26	11.22	0.00	0.00
ENSSSCG00000032613	SNAI1	464.91	2.91	0.12	25.24	0.00	0.00
ENSSSCG00000001989	CIDEB	246.72	2.91	0.16	18.56	0.00	0.00
ENSSSCG00000035993	C1QTNF2	7.22	2.91	0.89	3.28	0.00	0.00
ENSSSCG00000003115	MEIS3	203.50	2.91	0.18	16.48	0.00	0.00
ENSSSCG00000018032	TRPV2	79.54	2.92	0.29	10.19	0.00	0.00
ENSSSCG00000021899		14.51	2.92	0.64	4.60	0.00	0.00
ENSSSCG00000024132	TMEM47	29.02	2.92	0.44	6.58	0.00	0.00
ENSSSCG00000029077	TUBAL3	10.15	2.92	0.75	3.91	0.00	0.00
ENSSSCG00000005840	C8G	8.71	2.92	0.81	3.63	0.00	0.00
ENSSSCG00000040981	GMFG	10.20	2.93	0.77	3.82	0.00	0.00
ENSSSCG00000030706	FAM43B	16.03	2.93	0.62	4.75	0.00	0.00
ENSSSCG00000012700		8.74	2.93	0.82	3.59	0.00	0.00

ENSSSCG00000009179	MTTP	40.87	2.93	0.39	7.54	0.00	0.00
ENSSSCG00000038331		10.23	2.93	0.75	3.91	0.00	0.00
ENSSSCG00000007133	ACSS1	11.67	2.93	0.71	4.15	0.00	0.00
ENSSSCG00000034633	CFAP99	11.65	2.93	0.70	4.19	0.00	0.00
ENSSSCG00000002901	UPK1A	2.92	2.94	1.43	2.06	0.04	0.05
ENSSSCG00000039797	APCDD1	14.61	2.94	0.65	4.52	0.00	0.00
ENSSSCG00000000848	GLT8D2	32.23	2.94	0.42	6.93	0.00	0.00
ENSSSCG00000014565		168.61	2.94	0.19	15.60	0.00	0.00
ENSSSCG00000022738		2.94	2.94	1.39	2.11	0.03	0.05
ENSSSCG00000008159	IL1RL1	30.87	2.94	0.43	6.79	0.00	0.00
ENSSSCG00000023841	RF00006	2.95	2.95	1.41	2.09	0.04	0.05
ENSSSCG00000007261	BPIFB1	2.95	2.95	1.38	2.13	0.03	0.05
ENSSSCG00000030906	MPL	7.35	2.95	0.88	3.35	0.00	0.00
ENSSSCG00000034297	HES2	7.40	2.95	0.91	3.23	0.00	0.00
ENSSSCG00000006463	PEAR1	14.76	2.95	0.63	4.70	0.00	0.00
ENSSSCG00000004576	RORA	56.12	2.95	0.32	9.18	0.00	0.00
ENSSSCG00000003606	SPOCD1	13.32	2.95	0.66	4.46	0.00	0.00
ENSSSCG00000037567		87.13	2.95	0.48	6.15	0.00	0.00
ENSSSCG00000001963	EGLN3	1323.26	2.95	0.17	17.26	0.00	0.00
ENSSSCG00000022026	AIPL1	2.95	2.95	1.41	2.09	0.04	0.05
ENSSSCG00000008898		99.23	2.96	0.25	11.91	0.00	0.00
ENSSSCG00000004673	SLC28A2	4.45	2.96	1.13	2.62	0.01	0.01
ENSSSCG00000034563		2.98	2.96	1.41	2.10	0.04	0.05
ENSSSCG00000013991	C1orf35	5.95	2.96	1.00	2.95	0.00	0.00
ENSSSCG00000004154	TNFAIP3	2204.08	2.96	0.15	19.60	0.00	0.00

ENSSSCG00000017446		77.34	2.96	0.28	10.48	0.00	0.00
ENSSSCG00000037096	TMEM88	19.34	2.96	0.57	5.23	0.00	0.00
ENSSSCG00000002006	PSME1	10.42	2.97	0.75	3.95	0.00	0.00
ENSSSCG00000036352	RAB7B	47.83	2.97	0.35	8.38	0.00	0.00
ENSSSCG00000023837	WFDC1	178.24	2.98	0.19	15.79	0.00	0.00
ENSSSCG00000035372	PSCA	160.41	2.98	0.20	15.20	0.00	0.00
ENSSSCG00000038825		141.05	2.98	0.22	13.78	0.00	0.00
ENSSSCG00000002001	REC8	21.03	2.98	0.53	5.64	0.00	0.00
ENSSSCG00000006204	PPP1R42	7.53	2.98	0.89	3.37	0.00	0.00
ENSSSCG00000006932		18.03	2.98	0.59	5.04	0.00	0.00
ENSSSCG00000008311	CYP26B1	110.11	2.99	0.23	12.77	0.00	0.00
ENSSSCG00000038706	C1QC	70.91	2.99	0.29	10.38	0.00	0.00
ENSSSCG00000024837	SYT12	46.77	2.99	0.35	8.44	0.00	0.00
ENSSSCG00000040205	TLCD2	109.10	2.99	0.24	12.55	0.00	0.00
ENSSSCG00000031648	CCN5	113.68	2.99	0.23	13.15	0.00	0.00
ENSSSCG00000010337	MAT1A	68.27	2.99	0.31	9.69	0.00	0.00
ENSSSCG00000015540	MR1	51.58	3.00	0.34	8.91	0.00	0.00
ENSSSCG00000012278	CFP	6.08	3.00	0.97	3.08	0.00	0.00
ENSSSCG00000035964	LCN1	6.08	3.00	0.98	3.05	0.00	0.00
ENSSSCG00000003137	PLEKHA4	450.26	3.00	0.12	24.49	0.00	0.00
ENSSSCG00000035402	RGS9	6.09	3.00	0.98	3.05	0.00	0.00
ENSSSCG00000022584	PPP1R3F	35.06	3.00	0.42	7.19	0.00	0.00
ENSSSCG00000040135		4.59	3.01	1.14	2.63	0.01	0.01
ENSSSCG00000015493	SERPINC1	4.58	3.01	1.12	2.69	0.01	0.01
ENSSSCG00000039382		4.59	3.01	1.12	2.69	0.01	0.01

ENSSSCG00000031700	SLC2A9	4.60	3.01	1.13	2.66	0.01	0.01
ENSSSCG00000007007	IDO1	23.05	3.01	0.53	5.69	0.00	0.00
ENSSSCG00000001572	FGD2	27.77	3.02	0.46	6.54	0.00	0.00
ENSSSCG00000039315	SOX10	4.66	3.03	1.14	2.65	0.01	0.01
ENSSSCG00000011890	NR1I2	13.93	3.03	0.65	4.66	0.00	0.00
ENSSSCG00000002918	HCST	10.83	3.03	0.73	4.13	0.00	0.00
ENSSSCG00000021910	GAST	13.93	3.03	0.65	4.68	0.00	0.00
ENSSSCG00000001637	GUCA1B	3.10	3.03	1.38	2.19	0.03	0.04
ENSSSCG00000003145	FUT2	3.11	3.04	1.37	2.22	0.03	0.04
ENSSSCG00000002501	BDKRB1	3.12	3.04	1.39	2.19	0.03	0.04
ENSSSCG00000011754	GHSR	3.12	3.04	1.37	2.22	0.03	0.04
ENSSSCG00000039013	NOTUM	9.36	3.04	0.80	3.80	0.00	0.00
ENSSSCG00000033548	LKAAEAR1	3.12	3.04	1.37	2.22	0.03	0.04
ENSSSCG00000003891	CYP4A24	3.13	3.04	1.40	2.18	0.03	0.04
ENSSSCG00000013864	TMEM38A	6.28	3.04	1.00	3.04	0.00	0.00
ENSSSCG00000040053	LSMEM1	7.81	3.04	0.89	3.44	0.00	0.00
ENSSSCG00000004223	HEY2	43.83	3.05	0.37	8.16	0.00	0.00
ENSSSCG00000014219	CDO1	81.55	3.05	0.28	10.92	0.00	0.00
ENSSSCG00000035447		4.75	3.06	1.15	2.67	0.01	0.01
ENSSSCG00000037644	ssc-mir-6782	22.11	3.06	0.54	5.68	0.00	0.00
ENSSSCG00000003040	ARHGEF1	26.99	3.07	0.47	6.50	0.00	0.00
ENSSSCG00000002524	AMN	9.51	3.07	0.80	3.83	0.00	0.00
ENSSSCG00000038056	B3GALT5	35.15	3.08	0.41	7.42	0.00	0.00
ENSSSCG00000002957	GGN	11.18	3.08	0.73	4.21	0.00	0.00
ENSSSCG00000006312	GPA33	6.40	3.08	0.96	3.19	0.00	0.00

ENSSSCG00000035216	SPRED3	19.24	3.08	0.56	5.48	0.00	0.00
ENSSSCG00000010099		6.41	3.09	0.97	3.18	0.00	0.00
ENSSSCG00000007435		394.92	3.09	0.13	23.87	0.00	0.00
ENSSSCG00000036056	DNAH17	24.19	3.10	0.50	6.21	0.00	0.00
ENSSSCG00000010449	CH25H	8.09	3.10	0.87	3.56	0.00	0.00
ENSSSCG00000037177	C2CD4C	8.09	3.10	0.88	3.54	0.00	0.00
ENSSSCG00000037866	MAGEA10	14.57	3.10	0.64	4.84	0.00	0.00
ENSSSCG00000012993	SLC25A45	40.60	3.10	0.39	7.88	0.00	0.00
ENSSSCG00000003399	RBP7	3.25	3.11	1.38	2.26	0.02	0.03
ENSSSCG00000003073	LYPD5	118.94	3.11	0.24	13.12	0.00	0.00
ENSSSCG00000003463	AGMAT	104.50	3.11	0.24	12.82	0.00	0.00
ENSSSCG00000012397		199.28	3.11	0.18	17.51	0.00	0.00
ENSSSCG00000006796	CHI3L2	4.90	3.12	1.13	2.76	0.01	0.01
ENSSSCG00000005946	CCN4	4.92	3.12	1.11	2.80	0.01	0.01
ENSSSCG00000006383	VANGL2	6.56	3.12	0.97	3.21	0.00	0.00
ENSSSCG00000039998	CLIC2	78.85	3.12	0.28	11.00	0.00	0.00
ENSSSCG00000039041	ZNF467	8.24	3.12	0.87	3.59	0.00	0.00
ENSSSCG00000013115	CD5	23.01	3.12	0.52	6.03	0.00	0.00
ENSSSCG00000024518		14.82	3.12	0.64	4.86	0.00	0.00
ENSSSCG00000009151	CYP2U1	406.25	3.13	0.13	23.27	0.00	0.00
ENSSSCG00000003149	TULP2	105.29	3.13	0.24	12.84	0.00	0.00
ENSSSCG00000039074	TNFRSF13C	24.71	3.13	0.51	6.13	0.00	0.00
ENSSSCG00000017223	USH1G	42.90	3.13	0.38	8.17	0.00	0.00
ENSSSCG00000035756		14.86	3.13	0.66	4.77	0.00	0.00
ENSSSCG00000000413	MYO1A	52.79	3.13	0.34	9.18	0.00	0.00

ENSSSCG00000037792	COX7A1	3.30	3.13	1.37	2.29	0.02	0.03
ENSSSCG00000040162	NUPR1	599.54	3.13	0.11	28.24	0.00	0.00
ENSSSCG00000017356	FAM171A2	134.18	3.14	0.22	14.43	0.00	0.00
ENSSSCG00000008090	IL1A	565.62	3.14	0.12	25.50	0.00	0.00
ENSSSCG00000040524	ISYNA1	166.61	3.15	0.20	16.10	0.00	0.00
ENSSSCG00000005852	ENTPD8	6.65	3.15	0.99	3.18	0.00	0.00
ENSSSCG00000003805	PDE4B	158.29	3.16	0.21	14.96	0.00	0.00
ENSSSCG00000015662	C4BPA	67.35	3.16	0.30	10.44	0.00	0.00
ENSSSCG00000032225	LRRC31	21.89	3.17	0.55	5.75	0.00	0.00
ENSSSCG00000028373	ENO2	28.68	3.17	0.46	6.83	0.00	0.00
ENSSSCG00000040773	TOB1	1209.79	3.17	0.08	38.05	0.00	0.00
ENSSSCG00000005641	DNM1	10.16	3.17	0.78	4.08	0.00	0.00
ENSSSCG00000012971	EFEMP2	59.40	3.18	0.33	9.72	0.00	0.00
ENSSSCG00000033089		5.12	3.18	1.14	2.78	0.01	0.01
ENSSSCG00000007189	SDCBP2	258.07	3.18	0.18	17.74	0.00	0.00
ENSSSCG00000008835	RASL11B	8.50	3.18	0.87	3.67	0.00	0.00
ENSSSCG00000031446		5.13	3.18	1.11	2.86	0.00	0.01
ENSSSCG00000008954		129.53	3.19	0.23	13.58	0.00	0.00
ENSSSCG00000003135	KCNJ14	124.82	3.19	0.24	13.47	0.00	0.00
ENSSSCG00000013501	CREB3L3	448.64	3.19	0.13	23.98	0.00	0.00
ENSSSCG00000002777	HSD11B2	25.63	3.19	0.50	6.35	0.00	0.00
ENSSSCG00000035971	DUSP2	37.69	3.19	0.41	7.75	0.00	0.00
ENSSSCG00000026140		39.41	3.19	0.40	7.96	0.00	0.00
ENSSSCG00000007744	PHKG1	3.44	3.20	1.34	2.39	0.02	0.02
ENSSSCG00000005125	LRRC19	3.44	3.20	1.34	2.38	0.02	0.02

ENSSSCG00000029523		6.87	3.20	0.96	3.33	0.00	0.00
ENSSSCG00000024428	CHRNA9	49.96	3.20	0.36	8.99	0.00	0.00
ENSSSCG00000014092	S100Z	8.62	3.21	0.86	3.73	0.00	0.00
ENSSSCG00000040025	NANOS1	27.69	3.21	0.47	6.76	0.00	0.00
ENSSSCG00000011775	KLHL24	235.87	3.21	0.18	17.75	0.00	0.00
ENSSSCG00000009322	SLC46A3	69.37	3.21	0.31	10.47	0.00	0.00
ENSSSCG00000025967	SMKR1	22.56	3.21	0.53	6.06	0.00	0.00
ENSSSCG00000035148	ZNF268	3.47	3.21	1.37	2.35	0.02	0.03
ENSSSCG00000040729	C11orf86	48.69	3.21	0.37	8.67	0.00	0.00
ENSSSCG00000006159	HEY1	5.24	3.22	1.09	2.95	0.00	0.00
ENSSSCG00000009935	MYO1H	5.24	3.22	1.09	2.97	0.00	0.00
ENSSSCG00000017801	TRARG1	20.99	3.22	0.55	5.88	0.00	0.00
ENSSSCG00000015116		267.63	3.22	0.16	20.17	0.00	0.00
ENSSSCG00000025052	BPIFB6	22.78	3.23	0.53	6.13	0.00	0.00
ENSSSCG00000023487	MSLNL	12.28	3.23	0.72	4.46	0.00	0.00
ENSSSCG00000010837	FAM177B	7.03	3.23	0.96	3.37	0.00	0.00
ENSSSCG00000032721	TRAF3IP3	5.27	3.23	1.10	2.94	0.00	0.01
ENSSSCG00000007216		7.04	3.23	0.96	3.38	0.00	0.00
ENSSSCG00000035360	TMEM235	12.32	3.23	0.73	4.43	0.00	0.00
ENSSSCG00000037705	SLC6A5	5.30	3.24	1.12	2.89	0.00	0.01
ENSSSCG00000003336	TTLL10	8.85	3.24	0.85	3.83	0.00	0.00
ENSSSCG00000004714	EPB42	7.10	3.24	0.96	3.37	0.00	0.00
ENSSSCG00000000419	RDH16	15.92	3.24	0.63	5.12	0.00	0.00
ENSSSCG00000029158	ZMAT2	8.82	3.24	0.87	3.75	0.00	0.00
ENSSSCG00000040751	DDIT3	1026.99	3.24	0.09	36.29	0.00	0.00

ENSSSCG00000001229		504.76	3.24	0.12	26.64	0.00	0.00
ENSSSCG00000011557	CIDEC	55.01	3.25	0.35	9.41	0.00	0.00
ENSSSCG00000013408	ADM	524.71	3.25	0.11	28.38	0.00	0.00
ENSSSCG00000035152	TEF	65.53	3.25	0.32	10.22	0.00	0.00
ENSSSCG00000039927		12.42	3.25	0.72	4.50	0.00	0.00
ENSSSCG00000040095	GPR35	58.66	3.25	0.35	9.42	0.00	0.00
ENSSSCG00000038514	DDAH2	17.84	3.26	0.59	5.48	0.00	0.00
ENSSSCG00000003306	HELZ2	8.94	3.26	0.86	3.79	0.00	0.00
ENSSSCG00000036645	PDE6B	23.28	3.26	0.52	6.23	0.00	0.00
ENSSSCG00000007593	ANKRD61	7.15	3.26	0.95	3.42	0.00	0.00
ENSSSCG00000029230	ECM1	2881.98	3.27	0.06	50.48	0.00	0.00
ENSSSCG00000031398		5.39	3.27	1.09	3.00	0.00	0.00
ENSSSCG00000000239		5.38	3.27	1.10	2.96	0.00	0.00
ENSSSCG00000029289		16.26	3.27	0.63	5.18	0.00	0.00
ENSSSCG00000006731	VTCN1	7.22	3.27	0.94	3.49	0.00	0.00
ENSSSCG00000028115	ALDH8A1	5.44	3.28	1.10	2.98	0.00	0.00
ENSSSCG00000002252	ARRDC4	1098.20	3.28	0.09	37.70	0.00	0.00
ENSSSCG00000005830		9.06	3.28	0.85	3.84	0.00	0.00
ENSSSCG00000024495		3.62	3.28	1.33	2.47	0.01	0.02
ENSSSCG00000014982	MMP7	56.08	3.28	0.35	9.45	0.00	0.00
ENSSSCG00000026754	ATCAY	25.38	3.28	0.52	6.35	0.00	0.00
ENSSSCG00000007998	RHBDL1	105.08	3.28	0.25	13.19	0.00	0.00
ENSSSCG00000027801	VTN	14.53	3.29	0.67	4.94	0.00	0.00
ENSSSCG00000028758	LBP	5.46	3.29	1.10	2.98	0.00	0.00
ENSSSCG00000026345		143.83	3.29	0.22	14.93	0.00	0.00

ENSSSCG00000008557	EMILIN1	54.65	3.29	0.35	9.35	0.00	0.00
ENSSSCG00000022017	LGALS4	54.76	3.29	0.35	9.52	0.00	0.00
ENSSSCG00000033922	ALKAL1	1.95	3.30	1.67	1.97	0.05	0.06
ENSSSCG00000032686	RUNX3	1.95	3.30	1.67	1.98	0.05	0.06
ENSSSCG00000000907	PLXNC1	1.95	3.30	1.67	1.98	0.05	0.06
ENSSSCG00000039909	ICAM2	11.00	3.30	0.77	4.31	0.00	0.00
ENSSSCG00000027252	HCFC1R1	484.06	3.30	0.13	26.39	0.00	0.00
ENSSSCG00000038898	RF00026	1.96	3.30	1.66	1.99	0.05	0.06
ENSSSCG00000009805	HPD	1.96	3.30	1.67	1.98	0.05	0.06
ENSSSCG00000008554	ABHD1	73.57	3.30	0.30	10.88	0.00	0.00
ENSSSCG00000024431	ATG9B	18.42	3.30	0.60	5.51	0.00	0.00
ENSSSCG00000018044	ALDH3A1	197.07	3.31	0.18	17.91	0.00	0.00
ENSSSCG00000003539	GRHL3	5.54	3.31	1.13	2.94	0.00	0.01
ENSSSCG00000019852	RF00420	1.97	3.31	1.66	1.99	0.05	0.06
ENSSSCG00000011487	FEZF2	5.54	3.31	1.09	3.02	0.00	0.00
ENSSSCG00000016388		1.98	3.32	1.69	1.96	0.05	0.07
ENSSSCG00000014066	TMEM171	11.16	3.32	0.77	4.29	0.00	0.00
ENSSSCG00000040934	ISX	1.99	3.32	1.69	1.97	0.05	0.07
ENSSSCG00000040579	ZNF683	1.99	3.32	1.69	1.97	0.05	0.07
ENSSSCG00000003969	GUCA2A	1.99	3.32	1.69	1.97	0.05	0.07
ENSSSCG00000024344	CCR5	5.59	3.32	1.09	3.06	0.00	0.00
ENSSSCG00000033735	PERM1	14.90	3.32	0.67	4.99	0.00	0.00
ENSSSCG00000007978		9.29	3.33	0.85	3.92	0.00	0.00
ENSSSCG00000034567		9.32	3.33	0.83	3.99	0.00	0.00
ENSSSCG00000024357	DNAI2	80.19	3.33	0.29	11.48	0.00	0.00

ENSSSCG00000032309		14.90	3.33	0.67	4.93	0.00	0.00
ENSSSCG00000006679		5.60	3.33	1.11	3.00	0.00	0.00
ENSSSCG00000034657	LHX3	3.74	3.33	1.33	2.51	0.01	0.02
ENSSSCG00000013888	JAK3	166.96	3.34	0.20	16.48	0.00	0.00
ENSSSCG00000022961	CLMP	3.76	3.34	1.35	2.47	0.01	0.02
ENSSSCG00000028345		3.77	3.34	1.34	2.50	0.01	0.02
ENSSSCG00000002536		3.77	3.35	1.32	2.54	0.01	0.02
ENSSSCG00000038037	SPTY2D1OS	18.88	3.35	0.60	5.58	0.00	0.00
ENSSSCG00000031924	NKX3-1	98.27	3.35	0.26	12.83	0.00	0.00
ENSSSCG00000029092	LRRD1	5.65	3.35	1.11	3.01	0.00	0.00
ENSSSCG00000010746	ADAM12	17.03	3.35	0.62	5.39	0.00	0.00
ENSSSCG00000010792		3.81	3.35	1.33	2.53	0.01	0.02
ENSSSCG00000024549	LYL1	26.50	3.35	0.50	6.66	0.00	0.00
ENSSSCG00000017643	SEPT4	11.39	3.35	0.78	4.30	0.00	0.00
ENSSSCG00000000275	MAP3K12	216.28	3.35	0.18	18.77	0.00	0.00
ENSSSCG00000004733	PLA2G4D	24.68	3.35	0.53	6.38	0.00	0.00
ENSSSCG00000040888	KCTD13	432.49	3.35	0.13	25.94	0.00	0.00
ENSSSCG00000006002	CCN3	41.80	3.36	0.40	8.38	0.00	0.00
ENSSSCG00000011851		17.17	3.36	0.62	5.39	0.00	0.00
ENSSSCG00000000774	USP18	21.02	3.36	0.57	5.95	0.00	0.00
ENSSSCG00000000675	C1R	106.96	3.36	0.25	13.30	0.00	0.00
ENSSSCG00000008509	RASGRP3	11.45	3.37	0.77	4.40	0.00	0.00
ENSSSCG00000023527	NRROS	5.73	3.37	1.08	3.13	0.00	0.00
ENSSSCG00000034069		7.67	3.37	0.95	3.54	0.00	0.00
ENSSSCG00000013545	TUBB4A	837.27	3.37	0.10	35.02	0.00	0.00

ENSSSCG00000023379	UBE2L6	286.66	3.38	0.16	21.15	0.00	0.00
ENSSSCG00000023329	APC2	36.55	3.38	0.43	7.84	0.00	0.00
ENSSSCG00000017717	UNC45B	19.34	3.39	0.59	5.76	0.00	0.00
ENSSSCG00000037473		9.72	3.39	0.84	4.06	0.00	0.00
ENSSSCG00000022280	DACT3	23.38	3.39	0.55	6.21	0.00	0.00
ENSSSCG00000001807	AP3B2	3.91	3.40	1.31	2.60	0.01	0.01
ENSSSCG00000032079		19.55	3.40	0.60	5.72	0.00	0.00
ENSSSCG00000011441	TNNC1	5.86	3.40	1.07	3.18	0.00	0.00
ENSSSCG00000016331	RAMP1	5.84	3.40	1.11	3.08	0.00	0.00
ENSSSCG00000037351		3.94	3.41	1.32	2.57	0.01	0.01
ENSSSCG00000036263		3.93	3.41	1.31	2.61	0.01	0.01
ENSSSCG00000000584	SLCO1A2	3.93	3.41	1.31	2.61	0.01	0.01
ENSSSCG00000033456	GPR157	17.74	3.41	0.63	5.41	0.00	0.00
ENSSSCG00000006846	HENMT1	2.09	3.41	1.70	2.01	0.04	0.06
ENSSSCG00000016706	HOXA2	5.91	3.41	1.06	3.21	0.00	0.00
ENSSSCG00000030882		3.94	3.41	1.33	2.56	0.01	0.02
ENSSSCG00000004452	PRSS35	313.01	3.41	0.15	22.02	0.00	0.00
ENSSSCG00000034606	RF02271	3.97	3.42	1.34	2.54	0.01	0.02
ENSSSCG00000015638	RHEX	3.95	3.42	1.31	2.61	0.01	0.01
ENSSSCG00000013553	VAV1	3.95	3.42	1.32	2.59	0.01	0.01
ENSSSCG00000005488		2.11	3.42	1.67	2.05	0.04	0.05
ENSSSCG00000039855	LCN9	2.11	3.42	1.64	2.09	0.04	0.05
ENSSSCG00000000232	ACVRL1	5.96	3.42	1.08	3.16	0.00	0.00
ENSSSCG00000039057	ALKAL2	2.12	3.43	1.68	2.04	0.04	0.06
ENSSSCG00000001232		2.12	3.43	1.68	2.04	0.04	0.06

ENSSSCG00000001405	LTB	2.12	3.43	1.68	2.04	0.04	0.06
ENSSSCG00000009498		2.12	3.43	1.62	2.11	0.03	0.05
ENSSSCG00000017283	CD79B	2.12	3.43	1.62	2.11	0.03	0.05
ENSSSCG00000006479		43.79	3.43	0.40	8.50	0.00	0.00
ENSSSCG00000017932	ASGR2	21.92	3.43	0.58	5.92	0.00	0.00
ENSSSCG00000016448	KCNH2	11.92	3.43	0.76	4.53	0.00	0.00
ENSSSCG00000009500	CLDN10	2.13	3.43	1.62	2.11	0.03	0.05
ENSSSCG00000035154		2.13	3.43	1.62	2.12	0.03	0.05
ENSSSCG00000037555		2.13	3.43	1.62	2.11	0.03	0.05
ENSSSCG00000038588		6.00	3.44	1.10	3.11	0.00	0.00
ENSSSCG00000011894	POPDC2	7.99	3.44	0.94	3.65	0.00	0.00
ENSSSCG00000033266	C7orf57	52.05	3.44	0.37	9.26	0.00	0.00
ENSSSCG00000005856	RNF224	2.15	3.44	1.64	2.11	0.04	0.05
ENSSSCG00000036348	PCDHB15	2.15	3.44	1.64	2.11	0.04	0.05
ENSSSCG00000013868	F2RL3	2.15	3.44	1.64	2.11	0.04	0.05
ENSSSCG00000011443	STAB1	28.17	3.45	0.49	6.98	0.00	0.00
ENSSSCG00000010086		28.25	3.45	0.50	6.96	0.00	0.00
ENSSSCG00000032240	RSPO1	2.16	3.45	1.66	2.07	0.04	0.05
ENSSSCG00000039973	TAS1R2	6.07	3.45	1.06	3.24	0.00	0.00
ENSSSCG00000003711	CABYR	44.51	3.45	0.40	8.69	0.00	0.00
ENSSSCG00000035960		6.09	3.46	1.07	3.23	0.00	0.00
ENSSSCG00000021654	ZNF641	34.40	3.46	0.45	7.72	0.00	0.00
ENSSSCG00000001723	PLA2G7	117.99	3.46	0.24	14.15	0.00	0.00
ENSSSCG00000030137	LMNTD2	18.33	3.47	0.61	5.68	0.00	0.00
ENSSSCG00000013010	NAALADL1	32.62	3.47	0.46	7.53	0.00	0.00

ENSSSCG00000032457		10.23	3.47	0.83	4.17	0.00	0.00
ENSSSCG00000032255		4.08	3.47	1.30	2.66	0.01	0.01
ENSSSCG00000039030		26.53	3.47	0.51	6.81	0.00	0.00
ENSSSCG00000016328	RAB17	8.18	3.47	0.93	3.73	0.00	0.00
ENSSSCG00000017990	PIK3R6	8.17	3.47	0.92	3.79	0.00	0.00
ENSSSCG00000023906	RF00553	4.09	3.47	1.31	2.65	0.01	0.01
ENSSSCG00000017759	ALDOC	1210.94	3.47	0.09	39.27	0.00	0.00
ENSSSCG00000009240	PLAC8	96.22	3.47	0.27	12.70	0.00	0.00
ENSSSCG00000031365		8.17	3.48	0.93	3.73	0.00	0.00
ENSSSCG00000027226	EPN3	12.30	3.48	0.76	4.59	0.00	0.00
ENSSSCG00000017410	HCRT	4.10	3.48	1.33	2.62	0.01	0.01
ENSSSCG00000039236	FGF22	4.11	3.48	1.30	2.67	0.01	0.01
ENSSSCG00000004125	STX11	269.43	3.48	0.17	20.83	0.00	0.00
ENSSSCG00000031487	LSP1	4.12	3.48	1.32	2.64	0.01	0.01
ENSSSCG00000003006	CYP2B22	24.74	3.49	0.54	6.47	0.00	0.00
ENSSSCG00000007178		10.38	3.49	0.85	4.11	0.00	0.00
ENSSSCG00000032821	FRAT1	10.38	3.49	0.83	4.23	0.00	0.00
ENSSSCG00000040893	ZNF488	39.52	3.50	0.43	8.11	0.00	0.00
ENSSSCG00000039034	DNALI1	130.00	3.51	0.24	14.85	0.00	0.00
ENSSSCG00000000240	KRT85	14.65	3.51	0.71	4.96	0.00	0.00
ENSSSCG00000004009	TCTE3	4.19	3.52	1.35	2.60	0.01	0.01
ENSSSCG00000002916	APLP1	35.95	3.52	0.45	7.85	0.00	0.00
ENSSSCG00000026618	CAVIN2	63.35	3.52	0.34	10.34	0.00	0.00
ENSSSCG00000000577	GYS2	2.26	3.53	1.64	2.15	0.03	0.04
ENSSSCG00000004565	CA12	12.73	3.53	0.75	4.68	0.00	0.00

ENSSSCG00000016442	AOC1	27.54	3.53	0.52	6.81	0.00	0.00
ENSSSCG00000002788	EXOC3L1	21.21	3.53	0.59	6.04	0.00	0.00
ENSSSCG00000017921	ZMYND15	57.36	3.53	0.36	9.74	0.00	0.00
ENSSSCG00000010725	HMX2	4.25	3.53	1.30	2.72	0.01	0.01
ENSSSCG00000002029	MYH7	2.27	3.53	1.63	2.17	0.03	0.04
ENSSSCG00000024236	MMP17	4.25	3.53	1.29	2.74	0.01	0.01
ENSSSCG00000000713		4.26	3.54	1.29	2.74	0.01	0.01
ENSSSCG00000023745	MCOLN3	4.27	3.54	1.29	2.75	0.01	0.01
ENSSSCG00000038327		4.27	3.54	1.29	2.75	0.01	0.01
ENSSSCG00000037275	SNX22	14.94	3.54	0.71	5.01	0.00	0.00
ENSSSCG00000007192	C20orf202	4.30	3.54	1.29	2.74	0.01	0.01
ENSSSCG00000037579	PPBP	2.29	3.55	1.59	2.22	0.03	0.04
ENSSSCG00000038635	ZNF554	2.29	3.55	1.59	2.23	0.03	0.04
ENSSSCG00000017383		2.30	3.55	1.60	2.22	0.03	0.04
ENSSSCG00000007745		2.30	3.55	1.61	2.20	0.03	0.04
ENSSSCG00000032353	THEGL	2.30	3.55	1.61	2.20	0.03	0.04
ENSSSCG00000017915	VMO1	419.34	3.55	0.14	26.01	0.00	0.00
ENSSSCG00000013853	HSH2D	17.24	3.55	0.65	5.50	0.00	0.00
ENSSSCG00000021617	FNDC8	2.31	3.56	1.60	2.22	0.03	0.04
ENSSSCG00000032760		2.31	3.56	1.60	2.22	0.03	0.04
ENSSSCG00000031228	OPRL1	77.68	3.56	0.31	11.59	0.00	0.00
ENSSSCG00000013559		2.31	3.56	1.64	2.17	0.03	0.04
ENSSSCG00000017129	RAB40B	28.06	3.56	0.52	6.83	0.00	0.00
ENSSSCG00000034398	C8orf37	864.45	3.56	0.10	35.58	0.00	0.00
ENSSSCG00000028816		2.32	3.56	1.61	2.22	0.03	0.04

ENSSSCG00000025787	TMEM72	2.32	3.56	1.61	2.21	0.03	0.04
ENSSSCG00000006489	TMEM79	2.33	3.57	1.64	2.17	0.03	0.04
ENSSSCG00000010055	GGT5	4.37	3.58	1.32	2.71	0.01	0.01
ENSSSCG00000038411	ssc-mir-6782	26.19	3.58	0.53	6.80	0.00	0.00
ENSSSCG00000013022	PYGM	65.57	3.58	0.34	10.60	0.00	0.00
ENSSSCG00000004699	ELL3	6.57	3.58	1.05	3.41	0.00	0.00
ENSSSCG00000037740	RF02160	4.37	3.58	1.30	2.76	0.01	0.01
ENSSSCG00000014869	LRRC32	33.03	3.59	0.47	7.56	0.00	0.00
ENSSSCG00000036474	GJB6	4.40	3.59	1.28	2.80	0.01	0.01
ENSSSCG00000032436		4.41	3.59	1.29	2.77	0.01	0.01
ENSSSCG00000001052	PHACTR1	4.42	3.59	1.28	2.82	0.00	0.01
ENSSSCG00000014315	LECT2	4.43	3.60	1.28	2.82	0.00	0.01
ENSSSCG00000030801		13.25	3.60	0.75	4.77	0.00	0.00
ENSSSCG00000003218	MYBPC2	8.88	3.60	0.92	3.93	0.00	0.00
ENSSSCG00000007199	SLC52A3	152.93	3.60	0.22	16.25	0.00	0.00
ENSSSCG00000032320	TCIM	1141.92	3.60	0.09	38.63	0.00	0.00
ENSSSCG00000018007	MYH3	17.85	3.61	0.66	5.47	0.00	0.00
ENSSSCG00000036438	GPX3	1540.51	3.61	0.08	46.22	0.00	0.00
ENSSSCG00000021434		29.00	3.61	0.51	7.06	0.00	0.00
ENSSSCG00000002385	TGFB3	37.95	3.61	0.44	8.22	0.00	0.00
ENSSSCG00000033553		36.00	3.62	0.46	7.89	0.00	0.00
ENSSSCG00000003374	ESPN	83.57	3.63	0.30	12.00	0.00	0.00
ENSSSCG00000009798	B3GNT4	20.35	3.63	0.61	5.99	0.00	0.00
ENSSSCG00000020906	TNFSF10	133.48	3.63	0.24	15.10	0.00	0.00
ENSSSCG00000035430	YPEL3	162.15	3.64	0.22	16.74	0.00	0.00

ENSSSCG00000002978	PSP-II	2.44	3.65	1.58	2.30	0.02	0.03
ENSSSCG00000024796	UPK3B	2.44	3.65	1.58	2.31	0.02	0.03
ENSSSCG00000038990		2.44	3.65	1.58	2.30	0.02	0.03
ENSSSCG00000014930		9.12	3.65	0.92	3.98	0.00	0.00
ENSSSCG00000014222	ARL14EPL	4.58	3.65	1.27	2.88	0.00	0.01
ENSSSCG00000014931		34.32	3.65	0.47	7.81	0.00	0.00
ENSSSCG00000003990		16.07	3.65	0.69	5.31	0.00	0.00
ENSSSCG00000036439	IL29	2.45	3.65	1.57	2.33	0.02	0.03
ENSSSCG00000038350	KISS1	2.45	3.65	1.57	2.33	0.02	0.03
ENSSSCG00000031524		2.45	3.65	1.60	2.28	0.02	0.03
ENSSSCG00000016502	PARP12	4.59	3.65	1.27	2.88	0.00	0.01
ENSSSCG00000009950		4.61	3.65	1.28	2.85	0.00	0.01
ENSSSCG00000017763	SLC13A2	18.36	3.65	0.64	5.74	0.00	0.00
ENSSSCG00000007365		25.30	3.65	0.55	6.67	0.00	0.00
ENSSSCG00000016184		2.46	3.66	1.57	2.33	0.02	0.03
ENSSSCG00000000740	TEX52	2.46	3.66	1.58	2.32	0.02	0.03
ENSSSCG00000025485		4.62	3.66	1.27	2.88	0.00	0.01
ENSSSCG00000037251		43.76	3.66	0.42	8.73	0.00	0.00
ENSSSCG00000008552	PRR30	29.99	3.66	0.51	7.22	0.00	0.00
ENSSSCG00000037822	FBXL13	71.59	3.66	0.33	11.27	0.00	0.00
ENSSSCG00000013181	SERPING1	348.79	3.66	0.15	24.57	0.00	0.00
ENSSSCG00000034731		88.26	3.67	0.30	12.39	0.00	0.00
ENSSSCG00000038727	GDNF	2.50	3.67	1.62	2.27	0.02	0.03
ENSSSCG00000015799	KLKB1	2.51	3.68	1.63	2.26	0.02	0.03
ENSSSCG00000001394		81.70	3.68	0.31	11.99	0.00	0.00

ENSSSCG00000031579	PCP4L1	30.35	3.68	0.50	7.32	0.00	0.00
ENSSSCG00000006624	SELENBP1	152.11	3.68	0.23	15.89	0.00	0.00
ENSSSCG00000036851		14.04	3.68	0.74	4.99	0.00	0.00
ENSSSCG00000015589	VASH2	112.63	3.69	0.26	13.94	0.00	0.00
ENSSSCG00000035996		7.04	3.69	1.04	3.55	0.00	0.00
ENSSSCG00000016182	RUFY4	4.72	3.70	1.27	2.91	0.00	0.01
ENSSSCG00000016583	FAM71F2	14.21	3.70	0.74	5.01	0.00	0.00
ENSSSCG00000017445		9.47	3.70	0.90	4.13	0.00	0.00
ENSSSCG00000031474	PTK6	23.71	3.70	0.59	6.29	0.00	0.00
ENSSSCG00000013504	TMIGD2	9.52	3.71	0.90	4.12	0.00	0.00
ENSSSCG00000009543	MYO16	4.74	3.71	1.29	2.88	0.00	0.01
ENSSSCG00000007741	NUPR2	4.77	3.71	1.26	2.94	0.00	0.01
ENSSSCG00000025975	FAM83E	7.18	3.72	1.03	3.60	0.00	0.00
ENSSSCG00000001726	ADGRF1	9.62	3.73	0.91	4.09	0.00	0.00
ENSSSCG00000037632		2.58	3.73	1.60	2.33	0.02	0.03
ENSSSCG00000024379	PCSK4	2.59	3.74	1.58	2.37	0.02	0.03
ENSSSCG00000017478	RAPGEFL1	58.29	3.74	0.37	10.13	0.00	0.00
ENSSSCG00000023710	REEP1	43.80	3.74	0.44	8.50	0.00	0.00
ENSSSCG00000003810		2.60	3.74	1.58	2.37	0.02	0.03
ENSSSCG00000002863	LRP3	2.60	3.74	1.57	2.39	0.02	0.02
ENSSSCG00000037803	MARCKS	29.25	3.74	0.53	7.02	0.00	0.00
ENSSSCG00000027404	UNC93A	19.50	3.75	0.63	5.90	0.00	0.00
ENSSSCG00000031003		2.61	3.75	1.55	2.42	0.02	0.02
ENSSSCG00000016591	PAX4	2.61	3.75	1.55	2.42	0.02	0.02
ENSSSCG00000003998	ZNF274	2.61	3.75	1.56	2.41	0.02	0.02

ENSSSCG00000011690	PLSCR4	280.89	3.75	0.17	21.82	0.00	0.00
ENSSSCG00000017991	PIK3R5	90.49	3.75	0.30	12.49	0.00	0.00
ENSSSCG00000032810	GZMM	2.62	3.75	1.56	2.41	0.02	0.02
ENSSSCG00000026537	CACNA2D4	2.62	3.75	1.55	2.42	0.02	0.02
ENSSSCG00000040575	ISG15	345.34	3.75	0.17	22.71	0.00	0.00
ENSSSCG00000001459	HLA-DOB	58.83	3.76	0.36	10.30	0.00	0.00
ENSSSCG00000013551	C3	1202.38	3.76	0.09	41.80	0.00	0.00
ENSSSCG00000015425	SLC26A5	7.39	3.76	1.04	3.60	0.00	0.00
ENSSSCG00000024537	CYP2C42	2.63	3.76	1.56	2.42	0.02	0.02
ENSSSCG00000026184	MPP4	2.64	3.76	1.57	2.40	0.02	0.02
ENSSSCG00000026849	CCNO	76.50	3.76	0.32	11.72	0.00	0.00
ENSSSCG00000022689	GADD45B	444.01	3.77	0.14	26.45	0.00	0.00
ENSSSCG00000016527	SLC13A4	12.39	3.77	0.80	4.70	0.00	0.00
ENSSSCG00000039630		10.00	3.78	0.89	4.25	0.00	0.00
ENSSSCG00000015197	SPA17	315.03	3.79	0.16	23.22	0.00	0.00
ENSSSCG00000011828	FAM43A	97.56	3.79	0.29	13.04	0.00	0.00
ENSSSCG00000021724	KIF19	10.01	3.79	0.89	4.24	0.00	0.00
ENSSSCG00000011936	ZBED2	22.58	3.79	0.60	6.35	0.00	0.00
ENSSSCG00000002736	TAT	20.02	3.79	0.64	5.94	0.00	0.00
ENSSSCG00000023714	SLC31A2	87.82	3.79	0.31	12.37	0.00	0.00
ENSSSCG00000006186	TRPA1	1.29	3.79	1.92	1.98	0.05	0.06
ENSSSCG00000027322	PGLYRP3	1.29	3.79	1.92	1.98	0.05	0.06
ENSSSCG00000031485		1.29	3.79	1.92	1.98	0.05	0.06
ENSSSCG00000001045	ELOVL2	1.29	3.79	1.91	1.99	0.05	0.06
ENSSSCG00000021963	LCN8	1.29	3.79	1.91	1.99	0.05	0.06

ENSSSCG00000031106	PLA2G2D	1.29	3.79	1.91	1.99	0.05	0.06
ENSSSCG00000036449	G6PC	1.29	3.79	1.91	1.99	0.05	0.06
ENSSSCG00000025597		15.11	3.79	0.73	5.21	0.00	0.00
ENSSSCG00000040513	AQP3	62.93	3.80	0.36	10.62	0.00	0.00
ENSSSCG00000003601	HCRTR1	1.30	3.80	1.92	1.99	0.05	0.06
ENSSSCG00000018700	RF00030	1.30	3.80	1.92	1.99	0.05	0.06
ENSSSCG00000000656	CLEC2B	1.30	3.80	1.88	2.02	0.04	0.06
ENSSSCG00000000781	ABCD2	1.30	3.80	1.88	2.02	0.04	0.06
ENSSSCG00000024791		1.30	3.80	1.88	2.02	0.04	0.06
ENSSSCG00000001552	CLPS	1.30	3.80	1.88	2.03	0.04	0.06
ENSSSCG00000017433		1.30	3.80	1.88	2.03	0.04	0.06
ENSSSCG00000021460	NYX	1.30	3.80	1.88	2.03	0.04	0.06
ENSSSCG00000031313		1.30	3.80	1.88	2.02	0.04	0.06
ENSSSCG00000000271	AMHR2	10.11	3.80	0.89	4.29	0.00	0.00
ENSSSCG00000038077	PPP1R14A	10.14	3.81	0.88	4.32	0.00	0.00
ENSSSCG00000025759		1.31	3.81	1.90	2.01	0.04	0.06
ENSSSCG00000017109	ADAMTS16	1.31	3.81	1.87	2.04	0.04	0.06
ENSSSCG00000025468		1.31	3.81	1.87	2.04	0.04	0.06
ENSSSCG00000008410	C2orf73	1.31	3.81	1.87	2.04	0.04	0.06
ENSSSCG00000034792	KLK5	1.31	3.81	1.87	2.04	0.04	0.06
ENSSSCG00000035409		1.31	3.81	1.87	2.04	0.04	0.06
ENSSSCG00000002915	KIRREL2	1.31	3.81	1.90	2.01	0.04	0.06
ENSSSCG00000012527	TCEAL9	1.31	3.81	1.90	2.01	0.04	0.06
ENSSSCG00000033416	RF00026	1.31	3.81	1.90	2.01	0.04	0.06
ENSSSCG00000032250	SRRM3	5.11	3.81	1.26	3.02	0.00	0.00

ENSSSCG00000005352	FRMPD1	5.11	3.81	1.26	3.02	0.00	0.00
ENSSSCG00000007949	SRL	5.12	3.82	1.26	3.02	0.00	0.00
ENSSSCG00000039569		5.10	3.82	1.26	3.04	0.00	0.00
ENSSSCG00000003282		10.19	3.82	0.94	4.08	0.00	0.00
ENSSSCG00000022305	WDR78	51.15	3.82	0.40	9.54	0.00	0.00
ENSSSCG00000039316	GSG1	1.32	3.82	1.88	2.04	0.04	0.06
ENSSSCG00000007240	HCK	1.32	3.82	1.90	2.02	0.04	0.06
ENSSSCG00000028145	GPR32	1.32	3.82	1.90	2.02	0.04	0.06
ENSSSCG00000033455		1.32	3.82	1.90	2.02	0.04	0.06
ENSSSCG00000033532	SBK2	1.32	3.82	1.90	2.02	0.04	0.06
ENSSSCG00000016623		7.68	3.82	1.02	3.74	0.00	0.00
ENSSSCG00000039678	TMEM269	25.67	3.82	0.57	6.70	0.00	0.00
ENSSSCG00000011618	GATA2	141.25	3.83	0.25	15.31	0.00	0.00
ENSSSCG00000034756	PLB1	10.28	3.83	0.91	4.19	0.00	0.00
ENSSSCG00000026850	SNCG	69.45	3.83	0.34	11.20	0.00	0.00
ENSSSCG00000022068		1.33	3.83	1.91	2.01	0.04	0.06
ENSSSCG00000033562	GJB7	1.33	3.83	1.91	2.01	0.04	0.06
ENSSSCG00000003498	UBXN10	2.75	3.83	1.56	2.46	0.01	0.02
ENSSSCG00000033100		43.80	3.83	0.43	8.86	0.00	0.00
ENSSSCG00000013733	BEST2	10.31	3.83	0.89	4.32	0.00	0.00
ENSSSCG00000031311		2.76	3.84	1.55	2.47	0.01	0.02
ENSSSCG00000037260		2.76	3.84	1.54	2.49	0.01	0.02
ENSSSCG00000031804		2.77	3.84	1.53	2.50	0.01	0.02
ENSSSCG00000037976		1.34	3.84	1.94	1.98	0.05	0.06
ENSSSCG00000001089	GPLD1	7.80	3.84	1.04	3.68	0.00	0.00

ENSSSCG00000027609	GC	72.85	3.85	0.34	11.23	0.00	0.00
ENSSSCG00000006867	LRRC39	7.82	3.85	1.03	3.74	0.00	0.00
ENSSSCG00000010144	ACTN2	2.79	3.85	1.54	2.50	0.01	0.02
ENSSSCG00000039445	LRRC55	5.19	3.85	1.27	3.04	0.00	0.00
ENSSSCG00000040110		2.80	3.85	1.53	2.51	0.01	0.02
ENSSSCG00000033684	CD79A	138.56	3.86	0.24	15.85	0.00	0.00
ENSSSCG00000005923	SPATC1	18.31	3.86	0.67	5.79	0.00	0.00
ENSSSCG00000015276	ETNK2	65.54	3.86	0.36	10.71	0.00	0.00
ENSSSCG00000028277		2.81	3.86	1.54	2.50	0.01	0.02
ENSSSCG00000039834	GRIN1	5.28	3.86	1.26	3.08	0.00	0.00
ENSSSCG00000034400		39.44	3.86	0.46	8.44	0.00	0.00
ENSSSCG00000033691	RAB26	2.82	3.86	1.58	2.44	0.01	0.02
ENSSSCG00000017416	DHX58	26.35	3.87	0.56	6.91	0.00	0.00
ENSSSCG00000002376	PGF	103.21	3.87	0.28	13.67	0.00	0.00
ENSSSCG00000038471	NUAK2	484.48	3.87	0.14	27.49	0.00	0.00
ENSSSCG00000035105	DUSP13	10.63	3.88	0.89	4.37	0.00	0.00
ENSSSCG00000035598	EDN1	154.41	3.88	0.24	16.03	0.00	0.00
ENSSSCG00000001395		8.02	3.89	1.01	3.84	0.00	0.00
ENSSSCG00000003834	C8B	8.03	3.89	1.01	3.84	0.00	0.00
ENSSSCG00000000636		5.36	3.89	1.26	3.08	0.00	0.00
ENSSSCG00000017583	SGCA	13.53	3.91	0.80	4.90	0.00	0.00
ENSSSCG00000009720	DDX60	13.56	3.91	0.78	5.00	0.00	0.00
ENSSSCG00000004755	DLL4	119.59	3.91	0.27	14.47	0.00	0.00
ENSSSCG00000037288	YPEL1	114.24	3.92	0.27	14.39	0.00	0.00
ENSSSCG00000018042	SLC47A2	5.44	3.92	1.25	3.13	0.00	0.00

ENSSSCG00000011349	NPG3	2.90	3.92	1.56	2.51	0.01	0.02
ENSSSCG00000039511		2.91	3.92	1.54	2.54	0.01	0.02
ENSSSCG00000038188		2.94	3.93	1.51	2.60	0.01	0.01
ENSSSCG00000038345	IKZF1	2.94	3.93	1.51	2.60	0.01	0.01
ENSSSCG00000030217	COLGALT2	2.94	3.93	1.51	2.60	0.01	0.01
ENSSSCG00000034107	RNF223	5.48	3.93	1.27	3.09	0.00	0.00
ENSSSCG00000000279	HOXC13	2.95	3.94	1.52	2.60	0.01	0.01
ENSSSCG00000010512	SLIT1	11.03	3.94	0.90	4.38	0.00	0.00
ENSSSCG00000036362	KLK14	2.96	3.94	1.52	2.60	0.01	0.01
ENSSSCG00000034609	RNF112	2.96	3.94	1.52	2.58	0.01	0.01
ENSSSCG00000040622	HIST3H2A	5.56	3.95	1.24	3.19	0.00	0.00
ENSSSCG00000022045	PTCHD3	22.26	3.95	0.62	6.38	0.00	0.00
ENSSSCG00000036223	ACKR1	1.44	3.95	1.95	2.02	0.04	0.06
ENSSSCG00000035052	KREMEN2	220.64	3.96	0.20	19.68	0.00	0.00
ENSSSCG00000012141	CA5B	14.03	3.96	0.80	4.95	0.00	0.00
ENSSSCG00000026978	ROS1	1.45	3.96	1.90	2.08	0.04	0.05
ENSSSCG00000026695	TNFSF14	1.45	3.96	1.87	2.12	0.03	0.05
ENSSSCG00000021179		1.45	3.96	1.85	2.14	0.03	0.04
ENSSSCG00000038112	SPDYC	1.45	3.96	1.85	2.14	0.03	0.04
ENSSSCG00000037190	TAGLN3	1.45	3.96	1.85	2.14	0.03	0.04
ENSSSCG00000040139	B3GNT9	16.82	3.96	0.71	5.56	0.00	0.00
ENSSSCG00000006555	C1orf189	13.99	3.96	0.78	5.06	0.00	0.00
ENSSSCG00000015203	ROBO4	33.75	3.97	0.50	7.89	0.00	0.00
ENSSSCG00000005778	HSBP1L1	188.43	3.97	0.22	17.96	0.00	0.00
ENSSSCG00000007332	GHRH	1.46	3.97	1.82	2.19	0.03	0.04

ENSSSCG00000034846		1.46	3.97	1.82	2.19	0.03	0.04
ENSSSCG00000037762	TBX1	1.46	3.97	1.82	2.19	0.03	0.04
ENSSSCG00000009645	ADAMDEC1	1.46	3.97	1.82	2.19	0.03	0.04
ENSSSCG00000011701	TM4SF18	1.46	3.97	1.82	2.19	0.03	0.04
ENSSSCG00000005591	ADGRD2	1.46	3.97	1.83	2.17	0.03	0.04
ENSSSCG00000037309	TIGD7	1.46	3.97	1.83	2.17	0.03	0.04
ENSSSCG00000039651	SLC2A5	79.05	3.97	0.34	11.85	0.00	0.00
ENSSSCG00000020967		14.12	3.97	0.78	5.10	0.00	0.00
ENSSSCG00000032857	S100A12	835.84	3.97	0.11	34.64	0.00	0.00
ENSSSCG00000000435		56.52	3.98	0.40	9.99	0.00	0.00
ENSSSCG00000010404	ALOX5	36.78	3.98	0.48	8.23	0.00	0.00
ENSSSCG00000032429	RAX2	8.49	3.98	1.01	3.96	0.00	0.00
ENSSSCG00000002990	CNTD2	1.47	3.98	1.81	2.19	0.03	0.04
ENSSSCG00000017692	LHX1	1.47	3.98	1.81	2.19	0.03	0.04
ENSSSCG00000025128	CPN2	1.47	3.98	1.81	2.19	0.03	0.04
ENSSSCG00000040960		1.47	3.98	1.81	2.19	0.03	0.04
ENSSSCG00000016832	IL7R	1.47	3.98	1.81	2.20	0.03	0.04
ENSSSCG00000026221		1.47	3.98	1.81	2.20	0.03	0.04
ENSSSCG00000030903	CDSN	1.47	3.98	1.81	2.20	0.03	0.04
ENSSSCG00000031915	ZFP82	1.47	3.98	1.81	2.20	0.03	0.04
ENSSSCG00000035197		1.47	3.98	1.81	2.20	0.03	0.04
ENSSSCG00000040594	C3orf20	1.47	3.98	1.81	2.20	0.03	0.04
ENSSSCG00000004285	OOEP	1.47	3.98	1.81	2.19	0.03	0.04
ENSSSCG00000012607		1.47	3.98	1.81	2.19	0.03	0.04
ENSSSCG00000002487	SERPINA3-2	25.55	3.98	0.58	6.83	0.00	0.00

ENSSSCG00000015291	LEMD1	1.48	3.99	1.84	2.17	0.03	0.04
ENSSSCG00000017098	C5orf49	1.48	3.99	1.84	2.17	0.03	0.04
ENSSSCG00000034385	ADAD2	1.48	3.99	1.81	2.20	0.03	0.04
ENSSSCG00000002734	MARVELD3	1.48	3.99	1.84	2.17	0.03	0.04
ENSSSCG00000005311	CD72	1.48	3.99	1.84	2.17	0.03	0.04
ENSSSCG00000014124	ANKRD34B	1.48	3.99	1.84	2.17	0.03	0.04
ENSSSCG00000032664	HPCA	1.48	3.99	1.94	2.06	0.04	0.05
ENSSSCG00000006791	OVGP1	96.93	3.99	0.30	13.12	0.00	0.00
ENSSSCG00000011556		14.33	3.99	0.78	5.10	0.00	0.00
ENSSSCG00000028561	CD28	3.06	4.00	1.56	2.56	0.01	0.02
ENSSSCG00000028643	SLC6A20	1.49	4.00	1.82	2.19	0.03	0.04
ENSSSCG00000001234	TRIM10	1.49	4.00	1.84	2.17	0.03	0.04
ENSSSCG00000012380	P2RY4	1.49	4.00	1.84	2.17	0.03	0.04
ENSSSCG00000039779	CHGB	1.49	4.00	1.84	2.17	0.03	0.04
ENSSSCG00000030538	CDCP2	1.49	4.00	1.84	2.17	0.03	0.04
ENSSSCG00000032680	NRN1L	1.49	4.00	1.94	2.06	0.04	0.05
ENSSSCG00000017200	UNC13D	92.01	4.00	0.32	12.46	0.00	0.00
ENSSSCG00000004369	PRDM1	551.94	4.00	0.26	15.58	0.00	0.00
ENSSSCG00000035013		11.53	4.00	0.87	4.59	0.00	0.00
ENSSSCG00000007281	MAP1LC3A	434.91	4.00	0.15	27.15	0.00	0.00
ENSSSCG00000037973	KCNAB3	40.31	4.00	0.47	8.51	0.00	0.00
ENSSSCG00000006329	LMX1A	3.08	4.00	1.51	2.65	0.01	0.01
ENSSSCG00000014872		1.50	4.00	1.85	2.16	0.03	0.04
ENSSSCG00000021755		1.50	4.00	1.85	2.16	0.03	0.04
ENSSSCG00000028363	TMPRSS9	1.50	4.00	1.85	2.16	0.03	0.04

ENSSSCG00000025188	LEPR	3.09	4.01	1.50	2.67	0.01	0.01
ENSSSCG00000035586		14.47	4.01	0.78	5.16	0.00	0.00
ENSSSCG00000000067	CHADL	3.10	4.01	1.51	2.66	0.01	0.01
ENSSSCG00000032950	SLC25A34	3.10	4.01	1.50	2.68	0.01	0.01
ENSSSCG00000013093	VWCE	3.10	4.01	1.50	2.68	0.01	0.01
ENSSSCG00000000186	RHEBL1	63.87	4.02	0.37	10.75	0.00	0.00
ENSSSCG00000012970	CTSW	3.11	4.02	1.50	2.67	0.01	0.01
ENSSSCG00000008162	IL1R1	3.11	4.02	1.50	2.67	0.01	0.01
ENSSSCG00000039573	SLPI	320.40	4.02	0.17	23.89	0.00	0.00
ENSSSCG00000034499		3.12	4.02	1.50	2.69	0.01	0.01
ENSSSCG00000037140		3.12	4.02	1.50	2.68	0.01	0.01
ENSSSCG00000027053	PACSIN1	20.49	4.03	0.65	6.16	0.00	0.00
ENSSSCG00000024048	MMRN2	99.65	4.03	0.31	13.00	0.00	0.00
ENSSSCG00000027882	C1orf115	395.92	4.03	0.16	25.88	0.00	0.00
ENSSSCG00000015969	CHRNA1	5.86	4.03	1.22	3.30	0.00	0.00
ENSSSCG00000006588	S100A9	117.61	4.03	0.29	14.03	0.00	0.00
ENSSSCG00000017886	FBXO39	117.74	4.04	0.28	14.47	0.00	0.00
ENSSSCG00000006384	NHLH1	11.83	4.04	0.87	4.66	0.00	0.00
ENSSSCG00000025126	LGI4	14.80	4.04	0.78	5.18	0.00	0.00
ENSSSCG00000003101	HIF3A	17.80	4.05	0.70	5.75	0.00	0.00
ENSSSCG00000004670	C15orf48	35.65	4.05	0.50	8.14	0.00	0.00
ENSSSCG00000031086		14.91	4.06	0.78	5.21	0.00	0.00
ENSSSCG00000024930		14.91	4.06	0.78	5.23	0.00	0.00
ENSSSCG00000024926	ASGR1	23.88	4.06	0.61	6.62	0.00	0.00
ENSSSCG00000016746	NPC1L1	36.01	4.07	0.51	7.92	0.00	0.00

ENSSSCG00000028488	LTC4S	9.05	4.07	1.00	4.06	0.00	0.00
ENSSSCG00000039340	CCDC184	3.21	4.07	1.55	2.63	0.01	0.01
ENSSSCG00000032299	LEFTY2	6.03	4.07	1.22	3.35	0.00	0.00
ENSSSCG00000014368		6.05	4.08	1.22	3.33	0.00	0.00
ENSSSCG00000016203	CFAP65	27.33	4.08	0.57	7.11	0.00	0.00
ENSSSCG00000033613	FOXS1	276.35	4.08	0.19	21.55	0.00	0.00
ENSSSCG00000009997	OSM	6.08	4.09	1.24	3.30	0.00	0.00
ENSSSCG00000034623	ADAM2	3.25	4.09	1.50	2.73	0.01	0.01
ENSSSCG00000007239	CCM2L	15.20	4.09	0.77	5.29	0.00	0.00
ENSSSCG00000002983	LGALS13	3.26	4.09	1.48	2.76	0.01	0.01
ENSSSCG00000021862	PTGER2	3.26	4.09	1.49	2.75	0.01	0.01
ENSSSCG00000021534	SLC30A3	61.00	4.09	0.39	10.61	0.00	0.00
ENSSSCG00000036119	LYPD2	3.28	4.10	1.48	2.77	0.01	0.01
ENSSSCG00000009601	SLC18A1	3.28	4.10	1.49	2.75	0.01	0.01
ENSSSCG00000025772	GPR45	3.28	4.10	1.49	2.75	0.01	0.01
ENSSSCG00000035297	ISG12(A)	6.13	4.10	1.26	3.25	0.00	0.00
ENSSSCG00000009062	MGARP	46.03	4.10	0.45	9.13	0.00	0.00
ENSSSCG00000011287	HHATL	3.29	4.10	1.49	2.75	0.01	0.01
ENSSSCG00000003989		1.60	4.10	1.90	2.16	0.03	0.04
ENSSSCG00000034144		3.32	4.11	1.50	2.74	0.01	0.01
ENSSSCG00000025789		1.61	4.11	1.86	2.21	0.03	0.04
ENSSSCG00000009237	HPSE	27.84	4.11	0.57	7.20	0.00	0.00
ENSSSCG00000021483	NPBWR1	1.61	4.11	1.80	2.29	0.02	0.03
ENSSSCG00000040438		1.61	4.11	1.80	2.29	0.02	0.03
ENSSSCG00000037551		1.61	4.11	1.81	2.28	0.02	0.03

ENSSSCG00000025856	TMEM106A	204.36	4.11	0.22	18.94	0.00	0.00
ENSSSCG00000016582	FAM71F1	9.31	4.12	0.99	4.16	0.00	0.00
ENSSSCG00000036331		15.56	4.12	0.77	5.32	0.00	0.00
ENSSSCG00000014789		9.31	4.12	1.00	4.14	0.00	0.00
ENSSSCG00000010358	OPN4	18.67	4.12	0.70	5.86	0.00	0.00
ENSSSCG00000033277	IQCF5	1.62	4.12	1.77	2.33	0.02	0.03
ENSSSCG00000003391	CA6	1.62	4.12	1.79	2.31	0.02	0.03
ENSSSCG00000005463	SHOC1	1.62	4.12	1.77	2.33	0.02	0.03
ENSSSCG00000039416	CXCR4	1.63	4.13	1.77	2.33	0.02	0.03
ENSSSCG00000023338	CHRNA6	1.63	4.13	1.76	2.35	0.02	0.03
ENSSSCG00000036443	HECW1	1.63	4.13	1.76	2.35	0.02	0.03
ENSSSCG00000039983	GJA4	1.63	4.13	1.76	2.35	0.02	0.03
ENSSSCG00000010065	GSTT4	1.63	4.13	1.80	2.29	0.02	0.03
ENSSSCG00000017589	DLX3	6.27	4.13	1.22	3.40	0.00	0.00
ENSSSCG00000001566	RAB44	40.73	4.13	0.48	8.67	0.00	0.00
ENSSSCG00000007232	DUSP15	65.84	4.13	0.38	10.91	0.00	0.00
ENSSSCG00000026701		59.59	4.13	0.40	10.31	0.00	0.00
ENSSSCG00000010452	IFIT1	43.91	4.13	0.46	8.99	0.00	0.00
ENSSSCG00000012912	TBC1D10C	15.71	4.13	0.78	5.33	0.00	0.00
ENSSSCG00000008465	KCNG3	1.64	4.14	1.76	2.35	0.02	0.03
ENSSSCG00000034820	MAFA	1.64	4.14	1.76	2.35	0.02	0.03
ENSSSCG00000012169		1.64	4.14	1.75	2.36	0.02	0.03
ENSSSCG00000011326	PTH1R	1.64	4.14	1.76	2.35	0.02	0.03
ENSSSCG00000026526	CATSPER4	1.64	4.14	1.76	2.35	0.02	0.03
ENSSSCG00000033759	TBXA2R	1.64	4.14	1.76	2.35	0.02	0.03

ENSSSCG00000035121	RHBG	474.35	4.14	0.15	28.37	0.00	0.00
ENSSSCG00000027024	ASIC3	12.58	4.14	0.86	4.83	0.00	0.00
ENSSSCG00000006165	SEC31B	56.72	4.14	0.40	10.25	0.00	0.00
ENSSSCG00000025795	CSF3R	1.65	4.14	1.76	2.35	0.02	0.03
ENSSSCG00000027409	TCF23	1.65	4.14	1.76	2.35	0.02	0.03
ENSSSCG00000003286		1.65	4.14	1.76	2.35	0.02	0.03
ENSSSCG00000038055	CORO1A	1.65	4.14	1.76	2.35	0.02	0.03
ENSSSCG00000017005	KCNMB1	1.65	4.14	1.79	2.32	0.02	0.03
ENSSSCG00000027762	TNFRSF11B	1.65	4.14	1.79	2.32	0.02	0.03
ENSSSCG00000039557	TRIM54	22.24	4.15	0.66	6.26	0.00	0.00
ENSSSCG00000034644		1.66	4.15	1.78	2.33	0.02	0.03
ENSSSCG00000036209	RF02142	1.66	4.15	1.80	2.31	0.02	0.03
ENSSSCG00000037775		1.66	4.15	1.80	2.31	0.02	0.03
ENSSSCG00000007231	MYLK2	15.88	4.15	0.77	5.39	0.00	0.00
ENSSSCG00000010971	ARID3C	15.92	4.16	0.77	5.43	0.00	0.00
ENSSSCG00000017222	OTOP2	6.40	4.16	1.21	3.43	0.00	0.00
ENSSSCG00000034321	TP53INP1	1095.61	4.16	0.10	41.05	0.00	0.00
ENSSSCG00000010609		3.44	4.17	1.51	2.76	0.01	0.01
ENSSSCG00000007983	PDIA2	9.66	4.17	0.99	4.23	0.00	0.00
ENSSSCG00000007674	ZAN	19.31	4.17	0.72	5.82	0.00	0.00
ENSSSCG00000016260	SLC16A14	25.80	4.18	0.62	6.75	0.00	0.00
ENSSSCG00000032849	MLPH	3.47	4.18	1.48	2.82	0.00	0.01
ENSSSCG00000015986	HOXD1	6.48	4.19	1.22	3.44	0.00	0.00
ENSSSCG00000025629	COLEC11	6.52	4.19	1.20	3.49	0.00	0.00
ENSSSCG00000013023	RASGRP2	22.89	4.20	0.66	6.37	0.00	0.00

ENSSSCG00000025345	GUCY2D	58.89	4.20	0.40	10.37	0.00	0.00
ENSSSCG00000005844	NRARP	19.63	4.20	0.70	6.02	0.00	0.00
ENSSSCG00000032438	FFAR2	6.55	4.20	1.20	3.50	0.00	0.00
ENSSSCG00000035998	C4orf54	65.51	4.20	0.38	10.99	0.00	0.00
ENSSSCG00000033193	TPO	6.56	4.20	1.21	3.47	0.00	0.00
ENSSSCG00000009530		45.98	4.20	0.46	9.07	0.00	0.00
ENSSSCG00000014823	P2RY6	16.49	4.21	0.79	5.35	0.00	0.00
ENSSSCG00000017909	CHRNE	19.83	4.21	0.69	6.08	0.00	0.00
ENSSSCG00000001931		82.80	4.22	0.35	11.92	0.00	0.00
ENSSSCG00000027621	NDUFA4L2	16.69	4.23	0.76	5.56	0.00	0.00
ENSSSCG00000001422	C2	46.80	4.23	0.46	9.26	0.00	0.00
ENSSSCG00000000234	GRASP	57.01	4.23	0.42	10.05	0.00	0.00
ENSSSCG00000028858	FAM83C	3.59	4.24	1.46	2.90	0.00	0.01
ENSSSCG00000013041	FERMT3	3.60	4.24	1.46	2.91	0.00	0.01
ENSSSCG00000008841	PDGFRA	3.61	4.24	1.47	2.89	0.00	0.01
ENSSSCG00000006472	CRABP2	249.56	4.24	0.21	20.48	0.00	0.00
ENSSSCG00000034371		30.41	4.25	0.59	7.22	0.00	0.00
ENSSSCG00000010481	LGI1	1.77	4.25	1.76	2.42	0.02	0.02
ENSSSCG00000039984	KLK6	3.63	4.25	1.48	2.87	0.00	0.01
ENSSSCG00000005737	GFI1B	3.64	4.25	1.50	2.84	0.00	0.01
ENSSSCG00000012384	AWAT2	1.78	4.26	1.73	2.46	0.01	0.02
ENSSSCG00000004157	IL20RA	1.78	4.26	1.73	2.47	0.01	0.02
ENSSSCG00000007263		1.78	4.26	1.73	2.47	0.01	0.02
ENSSSCG00000009729	ZNF84	1.78	4.26	1.73	2.47	0.01	0.02
ENSSSCG00000031750		1.78	4.26	1.73	2.47	0.01	0.02

ENSSSCG00000038616		1.78	4.26	1.75	2.43	0.02	0.02
ENSSSCG00000009513	SLC15A1	1.78	4.26	1.73	2.47	0.01	0.02
ENSSSCG00000001698	CAPN11	1.78	4.26	1.73	2.46	0.01	0.02
ENSSSCG00000014988	MMP13	413.05	4.26	0.16	25.87	0.00	0.00
ENSSSCG00000039446		1.79	4.26	1.77	2.41	0.02	0.02
ENSSSCG00000040897		1.79	4.26	1.73	2.46	0.01	0.02
ENSSSCG00000007859	UMOD	1.79	4.26	1.72	2.48	0.01	0.02
ENSSSCG00000004021	PRR18	1.79	4.26	1.71	2.49	0.01	0.02
ENSSSCG00000033329	SLC47A1	1.79	4.26	1.71	2.49	0.01	0.02
ENSSSCG00000040610		1.79	4.26	1.71	2.49	0.01	0.02
ENSSSCG00000024872	CPT1C	1.79	4.26	1.72	2.48	0.01	0.02
ENSSSCG00000006862	VCAM1	229.37	4.27	0.22	19.66	0.00	0.00
ENSSSCG00000028661	ENKD1	13.71	4.27	0.85	5.02	0.00	0.00
ENSSSCG00000025335	ZSCAN31	1.80	4.27	1.71	2.50	0.01	0.02
ENSSSCG00000028627		1.80	4.27	1.71	2.50	0.01	0.02
ENSSSCG00000027849	CNR2	1.80	4.27	1.72	2.48	0.01	0.02
ENSSSCG00000030165	MAFF	415.82	4.27	0.17	25.59	0.00	0.00
ENSSSCG00000002761		6.87	4.27	1.20	3.56	0.00	0.00
ENSSSCG00000040105	SLC34A3	13.78	4.28	0.85	5.05	0.00	0.00
ENSSSCG00000022099	TP53INP2	1547.47	4.28	0.09	47.32	0.00	0.00
ENSSSCG00000006415	CADM3	1.81	4.28	1.75	2.44	0.01	0.02
ENSSSCG00000040535	LY6E	1.81	4.28	1.72	2.49	0.01	0.02
ENSSSCG00000038501		1.81	4.28	1.75	2.45	0.01	0.02
ENSSSCG00000008453	ABCG8	1.82	4.28	1.73	2.48	0.01	0.02
ENSSSCG00000003259	OSCAR	1.82	4.28	1.75	2.45	0.01	0.02

ENSSSCG00000013934	CILP2	1.82	4.28	1.75	2.45	0.01	0.02
ENSSSCG00000017548	NGFR	76.27	4.29	0.36	11.77	0.00	0.00
ENSSSCG00000033513		1.83	4.29	1.74	2.46	0.01	0.02
ENSSSCG00000036549	DPYSL3	1.83	4.29	1.74	2.46	0.01	0.02
ENSSSCG00000032282	ACP5	115.02	4.29	0.30	14.55	0.00	0.00
ENSSSCG00000000080	GRAP2	3.74	4.30	1.47	2.93	0.00	0.01
ENSSSCG00000009388	KCNRG	13.97	4.30	0.86	5.03	0.00	0.00
ENSSSCG00000003071	ZNF404	3.75	4.30	1.45	2.96	0.00	0.00
ENSSSCG00000004918	ALPK2	3.77	4.31	1.45	2.97	0.00	0.00
ENSSSCG00000002738	ZNF23	24.67	4.31	0.64	6.70	0.00	0.00
ENSSSCG00000034283		3.81	4.32	1.46	2.95	0.00	0.00
ENSSSCG00000003080		46.28	4.33	0.49	8.91	0.00	0.00
ENSSSCG00000013081	PPP1R32	14.31	4.33	0.84	5.13	0.00	0.00
ENSSSCG00000029311	MYPN	35.88	4.34	0.53	8.14	0.00	0.00
ENSSSCG00000034634	ssc-mir-6782	301.22	4.34	0.19	23.23	0.00	0.00
ENSSSCG00000023716	TNFAIP6	14.38	4.34	0.85	5.08	0.00	0.00
ENSSSCG00000017498	PPP1R1B	172.64	4.34	0.26	16.99	0.00	0.00
ENSSSCG00000002997		7.21	4.35	1.20	3.63	0.00	0.00
ENSSSCG00000007436	MMP9	7.21	4.35	1.19	3.64	0.00	0.00
ENSSSCG00000017981	ALOXE3	10.82	4.35	0.97	4.47	0.00	0.00
ENSSSCG00000040689	APOA4	137.34	4.35	0.29	15.19	0.00	0.00
ENSSSCG00000013892	KCNN1	3.90	4.36	1.45	3.00	0.00	0.00
ENSSSCG00000006311	DUSP27	3.92	4.37	1.44	3.04	0.00	0.00
ENSSSCG00000007146	SIGLEC1	3.92	4.37	1.44	3.04	0.00	0.00
ENSSSCG00000021902	GABRP	337.03	4.37	0.18	23.87	0.00	0.00

ENSSSCG00000013909	CRLF1	32.98	4.37	0.56	7.76	0.00	0.00
ENSSSCG00000040681	FABP4	3.95	4.38	1.45	3.02	0.00	0.00
ENSSSCG00000040910	APOH	36.77	4.38	0.53	8.26	0.00	0.00
ENSSSCG00000010190	ACTA1	3.95	4.38	1.45	3.02	0.00	0.00
ENSSSCG00000003113	C5AR2	3.95	4.38	1.44	3.03	0.00	0.00
ENSSSCG00000002464	PRIMA1	3.95	4.38	1.46	2.99	0.00	0.00
ENSSSCG00000025783	ENTPD3	47.93	4.38	0.47	9.41	0.00	0.00
ENSSSCG00000007116	CD93	1.94	4.38	1.70	2.57	0.01	0.01
ENSSSCG00000011198	RFTN1	1.94	4.38	1.70	2.57	0.01	0.01
ENSSSCG00000037400		1.94	4.38	1.69	2.59	0.01	0.01
ENSSSCG00000017121	SLC6A3	3.97	4.38	1.45	3.02	0.00	0.00
ENSSSCG00000012852	CDHR5	29.61	4.38	0.60	7.36	0.00	0.00
ENSSSCG00000040151	SMIM3	37.08	4.39	0.54	8.18	0.00	0.00
ENSSSCG00000004502	RNF165	1.95	4.39	1.68	2.62	0.01	0.01
ENSSSCG00000028376		1.95	4.39	1.68	2.62	0.01	0.01
ENSSSCG00000003318	GALP	1.95	4.39	1.68	2.62	0.01	0.01
ENSSSCG00000013111	CD6	1.95	4.39	1.68	2.62	0.01	0.01
ENSSSCG00000011793	LIPH	52.07	4.39	0.45	9.67	0.00	0.00
ENSSSCG00000026185	VWA5B2	67.01	4.39	0.40	11.00	0.00	0.00
ENSSSCG00000006336	CCDC190	1.96	4.39	1.67	2.63	0.01	0.01
ENSSSCG00000011362	TMEM89	1.96	4.39	1.67	2.63	0.01	0.01
ENSSSCG00000034914		1.96	4.39	1.67	2.62	0.01	0.01
ENSSSCG00000037694		1.96	4.39	1.69	2.60	0.01	0.01
ENSSSCG00000037638		1.97	4.40	1.69	2.61	0.01	0.01
ENSSSCG00000013887	INSL3	1.97	4.40	1.67	2.63	0.01	0.01

ENSSSCG00000029231		1.97	4.40	1.72	2.56	0.01	0.02
ENSSSCG00000003585	GJB5	74.98	4.40	0.38	11.59	0.00	0.00
ENSSSCG00000025686	KMO	11.30	4.41	0.97	4.55	0.00	0.00
ENSSSCG00000004172	SLC2A12	1.99	4.41	1.72	2.57	0.01	0.01
ENSSSCG00000017380	ARL4D	90.71	4.42	0.34	12.85	0.00	0.00
ENSSSCG00000002883		4.05	4.42	1.45	3.05	0.00	0.00
ENSSSCG00000040328	NXNL1	2.01	4.42	1.73	2.55	0.01	0.02
ENSSSCG00000002512	DEGS2	243.14	4.42	0.44	9.98	0.00	0.00
ENSSSCG00000009662	STMN4	4.07	4.43	1.43	3.09	0.00	0.00
ENSSSCG00000034799		2.02	4.43	1.75	2.53	0.01	0.02
ENSSSCG00000012076	MX2	114.93	4.44	0.31	14.39	0.00	0.00
ENSSSCG00000006595	IVL	4.11	4.44	1.44	3.08	0.00	0.00
ENSSSCG00000005629	PIP5KL1	30.87	4.45	0.59	7.48	0.00	0.00
ENSSSCG00000029323	KCNE3	19.30	4.45	0.75	5.93	0.00	0.00
ENSSSCG00000006836	KIAA1324	7.81	4.46	1.20	3.71	0.00	0.00
ENSSSCG00000013762	PODNL1	11.70	4.46	0.97	4.60	0.00	0.00
ENSSSCG00000017645	TEX14	82.01	4.47	0.37	12.15	0.00	0.00
ENSSSCG00000007495	CASS4	11.74	4.47	0.96	4.64	0.00	0.00
ENSSSCG00000009621	PHYHIP	4.22	4.48	1.44	3.12	0.00	0.00
ENSSSCG00000024765		2.09	4.49	1.69	2.65	0.01	0.01
ENSSSCG00000001705	TCTE1	4.25	4.49	1.42	3.16	0.00	0.00
ENSSSCG00000034664	MPIG6B	4.26	4.49	1.42	3.17	0.00	0.00
ENSSSCG00000011846	SLC51A	47.74	4.49	0.48	9.28	0.00	0.00
ENSSSCG00000001235	TRIM15	4.28	4.50	1.42	3.16	0.00	0.00
ENSSSCG00000002921	CLIP3	19.98	4.50	0.75	6.03	0.00	0.00

ENSSSCG00000000760	LRTM2	2.11	4.50	1.66	2.72	0.01	0.01
ENSSSCG00000030960		2.11	4.50	1.66	2.72	0.01	0.01
ENSSSCG00000008978	CXCL11	2.11	4.50	1.65	2.73	0.01	0.01
ENSSSCG00000035340		2.11	4.50	1.65	2.73	0.01	0.01
ENSSSCG00000013301	ELF5	2.11	4.50	1.64	2.74	0.01	0.01
ENSSSCG00000029513	TEX28	2.11	4.50	1.64	2.74	0.01	0.01
ENSSSCG00000014852	ARRB1	2.11	4.50	1.66	2.72	0.01	0.01
ENSSSCG00000023403		44.00	4.50	0.50	9.00	0.00	0.00
ENSSSCG00000013418	CFD	344.04	4.50	0.18	24.76	0.00	0.00
ENSSSCG00000033908	SMIM2	4.31	4.51	1.43	3.14	0.00	0.00
ENSSSCG00000021866	EDDM3B	4.31	4.51	1.44	3.13	0.00	0.00
ENSSSCG00000010717	C14H10orf120	2.12	4.51	1.64	2.75	0.01	0.01
ENSSSCG00000016486		2.12	4.51	1.64	2.75	0.01	0.01
ENSSSCG00000034284		2.12	4.51	1.64	2.75	0.01	0.01
ENSSSCG00000014987	MMP12	2.13	4.51	1.64	2.76	0.01	0.01
ENSSSCG00000022423		2.13	4.51	1.64	2.76	0.01	0.01
ENSSSCG00000040342		2.13	4.51	1.64	2.76	0.01	0.01
ENSSSCG00000003324	ZNF471	2.13	4.51	1.64	2.75	0.01	0.01
ENSSSCG00000023014		2.13	4.51	1.64	2.75	0.01	0.01
ENSSSCG00000033936		2.13	4.51	1.64	2.75	0.01	0.01
ENSSSCG00000034972	CNIH3	2.14	4.52	1.66	2.73	0.01	0.01
ENSSSCG00000038892		2.14	4.52	1.66	2.73	0.01	0.01
ENSSSCG00000017347	HIGD1B	2.15	4.53	1.66	2.72	0.01	0.01
ENSSSCG00000033941	C2orf88	20.35	4.53	0.75	6.06	0.00	0.00
ENSSSCG00000032854	C15orf65	16.36	4.54	0.83	5.50	0.00	0.00

ENSSSCG00000005616	TTC16	20.46	4.54	0.74	6.14	0.00	0.00
ENSSSCG00000010504	BLNK	4.40	4.54	1.41	3.22	0.00	0.00
ENSSSCG00000001419	SLC44A4	309.43	4.55	0.20	22.94	0.00	0.00
ENSSSCG00000035419	RARRES2	20.74	4.55	0.75	6.11	0.00	0.00
ENSSSCG00000001473	COL11A2	12.44	4.56	0.96	4.77	0.00	0.00
ENSSSCG00000038889		4.45	4.56	1.42	3.21	0.00	0.00
ENSSSCG00000021971	DPEP1	12.48	4.56	0.96	4.73	0.00	0.00
ENSSSCG00000008959	CXCL2	1732.89	4.57	0.24	19.08	0.00	0.00
ENSSSCG00000007424	TNNC2	4.49	4.57	1.46	3.12	0.00	0.00
ENSSSCG00000001691	RSPH9	54.46	4.57	0.47	9.82	0.00	0.00
ENSSSCG00000029239	MZB1	8.48	4.59	1.16	3.95	0.00	0.00
ENSSSCG00000016384		4.54	4.59	1.42	3.24	0.00	0.00
ENSSSCG00000022797	PPP1R3B	174.16	4.59	0.26	17.51	0.00	0.00
ENSSSCG00000016892	FST	50.93	4.59	0.48	9.59	0.00	0.00
ENSSSCG00000006335	RGS4	4.55	4.59	1.41	3.26	0.00	0.00
ENSSSCG00000037241	RGS2	55.27	4.59	0.46	10.03	0.00	0.00
ENSSSCG00000034878		2.26	4.60	1.63	2.82	0.00	0.01
ENSSSCG00000010348	CDHR1	4.59	4.60	1.40	3.28	0.00	0.00
ENSSSCG00000025086		2.27	4.61	1.69	2.73	0.01	0.01
ENSSSCG00000031421	DYNLRB2	34.37	4.61	0.58	7.90	0.00	0.00
ENSSSCG00000031216	HS3ST6	21.50	4.61	0.74	6.21	0.00	0.00
ENSSSCG00000039482		2.28	4.61	1.61	2.86	0.00	0.01
ENSSSCG00000013513	PLIN5	2.28	4.61	1.61	2.86	0.00	0.01
ENSSSCG00000021950	TPPP2	2.28	4.61	1.61	2.86	0.00	0.01
ENSSSCG00000023718		2.28	4.61	1.68	2.75	0.01	0.01

ENSSSCG00000007710	MLXIPL	8.61	4.61	1.17	3.95	0.00	0.00
ENSSSCG00000000400	MIP	2.29	4.62	1.63	2.83	0.00	0.01
ENSSSCG00000006736	CD2	2.29	4.62	1.63	2.83	0.00	0.01
ENSSSCG00000006403	CRP	2.29	4.62	1.61	2.87	0.00	0.01
ENSSSCG00000010506	OPALIN	2.29	4.62	1.61	2.87	0.00	0.01
ENSSSCG00000032591		2.29	4.62	1.61	2.87	0.00	0.01
ENSSSCG00000033071	SLC5A11	2.29	4.62	1.61	2.87	0.00	0.01
ENSSSCG00000040038	ZNF8	2.30	4.62	1.62	2.86	0.00	0.01
ENSSSCG00000004057	SYTL3	2.30	4.62	1.63	2.83	0.00	0.01
ENSSSCG00000003423	DRAXIN	30.47	4.63	0.62	7.44	0.00	0.00
ENSSSCG00000031916		2.31	4.63	1.63	2.84	0.00	0.01
ENSSSCG00000014235	SNCAIP	17.49	4.64	0.82	5.66	0.00	0.00
ENSSSCG00000038182		57.06	4.64	0.48	9.62	0.00	0.00
ENSSSCG00000040680		4.73	4.65	1.41	3.30	0.00	0.00
ENSSSCG00000040566		26.56	4.65	0.67	6.92	0.00	0.00
ENSSSCG00000010659	PNLIPRP1	4.76	4.66	1.40	3.33	0.00	0.00
ENSSSCG00000013654		13.32	4.66	0.96	4.88	0.00	0.00
ENSSSCG00000010224	EGR2	49.20	4.67	0.50	9.34	0.00	0.00
ENSSSCG00000023127		8.97	4.68	1.16	4.04	0.00	0.00
ENSSSCG00000040673	TMEM140	31.66	4.68	0.62	7.53	0.00	0.00
ENSSSCG00000022258		9.07	4.69	1.16	4.03	0.00	0.00
ENSSSCG00000001832	ACAN	2.41	4.69	1.65	2.85	0.00	0.01
ENSSSCG00000026602	PTGIR	22.74	4.70	0.73	6.43	0.00	0.00
ENSSSCG00000011583	TMEM40	4.87	4.70	1.40	3.35	0.00	0.00
ENSSSCG00000013765		13.66	4.70	0.95	4.95	0.00	0.00

ENSSSCG00000035511	C1QTNF1	113.97	4.70	0.33	14.04	0.00	0.00
ENSSSCG00000016676	INMT	2.42	4.70	1.61	2.91	0.00	0.01
ENSSSCG00000002709		9.15	4.70	1.15	4.08	0.00	0.00
ENSSSCG00000015199	VSIG2	2.43	4.71	1.64	2.87	0.00	0.01
ENSSSCG00000015249		2.43	4.71	1.60	2.95	0.00	0.00
ENSSSCG00000035972	TULP1	2.43	4.71	1.60	2.95	0.00	0.00
ENSSSCG00000015100	UPK2	13.80	4.71	0.95	4.98	0.00	0.00
ENSSSCG00000026861	NECTIN4	124.15	4.71	0.32	14.54	0.00	0.00
ENSSSCG00000036820		2.44	4.71	1.62	2.90	0.00	0.01
ENSSSCG00000005922		2.44	4.71	1.59	2.96	0.00	0.00
ENSSSCG00000011802	KNG1	2.44	4.71	1.59	2.96	0.00	0.00
ENSSSCG00000002829	MMP2	2.44	4.71	1.59	2.97	0.00	0.00
ENSSSCG00000036541		23.00	4.71	0.73	6.43	0.00	0.00
ENSSSCG00000032975		2.45	4.72	1.60	2.95	0.00	0.00
ENSSSCG00000011322	CCR1	2.45	4.72	1.59	2.97	0.00	0.00
ENSSSCG00000003975	KCNQ4	2.45	4.72	1.58	2.98	0.00	0.00
ENSSSCG00000009105		2.45	4.72	1.60	2.95	0.00	0.00
ENSSSCG00000037013	WFIKKN1	13.89	4.72	0.94	5.01	0.00	0.00
ENSSSCG00000017861	ASPA	2.46	4.72	1.59	2.98	0.00	0.00
ENSSSCG00000037949		2.46	4.72	1.59	2.98	0.00	0.00
ENSSSCG00000002011	CPNE6	2.46	4.72	1.59	2.96	0.00	0.00
ENSSSCG00000013932	LPAR2	13.94	4.73	0.94	5.02	0.00	0.00
ENSSSCG00000004017	FRMD1	9.29	4.73	1.15	4.10	0.00	0.00
ENSSSCG00000010448	FAS	204.75	4.73	0.25	18.64	0.00	0.00
ENSSSCG00000026339		28.22	4.74	0.67	7.12	0.00	0.00

ENSSSCG00000000248		18.84	4.75	0.82	5.81	0.00	0.00
ENSSSCG00000002814	ADGRG3	18.91	4.75	0.82	5.79	0.00	0.00
ENSSSCG00000000289	NFE2	14.20	4.76	0.95	5.00	0.00	0.00
ENSSSCG00000004195	ARG1	752.99	4.76	0.21	22.79	0.00	0.00
ENSSSCG00000031262	TXNIP	626.54	4.76	0.26	18.34	0.00	0.00
ENSSSCG00000004290	TBX18	5.12	4.76	1.40	3.41	0.00	0.00
ENSSSCG00000037087	PRRT4	5.14	4.77	1.40	3.40	0.00	0.00
ENSSSCG00000037959		9.64	4.78	1.15	4.17	0.00	0.00
ENSSSCG00000034192	GNAO1	19.34	4.79	0.81	5.88	0.00	0.00
ENSSSCG00000031874	REM2	111.40	4.79	0.34	13.99	0.00	0.00
ENSSSCG00000038651	FNDC11	5.19	4.79	1.39	3.45	0.00	0.00
ENSSSCG00000023557	CCRL2	19.38	4.79	0.82	5.87	0.00	0.00
ENSSSCG00000026317	SLC10A2	106.67	4.79	0.35	13.77	0.00	0.00
ENSSSCG00000028171	RF00026	2.58	4.79	1.59	3.01	0.00	0.00
ENSSSCG00000035736		2.58	4.79	1.59	3.01	0.00	0.00
ENSSSCG00000006286		5.21	4.80	1.39	3.46	0.00	0.00
ENSSSCG00000037626		5.22	4.80	1.38	3.47	0.00	0.00
ENSSSCG00000010278	CDH23	43.90	4.80	0.54	8.87	0.00	0.00
ENSSSCG00000035503	RF01210	9.75	4.80	1.15	4.16	0.00	0.00
ENSSSCG00000034223		2.60	4.80	1.57	3.06	0.00	0.00
ENSSSCG00000001236	TRIM40	19.55	4.80	0.82	5.88	0.00	0.00
ENSSSCG00000037484	KCNK16	5.26	4.81	1.38	3.48	0.00	0.00
ENSSSCG00000001555	SLC26A8	2.61	4.81	1.58	3.05	0.00	0.00
ENSSSCG00000032852	FAM167A	2.61	4.81	1.56	3.08	0.00	0.00
ENSSSCG00000015617	G0S2	2.61	4.81	1.57	3.07	0.00	0.00

ENSSSCG00000017691	MRM1	2.61	4.81	1.57	3.07	0.00	0.00
ENSSSCG00000013231	C1QTNF4	19.63	4.81	0.81	5.91	0.00	0.00
ENSSSCG00000021448	CCDC17	5.27	4.81	1.39	3.47	0.00	0.00
ENSSSCG00000017044	IL12B	2.62	4.81	1.56	3.08	0.00	0.00
ENSSSCG00000002720		2.62	4.81	1.56	3.08	0.00	0.00
ENSSSCG00000034701	CALY	2.62	4.81	1.56	3.08	0.00	0.00
ENSSSCG00000002668	FAM92B	2.63	4.82	1.57	3.08	0.00	0.00
ENSSSCG00000036461	CACNG5	2.63	4.82	1.57	3.06	0.00	0.00
ENSSSCG00000035429	HJV	2.63	4.82	1.59	3.03	0.00	0.00
ENSSSCG00000013358	PTPN5	9.95	4.83	1.16	4.15	0.00	0.00
ENSSSCG00000012376	GDPD2	25.00	4.84	0.73	6.64	0.00	0.00
ENSSSCG00000011201	SATB1	5.37	4.84	1.37	3.52	0.00	0.00
ENSSSCG00000016733		5.38	4.84	1.38	3.52	0.00	0.00
ENSSSCG00000016868		55.75	4.86	0.49	9.91	0.00	0.00
ENSSSCG00000030350		2.74	4.88	1.58	3.10	0.00	0.00
ENSSSCG00000012066	KCNJ15	2.75	4.88	1.61	3.04	0.00	0.00
ENSSSCG00000012760		2.75	4.88	1.56	3.12	0.00	0.00
ENSSSCG00000025416	CAMKV	2.75	4.88	1.57	3.11	0.00	0.00
ENSSSCG00000010826	MARC2	2.75	4.88	1.56	3.14	0.00	0.00
ENSSSCG00000039488	SPON2	31.06	4.89	0.66	7.42	0.00	0.00
ENSSSCG00000009936		2.76	4.89	1.55	3.16	0.00	0.00
ENSSSCG00000012824	GAB3	2.76	4.89	1.55	3.16	0.00	0.00
ENSSSCG00000002007	FITM1	5.56	4.89	1.37	3.57	0.00	0.00
ENSSSCG00000005710	LAMC3	2.78	4.90	1.54	3.18	0.00	0.00
ENSSSCG00000040648	CCL11	2.79	4.90	1.55	3.17	0.00	0.00

ENSSSCG00000017305	EFCAB13	2.79	4.90	1.56	3.15	0.00	0.00
ENSSSCG00000003333	C1QTNF12	10.47	4.90	1.14	4.31	0.00	0.00
ENSSSCG00000007528	PHACTR3	10.46	4.90	1.14	4.30	0.00	0.00
ENSSSCG00000026512		2.80	4.91	1.55	3.17	0.00	0.00
ENSSSCG00000021401	GFRA4	2.82	4.91	1.56	3.15	0.00	0.00
ENSSSCG00000011212	RARB	2.82	4.91	1.56	3.15	0.00	0.00
ENSSSCG00000001588	DNAH8	2.83	4.92	1.58	3.11	0.00	0.00
ENSSSCG00000000291	GPR84	10.58	4.92	1.14	4.32	0.00	0.00
ENSSSCG00000008165	RFX8	10.66	4.93	1.14	4.31	0.00	0.00
ENSSSCG00000004301	C6orf163	10.65	4.93	1.14	4.34	0.00	0.00
ENSSSCG00000026662		5.72	4.93	1.36	3.63	0.00	0.00
ENSSSCG00000000623	BCL2L14	26.72	4.94	0.74	6.71	0.00	0.00
ENSSSCG00000017508	STAC2	80.42	4.94	0.42	11.68	0.00	0.00
ENSSSCG00000008299	C2orf78	5.77	4.94	1.37	3.61	0.00	0.00
ENSSSCG00000003578	FGR	37.67	4.94	0.62	8.02	0.00	0.00
ENSSSCG00000023128		10.74	4.95	1.14	4.34	0.00	0.00
ENSSSCG00000027911	LTB4R	10.80	4.95	1.13	4.37	0.00	0.00
ENSSSCG00000016305	MROH2A	10.78	4.95	1.13	4.36	0.00	0.00
ENSSSCG00000031646	APOC4	2.89	4.96	1.60	3.09	0.00	0.00
ENSSSCG00000014561	NLRP6	10.89	4.96	1.14	4.34	0.00	0.00
ENSSSCG00000009630	EGR3	21.83	4.97	0.80	6.18	0.00	0.00
ENSSSCG00000032785		5.85	4.97	1.36	3.65	0.00	0.00
ENSSSCG00000011622	KBTBD12	2.92	4.97	1.54	3.23	0.00	0.00
ENSSSCG00000015652	IL10	2.93	4.97	1.52	3.26	0.00	0.00
ENSSSCG00000010291	PLA2G12B	5.89	4.98	1.36	3.67	0.00	0.00

ENSSSCG00000031717	ADCY8	2.94	4.98	1.52	3.27	0.00	0.00
ENSSSCG00000031649	RNASE1	2.95	4.98	1.53	3.26	0.00	0.00
ENSSSCG00000034174	AURKC	2.95	4.98	1.53	3.26	0.00	0.00
ENSSSCG00000015086	TMPRSS4	2.95	4.98	1.52	3.27	0.00	0.00
ENSSSCG00000015616	HSD11B1	66.30	4.99	0.46	10.73	0.00	0.00
ENSSSCG00000002033	CEBPE	2.96	4.99	1.53	3.26	0.00	0.00
ENSSSCG00000003368	RNF207	2.96	4.99	1.53	3.26	0.00	0.00
ENSSSCG00000032522		2.96	4.99	1.54	3.24	0.00	0.00
ENSSSCG00000022728	HOXC10	11.14	5.00	1.13	4.42	0.00	0.00
ENSSSCG00000008988	CCNG2	651.87	5.00	0.16	30.92	0.00	0.00
ENSSSCG00000011195	GALNT15	6.02	5.01	1.36	3.68	0.00	0.00
ENSSSCG00000014284		16.87	5.01	0.92	5.42	0.00	0.00
ENSSSCG00000002399	NGB	6.04	5.01	1.36	3.69	0.00	0.00
ENSSSCG00000019556	RF00100	90.91	5.03	0.45	11.11	0.00	0.00
ENSSSCG00000026407	NCCRP1	478.44	5.03	0.18	28.05	0.00	0.00
ENSSSCG00000035420	HES4	97.25	5.04	0.39	12.96	0.00	0.00
ENSSSCG00000007760	PRSS36	11.50	5.04	1.14	4.44	0.00	0.00
ENSSSCG00000004128	ZC2HC1B	3.07	5.04	1.53	3.30	0.00	0.00
ENSSSCG00000032643	SLC12A3	3.08	5.05	1.52	3.32	0.00	0.00
ENSSSCG00000014570	NRIP3	3.08	5.05	1.52	3.33	0.00	0.00
ENSSSCG00000013027		6.19	5.05	1.35	3.74	0.00	0.00
ENSSSCG00000033457		6.20	5.05	1.36	3.73	0.00	0.00
ENSSSCG00000002039	MMP14	3.10	5.06	1.51	3.35	0.00	0.00
ENSSSCG00000007691	ZP3	3.10	5.06	1.51	3.35	0.00	0.00
ENSSSCG00000010336	SFTPA1	3.10	5.06	1.51	3.36	0.00	0.00

ENSSSCG00000028777	MYLK4	3.10	5.06	1.51	3.36	0.00	0.00
ENSSSCG00000006465	INSRR	11.59	5.06	1.14	4.45	0.00	0.00
ENSSSCG00000022035	SPINK7	3.11	5.06	1.55	3.27	0.00	0.00
ENSSSCG00000034653	DLX4	40.65	5.06	0.61	8.33	0.00	0.00
ENSSSCG00000040431	CRYGS	6.23	5.06	1.35	3.75	0.00	0.00
ENSSSCG00000034429	PLA2G5	6.24	5.06	1.36	3.73	0.00	0.00
ENSSSCG00000032416	RGS1	3.12	5.06	1.51	3.34	0.00	0.00
ENSSSCG00000004147	ECT2L	3.15	5.07	1.53	3.31	0.00	0.00
ENSSSCG00000023460	HPCAL4	416.03	5.07	0.19	26.38	0.00	0.00
ENSSSCG00000038562	RND1	973.90	5.08	0.13	39.15	0.00	0.00
ENSSSCG00000029714	BPIFB2	17.65	5.08	0.92	5.51	0.00	0.00
ENSSSCG00000011299	CLEC3B	6.38	5.10	1.35	3.76	0.00	0.00
ENSSSCG00000004898	TNFRSF11A	6.40	5.10	1.35	3.79	0.00	0.00
ENSSSCG00000006590	S100A8	107.53	5.10	0.38	13.56	0.00	0.00
ENSSSCG00000033777	GPR152	6.43	5.10	1.35	3.78	0.00	0.00
ENSSSCG00000032108		6.44	5.11	1.36	3.77	0.00	0.00
ENSSSCG00000003967	ZMYND12	12.01	5.11	1.14	4.50	0.00	0.00
ENSSSCG00000039978		3.24	5.12	1.51	3.40	0.00	0.00
ENSSSCG00000031538	ANG	54.49	5.12	0.53	9.66	0.00	0.00
ENSSSCG00000036237	ITGA7	3.25	5.12	1.50	3.42	0.00	0.00
ENSSSCG00000038114		3.25	5.12	1.53	3.35	0.00	0.00
ENSSSCG00000032343		3.27	5.13	1.50	3.43	0.00	0.00
ENSSSCG00000010332	PLAC9	3.27	5.13	1.50	3.43	0.00	0.00
ENSSSCG00000004493	SIGLEC15	3.27	5.13	1.50	3.41	0.00	0.00
ENSSSCG00000004776	DISP2	18.30	5.13	0.92	5.56	0.00	0.00

ENSSSCG00000000121	GALR3	3.28	5.14	1.50	3.43	0.00	0.00
ENSSSCG00000010974	CNTFR	3.28	5.14	1.50	3.42	0.00	0.00
ENSSSCG00000036130		3.28	5.14	1.49	3.44	0.00	0.00
ENSSSCG00000005312	SIT1	3.28	5.14	1.51	3.39	0.00	0.00
ENSSSCG00000035950		24.43	5.14	0.80	6.38	0.00	0.00
ENSSSCG00000028741		55.07	5.14	0.53	9.60	0.00	0.00
ENSSSCG00000000657		3.29	5.14	1.50	3.43	0.00	0.00
ENSSSCG00000003794	RPE65	3.31	5.14	1.52	3.40	0.00	0.00
ENSSSCG00000024973		166.16	5.15	0.31	16.60	0.00	0.00
ENSSSCG00000019768	RF00007	24.60	5.15	0.79	6.48	0.00	0.00
ENSSSCG00000037660		3.33	5.15	1.53	3.37	0.00	0.00
ENSSSCG00000037751	OTOF	12.43	5.16	1.12	4.59	0.00	0.00
ENSSSCG00000005322	NPR2	12.47	5.17	1.13	4.56	0.00	0.00
ENSSSCG00000025748	SEBOX	6.72	5.17	1.34	3.87	0.00	0.00
ENSSSCG00000021036	RAB3B	12.56	5.18	1.12	4.62	0.00	0.00
ENSSSCG00000032298	ABCC11	6.78	5.18	1.35	3.84	0.00	0.00
ENSSSCG00000027923	GPR142	3.40	5.19	1.49	3.48	0.00	0.00
ENSSSCG00000038598	ADRB2	12.69	5.19	1.12	4.63	0.00	0.00
ENSSSCG00000013854	CIB3	3.42	5.20	1.48	3.51	0.00	0.00
ENSSSCG00000017439	KRT32	3.42	5.20	1.49	3.49	0.00	0.00
ENSSSCG00000001736	CRISP2	3.42	5.20	1.53	3.39	0.00	0.00
ENSSSCG00000017893	PIMREG	6.84	5.20	1.34	3.89	0.00	0.00
ENSSSCG00000001985	CBLN3	3.43	5.20	1.49	3.48	0.00	0.00
ENSSSCG00000025858	ELN	6.87	5.21	1.33	3.90	0.00	0.00
ENSSSCG00000004831		6.89	5.21	1.35	3.86	0.00	0.00

ENSSSCG00000039203	CYSLTR1	3.47	5.21	1.50	3.47	0.00	0.00
ENSSSCG00000010054	ADORA2A	25.75	5.21	0.80	6.54	0.00	0.00
ENSSSCG00000025097	TMEM61	3.49	5.22	1.50	3.47	0.00	0.00
ENSSSCG00000028387	CNKSR1	32.37	5.22	0.71	7.37	0.00	0.00
ENSSSCG00000000660	A2M	19.48	5.22	0.91	5.72	0.00	0.00
ENSSSCG00000035227	ESRP2	163.04	5.23	0.32	16.36	0.00	0.00
ENSSSCG00000031104		7.01	5.24	1.33	3.93	0.00	0.00
ENSSSCG00000016183		7.02	5.24	1.33	3.94	0.00	0.00
ENSSSCG00000016900	ESM1	39.29	5.24	0.65	8.09	0.00	0.00
ENSSSCG00000021696	NRSN1	3.56	5.26	1.48	3.54	0.00	0.00
ENSSSCG00000015954	DLX2	46.48	5.26	0.60	8.74	0.00	0.00
ENSSSCG00000032652	SLFN11	3.58	5.26	1.47	3.58	0.00	0.00
ENSSSCG00000023868		3.58	5.26	1.49	3.53	0.00	0.00
ENSSSCG00000036554		7.15	5.27	1.33	3.95	0.00	0.00
ENSSSCG00000001844	PLIN1	3.59	5.27	1.47	3.59	0.00	0.00
ENSSSCG00000013236	MYBPC3	13.40	5.27	1.12	4.72	0.00	0.00
ENSSSCG00000025022	FER1L5	3.60	5.27	1.49	3.53	0.00	0.00
ENSSSCG00000003650	GJA9	3.61	5.27	1.47	3.58	0.00	0.00
ENSSSCG00000003314	NLRP11	3.61	5.27	1.47	3.59	0.00	0.00
ENSSSCG00000012531		3.61	5.27	1.47	3.59	0.00	0.00
ENSSSCG00000029545	GPRC5D	3.61	5.27	1.47	3.58	0.00	0.00
ENSSSCG00000017908	GP1BA	7.20	5.27	1.33	3.98	0.00	0.00
ENSSSCG00000038149	KCNE4	3.62	5.28	1.49	3.54	0.00	0.00
ENSSSCG00000021757		7.25	5.28	1.33	3.96	0.00	0.00
ENSSSCG00000032676		13.51	5.29	1.12	4.74	0.00	0.00

ENSSSCG00000000136	CSF2RB	20.44	5.30	0.91	5.82	0.00	0.00
ENSSSCG00000025294	AIRE	7.33	5.30	1.33	4.00	0.00	0.00
ENSSSCG00000026516	EPHB3	7.36	5.31	1.32	4.01	0.00	0.00
ENSSSCG00000013360	TMEM86A	89.56	5.31	0.44	12.14	0.00	0.00
ENSSSCG00000013842	CYP4F3	3.73	5.32	1.48	3.60	0.00	0.00
ENSSSCG00000026478	PADI2	3.74	5.33	1.46	3.66	0.00	0.00
ENSSSCG00000035825	UBE2QL1	48.66	5.33	0.60	8.90	0.00	0.00
ENSSSCG00000033675	SPAI-2	3.75	5.33	1.46	3.66	0.00	0.00
ENSSSCG00000032196		3.75	5.33	1.47	3.64	0.00	0.00
ENSSSCG00000032490	LEAP2	55.95	5.34	0.56	9.59	0.00	0.00
ENSSSCG00000030195		3.77	5.34	1.46	3.66	0.00	0.00
ENSSSCG00000011627	ACPP	3.77	5.34	1.47	3.63	0.00	0.00
ENSSSCG00000015487	TNFSF18	42.01	5.34	0.65	8.27	0.00	0.00
ENSSSCG00000029284	NPHS1	7.54	5.34	1.32	4.04	0.00	0.00
ENSSSCG00000038071	RASL12	7.55	5.34	1.32	4.04	0.00	0.00
ENSSSCG00000002515	SLC25A47	14.09	5.35	1.12	4.77	0.00	0.00
ENSSSCG00000039673	ASB16	77.58	5.35	0.48	11.18	0.00	0.00
ENSSSCG00000006350		128.06	5.36	0.37	14.44	0.00	0.00
ENSSSCG00000007964		7.68	5.37	1.32	4.06	0.00	0.00
ENSSSCG00000029567	SLC8A2	3.91	5.39	1.45	3.71	0.00	0.00
ENSSSCG00000013503	SHD	3.92	5.39	1.44	3.73	0.00	0.00
ENSSSCG00000015700	TMEM163	3.92	5.39	1.44	3.73	0.00	0.00
ENSSSCG00000015653	IL19	3.92	5.39	1.48	3.65	0.00	0.00
ENSSSCG00000034163	PLN	3.93	5.40	1.45	3.73	0.00	0.00
ENSSSCG00000007186		3.93	5.40	1.44	3.74	0.00	0.00

ENSSSCG00000035521	KLHL38	3.93	5.40	1.45	3.72	0.00	0.00
ENSSSCG00000009616	HR	7.84	5.40	1.32	4.10	0.00	0.00
ENSSSCG00000038057	TAC4	14.64	5.40	1.11	4.85	0.00	0.00
ENSSSCG00000039339		3.96	5.40	1.45	3.72	0.00	0.00
ENSSSCG00000013935		3.96	5.40	1.47	3.67	0.00	0.00
ENSSSCG00000028166		3.96	5.40	1.46	3.70	0.00	0.00
ENSSSCG00000011053	CDNF	14.64	5.40	1.11	4.87	0.00	0.00
ENSSSCG00000024166	SLC2A6	147.91	5.42	0.36	15.01	0.00	0.00
ENSSSCG00000017154	CARD14	7.96	5.42	1.32	4.12	0.00	0.00
ENSSSCG00000000231	ANKRD33	14.99	5.44	1.11	4.88	0.00	0.00
ENSSSCG00000021813	SLC26A3	15.08	5.45	1.12	4.86	0.00	0.00
ENSSSCG00000002479	SERPINA11	15.10	5.45	1.11	4.91	0.00	0.00
ENSSSCG00000037364		4.07	5.45	1.44	3.77	0.00	0.00
ENSSSCG00000003386	TNFRSF9	52.88	5.45	0.59	9.21	0.00	0.00
ENSSSCG00000003482	PADI3	37.86	5.45	0.70	7.78	0.00	0.00
ENSSSCG00000034328		4.08	5.45	1.45	3.77	0.00	0.00
ENSSSCG00000007260	BPIFA1	8.15	5.46	1.32	4.15	0.00	0.00
ENSSSCG00000023225	SYN2	4.12	5.46	1.44	3.80	0.00	0.00
ENSSSCG00000037532	MNDA	4.13	5.46	1.44	3.78	0.00	0.00
ENSSSCG00000033382		15.40	5.48	1.11	4.96	0.00	0.00
ENSSSCG00000024977	NOBOX	8.35	5.49	1.31	4.18	0.00	0.00
ENSSSCG00000028284	KCND1	4.24	5.51	1.43	3.86	0.00	0.00
ENSSSCG00000014861		4.25	5.51	1.42	3.87	0.00	0.00
ENSSSCG00000013469	ZNF555	4.25	5.51	1.43	3.86	0.00	0.00
ENSSSCG00000007961		4.26	5.51	1.43	3.86	0.00	0.00

ENSSSCG00000006399	VSIG8	4.26	5.51	1.43	3.87	0.00	0.00
ENSSSCG00000011899	CD80	4.26	5.51	1.43	3.86	0.00	0.00
ENSSSCG00000017384	AOC2	181.61	5.51	0.33	16.79	0.00	0.00
ENSSSCG00000022236	FOLR1	8.49	5.52	1.31	4.23	0.00	0.00
ENSSSCG00000017883	GGT6	4.29	5.52	1.43	3.85	0.00	0.00
ENSSSCG00000014012	GFPT2	8.55	5.53	1.31	4.21	0.00	0.00
ENSSSCG00000013498	EBI3	16.10	5.54	1.10	5.03	0.00	0.00
ENSSSCG00000002821	CCL22	137.15	5.55	0.39	14.29	0.00	0.00
ENSSSCG00000007673	EPO	4.40	5.56	1.42	3.91	0.00	0.00
ENSSSCG00000029754	SLC39A2	16.28	5.56	1.10	5.05	0.00	0.00
ENSSSCG00000023151	GRIN2C	8.74	5.56	1.32	4.22	0.00	0.00
ENSSSCG00000023396	LIX1	16.36	5.57	1.10	5.06	0.00	0.00
ENSSSCG00000000385	SLC39A5	32.68	5.57	0.78	7.16	0.00	0.00
ENSSSCG00000017411	KCNH4	4.42	5.57	1.42	3.92	0.00	0.00
ENSSSCG00000035334		4.42	5.57	1.43	3.89	0.00	0.00
ENSSSCG00000033641	COL8A2	4.44	5.57	1.42	3.92	0.00	0.00
ENSSSCG00000034015		58.12	5.59	0.59	9.52	0.00	0.00
ENSSSCG00000040414		8.91	5.59	1.32	4.22	0.00	0.00
ENSSSCG00000006919		9.02	5.60	1.31	4.29	0.00	0.00
ENSSSCG00000003483		16.85	5.61	1.09	5.12	0.00	0.00
ENSSSCG00000038768	GPR37L1	4.55	5.61	1.41	3.97	0.00	0.00
ENSSSCG00000006546	TDRD10	4.55	5.61	1.42	3.95	0.00	0.00
ENSSSCG00000031321	NR4A1	1004.81	5.61	0.15	36.68	0.00	0.00
ENSSSCG00000011875	PARP15	4.58	5.62	1.41	3.99	0.00	0.00
ENSSSCG00000030681	MYBPH	4.58	5.62	1.41	3.98	0.00	0.00

ENSSSCG00000014363	SLC4A9	25.56	5.63	0.91	6.19	0.00	0.00
ENSSSCG00000034184		9.23	5.64	1.30	4.32	0.00	0.00
ENSSSCG00000012492	DRP2	9.23	5.64	1.30	4.32	0.00	0.00
ENSSSCG00000034838	MAP1LC3C	9.23	5.64	1.30	4.32	0.00	0.00
ENSSSCG00000006829	SYPL2	86.36	5.65	0.49	11.52	0.00	0.00
ENSSSCG00000024917	LRRN4CL	9.31	5.65	1.29	4.37	0.00	0.00
ENSSSCG00000029295	BPIFB4	9.33	5.65	1.29	4.37	0.00	0.00
ENSSSCG00000013473	GNA15	9.34	5.66	1.30	4.36	0.00	0.00
ENSSSCG00000033648		9.35	5.66	1.30	4.37	0.00	0.00
ENSSSCG00000030597	HAPLN3	4.71	5.66	1.41	4.03	0.00	0.00
ENSSSCG00000003576	GPR3	17.42	5.66	1.10	5.13	0.00	0.00
ENSSSCG00000039407		4.72	5.66	1.40	4.04	0.00	0.00
ENSSSCG00000015661	C4BPB	4.72	5.66	1.40	4.04	0.00	0.00
ENSSSCG00000024179	NAGS	9.39	5.66	1.30	4.35	0.00	0.00
ENSSSCG00000033623		35.16	5.67	0.78	7.28	0.00	0.00
ENSSSCG00000015069	APOC3	167.00	5.67	0.36	15.71	0.00	0.00
ENSSSCG00000001099	CMAH	4.77	5.67	1.41	4.03	0.00	0.00
ENSSSCG00000038965	ARC	26.41	5.67	0.90	6.34	0.00	0.00
ENSSSCG00000010948	CTSL	70.38	5.67	0.55	10.28	0.00	0.00
ENSSSCG00000031712	MFAP5	9.51	5.68	1.29	4.40	0.00	0.00
ENSSSCG00000005439	ACTL7A	4.87	5.71	1.40	4.07	0.00	0.00
ENSSSCG00000009100	TNIP3	99.29	5.71	0.47	12.26	0.00	0.00
ENSSSCG00000033682	CPLX2	9.71	5.71	1.30	4.41	0.00	0.00
ENSSSCG00000013031	KCNK4	4.89	5.71	1.40	4.09	0.00	0.00
ENSSSCG00000002967	CAPN12	4.91	5.72	1.40	4.09	0.00	0.00

ENSSSCG00000036190	GIPC3	4.91	5.72	1.39	4.10	0.00	0.00
ENSSSCG00000037684	PDYN	4.94	5.72	1.42	4.03	0.00	0.00
ENSSSCG00000003980	EXO5	4.96	5.73	1.41	4.07	0.00	0.00
ENSSSCG00000015223	DDX25	9.80	5.73	1.30	4.42	0.00	0.00
ENSSSCG00000024634	GPRL15	109.73	5.73	0.45	12.82	0.00	0.00
ENSSSCG00000036566	LY6G6C	120.06	5.75	0.43	13.36	0.00	0.00
ENSSSCG00000017441		5.02	5.75	1.40	4.11	0.00	0.00
ENSSSCG00000010552	PKD2L1	5.03	5.75	1.40	4.12	0.00	0.00
ENSSSCG00000036618		55.89	5.76	0.64	8.96	0.00	0.00
ENSSSCG00000040035		5.07	5.76	1.41	4.09	0.00	0.00
ENSSSCG00000003284	FCAR	5.09	5.77	1.41	4.10	0.00	0.00
ENSSSCG00000001132	BTN1A1	5.10	5.77	1.39	4.14	0.00	0.00
ENSSSCG00000037358		905.64	5.78	0.26	22.63	0.00	0.00
ENSSSCG00000032691	ANKRD66	10.14	5.78	1.29	4.48	0.00	0.00
ENSSSCG00000009002	TLR2	28.41	5.78	0.89	6.51	0.00	0.00
ENSSSCG00000028322	BTG2	379.96	5.79	0.25	22.77	0.00	0.00
ENSSSCG00000008348	PLEK	104.60	5.79	0.47	12.40	0.00	0.00
ENSSSCG00000017466	CCR7	238.02	5.79	0.32	18.25	0.00	0.00
ENSSSCG00000017755	NOS2	182.08	5.80	0.36	16.14	0.00	0.00
ENSSSCG00000000418	TAC3	5.21	5.81	1.38	4.20	0.00	0.00
ENSSSCG00000012493	TAF7L	5.21	5.81	1.39	4.19	0.00	0.00
ENSSSCG00000029371	C5AR1	5.22	5.81	1.38	4.20	0.00	0.00
ENSSSCG00000012133	ASB11	5.23	5.81	1.40	4.16	0.00	0.00
ENSSSCG00000039677	CHRNA4	10.53	5.83	1.29	4.52	0.00	0.00
ENSSSCG00000035053		49.51	5.85	0.69	8.51	0.00	0.00

ENSSSCG00000024552		5.37	5.85	1.38	4.23	0.00	0.00
ENSSSCG00000017389	RAMP2	5.39	5.85	1.39	4.22	0.00	0.00
ENSSSCG00000000188	DHH	19.90	5.86	1.09	5.39	0.00	0.00
ENSSSCG00000037735		70.16	5.86	0.58	10.05	0.00	0.00
ENSSSCG00000000216	ASIC1	10.77	5.87	1.29	4.56	0.00	0.00
ENSSSCG00000029875	ZNF461	10.80	5.87	1.28	4.59	0.00	0.00
ENSSSCG00000001560	C6orf222	70.68	5.88	0.58	10.09	0.00	0.00
ENSSSCG00000021222		10.92	5.89	1.28	4.60	0.00	0.00
ENSSSCG00000031581		5.54	5.89	1.37	4.30	0.00	0.00
ENSSSCG00000001636		5.54	5.89	1.37	4.29	0.00	0.00
ENSSSCG00000013667	COL5A3	5.54	5.89	1.38	4.27	0.00	0.00
ENSSSCG00000005438	ACTL7B	5.55	5.90	1.37	4.30	0.00	0.00
ENSSSCG00000013530		5.56	5.90	1.38	4.29	0.00	0.00
ENSSSCG00000035150		5.60	5.90	1.38	4.28	0.00	0.00
ENSSSCG00000017978	ALOX15B	11.13	5.91	1.28	4.61	0.00	0.00
ENSSSCG00000008347	FBXO48	250.19	5.92	0.32	18.78	0.00	0.00
ENSSSCG00000017387	WNK4	41.87	5.93	0.77	7.75	0.00	0.00
ENSSSCG00000007574	SDK1	5.69	5.93	1.37	4.32	0.00	0.00
ENSSSCG00000017488	CSF3	347.02	5.94	0.29	20.53	0.00	0.00
ENSSSCG00000027124		11.30	5.94	1.27	4.66	0.00	0.00
ENSSSCG00000029189	DCHS1	5.74	5.94	1.37	4.34	0.00	0.00
ENSSSCG00000017988	CCDC42	5.83	5.97	1.37	4.35	0.00	0.00
ENSSSCG00000039186	RAB25	5.85	5.97	1.37	4.36	0.00	0.00
ENSSSCG00000003788	PTGER3	5.88	5.98	1.36	4.40	0.00	0.00
ENSSSCG00000032431	TMEM52B	5.88	5.98	1.36	4.40	0.00	0.00

ENSSSCG00000017444	KRT15	5.91	5.98	1.37	4.36	0.00	0.00
ENSSSCG00000013989	GJC2	21.77	5.99	1.08	5.54	0.00	0.00
ENSSSCG00000017068	FAXDC2	98.81	6.00	0.51	11.77	0.00	0.00
ENSSSCG00000008832	LRRC66	6.02	6.01	1.36	4.43	0.00	0.00
ENSSSCG00000003147	CA11	22.21	6.02	1.08	5.58	0.00	0.00
ENSSSCG00000008634	ATP6V1C2	12.11	6.04	1.27	4.76	0.00	0.00
ENSSSCG00000022163	GNAL	12.16	6.04	1.27	4.75	0.00	0.00
ENSSSCG00000014996		6.17	6.05	1.36	4.45	0.00	0.00
ENSSSCG00000034821	ARMCX4	6.17	6.05	1.36	4.46	0.00	0.00
ENSSSCG00000010304	MYOZ1	6.19	6.05	1.35	4.47	0.00	0.00
ENSSSCG00000011307		6.35	6.09	1.35	4.50	0.00	0.00
ENSSSCG00000037852		6.37	6.09	1.35	4.53	0.00	0.00
ENSSSCG00000040036		6.37	6.09	1.36	4.47	0.00	0.00
ENSSSCG00000010322	ZNF503	12.73	6.11	1.26	4.83	0.00	0.00
ENSSSCG00000028875	HAVCR2	47.42	6.11	0.76	8.02	0.00	0.00
ENSSSCG00000013617	ELAVL3	6.50	6.12	1.37	4.48	0.00	0.00
ENSSSCG00000005742	DBH	24.04	6.13	1.08	5.65	0.00	0.00
ENSSSCG00000039847	C1S	48.04	6.13	0.77	8.00	0.00	0.00
ENSSSCG00000017372	MPP3	6.59	6.14	1.36	4.53	0.00	0.00
ENSSSCG00000006359	ADAMTS4	60.79	6.15	0.68	9.02	0.00	0.00
ENSSSCG00000001613	TREML1	6.65	6.16	1.36	4.53	0.00	0.00
ENSSSCG00000031616	FOSB	196.22	6.16	0.38	16.04	0.00	0.00
ENSSSCG00000024233	RAB39B	13.20	6.17	1.26	4.89	0.00	0.00
ENSSSCG00000040725	IL11	62.13	6.18	0.69	8.97	0.00	0.00
ENSSSCG00000033085		13.38	6.18	1.28	4.84	0.00	0.00

ENSSSCG00000003062	LYPD3	13.37	6.18	1.26	4.90	0.00	0.00
ENSSSCG00000020970	IL6	311.49	6.19	0.31	20.00	0.00	0.00
ENSSSCG00000017507		50.26	6.20	0.76	8.12	0.00	0.00
ENSSSCG00000006788	ADORA3	13.61	6.21	1.26	4.91	0.00	0.00
ENSSSCG00000032405	TMEM190	13.74	6.22	1.26	4.94	0.00	0.00
ENSSSCG00000022692	RINL	13.76	6.22	1.26	4.95	0.00	0.00
ENSSSCG00000039190		6.97	6.23	1.35	4.62	0.00	0.00
ENSSSCG00000006808	SLC16A4	13.89	6.24	1.26	4.96	0.00	0.00
ENSSSCG00000031111	CBR1	7.06	6.24	1.35	4.62	0.00	0.00
ENSSSCG00000033245	GLYATL3	7.12	6.26	1.34	4.66	0.00	0.00
ENSSSCG00000015119	MFRP	14.24	6.27	1.26	4.98	0.00	0.00
ENSSSCG00000006455		26.55	6.28	1.08	5.83	0.00	0.00
ENSSSCG00000035592	GPR55	7.31	6.29	1.33	4.72	0.00	0.00
ENSSSCG00000015433		7.34	6.30	1.33	4.75	0.00	0.00
ENSSSCG00000007163	AVP	7.35	6.30	1.33	4.73	0.00	0.00
ENSSSCG00000002483		7.36	6.30	1.33	4.75	0.00	0.00
ENSSSCG00000035724		14.50	6.30	1.25	5.02	0.00	0.00
ENSSSCG00000039661	SSPO	7.42	6.31	1.33	4.73	0.00	0.00
ENSSSCG00000040057	EOMES	7.42	6.31	1.34	4.72	0.00	0.00
ENSSSCG00000039807		41.23	6.33	0.87	7.25	0.00	0.00
ENSSSCG00000035612	COX6B2	7.54	6.34	1.33	4.77	0.00	0.00
ENSSSCG00000006704		14.92	6.34	1.25	5.06	0.00	0.00
ENSSSCG00000004597	AQP9	7.65	6.36	1.32	4.81	0.00	0.00
ENSSSCG00000040720	SLC26A9	28.23	6.37	1.07	5.96	0.00	0.00
ENSSSCG00000037892	YPEL4	15.19	6.37	1.25	5.09	0.00	0.00

ENSSSCG00000001764	SH2D7	7.73	6.37	1.32	4.81	0.00	0.00
ENSSSCG00000023591	ADGRF2	86.24	6.39	0.62	10.30	0.00	0.00
ENSSSCG00000037487		7.87	6.40	1.32	4.85	0.00	0.00
ENSSSCG00000007607	KPNA7	7.87	6.40	1.32	4.85	0.00	0.00
ENSSSCG00000038013	DIRAS1	15.61	6.41	1.25	5.12	0.00	0.00
ENSSSCG00000021511	GCSAM	8.01	6.42	1.33	4.84	0.00	0.00
ENSSSCG00000028331	IL1R2	8.04	6.43	1.33	4.82	0.00	0.00
ENSSSCG00000036060	RRAD	15.94	6.44	1.25	5.15	0.00	0.00
ENSSSCG00000013839	RASAL3	16.00	6.45	1.25	5.15	0.00	0.00
ENSSSCG00000012490	TMEM35A	8.12	6.45	1.32	4.90	0.00	0.00
ENSSSCG00000035906	SLC13A3	8.16	6.45	1.31	4.92	0.00	0.00
ENSSSCG00000009642	STC1	8.24	6.46	1.32	4.90	0.00	0.00
ENSSSCG00000039921		30.18	6.46	1.07	6.06	0.00	0.00
ENSSSCG00000039442	BMP2	90.60	6.46	0.61	10.52	0.00	0.00
ENSSSCG00000033590		8.29	6.48	1.31	4.93	0.00	0.00
ENSSSCG00000000688	LAG3	30.54	6.48	1.07	6.08	0.00	0.00
ENSSSCG00000031092		8.39	6.49	1.32	4.92	0.00	0.00
ENSSSCG00000000892	HAL	30.74	6.49	1.07	6.09	0.00	0.00
ENSSSCG00000013420	KISS1R	16.77	6.52	1.25	5.23	0.00	0.00
ENSSSCG00000039500		156.80	6.52	0.48	13.69	0.00	0.00
ENSSSCG00000001751	CHRNA5	8.65	6.54	1.31	5.00	0.00	0.00
ENSSSCG00000017705	CCL5	238.32	6.54	0.40	16.30	0.00	0.00
ENSSSCG00000000983		8.71	6.54	1.31	5.00	0.00	0.00
ENSSSCG00000000162	BTBD11	17.26	6.55	1.25	5.26	0.00	0.00
ENSSSCG00000017462	KRT27	8.78	6.56	1.32	4.97	0.00	0.00

ENSSSCG00000008603	MATN3	8.82	6.56	1.30	5.04	0.00	0.00
ENSSSCG00000021651	SCN2B	8.83	6.56	1.30	5.04	0.00	0.00
ENSSSCG00000027684	TRIM63	17.60	6.58	1.25	5.25	0.00	0.00
ENSSSCG00000032257		9.10	6.61	1.31	5.05	0.00	0.00
ENSSSCG00000034812	EFCAB8	33.47	6.61	1.06	6.24	0.00	0.00
ENSSSCG00000011107	CCNY	9.15	6.62	1.30	5.10	0.00	0.00
ENSSSCG00000027465	NLGN3	33.47	6.62	1.06	6.24	0.00	0.00
ENSSSCG00000008731	OTOP1	9.19	6.62	1.30	5.09	0.00	0.00
ENSSSCG00000001403	LTA	9.22	6.63	1.32	5.02	0.00	0.00
ENSSSCG00000031114	SLFN14	9.25	6.63	1.30	5.09	0.00	0.00
ENSSSCG00000033192		9.31	6.64	1.30	5.10	0.00	0.00
ENSSSCG00000006754	AMPD1	51.99	6.67	0.86	7.72	0.00	0.00
ENSSSCG00000022014	DCDC2B	18.84	6.68	1.24	5.40	0.00	0.00
ENSSSCG00000017762	FOXN1	9.60	6.69	1.29	5.17	0.00	0.00
ENSSSCG00000015923	NOSTRIN	9.63	6.69	1.30	5.14	0.00	0.00
ENSSSCG00000017377		9.91	6.73	1.30	5.19	0.00	0.00
ENSSSCG00000001550	ARMC12	9.92	6.73	1.29	5.22	0.00	0.00
ENSSSCG00000015579	PTGS2	276.37	6.75	0.39	17.31	0.00	0.00
ENSSSCG00000010100	LRRC74B	10.15	6.76	1.29	5.26	0.00	0.00
ENSSSCG00000028218	CEACAM20	37.16	6.77	1.06	6.40	0.00	0.00
ENSSSCG00000032149	PLET1	94.62	6.79	0.67	10.10	0.00	0.00
ENSSSCG00000038606		132.76	6.80	0.56	12.03	0.00	0.00
ENSSSCG00000033909		135.09	6.82	0.57	11.96	0.00	0.00
ENSSSCG00000017862	TRPV3	96.86	6.83	0.67	10.18	0.00	0.00
ENSSSCG00000011401	LSMEM2	11.12	6.90	1.28	5.40	0.00	0.00

ENSSSCG00000029635	GRIN3B	21.88	6.90	1.23	5.62	0.00	0.00
ENSSSCG00000005385	NR4A3	269.54	6.93	0.42	16.41	0.00	0.00
ENSSSCG00000028923	SCNN1B	22.32	6.93	1.23	5.62	0.00	0.00
ENSSSCG00000002140		148.08	6.96	0.56	12.36	0.00	0.00
ENSSSCG00000013665		11.61	6.96	1.27	5.47	0.00	0.00
ENSSSCG00000025500		11.71	6.97	1.28	5.46	0.00	0.00
ENSSSCG00000028411	MC5R	23.21	6.99	1.23	5.69	0.00	0.00
ENSSSCG00000012986	KCNK7	23.57	7.01	1.23	5.71	0.00	0.00
ENSSSCG00000039300	IL27	12.04	7.01	1.27	5.52	0.00	0.00
ENSSSCG00000011561	PRRT3	23.82	7.02	1.23	5.72	0.00	0.00
ENSSSCG00000034639	MGAM	12.43	7.06	1.27	5.57	0.00	0.00
ENSSSCG00000026043		68.61	7.07	0.87	8.13	0.00	0.00
ENSSSCG00000033542		12.61	7.08	1.29	5.49	0.00	0.00
ENSSSCG00000013401	DKK3	24.85	7.09	1.23	5.78	0.00	0.00
ENSSSCG00000036854		94.00	7.11	0.74	9.56	0.00	0.00
ENSSSCG00000036064	CALHM6	25.84	7.14	1.22	5.84	0.00	0.00
ENSSSCG00000006551	AQP10	13.57	7.18	1.26	5.70	0.00	0.00
ENSSSCG00000001749	IL17F	13.73	7.20	1.26	5.72	0.00	0.00
ENSSSCG00000006378	SLAMF7	13.75	7.20	1.26	5.72	0.00	0.00
ENSSSCG00000013890	SLC5A5	14.12	7.24	1.26	5.74	0.00	0.00
ENSSSCG00000032131	NCAN	14.24	7.25	1.26	5.77	0.00	0.00
ENSSSCG00000039986	RGS8	14.25	7.25	1.27	5.72	0.00	0.00
ENSSSCG00000029849	S1PR1	28.40	7.28	1.22	5.98	0.00	0.00
ENSSSCG00000015085	IL10RA	479.05	7.29	0.35	20.80	0.00	0.00
ENSSSCG00000000647	OLR1	29.42	7.33	1.23	5.94	0.00	0.00

ENSSSCG00000015596	FAM71A	15.06	7.33	1.25	5.86	0.00	0.00
ENSSSCG00000033520	IL23A	55.08	7.34	1.05	6.99	0.00	0.00
ENSSSCG00000000184	DDN	15.19	7.35	1.25	5.86	0.00	0.00
ENSSSCG00000001404	TNF	86.43	7.40	0.85	8.68	0.00	0.00
ENSSSCG00000017863	TRPV1	57.78	7.41	1.05	7.07	0.00	0.00
ENSSSCG00000011286	KLHL40	16.20	7.44	1.25	5.96	0.00	0.00
ENSSSCG00000031708		889.26	7.45	0.28	27.02	0.00	0.00
ENSSSCG00000006792		16.40	7.46	1.25	5.96	0.00	0.00
ENSSSCG00000003231	NKG7	32.89	7.49	1.22	6.14	0.00	0.00
ENSSSCG00000000195	PRPH	34.04	7.54	1.21	6.22	0.00	0.00
ENSSSCG00000030371		17.74	7.57	1.24	6.09	0.00	0.00
ENSSSCG00000011862	MUC13	130.44	7.58	0.74	10.26	0.00	0.00
ENSSSCG00000034802		17.98	7.59	1.24	6.11	0.00	0.00
ENSSSCG00000003088	APOE	18.49	7.63	1.24	6.16	0.00	0.00
ENSSSCG00000009444	OLFM4	39.25	7.75	1.21	6.42	0.00	0.00
ENSSSCG00000017770	PROCA1	39.25	7.75	1.21	6.42	0.00	0.00
ENSSSCG00000036146	FGF8	20.45	7.78	1.24	6.28	0.00	0.00
ENSSSCG00000022849	IL2RA	20.58	7.79	1.24	6.30	0.00	0.00
ENSSSCG00000001252		114.40	7.81	0.85	9.19	0.00	0.00
ENSSSCG00000003081	CEACAM16	21.81	7.87	1.23	6.39	0.00	0.00
ENSSSCG00000023607	CYP4F22	42.85	7.88	1.21	6.51	0.00	0.00
ENSSSCG00000029827	IQCF1	23.66	7.99	1.23	6.51	0.00	0.00
ENSSSCG00000006666	SV2A	24.11	8.01	1.23	6.53	0.00	0.00
ENSSSCG00000008953	CXCL8	8793.06	8.03	0.17	47.16	0.00	0.00
ENSSSCG00000016254	CCL20	3076.65	8.06	0.19	42.39	0.00	0.00

ENSSSCG00000017723	CCL2	227.45	8.07	0.66	12.22	0.00	0.00
ENSSSCG00000007757	TRIM72	51.68	8.15	1.20	6.79	0.00	0.00
ENSSSCG00000002919	TYROBP	26.92	8.17	1.22	6.69	0.00	0.00
ENSSSCG00000022614	DQX1	27.06	8.18	1.22	6.70	0.00	0.00
ENSSSCG00000006379	CD48	28.53	8.26	1.22	6.75	0.00	0.00
ENSSSCG00000034568	RNASE4	57.61	8.31	1.20	6.92	0.00	0.00
ENSSSCG00000017720	CCL1	30.59	8.36	1.22	6.87	0.00	0.00
ENSSSCG00000011657	CLDN18	31.36	8.39	1.21	6.91	0.00	0.00
ENSSSCG00000031653	DOK3	31.77	8.41	1.22	6.91	0.00	0.00
ENSSSCG00000003599		34.39	8.53	1.22	6.97	0.00	0.00
ENSSSCG00000013653	ICAM5	35.12	8.56	1.21	7.07	0.00	0.00
ENSSSCG00000003616	FAM167B	36.20	8.60	1.21	7.09	0.00	0.00
ENSSSCG00000034087	TNFSF15	212.27	8.71	0.84	10.35	0.00	0.00
ENSSSCG00000022675	NCR1	77.72	8.74	1.19	7.31	0.00	0.00
ENSSSCG00000013976		79.30	8.77	1.20	7.31	0.00	0.00
ENSSSCG00000015654	IL20	46.31	8.96	1.21	7.40	0.00	0.00
ENSSSCG00000014985	MMP3	47.37	8.99	1.21	7.44	0.00	0.00
ENSSSCG00000031898	CALHM5	48.84	9.03	1.20	7.51	0.00	0.00
ENSSSCG00000022089	ADGRF4	56.63	9.25	1.20	7.71	0.00	0.00
ENSSSCG00000032474	CXCL10	138.21	9.57	1.19	8.06	0.00	0.00
ENSSSCG00000025560	PGLYRP2	155.00	9.74	1.19	8.21	0.00	0.00
ENSSSCG00000017700	CCL3L1	245.29	10.40	1.19	8.74	0.00	0.00
ENSSSCG00000004890	SERPINB2	226.69	11.25	1.19	9.49	0.00	0.00

Table S9. Differential gene expression analysis between AFB_1-treated PK-15 cells (WT) and AFB_1-treated BACH1-KO cells by

RNA-Seq.

Gene names	Gene ID	baseMean	log2FoldChange	lfcSE	stat	pvalue	padj
ENSSSCG00000024067	KRT23	311.29	-9.89	1.02	-9.65	0.00	0.00
ENSSSCG00000008930	TMPRSS11F	70.16	-9.60	1.20	-8.03	0.00	0.00
ENSSSCG00000015320	CALCR	38.56	-7.77	1.21	-6.41	0.00	0.00
ENSSSCG00000028810		19.10	-7.72	1.23	-6.26	0.00	0.00
ENSSSCG00000007807	CD19	32.26	-7.51	1.21	-6.18	0.00	0.00
ENSSSCG00000017723	CCL2	234.35	-7.31	0.50	-14.61	0.00	0.00
ENSSSCG00000000240	KRT85	13.87	-7.26	1.26	-5.77	0.00	0.00
ENSSSCG00000032676		13.56	-7.23	1.26	-5.74	0.00	0.00
ENSSSCG00000014985	MMP3	48.99	-7.21	1.05	-6.86	0.00	0.00
ENSSSCG00000038598	ADRB2	12.71	-7.13	1.26	-5.65	0.00	0.00
ENSSSCG00000008929		21.78	-6.94	1.23	-5.66	0.00	0.00
ENSSSCG00000008809	GABRG1	21.41	-6.91	1.23	-5.63	0.00	0.00
ENSSSCG00000034731		85.06	-6.68	0.67	-9.97	0.00	0.00
ENSSSCG00000034838	MAP1LC3C	9.31	-6.68	1.30	-5.15	0.00	0.00
ENSSSCG00000014789		9.06	-6.64	1.29	-5.13	0.00	0.00
ENSSSCG00000017462	KRT27	9.02	-6.64	1.31	-5.08	0.00	0.00
ENSSSCG00000014930		8.71	-6.59	1.30	-5.06	0.00	0.00
ENSSSCG00000017720	CCL1	31.77	-6.58	1.06	-6.22	0.00	0.00
ENSSSCG00000034113		8.23	-6.51	1.30	-5.00	0.00	0.00
ENSSSCG00000027093	FOLH1B	8.04	-6.47	1.31	-4.95	0.00	0.00
ENSSSCG00000004597	AQP9	7.87	-6.44	1.31	-4.92	0.00	0.00
ENSSSCG00000002479	SERPINA11	15.36	-6.43	1.25	-5.14	0.00	0.00

ENSSSCG00000006867	LRRC39	7.51	-6.38	1.32	-4.82	0.00	0.00
ENSSSCG00000006808	SLC16A4	14.29	-6.32	1.25	-5.06	0.00	0.00
ENSSSCG00000014924	CTSC	971.00	-6.29	0.18	-35.19	0.00	0.00
ENSSSCG00000033085		13.71	-6.26	1.27	-4.93	0.00	0.00
ENSSSCG00000006666	SV2A	25.10	-6.25	1.07	-5.83	0.00	0.00
ENSSSCG00000003011		36.82	-6.21	0.91	-6.84	0.00	0.00
ENSSSCG00000011307		6.53	-6.17	1.34	-4.61	0.00	0.00
ENSSSCG00000032108		6.44	-6.15	1.35	-4.56	0.00	0.00
ENSSSCG00000014996		6.36	-6.13	1.35	-4.56	0.00	0.00
ENSSSCG00000015986	HOXD1	6.34	-6.13	1.35	-4.53	0.00	0.00
ENSSSCG00000039186	RAB25	6.03	-6.06	1.35	-4.48	0.00	0.00
ENSSSCG00000039927		11.73	-6.03	1.27	-4.76	0.00	0.00
ENSSSCG00000000943	OTOGL	5.86	-6.02	1.36	-4.42	0.00	0.00
ENSSSCG00000011047	FAM171A1	5.76	-5.99	1.36	-4.39	0.00	0.00
ENSSSCG00000005475		5.35	-5.89	1.37	-4.31	0.00	0.00
ENSSSCG00000000248		18.99	-5.84	1.08	-5.38	0.00	0.00
ENSSSCG00000017441		5.16	-5.84	1.38	-4.23	0.00	0.00
ENSSSCG00000000636		5.16	-5.83	1.39	-4.21	0.00	0.00
ENSSSCG00000013733	BEST2	10.06	-5.81	1.28	-4.54	0.00	0.00
ENSSSCG00000032023		5.02	-5.79	1.38	-4.19	0.00	0.00
ENSSSCG00000031712	MFAP5	9.76	-5.76	1.28	-4.49	0.00	0.00
ENSSSCG00000039569		4.89	-5.75	1.39	-4.14	0.00	0.00
ENSSSCG00000039407		4.86	-5.75	1.38	-4.15	0.00	0.00
ENSSSCG00000032257		9.51	-5.73	1.29	-4.43	0.00	0.00
ENSSSCG00000010659	PNLIPRP1	4.72	-5.70	1.39	-4.10	0.00	0.00

ENSSSCG00000040680		4.70	-5.70	1.40	-4.06	0.00	0.00
ENSSSCG00000035334		4.54	-5.65	1.41	-4.01	0.00	0.00
ENSSSCG00000031653	DOK3	33.34	-5.65	0.78	-7.25	0.00	0.00
ENSSSCG00000006335	RGS4	4.51	-5.64	1.40	-4.02	0.00	0.00
ENSSSCG00000003077		8.92	-5.63	1.29	-4.35	0.00	0.00
ENSSSCG00000021866	EDDM3B	4.25	-5.55	1.43	-3.87	0.00	0.00
ENSSSCG00000037532	MNDA	4.24	-5.54	1.42	-3.90	0.00	0.00
ENSSSCG00000021511	GCSAM	8.38	-5.54	1.31	-4.23	0.00	0.00
ENSSSCG00000008054	NTN3	15.25	-5.51	1.10	-5.01	0.00	0.00
ENSSSCG00000032131	NCAN	14.99	-5.48	1.10	-4.98	0.00	0.00
ENSSSCG00000009662	STMN4	4.01	-5.47	1.43	-3.83	0.00	0.00
ENSSSCG00000035612	COX6B2	7.90	-5.45	1.31	-4.16	0.00	0.00
ENSSSCG00000035592	GPR55	7.67	-5.41	1.32	-4.12	0.00	0.00
ENSSSCG00000017700	CCL3L1	257.65	-5.41	0.39	-13.94	0.00	0.00
ENSSSCG00000013932	LPAR2	14.14	-5.40	1.10	-4.89	0.00	0.00
ENSSSCG00000028875	HAVCR2	49.27	-5.39	0.59	-9.09	0.00	0.00
ENSSSCG00000003314	NLRP11	3.71	-5.36	1.44	-3.71	0.00	0.00
ENSSSCG00000001844	PLIN1	3.69	-5.35	1.44	-3.70	0.00	0.00
ENSSSCG00000021696	NRSN1	3.67	-5.34	1.46	-3.66	0.00	0.00
ENSSSCG00000022675	NCR1	81.65	-5.34	0.46	-11.69	0.00	0.00
ENSSSCG00000014964		3.67	-5.34	1.45	-3.67	0.00	0.00
ENSSSCG00000037569		3.66	-5.34	1.46	-3.67	0.00	0.00
ENSSSCG00000015425	SLC26A5	7.24	-5.32	1.32	-4.04	0.00	0.00
ENSSSCG00000014931		33.53	-5.32	0.70	-7.56	0.00	0.00
ENSSSCG00000039203	CYSLTR1	3.55	-5.29	1.48	-3.59	0.00	0.00

ENSSSCG00000005707	FIBCD1	19.58	-5.28	0.91	-5.81	0.00	0.00
ENSSSCG00000001736	CRISP2	3.51	-5.28	1.50	-3.51	0.00	0.00
ENSSSCG00000000688	LAG3	31.91	-5.25	0.71	-7.42	0.00	0.00
ENSSSCG00000032321	MGAT4C	6.85	-5.25	1.34	-3.91	0.00	0.00
ENSSSCG00000003794	RPE65	3.39	-5.22	1.49	-3.51	0.00	0.00
ENSSSCG00000005312	SIT1	3.38	-5.22	1.49	-3.51	0.00	0.00
ENSSSCG00000029827	IQCF1	25.01	-5.21	0.79	-6.61	0.00	0.00
ENSSSCG00000010837	FAM177B	6.70	-5.21	1.33	-3.92	0.00	0.00
ENSSSCG00000039978		3.34	-5.21	1.48	-3.52	0.00	0.00
ENSSSCG00000038114		3.33	-5.20	1.50	-3.47	0.00	0.00
ENSSSCG00000034015		60.28	-5.16	0.50	-10.28	0.00	0.00
ENSSSCG00000032416	RGS1	3.21	-5.14	1.49	-3.46	0.00	0.00
ENSSSCG00000014186		3.20	-5.14	1.49	-3.46	0.00	0.00
ENSSSCG00000011208	ZNF385D	3.20	-5.14	1.48	-3.47	0.00	0.00
ENSSSCG00000007384		6.41	-5.14	1.33	-3.85	0.00	0.00
ENSSSCG00000002983	LGALS13	3.18	-5.13	1.48	-3.47	0.00	0.00
ENSSSCG00000006792		17.38	-5.11	0.92	-5.53	0.00	0.00
ENSSSCG00000003788	PTGER3	6.23	-5.10	1.34	-3.81	0.00	0.00
ENSSSCG00000004890	SERPINB2	240.02	-5.08	0.26	-19.87	0.00	0.00
ENSSSCG00000034174	AURKC	3.04	-5.07	1.50	-3.38	0.00	0.00
ENSSSCG00000025188	LEPR	3.01	-5.06	1.50	-3.37	0.00	0.00
ENSSSCG00000006329	LMX1A	2.99	-5.05	1.51	-3.34	0.00	0.00
ENSSSCG00000003482	PADI3	39.20	-5.05	0.60	-8.36	0.00	0.00
ENSSSCG00000003399	RBP7	2.99	-5.05	1.52	-3.32	0.00	0.00
ENSSSCG00000035150		5.91	-5.02	1.36	-3.71	0.00	0.00

ENSSSCG00000039973	TAS1R2	5.90	-5.02	1.35	-3.73	0.00	0.00
ENSSSCG00000031581		5.86	-5.01	1.35	-3.72	0.00	0.00
ENSSSCG00000038588		5.82	-5.01	1.38	-3.63	0.00	0.00
ENSSSCG00000015200	ESAM	21.51	-4.99	0.80	-6.22	0.00	0.00
ENSSSCG00000026512		2.88	-4.99	1.52	-3.28	0.00	0.00
ENSSSCG00000017305	EFCAB13	2.87	-4.98	1.52	-3.27	0.00	0.00
ENSSSCG00000002007	FITM1	5.70	-4.97	1.36	-3.67	0.00	0.00
ENSSSCG00000009936		2.84	-4.97	1.52	-3.28	0.00	0.00
ENSSSCG00000012760		2.84	-4.97	1.53	-3.24	0.00	0.00
ENSSSCG00000025416	CAMKV	2.82	-4.97	1.54	-3.22	0.00	0.00
ENSSSCG00000012705	ZIC3	2.82	-4.96	1.55	-3.20	0.00	0.00
ENSSSCG00000036331		15.64	-4.94	0.93	-5.33	0.00	0.00
ENSSSCG00000000418	TAC3	5.52	-4.93	1.36	-3.63	0.00	0.00
ENSSSCG00000015223	DDX25	10.24	-4.92	1.14	-4.30	0.00	0.00
ENSSSCG00000006350		132.75	-4.92	0.32	-15.51	0.00	0.00
ENSSSCG00000032474	CXCL10	146.68	-4.90	0.30	-16.10	0.00	0.00
ENSSSCG00000035429	HJV	2.70	-4.90	1.56	-3.15	0.00	0.00
ENSSSCG00000015617	G0S2	2.68	-4.89	1.53	-3.19	0.00	0.00
ENSSSCG00000023841	RF00006	2.68	-4.89	1.53	-3.19	0.00	0.00
ENSSSCG00000031804		2.68	-4.89	1.53	-3.18	0.00	0.00
ENSSSCG00000022089	ADGRF4	60.20	-4.89	0.47	-10.44	0.00	0.00
ENSSSCG00000031311		2.66	-4.88	1.55	-3.14	0.00	0.00
ENSSSCG00000006286		5.34	-4.88	1.37	-3.56	0.00	0.00
ENSSSCG00000033192		9.87	-4.87	1.14	-4.26	0.00	0.00
ENSSSCG00000008731	OTOP1	9.75	-4.85	1.14	-4.25	0.00	0.00

ENSSSCG00000006378	SLAMF7	14.60	-4.85	0.93	-5.20	0.00	0.00
ENSSSCG00000003980	EXO5	5.27	-4.85	1.38	-3.52	0.00	0.00
ENSSSCG00000004290	TBX18	5.26	-4.84	1.38	-3.52	0.00	0.00
ENSSSCG00000013031	KCNK4	5.19	-4.84	1.37	-3.54	0.00	0.00
ENSSSCG00000028522	KRT10	33.28	-4.81	0.62	-7.79	0.00	0.00
ENSSSCG00000015049	TMPRSS5	2.53	-4.80	1.55	-3.09	0.00	0.00
ENSSSCG00000017458	KRT39	2.53	-4.80	1.56	-3.08	0.00	0.00
ENSSSCG00000032975		2.53	-4.80	1.56	-3.07	0.00	0.00
ENSSSCG00000009105		2.52	-4.80	1.56	-3.07	0.00	0.00
ENSSSCG00000031003		2.51	-4.79	1.55	-3.09	0.00	0.00
ENSSSCG00000002709		9.39	-4.79	1.15	-4.18	0.00	0.00
ENSSSCG00000015199	VSIG2	2.51	-4.79	1.60	-2.99	0.00	0.00
ENSSSCG00000003998	ZNF274	2.51	-4.79	1.56	-3.08	0.00	0.00
ENSSSCG00000015661	C4BPB	5.04	-4.78	1.37	-3.49	0.00	0.00
ENSSSCG00000037632		2.48	-4.78	1.61	-2.97	0.00	0.00
ENSSSCG00000017500	TCAP	5.00	-4.78	1.38	-3.47	0.00	0.00
ENSSSCG00000006555	C1orf189	14.06	-4.78	0.94	-5.09	0.00	0.00
ENSSSCG00000023151	GRIN2C	9.15	-4.76	1.17	-4.09	0.00	0.00
ENSSSCG00000014235	SNCAIP	17.91	-4.73	0.81	-5.81	0.00	0.00
ENSSSCG00000017012	SLIT3	13.36	-4.72	0.96	-4.91	0.00	0.00
ENSSSCG00000015799	KLKB1	2.39	-4.72	1.63	-2.89	0.00	0.01
ENSSSCG00000006736	CD2	2.36	-4.70	1.60	-2.94	0.00	0.01
ENSSSCG00000013513	PLIN5	2.34	-4.69	1.58	-2.98	0.00	0.00
ENSSSCG00000017387	WNK4	44.00	-4.69	0.52	-9.11	0.00	0.00
ENSSSCG00000039008		2.33	-4.69	1.60	-2.93	0.00	0.01

ENSSSCG00000034878		2.33	-4.69	1.60	-2.93	0.00	0.01
ENSSSCG00000023396	LIX1	17.12	-4.66	0.83	-5.64	0.00	0.00
ENSSSCG00000007424	TNNC2	4.62	-4.65	1.44	-3.22	0.00	0.00
ENSSSCG00000007961		4.54	-4.63	1.39	-3.33	0.00	0.00
ENSSSCG00000013469	ZNF555	4.52	-4.63	1.39	-3.32	0.00	0.00
ENSSSCG00000036080	HIST1H2AB	2.21	-4.60	1.62	-2.85	0.00	0.01
ENSSSCG00000013559		2.21	-4.60	1.65	-2.80	0.01	0.01
ENSSSCG00000038892		2.20	-4.60	1.62	-2.84	0.00	0.01
ENSSSCG00000038419		98.54	-4.60	0.34	-13.49	0.00	0.00
ENSSSCG00000035531		2.19	-4.59	1.60	-2.87	0.00	0.01
ENSSSCG00000034284		2.18	-4.59	1.60	-2.87	0.00	0.01
ENSSSCG00000008978	CXCL11	2.17	-4.59	1.61	-2.85	0.00	0.01
ENSSSCG00000036400		2.16	-4.58	1.63	-2.81	0.00	0.01
ENSSSCG00000000577	GYS2	2.15	-4.58	1.65	-2.77	0.01	0.01
ENSSSCG00000001705	TCTE1	4.38	-4.57	1.40	-3.27	0.00	0.00
ENSSSCG00000007495	CASS4	12.02	-4.57	0.96	-4.75	0.00	0.00
ENSSSCG00000013503	SHD	4.22	-4.52	1.41	-3.21	0.00	0.00
ENSSSCG00000009530		46.84	-4.51	0.48	-9.37	0.00	0.00
ENSSSCG00000035013		11.62	-4.51	0.96	-4.69	0.00	0.00
ENSSSCG00000034794		4.17	-4.51	1.42	-3.18	0.00	0.00
ENSSSCG00000017384	AOC2	190.80	-4.51	0.41	-10.90	0.00	0.00
ENSSSCG00000034326		2.06	-4.50	1.67	-2.69	0.01	0.01
ENSSSCG00000013989	GJC2	23.02	-4.49	0.68	-6.62	0.00	0.00
ENSSSCG00000013887	INSL3	2.03	-4.48	1.63	-2.74	0.01	0.01
ENSSSCG00000033878	RAB38	2.02	-4.48	1.68	-2.67	0.01	0.01

ENSSSCG00000007116	CD93	1.99	-4.46	1.66	-2.69	0.01	0.01
ENSSSCG00000007973	IL9R	1.99	-4.46	1.68	-2.65	0.01	0.01
ENSSSCG00000007486	CYP24A1	4.03	-4.45	1.42	-3.14	0.00	0.00
ENSSSCG00000016900	ESM1	41.15	-4.45	0.50	-8.83	0.00	0.00
ENSSSCG00000027684	TRIM63	18.69	-4.45	0.76	-5.86	0.00	0.00
ENSSSCG00000038215		4.04	-4.45	1.42	-3.14	0.00	0.00
ENSSSCG00000008159	IL1RL1	29.33	-4.43	0.59	-7.48	0.00	0.00
ENSSSCG00000036146	FGF8	22.01	-4.43	0.69	-6.44	0.00	0.00
ENSSSCG00000003135	KCNJ14	121.08	-4.43	0.30	-14.80	0.00	0.00
ENSSSCG00000001749	IL17F	14.80	-4.43	0.83	-5.34	0.00	0.00
ENSSSCG00000031122	HIST1H2BB	11.02	-4.42	0.98	-4.51	0.00	0.00
ENSSSCG00000014072	ENC1	858.22	-4.42	0.12	-35.65	0.00	0.00
ENSSSCG00000022614	DQX1	29.16	-4.41	0.60	-7.40	0.00	0.00
ENSSSCG00000026043		73.26	-4.41	0.40	-10.99	0.00	0.00
ENSSSCG00000024809	CLCN4	3.91	-4.41	1.46	-3.02	0.00	0.00
ENSSSCG00000038149	KCNE4	3.88	-4.40	1.45	-3.04	0.00	0.00
ENSSSCG00000012531		3.86	-4.39	1.43	-3.08	0.00	0.00
ENSSSCG00000029545	GPRC5D	3.89	-4.39	1.43	-3.07	0.00	0.00
ENSSSCG00000002140		158.24	-4.39	0.25	-17.32	0.00	0.00
ENSSSCG00000000080	GRAP2	3.84	-4.38	1.44	-3.04	0.00	0.00
ENSSSCG00000023868		3.83	-4.38	1.45	-3.03	0.00	0.00
ENSSSCG00000001097		60.50	-4.38	0.42	-10.33	0.00	0.00
ENSSSCG00000004207		1.86	-4.36	1.68	-2.60	0.01	0.01
ENSSSCG00000003527	EPHB2	1.84	-4.35	1.67	-2.61	0.01	0.01
ENSSSCG00000024872	CPT1C	1.84	-4.35	1.67	-2.60	0.01	0.01

ENSSSCG00000007757	TRIM72	55.52	-4.34	0.42	-10.24	0.00	0.00
ENSSSCG00000033922	ALKAL1	1.84	-4.34	1.69	-2.57	0.01	0.02
ENSSSCG00000028403	RF00091	1.84	-4.34	1.72	-2.52	0.01	0.02
ENSSSCG00000038616		1.83	-4.34	1.71	-2.54	0.01	0.02
ENSSSCG00000007216		6.86	-4.32	1.19	-3.62	0.00	0.00
ENSSSCG00000013041	FERMT3	3.68	-4.32	1.44	-3.01	0.00	0.00
ENSSSCG00000033451	HIST1H2AG	27.46	-4.31	0.60	-7.19	0.00	0.00
ENSSSCG00000013839	RASAL3	17.08	-4.31	0.76	-5.70	0.00	0.00
ENSSSCG00000013231	C1QTNF4	20.49	-4.31	0.69	-6.28	0.00	0.00
ENSSSCG00000035955		6.66	-4.29	1.20	-3.56	0.00	0.00
ENSSSCG00000037959		10.06	-4.29	0.97	-4.41	0.00	0.00
ENSSSCG00000038013	DIRAS1	16.71	-4.28	0.76	-5.66	0.00	0.00
ENSSSCG00000000195	PRPH	36.63	-4.27	0.52	-8.26	0.00	0.00
ENSSSCG00000040431	CRYGS	6.54	-4.26	1.20	-3.55	0.00	0.00
ENSSSCG00000013482	C19orf71	3.58	-4.26	1.46	-2.92	0.00	0.01
ENSSSCG00000000657		3.54	-4.26	1.45	-2.93	0.00	0.01
ENSSSCG00000039030		26.33	-4.26	0.60	-7.15	0.00	0.00
ENSSSCG00000000184	DDN	16.44	-4.26	0.76	-5.62	0.00	0.00
ENSSSCG00000024549	LYL1	26.14	-4.25	0.60	-7.11	0.00	0.00
ENSSSCG00000034644		1.71	-4.23	1.73	-2.44	0.01	0.02
ENSSSCG00000038818		1.71	-4.23	1.73	-2.44	0.01	0.02
ENSSSCG00000039921		32.29	-4.23	0.54	-7.84	0.00	0.00
ENSSSCG00000027465	NLGN3	36.01	-4.23	0.52	-8.19	0.00	0.00
ENSSSCG00000027762	TNFRSF11B	1.70	-4.23	1.74	-2.43	0.02	0.02
ENSSSCG00000017445		9.51	-4.22	0.99	-4.25	0.00	0.00

ENSSSCG00000036708		1.68	-4.21	1.75	-2.40	0.02	0.02
ENSSSCG00000038321		1.68	-4.21	1.72	-2.45	0.01	0.02
ENSSSCG00000023338	CHRNA6	1.68	-4.21	1.71	-2.46	0.01	0.02
ENSSSCG00000036808	HMGCS2	1.68	-4.21	1.71	-2.46	0.01	0.02
ENSSSCG00000034651		1.68	-4.21	1.71	-2.46	0.01	0.02
ENSSSCG00000036443	HECW1	1.68	-4.21	1.71	-2.46	0.01	0.02
ENSSSCG00000039983	GJA4	1.68	-4.21	1.71	-2.46	0.01	0.02
ENSSSCG00000015366	PRPS1L1	1.67	-4.21	1.72	-2.44	0.01	0.02
ENSSSCG00000015085	IL10RA	516.02	-4.21	0.14	-30.19	0.00	0.00
ENSSSCG00000033277	IQCF5	1.67	-4.20	1.72	-2.45	0.01	0.02
ENSSSCG00000036662	CHRM5	1.67	-4.20	1.72	-2.45	0.01	0.02
ENSSSCG00000040440		1.67	-4.20	1.72	-2.45	0.01	0.02
ENSSSCG00000025789		1.67	-4.20	1.81	-2.32	0.02	0.03
ENSSSCG00000005463	SHOC1	1.67	-4.20	1.72	-2.44	0.01	0.02
ENSSSCG00000038606		142.63	-4.20	0.26	-16.38	0.00	0.00
ENSSSCG00000040438		1.66	-4.20	1.75	-2.40	0.02	0.02
ENSSSCG00000037551		1.65	-4.20	1.76	-2.39	0.02	0.02
ENSSSCG00000001691	RSPH9	56.74	-4.19	0.41	-10.29	0.00	0.00
ENSSSCG00000004147	ECT2L	3.38	-4.19	1.48	-2.83	0.00	0.01
ENSSSCG00000035598	EDN1	156.93	-4.18	0.25	-16.98	0.00	0.00
ENSSSCG00000020970	IL6	333.65	-4.18	0.18	-23.70	0.00	0.00
ENSSSCG00000004128	ZC2HC1B	3.32	-4.17	1.48	-2.82	0.00	0.01
ENSSSCG00000009444	OLFM4	42.43	-4.14	0.46	-9.01	0.00	0.00
ENSSSCG00000013022	PYGM	65.81	-4.11	0.37	-11.23	0.00	0.00
ENSSSCG00000031649	RNASE1	3.20	-4.10	1.48	-2.78	0.01	0.01

ENSSSCG00000015652	IL10	3.20	-4.10	1.47	-2.78	0.01	0.01
ENSSSCG00000006754	AMPD1	55.98	-4.09	0.40	-10.35	0.00	0.00
ENSSSCG00000022692	RINL	14.82	-4.08	0.77	-5.32	0.00	0.00
ENSSSCG00000021755		1.54	-4.08	1.80	-2.26	0.02	0.03
ENSSSCG00000032001		1.54	-4.08	1.80	-2.26	0.02	0.03
ENSSSCG00000036306		1.54	-4.08	1.80	-2.26	0.02	0.03
ENSSSCG00000028363	TMPRSS9	1.54	-4.08	1.80	-2.26	0.02	0.03
ENSSSCG00000028561	CD28	3.13	-4.08	1.53	-2.66	0.01	0.01
ENSSSCG00000015291	LEMD1	1.53	-4.07	1.79	-2.28	0.02	0.03
ENSSSCG00000005122	TEK	1.52	-4.07	1.76	-2.31	0.02	0.03
ENSSSCG00000014124	ANKRD34B	1.52	-4.07	1.79	-2.27	0.02	0.03
ENSSSCG00000022014	DCDC2B	20.35	-4.06	0.65	-6.22	0.00	0.00
ENSSSCG00000002990	CNTD2	1.51	-4.06	1.76	-2.30	0.02	0.03
ENSSSCG00000017692	LHX1	1.51	-4.06	1.76	-2.30	0.02	0.03
ENSSSCG00000040960		1.51	-4.06	1.76	-2.30	0.02	0.03
ENSSSCG00000005474	SAL1	1.51	-4.06	1.76	-2.31	0.02	0.03
ENSSSCG00000009865	TBX3	14.50	-4.06	0.78	-5.22	0.00	0.00
ENSSSCG00000004285	OOEP	1.51	-4.06	1.76	-2.30	0.02	0.03
ENSSSCG00000005938		1.51	-4.06	1.76	-2.30	0.02	0.03
ENSSSCG00000003062	LYPD3	14.40	-4.06	0.77	-5.26	0.00	0.00
ENSSSCG00000006011	SLC30A8	1.51	-4.06	1.78	-2.28	0.02	0.03
ENSSSCG00000035950		25.94	-4.06	0.59	-6.90	0.00	0.00
ENSSSCG00000018533	RF00071	1.50	-4.06	1.77	-2.30	0.02	0.03
ENSSSCG00000034846		1.50	-4.06	1.77	-2.30	0.02	0.03
ENSSSCG00000011701	TM4SF18	1.50	-4.05	1.77	-2.29	0.02	0.03

ENSSSCG00000020984	LXN	1.50	-4.05	1.77	-2.29	0.02	0.03
ENSSSCG00000038112	SPDYC	1.50	-4.05	1.80	-2.25	0.02	0.03
ENSSSCG00000006649	HORMAD1	1.49	-4.05	1.79	-2.26	0.02	0.03
ENSSSCG00000032854	C15orf65	17.11	-4.04	0.70	-5.77	0.00	0.00
ENSSSCG00000021401	GFRA4	3.04	-4.03	1.50	-2.68	0.01	0.01
ENSSSCG00000040648	CCL11	3.03	-4.02	1.49	-2.70	0.01	0.01
ENSSSCG00000002501	BDKRB1	3.02	-4.02	1.49	-2.70	0.01	0.01
ENSSSCG00000029092	LRRD1	5.63	-4.02	1.25	-3.21	0.00	0.00
ENSSSCG00000038345	IKZF1	3.03	-4.01	1.48	-2.70	0.01	0.01
ENSSSCG00000013540	PSPN	3.02	-4.01	1.49	-2.69	0.01	0.01
ENSSSCG00000035053		53.16	-4.01	0.40	-10.06	0.00	0.00
ENSSSCG00000003284	FCAR	5.52	-4.01	1.25	-3.21	0.00	0.00
ENSSSCG00000021448	CCDC17	5.55	-4.01	1.23	-3.26	0.00	0.00
ENSSSCG00000028411	MC5R	25.13	-4.01	0.58	-6.94	0.00	0.00
ENSSSCG00000030350		2.98	-4.00	1.51	-2.64	0.01	0.01
ENSSSCG00000004301	C6orf163	11.31	-4.00	0.86	-4.63	0.00	0.00
ENSSSCG00000037626		5.50	-4.00	1.23	-3.26	0.00	0.00
ENSSSCG00000015596	FAM71A	16.43	-3.99	0.71	-5.60	0.00	0.00
ENSSSCG00000035104	TERB1	13.53	-3.96	0.79	-5.01	0.00	0.00
ENSSSCG00000011271	ZNF619	10.85	-3.96	0.89	-4.43	0.00	0.00
ENSSSCG00000026339		29.79	-3.95	0.53	-7.51	0.00	0.00
ENSSSCG00000034563		2.88	-3.94	1.53	-2.58	0.01	0.01
ENSSSCG00000028277		2.88	-3.94	1.52	-2.60	0.01	0.01
ENSSSCG00000034701	CALY	2.87	-3.93	1.50	-2.62	0.01	0.01
ENSSSCG00000032852	FAM167A	2.87	-3.93	1.50	-2.62	0.01	0.01

ENSSSCG00000017691	MRM1	2.84	-3.93	1.51	-2.61	0.01	0.01
ENSSSCG00000006829	SYPL2	92.84	-3.93	0.30	-13.22	0.00	0.00
ENSSSCG00000031526		21.30	-3.92	0.63	-6.25	0.00	0.00
ENSSSCG00000039300	IL27	13.20	-3.92	0.78	-5.04	0.00	0.00
ENSSSCG00000037976		1.38	-3.92	1.88	-2.08	0.04	0.05
ENSSSCG00000003498	UBXN10	2.81	-3.92	1.53	-2.56	0.01	0.02
ENSSSCG00000007178		10.43	-3.91	0.90	-4.36	0.00	0.00
ENSSSCG00000038099		5.37	-3.91	1.24	-3.16	0.00	0.00
ENSSSCG00000012266	SLC9A7	1.37	-3.91	1.85	-2.11	0.03	0.05
ENSSSCG00000011053	CDNF	15.68	-3.91	0.72	-5.46	0.00	0.00
ENSSSCG00000033909		146.75	-3.91	0.25	-15.75	0.00	0.00
ENSSSCG00000007240	HCK	1.36	-3.90	1.84	-2.12	0.03	0.05
ENSSSCG00000025759		1.35	-3.90	1.84	-2.11	0.03	0.05
ENSSSCG00000013420	KISS1R	18.23	-3.90	0.66	-5.89	0.00	0.00
ENSSSCG00000009489		1.35	-3.89	1.82	-2.14	0.03	0.04
ENSSSCG00000025468		1.35	-3.89	1.82	-2.14	0.03	0.04
ENSSSCG00000003833	DAB1	1.35	-3.89	1.82	-2.14	0.03	0.05
ENSSSCG00000008410	C2orf73	1.35	-3.89	1.82	-2.14	0.03	0.05
ENSSSCG00000035409		1.35	-3.89	1.82	-2.14	0.03	0.05
ENSSSCG00000002915	KIRREL2	1.34	-3.89	1.85	-2.11	0.03	0.05
ENSSSCG00000012527	TCEAL9	1.34	-3.89	1.85	-2.11	0.03	0.05
ENSSSCG00000021018	GSDMB	1.34	-3.89	1.85	-2.11	0.03	0.05
ENSSSCG00000040115	RF02104	1.34	-3.89	1.85	-2.11	0.03	0.05
ENSSSCG00000018700	RF00030	1.34	-3.89	1.86	-2.09	0.04	0.05
ENSSSCG00000039739		1.34	-3.89	1.86	-2.09	0.04	0.05

ENSSSCG00000000781	ABCD2	1.34	-3.89	1.83	-2.13	0.03	0.05
ENSSSCG00000036727		1.34	-3.89	1.83	-2.13	0.03	0.05
ENSSSCG00000014219	CDO1	79.97	-3.88	0.32	-12.05	0.00	0.00
ENSSSCG00000035595	HMCN1	1.34	-3.88	1.83	-2.12	0.03	0.05
ENSSSCG00000027405	RF00004	1.33	-3.88	1.86	-2.09	0.04	0.05
ENSSSCG00000031485		1.33	-3.88	1.86	-2.08	0.04	0.05
ENSSSCG00000030597	HAPLN3	5.19	-3.88	1.23	-3.15	0.00	0.00
ENSSSCG00000001403	LTA	10.13	-3.87	0.91	-4.26	0.00	0.00
ENSSSCG00000019512	RF00026	5.06	-3.87	1.27	-3.05	0.00	0.00
ENSSSCG00000006791	OVGP1	100.33	-3.87	0.29	-13.48	0.00	0.00
ENSSSCG00000000668	APOBEC1	305.40	-3.86	0.16	-23.45	0.00	0.00
ENSSSCG00000002737	CHST4	135.90	-3.86	0.25	-15.32	0.00	0.00
ENSSSCG00000000138	PVALB	5.00	-3.85	1.24	-3.10	0.00	0.00
ENSSSCG00000016183		7.54	-3.85	1.01	-3.80	0.00	0.00
ENSSSCG00000026317	SLC10A2	113.23	-3.84	0.26	-14.55	0.00	0.00
ENSSSCG00000012492	DRP2	9.93	-3.84	0.90	-4.29	0.00	0.00
ENSSSCG00000032810	GZMM	2.71	-3.84	1.53	-2.51	0.01	0.02
ENSSSCG00000033007		2.68	-3.84	1.52	-2.53	0.01	0.02
ENSSSCG00000003810		2.69	-3.83	1.55	-2.48	0.01	0.02
ENSSSCG00000003819	ANGPTL3	2.66	-3.83	1.53	-2.50	0.01	0.02
ENSSSCG00000033894		4.98	-3.83	1.26	-3.03	0.00	0.00
ENSSSCG00000028387	CNKSR1	34.70	-3.83	0.47	-8.10	0.00	0.00
ENSSSCG00000017893	PIMREG	7.32	-3.82	1.03	-3.70	0.00	0.00
ENSSSCG00000040139	B3GNT9	17.45	-3.82	0.67	-5.72	0.00	0.00
ENSSSCG00000039673	ASB16	83.46	-3.81	0.31	-12.15	0.00	0.00

ENSSSCG00000037013	WFIKKN1	14.75	-3.81	0.72	-5.29	0.00	0.00
ENSSSCG00000040414		9.60	-3.80	0.92	-4.11	0.00	0.00
ENSSSCG00000021651	SCN2B	9.72	-3.80	0.88	-4.32	0.00	0.00
ENSSSCG00000035337		4.84	-3.78	1.26	-2.99	0.00	0.00
ENSSSCG00000013617	ELAVL3	7.20	-3.77	1.04	-3.63	0.00	0.00
ENSSSCG00000001751	CHRNA5	9.56	-3.77	0.89	-4.25	0.00	0.00
ENSSSCG00000036338		2.56	-3.76	1.65	-2.28	0.02	0.03
ENSSSCG00000007805	ATP2A1	37.86	-3.75	0.44	-8.45	0.00	0.00
ENSSSCG00000031346	CMKLR1	2.53	-3.75	1.59	-2.36	0.02	0.03
ENSSSCG00000040038	ZNF8	2.53	-3.74	1.55	-2.42	0.02	0.02
ENSSSCG00000016184		2.51	-3.74	1.54	-2.43	0.02	0.02
ENSSSCG00000000740	TEX52	2.51	-3.74	1.55	-2.41	0.02	0.02
ENSSSCG00000014861		4.71	-3.73	1.25	-2.99	0.00	0.00
ENSSSCG00000023718		2.50	-3.73	1.60	-2.33	0.02	0.03
ENSSSCG00000017358	SLC4A1	4.71	-3.73	1.25	-2.99	0.00	0.00
ENSSSCG00000037803	MARCKS	30.02	-3.73	0.51	-7.33	0.00	0.00
ENSSSCG00000023329	APC2	36.95	-3.71	0.44	-8.34	0.00	0.00
ENSSSCG00000034821	ARMCX4	6.85	-3.69	1.03	-3.57	0.00	0.00
ENSSSCG00000003483		18.34	-3.68	0.64	-5.80	0.00	0.00
ENSSSCG00000024357	DNAI2	80.82	-3.67	0.30	-12.16	0.00	0.00
ENSSSCG00000014328	GFRA3	15.57	-3.66	0.70	-5.26	0.00	0.00
ENSSSCG00000006489	TMEM79	2.38	-3.65	1.61	-2.26	0.02	0.03
ENSSSCG00000017347	HIGD1B	2.37	-3.64	1.58	-2.30	0.02	0.03
ENSSSCG00000013935		4.39	-3.64	1.31	-2.78	0.01	0.01
ENSSSCG00000029158	ZMAT2	8.89	-3.64	0.90	-4.04	0.00	0.00

ENSSSCG00000000713		4.35	-3.64	1.27	-2.86	0.00	0.01
ENSSSCG00000022423		2.34	-3.63	1.56	-2.32	0.02	0.03
ENSSSCG00000003324	ZNF471	2.34	-3.63	1.57	-2.32	0.02	0.03
ENSSSCG00000033936		2.34	-3.63	1.57	-2.32	0.02	0.03
ENSSSCG00000036586		4.45	-3.63	1.34	-2.71	0.01	0.01
ENSSSCG00000038635	ZNF554	2.34	-3.63	1.56	-2.32	0.02	0.03
ENSSSCG00000035340		2.33	-3.62	1.57	-2.31	0.02	0.03
ENSSSCG00000039339		4.39	-3.62	1.27	-2.85	0.00	0.01
ENSSSCG00000035175		8.63	-3.62	0.92	-3.93	0.00	0.00
ENSSSCG00000017770	PROCA1	43.46	-3.62	0.41	-8.89	0.00	0.00
ENSSSCG00000000188	DHH	21.81	-3.62	0.57	-6.35	0.00	0.00
ENSSSCG00000006551	AQP10	15.07	-3.61	0.68	-5.29	0.00	0.00
ENSSSCG00000006153	FABP5	4.36	-3.61	1.26	-2.85	0.00	0.01
ENSSSCG00000012700		8.61	-3.59	0.90	-3.97	0.00	0.00
ENSSSCG00000037822	FBXL13	73.91	-3.58	0.31	-11.58	0.00	0.00
ENSSSCG00000008835	RASL11B	8.55	-3.58	0.91	-3.94	0.00	0.00
ENSSSCG00000000271	AMHR2	10.52	-3.57	0.81	-4.41	0.00	0.00
ENSSSCG00000011627	ACPP	4.22	-3.56	1.29	-2.77	0.01	0.01
ENSSSCG00000033675	SPAI-2	4.20	-3.55	1.27	-2.79	0.01	0.01
ENSSSCG00000038182		61.40	-3.54	0.55	-6.47	0.00	0.00
ENSSSCG00000034799		2.22	-3.54	1.66	-2.13	0.03	0.05
ENSSSCG00000011894	POPDC2	8.13	-3.54	0.94	-3.77	0.00	0.00
ENSSSCG00000008595	APOB	316.26	-3.54	0.15	-23.38	0.00	0.00
ENSSSCG00000007079	FLRT3	1103.38	-3.53	0.11	-33.59	0.00	0.00
ENSSSCG00000035214		2.19	-3.53	1.63	-2.17	0.03	0.04

ENSSSCG00000040553		2.19	-3.52	1.60	-2.20	0.03	0.04
ENSSSCG00000015891	TBR1	2.18	-3.52	1.63	-2.16	0.03	0.04
ENSSSCG00000018007	MYH3	18.41	-3.52	0.63	-5.60	0.00	0.00
ENSSSCG00000037638		2.19	-3.52	1.60	-2.20	0.03	0.04
ENSSSCG00000000932	NTS	2.21	-3.52	1.59	-2.22	0.03	0.04
ENSSSCG00000011362	TMEM89	2.17	-3.51	1.59	-2.21	0.03	0.04
ENSSSCG00000035154		2.18	-3.51	1.59	-2.21	0.03	0.04
ENSSSCG00000037466		2.17	-3.51	1.59	-2.21	0.03	0.04
ENSSSCG00000037555		2.17	-3.51	1.59	-2.21	0.03	0.04
ENSSSCG00000034914		2.17	-3.51	1.59	-2.21	0.03	0.04
ENSSSCG00000007211	ZCCHC3	4.04	-3.51	1.32	-2.65	0.01	0.01
ENSSSCG00000013111	CD6	2.17	-3.51	1.59	-2.20	0.03	0.04
ENSSSCG00000034237	C10orf82	2.17	-3.51	1.59	-2.20	0.03	0.04
ENSSSCG00000021473		6.07	-3.51	1.09	-3.22	0.00	0.00
ENSSSCG00000001405	LTB	2.15	-3.50	1.65	-2.13	0.03	0.05
ENSSSCG00000028214		52.53	-3.50	0.36	-9.73	0.00	0.00
ENSSSCG00000016192	PLCD4	15.91	-3.50	0.66	-5.33	0.00	0.00
ENSSSCG00000001404	TNF	96.08	-3.49	0.27	-12.93	0.00	0.00
ENSSSCG00000017488	CSF3	381.73	-3.49	0.25	-13.89	0.00	0.00
ENSSSCG00000017389	RAMP2	6.05	-3.48	1.06	-3.28	0.00	0.00
ENSSSCG00000001807	AP3B2	4.02	-3.48	1.29	-2.71	0.01	0.01
ENSSSCG00000013740	SYCE2	71.11	-3.47	0.31	-11.34	0.00	0.00
ENSSSCG00000029371	C5AR1	5.84	-3.46	1.06	-3.26	0.00	0.00
ENSSSCG00000034420	RF00026	3.83	-3.45	1.31	-2.63	0.01	0.01
ENSSSCG00000000660	A2M	21.31	-3.44	0.55	-6.19	0.00	0.00

ENSSSCG00000017439	KRT32	3.83	-3.43	1.32	-2.60	0.01	0.01
ENSSSCG00000031377	RF00003	3.81	-3.42	1.37	-2.50	0.01	0.02
ENSSSCG00000036566	LY6G6C	132.57	-3.42	0.23	-15.09	0.00	0.00
ENSSSCG00000033256		69.22	-3.42	0.31	-11.01	0.00	0.00
ENSSSCG00000035865		5.68	-3.42	1.07	-3.18	0.00	0.00
ENSSSCG00000035825	UBE2QL1	53.35	-3.42	0.35	-9.71	0.00	0.00
ENSSSCG00000040308		3.86	-3.41	1.29	-2.65	0.01	0.01
ENSSSCG00000027923	GPR142	3.84	-3.41	1.30	-2.62	0.01	0.01
ENSSSCG00000036549	DPYSL3	2.03	-3.41	1.65	-2.06	0.04	0.05
ENSSSCG00000011618	GATA2	148.69	-3.41	0.22	-15.19	0.00	0.00
ENSSSCG00000011487	FEZF2	5.68	-3.40	1.07	-3.19	0.00	0.00
ENSSSCG00000006415	CADM3	2.05	-3.40	1.65	-2.06	0.04	0.05
ENSSSCG00000009126		2.01	-3.39	1.62	-2.10	0.04	0.05
ENSSSCG00000039446		2.01	-3.39	1.67	-2.04	0.04	0.06
ENSSSCG00000005657	PKN3	69.27	-3.39	0.31	-10.98	0.00	0.00
ENSSSCG00000037684	PDYN	5.52	-3.39	1.12	-3.02	0.00	0.00
ENSSSCG00000028446	PCBP4	2.00	-3.39	1.62	-2.09	0.04	0.05
ENSSSCG00000017377		11.18	-3.38	0.77	-4.41	0.00	0.00
ENSSSCG00000029849	S1PR1	31.84	-3.38	0.45	-7.49	0.00	0.00
ENSSSCG00000021220	CKB	3107.61	-3.38	0.07	-51.63	0.00	0.00
ENSSSCG00000004157	IL20RA	1.99	-3.38	1.63	-2.07	0.04	0.05
ENSSSCG00000031750		1.99	-3.38	1.63	-2.07	0.04	0.05
ENSSSCG00000039442	BMP2	100.98	-3.38	0.26	-13.23	0.00	0.00
ENSSSCG00000000664		1.98	-3.37	1.66	-2.03	0.04	0.06
ENSSSCG00000036237	ITGA7	3.66	-3.37	1.31	-2.56	0.01	0.02

ENSSSCG00000037660		3.75	-3.36	1.33	-2.52	0.01	0.02
ENSSSCG00000028908	DYNLT3	22.06	-3.36	0.54	-6.26	0.00	0.00
ENSSSCG00000012720		3.74	-3.36	1.31	-2.55	0.01	0.02
ENSSSCG00000010084		53.47	-3.36	0.35	-9.63	0.00	0.00
ENSSSCG00000028115	ALDH8A1	5.56	-3.36	1.08	-3.11	0.00	0.00
ENSSSCG00000012490	TMEM35A	9.18	-3.35	0.84	-4.00	0.00	0.00
ENSSSCG00000016999	TLX3	132.09	-3.35	0.22	-15.04	0.00	0.00
ENSSSCG00000034056		8.97	-3.34	0.84	-3.96	0.00	0.00
ENSSSCG00000029754	SLC39A2	18.03	-3.33	0.59	-5.61	0.00	0.00
ENSSSCG00000019768	RF00007	27.09	-3.32	0.48	-6.89	0.00	0.00
ENSSSCG00000033542		14.18	-3.32	0.71	-4.70	0.00	0.00
ENSSSCG00000003192	IL4I1	39.27	-3.32	0.41	-8.18	0.00	0.00
ENSSSCG00000036060	RRAD	17.86	-3.31	0.59	-5.58	0.00	0.00
ENSSSCG00000014093	CRHBP	55.70	-3.31	0.34	-9.67	0.00	0.00
ENSSSCG00000010336	SFTPA1	3.50	-3.30	1.32	-2.50	0.01	0.02
ENSSSCG00000033173	SPINK14	3.50	-3.30	1.32	-2.50	0.01	0.02
ENSSSCG00000031114	SLFN14	10.48	-3.29	0.78	-4.23	0.00	0.00
ENSSSCG00000004898	TNFRSF11A	7.07	-3.29	0.93	-3.52	0.00	0.00
ENSSSCG00000002001	REC8	21.17	-3.28	0.54	-6.03	0.00	0.00
ENSSSCG00000011734	ARL14	5.22	-3.27	1.12	-2.94	0.00	0.01
ENSSSCG00000013473	GNA15	10.36	-3.27	0.78	-4.19	0.00	0.00
ENSSSCG00000006542	KCNN3	7.02	-3.27	0.94	-3.48	0.00	0.00
ENSSSCG00000033919	DCLK1	22.73	-3.27	0.53	-6.22	0.00	0.00
ENSSSCG00000003286		1.85	-3.26	1.66	-1.96	0.05	0.07
ENSSSCG00000017095	SEMA5A	1.86	-3.26	1.66	-1.96	0.05	0.07

ENSSSCG00000038052		1.87	-3.26	1.65	-1.97	0.05	0.07
ENSSSCG00000024431	ATG9B	18.96	-3.26	0.57	-5.68	0.00	0.00
ENSSSCG00000010971	ARID3C	17.08	-3.25	0.60	-5.40	0.00	0.00
ENSSSCG00000022017	LGALS4	56.43	-3.25	0.34	-9.69	0.00	0.00
ENSSSCG00000034429	PLA2G5	6.93	-3.24	0.94	-3.44	0.00	0.00
ENSSSCG00000038148	PROKR1	12.05	-3.24	0.71	-4.56	0.00	0.00
ENSSSCG00000038945		28.87	-3.24	0.47	-6.87	0.00	0.00
ENSSSCG00000006379	CD48	32.42	-3.23	0.45	-7.24	0.00	0.00
ENSSSCG00000009388	KCNRG	15.16	-3.22	0.65	-5.00	0.00	0.00
ENSSSCG00000031717	ADCY8	3.34	-3.22	1.33	-2.42	0.02	0.02
ENSSSCG00000040393	DCAF16	16.88	-3.22	0.61	-5.28	0.00	0.00
ENSSSCG00000023004	FZD9	8.38	-3.21	0.85	-3.80	0.00	0.00
ENSSSCG00000038514	DDAH2	18.40	-3.21	0.57	-5.65	0.00	0.00
ENSSSCG00000011622	KBTBD12	3.32	-3.21	1.36	-2.36	0.02	0.03
ENSSSCG00000013653	ICAM5	40.08	-3.20	0.39	-8.27	0.00	0.00
ENSSSCG00000037973	KCNAB3	43.35	-3.20	0.39	-8.28	0.00	0.00
ENSSSCG00000037177	C2CD4C	8.25	-3.19	0.86	-3.71	0.00	0.00
ENSSSCG00000034481		6.66	-3.18	0.97	-3.30	0.00	0.00
ENSSSCG00000016623		8.18	-3.18	0.88	-3.60	0.00	0.00
ENSSSCG00000012485	NOX1	14.97	-3.18	0.65	-4.91	0.00	0.00
ENSSSCG00000032785		6.46	-3.18	0.97	-3.29	0.00	0.00
ENSSSCG00000009239		318.19	-3.17	0.14	-22.33	0.00	0.00
ENSSSCG00000001427	C4A	24.68	-3.17	0.49	-6.46	0.00	0.00
ENSSSCG00000039190		7.93	-3.16	0.89	-3.55	0.00	0.00
ENSSSCG00000009950		4.88	-3.15	1.09	-2.90	0.00	0.01

ENSSSCG00000028284	KCND1	4.85	-3.15	1.09	-2.89	0.00	0.01
ENSSSCG00000033369		9.70	-3.15	0.78	-4.04	0.00	0.00
ENSSSCG00000038289	ZNF234	9.73	-3.15	0.77	-4.06	0.00	0.00
ENSSSCG00000017645	TEX14	89.73	-3.14	0.26	-11.94	0.00	0.00
ENSSSCG00000003145	FUT2	3.16	-3.14	1.35	-2.33	0.02	0.03
ENSSSCG00000005438	ACTL7B	6.35	-3.13	0.96	-3.25	0.00	0.00
ENSSSCG00000040053	LSMEM1	7.98	-3.13	0.87	-3.60	0.00	0.00
ENSSSCG00000006858	OLFM3	53.99	-3.12	0.33	-9.44	0.00	0.00
ENSSSCG00000033590		9.50	-3.12	0.79	-3.95	0.00	0.00
ENSSSCG00000029761		18.92	-3.11	0.55	-5.61	0.00	0.00
ENSSSCG00000039101	C1QL1	36.25	-3.11	0.41	-7.57	0.00	0.00
ENSSSCG00000001459	HLA-DOB	62.90	-3.10	0.31	-10.09	0.00	0.00
ENSSSCG00000025870	HEPACAM2	7.85	-3.10	0.87	-3.58	0.00	0.00
ENSSSCG00000012066	KCNJ15	3.16	-3.09	1.40	-2.21	0.03	0.04
ENSSSCG00000028331	IL1R2	9.17	-3.09	0.82	-3.75	0.00	0.00
ENSSSCG00000033684	CD79A	149.12	-3.08	0.20	-15.50	0.00	0.00
ENSSSCG00000015659	YOD1	1897.98	-3.07	0.08	-39.04	0.00	0.00
ENSSSCG00000016254	CCL20	3525.16	-3.07	0.14	-22.02	0.00	0.00
ENSSSCG00000000186	RHEBL1	69.25	-3.07	0.29	-10.44	0.00	0.00
ENSSSCG00000033390		631.83	-3.07	0.11	-27.38	0.00	0.00
ENSSSCG00000021813	SLC26A3	17.07	-3.06	0.61	-5.01	0.00	0.00
ENSSSCG00000031143		6.19	-3.06	0.97	-3.15	0.00	0.00
ENSSSCG00000013991	C1orf35	6.04	-3.06	1.00	-3.05	0.00	0.00
ENSSSCG00000001555	SLC26A8	3.00	-3.05	1.38	-2.22	0.03	0.04
ENSSSCG00000000413	MYO1A	54.51	-3.05	0.33	-9.35	0.00	0.00

ENSSSCG00000031856	DACT1	94.28	-3.05	0.26	-11.91	0.00	0.00
ENSSSCG00000017044	IL12B	3.02	-3.05	1.38	-2.21	0.03	0.04
ENSSSCG00000031756		6.13	-3.05	1.01	-3.02	0.00	0.00
ENSSSCG00000013765		15.20	-3.04	0.61	-4.95	0.00	0.00
ENSSSCG00000002761		7.53	-3.04	0.86	-3.54	0.00	0.00
ENSSSCG00000005890	ZNF7	38.03	-3.04	0.39	-7.80	0.00	0.00
ENSSSCG00000038927	ZNF649	3.00	-3.04	1.38	-2.20	0.03	0.04
ENSSSCG00000002901	UPK1A	2.98	-3.04	1.42	-2.13	0.03	0.05
ENSSSCG00000036618		63.22	-3.03	0.32	-9.41	0.00	0.00
ENSSSCG00000010725	HMX2	4.53	-3.03	1.13	-2.68	0.01	0.01
ENSSSCG00000000464	C12orf56	14.86	-3.03	0.63	-4.84	0.00	0.00
ENSSSCG00000012678		7.56	-3.03	0.87	-3.46	0.00	0.00
ENSSSCG00000032749	PCDH18	67.46	-3.02	0.29	-10.33	0.00	0.00
ENSSSCG00000013401	DKK3	28.55	-3.02	0.46	-6.56	0.00	0.00
ENSSSCG00000021434		31.01	-3.00	0.43	-7.01	0.00	0.00
ENSSSCG00000032902	KLK7	16.09	-3.00	0.59	-5.05	0.00	0.00
ENSSSCG00000003669	MFSD2A	518.67	-2.99	0.11	-27.27	0.00	0.00
ENSSSCG00000039481		4.42	-2.99	1.16	-2.57	0.01	0.01
ENSSSCG00000038946	SLC17A8	4.31	-2.98	1.14	-2.63	0.01	0.01
ENSSSCG00000010190	ACTA1	4.39	-2.98	1.13	-2.64	0.01	0.01
ENSSSCG00000027226	EPN3	13.07	-2.98	0.65	-4.58	0.00	0.00
ENSSSCG00000016239	NYAP2	4.37	-2.98	1.13	-2.64	0.01	0.01
ENSSSCG00000037275	SNX22	15.98	-2.97	0.60	-4.99	0.00	0.00
ENSSSCG00000018682	RF01268	10.02	-2.97	0.74	-4.01	0.00	0.00
ENSSSCG00000002863	LRP3	2.80	-2.96	1.42	-2.08	0.04	0.05

ENSSSCG00000034848		7.20	-2.96	0.87	-3.41	0.00	0.00
ENSSSCG00000031751		44.49	-2.95	0.37	-7.91	0.00	0.00
ENSSSCG00000040681	FABP4	4.39	-2.95	1.14	-2.60	0.01	0.01
ENSSSCG00000032149	PLET1	108.79	-2.95	0.24	-12.34	0.00	0.00
ENSSSCG00000035972	TULP1	2.81	-2.95	1.39	-2.13	0.03	0.05
ENSSSCG00000023906	RF00553	4.38	-2.95	1.12	-2.63	0.01	0.01
ENSSSCG00000006384	NHLH1	12.96	-2.95	0.65	-4.53	0.00	0.00
ENSSSCG00000011884	HCLS1	2.83	-2.95	1.40	-2.11	0.04	0.05
ENSSSCG00000017863	TRPV1	66.74	-2.94	0.30	-9.82	0.00	0.00
ENSSSCG00000036190	GIPC3	5.73	-2.94	0.97	-3.02	0.00	0.00
ENSSSCG00000000231	ANKRD33	17.04	-2.93	0.58	-5.06	0.00	0.00
ENSSSCG00000017948	EFNB3	126.94	-2.93	0.21	-13.89	0.00	0.00
ENSSSCG00000025686	KMO	12.53	-2.93	0.67	-4.39	0.00	0.00
ENSSSCG00000006512	FDPS	56.37	-2.92	0.32	-9.17	0.00	0.00
ENSSSCG00000033245	GLYATL3	8.29	-2.91	0.82	-3.55	0.00	0.00
ENSSSCG00000037563	RF00002	94.40	-2.91	0.25	-11.86	0.00	0.00
ENSSSCG00000028661	ENKD1	15.17	-2.91	0.60	-4.86	0.00	0.00
ENSSSCG00000033340		6.82	-2.90	0.89	-3.25	0.00	0.00
ENSSSCG00000005636	SLC25A25	495.41	-2.90	0.12	-24.60	0.00	0.00
ENSSSCG00000002821	CCL22	156.72	-2.88	0.21	-13.82	0.00	0.00
ENSSSCG00000035227	ESRP2	185.34	-2.88	0.18	-15.68	0.00	0.00
ENSSSCG00000019599	RF00432	9.50	-2.88	0.75	-3.82	0.00	0.00
ENSSSCG00000040973	HYDIN	8.20	-2.87	0.80	-3.60	0.00	0.00
ENSSSCG00000024930		16.43	-2.87	0.57	-5.04	0.00	0.00
ENSSSCG00000035906	SLC13A3	9.54	-2.87	0.74	-3.87	0.00	0.00

ENSSSCG00000013385	INSC	10.86	-2.87	0.70	-4.11	0.00	0.00
ENSSSCG00000029189	DCHS1	6.67	-2.87	0.90	-3.19	0.00	0.00
ENSSSCG00000003811	ROR1	2.63	-2.86	1.45	-1.98	0.05	0.07
ENSSSCG00000036439	IL29	2.66	-2.86	1.40	-2.04	0.04	0.06
ENSSSCG00000002006	PSME1	10.83	-2.86	0.71	-4.03	0.00	0.00
ENSSSCG00000010475	CYP26A1	143.34	-2.86	0.20	-14.62	0.00	0.00
ENSSSCG00000033020	SLA2	2.68	-2.86	1.43	-2.00	0.05	0.06
ENSSSCG00000033049	KLHL41	3.98	-2.85	1.15	-2.47	0.01	0.02
ENSSSCG00000031379	RF02137	4.03	-2.85	1.15	-2.49	0.01	0.02
ENSSSCG00000011730	IL12A	12.13	-2.85	0.67	-4.27	0.00	0.00
ENSSSCG00000011107	CCNY	10.71	-2.85	0.70	-4.08	0.00	0.00
ENSSSCG00000032429	RAX2	9.34	-2.85	0.75	-3.78	0.00	0.00
ENSSSCG00000006165	SEC31B	62.87	-2.85	0.30	-9.65	0.00	0.00
ENSSSCG00000000400	MIP	2.70	-2.84	1.41	-2.01	0.04	0.06
ENSSSCG00000010298	CFAP70	5.41	-2.84	1.00	-2.84	0.00	0.01
ENSSSCG00000022395	SLC25A35	73.14	-2.84	0.28	-10.18	0.00	0.00
ENSSSCG00000040923	HIST1H2BN	5.35	-2.83	1.00	-2.84	0.00	0.01
ENSSSCG00000019224	RF00606	4.06	-2.83	1.17	-2.42	0.02	0.02
ENSSSCG00000027325	TRNP1	138.76	-2.83	0.20	-14.09	0.00	0.00
ENSSSCG00000026849	CCNO	83.62	-2.83	0.25	-11.21	0.00	0.00
ENSSSCG00000024072	P2RY11	39.71	-2.82	0.38	-7.43	0.00	0.00
ENSSSCG00000014948	C11orf54	621.86	-2.82	0.11	-26.30	0.00	0.00
ENSSSCG00000038990		2.67	-2.82	1.40	-2.01	0.04	0.06
ENSSSCG00000008299	C2orf78	6.55	-2.81	0.90	-3.14	0.00	0.00
ENSSSCG00000001252		133.59	-2.81	0.21	-13.42	0.00	0.00

ENSSSCG00000010348	CDHR1	5.17	-2.81	1.00	-2.80	0.01	0.01
ENSSSCG00000039909	ICAM2	11.68	-2.81	0.67	-4.20	0.00	0.00
ENSSSCG00000034657	LHX3	4.02	-2.81	1.16	-2.43	0.02	0.02
ENSSSCG00000032298	ABCC11	7.72	-2.81	0.85	-3.30	0.00	0.00
ENSSSCG00000003585	GJB5	84.20	-2.81	0.26	-10.91	0.00	0.00
ENSSSCG00000006051	CTHRC1	41.43	-2.81	0.37	-7.66	0.00	0.00
ENSSSCG00000010506	OPALIN	2.72	-2.80	1.41	-1.99	0.05	0.06
ENSSSCG00000037039	PPP1R27	5.13	-2.80	1.01	-2.76	0.01	0.01
ENSSSCG00000037567		90.65	-2.80	0.54	-5.21	0.00	0.00
ENSSSCG00000039482		2.71	-2.80	1.41	-1.99	0.05	0.06
ENSSSCG00000015969	CHRNA1	6.50	-2.80	0.90	-3.12	0.00	0.00
ENSSSCG00000004493	SIGLEC15	3.82	-2.80	1.17	-2.38	0.02	0.02
ENSSSCG00000012649	SMARCA1	3.78	-2.79	1.21	-2.30	0.02	0.03
ENSSSCG00000001613	TREML1	7.83	-2.79	0.85	-3.29	0.00	0.00
ENSSSCG00000039183	NDUFAF8	183.55	-2.79	0.18	-15.70	0.00	0.00
ENSSSCG00000004831		7.95	-2.79	0.84	-3.31	0.00	0.00
ENSSSCG00000017470	TNS4	513.82	-2.78	0.12	-23.93	0.00	0.00
ENSSSCG00000012517		320.64	-2.78	0.14	-20.10	0.00	0.00
ENSSSCG00000034449	RSRP1	1462.72	-2.77	0.08	-35.22	0.00	0.00
ENSSSCG00000012409	CITED1	59.87	-2.77	0.30	-9.17	0.00	0.00
ENSSSCG00000039660		5.16	-2.77	1.02	-2.72	0.01	0.01
ENSSSCG00000015326	COL1A2	85.74	-2.77	0.26	-10.80	0.00	0.00
ENSSSCG00000035610		3.87	-2.75	1.16	-2.37	0.02	0.03
ENSSSCG00000007593	ANKRD61	7.69	-2.75	0.83	-3.31	0.00	0.00
ENSSSCG00000035447		5.01	-2.75	1.02	-2.69	0.01	0.01

ENSSSCG00000033520	IL23A	64.61	-2.75	0.31	-8.95	0.00	0.00
ENSSSCG00000025500		13.83	-2.75	0.61	-4.49	0.00	0.00
ENSSSCG00000012278	CFP	6.41	-2.74	0.90	-3.06	0.00	0.00
ENSSSCG00000015433		8.66	-2.73	0.77	-3.56	0.00	0.00
ENSSSCG00000022292	NPL	43.55	-2.73	0.35	-7.81	0.00	0.00
ENSSSCG00000032411	ENGASE	237.33	-2.72	0.15	-17.94	0.00	0.00
ENSSSCG00000038829		13.69	-2.72	0.62	-4.41	0.00	0.00
ENSSSCG00000000436	PIP4K2C	20.85	-2.72	0.50	-5.44	0.00	0.00
ENSSSCG00000033100		48.55	-2.72	0.33	-8.26	0.00	0.00
ENSSSCG00000040714	RSPH6A	23.67	-2.72	0.46	-5.85	0.00	0.00
ENSSSCG00000002483		8.73	-2.71	0.76	-3.55	0.00	0.00
ENSSSCG00000009601	SLC18A1	3.68	-2.71	1.18	-2.29	0.02	0.03
ENSSSCG00000031898	CALHM5	57.92	-2.70	0.30	-8.97	0.00	0.00
ENSSSCG00000022305	WDR78	56.67	-2.70	0.31	-8.80	0.00	0.00
ENSSSCG00000034471	PSORS1C2	14.74	-2.70	0.58	-4.64	0.00	0.00
ENSSSCG00000028777	MYLK4	3.68	-2.70	1.15	-2.34	0.02	0.03
ENSSSCG00000035724		16.99	-2.69	0.55	-4.92	0.00	0.00
ENSSSCG00000014570	NRIP3	3.66	-2.69	1.16	-2.31	0.02	0.03
ENSSSCG00000040690	LRRC61	111.23	-2.69	0.22	-12.37	0.00	0.00
ENSSSCG00000034942	STAR	17.08	-2.69	0.54	-4.93	0.00	0.00
ENSSSCG00000017544	ATP5MC1	2051.04	-2.68	0.07	-36.05	0.00	0.00
ENSSSCG00000035518		210.86	-2.68	0.16	-16.76	0.00	0.00
ENSSSCG00000003333	C1QTNF12	12.03	-2.68	0.65	-4.15	0.00	0.00
ENSSSCG00000021862	PTGER2	3.70	-2.67	1.17	-2.28	0.02	0.03
ENSSSCG00000024634	GPRL15	128.22	-2.66	0.20	-13.22	0.00	0.00

ENSSSCG00000003576	GPR3	20.34	-2.66	0.51	-5.22	0.00	0.00
ENSSSCG00000002039	MMP14	3.74	-2.65	1.19	-2.22	0.03	0.04
ENSSSCG00000006932		19.12	-2.65	0.54	-4.93	0.00	0.00
ENSSSCG00000003306	HELZ2	9.64	-2.65	0.74	-3.57	0.00	0.00
ENSSSCG00000021569	MMP25	25.16	-2.65	0.45	-5.96	0.00	0.00
ENSSSCG00000032536	B3GNT8	7.20	-2.65	0.83	-3.19	0.00	0.00
ENSSSCG00000040720	SLC26A9	33.24	-2.65	0.39	-6.78	0.00	0.00
ENSSSCG00000013241	NR1H3	25.97	-2.63	0.44	-5.98	0.00	0.00
ENSSSCG00000001207	PGBD1	128.60	-2.63	0.20	-12.91	0.00	0.00
ENSSSCG00000040566		30.55	-2.62	0.41	-6.46	0.00	0.00
ENSSSCG00000027689		1437.82	-2.62	0.09	-30.75	0.00	0.00
ENSSSCG00000003616	FAM167B	43.20	-2.62	0.35	-7.58	0.00	0.00
ENSSSCG00000022706	ERICH6	3.54	-2.62	1.20	-2.18	0.03	0.04
ENSSSCG00000013880		169.03	-2.61	0.18	-14.88	0.00	0.00
ENSSSCG00000022584	PPP1R3F	37.28	-2.61	0.37	-7.01	0.00	0.00
ENSSSCG00000011195	GALNT15	7.01	-2.61	0.84	-3.09	0.00	0.00
ENSSSCG00000000385	SLC39A5	38.30	-2.61	0.36	-7.31	0.00	0.00
ENSSSCG00000012950	RIN1	230.84	-2.61	0.16	-16.37	0.00	0.00
ENSSSCG00000012658	RAB33A	18.55	-2.60	0.52	-5.05	0.00	0.00
ENSSSCG00000004807	SCG5	13.80	-2.60	0.61	-4.24	0.00	0.00
ENSSSCG00000004573		4.74	-2.60	1.04	-2.51	0.01	0.02
ENSSSCG00000030300		682.93	-2.60	0.10	-24.84	0.00	0.00
ENSSSCG00000012970	CTSW	3.55	-2.60	1.19	-2.19	0.03	0.04
ENSSSCG00000009935	MYO1H	5.64	-2.59	0.93	-2.78	0.01	0.01
ENSSSCG00000039557	TRIM54	25.16	-2.59	0.46	-5.64	0.00	0.00

ENSSSCG00000017410	HCRT	4.49	-2.59	1.07	-2.42	0.02	0.02
ENSSSCG00000038753	RF00002	39.21	-2.59	0.35	-7.31	0.00	0.00
ENSSSCG00000013092	PGA5	74.24	-2.59	0.27	-9.57	0.00	0.00
ENSSSCG00000014035	B4GALT7	103.77	-2.59	0.22	-11.52	0.00	0.00
ENSSSCG00000006639	C1orf56	11.51	-2.58	0.67	-3.87	0.00	0.00
ENSSSCG00000032254	ssc-mir-7144	13.77	-2.58	0.62	-4.19	0.00	0.00
ENSSSCG00000034568	RNASE4	69.14	-2.58	0.28	-9.17	0.00	0.00
ENSSSCG00000009979	NEFH	5.71	-2.57	0.92	-2.80	0.01	0.01
ENSSSCG00000015662	C4BPA	72.83	-2.57	0.26	-9.89	0.00	0.00
ENSSSCG00000001637	GUCA1B	3.31	-2.56	1.22	-2.10	0.04	0.05
ENSSSCG00000034609	RNF112	3.35	-2.56	1.25	-2.05	0.04	0.06
ENSSSCG00000000743	FKBP4	3062.98	-2.56	0.06	-44.33	0.00	0.00
ENSSSCG00000040888	KCTD13	474.15	-2.56	0.11	-23.28	0.00	0.00
ENSSSCG00000021036	RAB3B	14.76	-2.55	0.58	-4.40	0.00	0.00
ENSSSCG00000038191	ABT1	579.69	-2.55	0.10	-24.52	0.00	0.00
ENSSSCG00000003051	CD177	12.44	-2.55	0.63	-4.02	0.00	0.00
ENSSSCG00000031216	HS3ST6	24.95	-2.54	0.46	-5.54	0.00	0.00
ENSSSCG00000011754	GHSR	3.35	-2.54	1.19	-2.14	0.03	0.05
ENSSSCG00000003088	APOE	22.29	-2.54	0.46	-5.47	0.00	0.00
ENSSSCG00000022866	NXF1	3301.01	-2.52	0.06	-41.47	0.00	0.00
ENSSSCG00000023775	TFB2M	277.03	-2.51	0.14	-18.14	0.00	0.00
ENSSSCG00000000584	SLCO1A2	4.33	-2.51	1.06	-2.37	0.02	0.03
ENSSSCG00000024568		176.02	-2.51	0.18	-14.11	0.00	0.00
ENSSSCG00000015638	RHEX	4.36	-2.51	1.04	-2.41	0.02	0.02
ENSSSCG00000000401	GLS2	159.14	-2.51	0.19	-13.30	0.00	0.00

ENSSSCG00000038037	SPTY2D1OS	20.80	-2.51	0.48	-5.22	0.00	0.00
ENSSSCG00000035755	SHISA8	26.34	-2.50	0.43	-5.75	0.00	0.00
ENSSSCG00000019046	RF00610	18.56	-2.50	0.51	-4.90	0.00	0.00
ENSSSCG00000001440	NOTCH4	27.03	-2.49	0.44	-5.71	0.00	0.00
ENSSSCG00000000738	RHNO1	2135.82	-2.49	0.07	-34.83	0.00	0.00
ENSSSCG00000017808		8.69	-2.49	0.74	-3.37	0.00	0.00
ENSSSCG00000007453	EYA2	7.53	-2.48	0.80	-3.12	0.00	0.00
ENSSSCG00000035144	RF02138	6.56	-2.48	0.85	-2.92	0.00	0.01
ENSSSCG00000033788	OXLD1	317.14	-2.47	0.14	-17.95	0.00	0.00
ENSSSCG00000025000	SYNDIG1L	3.19	-2.45	1.20	-2.05	0.04	0.06
ENSSSCG00000031977	PTCH2	10.50	-2.45	0.67	-3.67	0.00	0.00
ENSSSCG00000012376	GDPD2	29.37	-2.44	0.40	-6.04	0.00	0.00
ENSSSCG00000037715	RF00026	6.42	-2.44	0.87	-2.79	0.01	0.01
ENSSSCG00000035998	C4orf54	75.77	-2.44	0.25	-9.62	0.00	0.00
ENSSSCG00000011604	CHCHD4	522.65	-2.43	0.11	-22.87	0.00	0.00
ENSSSCG00000002997		8.39	-2.43	0.76	-3.22	0.00	0.00
ENSSSCG00000019135	RF00278	34.80	-2.43	0.38	-6.33	0.00	0.00
ENSSSCG00000039649	C19orf84	3.20	-2.43	1.21	-2.01	0.04	0.06
ENSSSCG00000015981	HOXD10	27.38	-2.43	0.42	-5.76	0.00	0.00
ENSSSCG00000013654		15.63	-2.43	0.56	-4.33	0.00	0.00
ENSSSCG00000035360	TMEM235	13.59	-2.42	0.59	-4.11	0.00	0.00
ENSSSCG00000006582	S100A14	39.39	-2.42	0.34	-7.04	0.00	0.00
ENSSSCG00000001132	BTN1A1	6.19	-2.42	0.87	-2.79	0.01	0.01
ENSSSCG00000010100	LRRC74B	12.35	-2.42	0.62	-3.87	0.00	0.00
ENSSSCG00000010339	DYDC2	181.02	-2.42	0.17	-14.21	0.00	0.00

ENSSSCG00000037513	GINS4	1153.95	-2.42	0.08	-30.29	0.00	0.00
ENSSSCG00000001993	TGM1	60.96	-2.42	0.28	-8.54	0.00	0.00
ENSSSCG00000018372	RF00594	89.29	-2.41	0.23	-10.43	0.00	0.00
ENSSSCG00000034014	MRPL40	1562.56	-2.41	0.08	-31.67	0.00	0.00
ENSSSCG00000002738	ZNF23	28.72	-2.40	0.41	-5.86	0.00	0.00
ENSSSCG00000035971	DUSP2	41.60	-2.40	0.35	-6.92	0.00	0.00
ENSSSCG00000000092	NPTXR	131.93	-2.40	0.19	-12.33	0.00	0.00
ENSSSCG00000011439	PHF7	55.39	-2.40	0.29	-8.26	0.00	0.00
ENSSSCG00000006399	VSIG8	5.24	-2.40	0.94	-2.54	0.01	0.02
ENSSSCG00000036469		5.23	-2.40	0.94	-2.54	0.01	0.02
ENSSSCG00000015954	DLX2	55.57	-2.39	0.30	-7.94	0.00	0.00
ENSSSCG00000011936	ZBED2	25.82	-2.39	0.43	-5.53	0.00	0.00
ENSSSCG00000023599	TIMM8B	1048.92	-2.39	0.09	-26.47	0.00	0.00
ENSSSCG00000037735		84.46	-2.38	0.24	-9.78	0.00	0.00
ENSSSCG00000013501	CREB3L3	495.33	-2.38	0.11	-20.87	0.00	0.00
ENSSSCG00000029239	MZB1	9.94	-2.38	0.70	-3.41	0.00	0.00
ENSSSCG00000031924	NKX3-1	109.77	-2.38	0.21	-11.37	0.00	0.00
ENSSSCG00000001394		92.83	-2.37	0.23	-10.37	0.00	0.00
ENSSSCG00000015316		15.21	-2.37	0.56	-4.27	0.00	0.00
ENSSSCG00000035565	CCDC134	399.93	-2.37	0.12	-20.00	0.00	0.00
ENSSSCG00000032395		15.08	-2.37	0.56	-4.22	0.00	0.00
ENSSSCG00000015493	SERPINC1	4.99	-2.37	0.96	-2.48	0.01	0.02
ENSSSCG00000009621	PHYHIP	4.98	-2.36	0.97	-2.43	0.02	0.02
ENSSSCG00000025783	ENTPD3	56.13	-2.36	0.29	-8.21	0.00	0.00
ENSSSCG00000010772	ADAM8	71.21	-2.36	0.26	-8.96	0.00	0.00

ENSSSCG00000021871	FDXACB1	74.19	-2.36	0.25	-9.44	0.00	0.00
ENSSSCG00000017277	PECAM1	44.90	-2.36	0.32	-7.33	0.00	0.00
ENSSSCG00000033217		7.07	-2.35	0.82	-2.86	0.00	0.01
ENSSSCG00000018309	RF00567	5.02	-2.35	0.95	-2.49	0.01	0.02
ENSSSCG00000003608	LCK	4.02	-2.35	1.08	-2.17	0.03	0.04
ENSSSCG00000001752	CHRNA3	13.89	-2.35	0.57	-4.09	0.00	0.00
ENSSSCG00000009209	NAP1L5	217.99	-2.34	0.15	-15.50	0.00	0.00
ENSSSCG00000000398	APOF	24.67	-2.34	0.44	-5.34	0.00	0.00
ENSSSCG00000024043	ADAMTS2	8.01	-2.34	0.79	-2.96	0.00	0.00
ENSSSCG00000008603	MATN3	10.85	-2.33	0.64	-3.63	0.00	0.00
ENSSSCG00000024060	LIPT2	38.40	-2.33	0.35	-6.72	0.00	0.00
ENSSSCG00000003928	PLK3	379.67	-2.32	0.12	-19.86	0.00	0.00
ENSSSCG00000040087	SPRTN	708.28	-2.32	0.09	-24.72	0.00	0.00
ENSSSCG00000007373	GDAP1L1	13.70	-2.32	0.58	-3.98	0.00	0.00
ENSSSCG00000026323	FAM50B	4.03	-2.32	1.12	-2.08	0.04	0.05
ENSSSCG00000035923	JMJD6	700.16	-2.32	0.09	-25.59	0.00	0.00
ENSSSCG00000009062	MGARP	53.74	-2.31	0.30	-7.82	0.00	0.00
ENSSSCG00000032821	FRAT1	11.76	-2.31	0.62	-3.71	0.00	0.00
ENSSSCG00000034144		3.86	-2.31	1.11	-2.08	0.04	0.05
ENSSSCG00000016115	ICA1L	27.12	-2.31	0.43	-5.37	0.00	0.00
ENSSSCG00000017380	ARL4D	107.08	-2.31	0.21	-10.96	0.00	0.00
ENSSSCG00000037184	GPR4	173.33	-2.31	0.18	-12.76	0.00	0.00
ENSSSCG00000029567	SLC8A2	4.82	-2.30	0.96	-2.39	0.02	0.02
ENSSSCG00000025772	GPR45	3.83	-2.30	1.10	-2.09	0.04	0.05
ENSSSCG00000010006		463.88	-2.30	0.11	-21.88	0.00	0.00

ENSSSCG00000028964	PIM2	1293.27	-2.30	0.07	-31.74	0.00	0.00
ENSSSCG00000031166		112.73	-2.30	0.21	-10.76	0.00	0.00
ENSSSCG00000004776	DISP2	22.01	-2.29	0.48	-4.81	0.00	0.00
ENSSSCG00000035420	HES4	116.86	-2.29	0.20	-11.53	0.00	0.00
ENSSSCG00000021576	CD83	131.17	-2.29	0.19	-12.21	0.00	0.00
ENSSSCG00000008997	FGB	28.17	-2.29	0.41	-5.64	0.00	0.00
ENSSSCG00000017340	DCAKD	637.62	-2.28	0.09	-24.49	0.00	0.00
ENSSSCG00000018530	RF00429	6.65	-2.28	0.82	-2.79	0.01	0.01
ENSSSCG00000029776	FAM219B	461.84	-2.28	0.11	-20.58	0.00	0.00
ENSSSCG00000017068	FAXDC2	120.65	-2.28	0.20	-11.47	0.00	0.00
ENSSSCG00000021910	GAST	15.41	-2.28	0.54	-4.24	0.00	0.00
ENSSSCG00000006511	RUSC1	23.84	-2.28	0.45	-5.05	0.00	0.00
ENSSSCG00000034371		35.89	-2.27	0.39	-5.79	0.00	0.00
ENSSSCG00000014898	DDIAS	1935.16	-2.27	0.07	-33.80	0.00	0.00
ENSSSCG00000024666		16.19	-2.27	0.52	-4.33	0.00	0.00
ENSSSCG00000027100	MED31	459.35	-2.27	0.11	-21.00	0.00	0.00
ENSSSCG00000031615	SERPINB9	75.04	-2.26	0.25	-8.98	0.00	0.00
ENSSSCG00000040377	FCER2	77.59	-2.26	0.25	-9.03	0.00	0.00
ENSSSCG00000033068		947.79	-2.26	0.08	-27.13	0.00	0.00
ENSSSCG00000035742	MIR3064	45.79	-2.26	0.33	-6.83	0.00	0.00
ENSSSCG00000008347	FBXO48	306.09	-2.26	0.13	-17.34	0.00	0.00
ENSSSCG00000007007	IDO1	25.43	-2.26	0.43	-5.23	0.00	0.00
ENSSSCG00000004671	SPATA5L1	208.46	-2.25	0.15	-14.59	0.00	0.00
ENSSSCG00000011201	SATB1	6.44	-2.25	0.85	-2.64	0.01	0.01
ENSSSCG00000018734	MIR1282	42.77	-2.25	0.35	-6.40	0.00	0.00

ENSSSCG00000012986	KCNK7	29.09	-2.25	0.40	-5.68	0.00	0.00
ENSSSCG00000002855		275.74	-2.25	0.13	-16.79	0.00	0.00
ENSSSCG00000009100	TNIP3	121.41	-2.24	0.20	-11.30	0.00	0.00
ENSSSCG00000031786	NCDN	1766.16	-2.24	0.07	-32.47	0.00	0.00
ENSSSCG00000024363		36.63	-2.24	0.35	-6.41	0.00	0.00
ENSSSCG00000018563	RF00554	4.67	-2.24	0.97	-2.31	0.02	0.03
ENSSSCG00000007703	TRIM50	55.28	-2.23	0.30	-7.53	0.00	0.00
ENSSSCG00000010892	KCNT2	8.30	-2.23	0.73	-3.03	0.00	0.00
ENSSSCG00000038185	EREG	4889.70	-2.23	0.06	-38.66	0.00	0.00
ENSSSCG00000019602	RF00154	6.44	-2.22	0.87	-2.55	0.01	0.02
ENSSSCG00000006311	DUSP27	4.71	-2.22	0.98	-2.26	0.02	0.03
ENSSSCG00000013105	CCDC86	455.64	-2.22	0.12	-18.69	0.00	0.00
ENSSSCG00000015923	NOSTRIN	12.01	-2.22	0.61	-3.61	0.00	0.00
ENSSSCG00000040725	IL11	76.37	-2.22	0.27	-8.31	0.00	0.00
ENSSSCG00000034474		147.57	-2.21	0.18	-12.10	0.00	0.00
ENSSSCG00000034606	RF02271	4.49	-2.21	1.04	-2.13	0.03	0.05
ENSSSCG00000003278		49.72	-2.21	0.30	-7.34	0.00	0.00
ENSSSCG00000031844		13.73	-2.21	0.61	-3.61	0.00	0.00
ENSSSCG00000034634	ssc-mir-6782	359.04	-2.20	0.12	-18.16	0.00	0.00
ENSSSCG00000003967	ZMYND12	14.64	-2.20	0.56	-3.93	0.00	0.00
ENSSSCG00000036387	ATPSCKMT	223.72	-2.20	0.15	-14.46	0.00	0.00
ENSSSCG00000032522		3.71	-2.20	1.10	-2.00	0.05	0.06
ENSSSCG00000007362	SRSF6	3642.40	-2.20	0.14	-15.15	0.00	0.00
ENSSSCG00000036905		10.79	-2.19	0.67	-3.27	0.00	0.00
ENSSSCG00000038650	TLCD1	792.72	-2.19	0.17	-12.78	0.00	0.00

ENSSSCG00000007243	PLAGL2	1618.47	-2.19	0.07	-32.72	0.00	0.00
ENSSSCG00000036824	AVPI1	896.09	-2.19	0.09	-25.31	0.00	0.00
ENSSSCG00000037792	COX7A1	3.73	-2.19	1.11	-1.97	0.05	0.07
ENSSSCG00000034283		4.54	-2.19	0.99	-2.21	0.03	0.04
ENSSSCG00000003539	GRHL3	6.28	-2.19	0.86	-2.55	0.01	0.02
ENSSSCG00000007556	PSMG3	407.88	-2.19	0.11	-19.52	0.00	0.00
ENSSSCG00000036592	SRSF2	5427.88	-2.19	0.06	-39.68	0.00	0.00
ENSSSCG00000012967	FOSL1	870.15	-2.19	0.08	-26.32	0.00	0.00
ENSSSCG00000034297	HES2	8.23	-2.19	0.74	-2.94	0.00	0.01
ENSSSCG00000009739	NOC4L	732.49	-2.19	0.09	-23.42	0.00	0.00
ENSSSCG00000021724	KIF19	11.67	-2.18	0.61	-3.56	0.00	0.00
ENSSSCG00000037577	CHAC2	37.71	-2.18	0.35	-6.19	0.00	0.00
ENSSSCG00000039630		11.69	-2.18	0.62	-3.49	0.00	0.00
ENSSSCG00000037351		4.52	-2.18	0.99	-2.21	0.03	0.04
ENSSSCG00000015197	SPA17	369.12	-2.18	0.11	-18.92	0.00	0.00
ENSSSCG00000001987	RIPK3	26.10	-2.17	0.42	-5.14	0.00	0.00
ENSSSCG00000012912	TBC1D10C	18.66	-2.17	0.50	-4.31	0.00	0.00
ENSSSCG00000028460	S1PR5	58.94	-2.17	0.29	-7.58	0.00	0.00
ENSSSCG00000013093	VWCE	3.72	-2.17	1.10	-1.96	0.05	0.07
ENSSSCG00000031321	NR4A1	1237.42	-2.17	0.17	-13.04	0.00	0.00
ENSSSCG00000032691	ANKRD66	12.51	-2.16	0.60	-3.61	0.00	0.00
ENSSSCG00000009945	FICD	230.81	-2.16	0.14	-15.21	0.00	0.00
ENSSSCG00000030209	MFNG	26.84	-2.16	0.41	-5.22	0.00	0.00
ENSSSCG00000028019	LRRC59	3185.86	-2.16	0.06	-36.30	0.00	0.00
ENSSSCG00000024789	SRRT	4158.66	-2.16	0.05	-40.93	0.00	0.00

ENSSSCG00000039986	RGS8	17.93	-2.15	0.51	-4.19	0.00	0.00
ENSSSCG00000023591	ADGRF2	107.16	-2.15	0.22	-9.79	0.00	0.00
ENSSSCG00000032652	SLFN11	4.54	-2.15	0.98	-2.19	0.03	0.04
ENSSSCG00000002967	CAPN12	6.18	-2.15	0.85	-2.54	0.01	0.02
ENSSSCG00000007382	PABPC1L	9.97	-2.15	0.67	-3.19	0.00	0.00
ENSSSCG00000004342	NDUFAF4	174.19	-2.14	0.17	-12.86	0.00	0.00
ENSSSCG00000010162	SLC35F3	6.21	-2.14	0.84	-2.56	0.01	0.02
ENSSSCG00000040205	TLCD2	122.40	-2.14	0.20	-10.91	0.00	0.00
ENSSSCG00000039890	RASL11A	73.44	-2.14	0.26	-8.20	0.00	0.00
ENSSSCG00000006764	PTPN22	125.73	-2.14	0.20	-10.75	0.00	0.00
ENSSSCG00000007995	MCRIP2	517.46	-2.14	0.11	-20.29	0.00	0.00
ENSSSCG00000037971		16.75	-2.14	0.51	-4.18	0.00	0.00
ENSSSCG00000027676	NOP56	4115.05	-2.14	0.06	-36.24	0.00	0.00
ENSSSCG00000005439	ACTL7A	6.16	-2.13	0.84	-2.54	0.01	0.02
ENSSSCG00000007463	PTGIS	13.22	-2.13	0.57	-3.72	0.00	0.00
ENSSSCG00000000045	NDUFA6	903.69	-2.13	0.08	-25.67	0.00	0.00
ENSSSCG00000037241	RGS2	67.13	-2.13	0.26	-8.33	0.00	0.00
ENSSSCG00000003351	MMP23B	4.28	-2.13	1.02	-2.08	0.04	0.05
ENSSSCG00000032831	BRI3BP	1830.19	-2.13	0.07	-32.34	0.00	0.00
ENSSSCG00000038941	SNAPC5	36.94	-2.13	0.35	-6.07	0.00	0.00
ENSSSCG00000012494	TIMM8A	533.43	-2.12	0.11	-20.01	0.00	0.00
ENSSSCG00000037120	TK1	829.58	-2.12	0.09	-24.92	0.00	0.00
ENSSSCG00000032092	DDX5	10068.13	-2.12	0.05	-46.12	0.00	0.00
ENSSSCG00000036064	CALHM6	32.49	-2.12	0.37	-5.74	0.00	0.00
ENSSSCG00000005844	NRARP	23.56	-2.12	0.43	-4.93	0.00	0.00

ENSSSCG00000036779	RF00002	32.39	-2.12	0.37	-5.76	0.00	0.00
ENSSSCG00000022202	TOX2	6.00	-2.11	0.88	-2.40	0.02	0.02
ENSSSCG00000028452	ERAS	7.88	-2.11	0.75	-2.81	0.01	0.01
ENSSSCG00000033207	SULT2A1	33.17	-2.11	0.36	-5.81	0.00	0.00
ENSSSCG00000025589	SMIM26	311.31	-2.10	0.13	-15.69	0.00	0.00
ENSSSCG00000007231	MYLK2	19.08	-2.10	0.48	-4.36	0.00	0.00
ENSSSCG00000037912	FITM2	1387.46	-2.10	0.07	-29.85	0.00	0.00
ENSSSCG00000006095	CCNE2	1185.20	-2.10	0.08	-27.94	0.00	0.00
ENSSSCG00000013393	SPON1	19.83	-2.10	0.47	-4.49	0.00	0.00
ENSSSCG00000005385	NR4A3	338.96	-2.10	0.14	-14.77	0.00	0.00
ENSSSCG00000038965	ARC	32.94	-2.09	0.37	-5.67	0.00	0.00
ENSSSCG00000039947	KCNJ2	413.34	-2.09	0.12	-17.65	0.00	0.00
ENSSSCG00000038056	B3GALT5	39.90	-2.09	0.33	-6.31	0.00	0.00
ENSSSCG00000009474	SCEL	60.24	-2.09	0.28	-7.47	0.00	0.00
ENSSSCG00000032721	TRAF3IP3	6.04	-2.09	0.84	-2.50	0.01	0.02
ENSSSCG00000018540	RF00569	34.79	-2.09	0.36	-5.74	0.00	0.00
ENSSSCG00000014034	N4BP3	626.33	-2.09	0.10	-21.79	0.00	0.00
ENSSSCG00000018067		5.82	-2.08	0.91	-2.29	0.02	0.03
ENSSSCG00000040581	CISH	294.60	-2.08	0.13	-16.12	0.00	0.00
ENSSSCG00000032360	PANX1	464.17	-2.08	0.11	-19.25	0.00	0.00
ENSSSCG00000037473		11.30	-2.08	0.62	-3.34	0.00	0.00
ENSSSCG00000013504	TMIGD2	11.26	-2.08	0.62	-3.36	0.00	0.00
ENSSSCG00000021585	OXTR	16.44	-2.08	0.52	-4.02	0.00	0.00
ENSSSCG00000007830	RBBP6	3890.79	-2.08	0.06	-33.48	0.00	0.00
ENSSSCG00000031168	TMEM210	5.22	-2.08	0.90	-2.30	0.02	0.03

ENSSSCG00000012652	SASH3	65.90	-2.08	0.26	-8.11	0.00	0.00
ENSSSCG00000040448		71.90	-2.08	0.25	-8.37	0.00	0.00
ENSSSCG00000039443		149.23	-2.08	0.18	-11.49	0.00	0.00
ENSSSCG00000015579	PTGS2	347.97	-2.08	0.13	-16.51	0.00	0.00
ENSSSCG00000011828	FAM43A	115.84	-2.08	0.20	-10.18	0.00	0.00
ENSSSCG00000032710	E2F2	523.43	-2.07	0.11	-19.75	0.00	0.00
ENSSSCG00000016868		68.62	-2.07	0.26	-7.91	0.00	0.00
ENSSSCG00000026618	CAVIN2	74.27	-2.07	0.25	-8.40	0.00	0.00
ENSSSCG00000014920	FZD4	3221.17	-2.07	0.05	-38.26	0.00	0.00
ENSSSCG00000034870		136.63	-2.07	0.19	-10.78	0.00	0.00
ENSSSCG00000017178	SPHK1	465.65	-2.07	0.11	-19.53	0.00	0.00
ENSSSCG00000017336		583.11	-2.07	0.11	-19.27	0.00	0.00
ENSSSCG00000003922	UROD	661.35	-2.07	0.09	-22.84	0.00	0.00
ENSSSCG00000031990	DIMT1	829.44	-2.07	0.09	-23.51	0.00	0.00
ENSSSCG00000037606	PSMB3	1563.13	-2.07	0.07	-29.82	0.00	0.00
ENSSSCG00000025621	CXXC4	4.14	-2.06	1.03	-2.00	0.05	0.06
ENSSSCG00000036218		7.44	-2.06	0.77	-2.66	0.01	0.01
ENSSSCG00000005216		21.26	-2.06	0.45	-4.60	0.00	0.00
ENSSSCG00000016892	FST	62.35	-2.06	0.27	-7.71	0.00	0.00
ENSSSCG00000009775	CDK2AP1	1359.10	-2.06	0.07	-27.62	0.00	0.00
ENSSSCG00000026454	PMAIP1	1083.61	-2.05	0.08	-25.55	0.00	0.00
ENSSSCG00000007770	ORAI3	918.63	-2.05	0.09	-23.07	0.00	0.00
ENSSSCG00000015589	VASH2	133.32	-2.05	0.18	-11.17	0.00	0.00
ENSSSCG00000032343		4.17	-2.05	1.01	-2.04	0.04	0.06
ENSSSCG00000022031	CHST2	5.85	-2.05	0.86	-2.39	0.02	0.02

ENSSSCG00000020737	ZNRD2	761.54	-2.05	0.09	-22.52	0.00	0.00
ENSSSCG00000017420	CNP	1374.84	-2.05	0.07	-27.69	0.00	0.00
ENSSSCG00000032819	QPCTL	765.56	-2.05	0.08	-24.64	0.00	0.00
ENSSSCG00000021573	KCNJ5	1980.73	-2.04	0.07	-29.80	0.00	0.00
ENSSSCG00000014157	NR2F1	73.04	-2.04	0.25	-8.06	0.00	0.00
ENSSSCG00000023460	HPCAL4	516.44	-2.04	0.10	-20.92	0.00	0.00
ENSSSCG00000006481	GPATCH4	229.03	-2.04	0.14	-14.36	0.00	0.00
ENSSSCG00000024313		2288.49	-2.04	0.06	-31.70	0.00	0.00
ENSSSCG00000011561	PRRT3	30.27	-2.04	0.38	-5.35	0.00	0.00
ENSSSCG00000019949	RF00138	11.08	-2.04	0.63	-3.22	0.00	0.00
ENSSSCG00000027875	CA14	7.47	-2.04	0.80	-2.56	0.01	0.02
ENSSSCG00000017471	CDC6	1200.65	-2.04	0.07	-29.18	0.00	0.00
ENSSSCG00000009844	HSPB8	1120.80	-2.03	0.08	-26.66	0.00	0.00
ENSSSCG00000023441	KLHDC8A	27.55	-2.03	0.40	-5.05	0.00	0.00
ENSSSCG00000030428	TWNK	1247.85	-2.03	0.07	-27.52	0.00	0.00
ENSSSCG00000006546	TDRD10	5.82	-2.03	0.86	-2.37	0.02	0.03
ENSSSCG00000034543	RF00026	6.65	-2.03	0.81	-2.51	0.01	0.02
ENSSSCG00000028010	ABHD3	744.03	-2.03	0.08	-23.88	0.00	0.00
ENSSSCG00000018094	CYTB	10506.25	-2.03	0.26	-7.86	0.00	0.00
ENSSSCG00000004928	CILP	4.19	-2.03	1.00	-2.04	0.04	0.06
ENSSSCG00000017605	MMD	479.56	-2.02	0.10	-20.00	0.00	0.00
ENSSSCG00000024622	ZNF667	8.47	-2.02	0.73	-2.76	0.01	0.01
ENSSSCG00000035473		8.41	-2.02	0.74	-2.73	0.01	0.01
ENSSSCG00000023177	RRP9	460.66	-2.02	0.10	-19.30	0.00	0.00
ENSSSCG00000035628	CCDC115	720.00	-2.02	0.09	-22.00	0.00	0.00

ENSSSCG00000012493	TAF7L	6.71	-2.02	0.80	-2.53	0.01	0.02
ENSSSCG00000023162	CDH3	10.65	-2.02	0.63	-3.19	0.00	0.00
ENSSSCG00000013110	TMEM109	516.37	-2.01	0.11	-18.17	0.00	0.00
ENSSSCG00000037600	C1orf216	20.74	-2.01	0.46	-4.38	0.00	0.00
ENSSSCG00000017295	KCNH6	12.41	-2.01	0.60	-3.36	0.00	0.00
ENSSSCG00000029077	TUBAL3	11.53	-2.01	0.60	-3.32	0.00	0.00
ENSSSCG00000022187		980.88	-2.01	0.08	-23.94	0.00	0.00
ENSSSCG00000036582	MTLN	160.18	-2.01	0.17	-11.89	0.00	0.00
ENSSSCG00000006640	BNIPL	30.52	-2.00	0.37	-5.35	0.00	0.00
ENSSSCG00000038677	GJB3	200.47	-2.00	0.15	-13.10	0.00	0.00
ENSSSCG00000036960	NOP10	75.62	-2.00	0.25	-8.16	0.00	0.00
ENSSSCG00000014278	IL5	45.74	-2.00	0.31	-6.41	0.00	0.00
ENSSSCG00000037063	PROKR2	498.26	-2.00	0.10	-20.33	0.00	0.00
ENSSSCG00000032709	ARL4A	353.98	-2.00	0.12	-17.14	0.00	0.00
ENSSSCG00000011703	TM4SF4	3818.62	-1.99	0.06	-33.58	0.00	0.00
ENSSSCG00000001392	TCF19	388.93	-1.99	0.11	-17.75	0.00	0.00
ENSSSCG00000034765		4.83	-1.99	0.94	-2.12	0.03	0.05
ENSSSCG00000037016	ID1	689.36	-1.99	0.10	-19.57	0.00	0.00
ENSSSCG00000030371		22.91	-1.99	0.43	-4.58	0.00	0.00
ENSSSCG00000028372	FAM131A	69.73	-1.99	0.25	-7.96	0.00	0.00
ENSSSCG00000026233		287.12	-1.99	0.13	-14.75	0.00	0.00
ENSSSCG00000038888	C1orf122	191.37	-1.99	0.15	-12.93	0.00	0.00
ENSSSCG00000007741	NUPR2	5.72	-1.98	0.86	-2.31	0.02	0.03
ENSSSCG00000015126	TRIM29	24.95	-1.98	0.42	-4.70	0.00	0.00
ENSSSCG00000030195		4.86	-1.98	0.93	-2.14	0.03	0.04

ENSSSCG00000025560	PGLYRP2	199.45	-1.98	0.15	-13.18	0.00	0.00
ENSSSCG00000035833	NKPD1	7.40	-1.98	0.79	-2.52	0.01	0.02
ENSSSCG00000034653	DLX4	50.84	-1.98	0.30	-6.60	0.00	0.00
ENSSSCG00000032261	CCDC117	1984.94	-1.98	0.07	-29.82	0.00	0.00
ENSSSCG00000009997	OSM	7.42	-1.98	0.82	-2.42	0.02	0.02
ENSSSCG00000004046	PNLDC1	6.56	-1.97	0.80	-2.48	0.01	0.02
ENSSSCG00000017549	PHB	3852.37	-1.97	0.06	-34.77	0.00	0.00
ENSSSCG00000034812	EFCAB8	42.71	-1.97	0.32	-6.24	0.00	0.00
ENSSSCG00000006919		11.43	-1.97	0.62	-3.19	0.00	0.00
ENSSSCG00000011690	PLSCR4	337.49	-1.97	0.12	-16.42	0.00	0.00
ENSSSCG00000014825	RELT	33.74	-1.97	0.35	-5.56	0.00	0.00
ENSSSCG00000016808	C5orf22	266.73	-1.97	0.14	-14.59	0.00	0.00
ENSSSCG00000030247	EPM2AIP1	951.44	-1.97	0.08	-25.39	0.00	0.00
ENSSSCG00000026446		1885.20	-1.97	0.07	-28.98	0.00	0.00
ENSSSCG00000019590	RF00443	8.03	-1.97	0.72	-2.74	0.01	0.01
ENSSSCG00000006463	PEAR1	16.86	-1.97	0.50	-3.95	0.00	0.00
ENSSSCG00000007268	E2F1	898.93	-1.97	0.08	-24.07	0.00	0.00
ENSSSCG00000010161	COA6	154.54	-1.96	0.17	-11.49	0.00	0.00
ENSSSCG00000007436	MMP9	8.89	-1.96	0.69	-2.86	0.00	0.01
ENSSSCG00000029311	MYPN	44.18	-1.96	0.31	-6.36	0.00	0.00
ENSSSCG00000023323		948.77	-1.96	0.08	-23.09	0.00	0.00
ENSSSCG00000029577		703.24	-1.96	0.09	-20.96	0.00	0.00
ENSSSCG00000001347	PPP1R10	1025.53	-1.96	0.10	-20.05	0.00	0.00
ENSSSCG00000017901	INCA1	18.42	-1.95	0.48	-4.11	0.00	0.00
ENSSSCG00000001636		7.17	-1.95	0.76	-2.58	0.01	0.01

ENSSSCG00000036437	NOG	5.42	-1.95	0.90	-2.16	0.03	0.04
ENSSSCG00000019428	RF00213	48.58	-1.95	0.30	-6.46	0.00	0.00
ENSSSCG00000015116		312.94	-1.95	0.12	-15.59	0.00	0.00
ENSSSCG00000004554	PCLAF	3145.57	-1.95	0.13	-15.49	0.00	0.00
ENSSSCG00000031442	CLP1	576.02	-1.95	0.10	-18.57	0.00	0.00
ENSSSCG00000021638	NEU3	309.51	-1.95	0.12	-16.00	0.00	0.00
ENSSSCG00000033479	NEUROG2	35.50	-1.94	0.34	-5.63	0.00	0.00
ENSSSCG00000038348	NME1	982.18	-1.94	0.08	-24.55	0.00	0.00
ENSSSCG00000006400	SLAMF9	5.54	-1.94	0.87	-2.24	0.02	0.04
ENSSSCG00000000234	GRASP	70.04	-1.94	0.26	-7.55	0.00	0.00
ENSSSCG00000023684	MT1A	192.39	-1.94	0.15	-12.50	0.00	0.00
ENSSSCG00000037832	PMP22	34.58	-1.94	0.35	-5.49	0.00	0.00
ENSSSCG00000010103	TUBA3D	7.89	-1.93	0.73	-2.66	0.01	0.01
ENSSSCG00000028802		6.34	-1.93	0.88	-2.19	0.03	0.04
ENSSSCG00000034239	ZNF189	604.36	-1.93	0.10	-19.17	0.00	0.00
ENSSSCG00000006204	PPP1R42	8.68	-1.93	0.69	-2.81	0.01	0.01
ENSSSCG00000029991	SNIP1	606.61	-1.93	0.10	-19.36	0.00	0.00
ENSSSCG00000016706	HOXA2	7.00	-1.92	0.77	-2.50	0.01	0.02
ENSSSCG00000033526	BAG2	274.84	-1.92	0.14	-14.18	0.00	0.00
ENSSSCG00000004918	ALPK2	4.67	-1.92	0.94	-2.05	0.04	0.06
ENSSSCG00000005925	EXOSC4	253.25	-1.92	0.14	-14.10	0.00	0.00
ENSSSCG00000031098	DBR1	494.99	-1.92	0.10	-18.93	0.00	0.00
ENSSSCG00000037575	FKBP14	424.07	-1.92	0.11	-17.79	0.00	0.00
ENSSSCG00000039235	PSMC4	2415.92	-1.92	0.06	-33.15	0.00	0.00
ENSSSCG00000001099	CMAH	6.17	-1.92	0.86	-2.23	0.03	0.04

ENSSSCG00000018076		23.43	-1.91	0.42	-4.51	0.00	0.00
ENSSSCG00000029284	NPHS1	9.63	-1.91	0.69	-2.77	0.01	0.01
ENSSSCG00000005661	ENDOG	71.27	-1.91	0.24	-7.81	0.00	0.00
ENSSSCG00000000774	USP18	24.93	-1.91	0.41	-4.64	0.00	0.00
ENSSSCG00000026931	SERTAD1	543.43	-1.91	0.10	-18.91	0.00	0.00
ENSSSCG00000034190		2171.65	-1.91	0.06	-32.09	0.00	0.00
ENSSSCG00000033614	RF00604	28.01	-1.91	0.40	-4.75	0.00	0.00
ENSSSCG00000010449	CH25H	9.45	-1.90	0.68	-2.81	0.00	0.01
ENSSSCG00000015030	DLAT	5727.19	-1.90	0.05	-36.28	0.00	0.00
ENSSSCG00000013361	SPTY2D1	2622.45	-1.90	0.06	-31.92	0.00	0.00
ENSSSCG00000032063	THEM6	34.77	-1.90	0.35	-5.37	0.00	0.00
ENSSSCG00000030493	UTP23	494.52	-1.90	0.11	-17.76	0.00	0.00
ENSSSCG00000010338	DYDC1	117.04	-1.90	0.20	-9.50	0.00	0.00
ENSSSCG00000011196	DPH3	936.13	-1.90	0.08	-25.18	0.00	0.00
ENSSSCG00000017414	KAT2A	1835.77	-1.89	0.07	-26.61	0.00	0.00
ENSSSCG00000017589	DLX3	7.72	-1.89	0.73	-2.59	0.01	0.01
ENSSSCG00000002838	ZNF423	5.34	-1.89	0.87	-2.16	0.03	0.04
ENSSSCG00000010929	CYB5R1	81.86	-1.89	0.23	-8.24	0.00	0.00
ENSSSCG00000040745	ZNF771	102.94	-1.89	0.20	-9.26	0.00	0.00
ENSSSCG00000003709	LAMA3	4034.98	-1.88	0.05	-34.63	0.00	0.00
ENSSSCG00000026590	HGH1	476.10	-1.88	0.11	-17.84	0.00	0.00
ENSSSCG00000028483	RF00133	7.77	-1.88	0.80	-2.36	0.02	0.03
ENSSSCG00000022849	IL2RA	26.89	-1.88	0.40	-4.70	0.00	0.00
ENSSSCG00000012133	ASB11	6.84	-1.88	0.79	-2.38	0.02	0.03
ENSSSCG00000005007	DNAAF2	451.03	-1.88	0.11	-17.17	0.00	0.00

ENSSSCG00000006458	FCRL4	5.35	-1.88	0.86	-2.17	0.03	0.04
ENSSSCG00000002805	CFAP20	925.32	-1.88	0.08	-23.83	0.00	0.00
ENSSSCG00000000136	CSF2RB	26.11	-1.88	0.40	-4.72	0.00	0.00
ENSSSCG00000012968	CCDC85B	174.13	-1.88	0.16	-11.94	0.00	0.00
ENSSSCG00000033926	NUDT1	735.95	-1.87	0.09	-21.91	0.00	0.00
ENSSSCG00000014967	FAM76B	803.45	-1.87	0.09	-21.63	0.00	0.00
ENSSSCG00000038308	RANBP1	3473.34	-1.87	0.07	-28.64	0.00	0.00
ENSSSCG00000004411	ZBTB24	433.68	-1.87	0.11	-17.65	0.00	0.00
ENSSSCG00000015953	DLX1	104.17	-1.87	0.20	-9.26	0.00	0.00
ENSSSCG00000035993	C1QTNF2	8.36	-1.86	0.70	-2.65	0.01	0.01
ENSSSCG00000009938	UNG	326.37	-1.86	0.13	-14.30	0.00	0.00
ENSSSCG00000001572	FGD2	32.32	-1.86	0.37	-5.09	0.00	0.00
ENSSSCG00000031963	SAXO2	249.32	-1.86	0.14	-13.77	0.00	0.00
ENSSSCG00000032328	MAP6D1	274.96	-1.86	0.13	-13.94	0.00	0.00
ENSSSCG00000015413	FGL2	37.85	-1.86	0.33	-5.66	0.00	0.00
ENSSSCG00000007212	C20orf96	8.30	-1.86	0.70	-2.64	0.01	0.01
ENSSSCG00000037431		650.49	-1.86	0.09	-20.03	0.00	0.00
ENSSSCG00000031938	NEDD8	744.85	-1.86	0.08	-22.28	0.00	0.00
ENSSSCG00000029412	RF00319	26.62	-1.85	0.40	-4.67	0.00	0.00
ENSSSCG00000007713	BUD23	1998.10	-1.85	0.06	-28.58	0.00	0.00
ENSSSCG00000003379	KLHL21	1001.96	-1.85	0.08	-23.82	0.00	0.00
ENSSSCG00000019556	RF00100	115.54	-1.85	0.45	-4.11	0.00	0.00
ENSSSCG00000013352	E2F8	1112.27	-1.85	0.07	-25.84	0.00	0.00
ENSSSCG00000038436	HYPK	1273.26	-1.85	0.07	-25.05	0.00	0.00
ENSSSCG00000013008	CDCA5	2165.81	-1.85	0.07	-25.71	0.00	0.00

ENSSSCG00000006252	TMEM68	603.32	-1.85	0.09	-20.05	0.00	0.00
ENSSSCG00000040135		5.40	-1.85	0.89	-2.08	0.04	0.05
ENSSSCG00000002783	SLC9A5	455.35	-1.85	0.11	-17.01	0.00	0.00
ENSSSCG00000015436	CCDC71L	24.93	-1.85	0.41	-4.56	0.00	0.00
ENSSSCG00000004464	TENT5A	1563.09	-1.85	0.07	-27.37	0.00	0.00
ENSSSCG00000015395	GRM3	7.50	-1.84	0.73	-2.53	0.01	0.02
ENSSSCG00000002917	NFKBID	601.59	-1.84	0.09	-19.55	0.00	0.00
ENSSSCG00000040057	EOMES	9.73	-1.84	0.66	-2.78	0.01	0.01
ENSSSCG00000012141	CA5B	17.32	-1.84	0.51	-3.63	0.00	0.00
ENSSSCG00000037536	SLC25A28	558.18	-1.84	0.10	-18.47	0.00	0.00
ENSSSCG00000005896	LRRC24	94.04	-1.84	0.23	-8.06	0.00	0.00
ENSSSCG00000031446		6.09	-1.83	0.83	-2.22	0.03	0.04
ENSSSCG00000015664		1423.60	-1.83	0.07	-27.18	0.00	0.00
ENSSSCG00000011538	LMCD1	15.75	-1.83	0.51	-3.62	0.00	0.00
ENSSSCG00000022693	LBX1	32.06	-1.83	0.36	-5.12	0.00	0.00
ENSSSCG00000024588		133.46	-1.83	0.18	-10.00	0.00	0.00
ENSSSCG00000016827	BRIX1	1885.51	-1.83	0.06	-30.03	0.00	0.00
ENSSSCG00000002828	LPCAT2	17.10	-1.83	0.50	-3.64	0.00	0.00
ENSSSCG00000038701	EFNA2	117.47	-1.83	0.19	-9.73	0.00	0.00
ENSSSCG00000016698	HOXA11	11.21	-1.83	0.60	-3.05	0.00	0.00
ENSSSCG00000011205	EFHB	7.50	-1.82	0.79	-2.31	0.02	0.03
ENSSSCG00000014956	ANKRD49	674.39	-1.82	0.09	-20.54	0.00	0.00
ENSSSCG00000020533	RF00410	21.77	-1.82	0.45	-4.09	0.00	0.00
ENSSSCG00000007084	BFSP1	49.65	-1.82	0.30	-6.14	0.00	0.00
ENSSSCG00000039662	MRPL51	693.82	-1.82	0.09	-20.01	0.00	0.00

ENSSSCG00000000696		2291.70	-1.82	0.07	-25.22	0.00	0.00
ENSSSCG00000038459	SURF2	760.40	-1.82	0.09	-20.81	0.00	0.00
ENSSSCG00000007312	SCAND1	1338.31	-1.82	0.07	-24.90	0.00	0.00
ENSSSCG00000020876	RTL5	140.24	-1.82	0.17	-10.52	0.00	0.00
ENSSSCG00000009334	HSPH1	1839.74	-1.82	0.06	-29.13	0.00	0.00
ENSSSCG00000036608		29.63	-1.82	0.39	-4.70	0.00	0.00
ENSSSCG00000036593		756.23	-1.81	0.10	-18.96	0.00	0.00
ENSSSCG00000009798	B3GNT4	24.85	-1.81	0.42	-4.31	0.00	0.00
ENSSSCG00000013772	ASF1B	1015.18	-1.80	0.08	-21.29	0.00	0.00
ENSSSCG00000002627	GSTA4	1910.26	-1.80	0.07	-25.46	0.00	0.00
ENSSSCG00000039875	NKD1	5.16	-1.80	0.89	-2.02	0.04	0.06
ENSSSCG00000030167	SLC25A39	4644.44	-1.80	0.05	-37.33	0.00	0.00
ENSSSCG00000027911	LTB4R	13.79	-1.80	0.54	-3.32	0.00	0.00
ENSSSCG00000017730	CDK5R1	145.64	-1.80	0.17	-10.44	0.00	0.00
ENSSSCG00000034163	PLN	5.24	-1.80	0.89	-2.03	0.04	0.06
ENSSSCG00000015504	CACYBP	2812.66	-1.80	0.06	-28.60	0.00	0.00
ENSSSCG00000039950	RMI1	268.42	-1.80	0.13	-13.79	0.00	0.00
ENSSSCG00000011500	KBTBD8	236.63	-1.80	0.14	-12.89	0.00	0.00
ENSSSCG00000010746	ADAM12	20.57	-1.80	0.44	-4.07	0.00	0.00
ENSSSCG00000031398		6.44	-1.79	0.82	-2.19	0.03	0.04
ENSSSCG00000036787	APOLD1	36.87	-1.79	0.35	-5.19	0.00	0.00
ENSSSCG00000038726	RANGRF	169.82	-1.79	0.16	-11.02	0.00	0.00
ENSSSCG00000011893	COX17	759.02	-1.79	0.09	-19.94	0.00	0.00
ENSSSCG00000025486	MDH2	11000.03	-1.79	0.05	-38.13	0.00	0.00
ENSSSCG00000008309		1680.56	-1.79	0.07	-26.96	0.00	0.00

ENSSSCG00000024802	MACC1	8.02	-1.79	0.70	-2.54	0.01	0.02
ENSSSCG00000004503	LOXHD1	23.06	-1.79	0.42	-4.21	0.00	0.00
ENSSSCG00000002268	AKAP5	106.64	-1.78	0.21	-8.47	0.00	0.00
ENSSSCG00000018065	ND1	4117.41	-1.78	0.25	-7.11	0.00	0.00
ENSSSCG00000033420	ZNF227	5.84	-1.78	0.82	-2.17	0.03	0.04
ENSSSCG00000039700	RF00604	16.73	-1.78	0.49	-3.63	0.00	0.00
ENSSSCG00000018078	COX2	10796.62	-1.77	0.17	-10.64	0.00	0.00
ENSSSCG00000024132	TMEM47	34.08	-1.77	0.34	-5.18	0.00	0.00
ENSSSCG00000008130	CIAO1	2930.71	-1.77	0.06	-30.72	0.00	0.00
ENSSSCG00000007149	SPEF1	323.53	-1.77	0.12	-14.80	0.00	0.00
ENSSSCG00000022175		10.22	-1.77	0.63	-2.81	0.00	0.01
ENSSSCG00000007475	MOCS3	437.95	-1.77	0.10	-16.85	0.00	0.00
ENSSSCG00000039500		206.38	-1.77	0.15	-12.16	0.00	0.00
ENSSSCG00000007704	NSUN5	614.68	-1.76	0.09	-19.64	0.00	0.00
ENSSSCG00000037866	MAGEA10	17.41	-1.76	0.48	-3.67	0.00	0.00
ENSSSCG00000004369	PRDM1	691.02	-1.76	0.10	-18.44	0.00	0.00
ENSSSCG00000036063	LPAR6	264.95	-1.76	0.13	-13.88	0.00	0.00
ENSSSCG00000008035		722.83	-1.76	0.10	-18.51	0.00	0.00
ENSSSCG00000015252	SNX19	856.66	-1.76	0.08	-22.78	0.00	0.00
ENSSSCG00000022163	GNAL	15.98	-1.76	0.51	-3.47	0.00	0.00
ENSSSCG00000005267	ANXA1	32228.95	-1.76	0.06	-28.75	0.00	0.00
ENSSSCG00000018612	RF00574	11.29	-1.76	0.61	-2.90	0.00	0.01
ENSSSCG00000004705	MAP1A	416.18	-1.76	0.11	-16.15	0.00	0.00
ENSSSCG00000033648		12.23	-1.76	0.57	-3.07	0.00	0.00
ENSSSCG00000026784	LMNB2	1529.56	-1.76	0.07	-24.21	0.00	0.00

ENSSSCG00000005576	ZBTB6	188.93	-1.76	0.15	-11.67	0.00	0.00
ENSSSCG00000036768	PRAG1	115.74	-1.75	0.20	-8.75	0.00	0.00
ENSSSCG00000032196		4.99	-1.75	0.89	-1.96	0.05	0.07
ENSSSCG00000031111	CBR1	9.45	-1.75	0.68	-2.57	0.01	0.01
ENSSSCG00000027974		334.01	-1.75	0.12	-15.15	0.00	0.00
ENSSSCG00000036314	HHEX	558.61	-1.75	0.10	-18.16	0.00	0.00
ENSSSCG00000005662	SPOUT1	552.92	-1.75	0.10	-18.09	0.00	0.00
ENSSSCG00000034398	C8orf37	1064.26	-1.74	0.08	-22.75	0.00	0.00
ENSSSCG00000009366	STOML3	138.65	-1.74	0.18	-9.65	0.00	0.00
ENSSSCG00000038534	TSR1	3000.97	-1.74	0.06	-30.53	0.00	0.00
ENSSSCG00000019093	RF00138	9.37	-1.74	0.66	-2.64	0.01	0.01
ENSSSCG00000037254	RNPC3	690.01	-1.74	0.09	-19.93	0.00	0.00
ENSSSCG00000009051	IL15	50.64	-1.74	0.29	-5.97	0.00	0.00
ENSSSCG00000017160	CBX4	849.10	-1.74	0.09	-18.92	0.00	0.00
ENSSSCG00000040685		445.45	-1.74	0.10	-17.03	0.00	0.00
ENSSSCG00000016078	HSPE1	2556.45	-1.74	0.06	-30.02	0.00	0.00
ENSSSCG00000030278	MLLT11	294.90	-1.73	0.14	-12.77	0.00	0.00
ENSSSCG00000036956		370.71	-1.73	0.11	-15.61	0.00	0.00
ENSSSCG00000007983	PDIA2	12.29	-1.73	0.59	-2.96	0.00	0.00
ENSSSCG00000012844	SLC25A22	1415.73	-1.73	0.07	-23.55	0.00	0.00
ENSSSCG00000015826		1157.00	-1.73	0.08	-21.93	0.00	0.00
ENSSSCG00000032341	AIF1	35.21	-1.73	0.34	-5.13	0.00	0.00
ENSSSCG00000017810	FAM57A	1652.18	-1.73	0.07	-26.46	0.00	0.00
ENSSSCG00000011372	IMPDH2	26.91	-1.73	0.39	-4.46	0.00	0.00
ENSSSCG00000038190	MPST	211.27	-1.72	0.15	-11.65	0.00	0.00

ENSSSCG00000012733		11.46	-1.72	0.62	-2.78	0.01	0.01
ENSSSCG00000016441		2276.66	-1.72	0.06	-27.45	0.00	0.00
ENSSSCG00000011465		674.98	-1.72	0.09	-19.42	0.00	0.00
ENSSSCG00000036914	C9orf116	133.01	-1.71	0.18	-9.45	0.00	0.00
ENSSSCG00000040773	TOB1	1461.49	-1.71	0.07	-25.37	0.00	0.00
ENSSSCG00000037096	TMEM88	22.92	-1.71	0.44	-3.86	0.00	0.00
ENSSSCG00000027455	SLC39A6	2696.34	-1.71	0.06	-30.96	0.00	0.00
ENSSSCG00000039034	DNALI1	160.40	-1.71	0.17	-9.89	0.00	0.00
ENSSSCG00000037238	DBF4B	434.35	-1.71	0.11	-16.25	0.00	0.00
ENSSSCG00000036220	CTXN1	103.82	-1.71	0.22	-7.92	0.00	0.00
ENSSSCG00000014934	CHORDC1	2226.23	-1.71	0.06	-28.09	0.00	0.00
ENSSSCG00000022784	PLEKHO1	1255.23	-1.71	0.07	-23.55	0.00	0.00
ENSSSCG00000015450	ZNF786	517.72	-1.71	0.10	-17.00	0.00	0.00
ENSSSCG00000022015	RF00604	14.11	-1.70	0.54	-3.17	0.00	0.00
ENSSSCG00000012519	GPRASP1	52.59	-1.70	0.28	-6.12	0.00	0.00
ENSSSCG00000017476	MSL1	1877.56	-1.70	0.07	-26.09	0.00	0.00
ENSSSCG00000034716	CCDC137	390.07	-1.70	0.11	-15.18	0.00	0.00
ENSSSCG00000037739	RF00056	6.84	-1.70	0.80	-2.11	0.03	0.05
ENSSSCG00000035472	TTC9C	1004.65	-1.70	0.07	-22.76	0.00	0.00
ENSSSCG00000002037	CDH24	808.97	-1.70	0.08	-20.76	0.00	0.00
ENSSSCG00000040857		4115.22	-1.69	0.05	-31.65	0.00	0.00
ENSSSCG00000004584	GCNT3	10.58	-1.69	0.62	-2.72	0.01	0.01
ENSSSCG00000039206	UCN	32.78	-1.69	0.35	-4.77	0.00	0.00
ENSSSCG00000000411	NAB2	336.59	-1.69	0.11	-14.72	0.00	0.00
ENSSSCG00000033669	RDH10	551.10	-1.69	0.10	-17.62	0.00	0.00

ENSSSCG00000033731	SLC35B1	938.90	-1.69	0.07	-22.74	0.00	0.00
ENSSSCG00000028132	NAA20	2376.94	-1.69	0.06	-28.63	0.00	0.00
ENSSSCG00000014957	C11orf97	423.74	-1.69	0.10	-16.44	0.00	0.00
ENSSSCG00000025674	SNRNP27	1017.23	-1.69	0.08	-21.76	0.00	0.00
ENSSSCG00000028609	SLC12A4	2183.95	-1.69	0.06	-29.85	0.00	0.00
ENSSSCG00000008165	RFX8	13.94	-1.69	0.54	-3.12	0.00	0.00
ENSSSCG00000030119	PRPF3	1350.22	-1.68	0.07	-25.38	0.00	0.00
ENSSSCG00000001042	MAK	381.49	-1.68	0.11	-15.41	0.00	0.00
ENSSSCG00000025856	TMEM106A	260.43	-1.68	0.13	-12.87	0.00	0.00
ENSSSCG00000014074	GFM2	1042.68	-1.68	0.08	-21.50	0.00	0.00
ENSSSCG00000009904	DYNLL1	2839.21	-1.68	0.07	-24.48	0.00	0.00
ENSSSCG00000026587	BATF3	112.17	-1.68	0.19	-8.63	0.00	0.00
ENSSSCG00000016620	TSPAN12	692.29	-1.68	0.09	-18.67	0.00	0.00
ENSSSCG00000015590	FLVCR1	1659.22	-1.68	0.07	-23.54	0.00	0.00
ENSSSCG00000023903	ATP5ME	871.70	-1.68	0.08	-21.42	0.00	0.00
ENSSSCG00000001560	C6orf222	93.65	-1.68	0.21	-7.81	0.00	0.00
ENSSSCG00000000377		5481.60	-1.68	0.06	-25.90	0.00	0.00
ENSSSCG00000034963	IQCH	48.80	-1.67	0.29	-5.87	0.00	0.00
ENSSSCG00000034752	SUPT4H1	1693.67	-1.67	0.06	-26.19	0.00	0.00
ENSSSCG00000008553	PREB	889.04	-1.67	0.09	-18.30	0.00	0.00
ENSSSCG00000010322	ZNF503	16.91	-1.67	0.49	-3.43	0.00	0.00
ENSSSCG00000024245	AIMP2	37.61	-1.67	0.33	-5.03	0.00	0.00
ENSSSCG00000026701		76.02	-1.67	0.25	-6.79	0.00	0.00
ENSSSCG00000038455	RBM24	13.74	-1.67	0.54	-3.09	0.00	0.00
ENSSSCG00000012620	NKRF	960.43	-1.67	0.08	-21.50	0.00	0.00

ENSSSCG00000033018	TM4SF1	4914.29	-1.67	0.06	-28.45	0.00	0.00
ENSSSCG00000008898		118.78	-1.67	0.19	-8.93	0.00	0.00
ENSSSCG00000003169	PIH1D1	804.40	-1.67	0.09	-18.13	0.00	0.00
ENSSSCG00000025766	SLC10A3	1117.05	-1.67	0.08	-21.56	0.00	0.00
ENSSSCG00000021494	CDC25A	642.70	-1.67	0.09	-18.95	0.00	0.00
ENSSSCG00000020808		8483.71	-1.66	0.05	-34.87	0.00	0.00
ENSSSCG00000000214	GPD1	291.19	-1.66	0.12	-13.60	0.00	0.00
ENSSSCG00000024312	ID4	195.05	-1.66	0.15	-10.90	0.00	0.00
ENSSSCG00000003451		4034.60	-1.66	0.05	-30.83	0.00	0.00
ENSSSCG00000000116	POLR2F	1102.12	-1.66	0.08	-21.43	0.00	0.00
ENSSSCG00000033303	MBD4	843.83	-1.66	0.09	-19.42	0.00	0.00
ENSSSCG00000015550	RGS16	29.32	-1.66	0.38	-4.40	0.00	0.00
ENSSSCG00000037053	AK6	110.29	-1.66	0.20	-8.38	0.00	0.00
ENSSSCG00000029050	MRPL55	918.93	-1.66	0.08	-20.21	0.00	0.00
ENSSSCG00000011449	GNL3	1859.35	-1.66	0.06	-26.42	0.00	0.00
ENSSSCG00000017788	TP53I13	704.99	-1.66	0.10	-16.65	0.00	0.00
ENSSSCG00000013753	IER2	590.82	-1.66	0.11	-15.52	0.00	0.00
ENSSSCG00000004163	BCLAF1	4814.91	-1.66	0.05	-30.49	0.00	0.00
ENSSSCG00000014952	IZUMO1R	17.75	-1.66	0.47	-3.52	0.00	0.00
ENSSSCG00000033682	CPLX2	12.89	-1.66	0.56	-2.98	0.00	0.00
ENSSSCG00000021757		9.58	-1.66	0.65	-2.56	0.01	0.02
ENSSSCG00000002374	DLST	4860.14	-1.66	0.06	-29.74	0.00	0.00
ENSSSCG00000005704	EXOSC2	919.55	-1.66	0.09	-19.14	0.00	0.00
ENSSSCG00000034146	CBX8	331.32	-1.65	0.12	-13.91	0.00	0.00
ENSSSCG00000015982	HOXD9	151.86	-1.65	0.17	-9.56	0.00	0.00

ENSSSCG00000002050	DAD1	1678.97	-1.65	0.07	-22.29	0.00	0.00
ENSSSCG00000017626		3058.67	-1.65	0.06	-29.81	0.00	0.00
ENSSSCG00000032980		44.20	-1.65	0.30	-5.48	0.00	0.00
ENSSSCG00000035741	TOR1AIP2	4156.10	-1.65	0.05	-34.36	0.00	0.00
ENSSSCG00000013436		746.49	-1.65	0.08	-19.84	0.00	0.00
ENSSSCG00000002535	HSP90AA1	41797.19	-1.65	0.04	-38.51	0.00	0.00
ENSSSCG00000010224	EGR2	64.18	-1.65	0.25	-6.54	0.00	0.00
ENSSSCG00000040037	MTSS2	411.31	-1.65	0.11	-15.29	0.00	0.00
ENSSSCG00000015056	REXO2	3153.54	-1.64	0.06	-25.68	0.00	0.00
ENSSSCG00000007046	TRMT6	1913.27	-1.64	0.07	-23.90	0.00	0.00
ENSSSCG00000034779	NT5C3B	806.19	-1.64	0.10	-15.94	0.00	0.00
ENSSSCG00000009151	CYP2U1	494.93	-1.64	0.10	-15.66	0.00	0.00
ENSSSCG00000031352	TCTEX1D1	12.20	-1.64	0.56	-2.91	0.00	0.01
ENSSSCG00000029752	C16orf54	184.45	-1.64	0.16	-10.03	0.00	0.00
ENSSSCG00000030642	PCNA	3683.08	-1.64	0.05	-33.52	0.00	0.00
ENSSSCG00000017419	DNAJC7	1542.64	-1.64	0.07	-22.94	0.00	0.00
ENSSSCG00000027307	NDUFA11	670.91	-1.64	0.09	-18.18	0.00	0.00
ENSSSCG00000006788	ADORA3	18.23	-1.64	0.47	-3.50	0.00	0.00
ENSSSCG00000023562	SNRPE	824.48	-1.64	0.08	-21.01	0.00	0.00
ENSSSCG00000001683	POLH	1503.93	-1.64	0.06	-26.15	0.00	0.00
ENSSSCG00000028537		7.89	-1.64	0.76	-2.15	0.03	0.04
ENSSSCG00000035666	C20orf27	1696.47	-1.64	0.06	-25.90	0.00	0.00
ENSSSCG00000039650	PCYT2	715.77	-1.64	0.09	-17.98	0.00	0.00
ENSSSCG00000009135	LRIT3	5.99	-1.63	0.80	-2.03	0.04	0.06
ENSSSCG00000023013	RF00412	29.51	-1.63	0.37	-4.44	0.00	0.00

ENSSSCG00000015649	DYRK3	750.32	-1.63	0.09	-19.02	0.00	0.00
ENSSSCG00000004049	ACAT2	1245.38	-1.63	0.08	-21.11	0.00	0.00
ENSSSCG00000032188	METTL26	795.67	-1.63	0.08	-20.03	0.00	0.00
ENSSSCG00000006865	RTCA	1149.05	-1.63	0.07	-23.79	0.00	0.00
ENSSSCG00000017427	HAP1	1171.10	-1.63	0.07	-21.88	0.00	0.00
ENSSSCG00000008484	SRSF7	1485.08	-1.63	0.07	-24.68	0.00	0.00
ENSSSCG00000002406	AHSA1	2228.04	-1.63	0.07	-23.98	0.00	0.00
ENSSSCG00000035520		2383.76	-1.63	0.27	-6.00	0.00	0.00
ENSSSCG00000013498	EBI3	21.41	-1.63	0.44	-3.73	0.00	0.00
ENSSSCG00000038005		35.52	-1.63	0.35	-4.59	0.00	0.00
ENSSSCG00000005683	TOR1B	507.52	-1.63	0.10	-15.83	0.00	0.00
ENSSSCG00000037617	ZBTB39	962.08	-1.63	0.07	-22.10	0.00	0.00
ENSSSCG00000017522	SP6	272.63	-1.62	0.13	-12.87	0.00	0.00
ENSSSCG00000001734		142.30	-1.62	0.17	-9.31	0.00	0.00
ENSSSCG00000000721	DYRK4	25.43	-1.62	0.39	-4.14	0.00	0.00
ENSSSCG00000018942	RF00591	42.01	-1.62	0.31	-5.28	0.00	0.00
ENSSSCG00000010568	NPM3	1351.54	-1.62	0.08	-19.37	0.00	0.00
ENSSSCG00000016582	FAM71F1	12.01	-1.62	0.57	-2.85	0.00	0.01
ENSSSCG00000010575	PPRC1	1452.17	-1.62	0.07	-22.08	0.00	0.00
ENSSSCG00000038189	SMCR8	2717.57	-1.62	0.06	-28.08	0.00	0.00
ENSSSCG00000004785	SRP14	1665.94	-1.62	0.07	-23.51	0.00	0.00
ENSSSCG00000040684		62.67	-1.62	0.25	-6.45	0.00	0.00
ENSSSCG00000015393	DMTF1	1230.92	-1.62	0.07	-23.96	0.00	0.00
ENSSSCG00000031893		1065.91	-1.61	0.08	-20.50	0.00	0.00
ENSSSCG00000032763	PYCR3	1210.20	-1.61	0.07	-21.97	0.00	0.00

ENSSSCG00000022655	PTGES2	553.73	-1.61	0.09	-17.72	0.00	0.00
ENSSSCG00000016384		5.97	-1.61	0.81	-1.98	0.05	0.06
ENSSSCG00000031262	TXNIP	825.84	-1.61	0.24	-6.81	0.00	0.00
ENSSSCG00000006312	GPA33	7.75	-1.61	0.75	-2.15	0.03	0.04
ENSSSCG00000017287	PSMC5	3267.44	-1.61	0.06	-28.57	0.00	0.00
ENSSSCG00000021307	USP44	260.05	-1.61	0.14	-11.75	0.00	0.00
ENSSSCG00000033693	SLC10A5	67.76	-1.61	0.24	-6.63	0.00	0.00
ENSSSCG00000011767	TTC14	1306.40	-1.61	0.07	-21.84	0.00	0.00
ENSSSCG00000030108	ZNFX1	3420.25	-1.61	0.05	-31.57	0.00	0.00
ENSSSCG00000008878	PPID	1285.81	-1.61	0.07	-24.31	0.00	0.00
ENSSSCG00000001095	GMNN	781.86	-1.61	0.09	-18.49	0.00	0.00
ENSSSCG00000012895		68.41	-1.60	0.25	-6.47	0.00	0.00
ENSSSCG00000017667	PTRH2	522.10	-1.60	0.10	-15.88	0.00	0.00
ENSSSCG00000017385	PSME3	3992.39	-1.60	0.05	-30.99	0.00	0.00
ENSSSCG00000021699		9.08	-1.60	0.67	-2.40	0.02	0.02
ENSSSCG00000036377	OVOL2	23.97	-1.60	0.41	-3.91	0.00	0.00
ENSSSCG00000013853	HSH2D	21.70	-1.60	0.43	-3.74	0.00	0.00
ENSSSCG00000002650	APRT	279.25	-1.60	0.13	-12.08	0.00	0.00
ENSSSCG00000007315	AAR2	1140.23	-1.60	0.07	-21.87	0.00	0.00
ENSSSCG00000021918	CEP19	395.73	-1.60	0.11	-14.37	0.00	0.00
ENSSSCG00000013757	C19orf53	1286.80	-1.59	0.07	-21.64	0.00	0.00
ENSSSCG00000037146	SWSAP1	268.76	-1.59	0.13	-12.37	0.00	0.00
ENSSSCG00000039322	NRGN	223.52	-1.59	0.14	-11.15	0.00	0.00
ENSSSCG00000037958	TOB2	2126.53	-1.59	0.06	-25.31	0.00	0.00
ENSSSCG00000036684	TTPAL	902.16	-1.59	0.08	-20.94	0.00	0.00

ENSSSCG00000017772	SDF2	1368.09	-1.59	0.06	-24.54	0.00	0.00
ENSSSCG00000010085	SDF2L1	73.62	-1.59	0.24	-6.66	0.00	0.00
ENSSSCG00000028593	TEPSIN	240.35	-1.59	0.15	-10.87	0.00	0.00
ENSSSCG00000003566	NUDC	3983.73	-1.59	0.05	-30.85	0.00	0.00
ENSSSCG00000009521	ZIC5	33.64	-1.58	0.36	-4.45	0.00	0.00
ENSSSCG00000033879	ZNF280B	840.18	-1.58	0.08	-20.40	0.00	0.00
ENSSSCG00000033824		7.18	-1.58	0.73	-2.18	0.03	0.04
ENSSSCG00000003990		20.45	-1.58	0.44	-3.58	0.00	0.00
ENSSSCG00000040351		12.89	-1.58	0.56	-2.81	0.00	0.01
ENSSSCG00000016077	HSPD1	17277.18	-1.58	0.06	-27.85	0.00	0.00
ENSSSCG00000035078	CD40	1074.48	-1.58	0.07	-21.07	0.00	0.00
ENSSSCG00000021746	MRPL35	1148.44	-1.58	0.08	-20.09	0.00	0.00
ENSSSCG00000023976	DCP1B	24.85	-1.58	0.39	-4.00	0.00	0.00
ENSSSCG00000008963	AREG	1238.75	-1.58	0.08	-20.62	0.00	0.00
ENSSSCG00000000892	HAL	41.71	-1.58	0.32	-4.94	0.00	0.00
ENSSSCG00000032364	SELENOH	184.81	-1.58	0.15	-10.56	0.00	0.00
ENSSSCG00000012319		74.90	-1.58	0.29	-5.48	0.00	0.00
ENSSSCG00000015234	KIRREL3	14.12	-1.57	0.54	-2.92	0.00	0.01
ENSSSCG00000023166	RNF227	10.96	-1.57	0.60	-2.61	0.01	0.01
ENSSSCG00000013776	DDX39A	1909.88	-1.57	0.07	-23.13	0.00	0.00
ENSSSCG00000031860	MRPS12	572.76	-1.57	0.09	-17.49	0.00	0.00
ENSSSCG00000025454	POLR2C	1781.30	-1.57	0.06	-24.19	0.00	0.00
ENSSSCG00000006183	SBSPON	8.30	-1.57	0.69	-2.26	0.02	0.03
ENSSSCG00000031421	DYNLRB2	45.35	-1.57	0.29	-5.33	0.00	0.00
ENSSSCG00000038130	CANT1	793.06	-1.57	0.08	-19.07	0.00	0.00

ENSSSCG00000035894		9.92	-1.56	0.63	-2.46	0.01	0.02
ENSSSCG00000004048	TCP1	5744.11	-1.56	0.05	-30.49	0.00	0.00
ENSSSCG00000017822	RPA1	3938.19	-1.56	0.05	-28.79	0.00	0.00
ENSSSCG00000026472	RPL7L1	2389.45	-1.56	0.06	-26.06	0.00	0.00
ENSSSCG00000013029	TRMT112	850.27	-1.56	0.09	-17.93	0.00	0.00
ENSSSCG00000007237	PDRG1	1345.27	-1.56	0.07	-22.40	0.00	0.00
ENSSSCG00000034720	IQCK	26.76	-1.56	0.40	-3.94	0.00	0.00
ENSSSCG00000003343	ATAD3A	691.48	-1.56	0.09	-17.30	0.00	0.00
ENSSSCG00000035082	PHF5A	1070.10	-1.56	0.08	-18.82	0.00	0.00
ENSSSCG00000010136	TRMT2A	1186.49	-1.56	0.07	-21.15	0.00	0.00
ENSSSCG00000026890	GALNT6	275.80	-1.56	0.13	-12.22	0.00	0.00
ENSSSCG00000002898		327.85	-1.56	0.12	-13.09	0.00	0.00
ENSSSCG00000008470	THUMPD2	358.30	-1.56	0.11	-13.69	0.00	0.00
ENSSSCG00000019069	RF00592	35.78	-1.55	0.34	-4.55	0.00	0.00
ENSSSCG00000010923	UBE2T	1581.29	-1.55	0.06	-24.17	0.00	0.00
ENSSSCG00000030284	TRNAU1AP	662.75	-1.55	0.09	-17.58	0.00	0.00
ENSSSCG00000022370	TNFSF9	636.11	-1.55	0.09	-17.52	0.00	0.00
ENSSSCG00000004752	EXD1	49.33	-1.55	0.29	-5.26	0.00	0.00
ENSSSCG00000003561	ZDHHC18	2310.76	-1.55	0.06	-25.16	0.00	0.00
ENSSSCG00000024344	CCR5	6.99	-1.55	0.75	-2.08	0.04	0.05
ENSSSCG00000017806	MRM3	672.32	-1.55	0.09	-16.61	0.00	0.00
ENSSSCG00000017208	SLC25A19	365.87	-1.55	0.11	-14.01	0.00	0.00
ENSSSCG00000035134	MRPS7	1400.19	-1.55	0.07	-22.12	0.00	0.00
ENSSSCG00000006471	ISG20L2	1100.47	-1.55	0.08	-18.95	0.00	0.00
ENSSSCG00000002651	CDT1	609.03	-1.55	0.10	-16.26	0.00	0.00

ENSSSCG00000032833	CD320	482.63	-1.55	0.10	-14.74	0.00	0.00
ENSSSCG00000017540	HOXB5	165.64	-1.55	0.16	-9.73	0.00	0.00
ENSSSCG00000003040	ARHGEF1	33.28	-1.55	0.35	-4.45	0.00	0.00
ENSSSCG00000040173	DLGAP3	13.49	-1.55	0.54	-2.85	0.00	0.01
ENSSSCG00000038470		390.22	-1.54	0.11	-14.32	0.00	0.00
ENSSSCG00000017288	FTSJ3	2701.30	-1.54	0.05	-28.31	0.00	0.00
ENSSSCG00000006233	CA8	21.18	-1.54	0.45	-3.42	0.00	0.00
ENSSSCG00000000291	GPR84	14.20	-1.54	0.52	-2.94	0.00	0.01
ENSSSCG00000040158	RF00612	20.95	-1.54	0.43	-3.59	0.00	0.00
ENSSSCG00000003326		16.54	-1.54	0.50	-3.11	0.00	0.00
ENSSSCG00000026290	BBS12	134.27	-1.54	0.17	-8.88	0.00	0.00
ENSSSCG00000015660	PFKFB2	827.72	-1.54	0.08	-19.19	0.00	0.00
ENSSSCG00000025965	SPDL1	1168.81	-1.54	0.07	-21.86	0.00	0.00
ENSSSCG00000029538	PPP2R1B	4389.51	-1.54	0.06	-26.37	0.00	0.00
ENSSSCG00000031219	NAPB	603.23	-1.53	0.09	-17.25	0.00	0.00
ENSSSCG00000003288	HSPBP1	889.65	-1.53	0.09	-17.43	0.00	0.00
ENSSSCG00000006079	RIDA	1810.92	-1.53	0.06	-24.24	0.00	0.00
ENSSSCG00000024019	GTF2H5	242.37	-1.53	0.13	-11.69	0.00	0.00
ENSSSCG00000040510	KIF9	172.89	-1.53	0.16	-9.65	0.00	0.00
ENSSSCG00000017227	FDXR	847.35	-1.53	0.08	-18.70	0.00	0.00
ENSSSCG00000036746	RASL10B	1003.67	-1.53	0.08	-19.05	0.00	0.00
ENSSSCG00000036272		9.40	-1.53	0.65	-2.35	0.02	0.03
ENSSSCG00000016313	HJURP	565.36	-1.53	0.09	-16.98	0.00	0.00
ENSSSCG00000039635		740.52	-1.53	0.08	-18.60	0.00	0.00
ENSSSCG00000039678	TMEM269	33.20	-1.53	0.34	-4.45	0.00	0.00

ENSSSCG00000017421	ACLY	6156.38	-1.53	0.05	-30.90	0.00	0.00
ENSSSCG00000037644	ssc-mir-6782	27.34	-1.53	0.37	-4.09	0.00	0.00
ENSSSCG00000029037	DRD1	66.18	-1.53	0.24	-6.25	0.00	0.00
ENSSSCG00000006665		3405.51	-1.53	0.06	-25.20	0.00	0.00
ENSSSCG00000039370		867.49	-1.53	0.08	-19.02	0.00	0.00
ENSSSCG00000010012	SLC35E4	168.97	-1.53	0.16	-9.38	0.00	0.00
ENSSSCG00000037000	SIGMAR1	1607.20	-1.53	0.07	-22.65	0.00	0.00
ENSSSCG00000009378	CKAP2	3464.79	-1.53	0.06	-25.96	0.00	0.00
ENSSSCG00000000203	KCNH3	660.98	-1.53	0.10	-16.06	0.00	0.00
ENSSSCG00000033689	GPS1	2209.03	-1.53	0.06	-26.49	0.00	0.00
ENSSSCG00000005102	AEN	841.20	-1.53	0.08	-20.15	0.00	0.00
ENSSSCG00000030271	GSTO2	14.70	-1.53	0.51	-2.98	0.00	0.00
ENSSSCG00000039351	PIGH	666.94	-1.53	0.10	-15.62	0.00	0.00
ENSSSCG00000039935	CRIPT	433.40	-1.53	0.11	-13.72	0.00	0.00
ENSSSCG00000024166	SLC2A6	200.05	-1.53	0.16	-9.49	0.00	0.00
ENSSSCG00000035189		627.73	-1.52	0.09	-17.31	0.00	0.00
ENSSSCG00000005596	ARPC5L	1089.75	-1.52	0.07	-20.51	0.00	0.00
ENSSSCG00000039962	HSPB1	12373.86	-1.52	0.05	-28.60	0.00	0.00
ENSSSCG00000028978	CAD	2600.04	-1.52	0.06	-26.33	0.00	0.00
ENSSSCG00000007095	POLR3F	788.57	-1.52	0.08	-18.31	0.00	0.00
ENSSSCG00000031708		1225.34	-1.52	0.09	-16.81	0.00	0.00
ENSSSCG00000013662	PPAN	427.45	-1.52	0.11	-14.17	0.00	0.00
ENSSSCG00000038916	LSM10	74.47	-1.52	0.24	-6.41	0.00	0.00
ENSSSCG00000011000	DNAJA1	3006.34	-1.52	0.06	-25.52	0.00	0.00
ENSSSCG00000014799		93.75	-1.52	0.22	-6.94	0.00	0.00

ENSSSCG00000027486	TRIP6	19.81	-1.52	0.46	-3.33	0.00	0.00
ENSSSCG00000006610	S100A11	9976.05	-1.52	0.05	-31.44	0.00	0.00
ENSSSCG00000028696	FEN1	1069.00	-1.52	0.07	-21.48	0.00	0.00
ENSSSCG00000010445	ANKRD22	238.53	-1.52	0.13	-11.51	0.00	0.00
ENSSSCG00000004077	FBXO5	442.55	-1.52	0.11	-13.99	0.00	0.00
ENSSSCG00000025160	DPF1	25.61	-1.52	0.41	-3.67	0.00	0.00
ENSSSCG00000015201	MSANTD2	370.41	-1.52	0.11	-13.63	0.00	0.00
ENSSSCG00000008589	WDCP	308.03	-1.51	0.12	-12.87	0.00	0.00
ENSSSCG00000006612	S100A10	10689.09	-1.51	0.06	-26.04	0.00	0.00
ENSSSCG00000015073	TAGLN	329.50	-1.51	0.12	-12.36	0.00	0.00
ENSSSCG00000017273	PSMD12	3259.95	-1.51	0.06	-27.09	0.00	0.00
ENSSSCG00000015868	ARL6IP6	1745.29	-1.51	0.07	-23.04	0.00	0.00
ENSSSCG00000017275	NOL11	1312.07	-1.51	0.07	-21.49	0.00	0.00
ENSSSCG00000007039	PRNP	4479.28	-1.51	0.06	-26.64	0.00	0.00
ENSSSCG00000025900	SH3BP5L	558.99	-1.51	0.09	-16.41	0.00	0.00
ENSSSCG00000011912	QTRT2	694.49	-1.51	0.09	-17.16	0.00	0.00
ENSSSCG00000009178	H2AFZ	4536.65	-1.51	0.05	-29.13	0.00	0.00
ENSSSCG00000004670	C15orf48	46.85	-1.51	0.29	-5.19	0.00	0.00
ENSSSCG00000025858	ELN	9.30	-1.50	0.65	-2.32	0.02	0.03
ENSSSCG00000011910		9.96	-1.50	0.62	-2.44	0.01	0.02
ENSSSCG00000021222		14.95	-1.50	0.51	-2.95	0.00	0.00
ENSSSCG00000007421		36.71	-1.50	0.33	-4.56	0.00	0.00
ENSSSCG00000014375	HARS	900.63	-1.50	0.08	-19.38	0.00	0.00
ENSSSCG00000006477	BCAN	55.09	-1.50	0.27	-5.58	0.00	0.00
ENSSSCG00000038950	VARS	2582.87	-1.50	0.06	-25.15	0.00	0.00

ENSSSCG00000009921	OASL	23.24	-1.50	0.41	-3.66	0.00	0.00
ENSSSCG00000009531	TEX30	577.68	-1.50	0.09	-15.94	0.00	0.00
ENSSSCG00000032960	MANEAL	236.78	-1.50	0.13	-11.16	0.00	0.00
ENSSSCG00000031380	MARCKSL1	1681.67	-1.50	0.07	-21.32	0.00	0.00
ENSSSCG00000026919	RF01294	13.91	-1.50	0.53	-2.82	0.00	0.01
ENSSSCG00000038651	FNDC11	7.04	-1.50	0.76	-1.98	0.05	0.07
ENSSSCG00000007800	SEPT1	10.64	-1.50	0.61	-2.44	0.01	0.02
ENSSSCG00000026752	BYSL	1177.65	-1.50	0.07	-20.22	0.00	0.00
ENSSSCG00000024529	TRMT5	676.78	-1.50	0.09	-16.24	0.00	0.00
ENSSSCG00000005008	POLE2	207.73	-1.50	0.14	-10.41	0.00	0.00
ENSSSCG00000014965	ENDOD1	677.67	-1.50	0.09	-16.99	0.00	0.00
ENSSSCG00000008729	LYAR	927.57	-1.50	0.08	-18.92	0.00	0.00
ENSSSCG00000036639	STOM	4745.95	-1.49	0.05	-32.07	0.00	0.00
ENSSSCG00000027530	TAF12	619.88	-1.49	0.09	-16.98	0.00	0.00
ENSSSCG00000032908	PSMD7	1856.20	-1.49	0.06	-24.62	0.00	0.00
ENSSSCG00000034278	SLC31A1	2218.73	-1.49	0.06	-26.08	0.00	0.00
ENSSSCG00000010576	NOLC1	3548.50	-1.49	0.05	-29.13	0.00	0.00
ENSSSCG00000032404	KLHL11	847.42	-1.49	0.08	-19.12	0.00	0.00
ENSSSCG00000017200	UNC13D	120.54	-1.49	0.20	-7.40	0.00	0.00
ENSSSCG00000013011	SAC3D1	76.15	-1.49	0.23	-6.48	0.00	0.00
ENSSSCG00000040497	RF00026	9.10	-1.49	0.68	-2.18	0.03	0.04
ENSSSCG00000017466	CCR7	325.80	-1.49	0.13	-11.51	0.00	0.00
ENSSSCG00000035253	MAFG	596.99	-1.49	0.09	-16.30	0.00	0.00
ENSSSCG00000027514	ELP5	1280.39	-1.48	0.08	-18.17	0.00	0.00
ENSSSCG00000038411	ssc-mir-6782	33.79	-1.48	0.34	-4.32	0.00	0.00

ENSSSCG00000004942	TIPIN	670.91	-1.48	0.09	-16.15	0.00	0.00
ENSSSCG00000029571	AVIL	50.59	-1.48	0.28	-5.28	0.00	0.00
ENSSSCG00000016758		431.71	-1.48	0.10	-14.72	0.00	0.00
ENSSSCG00000004130		975.60	-1.48	0.08	-18.59	0.00	0.00
ENSSSCG00000007528	PHACTR3	14.11	-1.48	0.52	-2.84	0.00	0.01
ENSSSCG00000000521	PHLDA1	722.57	-1.48	0.08	-17.63	0.00	0.00
ENSSSCG00000039472	SLC30A1	1522.41	-1.48	0.08	-18.04	0.00	0.00
ENSSSCG00000007963	ZNF263	809.44	-1.48	0.08	-19.16	0.00	0.00
ENSSSCG00000027372	SAMD9	4725.32	-1.48	0.06	-26.03	0.00	0.00
ENSSSCG00000009622	POLR3D	1363.20	-1.48	0.07	-22.35	0.00	0.00
ENSSSCG00000008545	ZNF512	261.62	-1.47	0.13	-10.96	0.00	0.00
ENSSSCG00000037170	POMP	2608.77	-1.47	0.06	-24.10	0.00	0.00
ENSSSCG00000040422		196.12	-1.47	0.15	-9.74	0.00	0.00
ENSSSCG00000006105	GEM	1445.23	-1.47	0.07	-20.60	0.00	0.00
ENSSSCG00000026761	CCT8	6655.12	-1.47	0.05	-29.97	0.00	0.00
ENSSSCG00000024970	ETV3	2664.08	-1.47	0.05	-26.75	0.00	0.00
ENSSSCG00000022258		12.15	-1.47	0.58	-2.52	0.01	0.02
ENSSSCG00000004223	HEY2	54.63	-1.47	0.27	-5.47	0.00	0.00
ENSSSCG00000013115	CD5	28.82	-1.47	0.37	-3.97	0.00	0.00
ENSSSCG00000009542	TNFSF13B	14.19	-1.47	0.51	-2.86	0.00	0.01
ENSSSCG00000027806	SAMHD1	3270.34	-1.47	0.06	-24.05	0.00	0.00
ENSSSCG00000033286		14.16	-1.47	0.52	-2.84	0.00	0.01
ENSSSCG00000036178	CCDC167	671.60	-1.47	0.09	-16.99	0.00	0.00
ENSSSCG00000039652	OTUD3	283.24	-1.46	0.12	-11.78	0.00	0.00
ENSSSCG00000015712	DDX18	3094.15	-1.46	0.06	-23.78	0.00	0.00

ENSSSCG00000033998	SLC43A3	1702.88	-1.46	0.07	-20.20	0.00	0.00
ENSSSCG00000013475	NCLN	1276.45	-1.46	0.07	-20.81	0.00	0.00
ENSSSCG00000021180	POLR2K	559.69	-1.46	0.09	-16.25	0.00	0.00
ENSSSCG00000017814		110.48	-1.46	0.19	-7.74	0.00	0.00
ENSSSCG00000017394	TUBG1	1197.75	-1.46	0.09	-15.82	0.00	0.00
ENSSSCG00000024550	UQCR10	721.23	-1.46	0.09	-15.68	0.00	0.00
ENSSSCG00000036015	TSSC4	277.05	-1.46	0.13	-11.53	0.00	0.00
ENSSSCG00000006520	SCAMP3	2390.29	-1.46	0.06	-24.05	0.00	0.00
ENSSSCG00000013778	NDUFB7	757.28	-1.46	0.08	-17.39	0.00	0.00
ENSSSCG00000012943	MRPL11	1000.88	-1.46	0.09	-16.39	0.00	0.00
ENSSSCG00000016410	MNX1	93.50	-1.45	0.21	-7.04	0.00	0.00
ENSSSCG00000034814	MRTO4	1026.64	-1.45	0.07	-19.48	0.00	0.00
ENSSSCG00000017429	P3H4	2050.67	-1.45	0.06	-22.83	0.00	0.00
ENSSSCG00000017373	CFAP97D1	12.46	-1.45	0.58	-2.52	0.01	0.02
ENSSSCG00000028290	RF00086	17.25	-1.45	0.47	-3.07	0.00	0.00
ENSSSCG00000002459	GON7	1104.15	-1.45	0.08	-18.61	0.00	0.00
ENSSSCG00000012317	GSPT2	336.75	-1.45	0.12	-11.97	0.00	0.00
ENSSSCG00000014958	FUT4	74.55	-1.45	0.23	-6.35	0.00	0.00
ENSSSCG00000015282	PPP1R15B	2156.61	-1.45	0.06	-25.39	0.00	0.00
ENSSSCG00000031251	DDA1	777.02	-1.45	0.08	-17.85	0.00	0.00
ENSSSCG00000038615	TAF9	1053.80	-1.45	0.07	-19.66	0.00	0.00
ENSSSCG00000032060		259.45	-1.45	0.14	-10.59	0.00	0.00
ENSSSCG00000033168	TMEM161A	834.78	-1.45	0.08	-18.09	0.00	0.00
ENSSSCG00000025736	RECQL4	581.60	-1.45	0.09	-15.79	0.00	0.00
ENSSSCG00000039135	ARHGAP33	70.03	-1.45	0.24	-6.15	0.00	0.00

ENSSSCG00000036554		9.85	-1.45	0.67	-2.17	0.03	0.04
ENSSSCG00000040359	GOLGA7B	157.21	-1.45	0.16	-9.08	0.00	0.00
ENSSSCG00000016659	KIAA0895	403.85	-1.45	0.11	-13.25	0.00	0.00
ENSSSCG00000017555		2713.58	-1.45	0.06	-25.96	0.00	0.00
ENSSSCG00000021917	HIPK4	9.55	-1.45	0.66	-2.19	0.03	0.04
ENSSSCG00000006842		467.80	-1.45	0.11	-13.48	0.00	0.00
ENSSSCG00000011061	PDSS1	264.34	-1.44	0.13	-11.41	0.00	0.00
ENSSSCG00000036183	MCIDAS	12.85	-1.44	0.55	-2.61	0.01	0.01
ENSSSCG00000040396		530.96	-1.44	0.10	-14.93	0.00	0.00
ENSSSCG00000040830		11.06	-1.44	0.59	-2.45	0.01	0.02
ENSSSCG00000013176		1268.90	-1.44	0.07	-20.27	0.00	0.00
ENSSSCG00000003089	TOMM40	1526.87	-1.44	0.07	-21.70	0.00	0.00
ENSSSCG00000018524	RF00016	9.67	-1.44	0.62	-2.30	0.02	0.03
ENSSSCG00000040282		10.36	-1.44	0.60	-2.38	0.02	0.03
ENSSSCG00000008959	CXCL2	2339.68	-1.44	0.13	-10.87	0.00	0.00
ENSSSCG00000014901	PCF11	2140.14	-1.44	0.06	-23.35	0.00	0.00
ENSSSCG00000006504	KHDC4	2135.88	-1.43	0.06	-22.71	0.00	0.00
ENSSSCG00000031730		838.93	-1.43	0.08	-18.85	0.00	0.00
ENSSSCG00000002004		1487.32	-1.43	0.07	-21.90	0.00	0.00
ENSSSCG00000025440	ELOA	3019.45	-1.43	0.06	-25.82	0.00	0.00
ENSSSCG00000006394	CFAP45	379.15	-1.43	0.11	-12.90	0.00	0.00
ENSSSCG00000003248		12.19	-1.43	0.56	-2.57	0.01	0.02
ENSSSCG00000027119	SELRC1	285.57	-1.43	0.12	-11.64	0.00	0.00
ENSSSCG00000030295		4019.45	-1.43	0.06	-23.86	0.00	0.00
ENSSSCG00000031869	VPS72	1529.80	-1.43	0.07	-21.43	0.00	0.00

ENSSSCG00000006523	MTX1	805.88	-1.43	0.09	-16.72	0.00	0.00
ENSSSCG00000010822	BPNT1	608.85	-1.43	0.09	-16.20	0.00	0.00
ENSSSCG00000035193	MRPS23	838.41	-1.43	0.08	-17.48	0.00	0.00
ENSSSCG00000036867	C11orf71	8.53	-1.43	0.67	-2.12	0.03	0.05
ENSSSCG00000004263	ZUP1	555.14	-1.43	0.09	-15.27	0.00	0.00
ENSSSCG00000003766	DNAJB4	569.39	-1.43	0.09	-15.72	0.00	0.00
ENSSSCG00000031580		453.55	-1.43	0.11	-13.47	0.00	0.00
ENSSSCG00000040906		18.84	-1.43	0.46	-3.12	0.00	0.00
ENSSSCG00000038146	RF00277	83.90	-1.43	0.22	-6.38	0.00	0.00
ENSSSCG00000033694	MRPL41	268.48	-1.42	0.12	-11.47	0.00	0.00
ENSSSCG00000002449		370.18	-1.42	0.11	-12.86	0.00	0.00
ENSSSCG00000024071	SCARF1	44.40	-1.42	0.30	-4.76	0.00	0.00
ENSSSCG00000025182	ELK4	1702.13	-1.42	0.06	-22.09	0.00	0.00
ENSSSCG00000013177	MED19	471.05	-1.42	0.10	-14.72	0.00	0.00
ENSSSCG00000010518	EXOSC1	1378.20	-1.42	0.06	-22.56	0.00	0.00
ENSSSCG00000008899		113.90	-1.42	0.18	-7.69	0.00	0.00
ENSSSCG00000015384	TOMM7	554.22	-1.42	0.10	-14.75	0.00	0.00
ENSSSCG00000038713	CRIP2	2631.01	-1.42	0.07	-20.23	0.00	0.00
ENSSSCG00000039781		22.85	-1.42	0.41	-3.49	0.00	0.00
ENSSSCG00000017571	EME1	767.80	-1.42	0.09	-16.30	0.00	0.00
ENSSSCG00000032309		19.12	-1.42	0.45	-3.18	0.00	0.00
ENSSSCG00000025116	TONSL	1208.03	-1.42	0.08	-18.45	0.00	0.00
ENSSSCG00000026612	TIMM50	1574.34	-1.42	0.06	-23.32	0.00	0.00
ENSSSCG00000027905		912.50	-1.42	0.08	-17.91	0.00	0.00
ENSSSCG00000026257	STMN1	3568.05	-1.42	0.06	-25.01	0.00	0.00

ENSSSCG00000034472	RF00614	13.03	-1.42	0.56	-2.55	0.01	0.02
ENSSSCG00000017971	NAA38	389.69	-1.41	0.11	-12.48	0.00	0.00
ENSSSCG00000009769	GTF2H3	1376.77	-1.41	0.07	-19.82	0.00	0.00
ENSSSCG00000036626		7.16	-1.41	0.72	-1.97	0.05	0.07
ENSSSCG00000001219	TRIM27	586.60	-1.41	0.09	-16.21	0.00	0.00
ENSSSCG00000017207	NUP85	1429.74	-1.41	0.07	-21.22	0.00	0.00
ENSSSCG00000031941	MED22	300.89	-1.41	0.13	-11.29	0.00	0.00
ENSSSCG00000011798		1141.19	-1.41	0.07	-20.04	0.00	0.00
ENSSSCG00000008268	AUP1	3352.56	-1.41	0.05	-26.90	0.00	0.00
ENSSSCG00000023379	UBE2L6	369.84	-1.41	0.11	-12.56	0.00	0.00
ENSSSCG00000003820	USP1	4301.77	-1.41	0.06	-25.11	0.00	0.00
ENSSSCG00000010719		267.17	-1.41	0.12	-11.47	0.00	0.00
ENSSSCG00000015428	PUS7	3821.73	-1.41	0.05	-27.76	0.00	0.00
ENSSSCG00000033212		607.41	-1.41	0.10	-14.49	0.00	0.00
ENSSSCG00000003423	DRAXIN	41.49	-1.41	0.30	-4.63	0.00	0.00
ENSSSCG00000039618		388.43	-1.41	0.10	-13.57	0.00	0.00
ENSSSCG00000006499	LAMTOR2	852.71	-1.41	0.08	-17.83	0.00	0.00
ENSSSCG00000003080		62.37	-1.41	0.27	-5.30	0.00	0.00
ENSSSCG00000016750	YKT6	2291.05	-1.41	0.06	-23.85	0.00	0.00
ENSSSCG00000021092	FAM53C	2334.18	-1.41	0.06	-23.60	0.00	0.00
ENSSSCG00000002378	MLH3	417.59	-1.41	0.11	-13.30	0.00	0.00
ENSSSCG00000002385	TGFB3	49.70	-1.41	0.28	-5.08	0.00	0.00
ENSSSCG00000032655	ALYREF	1352.14	-1.41	0.07	-20.58	0.00	0.00
ENSSSCG00000023229	ETV5	395.69	-1.41	0.11	-13.39	0.00	0.00
ENSSSCG00000008557	EMILIN1	70.40	-1.41	0.25	-5.64	0.00	0.00

ENSSSCG00000040425	YRDC	319.29	-1.41	0.11	-12.31	0.00	0.00
ENSSSCG00000003037	ZNF574	744.59	-1.40	0.09	-15.08	0.00	0.00
ENSSSCG00000029097	RCC1	1517.58	-1.40	0.07	-21.27	0.00	0.00
ENSSSCG00000023158		1096.68	-1.40	0.08	-18.29	0.00	0.00
ENSSSCG00000035663		295.95	-1.40	0.12	-11.39	0.00	0.00
ENSSSCG00000017591	NME2	4073.62	-1.40	0.06	-24.76	0.00	0.00
ENSSSCG00000035495	KITLG	189.12	-1.40	0.15	-9.18	0.00	0.00
ENSSSCG00000034843		511.99	-1.40	0.10	-14.67	0.00	0.00
ENSSSCG00000027555	DEFB1	11.38	-1.40	0.59	-2.38	0.02	0.02
ENSSSCG00000021359	CDC42EP3	547.87	-1.40	0.10	-13.84	0.00	0.00
ENSSSCG00000024261	CBX2	499.31	-1.40	0.10	-14.11	0.00	0.00
ENSSSCG00000014274	PDLIM4	9.98	-1.40	0.63	-2.20	0.03	0.04
ENSSSCG00000010058	SNRPD3	2163.03	-1.40	0.06	-22.30	0.00	0.00
ENSSSCG00000016652	LRRN3	39.19	-1.39	0.31	-4.52	0.00	0.00
ENSSSCG00000004691	EIF3J	1803.31	-1.39	0.06	-22.09	0.00	0.00
ENSSSCG00000032144	ZNF140	82.21	-1.39	0.23	-6.05	0.00	0.00
ENSSSCG00000023984	RSL1D1	4156.47	-1.39	0.06	-24.41	0.00	0.00
ENSSSCG00000003701	SNRPD1	883.88	-1.39	0.09	-16.01	0.00	0.00
ENSSSCG00000029095	RF00085	13.75	-1.39	0.53	-2.63	0.01	0.01
ENSSSCG00000025460	SPSB2	50.79	-1.39	0.28	-5.01	0.00	0.00
ENSSSCG00000006472	CRABP2	336.25	-1.39	0.12	-12.06	0.00	0.00
ENSSSCG00000027538	TMEM258	696.70	-1.39	0.09	-15.10	0.00	0.00
ENSSSCG00000037999	PPP1R14B	777.51	-1.39	0.09	-14.65	0.00	0.00
ENSSSCG00000024773	EID2	97.48	-1.39	0.20	-6.85	0.00	0.00
ENSSSCG00000022728	HOXC10	15.32	-1.39	0.51	-2.74	0.01	0.01

ENSSSCG00000013656	MRPL4	1268.49	-1.39	0.08	-17.00	0.00	0.00
ENSSSCG00000010121	UFD1	1471.79	-1.39	0.06	-21.68	0.00	0.00
ENSSSCG00000038440	GTPBP3	370.67	-1.39	0.11	-12.08	0.00	0.00
ENSSSCG00000017489	PSMD3	3612.30	-1.39	0.05	-28.46	0.00	0.00
ENSSSCG00000038425	RF01892	140.53	-1.39	0.17	-8.20	0.00	0.00
ENSSSCG00000040025	NANOS1	35.54	-1.39	0.33	-4.17	0.00	0.00
ENSSSCG00000016753	POLD2	619.11	-1.39	0.08	-16.34	0.00	0.00
ENSSSCG00000025041		215.85	-1.39	0.14	-9.66	0.00	0.00
ENSSSCG00000021815		100.57	-1.39	0.20	-6.78	0.00	0.00
ENSSSCG00000013313	PRRG4	1149.20	-1.39	0.08	-17.87	0.00	0.00
ENSSSCG00000016886		106.86	-1.39	0.19	-7.26	0.00	0.00
ENSSSCG00000018061		4127.97	-1.38	0.14	-9.97	0.00	0.00
ENSSSCG00000035419	RARRES2	28.23	-1.38	0.38	-3.67	0.00	0.00
ENSSSCG00000024676	SRPK3	17.06	-1.38	0.48	-2.86	0.00	0.01
ENSSSCG00000027124		15.79	-1.38	0.49	-2.82	0.00	0.01
ENSSSCG00000003918	TOE1	316.67	-1.38	0.12	-11.17	0.00	0.00
ENSSSCG00000036360	LURAP1L	13.05	-1.38	0.55	-2.50	0.01	0.02
ENSSSCG00000008579	CENPO	924.37	-1.38	0.07	-18.75	0.00	0.00
ENSSSCG00000026169	MFSD12	2788.20	-1.38	0.06	-24.54	0.00	0.00
ENSSSCG00000030510	FBL	1357.26	-1.38	0.08	-17.17	0.00	0.00
ENSSSCG00000037325	UBIAD1	531.02	-1.38	0.10	-13.52	0.00	0.00
ENSSSCG00000004480	COX7A2	1068.60	-1.38	0.07	-18.52	0.00	0.00
ENSSSCG00000013524	MICOS13	470.28	-1.38	0.10	-13.45	0.00	0.00
ENSSSCG00000036675		216.23	-1.38	0.14	-10.11	0.00	0.00
ENSSSCG00000014972	CCDC82	1063.34	-1.38	0.09	-15.71	0.00	0.00

ENSSSCG00000023783	MRPL27	207.38	-1.38	0.15	-9.06	0.00	0.00
ENSSSCG00000038631	TCFL5	643.08	-1.38	0.09	-15.06	0.00	0.00
ENSSSCG00000016755	POLM	287.71	-1.38	0.12	-11.53	0.00	0.00
ENSSSCG00000016578	FLNC	9.99	-1.38	0.61	-2.27	0.02	0.03
ENSSSCG00000029285		224.68	-1.38	0.14	-10.13	0.00	0.00
ENSSSCG00000039713	ANKRD9	146.32	-1.37	0.19	-7.34	0.00	0.00
ENSSSCG00000000373	CDK2	1213.74	-1.37	0.07	-18.35	0.00	0.00
ENSSSCG00000037749	RF00580	18.14	-1.37	0.46	-3.00	0.00	0.00
ENSSSCG00000010795		2304.12	-1.37	0.06	-23.72	0.00	0.00
ENSSSCG00000011299	CLEC3B	8.89	-1.37	0.66	-2.08	0.04	0.05
ENSSSCG00000011359	ATRIP	574.23	-1.37	0.09	-15.10	0.00	0.00
ENSSSCG00000007236	TTLL9	124.45	-1.37	0.18	-7.67	0.00	0.00
ENSSSCG00000038955	CAMLG	599.64	-1.37	0.09	-15.68	0.00	0.00
ENSSSCG00000028210	NT5C	712.16	-1.37	0.08	-16.44	0.00	0.00
ENSSSCG00000023865		220.16	-1.37	0.13	-10.16	0.00	0.00
ENSSSCG00000006309	CD247	27.61	-1.37	0.37	-3.66	0.00	0.00
ENSSSCG00000025334	POU4F1	68.99	-1.37	0.24	-5.62	0.00	0.00
ENSSSCG00000025206	RNF19B	1003.27	-1.37	0.07	-18.92	0.00	0.00
ENSSSCG00000014982	MMP7	72.51	-1.37	0.24	-5.74	0.00	0.00
ENSSSCG00000007064	MKKS	523.51	-1.37	0.09	-14.56	0.00	0.00
ENSSSCG00000026706	PIGS	2923.87	-1.37	0.05	-25.03	0.00	0.00
ENSSSCG00000011740	SERPINI1	178.85	-1.37	0.15	-9.06	0.00	0.00
ENSSSCG00000002803	CCDC113	61.36	-1.37	0.25	-5.40	0.00	0.00
ENSSSCG00000033167	SLC7A6OS	1233.30	-1.36	0.07	-19.87	0.00	0.00
ENSSSCG00000017954	SOX15	19.01	-1.36	0.47	-2.91	0.00	0.01

ENSSSCG00000005440		1023.27	-1.36	0.07	-18.25	0.00	0.00
ENSSSCG00000032320	TCIM	1506.77	-1.36	0.08	-18.13	0.00	0.00
ENSSSCG00000013591	TIMM44	487.31	-1.36	0.10	-13.50	0.00	0.00
ENSSSCG00000031078	SP8	55.13	-1.36	0.27	-5.06	0.00	0.00
ENSSSCG00000001504	WDR46	464.24	-1.36	0.10	-13.54	0.00	0.00
ENSSSCG00000025513	RF00099	39.06	-1.36	0.31	-4.34	0.00	0.00
ENSSSCG00000030677	GART	2708.55	-1.36	0.06	-24.39	0.00	0.00
ENSSSCG00000034069		10.04	-1.36	0.63	-2.16	0.03	0.04
ENSSSCG00000039761	MYCL	216.83	-1.36	0.14	-9.47	0.00	0.00
ENSSSCG00000013647	CDC37	3973.90	-1.36	0.05	-28.78	0.00	0.00
ENSSSCG00000037310	ATP6AP1	4645.99	-1.36	0.05	-27.66	0.00	0.00
ENSSSCG00000003000	ITPKC	403.83	-1.36	0.11	-12.21	0.00	0.00
ENSSSCG00000020020	RF00266	8.84	-1.36	0.66	-2.07	0.04	0.05
ENSSSCG00000003421		636.72	-1.36	0.09	-15.77	0.00	0.00
ENSSSCG00000013001	MRPL49	1374.08	-1.36	0.07	-19.81	0.00	0.00
ENSSSCG00000039538		1171.16	-1.36	0.07	-19.41	0.00	0.00
ENSSSCG00000035209		4018.60	-1.36	0.06	-22.61	0.00	0.00
ENSSSCG00000031413	PMPCA	2686.13	-1.36	0.06	-24.47	0.00	0.00
ENSSSCG00000005923	SPATC1	24.51	-1.36	0.41	-3.35	0.00	0.00
ENSSSCG00000040450		12.42	-1.36	0.56	-2.44	0.01	0.02
ENSSSCG00000003711	CABYR	58.38	-1.36	0.26	-5.31	0.00	0.00
ENSSSCG00000034802		25.71	-1.36	0.40	-3.42	0.00	0.00
ENSSSCG00000007623	BUD31	1177.09	-1.36	0.07	-18.80	0.00	0.00
ENSSSCG00000004047	MRPL18	777.11	-1.36	0.08	-16.18	0.00	0.00
ENSSSCG00000036700	RPUSD2	174.96	-1.35	0.15	-9.02	0.00	0.00

ENSSSCG00000036658		2631.04	-1.35	0.06	-23.52	0.00	0.00
ENSSSCG00000005713	PLPP7	47.90	-1.35	0.29	-4.73	0.00	0.00
ENSSSCG00000015311	CYP51A1	2930.32	-1.35	0.06	-22.18	0.00	0.00
ENSSSCG00000040778	NIPA1	1033.38	-1.35	0.08	-17.04	0.00	0.00
ENSSSCG00000000579	GOLT1B	885.50	-1.35	0.08	-17.61	0.00	0.00
ENSSSCG00000027361	OVOL1	316.40	-1.35	0.11	-11.80	0.00	0.00
ENSSSCG00000003015	EXOSC5	469.33	-1.35	0.10	-13.10	0.00	0.00
ENSSSCG00000000142	FOXRED2	110.45	-1.35	0.19	-7.25	0.00	0.00
ENSSSCG00000036935	RF01882	36.38	-1.35	0.33	-4.15	0.00	0.00
ENSSSCG00000007749		742.56	-1.35	0.09	-15.67	0.00	0.00
ENSSSCG00000019644	RF00281	53.01	-1.35	0.28	-4.89	0.00	0.00
ENSSSCG00000012893	UNC93B1	1595.10	-1.35	0.07	-19.20	0.00	0.00
ENSSSCG00000002814	ADGRG3	26.06	-1.35	0.39	-3.46	0.00	0.00
ENSSSCG00000039573	SLPI	432.75	-1.35	0.10	-13.09	0.00	0.00
ENSSSCG00000036871	EXOC3L4	157.91	-1.35	0.16	-8.17	0.00	0.00
ENSSSCG00000003861	ORC1	1988.82	-1.35	0.06	-21.58	0.00	0.00
ENSSSCG00000007097	SEC23B	4551.81	-1.34	0.05	-28.62	0.00	0.00
ENSSSCG00000015232	ST3GAL4	435.59	-1.34	0.10	-13.32	0.00	0.00
ENSSSCG00000009774	C12orf65	323.13	-1.34	0.12	-11.41	0.00	0.00
ENSSSCG00000035057	RUNDC1	871.31	-1.34	0.08	-15.84	0.00	0.00
ENSSSCG00000012277	TIMP1	3026.09	-1.34	0.06	-23.80	0.00	0.00
ENSSSCG00000033421		381.27	-1.34	0.11	-12.71	0.00	0.00
ENSSSCG00000014176	RGMB	84.57	-1.34	0.21	-6.25	0.00	0.00
ENSSSCG00000035048		4950.72	-1.34	0.05	-25.49	0.00	0.00
ENSSSCG00000000063	POLR3H	221.35	-1.34	0.14	-9.86	0.00	0.00

ENSSSCG00000014988	MMP13	562.99	-1.34	0.10	-13.48	0.00	0.00
ENSSSCG00000017218	CDR2L	1558.14	-1.34	0.08	-16.86	0.00	0.00
ENSSSCG00000009806	SETD1B	627.29	-1.34	0.09	-14.56	0.00	0.00
ENSSSCG00000011355		38.86	-1.34	0.31	-4.27	0.00	0.00
ENSSSCG00000016232	MRPL44	625.60	-1.34	0.09	-15.42	0.00	0.00
ENSSSCG00000013837	AKAP8	856.04	-1.34	0.08	-17.02	0.00	0.00
ENSSSCG00000032166		2655.15	-1.34	0.06	-21.15	0.00	0.00
ENSSSCG00000024299	SCN3B	186.87	-1.34	0.15	-9.12	0.00	0.00
ENSSSCG00000025187	TMEM126A	511.75	-1.34	0.09	-14.13	0.00	0.00
ENSSSCG00000000439	KIF5A	48.45	-1.34	0.30	-4.54	0.00	0.00
ENSSSCG00000001512	CUTA	683.21	-1.34	0.09	-14.83	0.00	0.00
ENSSSCG00000006487	CCT3	11474.85	-1.34	0.06	-22.05	0.00	0.00
ENSSSCG00000028433	DUS4L	74.44	-1.34	0.24	-5.61	0.00	0.00
ENSSSCG00000025791	MON1A	120.45	-1.34	0.19	-6.87	0.00	0.00
ENSSSCG00000036614	PGP	1217.78	-1.34	0.08	-17.22	0.00	0.00
ENSSSCG00000014961	CWC15	1526.44	-1.34	0.07	-20.19	0.00	0.00
ENSSSCG00000032752	ZBTB43	548.91	-1.34	0.10	-13.66	0.00	0.00
ENSSSCG00000032081	NDUFS3	1036.34	-1.34	0.08	-15.99	0.00	0.00
ENSSSCG00000022864	EIF6	4147.63	-1.33	0.05	-28.23	0.00	0.00
ENSSSCG00000026407	NCCRP1	666.93	-1.33	0.09	-15.09	0.00	0.00
ENSSSCG00000025597		20.26	-1.33	0.43	-3.07	0.00	0.00
ENSSSCG00000009820	GPN3	1001.88	-1.33	0.08	-16.93	0.00	0.00
ENSSSCG00000033090		62.11	-1.33	0.27	-4.85	0.00	0.00
ENSSSCG00000010400	MSMB	435.31	-1.33	0.11	-12.41	0.00	0.00
ENSSSCG00000011890	NR1I2	17.84	-1.33	0.46	-2.87	0.00	0.01

ENSSSCG00000026360	CLGN	2103.08	-1.33	0.07	-20.20	0.00	0.00
ENSSSCG00000004989	FBXO33	467.68	-1.33	0.10	-12.92	0.00	0.00
ENSSSCG00000029882	SPATA1	33.68	-1.33	0.34	-3.94	0.00	0.00
ENSSSCG00000039758		126.24	-1.33	0.19	-6.97	0.00	0.00
ENSSSCG00000024913	HYLS1	373.63	-1.33	0.11	-12.40	0.00	0.00
ENSSSCG00000000066	L3MBTL2	1542.69	-1.33	0.07	-20.30	0.00	0.00
ENSSSCG00000013478	DOHH	312.54	-1.33	0.12	-11.34	0.00	0.00
ENSSSCG00000007089		2860.34	-1.33	0.06	-22.79	0.00	0.00
ENSSSCG00000016714	CYCS	3093.42	-1.33	0.06	-23.39	0.00	0.00
ENSSSCG00000011848	TFRC	4458.12	-1.33	0.07	-19.19	0.00	0.00
ENSSSCG00000033732	ANKRD10	805.25	-1.33	0.08	-17.21	0.00	0.00
ENSSSCG00000008727	MSX1	456.18	-1.33	0.10	-13.39	0.00	0.00
ENSSSCG00000037277	RFK	541.33	-1.33	0.09	-14.57	0.00	0.00
ENSSSCG00000027334	EIF1AD	1207.33	-1.33	0.08	-16.82	0.00	0.00
ENSSSCG00000025134	FAM171B	17.56	-1.32	0.47	-2.83	0.00	0.01
ENSSSCG00000031686	FAM104A	1101.95	-1.32	0.07	-18.80	0.00	0.00
ENSSSCG00000005611	ZNF79	400.85	-1.32	0.11	-11.79	0.00	0.00
ENSSSCG00000040571	SMIM20	845.24	-1.32	0.09	-15.09	0.00	0.00
ENSSSCG00000016100		648.91	-1.32	0.09	-14.56	0.00	0.00
ENSSSCG00000021652	LSM8	976.11	-1.32	0.08	-17.52	0.00	0.00
ENSSSCG00000029918	CEPT1	968.87	-1.32	0.08	-17.28	0.00	0.00
ENSSSCG00000031612	ZNF570	118.61	-1.32	0.19	-6.81	0.00	0.00
ENSSSCG00000016189	AAMP	1612.06	-1.32	0.06	-21.42	0.00	0.00
ENSSSCG00000011827	LSG1	1634.96	-1.32	0.06	-20.66	0.00	0.00
ENSSSCG00000026812	EMG1	1828.44	-1.32	0.07	-18.60	0.00	0.00

ENSSSCG00000034087	TNFSF15	305.00	-1.32	0.12	-10.82	0.00	0.00
ENSSSCG00000033833		952.82	-1.32	0.07	-18.50	0.00	0.00
ENSSSCG00000026904	NFKBIB	1237.76	-1.32	0.07	-18.83	0.00	0.00
ENSSSCG00000008613	GEN1	796.47	-1.32	0.08	-17.07	0.00	0.00
ENSSSCG00000007965	ZNF200	544.41	-1.32	0.10	-13.63	0.00	0.00
ENSSSCG00000016087	TYW5	409.15	-1.32	0.11	-12.23	0.00	0.00
ENSSSCG00000030507	SMNDC1	964.02	-1.32	0.08	-16.11	0.00	0.00
ENSSSCG00000002802	GINS3	190.34	-1.32	0.16	-8.27	0.00	0.00
ENSSSCG00000010560	MRPL43	803.64	-1.32	0.08	-15.90	0.00	0.00
ENSSSCG00000026806	DHX9	7791.34	-1.32	0.05	-26.37	0.00	0.00
ENSSSCG00000000180	FKBP11	666.95	-1.32	0.09	-14.94	0.00	0.00
ENSSSCG00000012757		1472.89	-1.32	0.07	-18.83	0.00	0.00
ENSSSCG00000016191		532.48	-1.32	0.10	-12.87	0.00	0.00
ENSSSCG00000017396	PSMC3IP	406.39	-1.32	0.11	-11.91	0.00	0.00
ENSSSCG00000011051	HSPA14	652.88	-1.32	0.09	-15.30	0.00	0.00
ENSSSCG00000034632	PDXP	610.72	-1.32	0.09	-14.14	0.00	0.00
ENSSSCG00000003610	IQCC	223.44	-1.32	0.14	-9.57	0.00	0.00
ENSSSCG00000006090	MTERF3	202.30	-1.32	0.14	-9.29	0.00	0.00
ENSSSCG00000003612	EIF3I	2615.87	-1.31	0.06	-22.84	0.00	0.00
ENSSSCG00000012486	CSTF2	1308.88	-1.31	0.07	-18.52	0.00	0.00
ENSSSCG00000001866	RCN2	1005.49	-1.31	0.07	-18.11	0.00	0.00
ENSSSCG00000010105	SMPD4	1585.13	-1.31	0.06	-20.36	0.00	0.00
ENSSSCG00000017297	CYB561	1317.46	-1.31	0.07	-18.07	0.00	0.00
ENSSSCG00000039659	TIMM10	154.73	-1.31	0.16	-8.09	0.00	0.00
ENSSSCG00000035820	TXNDC17	1810.73	-1.31	0.06	-21.58	0.00	0.00

ENSSSCG00000036883	FABP3	4366.92	-1.31	0.05	-24.54	0.00	0.00
ENSSSCG00000038042	MRPS18C	236.18	-1.31	0.13	-9.85	0.00	0.00
ENSSSCG00000017766		333.65	-1.31	0.11	-11.60	0.00	0.00
ENSSSCG00000031835	GATAD1	1064.12	-1.31	0.07	-18.63	0.00	0.00
ENSSSCG00000031342		90.35	-1.31	0.22	-5.99	0.00	0.00
ENSSSCG00000016745	DDX56	831.57	-1.31	0.08	-16.47	0.00	0.00
ENSSSCG00000016275	NCL	14636.90	-1.31	0.05	-26.99	0.00	0.00
ENSSSCG00000039166	WTIP	73.95	-1.30	0.23	-5.60	0.00	0.00
ENSSSCG00000017246	C17orf80	344.40	-1.30	0.12	-10.92	0.00	0.00
ENSSSCG00000009796	LRRC43	212.63	-1.30	0.14	-9.36	0.00	0.00
ENSSSCG00000017913	PSMB6	2837.40	-1.30	0.05	-24.73	0.00	0.00
ENSSSCG00000016127	NDUFS1	3154.08	-1.30	0.05	-24.91	0.00	0.00
ENSSSCG00000008953	CXCL8	12654.07	-1.30	0.11	-12.31	0.00	0.00
ENSSSCG00000017430	EIF1	7952.20	-1.30	0.04	-29.74	0.00	0.00
ENSSSCG00000010337	MAT1A	87.66	-1.30	0.22	-5.78	0.00	0.00
ENSSSCG00000007622		666.11	-1.30	0.08	-15.75	0.00	0.00
ENSSSCG00000027287	TMEM189	2778.33	-1.30	0.05	-23.69	0.00	0.00
ENSSSCG00000011550	CAMK1	844.59	-1.30	0.08	-15.80	0.00	0.00
ENSSSCG00000011556		19.54	-1.30	0.46	-2.84	0.00	0.01
ENSSSCG00000039074	TNFRSF13C	31.94	-1.30	0.36	-3.58	0.00	0.00
ENSSSCG00000007607	KPNA7	11.39	-1.30	0.57	-2.28	0.02	0.03
ENSSSCG00000039220	RF00151	10.94	-1.30	0.64	-2.03	0.04	0.06
ENSSSCG00000027089	DONSON	1394.10	-1.30	0.06	-20.22	0.00	0.00
ENSSSCG00000024752	ALDH4A1	2635.48	-1.30	0.05	-24.02	0.00	0.00
ENSSSCG00000028202	RANGAP1	972.20	-1.30	0.08	-16.86	0.00	0.00

ENSSSCG00000008056	ATP6V0C	6137.50	-1.29	0.06	-22.63	0.00	0.00
ENSSSCG00000037734	UBE2M	2137.33	-1.29	0.06	-21.98	0.00	0.00
ENSSSCG00000014432	GRPEL2	617.82	-1.29	0.09	-14.80	0.00	0.00
ENSSSCG00000013049	RCOR2	557.11	-1.29	0.10	-13.13	0.00	0.00
ENSSSCG00000016206	CNPPD1	628.26	-1.29	0.09	-13.82	0.00	0.00
ENSSSCG00000024300	TMEM254	1865.51	-1.29	0.06	-20.52	0.00	0.00
ENSSSCG00000017213		157.34	-1.29	0.17	-7.75	0.00	0.00
ENSSSCG00000026910		57.40	-1.29	0.26	-4.91	0.00	0.00
ENSSSCG00000028638	UTP4	3150.31	-1.29	0.06	-22.19	0.00	0.00
ENSSSCG00000017490	GSDMA	8.94	-1.29	0.65	-1.97	0.05	0.07
ENSSSCG00000022351	GSTO1	585.60	-1.29	0.09	-13.63	0.00	0.00
ENSSSCG00000015072	PCSK7	895.35	-1.29	0.09	-14.75	0.00	0.00
ENSSSCG00000025488	MCM3	3141.03	-1.29	0.06	-22.38	0.00	0.00
ENSSSCG00000017379	ETV4	1219.08	-1.29	0.07	-18.49	0.00	0.00
ENSSSCG00000036893	PTHLH	63.54	-1.29	0.25	-5.21	0.00	0.00
ENSSSCG00000007786	RNF40	2926.99	-1.29	0.06	-22.30	0.00	0.00
ENSSSCG00000033135	VAMP5	234.05	-1.29	0.13	-9.61	0.00	0.00
ENSSSCG00000017882	MYBBP1A	6658.52	-1.29	0.05	-24.95	0.00	0.00
ENSSSCG00000037324		276.77	-1.29	0.12	-10.34	0.00	0.00
ENSSSCG00000040060		3408.00	-1.29	0.06	-21.04	0.00	0.00
ENSSSCG00000007179	STK35	1154.97	-1.29	0.07	-19.04	0.00	0.00
ENSSSCG00000016260	SLC16A14	35.44	-1.28	0.33	-3.87	0.00	0.00
ENSSSCG00000027070	ENTPD6	461.43	-1.28	0.10	-13.19	0.00	0.00
ENSSSCG00000035374	TRAPPC6A	472.83	-1.28	0.09	-13.52	0.00	0.00
ENSSSCG00000020754	CD3EAP	659.48	-1.28	0.08	-15.40	0.00	0.00

ENSSSCG00000040883	TMEM199	1517.83	-1.28	0.07	-18.62	0.00	0.00
ENSSSCG00000008648	RSAD2	10.65	-1.28	0.59	-2.17	0.03	0.04
ENSSSCG00000017239		5147.98	-1.28	0.05	-26.35	0.00	0.00
ENSSSCG00000029179	HIGD2A	1151.27	-1.28	0.07	-18.25	0.00	0.00
ENSSSCG00000032390	NDUFB2	382.63	-1.28	0.11	-12.15	0.00	0.00
ENSSSCG00000011904	UPK1B	595.91	-1.28	0.10	-12.96	0.00	0.00
ENSSSCG00000010514	RRP12	1515.31	-1.28	0.08	-16.74	0.00	0.00
ENSSSCG00000013775	ADGRE5	4289.06	-1.28	0.06	-23.17	0.00	0.00
ENSSSCG00000039295	MRPL34	411.21	-1.28	0.11	-11.99	0.00	0.00
ENSSSCG00000034012	CASP3	2251.78	-1.28	0.07	-19.06	0.00	0.00
ENSSSCG00000010477	CEP55	1345.64	-1.28	0.07	-17.42	0.00	0.00
ENSSSCG00000011350	CCDC51	205.25	-1.28	0.14	-8.97	0.00	0.00
ENSSSCG00000000895	SNRPF	621.96	-1.28	0.09	-14.81	0.00	0.00
ENSSSCG00000039757	SF3A2	1123.28	-1.27	0.07	-17.96	0.00	0.00
ENSSSCG00000032043	SLC35D1	467.71	-1.27	0.10	-12.73	0.00	0.00
ENSSSCG00000007559	MAFK	92.44	-1.27	0.20	-6.24	0.00	0.00
ENSSSCG00000010527	MARVELD1	119.54	-1.27	0.21	-6.21	0.00	0.00
ENSSSCG00000039255	RF00287	65.46	-1.27	0.25	-5.17	0.00	0.00
ENSSSCG00000017371	TMEM101	452.37	-1.27	0.10	-12.76	0.00	0.00
ENSSSCG00000040905	FAAP24	376.16	-1.27	0.11	-11.82	0.00	0.00
ENSSSCG00000015227	SRPRA	4173.72	-1.27	0.05	-27.74	0.00	0.00
ENSSSCG00000006830		2029.26	-1.27	0.06	-22.01	0.00	0.00
ENSSSCG00000013644	KRI1	936.38	-1.27	0.08	-16.69	0.00	0.00
ENSSSCG00000000058	SNU13	2522.32	-1.27	0.06	-20.84	0.00	0.00
ENSSSCG00000003267	MBOAT7	1423.66	-1.27	0.07	-18.09	0.00	0.00

ENSSSCG00000033613	FOXS1	379.50	-1.27	0.11	-11.35	0.00	0.00
ENSSSCG00000014989	DCUN1D5	518.22	-1.27	0.09	-13.83	0.00	0.00
ENSSSCG00000002470	DDX24	2872.61	-1.27	0.05	-23.58	0.00	0.00
ENSSSCG00000030421	GDI1	4684.01	-1.27	0.05	-23.10	0.00	0.00
ENSSSCG00000005219	CDC37L1	472.28	-1.27	0.10	-12.41	0.00	0.00
ENSSSCG00000023680	MEPCE	2025.31	-1.27	0.07	-18.88	0.00	0.00
ENSSSCG00000003876	CDKN2C	1331.20	-1.27	0.07	-19.25	0.00	0.00
ENSSSCG00000039654	NIF3L1	644.62	-1.27	0.08	-15.07	0.00	0.00
ENSSSCG00000005859	NDOR1	487.06	-1.27	0.10	-12.50	0.00	0.00
ENSSSCG00000000487	SLC35E3	593.20	-1.27	0.09	-13.60	0.00	0.00
ENSSSCG00000034185	RF00045	16.63	-1.27	0.48	-2.66	0.01	0.01
ENSSSCG00000014803	LRRC51	90.93	-1.27	0.21	-6.01	0.00	0.00
ENSSSCG00000034798	JKAMP	819.83	-1.27	0.08	-16.08	0.00	0.00
ENSSSCG00000038626	ZNF513	119.82	-1.27	0.18	-6.91	0.00	0.00
ENSSSCG00000026502	LIX1L	1496.55	-1.26	0.06	-20.39	0.00	0.00
ENSSSCG00000009937	ACACB	25.81	-1.26	0.39	-3.22	0.00	0.00
ENSSSCG00000014326	KIF20A	4392.79	-1.26	0.06	-22.80	0.00	0.00
ENSSSCG00000007991	WDR90	21.71	-1.26	0.46	-2.75	0.01	0.01
ENSSSCG00000002707	GABARAPL2	1111.70	-1.26	0.08	-16.47	0.00	0.00
ENSSSCG00000035863	PLIN2	5018.01	-1.26	0.06	-22.90	0.00	0.00
ENSSSCG00000034905	MVD	393.12	-1.26	0.11	-11.97	0.00	0.00
ENSSSCG00000017519	MRPL10	979.44	-1.26	0.07	-17.25	0.00	0.00
ENSSSCG00000027587	SPAG1	870.43	-1.26	0.08	-16.46	0.00	0.00
ENSSSCG00000024841	SLC35A2	744.02	-1.26	0.08	-15.50	0.00	0.00
ENSSSCG00000024823	RCN3	360.86	-1.26	0.12	-10.70	0.00	0.00

ENSSSCG00000028025	WDR74	615.62	-1.26	0.09	-14.70	0.00	0.00
ENSSSCG00000040413	RF01891	198.41	-1.26	0.15	-8.45	0.00	0.00
ENSSSCG00000033171	MAPK11	230.99	-1.26	0.13	-9.61	0.00	0.00
ENSSSCG00000022507	POLR2H	708.11	-1.26	0.10	-13.22	0.00	0.00
ENSSSCG00000008590	MFSD2B	20.39	-1.26	0.44	-2.85	0.00	0.01
ENSSSCG00000022647	TIMM17B	714.47	-1.26	0.11	-11.96	0.00	0.00
ENSSSCG00000033744		669.14	-1.26	0.09	-14.18	0.00	0.00
ENSSSCG00000036783	CCDC127	510.42	-1.26	0.10	-12.49	0.00	0.00
ENSSSCG00000014289	UQCRQ	2552.69	-1.25	0.06	-20.52	0.00	0.00
ENSSSCG00000000842	KANSL2	994.83	-1.25	0.08	-15.81	0.00	0.00
ENSSSCG00000040349	HOXD8	1061.17	-1.25	0.08	-16.34	0.00	0.00
ENSSSCG00000003339	INTS11	1351.53	-1.25	0.07	-18.63	0.00	0.00
ENSSSCG00000015094	CD3G	28.63	-1.25	0.36	-3.45	0.00	0.00
ENSSSCG00000017398	COASY	1141.32	-1.25	0.07	-18.59	0.00	0.00
ENSSSCG00000012617	UPF3B	1568.20	-1.25	0.07	-17.94	0.00	0.00
ENSSSCG00000015779	CDKN2AIP	526.64	-1.25	0.10	-12.94	0.00	0.00
ENSSSCG00000026079	TKTL2	47.48	-1.25	0.29	-4.30	0.00	0.00
ENSSSCG00000026568	DNPH1	247.81	-1.25	0.13	-9.30	0.00	0.00
ENSSSCG00000005629	PIP5KL1	43.06	-1.25	0.30	-4.18	0.00	0.00
ENSSSCG00000025943	C6orf136	332.01	-1.25	0.12	-10.63	0.00	0.00
ENSSSCG00000034450	ABHD10	453.67	-1.25	0.11	-11.83	0.00	0.00
ENSSSCG00000017446		100.03	-1.25	0.21	-6.06	0.00	0.00
ENSSSCG00000007715	ABHD11	1103.39	-1.25	0.08	-15.75	0.00	0.00
ENSSSCG00000012386	FAM155B	666.31	-1.25	0.08	-14.95	0.00	0.00
ENSSSCG00000017248		2612.36	-1.25	0.05	-22.70	0.00	0.00

ENSSSCG00000017378		1312.26	-1.24	0.07	-18.32	0.00	0.00
ENSSSCG00000035728		989.85	-1.24	0.07	-16.82	0.00	0.00
ENSSSCG00000039905	C1orf109	1019.05	-1.24	0.07	-16.77	0.00	0.00
ENSSSCG00000017395	RETREG3	1326.18	-1.24	0.06	-19.41	0.00	0.00
ENSSSCG00000013082	SDHAF2	824.64	-1.24	0.09	-14.47	0.00	0.00
ENSSSCG00000000005	CDPF1	170.48	-1.24	0.16	-8.00	0.00	0.00
ENSSSCG00000000405		14967.39	-1.24	0.04	-28.30	0.00	0.00
ENSSSCG00000013635	TIMM29	440.19	-1.24	0.10	-12.29	0.00	0.00
ENSSSCG00000009625		863.11	-1.24	0.08	-16.24	0.00	0.00
ENSSSCG00000032279	SAPCD2	171.74	-1.24	0.16	-7.64	0.00	0.00
ENSSSCG00000025151		2579.39	-1.24	0.06	-21.29	0.00	0.00
ENSSSCG00000017809	GEMIN4	3570.82	-1.24	0.05	-24.40	0.00	0.00
ENSSSCG00000003069	KCNN4	18.85	-1.24	0.45	-2.74	0.01	0.01
ENSSSCG00000017231	NAT9	238.52	-1.24	0.13	-9.36	0.00	0.00
ENSSSCG00000008352		1164.38	-1.24	0.07	-17.67	0.00	0.00
ENSSSCG00000032599		462.84	-1.24	0.32	-3.83	0.00	0.00
ENSSSCG00000017196	MRPL38	2053.41	-1.24	0.07	-18.12	0.00	0.00
ENSSSCG00000030328	NDUFB10	1143.43	-1.24	0.08	-16.23	0.00	0.00
ENSSSCG00000004733	PLA2G4D	32.96	-1.23	0.33	-3.70	0.00	0.00
ENSSSCG00000001531	SNRPC	1236.32	-1.23	0.07	-16.68	0.00	0.00
ENSSSCG00000029005	KPNA2	5756.84	-1.23	0.05	-26.74	0.00	0.00
ENSSSCG00000010444	LIPM	77.09	-1.23	0.23	-5.42	0.00	0.00
ENSSSCG00000028698	CCDC47	2913.80	-1.23	0.05	-23.23	0.00	0.00
ENSSSCG00000038969		91.66	-1.23	0.22	-5.72	0.00	0.00
ENSSSCG00000017346	EFTUD2	6738.51	-1.23	0.05	-23.55	0.00	0.00

ENSSSCG00000021319	ALG3	1091.71	-1.23	0.08	-15.10	0.00	0.00
ENSSSCG00000028553	ILF2	7826.45	-1.23	0.05	-25.79	0.00	0.00
ENSSSCG00000033854	LGALS1	8465.62	-1.23	0.04	-28.48	0.00	0.00
ENSSSCG00000007732	SBDS	2272.57	-1.23	0.06	-19.20	0.00	0.00
ENSSSCG00000035594	PRSS22	702.46	-1.23	0.08	-14.95	0.00	0.00
ENSSSCG00000005478	POLE3	2479.22	-1.23	0.06	-19.61	0.00	0.00
ENSSSCG00000039053	VGF	1316.04	-1.23	0.07	-16.73	0.00	0.00
ENSSSCG00000021275	DDX52	851.25	-1.23	0.08	-16.36	0.00	0.00
ENSSSCG00000027767	SHLD2	62.06	-1.23	0.25	-5.01	0.00	0.00
ENSSSCG00000032842	GIPC1	674.65	-1.23	0.09	-13.45	0.00	0.00
ENSSSCG00000034562	TMEM203	418.15	-1.23	0.10	-11.77	0.00	0.00
ENSSSCG00000011855	IQCG	13.79	-1.23	0.56	-2.18	0.03	0.04
ENSSSCG00000015034	SDHD	1878.20	-1.23	0.07	-18.84	0.00	0.00
ENSSSCG00000017199		414.72	-1.23	0.10	-12.12	0.00	0.00
ENSSSCG00000034137	PPIB	4118.92	-1.23	0.05	-23.80	0.00	0.00
ENSSSCG00000011486	C3orf14	54.61	-1.23	0.27	-4.61	0.00	0.00
ENSSSCG00000017362	ATXN7L3	889.42	-1.22	0.08	-15.85	0.00	0.00
ENSSSCG00000012940	B4GAT1	636.91	-1.22	0.10	-12.27	0.00	0.00
ENSSSCG00000010007	MTFP1	257.91	-1.22	0.15	-8.11	0.00	0.00
ENSSSCG00000033374	MRPL20	933.63	-1.22	0.08	-15.77	0.00	0.00
ENSSSCG00000035452	HTD2	2263.37	-1.22	0.06	-19.91	0.00	0.00
ENSSSCG00000040904	CLDN1	5139.34	-1.22	0.05	-22.88	0.00	0.00
ENSSSCG00000031772		9.82	-1.22	0.61	-2.01	0.04	0.06
ENSSSCG00000039267	ATP6V0B	2291.12	-1.22	0.06	-21.31	0.00	0.00
ENSSSCG00000016346	MAD2L1BP	358.78	-1.22	0.11	-11.38	0.00	0.00

ENSSSCG00000033524	KRR1	1065.74	-1.22	0.07	-16.41	0.00	0.00
ENSSSCG00000034913		2145.22	-1.22	0.06	-19.87	0.00	0.00
ENSSSCG00000013308	FBXO3	475.03	-1.22	0.10	-12.14	0.00	0.00
ENSSSCG00000034358	KCNJ16	184.67	-1.22	0.15	-7.88	0.00	0.00
ENSSSCG00000028423	RBBP4	8123.22	-1.22	0.05	-24.74	0.00	0.00
ENSSSCG00000012974	CFL1	11303.05	-1.22	0.05	-23.37	0.00	0.00
ENSSSCG00000001658	MRPL2	501.61	-1.22	0.11	-11.05	0.00	0.00
ENSSSCG00000033325	TRIM35	177.99	-1.22	0.15	-8.23	0.00	0.00
ENSSSCG00000036673	PPT1	2972.70	-1.22	0.05	-22.37	0.00	0.00
ENSSSCG00000028007	POLR1C	1079.50	-1.22	0.07	-16.61	0.00	0.00
ENSSSCG00000040893	ZNF488	53.33	-1.22	0.27	-4.58	0.00	0.00
ENSSSCG00000027847	CSKMT	54.42	-1.22	0.26	-4.70	0.00	0.00
ENSSSCG00000021620	STIP1	5667.82	-1.22	0.05	-25.50	0.00	0.00
ENSSSCG00000028807	RF00190	15.51	-1.22	0.51	-2.39	0.02	0.02
ENSSSCG00000000728	PARP11	97.30	-1.22	0.21	-5.92	0.00	0.00
ENSSSCG00000011342	DHX30	2479.85	-1.22	0.06	-21.15	0.00	0.00
ENSSSCG00000000264	MFSD5	499.35	-1.22	0.10	-11.82	0.00	0.00
ENSSSCG00000024126	CCT5	10988.05	-1.22	0.05	-26.69	0.00	0.00
ENSSSCG00000021084	S100A6	12156.40	-1.22	0.05	-22.54	0.00	0.00
ENSSSCG00000013896	MPV17L2	584.31	-1.22	0.09	-12.81	0.00	0.00
ENSSSCG00000032164	PEA15	5239.68	-1.21	0.07	-18.39	0.00	0.00
ENSSSCG00000032165	ATP5MD	1231.91	-1.21	0.07	-16.55	0.00	0.00
ENSSSCG00000026731	CEP85	619.46	-1.21	0.09	-14.08	0.00	0.00
ENSSSCG00000026772	C1orf52	286.14	-1.21	0.13	-9.44	0.00	0.00
ENSSSCG00000001703	NFKBIE	1411.57	-1.21	0.06	-19.67	0.00	0.00

ENSSSCG00000007803	TUFM	4457.89	-1.21	0.05	-24.85	0.00	0.00
ENSSSCG00000012914	RAD9A	604.48	-1.21	0.09	-12.82	0.00	0.00
ENSSSCG00000002346	PNMA1	200.22	-1.21	0.14	-8.64	0.00	0.00
ENSSSCG00000020783	SLC41A1	1502.98	-1.21	0.06	-19.29	0.00	0.00
ENSSSCG00000004884	TMX3	965.26	-1.21	0.07	-16.86	0.00	0.00
ENSSSCG00000009460	DIS3	1095.58	-1.21	0.07	-16.61	0.00	0.00
ENSSSCG00000037046	TUBB4B	6767.51	-1.21	0.06	-21.80	0.00	0.00
ENSSSCG00000014915	EED	434.56	-1.21	0.10	-12.25	0.00	0.00
ENSSSCG00000040825	PYM1	344.48	-1.21	0.11	-10.85	0.00	0.00
ENSSSCG00000017548	NGFR	106.66	-1.21	0.20	-5.95	0.00	0.00
ENSSSCG00000029874	DUSP14	464.65	-1.21	0.10	-11.84	0.00	0.00
ENSSSCG00000023557	CCRL2	27.56	-1.21	0.37	-3.27	0.00	0.00
ENSSSCG00000023998	PSMD2	10294.83	-1.21	0.04	-27.89	0.00	0.00
ENSSSCG00000031666	C11orf95	44.48	-1.21	0.31	-3.87	0.00	0.00
ENSSSCG00000030637	IRGQ	704.01	-1.21	0.08	-14.63	0.00	0.00
ENSSSCG00000038359		11924.56	-1.21	0.05	-23.95	0.00	0.00
ENSSSCG00000028465	ELAC2	1381.07	-1.21	0.07	-16.97	0.00	0.00
ENSSSCG00000006073	OSR2	574.17	-1.21	0.10	-11.84	0.00	0.00
ENSSSCG00000038370	UQCC3	414.14	-1.21	0.10	-11.64	0.00	0.00
ENSSSCG00000035693	POLRMT	1631.06	-1.21	0.07	-17.88	0.00	0.00
ENSSSCG00000032914	MANF	881.70	-1.20	0.08	-15.74	0.00	0.00
ENSSSCG00000032903		364.96	-1.20	0.11	-11.04	0.00	0.00
ENSSSCG00000037880	NSMF	2230.09	-1.20	0.06	-19.94	0.00	0.00
ENSSSCG00000007073	ISM1	23.08	-1.20	0.41	-2.91	0.00	0.01
ENSSSCG00000016583	FAM71F2	19.49	-1.20	0.44	-2.73	0.01	0.01

ENSSSCG00000011587	EFCAB12	220.69	-1.20	0.14	-8.64	0.00	0.00
ENSSSCG00000032080	ZNF35	255.22	-1.20	0.13	-9.58	0.00	0.00
ENSSSCG00000015040		1184.74	-1.20	0.08	-15.42	0.00	0.00
ENSSSCG00000029403	EMC8	910.49	-1.20	0.08	-15.66	0.00	0.00
ENSSSCG00000026837	RF00087	19.42	-1.20	0.45	-2.65	0.01	0.01
ENSSSCG00000036852	TSEN54	531.24	-1.20	0.09	-13.00	0.00	0.00
ENSSSCG00000004487	MTO1	860.23	-1.20	0.08	-14.83	0.00	0.00
ENSSSCG00000032180	MCM7	3811.56	-1.20	0.05	-22.34	0.00	0.00
ENSSSCG00000038172	PIP4P1	356.10	-1.20	0.11	-11.22	0.00	0.00
ENSSSCG00000035538		2447.70	-1.20	0.05	-22.20	0.00	0.00
ENSSSCG00000039307	H2AFJ	178.42	-1.20	0.15	-8.13	0.00	0.00
ENSSSCG00000040236		50.15	-1.20	0.30	-4.05	0.00	0.00
ENSSSCG00000016960	RAD17	854.51	-1.20	0.08	-15.05	0.00	0.00
ENSSSCG00000010235	SIRT1	723.32	-1.20	0.08	-14.60	0.00	0.00
ENSSSCG00000033823	XPNPEP3	973.14	-1.20	0.08	-15.23	0.00	0.00
ENSSSCG00000021597	PHLDA2	1370.66	-1.20	0.07	-17.40	0.00	0.00
ENSSSCG00000040265	UBFD1	1021.44	-1.20	0.07	-16.28	0.00	0.00
ENSSSCG00000023467		545.12	-1.20	0.19	-6.16	0.00	0.00
ENSSSCG00000002907		2197.19	-1.20	0.06	-19.23	0.00	0.00
ENSSSCG00000032213	DBI	2070.84	-1.20	0.06	-20.75	0.00	0.00
ENSSSCG00000036494	ERG28	2152.68	-1.20	0.07	-18.07	0.00	0.00
ENSSSCG00000023870	RF00581	22.81	-1.20	0.42	-2.88	0.00	0.01
ENSSSCG00000002906	ETV2	25.32	-1.19	0.39	-3.08	0.00	0.00
ENSSSCG00000038442	TMLHE	2130.49	-1.19	0.06	-20.99	0.00	0.00
ENSSSCG00000016793	ZNF622	630.16	-1.19	0.08	-14.05	0.00	0.00

ENSSSCG00000027057	SYVN1	263.07	-1.19	0.13	-9.21	0.00	0.00
ENSSSCG00000040352	DUS1L	1888.92	-1.19	0.06	-19.05	0.00	0.00
ENSSSCG00000017397	MLX	887.07	-1.19	0.08	-15.50	0.00	0.00
ENSSSCG00000013380		2400.43	-1.19	0.06	-19.77	0.00	0.00
ENSSSCG00000018229	RF00284	43.39	-1.19	0.29	-4.09	0.00	0.00
ENSSSCG00000001788	STARD5	197.79	-1.19	0.15	-8.07	0.00	0.00
ENSSSCG00000016826	RAD1	508.81	-1.19	0.10	-12.47	0.00	0.00
ENSSSCG00000028924	AURKB	1469.68	-1.19	0.07	-17.60	0.00	0.00
ENSSSCG00000008870	GUCY1B1	1256.46	-1.19	0.07	-17.42	0.00	0.00
ENSSSCG00000037127	CDKN2AIPNL	1169.23	-1.19	0.07	-17.90	0.00	0.00
ENSSSCG00000000523	BBS10	98.88	-1.19	0.20	-5.92	0.00	0.00
ENSSSCG00000012789	AVPR2	16.70	-1.19	0.47	-2.54	0.01	0.02
ENSSSCG00000017539	HOXB6	617.07	-1.19	0.09	-13.12	0.00	0.00
ENSSSCG00000027718		902.39	-1.19	0.08	-14.64	0.00	0.00
ENSSSCG00000011565	BRK1	2469.80	-1.19	0.06	-19.88	0.00	0.00
ENSSSCG00000040071		116.25	-1.19	0.18	-6.62	0.00	0.00
ENSSSCG00000039390	EIF3B	6306.65	-1.19	0.05	-25.72	0.00	0.00
ENSSSCG00000023636	TMEM222	686.95	-1.19	0.08	-14.35	0.00	0.00
ENSSSCG00000026083	DNAJC24	232.08	-1.19	0.14	-8.78	0.00	0.00
ENSSSCG00000037686	TBL3	1402.97	-1.19	0.07	-16.58	0.00	0.00
ENSSSCG00000017994	CFAP52	169.92	-1.19	0.15	-7.87	0.00	0.00
ENSSSCG00000006391	ATP1A2	115.02	-1.19	0.18	-6.43	0.00	0.00
ENSSSCG00000018935	RF00221	23.61	-1.19	0.40	-2.93	0.00	0.01
ENSSSCG00000017915	VMO1	572.15	-1.19	0.09	-12.91	0.00	0.00
ENSSSCG00000029029	ZNF713	32.05	-1.18	0.35	-3.41	0.00	0.00

ENSSSCG00000003617	TXLNA	2001.18	-1.18	0.06	-18.47	0.00	0.00
ENSSSCG00000031076	MAPK7	2386.22	-1.18	0.07	-17.19	0.00	0.00
ENSSSCG00000010308	CHCHD1	222.69	-1.18	0.14	-8.54	0.00	0.00
ENSSSCG00000028509	RBM8A	1654.31	-1.18	0.06	-18.74	0.00	0.00
ENSSSCG00000025717	HIC1	129.16	-1.18	0.17	-6.94	0.00	0.00
ENSSSCG00000031125	AMPD2	1369.82	-1.18	0.08	-15.65	0.00	0.00
ENSSSCG00000032228	C8orf89	16.53	-1.18	0.48	-2.45	0.01	0.02
ENSSSCG00000012823	DKC1	2093.00	-1.18	0.06	-18.34	0.00	0.00
ENSSSCG00000032936	PIM3	278.40	-1.18	0.13	-9.38	0.00	0.00
ENSSSCG00000006080	ERICH5	310.39	-1.18	0.12	-10.03	0.00	0.00
ENSSSCG00000038765	FAM89A	151.86	-1.18	0.16	-7.48	0.00	0.00
ENSSSCG00000000363	GDF11	970.10	-1.18	0.07	-16.07	0.00	0.00
ENSSSCG00000039380	NANP	593.95	-1.18	0.09	-12.96	0.00	0.00
ENSSSCG00000008552	PRR30	41.24	-1.18	0.31	-3.85	0.00	0.00
ENSSSCG00000037963		25.05	-1.18	0.40	-2.97	0.00	0.00
ENSSSCG00000017342	KIF18B	1257.44	-1.18	0.07	-16.47	0.00	0.00
ENSSSCG00000026947	GMPR2	1286.57	-1.18	0.07	-16.27	0.00	0.00
ENSSSCG00000025651	GFM1	1495.23	-1.17	0.07	-17.22	0.00	0.00
ENSSSCG00000023861	PFAS	1428.89	-1.17	0.06	-18.65	0.00	0.00
ENSSSCG00000027529	BIRC3	7731.44	-1.17	0.06	-20.56	0.00	0.00
ENSSSCG00000014288	GDF9	314.84	-1.17	0.11	-10.44	0.00	0.00
ENSSSCG00000013062	MTA2	2527.95	-1.17	0.06	-18.18	0.00	0.00
ENSSSCG00000009877	IQCD	128.34	-1.17	0.18	-6.61	0.00	0.00
ENSSSCG00000012111	HCCS	974.99	-1.17	0.08	-15.05	0.00	0.00
ENSSSCG00000017127	CYBC1	1042.58	-1.17	0.08	-15.22	0.00	0.00

ENSSSCG00000027230	SLC35G1	326.99	-1.17	0.11	-10.29	0.00	0.00
ENSSSCG00000014258		859.88	-1.17	0.08	-14.57	0.00	0.00
ENSSSCG00000015443	DLD	5491.84	-1.17	0.05	-24.67	0.00	0.00
ENSSSCG00000031152		605.26	-1.17	0.09	-13.29	0.00	0.00
ENSSSCG00000006342	UHMK1	5472.27	-1.17	0.12	-9.59	0.00	0.00
ENSSSCG00000009807	RHOF	677.37	-1.17	0.09	-13.76	0.00	0.00
ENSSSCG00000007709	TBL2	761.75	-1.17	0.08	-14.11	0.00	0.00
ENSSSCG00000017990	PIK3R6	11.12	-1.17	0.57	-2.06	0.04	0.05
ENSSSCG00000009085	NUDT6	69.59	-1.17	0.24	-4.87	0.00	0.00
ENSSSCG00000033636	AFMID	1432.91	-1.17	0.06	-18.24	0.00	0.00
ENSSSCG00000014068	UTP15	1126.21	-1.17	0.07	-16.04	0.00	0.00
ENSSSCG00000017214	ARMC7	240.43	-1.17	0.13	-8.99	0.00	0.00
ENSSSCG00000039550	EMC4	1522.51	-1.17	0.06	-18.74	0.00	0.00
ENSSSCG00000008233	TMEM150A	161.50	-1.17	0.16	-7.19	0.00	0.00
ENSSSCG00000039613	SVIP	133.82	-1.17	0.17	-6.87	0.00	0.00
ENSSSCG00000004167	MYB	169.91	-1.17	0.15	-7.72	0.00	0.00
ENSSSCG00000000022	RTL6	498.45	-1.17	0.10	-11.42	0.00	0.00
ENSSSCG00000005453		5598.01	-1.17	0.05	-23.10	0.00	0.00
ENSSSCG00000002884	LSR	2353.37	-1.16	0.06	-19.63	0.00	0.00
ENSSSCG00000006729	TENT5C	555.14	-1.16	0.09	-12.69	0.00	0.00
ENSSSCG00000008494	NDUFAF7	617.97	-1.16	0.09	-12.68	0.00	0.00
ENSSSCG00000035341	C6orf89	927.77	-1.16	0.08	-13.87	0.00	0.00
ENSSSCG00000039397	ETNK1	1235.19	-1.16	0.08	-15.07	0.00	0.00
ENSSSCG00000017360	UBTF	1440.13	-1.16	0.07	-16.27	0.00	0.00
ENSSSCG00000037001	PPM1N	20.46	-1.16	0.45	-2.57	0.01	0.02

ENSSSCG00000020842	TAF13	1215.85	-1.16	0.16	-7.46	0.00	0.00
ENSSSCG00000024672	KLF16	754.81	-1.16	0.08	-14.58	0.00	0.00
ENSSSCG00000034835	SLC7A6	647.31	-1.16	0.08	-14.09	0.00	0.00
ENSSSCG00000032748		206.43	-1.16	0.14	-8.24	0.00	0.00
ENSSSCG00000033741	NIP7	868.80	-1.16	0.08	-13.90	0.00	0.00
ENSSSCG00000012397		265.99	-1.16	0.13	-9.25	0.00	0.00
ENSSSCG00000039592	ZMYND19	258.11	-1.16	0.13	-9.27	0.00	0.00
ENSSSCG00000023482	DNAJC9	1095.68	-1.16	0.07	-16.02	0.00	0.00
ENSSSCG00000013927	ARMC6	431.54	-1.16	0.11	-10.89	0.00	0.00
ENSSSCG00000016907	DHX29	1328.96	-1.16	0.07	-16.77	0.00	0.00
ENSSSCG00000036256	MAD2L1	2368.95	-1.16	0.07	-17.32	0.00	0.00
ENSSSCG00000037674		33.81	-1.16	0.38	-3.04	0.00	0.00
ENSSSCG00000013063		462.83	-1.16	0.10	-11.45	0.00	0.00
ENSSSCG00000011569		568.51	-1.16	0.09	-12.99	0.00	0.00
ENSSSCG00000006756	BCAS2	1515.07	-1.16	0.06	-18.36	0.00	0.00
ENSSSCG00000007935		77.26	-1.16	0.22	-5.25	0.00	0.00
ENSSSCG00000034984	PSMB4	3338.87	-1.16	0.05	-24.14	0.00	0.00
ENSSSCG00000000611		4506.59	-1.16	0.05	-21.35	0.00	0.00
ENSSSCG00000034887	HAPLN4	1905.80	-1.16	0.07	-16.67	0.00	0.00
ENSSSCG00000034633	CFAP99	15.35	-1.16	0.49	-2.34	0.02	0.03
ENSSSCG00000028712	ATPAF2	759.03	-1.16	0.08	-14.47	0.00	0.00
ENSSSCG00000012915	CLCF1	213.73	-1.16	0.14	-8.30	0.00	0.00
ENSSSCG00000025953	OGFOD1	926.98	-1.16	0.08	-14.36	0.00	0.00
ENSSSCG00000019612	RF00069	56.55	-1.15	0.27	-4.31	0.00	0.00
ENSSSCG00000008055	TEDC2	233.38	-1.15	0.14	-8.39	0.00	0.00

ENSSSCG00000030643	SELENOI	870.74	-1.15	0.08	-14.93	0.00	0.00
ENSSSCG00000007278	AHCY	9189.11	-1.15	0.05	-22.98	0.00	0.00
ENSSSCG00000000158	RTCB	1865.83	-1.15	0.07	-16.69	0.00	0.00
ENSSSCG00000005920	BOP1	1000.49	-1.15	0.08	-15.15	0.00	0.00
ENSSSCG00000038423	GLA	1680.13	-1.15	0.06	-18.56	0.00	0.00
ENSSSCG00000009638	RHOBTB2	970.32	-1.15	0.08	-14.52	0.00	0.00
ENSSSCG00000021834	ERCC6L	406.71	-1.15	0.10	-11.20	0.00	0.00
ENSSSCG00000001773	TMED3	590.97	-1.15	0.09	-12.16	0.00	0.00
ENSSSCG00000034615	TNFAIP8L1	448.59	-1.15	0.10	-11.98	0.00	0.00
ENSSSCG00000033968	SLC39A3	291.02	-1.15	0.12	-9.83	0.00	0.00
ENSSSCG00000033298	SAMD1	389.78	-1.15	0.11	-10.56	0.00	0.00
ENSSSCG00000017705	CCL5	351.12	-1.15	0.24	-4.88	0.00	0.00
ENSSSCG00000027076	CYHR1	915.14	-1.15	0.07	-15.51	0.00	0.00
ENSSSCG00000009962	TFIP11	995.86	-1.15	0.08	-15.04	0.00	0.00
ENSSSCG00000005671	CRAT	4690.17	-1.15	0.06	-19.06	0.00	0.00
ENSSSCG00000021418	LSS	1321.65	-1.15	0.07	-17.01	0.00	0.00
ENSSSCG00000038543	CENPA	1156.71	-1.15	0.07	-16.24	0.00	0.00
ENSSSCG00000038071	RASL12	10.98	-1.15	0.58	-1.97	0.05	0.07
ENSSSCG00000009145		1564.38	-1.15	0.06	-18.63	0.00	0.00
ENSSSCG00000035390	C2orf49	972.98	-1.15	0.07	-16.19	0.00	0.00
ENSSSCG00000036747	WAS	16.22	-1.15	0.49	-2.35	0.02	0.03
ENSSSCG00000040144	BCDIN3D	208.94	-1.15	0.14	-8.34	0.00	0.00
ENSSSCG00000015897	IFIH1	445.15	-1.15	0.11	-10.81	0.00	0.00
ENSSSCG00000007173	IDH3B	2348.29	-1.15	0.06	-19.49	0.00	0.00
ENSSSCG00000021718	METTL17	806.79	-1.15	0.08	-14.96	0.00	0.00

ENSSSCG00000007323	DSN1	1055.75	-1.15	0.07	-16.19	0.00	0.00
ENSSSCG00000032123	SPX	148.44	-1.15	0.16	-7.08	0.00	0.00
ENSSSCG00000028085		70.84	-1.15	0.23	-4.92	0.00	0.00
ENSSSCG00000034024	TMEM250	111.09	-1.14	0.20	-5.80	0.00	0.00
ENSSSCG00000015654	IL20	68.97	-1.14	0.26	-4.44	0.00	0.00
ENSSSCG00000004499	ATP5F1A	15252.07	-1.14	0.04	-26.85	0.00	0.00
ENSSSCG00000040721	ZNF581	15.10	-1.14	0.49	-2.32	0.02	0.03
ENSSSCG00000001990	NOP9	473.25	-1.14	0.10	-11.84	0.00	0.00
ENSSSCG00000003073	LYPD5	159.26	-1.14	0.16	-6.96	0.00	0.00
ENSSSCG00000015790	SLC25A4	895.03	-1.14	0.09	-12.69	0.00	0.00
ENSSSCG00000003768	NEXN	652.02	-1.14	0.08	-13.84	0.00	0.00
ENSSSCG00000040400	GPR19	399.70	-1.14	0.10	-10.87	0.00	0.00
ENSSSCG00000028466	UTP3	369.62	-1.14	0.12	-9.84	0.00	0.00
ENSSSCG00000005112	WDR89	117.19	-1.14	0.21	-5.46	0.00	0.00
ENSSSCG00000008303	CCT7	5846.03	-1.14	0.04	-25.74	0.00	0.00
ENSSSCG00000037099	ANKLE1	176.66	-1.14	0.15	-7.63	0.00	0.00
ENSSSCG00000017541	HOXB13	264.61	-1.14	0.13	-8.99	0.00	0.00
ENSSSCG00000010585	ACTR1A	2540.20	-1.14	0.05	-21.62	0.00	0.00
ENSSSCG00000002680	TAF1C	321.11	-1.14	0.12	-9.78	0.00	0.00
ENSSSCG00000025507	RPL27	8019.08	-1.14	0.05	-22.17	0.00	0.00
ENSSSCG00000026018	MFSD1	217.38	-1.14	0.14	-8.21	0.00	0.00
ENSSSCG00000022915	DDX27	1473.53	-1.14	0.07	-16.45	0.00	0.00
ENSSSCG00000000682	GNB3	30.61	-1.14	0.35	-3.24	0.00	0.00
ENSSSCG00000017518		662.81	-1.14	0.08	-13.88	0.00	0.00
ENSSSCG00000021161	CKS2	888.37	-1.14	0.07	-15.18	0.00	0.00

ENSSSCG00000013721		1435.33	-1.14	0.06	-17.91	0.00	0.00
ENSSSCG00000025571	POLR2G	974.20	-1.14	0.07	-15.63	0.00	0.00
ENSSSCG00000035771	MRPS22	731.01	-1.14	0.09	-13.20	0.00	0.00
ENSSSCG00000006187	MSC	12.00	-1.14	0.56	-2.02	0.04	0.06
ENSSSCG00000031738		37.15	-1.14	0.32	-3.55	0.00	0.00
ENSSSCG00000010607	COL17A1	5753.38	-1.14	0.05	-24.22	0.00	0.00
ENSSSCG00000038471	NUAK2	678.40	-1.13	0.09	-13.17	0.00	0.00
ENSSSCG00000018047	FAM83G	158.01	-1.13	0.16	-7.19	0.00	0.00
ENSSSCG00000024329	C12orf43	733.61	-1.13	0.08	-13.35	0.00	0.00
ENSSSCG00000011314	LZTFL1	571.10	-1.13	0.09	-12.57	0.00	0.00
ENSSSCG00000013990	GUK1	1150.30	-1.13	0.08	-14.26	0.00	0.00
ENSSSCG00000005858	RNF208	11.97	-1.13	0.55	-2.06	0.04	0.05
ENSSSCG00000000981	CRELD2	269.13	-1.13	0.12	-9.25	0.00	0.00
ENSSSCG00000008694	GRK4	85.50	-1.13	0.21	-5.32	0.00	0.00
ENSSSCG00000027429		983.55	-1.13	0.07	-15.48	0.00	0.00
ENSSSCG00000006572	NPR1	32.00	-1.13	0.34	-3.32	0.00	0.00
ENSSSCG00000035532	ATP5F1D	2225.30	-1.13	0.06	-19.41	0.00	0.00
ENSSSCG00000012621	UBE2A	3044.80	-1.13	0.06	-20.01	0.00	0.00
ENSSSCG00000016202	RNF25	788.15	-1.13	0.08	-14.64	0.00	0.00
ENSSSCG00000034990		78.86	-1.13	0.22	-5.09	0.00	0.00
ENSSSCG00000014255	SLC12A2	727.68	-1.13	0.08	-13.42	0.00	0.00
ENSSSCG00000000973	SELENOO	415.15	-1.13	0.10	-11.36	0.00	0.00
ENSSSCG00000015424	PSMC2	4850.49	-1.13	0.05	-25.00	0.00	0.00
ENSSSCG00000038399	ATP5PF	1074.24	-1.13	0.07	-15.73	0.00	0.00
ENSSSCG00000033450	PDAP1	1691.73	-1.13	0.06	-18.44	0.00	0.00

ENSSSCG00000026894	NFE2L3	134.83	-1.13	0.18	-6.42	0.00	0.00
ENSSSCG00000037228	RRAGB	1101.71	-1.13	0.07	-16.17	0.00	0.00
ENSSSCG00000030150	UGDH	2260.01	-1.13	0.06	-19.15	0.00	0.00
ENSSSCG00000015106	HYOU1	6169.56	-1.13	0.04	-25.87	0.00	0.00
ENSSSCG00000002036		2394.82	-1.13	0.06	-19.00	0.00	0.00
ENSSSCG00000011862	MUC13	194.42	-1.13	0.15	-7.45	0.00	0.00
ENSSSCG00000022225	NOP58	2224.85	-1.13	0.06	-17.60	0.00	0.00
ENSSSCG00000003613	HDAC1	2991.65	-1.13	0.06	-20.13	0.00	0.00
ENSSSCG00000008693	NOP14	286.73	-1.13	0.13	-8.96	0.00	0.00
ENSSSCG00000024679	UPF3A	67.38	-1.13	0.24	-4.62	0.00	0.00
ENSSSCG00000033180	PSMD10	831.87	-1.13	0.08	-14.44	0.00	0.00
ENSSSCG00000037102	FSTL3	1404.60	-1.13	0.07	-16.01	0.00	0.00
ENSSSCG00000004518		514.76	-1.13	0.09	-12.17	0.00	0.00
ENSSSCG00000034455	RNF166	575.96	-1.13	0.09	-11.99	0.00	0.00
ENSSSCG00000002366	NPC2	3683.45	-1.13	0.05	-22.72	0.00	0.00
ENSSSCG00000003644	FHL3	575.93	-1.13	0.10	-11.26	0.00	0.00
ENSSSCG00000000965	NCAPH2	1274.31	-1.13	0.07	-16.05	0.00	0.00
ENSSSCG00000012581	NXT2	1506.09	-1.12	0.07	-16.48	0.00	0.00
ENSSSCG00000014012	GFPT2	12.55	-1.12	0.56	-2.00	0.05	0.06
ENSSSCG00000030577	PBDC1	852.74	-1.12	0.08	-14.20	0.00	0.00
ENSSSCG00000037982	RF00278	31.44	-1.12	0.35	-3.26	0.00	0.00
ENSSSCG00000010686	BAG3	1228.38	-1.12	0.07	-16.09	0.00	0.00
ENSSSCG00000023739	RF00055	32.88	-1.12	0.35	-3.18	0.00	0.00
ENSSSCG00000025647	SLC35C1	289.78	-1.12	0.13	-8.55	0.00	0.00
ENSSSCG00000038491	MEX3B	168.20	-1.12	0.15	-7.34	0.00	0.00

ENSSSCG00000012014		1245.37	-1.12	0.07	-16.64	0.00	0.00
ENSSSCG00000017528	SNX11	212.56	-1.12	0.13	-8.35	0.00	0.00
ENSSSCG00000017538	HOXB7	660.94	-1.12	0.09	-13.11	0.00	0.00
ENSSSCG00000031918	CYC1	4471.75	-1.12	0.06	-18.56	0.00	0.00
ENSSSCG00000025722	RF00056	242.03	-1.12	0.13	-8.47	0.00	0.00
ENSSSCG00000003393	TMEM201	348.00	-1.12	0.12	-9.68	0.00	0.00
ENSSSCG00000008225	PTCD3	2930.71	-1.12	0.06	-19.27	0.00	0.00
ENSSSCG00000039969	THAP1	752.78	-1.12	0.08	-14.18	0.00	0.00
ENSSSCG00000006372	ARHGAP30	168.56	-1.12	0.17	-6.58	0.00	0.00
ENSSSCG00000000386	ANKRD52	2095.45	-1.12	0.06	-18.62	0.00	0.00
ENSSSCG00000000265	ESPL1	2203.92	-1.12	0.06	-19.09	0.00	0.00
ENSSSCG00000002708	TMEM231	373.87	-1.12	0.10	-10.76	0.00	0.00
ENSSSCG00000032313	POGLUT1	426.75	-1.12	0.10	-11.08	0.00	0.00
ENSSSCG00000013928		15.42	-1.12	0.49	-2.27	0.02	0.03
ENSSSCG00000038760	ANKRD40	2118.28	-1.12	0.05	-20.31	0.00	0.00
ENSSSCG00000016732	TBRG4	1433.00	-1.12	0.06	-17.34	0.00	0.00
ENSSSCG00000017957	EIF4A1	8155.91	-1.12	0.05	-21.35	0.00	0.00
ENSSSCG00000011351	TMA7	1267.94	-1.12	0.07	-15.32	0.00	0.00
ENSSSCG00000000396	STAT2	490.19	-1.11	0.10	-11.19	0.00	0.00
ENSSSCG00000026064		414.42	-1.11	0.11	-10.35	0.00	0.00
ENSSSCG00000034754	PLEKHS1	524.68	-1.11	0.10	-10.75	0.00	0.00
ENSSSCG00000003177	BCL2L12	136.23	-1.11	0.17	-6.72	0.00	0.00
ENSSSCG00000015755	AGPAT5	1483.31	-1.11	0.06	-17.60	0.00	0.00
ENSSSCG00000018034		588.71	-1.11	0.09	-11.87	0.00	0.00
ENSSSCG00000036741		216.03	-1.11	0.14	-7.85	0.00	0.00

ENSSSCG00000021472	POLR2E	1009.50	-1.11	0.08	-14.72	0.00	0.00
ENSSSCG00000014046	ZNF346	241.55	-1.11	0.14	-8.13	0.00	0.00
ENSSSCG00000027301		13.07	-1.11	0.54	-2.06	0.04	0.05
ENSSSCG00000003653	NDUFS5	891.44	-1.11	0.08	-14.28	0.00	0.00
ENSSSCG00000013886	B3GNT3	333.36	-1.11	0.12	-9.50	0.00	0.00
ENSSSCG00000017496	MIEN1	454.08	-1.11	0.10	-11.11	0.00	0.00
ENSSSCG00000014557	RIC8A	910.91	-1.11	0.08	-13.52	0.00	0.00
ENSSSCG00000038967	NADK	2745.48	-1.11	0.06	-19.67	0.00	0.00
ENSSSCG00000013358	PTPN5	14.44	-1.11	0.53	-2.10	0.04	0.05
ENSSSCG00000008901	PAICS	1421.99	-1.11	0.08	-14.44	0.00	0.00
ENSSSCG00000010073	CHCHD10	241.79	-1.11	0.13	-8.51	0.00	0.00
ENSSSCG00000017066	GEMIN5	2627.40	-1.11	0.06	-18.83	0.00	0.00
ENSSSCG00000040575	ISG15	483.69	-1.11	0.11	-10.02	0.00	0.00
ENSSSCG00000028167	SLIRP	462.47	-1.11	0.10	-11.15	0.00	0.00
ENSSSCG00000017232	SLC9A3R1	1708.98	-1.11	0.07	-15.43	0.00	0.00
ENSSSCG00000013539	GTF2F1	1133.82	-1.11	0.07	-15.20	0.00	0.00
ENSSSCG00000015140	HSPA8	12563.07	-1.11	0.06	-18.38	0.00	0.00
ENSSSCG00000007033	AP3M2	245.77	-1.11	0.13	-8.62	0.00	0.00
ENSSSCG00000006703	PRKAB2	1621.48	-1.11	0.06	-18.03	0.00	0.00
ENSSSCG00000032056		163.72	-1.11	0.16	-7.01	0.00	0.00
ENSSSCG00000034647	MRPS15	1137.60	-1.11	0.07	-15.54	0.00	0.00
ENSSSCG00000018092	ND6	112.42	-1.11	0.22	-5.03	0.00	0.00
ENSSSCG00000013107	PRPF19	2593.89	-1.11	0.06	-17.97	0.00	0.00
ENSSSCG00000005918	DGAT1	432.50	-1.11	0.11	-10.30	0.00	0.00
ENSSSCG00000002792	HSF4	134.44	-1.11	0.17	-6.44	0.00	0.00

ENSSSCG00000005643		1876.62	-1.10	0.06	-19.37	0.00	0.00
ENSSSCG00000029256	AKR1A1	1512.78	-1.10	0.07	-15.90	0.00	0.00
ENSSSCG00000012910	RPS6KB2	367.62	-1.10	0.11	-9.82	0.00	0.00
ENSSSCG00000000893	AMDHD1	115.48	-1.10	0.18	-6.05	0.00	0.00
ENSSSCG00000003018	LIPE	271.09	-1.10	0.12	-9.05	0.00	0.00
ENSSSCG00000029201	AJUBA	1142.64	-1.10	0.08	-13.65	0.00	0.00
ENSSSCG00000020809	B3GAT3	1159.52	-1.10	0.07	-15.92	0.00	0.00
ENSSSCG00000034527		136.38	-1.10	0.18	-6.14	0.00	0.00
ENSSSCG00000026754	ATCAY	34.65	-1.10	0.34	-3.28	0.00	0.00
ENSSSCG00000013360	TMEM86A	131.58	-1.10	0.17	-6.44	0.00	0.00
ENSSSCG00000001197	ZNF165	37.29	-1.10	0.32	-3.49	0.00	0.00
ENSSSCG00000028157	CASP8	1219.09	-1.10	0.07	-15.76	0.00	0.00
ENSSSCG00000012955	KLC2	1824.61	-1.10	0.06	-17.27	0.00	0.00
ENSSSCG00000015136	UBASH3B	249.66	-1.10	0.13	-8.67	0.00	0.00
ENSSSCG00000032796	GRINA	2079.05	-1.10	0.07	-16.24	0.00	0.00
ENSSSCG00000006214	RRS1	243.85	-1.10	0.13	-8.46	0.00	0.00
ENSSSCG00000007767	ZNF668	152.68	-1.10	0.16	-6.77	0.00	0.00
ENSSSCG00000001011	SERPINB1	3027.95	-1.10	0.05	-20.35	0.00	0.00
ENSSSCG00000036291	ZNF576	219.31	-1.10	0.13	-8.26	0.00	0.00
ENSSSCG00000003645	UTP11	1047.78	-1.10	0.08	-14.35	0.00	0.00
ENSSSCG00000030177	EBNA1BP2	1118.26	-1.10	0.07	-14.72	0.00	0.00
ENSSSCG00000025892	PRR3	242.41	-1.10	0.14	-8.00	0.00	0.00
ENSSSCG00000036941	AKR1E2	553.47	-1.10	0.10	-11.54	0.00	0.00
ENSSSCG00000001471	BRD2	4607.07	-1.10	0.05	-20.68	0.00	0.00
ENSSSCG00000017155	EIF4A3	2731.48	-1.10	0.06	-19.87	0.00	0.00

ENSSSCG00000032985	GOT2	11792.22	-1.10	0.05	-22.63	0.00	0.00
ENSSSCG00000006157	MSTO1	829.89	-1.10	0.08	-13.81	0.00	0.00
ENSSSCG00000003553	MTFR1L	916.36	-1.10	0.08	-14.61	0.00	0.00
ENSSSCG00000002496	GLRX5	376.46	-1.10	0.11	-10.32	0.00	0.00
ENSSSCG00000025005	B4GALT6	198.95	-1.09	0.14	-7.67	0.00	0.00
ENSSSCG00000040889	LAMTOR5	353.19	-1.09	0.11	-9.95	0.00	0.00
ENSSSCG00000014905	TMEM126B	673.09	-1.09	0.08	-13.35	0.00	0.00
ENSSSCG00000021821	MYO1C	4627.80	-1.09	0.05	-23.96	0.00	0.00
ENSSSCG00000001245	ZNRD1	124.58	-1.09	0.18	-6.18	0.00	0.00
ENSSSCG00000012841	PNPLA2	405.53	-1.09	0.10	-10.51	0.00	0.00
ENSSSCG00000030017	FOXRED1	422.06	-1.09	0.10	-10.79	0.00	0.00
ENSSSCG00000002905	RBM42	1344.58	-1.09	0.06	-17.03	0.00	0.00
ENSSSCG00000006337	HSD17B7	189.39	-1.09	0.14	-7.59	0.00	0.00
ENSSSCG00000038694	CCND2	1563.92	-1.09	0.07	-16.36	0.00	0.00
ENSSSCG00000015298	SRI	991.35	-1.09	0.07	-14.88	0.00	0.00
ENSSSCG00000024907	SDHAF1	139.70	-1.09	0.16	-6.62	0.00	0.00
ENSSSCG00000029326	CCNB1	3810.98	-1.09	0.06	-18.28	0.00	0.00
ENSSSCG00000027986	ABCB8	1149.00	-1.09	0.07	-15.02	0.00	0.00
ENSSSCG00000035775	BCS1L	492.78	-1.09	0.10	-11.01	0.00	0.00
ENSSSCG00000003106	AP2S1	1396.09	-1.09	0.07	-16.08	0.00	0.00
ENSSSCG00000006333	NUF2	1178.89	-1.09	0.07	-14.69	0.00	0.00
ENSSSCG00000037597		18.91	-1.09	0.45	-2.43	0.02	0.02
ENSSSCG00000033500		1413.39	-1.09	0.09	-12.34	0.00	0.00
ENSSSCG00000001509	DAXX	601.04	-1.09	0.09	-12.76	0.00	0.00
ENSSSCG00000003583	RPA2	1257.82	-1.09	0.07	-15.58	0.00	0.00

ENSSSCG00000012273	USP11	1173.65	-1.09	0.07	-15.50	0.00	0.00
ENSSSCG00000012879	MRPL21	685.19	-1.09	0.09	-12.20	0.00	0.00
ENSSSCG00000016076	COQ10B	840.24	-1.09	0.09	-12.17	0.00	0.00
ENSSSCG00000031821	HASPIN	1132.90	-1.09	0.07	-15.21	0.00	0.00
ENSSSCG00000005016	L2HGDH	16.88	-1.09	0.48	-2.25	0.02	0.03
ENSSSCG00000007493		2507.52	-1.09	0.06	-18.00	0.00	0.00
ENSSSCG00000029944	FASN	5865.37	-1.09	0.05	-19.92	0.00	0.00
ENSSSCG00000014152	MBLAC2	110.36	-1.09	0.18	-5.93	0.00	0.00
ENSSSCG00000015938	SSB	5482.41	-1.08	0.05	-23.26	0.00	0.00
ENSSSCG00000039619	PIN1	830.40	-1.08	0.08	-13.93	0.00	0.00
ENSSSCG00000020838		99.29	-1.08	0.19	-5.60	0.00	0.00
ENSSSCG00000006809	RBM15	1949.10	-1.08	0.06	-18.10	0.00	0.00
ENSSSCG00000024428	CHRNA9	68.14	-1.08	0.23	-4.65	0.00	0.00
ENSSSCG00000007962	SNRNP25	561.71	-1.08	0.09	-11.57	0.00	0.00
ENSSSCG00000005131	DMRTA1	120.64	-1.08	0.18	-6.13	0.00	0.00
ENSSSCG00000002807	USB1	198.71	-1.08	0.14	-7.67	0.00	0.00
ENSSSCG00000023354	ACAD9	967.78	-1.08	0.07	-15.18	0.00	0.00
ENSSSCG00000020837		4965.05	-1.08	0.05	-22.07	0.00	0.00
ENSSSCG00000010855		838.26	-1.08	0.08	-13.57	0.00	0.00
ENSSSCG00000006862	VCAM1	330.34	-1.08	0.12	-8.68	0.00	0.00
ENSSSCG00000038867	PPM1K	317.86	-1.08	0.12	-9.31	0.00	0.00
ENSSSCG00000032085	CACTIN	958.67	-1.08	0.07	-15.14	0.00	0.00
ENSSSCG00000031600	SYNGR2	2300.75	-1.08	0.07	-15.71	0.00	0.00
ENSSSCG00000003903		1566.46	-1.08	0.06	-18.16	0.00	0.00
ENSSSCG00000004702	STRC	73.77	-1.08	0.24	-4.58	0.00	0.00

ENSSSCG00000017505	MED1	5162.72	-1.08	0.05	-23.32	0.00	0.00
ENSSSCG00000027135	PPM1G	2343.17	-1.08	0.06	-18.00	0.00	0.00
ENSSSCG00000003491	AKR7A2	1064.48	-1.08	0.08	-13.78	0.00	0.00
ENSSSCG00000036676	MAIP1	464.88	-1.08	0.10	-11.07	0.00	0.00
ENSSSCG00000000007	TRMU	247.58	-1.08	0.13	-8.21	0.00	0.00
ENSSSCG00000017099	FASTKD3	1228.41	-1.08	0.08	-14.18	0.00	0.00
ENSSSCG00000026042	ULBP1	499.02	-1.08	0.09	-11.71	0.00	0.00
ENSSSCG00000034494	MARS2	210.49	-1.08	0.14	-7.64	0.00	0.00
ENSSSCG00000038739	MYPOP	41.62	-1.07	0.31	-3.44	0.00	0.00
ENSSSCG00000008000	RPUSD1	507.73	-1.07	0.10	-10.57	0.00	0.00
ENSSSCG00000008702	DOK7	340.36	-1.07	0.11	-9.69	0.00	0.00
ENSSSCG00000002254	NR2F2	228.91	-1.07	0.14	-7.58	0.00	0.00
ENSSSCG00000032340		284.46	-1.07	0.12	-8.97	0.00	0.00
ENSSSCG00000027984	ZWINT	314.46	-1.07	0.12	-8.73	0.00	0.00
ENSSSCG00000010972	DCTN3	597.07	-1.07	0.09	-12.45	0.00	0.00
ENSSSCG00000006093	NDUFAF6	402.83	-1.07	0.10	-10.32	0.00	0.00
ENSSSCG00000015819	PLPP5	265.95	-1.07	0.12	-8.60	0.00	0.00
ENSSSCG00000003844	TTC4	287.59	-1.07	0.12	-8.66	0.00	0.00
ENSSSCG00000016919	SETD9	170.12	-1.07	0.15	-7.10	0.00	0.00
ENSSSCG00000034406	MRPL58	809.92	-1.07	0.08	-13.85	0.00	0.00
ENSSSCG00000025770	ST6GAL1	49.40	-1.07	0.27	-3.96	0.00	0.00
ENSSSCG00000039202	FCF1	1182.92	-1.07	0.08	-14.04	0.00	0.00
ENSSSCG00000006947	SYDE2	345.51	-1.07	0.12	-9.06	0.00	0.00
ENSSSCG00000017187	FOXJ1	2767.22	-1.07	0.12	-8.65	0.00	0.00
ENSSSCG00000011447	SPCS1	1911.42	-1.07	0.06	-17.37	0.00	0.00

ENSSSCG00000011444	NT5DC2	1353.81	-1.07	0.07	-15.24	0.00	0.00
ENSSSCG00000013744	TRMT1	1226.58	-1.07	0.07	-15.68	0.00	0.00
ENSSSCG00000003419	MAD2L2	1083.13	-1.07	0.07	-15.28	0.00	0.00
ENSSSCG00000034927		8506.41	-1.07	0.05	-22.54	0.00	0.00
ENSSSCG00000013936	NDUFA13	929.72	-1.07	0.07	-14.61	0.00	0.00
ENSSSCG00000040743	M6PR	2453.09	-1.07	0.06	-19.16	0.00	0.00
ENSSSCG00000010078	TOP3B	876.53	-1.07	0.08	-14.11	0.00	0.00
ENSSSCG00000027894	FAM76A	988.84	-1.07	0.07	-14.81	0.00	0.00
ENSSSCG00000001564	SRSF3	1382.80	-1.07	0.07	-14.84	0.00	0.00
ENSSSCG00000006869	TRMT13	632.84	-1.07	0.09	-11.94	0.00	0.00
ENSSSCG00000031958	KCTD2	1747.50	-1.07	0.07	-16.35	0.00	0.00
ENSSSCG00000023829	CCP110	1503.34	-1.07	0.06	-17.26	0.00	0.00
ENSSSCG00000013468	THOP1	550.09	-1.07	0.09	-11.63	0.00	0.00
ENSSSCG00000034129		131.75	-1.06	0.18	-6.06	0.00	0.00
ENSSSCG00000000690	MLF2	5780.80	-1.06	0.05	-21.05	0.00	0.00
ENSSSCG00000033518	LLGL2	1437.36	-1.06	0.06	-16.76	0.00	0.00
ENSSSCG00000040578	CASKIN2	797.39	-1.06	0.08	-13.59	0.00	0.00
ENSSSCG00000026962	A4GALT	455.83	-1.06	0.11	-9.26	0.00	0.00
ENSSSCG00000010312	PLAU	6895.61	-1.06	0.06	-18.26	0.00	0.00
ENSSSCG00000007487	PFDN4	1387.40	-1.06	0.07	-15.51	0.00	0.00
ENSSSCG00000015222	PUS3	414.93	-1.06	0.10	-10.41	0.00	0.00
ENSSSCG00000017134	FN3KRP	460.55	-1.06	0.12	-9.14	0.00	0.00
ENSSSCG00000014250		183.57	-1.06	0.15	-7.29	0.00	0.00
ENSSSCG00000011783	EIF2B5	1700.45	-1.06	0.06	-16.54	0.00	0.00
ENSSSCG00000005318	CREB3	2575.49	-1.06	0.06	-18.69	0.00	0.00

ENSSSCG00000040782		530.25	-1.06	0.11	-9.88	0.00	0.00
ENSSSCG00000008480	MORN2	416.87	-1.06	0.10	-10.54	0.00	0.00
ENSSSCG00000026177	SLC25A40	1718.64	-1.06	0.06	-17.48	0.00	0.00
ENSSSCG00000026700	RBM23	879.78	-1.06	0.08	-13.63	0.00	0.00
ENSSSCG00000017953	FXR2	1709.29	-1.06	0.06	-17.53	0.00	0.00
ENSSSCG00000031518	STARD4	324.83	-1.06	0.13	-8.31	0.00	0.00
ENSSSCG00000035068	GJB4	130.01	-1.06	0.17	-6.17	0.00	0.00
ENSSSCG00000002998	SNRPA	1192.07	-1.06	0.07	-14.66	0.00	0.00
ENSSSCG00000023156		686.84	-1.06	0.08	-12.87	0.00	0.00
ENSSSCG00000003017	TGFB1	1924.58	-1.06	0.06	-18.57	0.00	0.00
ENSSSCG00000004629	LYSMD2	156.01	-1.06	0.16	-6.43	0.00	0.00
ENSSSCG00000017415	TTC25	246.86	-1.06	0.13	-8.15	0.00	0.00
ENSSSCG00000002550		1251.73	-1.06	0.07	-15.16	0.00	0.00
ENSSSCG00000005610	SLC2A8	194.18	-1.06	0.15	-7.21	0.00	0.00
ENSSSCG00000038904		43.52	-1.06	0.30	-3.56	0.00	0.00
ENSSSCG00000008400	CFAP36	543.07	-1.06	0.09	-12.01	0.00	0.00
ENSSSCG00000040801		387.70	-1.06	0.12	-8.69	0.00	0.00
ENSSSCG00000020705	MAP3K8	249.45	-1.06	0.13	-7.97	0.00	0.00
ENSSSCG00000006556	TPM3	12070.73	-1.06	0.05	-22.47	0.00	0.00
ENSSSCG00000009881	OAS2	1169.45	-1.05	0.07	-15.73	0.00	0.00
ENSSSCG00000014264	HINT1	2605.21	-1.05	0.07	-16.16	0.00	0.00
ENSSSCG00000023669	RF00088	23.62	-1.05	0.39	-2.70	0.01	0.01
ENSSSCG00000001638	MRPS10	1437.94	-1.05	0.06	-16.27	0.00	0.00
ENSSSCG00000006632	SCNM1	842.01	-1.05	0.08	-13.88	0.00	0.00
ENSSSCG00000027779	TMEM259	2999.94	-1.05	0.06	-18.51	0.00	0.00

ENSSSCG00000005995	MTBP	345.56	-1.05	0.11	-9.59	0.00	0.00
ENSSSCG00000038383	DYNC2LI1	707.03	-1.05	0.08	-12.42	0.00	0.00
ENSSSCG00000017106	MED10	1517.06	-1.05	0.07	-15.17	0.00	0.00
ENSSSCG00000040385	RFNG	1253.23	-1.05	0.07	-15.35	0.00	0.00
ENSSSCG00000039265	COX6A1	3701.85	-1.05	0.06	-18.92	0.00	0.00
ENSSSCG00000012952	TMEM151A	36.50	-1.05	0.32	-3.28	0.00	0.00
ENSSSCG00000010330	PPIF	1268.55	-1.05	0.07	-14.65	0.00	0.00
ENSSSCG00000008237	RETSAT	2664.05	-1.05	0.05	-19.77	0.00	0.00
ENSSSCG00000015999	FKBP7	59.06	-1.05	0.25	-4.12	0.00	0.00
ENSSSCG00000030269		1442.04	-1.05	0.07	-16.15	0.00	0.00
ENSSSCG00000033149	DDX47	904.46	-1.05	0.08	-13.88	0.00	0.00
ENSSSCG00000021410	STRAP	1944.29	-1.05	0.06	-16.86	0.00	0.00
ENSSSCG00000017745	UTP6	3079.81	-1.05	0.05	-20.68	0.00	0.00
ENSSSCG00000039873	DCXR	686.04	-1.05	0.08	-12.77	0.00	0.00
ENSSSCG00000018057	SOCS7	1205.13	-1.05	0.07	-15.55	0.00	0.00
ENSSSCG00000000635		425.35	-1.05	0.10	-10.33	0.00	0.00
ENSSSCG00000017912	PLD2	2138.38	-1.05	0.05	-19.33	0.00	0.00
ENSSSCG00000038657		1204.98	-1.05	0.07	-14.97	0.00	0.00
ENSSSCG00000000706	LTBR	1751.25	-1.05	0.07	-15.85	0.00	0.00
ENSSSCG00000001838	POLG	1997.06	-1.05	0.06	-17.07	0.00	0.00
ENSSSCG00000025326	TMEM243	1181.72	-1.05	0.07	-15.74	0.00	0.00
ENSSSCG00000032464	PURB	304.56	-1.05	0.12	-8.88	0.00	0.00
ENSSSCG00000010574	LDB1	1560.47	-1.05	0.07	-15.43	0.00	0.00
ENSSSCG00000011601	LSM3	1096.11	-1.05	0.09	-11.55	0.00	0.00
ENSSSCG00000037630	RF00045	45.91	-1.05	0.29	-3.62	0.00	0.00

ENSSSCG00000022689	GADD45B	631.48	-1.05	0.10	-10.81	0.00	0.00
ENSSSCG00000007919		497.03	-1.05	0.10	-10.86	0.00	0.00
ENSSSCG00000036047	THG1L	1001.05	-1.05	0.08	-13.87	0.00	0.00
ENSSSCG00000010990	NOL6	788.76	-1.04	0.09	-11.37	0.00	0.00
ENSSSCG00000012056	CHAF1B	555.68	-1.04	0.09	-11.78	0.00	0.00
ENSSSCG00000014060		1967.26	-1.04	0.06	-18.49	0.00	0.00
ENSSSCG00000023873	AZIN1	1411.40	-1.04	0.08	-13.36	0.00	0.00
ENSSSCG00000025881	PDHB	3987.85	-1.04	0.06	-18.46	0.00	0.00
ENSSSCG00000039196	POLR2I	415.90	-1.04	0.12	-8.94	0.00	0.00
ENSSSCG00000032234	TMEM165	945.29	-1.04	0.07	-13.99	0.00	0.00
ENSSSCG00000018084	ND3	466.69	-1.04	0.22	-4.73	0.00	0.00
ENSSSCG00000015120	USP2	407.95	-1.04	0.10	-9.97	0.00	0.00
ENSSSCG00000031788	PTAFR	146.29	-1.04	0.17	-6.05	0.00	0.00
ENSSSCG00000001070	FAM8A1	449.06	-1.04	0.11	-9.89	0.00	0.00
ENSSSCG00000010179	ARV1	357.54	-1.04	0.11	-9.59	0.00	0.00
ENSSSCG00000036988		587.50	-1.04	0.09	-11.11	0.00	0.00
ENSSSCG00000012518	ARMCX5	234.98	-1.04	0.13	-7.96	0.00	0.00
ENSSSCG00000039494	NDUFC2	2631.54	-1.04	0.06	-16.43	0.00	0.00
ENSSSCG00000013911		1910.58	-1.04	0.06	-17.82	0.00	0.00
ENSSSCG00000032098		15.95	-1.04	0.48	-2.16	0.03	0.04
ENSSSCG00000003981	ZFP69B	664.37	-1.04	0.08	-12.60	0.00	0.00
ENSSSCG00000020870		2076.36	-1.04	0.06	-18.44	0.00	0.00
ENSSSCG00000031793	ZNF331	121.37	-1.04	0.18	-5.81	0.00	0.00
ENSSSCG00000010071	MMP11	1228.71	-1.04	0.07	-14.64	0.00	0.00
ENSSSCG00000009788		1776.72	-1.04	0.06	-16.44	0.00	0.00

ENSSSCG00000003216	POLD1	1634.27	-1.04	0.06	-16.27	0.00	0.00
ENSSSCG00000038682	TAF1D	2487.11	-1.04	0.05	-19.06	0.00	0.00
ENSSSCG00000011411	TMEM115	935.80	-1.04	0.08	-12.53	0.00	0.00
ENSSSCG00000006732	TRIM45	410.03	-1.04	0.10	-10.35	0.00	0.00
ENSSSCG00000039021	LSM2	259.83	-1.04	0.13	-8.20	0.00	0.00
ENSSSCG00000032806	NUDCD2	857.42	-1.04	0.08	-13.76	0.00	0.00
ENSSSCG00000015337		1133.17	-1.04	0.07	-15.17	0.00	0.00
ENSSSCG00000032146		910.61	-1.04	0.07	-14.19	0.00	0.00
ENSSSCG00000002639		800.60	-1.04	0.09	-11.68	0.00	0.00
ENSSSCG00000033490		19.20	-1.03	0.45	-2.31	0.02	0.03
ENSSSCG00000037197	BAG5	684.61	-1.03	0.08	-12.52	0.00	0.00
ENSSSCG00000011567		494.77	-1.03	0.11	-9.45	0.00	0.00
ENSSSCG00000040984	PPIL1	422.95	-1.03	0.10	-10.21	0.00	0.00
ENSSSCG00000013722	WDR83	159.75	-1.03	0.16	-6.47	0.00	0.00
ENSSSCG00000025564	GAS2L1	779.09	-1.03	0.08	-12.34	0.00	0.00
ENSSSCG00000025092	CDK4	1819.47	-1.03	0.06	-17.28	0.00	0.00
ENSSSCG00000008348	PLEK	157.24	-1.03	0.16	-6.33	0.00	0.00
ENSSSCG00000011548	BRPF1	919.75	-1.03	0.08	-12.87	0.00	0.00
ENSSSCG00000035034	CNIH4	548.88	-1.03	0.10	-10.23	0.00	0.00
ENSSSCG00000028227	RPN2	13492.00	-1.03	0.05	-22.54	0.00	0.00
ENSSSCG00000024776		1512.06	-1.03	0.06	-16.69	0.00	0.00
ENSSSCG00000034058	ARHGDIA	5763.31	-1.03	0.06	-17.16	0.00	0.00
ENSSSCG00000008305	SMYD5	659.51	-1.03	0.09	-11.20	0.00	0.00
ENSSSCG00000008750	PACRGL	194.59	-1.03	0.15	-7.07	0.00	0.00
ENSSSCG00000033295	EIF2S1	2602.43	-1.03	0.05	-19.48	0.00	0.00

ENSSSCG00000003035		2224.99	-1.03	0.06	-16.20	0.00	0.00
ENSSSCG00000002793		83.61	-1.03	0.21	-4.87	0.00	0.00
ENSSSCG00000035908		531.99	-1.03	0.10	-10.78	0.00	0.00
ENSSSCG00000000394	CNPY2	532.75	-1.03	0.09	-11.39	0.00	0.00
ENSSSCG00000007563	BRAT1	340.98	-1.03	0.11	-9.29	0.00	0.00
ENSSSCG00000015973	ATP5MC3	2134.93	-1.03	0.06	-15.89	0.00	0.00
ENSSSCG00000013596		1274.83	-1.03	0.07	-14.58	0.00	0.00
ENSSSCG00000033148	MRPL12	1286.85	-1.03	0.06	-16.14	0.00	0.00
ENSSSCG00000034997		15.82	-1.03	0.49	-2.11	0.03	0.05
ENSSSCG00000038644		21.04	-1.03	0.41	-2.48	0.01	0.02
ENSSSCG00000016101	CFLAR	731.86	-1.03	0.08	-12.57	0.00	0.00
ENSSSCG00000039663	TWSG1	856.26	-1.03	0.09	-11.85	0.00	0.00
ENSSSCG00000018069	ND2	5947.61	-1.02	0.22	-4.75	0.00	0.00
ENSSSCG00000037531	MAP3K11	917.16	-1.02	0.08	-13.18	0.00	0.00
ENSSSCG00000031874	REM2	164.94	-1.02	0.15	-6.63	0.00	0.00
ENSSSCG00000015616	HSD11B1	98.67	-1.02	0.20	-5.15	0.00	0.00
ENSSSCG00000037343	MRPS11	802.97	-1.02	0.08	-13.13	0.00	0.00
ENSSSCG00000039582		2168.78	-1.02	0.06	-16.74	0.00	0.00
ENSSSCG00000004894	SERPINB5	3835.05	-1.02	0.06	-16.17	0.00	0.00
ENSSSCG00000005082	PCNX4	754.73	-1.02	0.08	-12.76	0.00	0.00
ENSSSCG00000035042	RF01883	34.96	-1.02	0.32	-3.16	0.00	0.00
ENSSSCG00000008294	ACTG2	50.19	-1.02	0.28	-3.68	0.00	0.00
ENSSSCG00000032469		2054.38	-1.02	0.06	-17.23	0.00	0.00
ENSSSCG00000003643	SF3A3	2108.19	-1.02	0.07	-14.27	0.00	0.00
ENSSSCG00000009259	PAQR3	508.69	-1.02	0.09	-11.23	0.00	0.00

ENSSSCG00000037459	TCTA	307.52	-1.02	0.12	-8.72	0.00	0.00
ENSSSCG00000011304	ZNF197	116.41	-1.02	0.18	-5.57	0.00	0.00
ENSSSCG00000000370	DGKA	1759.30	-1.02	0.06	-17.43	0.00	0.00
ENSSSCG00000004986	TRAPPC6B	484.45	-1.02	0.10	-9.87	0.00	0.00
ENSSSCG00000010635	ZDHHC6	1473.22	-1.02	0.06	-16.19	0.00	0.00
ENSSSCG00000037920	TRIM28	5362.68	-1.02	0.06	-18.14	0.00	0.00
ENSSSCG00000023331		1665.14	-1.02	0.07	-15.34	0.00	0.00
ENSSSCG00000032909	CDCA8	1214.39	-1.02	0.07	-14.42	0.00	0.00
ENSSSCG00000036042		15.32	-1.02	0.49	-2.07	0.04	0.05
ENSSSCG00000015525	TOR3A	480.42	-1.02	0.10	-9.80	0.00	0.00
ENSSSCG00000030660	PDIK1L	368.68	-1.02	0.11	-9.24	0.00	0.00
ENSSSCG00000035677	EPOP	48.24	-1.02	0.29	-3.49	0.00	0.00
ENSSSCG00000009534		522.56	-1.02	0.10	-10.52	0.00	0.00
ENSSSCG00000003262	TFPT	200.32	-1.02	0.14	-7.33	0.00	0.00
ENSSSCG00000010259	TYSND1	309.71	-1.02	0.12	-8.45	0.00	0.00
ENSSSCG00000002719	EXOSC6	77.81	-1.02	0.23	-4.52	0.00	0.00
ENSSSCG00000025806	FAM118B	809.43	-1.02	0.08	-12.93	0.00	0.00
ENSSSCG00000035132	IDNK	349.30	-1.02	0.11	-9.50	0.00	0.00
ENSSSCG00000014832	DNAJB13	183.71	-1.02	0.15	-6.80	0.00	0.00
ENSSSCG00000007452	TP53RK	351.99	-1.02	0.11	-9.02	0.00	0.00
ENSSSCG00000034353		126.86	-1.02	0.18	-5.53	0.00	0.00
ENSSSCG00000037900	CCND1	1580.58	-1.02	0.07	-15.35	0.00	0.00
ENSSSCG00000012853	IRF7	942.96	-1.02	0.08	-12.78	0.00	0.00
ENSSSCG00000003589	SRSF4	2471.28	-1.02	0.06	-17.53	0.00	0.00
ENSSSCG00000025855	RNMT	918.14	-1.02	0.08	-13.33	0.00	0.00

ENSSSCG00000009912	POP5	241.49	-1.01	0.14	-7.25	0.00	0.00
ENSSSCG00000004711	LCMT2	150.28	-1.01	0.16	-6.39	0.00	0.00
ENSSSCG00000017198	ACOX1	6198.57	-1.01	0.05	-19.34	0.00	0.00
ENSSSCG00000031847	RBM28	941.22	-1.01	0.08	-13.50	0.00	0.00
ENSSSCG00000017955	MPDU1	723.59	-1.01	0.09	-11.13	0.00	0.00
ENSSSCG00000021145	AP1S1	2251.65	-1.01	0.06	-16.81	0.00	0.00
ENSSSCG00000013270	CRY2	743.34	-1.01	0.08	-13.10	0.00	0.00
ENSSSCG00000014284		25.15	-1.01	0.38	-2.68	0.01	0.01
ENSSSCG00000032154	ERF	743.40	-1.01	0.09	-11.68	0.00	0.00
ENSSSCG00000014371		563.42	-1.01	0.10	-10.17	0.00	0.00
ENSSSCG00000012965	DRAP1	1837.12	-1.01	0.06	-17.70	0.00	0.00
ENSSSCG00000023315	THOC6	496.40	-1.01	0.09	-10.82	0.00	0.00
ENSSSCG00000012961		6170.68	-1.01	0.05	-21.86	0.00	0.00
ENSSSCG00000018082	COX3	17192.89	-1.01	0.16	-6.31	0.00	0.00
ENSSSCG00000016707	HOXA1	33.50	-1.01	0.33	-3.07	0.00	0.00
ENSSSCG00000016041		647.25	-1.01	0.08	-11.99	0.00	0.00
ENSSSCG00000004192	CCN2	1044.79	-1.01	0.09	-11.53	0.00	0.00
ENSSSCG00000034265	DESI1	830.71	-1.01	0.08	-12.69	0.00	0.00
ENSSSCG00000036294	NUDT19	296.59	-1.01	0.11	-8.81	0.00	0.00
ENSSSCG00000004220	TRMT11	301.70	-1.01	0.13	-7.86	0.00	0.00
ENSSSCG00000008030	TSR3	549.56	-1.01	0.09	-10.75	0.00	0.00
ENSSSCG00000004517	CXXC1	1173.66	-1.01	0.08	-12.89	0.00	0.00
ENSSSCG00000025854	MMGT1	2008.17	-1.01	0.07	-14.93	0.00	0.00
ENSSSCG00000030277	ENDOV	129.95	-1.01	0.17	-5.87	0.00	0.00
ENSSSCG00000036755	TENT5B	334.06	-1.01	0.11	-8.81	0.00	0.00

ENSSSCG00000012957	SF3B2	6073.69	-1.01	0.05	-21.52	0.00	0.00
ENSSSCG00000009766	ATP6V0A2	1341.98	-1.01	0.07	-15.24	0.00	0.00
ENSSSCG00000014558	SIRT3	622.61	-1.01	0.09	-11.12	0.00	0.00
ENSSSCG00000016094	BZW1	7336.68	-1.01	0.04	-23.54	0.00	0.00
ENSSSCG00000030585	HOXC6	38.14	-1.01	0.33	-3.04	0.00	0.00
ENSSSCG00000011793	LIPH	76.62	-1.01	0.24	-4.28	0.00	0.00
ENSSSCG00000038067		192.63	-1.01	0.16	-6.46	0.00	0.00
ENSSSCG00000037779	CAPN10	269.92	-1.01	0.12	-8.36	0.00	0.00
ENSSSCG00000004575		23.78	-1.01	0.41	-2.47	0.01	0.02
ENSSSCG00000013045	OTUB1	2500.12	-1.01	0.06	-16.59	0.00	0.00
ENSSSCG00000012053	CBR3	45.97	-1.01	0.28	-3.54	0.00	0.00
ENSSSCG00000016203	CFAP65	39.69	-1.01	0.30	-3.34	0.00	0.00
ENSSSCG00000027443	MRAS	232.45	-1.01	0.13	-7.49	0.00	0.00
ENSSSCG00000035221	ENTR1	884.54	-1.01	0.08	-13.05	0.00	0.00
ENSSSCG00000001475	SLC39A7	1087.53	-1.01	0.07	-14.64	0.00	0.00
ENSSSCG00000008008	STUB1	1273.41	-1.00	0.07	-14.04	0.00	0.00
ENSSSCG00000040154	MRPL36	310.48	-1.00	0.12	-8.26	0.00	0.00
ENSSSCG00000038662	ZBED8	172.57	-1.00	0.16	-6.40	0.00	0.00
ENSSSCG00000007308	ROMO1	894.39	-1.00	0.07	-13.42	0.00	0.00
ENSSSCG00000032979	TMEM267	157.19	-1.00	0.15	-6.48	0.00	0.00
ENSSSCG00000024748	THUMPD1	1646.50	-1.00	0.07	-15.24	0.00	0.00
ENSSSCG00000002622	TMEM14A	916.74	-1.00	0.07	-13.76	0.00	0.00
ENSSSCG00000031011	CCDC160	243.21	-1.00	0.13	-7.90	0.00	0.00
ENSSSCG00000030426	C9orf78	995.38	-1.00	0.08	-13.23	0.00	0.00
ENSSSCG00000023231	RBM22	1552.67	-1.00	0.07	-15.33	0.00	0.00

ENSSSCG00000033027	CIAO2B	560.49	-1.00	0.09	-10.91	0.00	0.00
ENSSSCG00000007687	LRWD1	596.09	-1.00	0.08	-11.82	0.00	0.00
ENSSSCG00000014048	HK3	38.18	-1.00	0.31	-3.22	0.00	0.00
ENSSSCG00000034782	VPS25	2107.76	-1.00	0.06	-17.71	0.00	0.00
ENSSSCG00000036328	DRG2	1743.02	-1.00	0.06	-15.99	0.00	0.00
ENSSSCG00000004781	PAK6	308.41	1.00	0.11	8.71	0.00	0.00
ENSSSCG00000001986	NFATC4	90.21	1.00	0.21	4.88	0.00	0.00
ENSSSCG00000017895	RABEP1	2174.85	1.00	0.06	17.62	0.00	0.00
ENSSSCG00000030337	NYAP1	125.43	1.00	0.18	5.71	0.00	0.00
ENSSSCG00000017956	CD68	131.60	1.00	0.18	5.50	0.00	0.00
ENSSSCG00000011215	OXSM	431.15	1.00	0.11	9.55	0.00	0.00
ENSSSCG00000005654	SPTAN1	8781.30	1.00	0.04	23.05	0.00	0.00
ENSSSCG00000034730	PIAS2	912.86	1.01	0.08	13.20	0.00	0.00
ENSSSCG00000027404	UNC93A	55.95	1.01	0.27	3.75	0.00	0.00
ENSSSCG00000016406	ESYT2	970.74	1.01	0.07	13.89	0.00	0.00
ENSSSCG00000035331		166.64	1.01	0.16	6.21	0.00	0.00
ENSSSCG00000013630	DNM2	1892.74	1.01	0.06	17.07	0.00	0.00
ENSSSCG00000010926	SYT2	208.81	1.01	0.14	7.37	0.00	0.00
ENSSSCG00000017094	MARCH6	2840.84	1.01	0.05	18.70	0.00	0.00
ENSSSCG00000036887	CLPB	247.00	1.01	0.13	7.66	0.00	0.00
ENSSSCG00000021971	DPEP1	36.95	1.01	0.32	3.11	0.00	0.00
ENSSSCG00000002652	KLHDC4	554.19	1.01	0.09	11.06	0.00	0.00
ENSSSCG00000031912		2291.34	1.01	0.06	16.59	0.00	0.00
ENSSSCG00000034119	MEIG1	66.73	1.01	0.23	4.30	0.00	0.00
ENSSSCG00000008026	CRAMP1	491.88	1.01	0.10	10.35	0.00	0.00

ENSSSCG00000000862	GNPTAB	1100.44	1.01	0.07	13.64	0.00	0.00
ENSSSCG00000017585	SAMD14	46.71	1.01	0.29	3.50	0.00	0.00
ENSSSCG00000007695		61.87	1.01	0.25	4.08	0.00	0.00
ENSSSCG00000000037	ARFGAP3	1881.37	1.01	0.06	16.86	0.00	0.00
ENSSSCG00000005056	DLGAP5	1811.44	1.01	0.06	15.74	0.00	0.00
ENSSSCG00000033528	BPTF	1547.12	1.01	0.06	16.34	0.00	0.00
ENSSSCG00000000393	PAN2	898.08	1.01	0.07	13.83	0.00	0.00
ENSSSCG00000025417	BBS2	729.24	1.01	0.08	12.89	0.00	0.00
ENSSSCG00000008722		261.55	1.01	0.12	8.20	0.00	0.00
ENSSSCG00000009023	DCLK2	25.23	1.01	0.38	2.65	0.01	0.01
ENSSSCG00000010702	PLEKHA1	1653.73	1.01	0.06	16.34	0.00	0.00
ENSSSCG00000011443	STAB1	80.13	1.01	0.22	4.67	0.00	0.00
ENSSSCG00000012163	RPS6KA3	625.30	1.01	0.09	11.33	0.00	0.00
ENSSSCG00000030484	AHR	529.09	1.01	0.09	11.33	0.00	0.00
ENSSSCG00000017102	TENT4A	977.68	1.01	0.08	13.22	0.00	0.00
ENSSSCG00000008961	MTHFD2L	139.38	1.01	0.17	6.08	0.00	0.00
ENSSSCG00000031579	PCP4L1	87.47	1.01	0.21	4.81	0.00	0.00
ENSSSCG00000022492	AMPD3	2435.74	1.02	0.06	18.37	0.00	0.00
ENSSSCG00000027855	SOCS1	104.32	1.02	0.19	5.24	0.00	0.00
ENSSSCG00000007171	EBF4	249.32	1.02	0.13	7.82	0.00	0.00
ENSSSCG00000009629	BIN3	213.02	1.02	0.14	7.45	0.00	0.00
ENSSSCG00000006888	SLC44A3	238.04	1.02	0.14	7.50	0.00	0.00
ENSSSCG00000033937		286.72	1.02	0.13	7.57	0.00	0.00
ENSSSCG00000023662	CHST3	254.12	1.02	0.13	8.05	0.00	0.00
ENSSSCG00000025729	IRS1	246.53	1.02	0.13	7.79	0.00	0.00

ENSSSCG00000001561	ETV7	32.56	1.02	0.34	3.01	0.00	0.00
ENSSSCG00000025836	SULT1C4	181.04	1.02	0.15	6.99	0.00	0.00
ENSSSCG00000010997	SPINK4	17.67	1.02	0.47	2.15	0.03	0.04
ENSSSCG00000002287	MPP5	959.59	1.02	0.08	12.53	0.00	0.00
ENSSSCG00000012131	MOSPD2	789.61	1.02	0.08	12.87	0.00	0.00
ENSSSCG00000004357	SIM1	157.41	1.02	0.16	6.34	0.00	0.00
ENSSSCG00000035262	TMEM74B	95.24	1.02	0.20	5.10	0.00	0.00
ENSSSCG00000025114	FMNL3	927.67	1.02	0.07	14.01	0.00	0.00
ENSSSCG00000010738	ZRANB1	518.83	1.02	0.09	10.91	0.00	0.00
ENSSSCG00000040373	BCL2L13	695.45	1.02	0.08	12.84	0.00	0.00
ENSSSCG00000031705		2135.99	1.02	0.05	18.77	0.00	0.00
ENSSSCG00000026819	NID1	130.92	1.02	0.18	5.70	0.00	0.00
ENSSSCG00000014581	TUB	60.47	1.02	0.25	4.13	0.00	0.00
ENSSSCG00000036613	POLQ	538.75	1.02	0.10	10.12	0.00	0.00
ENSSSCG00000026488		788.52	1.02	0.08	12.40	0.00	0.00
ENSSSCG00000014580	RIC3	19.28	1.02	0.43	2.35	0.02	0.03
ENSSSCG00000034373	TNRC6C	187.15	1.02	0.16	6.42	0.00	0.00
ENSSSCG00000022508	UIMC1	102.41	1.02	0.19	5.35	0.00	0.00
ENSSSCG00000003256	PRKCG	284.62	1.02	0.12	8.48	0.00	0.00
ENSSSCG00000035501	SMARCAL1	342.27	1.02	0.11	9.34	0.00	0.00
ENSSSCG00000016491	KIAA1147	70.48	1.02	0.24	4.27	0.00	0.00
ENSSSCG00000012063	VPS26C	624.02	1.03	0.09	11.82	0.00	0.00
ENSSSCG00000002013	DHRS4	18.80	1.03	0.44	2.31	0.02	0.03
ENSSSCG00000035196	BTBD8	215.46	1.03	0.13	7.61	0.00	0.00
ENSSSCG00000022975	AMT	46.98	1.03	0.28	3.63	0.00	0.00

ENSSSCG00000000288	HNRNPA1	4886.26	1.03	0.05	21.69	0.00	0.00
ENSSSCG00000014943	DEUP1	706.77	1.03	0.09	11.61	0.00	0.00
ENSSSCG00000027053	PACSIN1	60.36	1.03	0.25	4.07	0.00	0.00
ENSSSCG00000000843	TXNRD1	1966.35	1.03	0.06	16.79	0.00	0.00
ENSSSCG00000022661	FCHO2	405.66	1.03	0.10	10.05	0.00	0.00
ENSSSCG00000022506		4298.68	1.03	0.05	22.52	0.00	0.00
ENSSSCG00000028814	SOD3	3016.14	1.03	0.06	17.71	0.00	0.00
ENSSSCG00000015782	IRF2	362.27	1.03	0.11	9.61	0.00	0.00
ENSSSCG00000003941	SZT2	1105.44	1.03	0.07	14.83	0.00	0.00
ENSSSCG00000012452	SH3BGRL	465.24	1.03	0.10	10.43	0.00	0.00
ENSSSCG00000007815	GTF3C1	1740.00	1.03	0.06	17.63	0.00	0.00
ENSSSCG00000011843	PAK2	4021.53	1.03	0.05	19.62	0.00	0.00
ENSSSCG00000006645	SETDB1	1243.54	1.03	0.07	14.88	0.00	0.00
ENSSSCG00000033602	UBE2K	937.04	1.03	0.08	13.31	0.00	0.00
ENSSSCG00000007836	SCNN1G	19.02	1.03	0.46	2.24	0.03	0.04
ENSSSCG00000001538	DEF6	49.20	1.04	0.28	3.74	0.00	0.00
ENSSSCG00000003021	MEGF8	918.98	1.04	0.07	14.23	0.00	0.00
ENSSSCG00000015535	QSOX1	2231.81	1.04	0.06	18.23	0.00	0.00
ENSSSCG00000015119	MFRP	44.15	1.04	0.30	3.49	0.00	0.00
ENSSSCG00000004620	MYO5A	4663.16	1.04	0.06	18.11	0.00	0.00
ENSSSCG00000040920	PRKACB	986.89	1.04	0.07	13.91	0.00	0.00
ENSSSCG00000024336	TMPRSS2	177.66	1.04	0.15	7.05	0.00	0.00
ENSSSCG00000005894	ARHGAP39	216.01	1.04	0.14	7.62	0.00	0.00
ENSSSCG00000007337	CTNNBL1	394.40	1.04	0.11	9.59	0.00	0.00
ENSSSCG00000014568	ST5	447.54	1.04	0.10	10.50	0.00	0.00

ENSSSCG00000010341	TSPAN14	592.68	1.04	0.09	11.84	0.00	0.00
ENSSSCG00000014927	NOX4	185.93	1.04	0.15	7.05	0.00	0.00
ENSSSCG00000009071	JADE1	932.75	1.04	0.08	13.24	0.00	0.00
ENSSSCG00000031942	YES1	3003.36	1.04	0.06	16.25	0.00	0.00
ENSSSCG00000029125	FAM13B	324.22	1.04	0.11	9.18	0.00	0.00
ENSSSCG00000029837	VWA8	467.58	1.04	0.10	10.69	0.00	0.00
ENSSSCG00000008499	STRN	1473.04	1.04	0.06	16.91	0.00	0.00
ENSSSCG00000026331	COQ8B	255.40	1.04	0.13	8.05	0.00	0.00
ENSSSCG00000004814	LRRC28	626.79	1.04	0.09	11.59	0.00	0.00
ENSSSCG00000009084	SPATA5	82.72	1.04	0.21	4.94	0.00	0.00
ENSSSCG00000027763	NDUFAF2	74.56	1.04	0.22	4.64	0.00	0.00
ENSSSCG00000014540		66522.93	1.04	0.04	25.66	0.00	0.00
ENSSSCG00000014030	PHYKPL	63.37	1.04	0.24	4.31	0.00	0.00
ENSSSCG00000024062		69.47	1.04	0.23	4.47	0.00	0.00
ENSSSCG00000021053	GIPR	84.34	1.04	0.22	4.85	0.00	0.00
ENSSSCG00000002847	GPT2	1268.48	1.04	0.07	14.05	0.00	0.00
ENSSSCG00000016677	GARS	5787.23	1.05	0.05	22.13	0.00	0.00
ENSSSCG00000009836	ACAD10	816.08	1.05	0.08	13.24	0.00	0.00
ENSSSCG00000037487		24.78	1.05	0.38	2.74	0.01	0.01
ENSSSCG00000031429	YAP1	1205.00	1.05	0.08	13.09	0.00	0.00
ENSSSCG00000024127	ABI1	1476.64	1.05	0.06	17.22	0.00	0.00
ENSSSCG00000025174	WDFY1	1317.04	1.05	0.07	15.24	0.00	0.00
ENSSSCG00000006531		160.02	1.05	0.16	6.74	0.00	0.00
ENSSSCG00000006033	EIF3E	4766.30	1.05	0.05	21.34	0.00	0.00
ENSSSCG00000008600	PUM2	1756.42	1.05	0.06	16.20	0.00	0.00

ENSSSCG00000022197	HS6ST1	1308.68	1.05	0.07	16.16	0.00	0.00
ENSSSCG00000017000	RANBP17	326.39	1.05	0.11	9.18	0.00	0.00
ENSSSCG00000038928		322.72	1.05	0.11	9.19	0.00	0.00
ENSSSCG00000014173	LNPEP	834.10	1.05	0.08	13.41	0.00	0.00
ENSSSCG00000009505	MBNL2	510.67	1.05	0.09	11.20	0.00	0.00
ENSSSCG00000013905	FKBP8	7317.89	1.05	0.04	23.73	0.00	0.00
ENSSSCG00000012329	PHF8	443.59	1.05	0.10	10.93	0.00	0.00
ENSSSCG00000007816	IL21R	55.25	1.05	0.26	3.99	0.00	0.00
ENSSSCG00000030535	SNX9	1260.04	1.05	0.07	15.22	0.00	0.00
ENSSSCG00000028855	GMPS	1673.53	1.05	0.06	17.18	0.00	0.00
ENSSSCG00000026006	KLF13	138.22	1.05	0.18	5.90	0.00	0.00
ENSSSCG00000004454	ME1	1240.59	1.06	0.07	15.63	0.00	0.00
ENSSSCG00000000003	TTC38	408.72	1.06	0.10	10.48	0.00	0.00
ENSSSCG00000000799	IRAK4	483.34	1.06	0.10	11.02	0.00	0.00
ENSSSCG00000016794	MYO10	2346.52	1.06	0.06	18.86	0.00	0.00
ENSSSCG00000006302	GPR161	160.02	1.06	0.16	6.68	0.00	0.00
ENSSSCG00000033521		31.05	1.06	0.34	3.10	0.00	0.00
ENSSSCG00000012146	CTPS2	311.42	1.06	0.12	9.18	0.00	0.00
ENSSSCG00000013312	HIPK3	2575.59	1.06	0.06	18.95	0.00	0.00
ENSSSCG00000002298	ZFYVE26	831.46	1.06	0.08	13.11	0.00	0.00
ENSSSCG00000008762	STIM2	88.93	1.06	0.22	4.75	0.00	0.00
ENSSSCG00000009308	LNX2	213.40	1.06	0.14	7.63	0.00	0.00
ENSSSCG00000011619	EEFSEC	56.30	1.06	0.26	4.06	0.00	0.00
ENSSSCG00000007812	XPO6	2391.14	1.06	0.06	17.74	0.00	0.00
ENSSSCG00000004146	REPS1	1059.77	1.06	0.07	15.07	0.00	0.00

ENSSSCG00000006156		2412.07	1.06	0.05	20.09	0.00	0.00
ENSSSCG00000002859	ANKRD27	1103.86	1.06	0.08	13.65	0.00	0.00
ENSSSCG00000038178	FUT10	182.53	1.06	0.15	7.24	0.00	0.00
ENSSSCG00000003908	TSPAN1	823.95	1.06	0.07	14.17	0.00	0.00
ENSSSCG00000017883	GGT6	13.55	1.06	0.52	2.04	0.04	0.06
ENSSSCG00000005375	CORO2A	625.54	1.06	0.08	12.73	0.00	0.00
ENSSSCG00000031139	IKZF4	82.16	1.06	0.22	4.84	0.00	0.00
ENSSSCG00000012377	KIF4A	2345.12	1.06	0.06	18.85	0.00	0.00
ENSSSCG00000015559	NCF2	35.29	1.06	0.32	3.28	0.00	0.00
ENSSSCG00000026636	ZBTB4	256.53	1.06	0.14	7.76	0.00	0.00
ENSSSCG00000027946	MVP	3869.51	1.06	0.05	23.07	0.00	0.00
ENSSSCG00000007284	NCOA6	2771.63	1.06	0.05	20.16	0.00	0.00
ENSSSCG00000005911	OPLAH	1176.43	1.06	0.07	15.79	0.00	0.00
ENSSSCG00000000860	NUP37	153.26	1.06	0.16	6.81	0.00	0.00
ENSSSCG00000025393	SUSD2	770.42	1.06	0.08	13.24	0.00	0.00
ENSSSCG00000036610	ARMC8	397.55	1.06	0.10	10.33	0.00	0.00
ENSSSCG00000005778	HSBP1L1	563.22	1.06	0.10	10.54	0.00	0.00
ENSSSCG00000025126	LGI4	44.36	1.07	0.29	3.62	0.00	0.00
ENSSSCG00000011970	CMSS1	144.02	1.07	0.16	6.59	0.00	0.00
ENSSSCG00000017877	ANKFY1	1320.57	1.07	0.06	16.75	0.00	0.00
ENSSSCG00000005273	OSTF1	350.56	1.07	0.11	9.47	0.00	0.00
ENSSSCG00000006137	WWP1	519.26	1.07	0.09	11.70	0.00	0.00
ENSSSCG00000002403	VIPAS39	603.98	1.07	0.09	12.51	0.00	0.00
ENSSSCG00000010290	MCU	3968.42	1.07	0.05	21.59	0.00	0.00
ENSSSCG00000025610	AGAP3	302.04	1.07	0.12	8.61	0.00	0.00

ENSSSCG00000033338		32.83	1.07	0.33	3.21	0.00	0.00
ENSSSCG00000015930	DHRS9	88.34	1.07	0.21	5.13	0.00	0.00
ENSSSCG00000038913	HERC4	711.55	1.07	0.09	11.89	0.00	0.00
ENSSSCG00000016708	SKAP2	595.05	1.07	0.09	12.32	0.00	0.00
ENSSSCG00000035774	ERRFI1	2874.87	1.07	0.07	15.44	0.00	0.00
ENSSSCG00000002672	MEAK7	189.08	1.07	0.14	7.39	0.00	0.00
ENSSSCG00000036236	ELOVL6	257.28	1.07	0.14	7.63	0.00	0.00
ENSSSCG00000028108	ASAP3	156.72	1.07	0.16	6.70	0.00	0.00
ENSSSCG00000036113		463.14	1.07	0.10	10.70	0.00	0.00
ENSSSCG00000001660	PTK7	726.95	1.07	0.08	12.65	0.00	0.00
ENSSSCG00000017255	ABCA5	531.08	1.07	0.10	11.22	0.00	0.00
ENSSSCG00000002309	PLEKHD1	40.02	1.07	0.31	3.50	0.00	0.00
ENSSSCG00000010034		242.49	1.07	0.13	8.32	0.00	0.00
ENSSSCG00000036202	RF02162	18.78	1.07	0.45	2.40	0.02	0.02
ENSSSCG00000027565		205.23	1.07	0.14	7.83	0.00	0.00
ENSSSCG00000012532	TCEAL1	90.01	1.07	0.21	5.15	0.00	0.00
ENSSSCG00000014828	RAB6A	3529.55	1.07	0.05	19.70	0.00	0.00
ENSSSCG00000012875		273.58	1.08	0.13	8.56	0.00	0.00
ENSSSCG00000024463	PJA1	2903.11	1.08	0.05	20.21	0.00	0.00
ENSSSCG00000024341	USP20	158.75	1.08	0.16	6.69	0.00	0.00
ENSSSCG00000004832	UBE3A	1224.92	1.08	0.07	15.84	0.00	0.00
ENSSSCG00000009440	ELF1	1255.91	1.08	0.07	16.45	0.00	0.00
ENSSSCG00000004813	TTC23	52.92	1.08	0.28	3.82	0.00	0.00
ENSSSCG00000027509	VPS54	637.27	1.08	0.09	12.54	0.00	0.00
ENSSSCG00000008422		724.68	1.08	0.08	13.63	0.00	0.00

ENSSSCG00000010817	LYPLAL1	374.58	1.08	0.11	10.07	0.00	0.00
ENSSSCG00000010579	GBF1	1279.62	1.08	0.06	16.70	0.00	0.00
ENSSSCG00000015411	PTPN12	3690.05	1.08	0.05	22.37	0.00	0.00
ENSSSCG00000040513	AQP3	187.91	1.08	0.15	7.34	0.00	0.00
ENSSSCG00000014387		505.39	1.08	0.10	10.72	0.00	0.00
ENSSSCG00000017272	HELZ	1571.62	1.08	0.06	17.74	0.00	0.00
ENSSSCG00000028406	FOCAD	657.46	1.08	0.08	13.07	0.00	0.00
ENSSSCG00000006016	EIF3H	2799.61	1.08	0.05	20.63	0.00	0.00
ENSSSCG00000033355	LGI3	1483.42	1.08	0.06	17.44	0.00	0.00
ENSSSCG00000010513	LCOR	1810.37	1.08	0.13	8.07	0.00	0.00
ENSSSCG00000026354		274.44	1.08	0.12	8.99	0.00	0.00
ENSSSCG00000006037	OXR1	1155.18	1.08	0.07	15.36	0.00	0.00
ENSSSCG00000038486	WDR60	140.63	1.08	0.17	6.52	0.00	0.00
ENSSSCG00000005749	BRD3	925.73	1.08	0.08	13.31	0.00	0.00
ENSSSCG00000033527	ITFG1	813.95	1.08	0.08	13.50	0.00	0.00
ENSSSCG00000005711	NUP214	1489.43	1.08	0.06	17.33	0.00	0.00
ENSSSCG00000009320	FLT1	1104.34	1.08	0.08	14.18	0.00	0.00
ENSSSCG00000035025	ADGRV1	261.27	1.08	0.12	8.74	0.00	0.00
ENSSSCG00000035403	RFX2	227.22	1.08	0.13	8.10	0.00	0.00
ENSSSCG00000009377	THSD1	85.64	1.08	0.22	4.97	0.00	0.00
ENSSSCG00000031112	TM4SF5	107.47	1.08	0.19	5.80	0.00	0.00
ENSSSCG00000005518		1877.60	1.08	0.06	16.93	0.00	0.00
ENSSSCG00000004339	UFL1	741.31	1.08	0.08	13.07	0.00	0.00
ENSSSCG00000012743	MTMR1	744.58	1.08	0.08	13.52	0.00	0.00
ENSSSCG00000010107	MED15	1034.94	1.08	0.08	13.94	0.00	0.00

ENSSSCG00000004529	POLI	720.04	1.09	0.08	13.15	0.00	0.00
ENSSSCG00000017923	ALOX15	25.12	1.09	0.40	2.68	0.01	0.01
ENSSSCG00000012181	PDK3	875.87	1.09	0.08	14.13	0.00	0.00
ENSSSCG00000039062		14.75	1.09	0.52	2.10	0.04	0.05
ENSSSCG00000010735	LHPP	58.76	1.09	0.26	4.19	0.00	0.00
ENSSSCG00000037549	GCLM	2620.08	1.09	0.06	19.26	0.00	0.00
ENSSSCG00000011877	CD86	256.27	1.09	0.13	8.57	0.00	0.00
ENSSSCG00000035256		194.35	1.09	0.14	7.52	0.00	0.00
ENSSSCG00000006889	ARHGAP29	3184.47	1.09	0.05	22.07	0.00	0.00
ENSSSCG00000016314	TRPM8	101.77	1.09	0.19	5.68	0.00	0.00
ENSSSCG00000006218	PDE7A	125.52	1.09	0.17	6.34	0.00	0.00
ENSSSCG00000003396	CLSTN1	4025.65	1.09	0.05	23.77	0.00	0.00
ENSSSCG00000012437	ATP7A	903.00	1.09	0.08	14.27	0.00	0.00
ENSSSCG00000013410	SWAP70	1046.27	1.09	0.07	15.65	0.00	0.00
ENSSSCG00000035960		17.95	1.09	0.45	2.40	0.02	0.02
ENSSSCG00000027024	ASIC3	38.34	1.09	0.31	3.50	0.00	0.00
ENSSSCG00000031509	MTMR12	548.82	1.09	0.09	11.53	0.00	0.00
ENSSSCG00000037451	PPFIA1	903.27	1.09	0.07	14.60	0.00	0.00
ENSSSCG00000013491	TJP3	861.27	1.09	0.08	13.96	0.00	0.00
ENSSSCG00000015497	KLHL20	518.93	1.09	0.10	11.15	0.00	0.00
ENSSSCG00000024162	IMPA2	143.41	1.09	0.17	6.57	0.00	0.00
ENSSSCG00000008987	CCNI	5066.92	1.09	0.06	18.30	0.00	0.00
ENSSSCG00000014435	CSNK1A1	3277.21	1.09	0.05	20.79	0.00	0.00
ENSSSCG00000014794	NUP98	2894.23	1.10	0.05	20.60	0.00	0.00
ENSSSCG00000037468	GNE	1048.32	1.10	0.07	15.25	0.00	0.00

ENSSSCG00000011710	EIF2A	2609.25	1.10	0.06	19.81	0.00	0.00
ENSSSCG00000006893	BCAR3	559.26	1.10	0.09	12.25	0.00	0.00
ENSSSCG00000012122	TCEANC	271.26	1.10	0.12	8.84	0.00	0.00
ENSSSCG00000028004	RIN2	422.52	1.10	0.10	11.00	0.00	0.00
ENSSSCG00000006890	ABCA4	44.28	1.10	0.29	3.79	0.00	0.00
ENSSSCG00000036076	SAPCD1	15.71	1.10	0.56	1.97	0.05	0.07
ENSSSCG00000003651	RHBDL2	194.03	1.10	0.14	7.72	0.00	0.00
ENSSSCG00000007942	HMOX2	1561.02	1.10	0.06	18.01	0.00	0.00
ENSSSCG00000017602	STXBP4	117.07	1.10	0.18	5.99	0.00	0.00
ENSSSCG00000011504	EOGT	455.27	1.10	0.10	10.91	0.00	0.00
ENSSSCG00000040731	TMEM65	654.00	1.10	0.11	10.02	0.00	0.00
ENSSSCG00000010283	SPOCK2	26.07	1.10	0.41	2.68	0.01	0.01
ENSSSCG00000010564	POLL	281.99	1.10	0.12	9.22	0.00	0.00
ENSSSCG00000009680	EXTL3	1205.27	1.10	0.07	16.42	0.00	0.00
ENSSSCG00000014156	ARRDC3	4561.07	1.10	0.06	19.27	0.00	0.00
ENSSSCG00000024999	PPIP5K2	1404.60	1.10	0.08	14.52	0.00	0.00
ENSSSCG00000031557	CHCHD6	138.46	1.10	0.17	6.59	0.00	0.00
ENSSSCG00000039085		1055.47	1.10	0.07	15.91	0.00	0.00
ENSSSCG00000029438	SESN2	702.43	1.10	0.08	13.18	0.00	0.00
ENSSSCG00000032145	IRX5	243.44	1.10	0.13	8.48	0.00	0.00
ENSSSCG00000023653	GLIS2	558.42	1.10	0.09	11.83	0.00	0.00
ENSSSCG00000017920		478.89	1.10	0.09	11.61	0.00	0.00
ENSSSCG00000002872	KIAA0355	414.09	1.10	0.10	10.93	0.00	0.00
ENSSSCG00000037815	ZC3H12A	1425.78	1.10	0.08	14.68	0.00	0.00
ENSSSCG00000028671	CEL	45.46	1.10	0.28	3.90	0.00	0.00

ENSSSCG00000004293	SNX14	3520.12	1.10	0.05	21.78	0.00	0.00
ENSSSCG00000024592	ANKRD17	2281.24	1.11	0.05	20.12	0.00	0.00
ENSSSCG00000014378		53.25	1.11	0.30	3.65	0.00	0.00
ENSSSCG00000016743	OGDH	6058.56	1.11	0.05	24.00	0.00	0.00
ENSSSCG00000004344	MMS22L	332.73	1.11	0.12	9.53	0.00	0.00
ENSSSCG00000008188	MGAT4A	2653.46	1.11	0.06	19.77	0.00	0.00
ENSSSCG00000006202	CSPP1	1090.20	1.11	0.07	15.94	0.00	0.00
ENSSSCG00000036156	RBM12	491.26	1.11	0.10	10.64	0.00	0.00
ENSSSCG00000036451		218.42	1.11	0.13	8.25	0.00	0.00
ENSSSCG00000002916	APLP1	107.27	1.11	0.19	5.79	0.00	0.00
ENSSSCG00000022845	PJA2	1522.62	1.11	0.07	14.95	0.00	0.00
ENSSSCG00000009226	KLHL8	516.97	1.11	0.09	12.13	0.00	0.00
ENSSSCG00000021232	SYNC	88.23	1.11	0.24	4.67	0.00	0.00
ENSSSCG00000003143	MAMSTR	70.38	1.11	0.24	4.71	0.00	0.00
ENSSSCG00000006894	FNBP1L	2617.43	1.11	0.05	21.29	0.00	0.00
ENSSSCG00000017552	NXPH3	55.78	1.11	0.26	4.30	0.00	0.00
ENSSSCG00000015592	TATDN3	254.21	1.11	0.13	8.82	0.00	0.00
ENSSSCG00000008599		162.29	1.11	0.16	6.94	0.00	0.00
ENSSSCG00000020871	ARSD	441.52	1.11	0.10	11.24	0.00	0.00
ENSSSCG00000008242	KCMF1	1276.86	1.12	0.07	16.52	0.00	0.00
ENSSSCG00000022794		431.79	1.12	0.11	10.32	0.00	0.00
ENSSSCG00000000695	IFFO1	45.74	1.12	0.28	3.95	0.00	0.00
ENSSSCG00000011013	WAC	1578.60	1.12	0.06	17.99	0.00	0.00
ENSSSCG00000016981	CPEB4	2428.52	1.12	0.05	20.42	0.00	0.00
ENSSSCG00000024131	MINDY2	379.10	1.12	0.11	9.73	0.00	0.00

ENSSSCG00000017321	LYZL6	12.72	1.12	0.54	2.08	0.04	0.05
ENSSSCG00000010672	RAB11FIP2	433.97	1.12	0.10	10.95	0.00	0.00
ENSSSCG00000031180		88.09	1.12	0.21	5.37	0.00	0.00
ENSSSCG00000029305	FNDC4	27.65	1.12	0.36	3.08	0.00	0.00
ENSSSCG00000022208	TNFRSF1B	3553.26	1.12	0.05	20.52	0.00	0.00
ENSSSCG00000028172	FAM78A	27.64	1.12	0.36	3.06	0.00	0.00
ENSSSCG00000001201	ZKSCAN8	211.30	1.12	0.17	6.70	0.00	0.00
ENSSSCG00000020879	FBXW7	359.13	1.12	0.11	10.05	0.00	0.00
ENSSSCG00000001792	EFL1	1066.69	1.12	0.07	15.73	0.00	0.00
ENSSSCG00000010005	RNF215	209.42	1.12	0.15	7.42	0.00	0.00
ENSSSCG00000036145	PRKAA2	704.68	1.12	0.09	12.35	0.00	0.00
ENSSSCG00000000920	POC1B	271.62	1.12	0.12	8.98	0.00	0.00
ENSSSCG00000006562	GATAD2B	1162.24	1.12	0.08	13.35	0.00	0.00
ENSSSCG00000012267	JADE3	647.80	1.12	0.09	12.83	0.00	0.00
ENSSSCG00000014598	PPFIBP2	79.06	1.12	0.22	5.13	0.00	0.00
ENSSSCG00000028740	CTDSPL	125.10	1.12	0.18	6.29	0.00	0.00
ENSSSCG00000002528	TECPR2	189.18	1.12	0.15	7.68	0.00	0.00
ENSSSCG00000039224	CSPG5	61.91	1.12	0.24	4.60	0.00	0.00
ENSSSCG00000031105	C9orf85	127.48	1.12	0.17	6.45	0.00	0.00
ENSSSCG00000014121	ZFYVE16	1367.43	1.12	0.07	15.38	0.00	0.00
ENSSSCG00000009927	FAM222A	26.67	1.12	0.38	2.98	0.00	0.00
ENSSSCG00000008020	IFT140	144.82	1.12	0.17	6.80	0.00	0.00
ENSSSCG00000000160	PRDM4	748.67	1.12	0.08	14.02	0.00	0.00
ENSSSCG00000038406		390.62	1.12	0.11	10.51	0.00	0.00
ENSSSCG00000014315	LECT2	13.46	1.13	0.54	2.08	0.04	0.05

ENSSSCG00000028741		175.10	1.13	0.16	7.22	0.00	0.00
ENSSSCG00000009268	CRYL1	171.38	1.13	0.15	7.41	0.00	0.00
ENSSSCG00000004439	TSPYL4	57.97	1.13	0.26	4.32	0.00	0.00
ENSSSCG00000008009	LMF1	413.69	1.13	0.10	11.11	0.00	0.00
ENSSSCG00000036505		6966.60	1.13	0.05	22.18	0.00	0.00
ENSSSCG00000028538	RLIM	1470.27	1.13	0.07	15.86	0.00	0.00
ENSSSCG00000008768	ARAP2	163.51	1.13	0.15	7.35	0.00	0.00
ENSSSCG00000016606	ASB15	32.71	1.13	0.34	3.35	0.00	0.00
ENSSSCG00000002508	SETD3	804.21	1.13	0.08	14.24	0.00	0.00
ENSSSCG00000015798	ANKRD37	592.37	1.13	0.19	5.89	0.00	0.00
ENSSSCG00000029226	UBC	3196.00	1.13	0.13	8.70	0.00	0.00
ENSSSCG00000023983	AGPS	1865.71	1.13	0.06	17.80	0.00	0.00
ENSSSCG00000016754	AEBP1	62.80	1.13	0.25	4.45	0.00	0.00
ENSSSCG00000010912	KIF14	692.66	1.13	0.09	13.03	0.00	0.00
ENSSSCG00000022708	ZNF638	1701.69	1.13	0.06	18.69	0.00	0.00
ENSSSCG00000028612	PTPRS	560.82	1.13	0.09	12.55	0.00	0.00
ENSSSCG00000036191	SLC17A2	47.25	1.13	0.28	4.04	0.00	0.00
ENSSSCG00000028552	BHLHE41	3676.95	1.13	0.06	19.04	0.00	0.00
ENSSSCG00000027489	TMCO4	117.56	1.13	0.18	6.14	0.00	0.00
ENSSSCG00000025821	C2CD2	697.52	1.13	0.08	13.77	0.00	0.00
ENSSSCG00000000295	NCKAP1L	899.13	1.13	0.07	15.46	0.00	0.00
ENSSSCG00000013233	CELF1	2571.21	1.13	0.05	21.13	0.00	0.00
ENSSSCG00000017783		2632.09	1.13	0.05	22.08	0.00	0.00
ENSSSCG00000005235	KANK1	1395.29	1.13	0.07	15.90	0.00	0.00
ENSSSCG00000033189	FAM107A	158.31	1.14	0.17	6.86	0.00	0.00

ENSSSCG00000005081	LRRC9	281.76	1.14	0.12	9.43	0.00	0.00
ENSSSCG00000013933	PBX4	77.34	1.14	0.22	5.18	0.00	0.00
ENSSSCG00000022155	RBPJ	852.41	1.14	0.17	6.69	0.00	0.00
ENSSSCG00000002329	MAP3K9	646.85	1.14	0.09	13.09	0.00	0.00
ENSSSCG00000009302	USP12	372.64	1.14	0.11	10.63	0.00	0.00
ENSSSCG00000014959	PIWIL4	237.86	1.14	0.13	8.65	0.00	0.00
ENSSSCG00000008796	RBM47	1200.50	1.14	0.07	15.42	0.00	0.00
ENSSSCG00000010968		130.43	1.14	0.18	6.47	0.00	0.00
ENSSSCG00000037427	PTDSS2	353.09	1.14	0.11	10.61	0.00	0.00
ENSSSCG00000034159	SNAPIN	528.83	1.14	0.09	12.16	0.00	0.00
ENSSSCG00000011933	NECTIN3	696.66	1.14	0.09	12.71	0.00	0.00
ENSSSCG00000036724	CRYAB	27.30	1.14	0.38	3.02	0.00	0.00
ENSSSCG00000039194	KANK2	1695.21	1.14	0.06	18.14	0.00	0.00
ENSSSCG00000025616	SENP8	99.82	1.15	0.20	5.85	0.00	0.00
ENSSSCG00000010639	HABP2	596.64	1.15	0.10	11.64	0.00	0.00
ENSSSCG00000009705	GALNT7	706.19	1.15	0.08	14.16	0.00	0.00
ENSSSCG00000031206	TFCP2	506.39	1.15	0.10	12.07	0.00	0.00
ENSSSCG00000008629	ROCK2	1361.37	1.15	0.07	15.75	0.00	0.00
ENSSSCG00000027859	HDAC7	959.60	1.15	0.07	16.02	0.00	0.00
ENSSSCG00000005631	FAM102A	631.31	1.15	0.08	13.63	0.00	0.00
ENSSSCG00000010586	SUFU	102.86	1.15	0.19	6.01	0.00	0.00
ENSSSCG00000033009		109.16	1.15	0.20	5.77	0.00	0.00
ENSSSCG00000027812	IQSEC1	212.78	1.15	0.14	8.33	0.00	0.00
ENSSSCG00000034921	CLDN19	33.03	1.15	0.33	3.45	0.00	0.00
ENSSSCG00000007282	PIGU	592.88	1.15	0.09	12.48	0.00	0.00

ENSSSCG00000031733	AKAP12	2481.30	1.15	0.06	20.76	0.00	0.00
ENSSSCG00000005620	SH2D3C	180.32	1.15	0.15	7.79	0.00	0.00
ENSSSCG00000009658	BNIP3L	3796.79	1.15	0.06	18.13	0.00	0.00
ENSSSCG00000031102	C1orf174	267.64	1.15	0.14	8.50	0.00	0.00
ENSSSCG00000011425	RAD54L2	678.79	1.15	0.09	12.95	0.00	0.00
ENSSSCG00000039953	PNRC1	1520.30	1.16	0.08	15.27	0.00	0.00
ENSSSCG00000037101	LRRC8B	124.28	1.16	0.18	6.32	0.00	0.00
ENSSSCG00000015695	RAB3GAP1	680.38	1.16	0.08	13.69	0.00	0.00
ENSSSCG00000004275	FAM135A	998.13	1.16	0.07	15.67	0.00	0.00
ENSSSCG00000035788		16.34	1.16	0.49	2.38	0.02	0.03
ENSSSCG00000000970	PPP6R2	570.49	1.16	0.09	12.95	0.00	0.00
ENSSSCG00000022073	ZBTB38	1052.25	1.16	0.08	14.94	0.00	0.00
ENSSSCG00000031199		67.88	1.16	0.24	4.78	0.00	0.00
ENSSSCG00000007978		28.14	1.16	0.38	3.06	0.00	0.00
ENSSSCG00000004368	PREP	825.14	1.16	0.08	14.75	0.00	0.00
ENSSSCG00000015955	ITGA6	4737.27	1.16	0.05	24.82	0.00	0.00
ENSSSCG00000014211	YTHDC2	1518.13	1.16	0.07	16.03	0.00	0.00
ENSSSCG00000011551	TADA3	603.19	1.17	0.08	13.72	0.00	0.00
ENSSSCG00000013078	MYRF	750.54	1.17	0.08	14.90	0.00	0.00
ENSSSCG00000002959	FAM98C	389.89	1.17	0.11	10.77	0.00	0.00
ENSSSCG00000024361	TFB1M	233.52	1.17	0.14	8.58	0.00	0.00
ENSSSCG00000027487	LAT2	3023.84	1.17	0.05	21.45	0.00	0.00
ENSSSCG00000030291	RPRD2	1462.52	1.17	0.06	19.00	0.00	0.00
ENSSSCG00000001438	PBX2	452.10	1.17	0.10	11.92	0.00	0.00
ENSSSCG00000022240	POLA1	171.93	1.17	0.16	7.44	0.00	0.00

ENSSSCG00000011575	ATG7	215.74	1.17	0.14	8.56	0.00	0.00
ENSSSCG00000009397	CAB39L	547.04	1.17	0.09	12.48	0.00	0.00
ENSSSCG00000013576	STXBP2	1599.04	1.17	0.06	18.97	0.00	0.00
ENSSSCG00000006571	INTS3	1388.28	1.17	0.07	17.88	0.00	0.00
ENSSSCG00000009335	B3GLCT	539.64	1.17	0.09	12.76	0.00	0.00
ENSSSCG00000005316	TPM2	205.06	1.17	0.14	8.21	0.00	0.00
ENSSSCG00000000647	OLR1	97.56	1.17	0.25	4.75	0.00	0.00
ENSSSCG00000010533	PYROXD2	176.78	1.17	0.15	7.60	0.00	0.00
ENSSSCG00000000293	ITGA5	6597.35	1.17	0.05	21.80	0.00	0.00
ENSSSCG00000031723	RAB39A	10.93	1.17	0.60	1.97	0.05	0.07
ENSSSCG00000009944	CORO1C	2415.89	1.17	0.06	21.20	0.00	0.00
ENSSSCG00000029420	ZNF569	48.64	1.17	0.28	4.21	0.00	0.00
ENSSSCG00000035027	ZDHHC14	119.20	1.17	0.18	6.40	0.00	0.00
ENSSSCG00000037557	DRC3	305.94	1.18	0.13	9.24	0.00	0.00
ENSSSCG00000021893	ROCK1	1596.22	1.18	0.07	16.79	0.00	0.00
ENSSSCG00000011424	TEX264	1281.60	1.18	0.07	17.78	0.00	0.00
ENSSSCG00000015720	BIN1	676.19	1.18	0.09	13.46	0.00	0.00
ENSSSCG00000014800	RNF121	145.34	1.18	0.16	7.23	0.00	0.00
ENSSSCG00000004132	PHACTR2	81.10	1.18	0.22	5.40	0.00	0.00
ENSSSCG00000005308	RUSC2	67.08	1.18	0.24	4.85	0.00	0.00
ENSSSCG00000006738	CD58	281.39	1.18	0.13	9.42	0.00	0.00
ENSSSCG00000000108	TMEM184B	1242.60	1.18	0.07	16.17	0.00	0.00
ENSSSCG00000003081	CEACAM16	73.34	1.18	0.23	5.15	0.00	0.00
ENSSSCG00000015789	SNX25	259.59	1.18	0.13	8.85	0.00	0.00
ENSSSCG00000010009	GAL3ST1	208.86	1.18	0.14	8.56	0.00	0.00

ENSSSCG00000032214		619.22	1.18	0.10	12.14	0.00	0.00
ENSSSCG00000013639	SLC44A2	562.30	1.18	0.09	13.29	0.00	0.00
ENSSSCG00000017282	SCN4A	65.26	1.18	0.24	4.83	0.00	0.00
ENSSSCG00000014113	HOMER1	453.95	1.18	0.10	11.98	0.00	0.00
ENSSSCG00000003872	EPS15	2819.57	1.18	0.05	23.14	0.00	0.00
ENSSSCG00000031593	NUDT3	562.84	1.18	0.09	13.15	0.00	0.00
ENSSSCG00000003479	MFAP2	13.18	1.19	0.53	2.24	0.02	0.04
ENSSSCG00000016853	RICTOR	713.43	1.19	0.10	12.09	0.00	0.00
ENSSSCG00000011193	BTD	506.66	1.19	0.09	12.57	0.00	0.00
ENSSSCG00000028135	PRTFDC1	1522.37	1.19	0.06	19.21	0.00	0.00
ENSSSCG00000024563	CEP83	350.65	1.19	0.12	10.16	0.00	0.00
ENSSSCG00000004233	SMPDL3A	63.85	1.19	0.25	4.81	0.00	0.00
ENSSSCG00000037508	GSN	3306.33	1.19	0.05	22.01	0.00	0.00
ENSSSCG00000017201	UNK	508.82	1.19	0.10	12.08	0.00	0.00
ENSSSCG00000008022	TELO2	1665.47	1.19	0.06	20.31	0.00	0.00
ENSSSCG00000007956	NLRC3	244.43	1.19	0.13	8.85	0.00	0.00
ENSSSCG00000022534	ZNF644	842.19	1.19	0.08	15.62	0.00	0.00
ENSSSCG00000006101		415.38	1.19	0.10	11.63	0.00	0.00
ENSSSCG00000039661	SSPO	24.89	1.19	0.40	3.00	0.00	0.00
ENSSSCG00000036201	NPR3	151.60	1.19	0.16	7.35	0.00	0.00
ENSSSCG00000028725	TMEM102	162.27	1.19	0.16	7.38	0.00	0.00
ENSSSCG00000011340	SMARCC1	1408.18	1.19	0.06	19.24	0.00	0.00
ENSSSCG00000003344	VWA1	274.50	1.19	0.12	9.72	0.00	0.00
ENSSSCG00000022504	CDON	88.32	1.19	0.22	5.52	0.00	0.00
ENSSSCG00000026554	PRXL2B	51.27	1.19	0.27	4.39	0.00	0.00

ENSSSCG00000011837	MELTF	1081.10	1.19	0.07	16.24	0.00	0.00
ENSSSCG00000010594	NT5C2	1605.22	1.19	0.06	18.64	0.00	0.00
ENSSSCG00000004612	RAB27A	1025.45	1.19	0.08	14.83	0.00	0.00
ENSSSCG00000039701	DCAF5	1313.01	1.19	0.07	18.23	0.00	0.00
ENSSSCG00000039368		71.65	1.19	0.23	5.12	0.00	0.00
ENSSSCG00000024389	SIGIRR	220.90	1.19	0.13	8.99	0.00	0.00
ENSSSCG00000037395	CLIC3	37.04	1.20	0.32	3.78	0.00	0.00
ENSSSCG00000028225	DICER1	1817.62	1.20	0.06	20.79	0.00	0.00
ENSSSCG00000007721	GTF2I	2292.73	1.20	0.06	21.03	0.00	0.00
ENSSSCG00000032984		159.90	1.20	0.16	7.67	0.00	0.00
ENSSSCG00000023487	MSLNL	37.48	1.20	0.34	3.51	0.00	0.00
ENSSSCG00000014884		545.60	1.20	0.09	12.65	0.00	0.00
ENSSSCG00000030882		12.17	1.20	0.56	2.14	0.03	0.05
ENSSSCG00000007602	BAIAP2L1	1169.35	1.20	0.07	17.55	0.00	0.00
ENSSSCG00000034156	GTF2F2	557.08	1.20	0.09	13.28	0.00	0.00
ENSSSCG00000009298	CDK8	621.31	1.20	0.09	13.26	0.00	0.00
ENSSSCG00000031488	ARHGAP17	455.58	1.20	0.10	11.75	0.00	0.00
ENSSSCG00000021173	BSPRY	396.23	1.20	0.10	11.66	0.00	0.00
ENSSSCG00000005905	SLC39A4	89.41	1.20	0.21	5.64	0.00	0.00
ENSSSCG00000008068	IARS	8557.04	1.20	0.05	25.37	0.00	0.00
ENSSSCG00000016420	INSIG1	397.16	1.20	0.13	9.45	0.00	0.00
ENSSSCG00000023948	UBTD2	744.67	1.20	0.08	15.07	0.00	0.00
ENSSSCG00000035891	ADGRB1	67.04	1.20	0.25	4.86	0.00	0.00
ENSSSCG00000016653	DNAJB9	432.20	1.20	0.10	11.75	0.00	0.00
ENSSSCG00000008591	ATAD2B	351.91	1.20	0.11	11.06	0.00	0.00

ENSSSCG00000007260	BPIFA1	27.25	1.21	0.38	3.18	0.00	0.00
ENSSSCG00000017376	MEOX1	583.96	1.21	0.09	13.68	0.00	0.00
ENSSSCG00000030681	MYBPH	15.59	1.21	0.51	2.36	0.02	0.03
ENSSSCG00000039847	C1S	161.08	1.21	0.18	6.88	0.00	0.00
ENSSSCG00000006978		48.95	1.21	0.29	4.16	0.00	0.00
ENSSSCG00000017880	SPNS3	148.04	1.21	0.17	7.21	0.00	0.00
ENSSSCG00000008402	CLHC1	120.26	1.21	0.19	6.25	0.00	0.00
ENSSSCG00000002844		923.35	1.21	0.08	15.14	0.00	0.00
ENSSSCG00000009040	SMAD1	455.62	1.21	0.10	12.42	0.00	0.00
ENSSSCG00000040657	STRN3	897.19	1.21	0.08	14.99	0.00	0.00
ENSSSCG00000023419	ARHGEF10	615.67	1.21	0.09	13.62	0.00	0.00
ENSSSCG00000013296	SLC1A2	158.85	1.21	0.16	7.69	0.00	0.00
ENSSSCG00000011854	LRCH3	940.60	1.21	0.08	16.11	0.00	0.00
ENSSSCG00000004857	ATP9B	108.20	1.21	0.19	6.41	0.00	0.00
ENSSSCG00000003818	DOCK7	2496.28	1.21	0.06	21.03	0.00	0.00
ENSSSCG00000012328	HUWE1	10737.96	1.21	0.05	26.80	0.00	0.00
ENSSSCG00000035379	JCHAIN	15.69	1.21	0.53	2.31	0.02	0.03
ENSSSCG00000007074	TASP1	113.49	1.21	0.18	6.56	0.00	0.00
ENSSSCG00000027857	DMXL1	2397.39	1.21	0.07	18.16	0.00	0.00
ENSSSCG00000008687	MXD4	122.08	1.21	0.18	6.87	0.00	0.00
ENSSSCG00000006088	SDC2	598.69	1.21	0.09	13.22	0.00	0.00
ENSSSCG00000037264	RAB31	1027.35	1.22	0.07	17.33	0.00	0.00
ENSSSCG00000014838	PGM2L1	52.33	1.22	0.27	4.47	0.00	0.00
ENSSSCG00000022004	SH3BP2	331.32	1.22	0.12	10.30	0.00	0.00
ENSSSCG00000033010	AHNAK	22909.83	1.22	0.05	25.18	0.00	0.00

ENSSSCG00000010654	ATRNL1	30.20	1.22	0.35	3.44	0.00	0.00
ENSSSCG00000004144	HECA	193.22	1.22	0.15	7.99	0.00	0.00
ENSSSCG00000027860	ERAP2	108.66	1.22	0.19	6.26	0.00	0.00
ENSSSCG00000032632	CMTM4	1176.48	1.22	0.09	14.22	0.00	0.00
ENSSSCG00000008478	SOS1	1175.31	1.22	0.07	18.39	0.00	0.00
ENSSSCG00000008293	DGUOK	179.79	1.22	0.15	8.10	0.00	0.00
ENSSSCG00000005524	DAB2IP	764.64	1.22	0.08	15.03	0.00	0.00
ENSSSCG00000021906	SYT7	525.23	1.22	0.09	13.30	0.00	0.00
ENSSSCG00000002637		53.02	1.22	0.27	4.59	0.00	0.00
ENSSSCG00000015322	TFPI2	463.46	1.22	0.10	12.15	0.00	0.00
ENSSSCG00000026744	ZDHHC20	2671.79	1.22	0.05	22.31	0.00	0.00
ENSSSCG00000032601	RASSF2	75.22	1.22	0.22	5.44	0.00	0.00
ENSSSCG00000021397	PODXL2	103.00	1.22	0.20	6.27	0.00	0.00
ENSSSCG00000034853		4607.08	1.22	0.07	18.68	0.00	0.00
ENSSSCG00000006502	ARHGEF2	4781.33	1.22	0.05	25.19	0.00	0.00
ENSSSCG00000037846	ACOXL	22.95	1.22	0.40	3.05	0.00	0.00
ENSSSCG00000022353	RAP1GDS1	1335.02	1.22	0.07	17.55	0.00	0.00
ENSSSCG00000010331	ANXA11	2894.16	1.23	0.05	23.09	0.00	0.00
ENSSSCG00000010026	PIK3IP1	188.98	1.23	0.14	8.50	0.00	0.00
ENSSSCG00000028322	BTG2	1280.08	1.23	0.08	14.77	0.00	0.00
ENSSSCG00000031141	ABCA13	11.20	1.23	0.59	2.09	0.04	0.05
ENSSSCG00000038873	SLMAP	1422.85	1.23	0.06	19.57	0.00	0.00
ENSSSCG00000015235	ETS1	3060.87	1.23	0.05	22.41	0.00	0.00
ENSSSCG00000032367	CEBPD	1114.25	1.23	0.08	15.98	0.00	0.00
ENSSSCG00000000753	WNK1	3039.88	1.23	0.05	23.21	0.00	0.00

ENSSSCG00000003738	MAPRE2	774.29	1.23	0.08	15.43	0.00	0.00
ENSSSCG00000026596	SYNRG	1252.70	1.23	0.07	18.34	0.00	0.00
ENSSSCG00000040388	ZBTB46	166.01	1.23	0.15	7.95	0.00	0.00
ENSSSCG00000001970	HEATR5A	1371.19	1.23	0.07	18.39	0.00	0.00
ENSSSCG00000015818	DDHD2	959.79	1.23	0.08	16.37	0.00	0.00
ENSSSCG00000003257	CACNG6	66.92	1.23	0.24	5.20	0.00	0.00
ENSSSCG00000003749	PIK3C3	343.77	1.23	0.11	11.29	0.00	0.00
ENSSSCG00000013572	PNPLA6	1308.48	1.23	0.07	18.70	0.00	0.00
ENSSSCG00000001641	UBR2	954.06	1.23	0.08	15.29	0.00	0.00
ENSSSCG00000017546	ZNF652	171.38	1.23	0.17	7.42	0.00	0.00
ENSSSCG00000013174	CTNND1	3388.95	1.23	0.05	22.99	0.00	0.00
ENSSSCG00000013506	STAP2	134.42	1.23	0.17	7.17	0.00	0.00
ENSSSCG00000038057	TAC4	49.22	1.23	0.29	4.25	0.00	0.00
ENSSSCG00000009326	KATNAL1	474.50	1.23	0.10	12.37	0.00	0.00
ENSSSCG00000013237	SPI1	108.23	1.23	0.20	6.20	0.00	0.00
ENSSSCG00000003719		167.15	1.24	0.15	8.13	0.00	0.00
ENSSSCG00000014101	AP3B1	1373.86	1.24	0.08	16.37	0.00	0.00
ENSSSCG00000016518	TRIM24	834.50	1.24	0.07	16.49	0.00	0.00
ENSSSCG00000029843	NPHP4	368.98	1.24	0.11	11.12	0.00	0.00
ENSSSCG00000015399	SEMA3E	482.12	1.24	0.10	12.22	0.00	0.00
ENSSSCG00000014802	NUMA1	3900.66	1.24	0.05	26.25	0.00	0.00
ENSSSCG00000016119		572.88	1.24	0.09	14.02	0.00	0.00
ENSSSCG00000002495	SYNE3	426.43	1.24	0.10	12.28	0.00	0.00
ENSSSCG00000008498	HEATR5B	981.74	1.24	0.07	17.20	0.00	0.00
ENSSSCG00000027890		304.80	1.24	0.12	10.23	0.00	0.00

ENSSSCG00000002041	SLC7A7	357.73	1.24	0.11	10.79	0.00	0.00
ENSSSCG00000008682	NSD2	1635.31	1.24	0.06	20.79	0.00	0.00
ENSSSCG00000000694	GAPDH	99400.74	1.24	0.04	28.73	0.00	0.00
ENSSSCG00000010402	ZFAND4	132.17	1.24	0.18	7.10	0.00	0.00
ENSSSCG00000032097	DUSP16	706.26	1.24	0.08	14.83	0.00	0.00
ENSSSCG00000009862	RNFT2	115.50	1.25	0.19	6.71	0.00	0.00
ENSSSCG00000030130	DPYSL2	1170.93	1.25	0.07	18.42	0.00	0.00
ENSSSCG00000022496	STK39	196.84	1.25	0.14	8.72	0.00	0.00
ENSSSCG00000029536	SCTR	78.84	1.25	0.22	5.66	0.00	0.00
ENSSSCG00000037746	NHLRC3	381.19	1.25	0.10	12.03	0.00	0.00
ENSSSCG00000009569	PSPC1	752.16	1.25	0.08	14.85	0.00	0.00
ENSSSCG00000008529	YPEL5	628.98	1.25	0.10	12.42	0.00	0.00
ENSSSCG00000001950		117.93	1.25	0.18	6.87	0.00	0.00
ENSSSCG00000020864	VDR	814.43	1.25	0.08	16.41	0.00	0.00
ENSSSCG00000010614	CFAP58	9.68	1.25	0.62	2.01	0.04	0.06
ENSSSCG00000034192	GNAO1	64.78	1.25	0.25	5.05	0.00	0.00
ENSSSCG00000009872	PLBD2	4577.31	1.25	0.05	25.54	0.00	0.00
ENSSSCG00000007979	LUC7L	758.55	1.25	0.08	15.29	0.00	0.00
ENSSSCG00000033497	RERE	1354.67	1.25	0.08	15.08	0.00	0.00
ENSSSCG00000001860	NRG4	39.18	1.25	0.31	4.03	0.00	0.00
ENSSSCG00000000793	PPHLN1	732.14	1.25	0.08	14.84	0.00	0.00
ENSSSCG00000020678	BANP	182.99	1.25	0.15	8.50	0.00	0.00
ENSSSCG00000038604	DOCK6	1256.40	1.25	0.07	18.79	0.00	0.00
ENSSSCG00000000402	RBMS2	1451.00	1.25	0.07	18.63	0.00	0.00
ENSSSCG00000004012	THBS2	109.30	1.25	0.19	6.55	0.00	0.00

ENSSSCG00000009793	CLIP1	2099.04	1.25	0.06	21.62	0.00	0.00
ENSSSCG00000010653	TRUB1	19.36	1.25	0.44	2.87	0.00	0.01
ENSSSCG00000028737	USP3	776.35	1.25	0.08	16.18	0.00	0.00
ENSSSCG00000034441	MRGPRF	17.61	1.25	0.47	2.69	0.01	0.01
ENSSSCG00000003682	ANKRD12	1650.35	1.25	0.07	18.93	0.00	0.00
ENSSSCG00000006717	PHGDH	1719.55	1.26	0.08	16.24	0.00	0.00
ENSSSCG00000039269	SHLD1	35.28	1.26	0.33	3.83	0.00	0.00
ENSSSCG00000013402	USP47	1852.64	1.26	0.07	18.90	0.00	0.00
ENSSSCG00000038235		218.44	1.26	0.14	8.72	0.00	0.00
ENSSSCG00000016158	LANCL1	725.83	1.26	0.08	14.98	0.00	0.00
ENSSSCG00000022994	CYB5R4	110.00	1.26	0.19	6.76	0.00	0.00
ENSSSCG00000015586	RPS6KC1	934.29	1.26	0.07	17.60	0.00	0.00
ENSSSCG00000011857	LMLN	108.75	1.26	0.19	6.53	0.00	0.00
ENSSSCG00000010476	MYOF	14258.68	1.26	0.05	25.81	0.00	0.00
ENSSSCG00000006310	POU2F1	162.25	1.26	0.16	8.13	0.00	0.00
ENSSSCG00000039780	RTN4RL1	442.36	1.26	0.10	12.25	0.00	0.00
ENSSSCG00000014291	AFF4	4743.49	1.27	0.05	26.50	0.00	0.00
ENSSSCG00000005015	SOS2	785.93	1.27	0.08	15.24	0.00	0.00
ENSSSCG00000005205	KIAA2026	255.42	1.27	0.13	9.78	0.00	0.00
ENSSSCG00000024694	TAF4	393.61	1.27	0.11	12.02	0.00	0.00
ENSSSCG00000011594	NR2C2	344.78	1.27	0.12	10.49	0.00	0.00
ENSSSCG00000008051	ABCA3	562.32	1.27	0.10	12.47	0.00	0.00
ENSSSCG00000033530	RF02124	17.26	1.27	0.49	2.61	0.01	0.01
ENSSSCG00000023862	MTMR8	96.77	1.27	0.20	6.22	0.00	0.00
ENSSSCG00000000884	APAF1	420.18	1.27	0.10	12.18	0.00	0.00

ENSSSCG00000038801	NPNT	8440.58	1.27	0.05	26.98	0.00	0.00
ENSSSCG00000010052	BCR	713.14	1.27	0.08	15.22	0.00	0.00
ENSSSCG00000023054	IDO2	41.97	1.27	0.31	4.05	0.00	0.00
ENSSSCG00000029901	UBE2O	1238.05	1.27	0.07	19.10	0.00	0.00
ENSSSCG00000009069	C4orf33	55.17	1.27	0.26	4.85	0.00	0.00
ENSSSCG00000040248		637.52	1.27	0.08	15.09	0.00	0.00
ENSSSCG00000027017	NR1D2	755.48	1.28	0.08	15.50	0.00	0.00
ENSSSCG00000030685	ARID2	821.63	1.28	0.08	15.43	0.00	0.00
ENSSSCG00000014161		21.98	1.28	0.42	3.01	0.00	0.00
ENSSSCG00000003582	SMPDL3B	418.30	1.28	0.11	12.13	0.00	0.00
ENSSSCG00000031706		137.73	1.28	0.17	7.52	0.00	0.00
ENSSSCG00000010955	GOLM1	4851.08	1.28	0.05	27.14	0.00	0.00
ENSSSCG00000025876	PBLD	350.64	1.28	0.12	11.07	0.00	0.00
ENSSSCG00000022429	KAZALD1	86.34	1.28	0.21	5.97	0.00	0.00
ENSSSCG00000017624	CUEDC1	532.15	1.28	0.10	12.84	0.00	0.00
ENSSSCG00000009216	SPP1	92560.76	1.28	0.04	29.06	0.00	0.00
ENSSSCG00000015998	PJVK	13.75	1.28	0.52	2.46	0.01	0.02
ENSSSCG00000036075	WASHC3	291.28	1.28	0.12	10.46	0.00	0.00
ENSSSCG00000023812		116.00	1.28	0.19	6.91	0.00	0.00
ENSSSCG00000014249	MARCH3	78.73	1.28	0.22	5.78	0.00	0.00
ENSSSCG00000008601	SDC1	2074.57	1.28	0.06	23.09	0.00	0.00
ENSSSCG00000003909	PIK3R3	116.27	1.28	0.18	6.94	0.00	0.00
ENSSSCG00000005659	ZER1	505.11	1.28	0.10	13.23	0.00	0.00
ENSSSCG00000004971	TLE3	1586.97	1.28	0.06	20.54	0.00	0.00
ENSSSCG00000009250	PRKG2	1223.58	1.28	0.07	19.33	0.00	0.00

ENSSSCG00000040130		21.95	1.29	0.41	3.12	0.00	0.00
ENSSSCG00000004823	LRRK1	290.42	1.29	0.12	10.65	0.00	0.00
ENSSSCG00000022434	ZNF235	9.85	1.29	0.62	2.08	0.04	0.05
ENSSSCG00000000021		81.32	1.29	0.22	5.90	0.00	0.00
ENSSSCG00000024697	TMEM125	131.19	1.29	0.18	7.27	0.00	0.00
ENSSSCG00000002280	RAB15	115.26	1.29	0.19	6.85	0.00	0.00
ENSSSCG00000002670	USP10	1549.78	1.29	0.06	19.97	0.00	0.00
ENSSSCG00000038483		63.71	1.29	0.26	4.93	0.00	0.00
ENSSSCG00000000278		1331.62	1.29	0.07	17.77	0.00	0.00
ENSSSCG00000032007	RTN4	4504.73	1.29	0.05	26.54	0.00	0.00
ENSSSCG00000002540	PPP2R5C	1396.97	1.29	0.07	19.69	0.00	0.00
ENSSSCG00000009666	EPHX2	727.29	1.29	0.08	15.78	0.00	0.00
ENSSSCG00000033842	CFDP1	1416.37	1.29	0.06	20.07	0.00	0.00
ENSSSCG00000008952	RASSF6	369.31	1.29	0.11	12.10	0.00	0.00
ENSSSCG00000001074	KDM1B	1595.26	1.29	0.06	20.91	0.00	0.00
ENSSSCG00000009714	NEK1	351.17	1.29	0.11	11.64	0.00	0.00
ENSSSCG00000016769	CDK13	564.96	1.30	0.09	14.37	0.00	0.00
ENSSSCG00000011589	IFT122	889.07	1.30	0.08	16.65	0.00	0.00
ENSSSCG00000025685	TP73	522.24	1.30	0.11	12.13	0.00	0.00
ENSSSCG00000005930	SLC45A4	155.07	1.30	0.16	8.10	0.00	0.00
ENSSSCG00000017300		148.58	1.30	0.16	7.93	0.00	0.00
ENSSSCG00000013366		27185.22	1.30	0.05	27.48	0.00	0.00
ENSSSCG00000010546		20.13	1.30	0.47	2.78	0.01	0.01
ENSSSCG00000015555	LAMC1	9496.48	1.30	0.04	30.02	0.00	0.00
ENSSSCG00000017812	VPS53	455.29	1.30	0.10	13.45	0.00	0.00

ENSSSCG00000005469	KIAA1958	140.57	1.30	0.17	7.59	0.00	0.00
ENSSSCG00000003700	ESCO1	402.27	1.30	0.12	11.18	0.00	0.00
ENSSSCG00000001871	HMG20A	1190.96	1.30	0.07	18.85	0.00	0.00
ENSSSCG00000007430	NEURL2	10.52	1.30	0.60	2.17	0.03	0.04
ENSSSCG00000014316	TGFBI	729.23	1.30	0.09	14.90	0.00	0.00
ENSSSCG00000033358	VPS37D	126.84	1.30	0.20	6.60	0.00	0.00
ENSSSCG00000031474	PTK6	78.17	1.30	0.24	5.50	0.00	0.00
ENSSSCG00000003722	CDH2	37.31	1.30	0.64	2.03	0.04	0.06
ENSSSCG00000038994	CLIC4	6842.21	1.30	0.07	19.61	0.00	0.00
ENSSSCG00000014410	SH3RF2	293.74	1.30	0.12	10.91	0.00	0.00
ENSSSCG00000009393	EBPL	583.32	1.31	0.09	14.59	0.00	0.00
ENSSSCG00000034692	SH3BP4	1976.63	1.31	0.06	21.73	0.00	0.00
ENSSSCG00000020906	TNFSF10	441.62	1.31	0.10	12.60	0.00	0.00
ENSSSCG00000015011		381.18	1.31	0.11	11.81	0.00	0.00
ENSSSCG00000028274	FAM131B	31.64	1.31	0.35	3.78	0.00	0.00
ENSSSCG00000000707	SCNN1A	747.99	1.31	0.08	16.39	0.00	0.00
ENSSSCG00000011394	RBM6	702.21	1.31	0.08	15.91	0.00	0.00
ENSSSCG00000001782	ABHD17C	435.35	1.31	0.10	12.81	0.00	0.00
ENSSSCG00000016291	GIGYF2	1094.01	1.31	0.07	19.06	0.00	0.00
ENSSSCG00000007659	ZCWPW1	35.81	1.31	0.33	3.96	0.00	0.00
ENSSSCG00000013773	ADGRL1	290.62	1.31	0.12	11.07	0.00	0.00
ENSSSCG00000006923	GBP2	78.79	1.31	0.22	5.94	0.00	0.00
ENSSSCG00000002786	ELMO3	962.52	1.31	0.08	17.17	0.00	0.00
ENSSSCG00000022343	ENO1	47537.18	1.31	0.04	34.42	0.00	0.00
ENSSSCG00000034742	MECP2	282.41	1.31	0.12	10.90	0.00	0.00

ENSSSCG00000034259	PMEPA1	1189.60	1.31	0.07	18.84	0.00	0.00
ENSSSCG00000000094	SUN2	1739.04	1.32	0.06	21.47	0.00	0.00
ENSSSCG00000033641	COL8A2	15.87	1.32	0.48	2.72	0.01	0.01
ENSSSCG00000009519	CLYBL	130.95	1.32	0.18	7.50	0.00	0.00
ENSSSCG00000017734	RHBDL3	197.92	1.32	0.15	8.85	0.00	0.00
ENSSSCG00000006725	TBX15	286.29	1.32	0.12	10.69	0.00	0.00
ENSSSCG00000006087	CPQ	222.96	1.32	0.14	9.23	0.00	0.00
ENSSSCG00000017756	NLK	699.86	1.32	0.08	16.20	0.00	0.00
ENSSSCG00000031147	ACAP2	1628.43	1.32	0.07	18.10	0.00	0.00
ENSSSCG00000028530	TMEM52	370.58	1.32	0.11	11.69	0.00	0.00
ENSSSCG00000002305	EXD2	224.73	1.32	0.14	9.34	0.00	0.00
ENSSSCG00000003513	ECE1	912.17	1.32	0.07	18.07	0.00	0.00
ENSSSCG00000037755	NSMCE2	66.39	1.32	0.25	5.34	0.00	0.00
ENSSSCG00000004956	SKOR1	80.20	1.32	0.23	5.71	0.00	0.00
ENSSSCG00000013072	FADS2	3228.74	1.32	0.05	26.68	0.00	0.00
ENSSSCG00000038989		8624.84	1.32	0.05	25.78	0.00	0.00
ENSSSCG00000038187		1356.29	1.32	0.07	18.83	0.00	0.00
ENSSSCG00000005641	DNM1	32.84	1.32	0.34	3.91	0.00	0.00
ENSSSCG00000003133	GRIN2D	9.33	1.32	0.64	2.07	0.04	0.05
ENSSSCG00000011720	MBNL1	1821.99	1.32	0.07	18.40	0.00	0.00
ENSSSCG00000033649		11.78	1.32	0.56	2.36	0.02	0.03
ENSSSCG00000016090	SPATS2L	488.85	1.32	0.10	13.34	0.00	0.00
ENSSSCG00000017690	DHRS11	2472.94	1.32	0.05	24.54	0.00	0.00
ENSSSCG00000003670	RLF	1336.62	1.32	0.08	17.55	0.00	0.00
ENSSSCG00000017137	METRNL	267.93	1.32	0.13	10.10	0.00	0.00

ENSSSCG00000038283	KLK11	81.24	1.32	0.22	5.93	0.00	0.00
ENSSSCG00000016665	BMPER	112.86	1.32	0.19	6.84	0.00	0.00
ENSSSCG00000008456	PLEKHH2	233.16	1.32	0.13	9.98	0.00	0.00
ENSSSCG00000021068	TRAF5	394.95	1.32	0.11	12.45	0.00	0.00
ENSSSCG00000037671	PACS2	1216.64	1.32	0.08	16.37	0.00	0.00
ENSSSCG00000032552		1793.30	1.32	0.07	20.27	0.00	0.00
ENSSSCG00000029778	SPATA17	17.58	1.32	0.47	2.83	0.00	0.01
ENSSSCG00000035901	SPIN1	1152.17	1.32	0.07	18.30	0.00	0.00
ENSSSCG00000008867	CTSO	194.24	1.33	0.15	9.12	0.00	0.00
ENSSSCG00000030513	CFAP74	361.36	1.33	0.13	10.52	0.00	0.00
ENSSSCG00000005598	SCAI	132.57	1.33	0.17	7.59	0.00	0.00
ENSSSCG00000003439	DHRS3	840.90	1.33	0.08	16.99	0.00	0.00
ENSSSCG00000007288		490.30	1.33	0.09	14.18	0.00	0.00
ENSSSCG00000006743	SLC22A15	126.40	1.33	0.18	7.31	0.00	0.00
ENSSSCG00000026697	UHRF1BP1L	1183.15	1.33	0.08	17.08	0.00	0.00
ENSSSCG00000006267	PCMTD1	1182.63	1.33	0.07	19.36	0.00	0.00
ENSSSCG00000011592	PLXND1	1258.99	1.33	0.08	16.89	0.00	0.00
ENSSSCG00000012572	COL4A5	34.16	1.33	0.33	4.02	0.00	0.00
ENSSSCG00000014822	ARHGEF17	377.69	1.33	0.11	12.61	0.00	0.00
ENSSSCG00000032438	FFAR2	22.43	1.33	0.41	3.28	0.00	0.00
ENSSSCG00000010184	AGT	460.26	1.33	0.11	12.13	0.00	0.00
ENSSSCG00000023112	PDS5A	3211.17	1.33	0.05	25.25	0.00	0.00
ENSSSCG00000004017	FRMD1	32.38	1.33	0.35	3.81	0.00	0.00
ENSSSCG00000014168	ELL2	1030.14	1.33	0.08	16.83	0.00	0.00
ENSSSCG00000001549	FKBP5	2041.12	1.33	0.06	24.08	0.00	0.00

ENSSSCG00000005935	AGO2	972.80	1.34	0.08	16.69	0.00	0.00
ENSSSCG00000000090	CBX7	56.83	1.34	0.26	5.16	0.00	0.00
ENSSSCG00000011181	NEK11	44.48	1.34	0.30	4.44	0.00	0.00
ENSSSCG00000010801	CDC73	1163.69	1.34	0.07	19.76	0.00	0.00
ENSSSCG00000026417	EPS8L1	91.07	1.34	0.21	6.48	0.00	0.00
ENSSSCG00000016298	INPP5D	28.41	1.34	0.36	3.68	0.00	0.00
ENSSSCG00000012744	CD99L2	143.91	1.34	0.17	8.07	0.00	0.00
ENSSSCG00000021384	BRIP1	446.19	1.34	0.10	13.36	0.00	0.00
ENSSSCG00000039511		10.02	1.34	0.62	2.16	0.03	0.04
ENSSSCG00000036152	TSPAN15	202.01	1.34	0.14	9.44	0.00	0.00
ENSSSCG00000012985		2102.53	1.34	0.06	22.50	0.00	0.00
ENSSSCG00000008748	LCORL	343.34	1.34	0.11	11.82	0.00	0.00
ENSSSCG00000000259	CSAD	226.80	1.34	0.14	9.81	0.00	0.00
ENSSSCG00000014436	ARHGEF37	117.68	1.34	0.19	7.23	0.00	0.00
ENSSSCG00000017998	GLP2R	16.68	1.35	0.49	2.73	0.01	0.01
ENSSSCG00000007516	STX16	1517.15	1.35	0.06	21.30	0.00	0.00
ENSSSCG00000004869	CNDP1	150.09	1.35	0.16	8.27	0.00	0.00
ENSSSCG00000000006	PPARA	33.79	1.35	0.34	3.97	0.00	0.00
ENSSSCG00000010342	SH2D4B	18.03	1.35	0.49	2.77	0.01	0.01
ENSSSCG00000027688	RNF183	77.94	1.35	0.23	5.87	0.00	0.00
ENSSSCG00000013857	EPS15L1	550.39	1.35	0.09	14.51	0.00	0.00
ENSSSCG00000017296	ACE	160.22	1.35	0.16	8.59	0.00	0.00
ENSSSCG00000003917	TESK2	12.53	1.35	0.58	2.31	0.02	0.03
ENSSSCG00000001582	MDGA1	225.77	1.35	0.14	9.47	0.00	0.00
ENSSSCG00000001527	ILRUN	1719.27	1.35	0.07	20.63	0.00	0.00

ENSSSCG00000001689	GTPBP2	1314.42	1.35	0.07	19.10	0.00	0.00
ENSSSCG00000007940		72.86	1.35	0.24	5.62	0.00	0.00
ENSSSCG00000036534	MYOM3	281.00	1.35	0.12	11.00	0.00	0.00
ENSSSCG00000012295	MAGIX	91.80	1.35	0.22	6.29	0.00	0.00
ENSSSCG00000035357	BCL2L1	1453.66	1.36	0.07	20.13	0.00	0.00
ENSSSCG00000031356	HES1	1718.79	1.36	0.07	19.09	0.00	0.00
ENSSSCG00000040977	SNX8	1405.93	1.36	0.07	19.85	0.00	0.00
ENSSSCG00000004349		181.17	1.36	0.15	9.03	0.00	0.00
ENSSSCG00000006274	PRKDC	1261.72	1.36	0.07	18.35	0.00	0.00
ENSSSCG00000023585	SERINC2	1399.50	1.36	0.07	20.39	0.00	0.00
ENSSSCG00000035596		122.98	1.36	0.18	7.53	0.00	0.00
ENSSSCG00000012089	ADARB1	56.33	1.36	0.26	5.15	0.00	0.00
ENSSSCG00000034262	FIGN	95.42	1.36	0.20	6.75	0.00	0.00
ENSSSCG00000028758	LBP	18.06	1.36	0.47	2.89	0.00	0.01
ENSSSCG00000027628	IL6R	242.37	1.36	0.14	9.51	0.00	0.00
ENSSSCG00000033750		241.36	1.36	0.13	10.26	0.00	0.00
ENSSSCG00000015901	GRB14	78.09	1.36	0.24	5.73	0.00	0.00
ENSSSCG00000016117	CARF	52.98	1.36	0.27	5.03	0.00	0.00
ENSSSCG00000008986	SEPT11	1144.49	1.36	0.08	17.55	0.00	0.00
ENSSSCG00000012315	SYNJ2	482.16	1.36	0.10	13.28	0.00	0.00
ENSSSCG00000006115	RUNX1T1	130.15	1.36	0.18	7.75	0.00	0.00
ENSSSCG00000002414	SEL1L	4000.40	1.36	0.05	25.94	0.00	0.00
ENSSSCG00000029456	SLC7A1	1177.23	1.37	0.07	19.08	0.00	0.00
ENSSSCG00000003807	DNAJC6	661.48	1.37	0.09	15.80	0.00	0.00
ENSSSCG00000009951	SGSM1	1149.20	1.37	0.08	17.80	0.00	0.00

ENSSSCG00000027621	NDUFA4L2	58.21	1.37	0.26	5.26	0.00	0.00
ENSSSCG00000023334	BCLAF3	101.11	1.37	0.20	6.80	0.00	0.00
ENSSSCG00000013455	IZUMO4	45.03	1.37	0.30	4.51	0.00	0.00
ENSSSCG00000000779	KIF21A	669.33	1.37	0.09	15.78	0.00	0.00
ENSSSCG00000014081	COL4A3BP	313.49	1.37	0.12	11.59	0.00	0.00
ENSSSCG00000008534	TOGARAM2	144.62	1.37	0.17	8.15	0.00	0.00
ENSSSCG00000017630	EPX	14.37	1.37	0.52	2.62	0.01	0.01
ENSSSCG00000021975		75.32	1.37	0.23	5.92	0.00	0.00
ENSSSCG00000006525		482.79	1.37	0.10	13.21	0.00	0.00
ENSSSCG00000004136	AIG1	229.95	1.37	0.14	9.85	0.00	0.00
ENSSSCG00000034964	STX8	291.41	1.37	0.12	11.50	0.00	0.00
ENSSSCG00000007682	SH2B2	107.46	1.37	0.21	6.55	0.00	0.00
ENSSSCG00000027646	TIPARP	3391.09	1.37	0.06	23.55	0.00	0.00
ENSSSCG00000021738		780.64	1.37	0.08	16.86	0.00	0.00
ENSSSCG00000033949	GNG7	10.16	1.37	0.64	2.14	0.03	0.05
ENSSSCG00000030110		224.24	1.37	0.14	9.89	0.00	0.00
ENSSSCG00000034630	ABI2	488.19	1.37	0.10	13.93	0.00	0.00
ENSSSCG00000032558	EMX2	1541.31	1.37	0.06	21.94	0.00	0.00
ENSSSCG00000015342	COL28A1	35.01	1.37	0.34	4.00	0.00	0.00
ENSSSCG00000004595	ADAM10	1991.12	1.37	0.06	22.20	0.00	0.00
ENSSSCG00000014364	ANKHD1	2271.83	1.37	0.06	24.49	0.00	0.00
ENSSSCG00000000169	POLR3B	482.10	1.37	0.10	14.25	0.00	0.00
ENSSSCG00000011543	LHFPL4	237.26	1.37	0.13	10.55	0.00	0.00
ENSSSCG00000023611		452.73	1.37	0.10	13.37	0.00	0.00
ENSSSCG00000016846	WDR70	210.21	1.38	0.14	9.97	0.00	0.00

ENSSSCG00000011249	DLEC1	71.30	1.38	0.23	5.86	0.00	0.00
ENSSSCG00000011772		978.10	1.38	0.08	18.16	0.00	0.00
ENSSSCG00000010031		287.67	1.38	0.12	11.50	0.00	0.00
ENSSSCG00000012074		1931.10	1.38	0.06	22.69	0.00	0.00
ENSSSCG00000004663	SEMA6D	211.07	1.38	0.14	9.73	0.00	0.00
ENSSSCG00000009742	ULK1	1977.36	1.38	0.07	19.61	0.00	0.00
ENSSSCG00000023307	FBXW11	3046.66	1.38	0.05	26.09	0.00	0.00
ENSSSCG00000030694	PKP1	33.92	1.38	0.33	4.17	0.00	0.00
ENSSSCG00000037539	SORCS2	12.66	1.38	0.56	2.47	0.01	0.02
ENSSSCG00000024236	MMP17	14.56	1.38	0.51	2.73	0.01	0.01
ENSSSCG00000024399	EVC2	111.57	1.38	0.20	6.84	0.00	0.00
ENSSSCG00000029594	WBP1L	859.37	1.38	0.08	18.12	0.00	0.00
ENSSSCG00000005630	NAIF1	24.29	1.39	0.39	3.51	0.00	0.00
ENSSSCG00000006620	TUFT1	1643.31	1.39	0.07	21.03	0.00	0.00
ENSSSCG00000034090	CRTC3	664.27	1.39	0.09	14.77	0.00	0.00
ENSSSCG00000040838	AGBL2	505.10	1.39	0.10	13.93	0.00	0.00
ENSSSCG00000003591	PUM1	1901.32	1.39	0.06	24.02	0.00	0.00
ENSSSCG00000011700	CP	25.43	1.39	0.42	3.30	0.00	0.00
ENSSSCG00000037583	GNB4	929.57	1.39	0.08	17.56	0.00	0.00
ENSSSCG00000008122	ADRA2B	17.08	1.39	0.50	2.80	0.01	0.01
ENSSSCG00000007166	PTPRA	1387.90	1.39	0.06	21.73	0.00	0.00
ENSSSCG00000011236		831.24	1.39	0.08	17.58	0.00	0.00
ENSSSCG00000032456		22.59	1.39	0.43	3.23	0.00	0.00
ENSSSCG00000009249	HNRNPD	1762.02	1.39	0.07	19.72	0.00	0.00
ENSSSCG00000021206	IL1RAP	623.99	1.39	0.09	14.81	0.00	0.00

ENSSSCG00000011254	XYLB	343.33	1.39	0.11	12.26	0.00	0.00
ENSSSCG00000010878	EFCAB2	14.03	1.39	0.52	2.70	0.01	0.01
ENSSSCG00000007839	EEF2K	944.65	1.39	0.08	18.03	0.00	0.00
ENSSSCG00000037154	SFTA2	102.77	1.39	0.21	6.66	0.00	0.00
ENSSSCG00000009152	SGMS2	490.93	1.39	0.10	13.92	0.00	0.00
ENSSSCG00000011498	SLC25A26	96.15	1.39	0.21	6.72	0.00	0.00
ENSSSCG00000012136	PIR	38.47	1.39	0.32	4.33	0.00	0.00
ENSSSCG00000013337	BBOX1	26.15	1.39	0.38	3.63	0.00	0.00
ENSSSCG00000005277	GCNT1	50.64	1.39	0.28	5.01	0.00	0.00
ENSSSCG00000001064	GMPR	174.36	1.40	0.15	9.07	0.00	0.00
ENSSSCG00000012324	IQSEC2	341.62	1.40	0.11	12.44	0.00	0.00
ENSSSCG00000005027	FRMD6	2589.76	1.40	0.06	23.87	0.00	0.00
ENSSSCG00000023235	MAN1C1	31.79	1.40	0.35	3.96	0.00	0.00
ENSSSCG00000004717	UBR1	1170.73	1.40	0.08	18.45	0.00	0.00
ENSSSCG00000036556	IL10RB	980.22	1.40	0.08	18.58	0.00	0.00
ENSSSCG00000038977	RSBN1L	1178.84	1.40	0.07	18.66	0.00	0.00
ENSSSCG00000021885	MDFIC	972.96	1.40	0.07	19.23	0.00	0.00
ENSSSCG00000011516	EIF4E3	382.71	1.40	0.11	13.25	0.00	0.00
ENSSSCG00000000455	LRIG3	336.97	1.40	0.12	12.11	0.00	0.00
ENSSSCG00000015491		172.16	1.40	0.16	8.85	0.00	0.00
ENSSSCG00000004980	THSD4	23.72	1.40	0.42	3.33	0.00	0.00
ENSSSCG00000008192	TMEM131	3629.53	1.40	0.05	26.53	0.00	0.00
ENSSSCG00000036274		7759.77	1.40	0.06	23.20	0.00	0.00
ENSSSCG00000027975	LRRC3	30.50	1.40	0.36	3.93	0.00	0.00
ENSSSCG00000008029	BAIAP3	96.58	1.40	0.20	6.94	0.00	0.00

ENSSSCG00000029633	USP24	1974.51	1.40	0.06	24.61	0.00	0.00
ENSSSCG00000034978	PNPLA7	154.12	1.40	0.17	8.49	0.00	0.00
ENSSSCG00000016550	KLF14	64.88	1.40	0.25	5.67	0.00	0.00
ENSSSCG00000027722	BMP2K	847.75	1.40	0.08	17.67	0.00	0.00
ENSSSCG00000017637	MPO	8.58	1.40	0.66	2.12	0.03	0.05
ENSSSCG00000027348		341.71	1.40	0.11	12.57	0.00	0.00
ENSSSCG00000007944		212.66	1.40	0.14	10.10	0.00	0.00
ENSSSCG00000026041	MAP3K5	332.17	1.41	0.13	10.87	0.00	0.00
ENSSSCG00000015507	TNN	71.19	1.41	0.23	6.03	0.00	0.00
ENSSSCG00000039539	DHTKD1	346.94	1.41	0.11	12.65	0.00	0.00
ENSSSCG00000017091	TNIP1	2853.10	1.41	0.06	22.96	0.00	0.00
ENSSSCG00000032697	RF02271	22.76	1.41	0.41	3.41	0.00	0.00
ENSSSCG00000016557	CPA1	119.34	1.41	0.19	7.25	0.00	0.00
ENSSSCG00000012759	ZNF185	34.30	1.41	0.34	4.11	0.00	0.00
ENSSSCG00000032574	RTTN	387.19	1.41	0.11	12.34	0.00	0.00
ENSSSCG00000028185	FGD3	208.74	1.41	0.14	9.91	0.00	0.00
ENSSSCG00000009432	DGKD	481.41	1.41	0.11	13.13	0.00	0.00
ENSSSCG00000024938	SH3BP5	491.28	1.41	0.09	14.88	0.00	0.00
ENSSSCG00000009400	RCBTB2	715.20	1.41	0.09	16.39	0.00	0.00
ENSSSCG00000036835	OSBPL2	616.90	1.41	0.09	16.17	0.00	0.00
ENSSSCG00000024904	SEC22A	223.93	1.41	0.14	10.37	0.00	0.00
ENSSSCG00000006757	TRIM33	1323.63	1.41	0.07	19.47	0.00	0.00
ENSSSCG00000004149	NHSL1	2228.00	1.41	0.06	23.87	0.00	0.00
ENSSSCG00000005582	STRBP	1215.92	1.41	0.07	20.38	0.00	0.00
ENSSSCG00000004070	SCAF8	833.13	1.41	0.08	17.19	0.00	0.00

ENSSSCG00000006099	ESRP1	592.48	1.41	0.09	15.94	0.00	0.00
ENSSSCG00000009148	LEF1	1039.10	1.41	0.08	17.66	0.00	0.00
ENSSSCG00000002368	LTBP2	11.02	1.41	0.59	2.39	0.02	0.02
ENSSSCG00000027169	ENOSF1	31.34	1.41	0.35	4.04	0.00	0.00
ENSSSCG00000010278	CDH23	160.04	1.42	0.16	8.93	0.00	0.00
ENSSSCG00000008423	PPP1R21	653.41	1.42	0.09	16.52	0.00	0.00
ENSSSCG00000007507	PCK1	528.24	1.42	0.20	7.15	0.00	0.00
ENSSSCG00000023130	ASCC1	590.42	1.42	0.09	16.26	0.00	0.00
ENSSSCG00000023890	ATG2A	2230.98	1.42	0.06	25.27	0.00	0.00
ENSSSCG00000006303	DCAF6	2224.87	1.42	0.06	25.51	0.00	0.00
ENSSSCG00000013300	EHF	596.90	1.42	0.09	15.37	0.00	0.00
ENSSSCG00000031423	UPK3A	9.86	1.42	0.61	2.32	0.02	0.03
ENSSSCG00000008644	KIDINS220	2173.45	1.42	0.06	24.56	0.00	0.00
ENSSSCG00000037898	CXXC5	185.58	1.42	0.15	9.23	0.00	0.00
ENSSSCG00000026964	STK36	587.78	1.42	0.09	15.24	0.00	0.00
ENSSSCG00000009834	ATXN2	1855.96	1.42	0.06	25.01	0.00	0.00
ENSSSCG00000010102	SLC7A4	92.01	1.42	0.21	6.87	0.00	0.00
ENSSSCG00000022194		40.82	1.42	0.31	4.57	0.00	0.00
ENSSSCG00000016988	ERGIC1	1157.52	1.42	0.09	16.65	0.00	0.00
ENSSSCG00000031825	NAPRT	596.80	1.42	0.09	15.09	0.00	0.00
ENSSSCG00000006289	F5	505.60	1.43	0.10	14.76	0.00	0.00
ENSSSCG00000004968	PAQR5	675.21	1.43	0.09	16.72	0.00	0.00
ENSSSCG00000010701	BTBD16	9.96	1.43	0.63	2.26	0.02	0.03
ENSSSCG00000037418		8.71	1.43	0.66	2.15	0.03	0.04
ENSSSCG00000037391		56.85	1.43	0.27	5.36	0.00	0.00

ENSSSCG00000026001	DNAJC1	191.16	1.43	0.16	9.17	0.00	0.00
ENSSSCG00000021867	HPN	209.31	1.43	0.16	9.21	0.00	0.00
ENSSSCG00000011940	DZIP3	230.86	1.43	0.14	10.35	0.00	0.00
ENSSSCG00000014368		21.78	1.43	0.43	3.36	0.00	0.00
ENSSSCG00000009276	XPO4	314.99	1.43	0.12	12.08	0.00	0.00
ENSSSCG00000029442		18.65	1.43	0.47	3.05	0.00	0.00
ENSSSCG00000013664	C19orf66	168.04	1.43	0.17	8.48	0.00	0.00
ENSSSCG00000015379	DNAH11	420.84	1.43	0.12	11.97	0.00	0.00
ENSSSCG00000010485	TBC1D12	359.60	1.43	0.11	12.60	0.00	0.00
ENSSSCG00000028536	LHFPL2	1136.92	1.43	0.08	17.98	0.00	0.00
ENSSSCG00000038911	KLF2	273.62	1.43	0.13	11.31	0.00	0.00
ENSSSCG00000016417	RBM33	628.00	1.44	0.09	15.21	0.00	0.00
ENSSSCG00000030354	MAP3K2	702.53	1.44	0.10	15.09	0.00	0.00
ENSSSCG00000008451		694.62	1.44	0.09	16.35	0.00	0.00
ENSSSCG00000016625	CTTNBP2	692.58	1.44	0.08	17.42	0.00	0.00
ENSSSCG00000002640	DEF8	1253.46	1.44	0.07	20.86	0.00	0.00
ENSSSCG00000039767	TH	270.92	1.44	0.13	11.24	0.00	0.00
ENSSSCG00000010473	EXOC6	238.15	1.44	0.14	10.65	0.00	0.00
ENSSSCG00000015390		1040.69	1.44	0.07	19.53	0.00	0.00
ENSSSCG00000002523	CDC42BPB	1384.92	1.44	0.06	22.30	0.00	0.00
ENSSSCG00000007599	LMTK2	941.08	1.44	0.08	18.78	0.00	0.00
ENSSSCG00000014893		228.90	1.44	0.13	10.79	0.00	0.00
ENSSSCG00000009142	SEC24B	1176.49	1.44	0.07	20.38	0.00	0.00
ENSSSCG00000023728	TECTA	725.78	1.44	0.08	17.70	0.00	0.00
ENSSSCG00000003022	TMEM145	18.14	1.44	0.46	3.15	0.00	0.00

ENSSSCG00000017804	ABR	2566.52	1.44	0.05	27.31	0.00	0.00
ENSSSCG00000006693	PDZK1	2058.70	1.44	0.06	25.34	0.00	0.00
ENSSSCG00000015196	SIAE	326.73	1.45	0.12	12.34	0.00	0.00
ENSSSCG00000020835	EIF4EBP3	23.87	1.45	0.41	3.52	0.00	0.00
ENSSSCG00000026349	ALDH3B2	8.71	1.45	0.67	2.16	0.03	0.04
ENSSSCG00000036229		469.92	1.45	0.11	13.74	0.00	0.00
ENSSSCG00000012833		19.64	1.45	0.50	2.89	0.00	0.01
ENSSSCG00000012314	SERAC1	126.78	1.45	0.18	7.88	0.00	0.00
ENSSSCG00000002669	CRISPLD2	18.80	1.45	0.46	3.13	0.00	0.00
ENSSSCG00000028018	EP400	1944.40	1.45	0.06	24.22	0.00	0.00
ENSSSCG00000007098		470.59	1.45	0.10	13.86	0.00	0.00
ENSSSCG00000012642	STAG2	2463.61	1.45	0.07	21.14	0.00	0.00
ENSSSCG00000007514	VAPB	3652.29	1.45	0.05	27.85	0.00	0.00
ENSSSCG00000003401	KIF1B	2785.34	1.46	0.06	26.34	0.00	0.00
ENSSSCG00000008831	DCUN1D4	523.44	1.46	0.09	15.36	0.00	0.00
ENSSSCG00000033384		10.01	1.46	0.66	2.20	0.03	0.04
ENSSSCG00000017938	YBX2	80.48	1.46	0.22	6.67	0.00	0.00
ENSSSCG00000022479	UBR5	4445.95	1.46	0.05	28.62	0.00	0.00
ENSSSCG00000009794	MLXIP	1150.26	1.46	0.07	19.67	0.00	0.00
ENSSSCG00000004139	ADGRG6	2421.52	1.46	0.06	26.01	0.00	0.00
ENSSSCG00000011896	ADPRH	136.29	1.46	0.18	8.17	0.00	0.00
ENSSSCG00000017762	FOXN1	37.10	1.46	0.32	4.53	0.00	0.00
ENSSSCG00000024663		226.90	1.46	0.14	10.76	0.00	0.00
ENSSSCG00000011493		559.87	1.46	0.10	14.20	0.00	0.00
ENSSSCG00000015689	DARS	2370.39	1.46	0.06	23.73	0.00	0.00

ENSSSCG00000009229	ARHGAP24	94.67	1.46	0.21	6.89	0.00	0.00
ENSSSCG00000030378	LIMK1	172.32	1.46	0.15	9.63	0.00	0.00
ENSSSCG00000004856	NFATC1	37.84	1.47	0.32	4.53	0.00	0.00
ENSSSCG00000004460	IBTK	1119.94	1.47	0.09	16.88	0.00	0.00
ENSSSCG00000004753	INO80	475.10	1.47	0.10	15.04	0.00	0.00
ENSSSCG00000039867	UCK2	1150.02	1.47	0.08	18.07	0.00	0.00
ENSSSCG00000000612	ATF7IP	603.75	1.47	0.09	16.71	0.00	0.00
ENSSSCG00000011471	FLNB	9299.80	1.47	0.05	32.53	0.00	0.00
ENSSSCG00000008785	FAM114A1	736.43	1.48	0.08	17.57	0.00	0.00
ENSSSCG00000006201	ARFGEF1	2290.84	1.48	0.05	26.90	0.00	0.00
ENSSSCG00000015859	SAP130	1211.52	1.48	0.07	22.21	0.00	0.00
ENSSSCG00000012742	MTM1	406.82	1.48	0.10	14.15	0.00	0.00
ENSSSCG00000027777		469.21	1.48	0.10	14.90	0.00	0.00
ENSSSCG00000012283	ZNF81	49.84	1.48	0.29	5.19	0.00	0.00
ENSSSCG00000037663	PKNOX1	759.19	1.48	0.08	17.93	0.00	0.00
ENSSSCG00000014993		197.99	1.48	0.15	10.00	0.00	0.00
ENSSSCG00000003730	RNF138	595.19	1.48	0.09	15.71	0.00	0.00
ENSSSCG00000004616	ONECUT1	8.30	1.48	0.69	2.15	0.03	0.04
ENSSSCG00000039185	EPB41L5	991.78	1.48	0.07	20.03	0.00	0.00
ENSSSCG00000010277	SLC29A3	73.79	1.48	0.23	6.32	0.00	0.00
ENSSSCG00000034328		15.97	1.48	0.50	2.94	0.00	0.01
ENSSSCG00000038708		1885.80	1.48	0.06	24.70	0.00	0.00
ENSSSCG00000007435		1380.07	1.48	0.07	21.42	0.00	0.00
ENSSSCG00000033941	C2orf88	76.17	1.49	0.23	6.40	0.00	0.00
ENSSSCG00000031479		43.91	1.49	0.30	4.90	0.00	0.00

ENSSSCG00000029281	PCDHAC2	130.93	1.49	0.18	8.40	0.00	0.00
ENSSSCG00000010252	HKDC1	764.97	1.49	0.08	17.92	0.00	0.00
ENSSSCG00000004289	CEP162	476.92	1.49	0.10	14.28	0.00	0.00
ENSSSCG00000005136	IFNE	28.16	1.49	0.37	4.00	0.00	0.00
ENSSSCG00000020505	RF00478	7.66	1.49	0.74	2.02	0.04	0.06
ENSSSCG00000012676	MBNL3	101.81	1.49	0.20	7.31	0.00	0.00
ENSSSCG00000008787	KLHL5	308.46	1.49	0.12	12.31	0.00	0.00
ENSSSCG00000002755	NFAT5	2080.56	1.49	0.06	25.59	0.00	0.00
ENSSSCG00000029485	FBXO11	1402.32	1.49	0.08	19.69	0.00	0.00
ENSSSCG00000004608	DNAAF4	42.97	1.49	0.30	4.97	0.00	0.00
ENSSSCG00000001913	STOML1	78.80	1.49	0.23	6.63	0.00	0.00
ENSSSCG00000018019	ARHGAP44	22.41	1.49	0.41	3.61	0.00	0.00
ENSSSCG00000035895	JDP2	140.22	1.49	0.18	8.31	0.00	0.00
ENSSSCG00000035629	CA5A	73.07	1.49	0.24	6.35	0.00	0.00
ENSSSCG00000037561	ATP11A	2125.27	1.49	0.06	26.68	0.00	0.00
ENSSSCG00000038401	TRAM2	1349.17	1.49	0.07	22.72	0.00	0.00
ENSSSCG00000034310		75.08	1.50	0.24	6.30	0.00	0.00
ENSSSCG00000006455		102.79	1.50	0.21	7.12	0.00	0.00
ENSSSCG00000003968	RIMKLA	434.17	1.50	0.10	14.79	0.00	0.00
ENSSSCG00000006900	DIPK1A	185.21	1.50	0.16	9.65	0.00	0.00
ENSSSCG00000027525	DHCR24	2177.78	1.50	0.07	21.78	0.00	0.00
ENSSSCG00000040453		95.99	1.50	0.22	6.86	0.00	0.00
ENSSSCG00000005609	GARNL3	84.76	1.50	0.22	6.82	0.00	0.00
ENSSSCG00000016942	CWC27	260.00	1.50	0.13	11.43	0.00	0.00
ENSSSCG00000021354	CNOT4	673.61	1.50	0.09	17.23	0.00	0.00

ENSSSCG00000036423	CCDC50	742.32	1.50	0.09	17.35	0.00	0.00
ENSSSCG00000024998	PCDHA11	105.34	1.50	0.19	7.73	0.00	0.00
ENSSSCG00000011729		207.06	1.50	0.14	10.61	0.00	0.00
ENSSSCG00000014440	HMGXB3	733.22	1.50	0.08	18.14	0.00	0.00
ENSSSCG00000006901	EVI5	713.63	1.50	0.09	17.38	0.00	0.00
ENSSSCG00000022643	KATNAL2	126.56	1.50	0.18	8.21	0.00	0.00
ENSSSCG00000005309	FAM166B	35.43	1.50	0.33	4.53	0.00	0.00
ENSSSCG00000004802	AQR	857.28	1.51	0.08	19.52	0.00	0.00
ENSSSCG00000023080	TPP1	9417.72	1.51	0.04	34.82	0.00	0.00
ENSSSCG00000000493	FRS2	359.03	1.51	0.12	12.95	0.00	0.00
ENSSSCG00000010941		177.48	1.51	0.17	9.08	0.00	0.00
ENSSSCG00000037284	CCDC85C	449.82	1.51	0.10	14.37	0.00	0.00
ENSSSCG00000008575	ASXL2	1239.34	1.51	0.07	21.53	0.00	0.00
ENSSSCG00000039084		35.55	1.51	0.33	4.58	0.00	0.00
ENSSSCG00000013427	CIRBP	1375.57	1.51	0.07	20.85	0.00	0.00
ENSSSCG00000009431	DGKH	301.90	1.51	0.12	12.27	0.00	0.00
ENSSSCG00000032171	TCF20	1117.93	1.51	0.08	19.35	0.00	0.00
ENSSSCG00000002281		222.50	1.51	0.14	10.93	0.00	0.00
ENSSSCG00000016751	GCK	140.36	1.51	0.17	8.92	0.00	0.00
ENSSSCG00000011113	NUDT5	805.19	1.51	0.18	8.59	0.00	0.00
ENSSSCG00000039658		4906.30	1.51	0.05	30.94	0.00	0.00
ENSSSCG00000016553	COPG2	741.03	1.51	0.09	17.37	0.00	0.00
ENSSSCG00000011788	VPS8	711.79	1.51	0.09	17.16	0.00	0.00
ENSSSCG00000000029	SCUBE1	9.06	1.51	0.65	2.33	0.02	0.03
ENSSSCG00000026423	SLC23A1	720.45	1.51	0.09	17.53	0.00	0.00

ENSSSCG00000023788	TBC1D30	439.37	1.51	0.10	14.93	0.00	0.00
ENSSSCG00000011911	DRD3	17.39	1.51	0.49	3.12	0.00	0.00
ENSSSCG00000008164	MAP4K4	4366.17	1.51	0.05	30.75	0.00	0.00
ENSSSCG00000005588	NR5A1	10.41	1.51	0.62	2.44	0.01	0.02
ENSSSCG00000010351	CCSER2	1089.02	1.52	0.07	21.75	0.00	0.00
ENSSSCG00000001878	PTPN9	575.80	1.52	0.09	16.97	0.00	0.00
ENSSSCG00000022846	SLC4A3	71.92	1.52	0.24	6.40	0.00	0.00
ENSSSCG00000029267	TIGIT	12.96	1.52	0.56	2.73	0.01	0.01
ENSSSCG00000027557		502.53	1.52	0.10	15.74	0.00	0.00
ENSSSCG00000026499	NMT2	142.46	1.52	0.17	8.77	0.00	0.00
ENSSSCG00000038594	SDC4	7721.61	1.52	0.06	26.39	0.00	0.00
ENSSSCG00000001532	UHRF1BP1	582.57	1.52	0.09	16.73	0.00	0.00
ENSSSCG00000011858	ZNF148	668.91	1.52	0.08	17.86	0.00	0.00
ENSSSCG00000023376	PARD6G	7.15	1.52	0.73	2.07	0.04	0.05
ENSSSCG00000024058	SLC5A2	232.13	1.52	0.13	11.26	0.00	0.00
ENSSSCG00000017146		1986.36	1.52	0.06	24.30	0.00	0.00
ENSSSCG00000005090	MNAT1	371.05	1.52	0.11	13.74	0.00	0.00
ENSSSCG00000037121	KIAA0319L	542.04	1.52	0.09	16.68	0.00	0.00
ENSSSCG00000004057	SYTL3	9.16	1.52	0.66	2.30	0.02	0.03
ENSSSCG00000029855	LHFPL6	218.55	1.52	0.14	10.99	0.00	0.00
ENSSSCG00000036702	ACTN1	4244.86	1.52	0.05	32.44	0.00	0.00
ENSSSCG00000010276	UNC5B	840.91	1.53	0.08	18.67	0.00	0.00
ENSSSCG00000023192		20.26	1.53	0.44	3.45	0.00	0.00
ENSSSCG00000007797	ITGAL	399.22	1.53	0.12	13.23	0.00	0.00
ENSSSCG00000011813	P3H2	1030.59	1.53	0.07	20.88	0.00	0.00

ENSSSCG00000024623	USP25	755.82	1.53	0.08	18.06	0.00	0.00
ENSSSCG00000004087	CCDC170	261.95	1.53	0.13	12.17	0.00	0.00
ENSSSCG00000021238	STX1B	192.99	1.53	0.15	10.06	0.00	0.00
ENSSSCG00000000625	LRP6	799.43	1.53	0.09	17.10	0.00	0.00
ENSSSCG00000009551	ARHGEF7	655.36	1.53	0.09	17.03	0.00	0.00
ENSSSCG00000029598	FAM124A	52.44	1.53	0.27	5.59	0.00	0.00
ENSSSCG00000011482	C3orf67	179.18	1.53	0.16	9.73	0.00	0.00
ENSSSCG00000031767	TANGO6	139.45	1.53	0.17	8.98	0.00	0.00
ENSSSCG00000033585	DUSP23	84.24	1.53	0.22	6.99	0.00	0.00
ENSSSCG00000016733		20.94	1.54	0.43	3.55	0.00	0.00
ENSSSCG00000007317	DLGAP4	1569.19	1.54	0.07	23.30	0.00	0.00
ENSSSCG00000021669	MARK3	1190.85	1.54	0.07	21.53	0.00	0.00
ENSSSCG00000000985	EXOC2	722.84	1.54	0.08	18.68	0.00	0.00
ENSSSCG00000017261	ARSG	21.00	1.54	0.43	3.57	0.00	0.00
ENSSSCG00000015692	R3HDM1	1168.17	1.54	0.07	22.14	0.00	0.00
ENSSSCG00000005465	SUSD1	78.17	1.54	0.23	6.81	0.00	0.00
ENSSSCG00000020764	CABP2	225.80	1.54	0.14	10.96	0.00	0.00
ENSSSCG00000011278	TRAK1	359.78	1.54	0.11	13.67	0.00	0.00
ENSSSCG00000028672	GCKR	159.82	1.54	0.16	9.44	0.00	0.00
ENSSSCG00000004347	FBXL4	310.94	1.54	0.13	12.32	0.00	0.00
ENSSSCG00000021814	ZMYM2	1920.66	1.54	0.06	24.94	0.00	0.00
ENSSSCG00000038539		11.72	1.54	0.59	2.60	0.01	0.01
ENSSSCG00000039461		7.83	1.55	0.71	2.19	0.03	0.04
ENSSSCG00000024373	TRIP12	3315.03	1.55	0.06	27.06	0.00	0.00
ENSSSCG00000001493	PRIM2	315.78	1.55	0.12	12.99	0.00	0.00

ENSSSCG00000016159	CPS1	94.21	1.55	0.20	7.55	0.00	0.00
ENSSSCG00000030345	INPP5J	59.09	1.55	0.26	5.84	0.00	0.00
ENSSSCG00000040562	C8orf88	6.61	1.55	0.76	2.03	0.04	0.06
ENSSSCG00000036963	LRRC25	19.15	1.55	0.47	3.32	0.00	0.00
ENSSSCG00000011020	JCAD	99.57	1.55	0.20	7.79	0.00	0.00
ENSSSCG00000013043	MACROD1	31.53	1.55	0.36	4.31	0.00	0.00
ENSSSCG00000005514	RAB14	46.28	1.55	0.30	5.20	0.00	0.00
ENSSSCG00000000082	CACNA1I	15.82	1.55	0.49	3.16	0.00	0.00
ENSSSCG00000008190	INPP4A	311.25	1.55	0.12	12.93	0.00	0.00
ENSSSCG00000035454	B4GALT1	714.00	1.55	0.09	17.98	0.00	0.00
ENSSSCG00000007153	ATRN	2049.86	1.55	0.06	27.83	0.00	0.00
ENSSSCG00000012408	NHSL2	340.73	1.55	0.12	13.07	0.00	0.00
ENSSSCG00000012083	RIPK4	895.49	1.56	0.08	19.50	0.00	0.00
ENSSSCG00000000475	IRAK3	451.41	1.56	0.11	13.84	0.00	0.00
ENSSSCG00000033481	RF00026	130.56	1.56	0.19	8.12	0.00	0.00
ENSSSCG00000014190	FBXL17	53.17	1.56	0.29	5.37	0.00	0.00
ENSSSCG00000014170	CAST	5585.65	1.56	0.05	30.40	0.00	0.00
ENSSSCG00000011850		7539.03	1.56	0.05	30.86	0.00	0.00
ENSSSCG00000000061	PMM1	424.25	1.56	0.11	14.40	0.00	0.00
ENSSSCG00000001040	C6orf52	37.33	1.56	0.33	4.69	0.00	0.00
ENSSSCG00000039215	PHYHIPL	767.95	1.56	0.08	19.44	0.00	0.00
ENSSSCG00000034768	CYTH3	252.50	1.56	0.13	12.07	0.00	0.00
ENSSSCG00000031657		811.66	1.56	0.08	19.75	0.00	0.00
ENSSSCG00000003572	SYTL1	129.43	1.56	0.19	8.39	0.00	0.00
ENSSSCG00000009860	FBXW8	298.65	1.56	0.12	12.80	0.00	0.00

ENSSSCG00000014892	USP35	59.39	1.56	0.27	5.88	0.00	0.00
ENSSSCG00000040105	SLC34A3	53.28	1.56	0.28	5.57	0.00	0.00
ENSSSCG00000023522	TGM2	2031.64	1.57	0.06	24.53	0.00	0.00
ENSSSCG00000021072	EPG5	305.21	1.57	0.13	12.53	0.00	0.00
ENSSSCG00000017043	RNF145	1561.15	1.57	0.07	22.33	0.00	0.00
ENSSSCG00000036909	ERI3	195.82	1.57	0.14	10.87	0.00	0.00
ENSSSCG00000006948	MROH6	10.68	1.57	0.60	2.61	0.01	0.01
ENSSSCG00000014303	JADE2	784.09	1.57	0.08	19.59	0.00	0.00
ENSSSCG00000016448	KCNH2	44.61	1.57	0.30	5.23	0.00	0.00
ENSSSCG00000035230	PIEZO1	1903.21	1.57	0.06	26.87	0.00	0.00
ENSSSCG00000038421		18.63	1.57	0.46	3.43	0.00	0.00
ENSSSCG00000017927		14.09	1.57	0.56	2.79	0.01	0.01
ENSSSCG00000009300	WASF3	102.73	1.57	0.20	8.01	0.00	0.00
ENSSSCG00000012394	GJB1	227.61	1.57	0.14	11.64	0.00	0.00
ENSSSCG00000016943	ADAMTS6	40.72	1.57	0.31	5.00	0.00	0.00
ENSSSCG00000004684	SPG11	1722.29	1.57	0.07	21.52	0.00	0.00
ENSSSCG00000005638	LCN2	64.84	1.57	0.29	5.37	0.00	0.00
ENSSSCG00000033302		8.69	1.57	0.69	2.27	0.02	0.03
ENSSSCG00000000587	AEBP2	384.08	1.58	0.11	14.51	0.00	0.00
ENSSSCG00000015557	NMNAT2	72.84	1.58	0.24	6.69	0.00	0.00
ENSSSCG00000014038	FAM193B	730.29	1.58	0.09	17.48	0.00	0.00
ENSSSCG00000011075	KIAA1217	971.89	1.58	0.08	20.83	0.00	0.00
ENSSSCG00000006495	SEMA4A	50.29	1.58	0.28	5.55	0.00	0.00
ENSSSCG00000017676	BCAS3	34.75	1.58	0.34	4.70	0.00	0.00
ENSSSCG00000009166	MANBA	1879.94	1.58	0.06	25.33	0.00	0.00

ENSSSCG00000017917	MINK1	4522.64	1.58	0.05	32.04	0.00	0.00
ENSSSCG00000003733	KLHL14	199.94	1.58	0.16	10.14	0.00	0.00
ENSSSCG00000006461	ARHGEF11	834.92	1.58	0.10	15.96	0.00	0.00
ENSSSCG00000003366	CHD5	78.96	1.58	0.23	6.95	0.00	0.00
ENSSSCG00000012440	PGK1	26210.65	1.58	0.05	34.63	0.00	0.00
ENSSSCG00000014269	FNIP1	1219.93	1.58	0.09	18.28	0.00	0.00
ENSSSCG00000020363	RF00100	19.52	1.58	0.46	3.45	0.00	0.00
ENSSSCG00000002931	ZNF565	15.44	1.58	0.50	3.16	0.00	0.00
ENSSSCG00000029507	RASGEF1B	288.98	1.58	0.12	12.82	0.00	0.00
ENSSSCG00000038207	ssc-mir-503	19.41	1.58	0.46	3.45	0.00	0.00
ENSSSCG00000008443	EPAS1	1017.55	1.59	0.07	22.16	0.00	0.00
ENSSSCG00000010253	HK1	8998.53	1.59	0.05	31.71	0.00	0.00
ENSSSCG00000037905	ITGB5	434.80	1.59	0.10	15.24	0.00	0.00
ENSSSCG00000030460	ARHGEF6	30.20	1.59	0.37	4.31	0.00	0.00
ENSSSCG00000011704	WWTR1	343.34	1.59	0.11	14.04	0.00	0.00
ENSSSCG00000014578	DENND5A	1361.50	1.59	0.07	21.54	0.00	0.00
ENSSSCG00000012084	PRDM15	204.39	1.59	0.15	10.53	0.00	0.00
ENSSSCG00000012251	USP9X	2650.98	1.59	0.07	23.50	0.00	0.00
ENSSSCG00000002965	ACTN4	7573.48	1.59	0.05	34.41	0.00	0.00
ENSSSCG00000021890	GNB1	5210.96	1.59	0.05	34.52	0.00	0.00
ENSSSCG00000031267	NIPAL2	111.59	1.59	0.20	8.00	0.00	0.00
ENSSSCG00000035479	DISP1	89.56	1.59	0.22	7.33	0.00	0.00
ENSSSCG00000004029	QKI	1452.23	1.59	0.07	23.26	0.00	0.00
ENSSSCG00000040735	DDAH1	2015.01	1.59	0.06	26.87	0.00	0.00
ENSSSCG00000001486	LRRC1	124.71	1.59	0.18	8.74	0.00	0.00

ENSSSCG00000005753	CAMSAP1	1140.07	1.59	0.07	23.47	0.00	0.00
ENSSSCG00000025672		722.20	1.59	0.09	18.41	0.00	0.00
ENSSSCG00000004859	ZNF516	405.60	1.59	0.11	13.93	0.00	0.00
ENSSSCG00000025704	DENND2D	221.11	1.59	0.14	11.12	0.00	0.00
ENSSSCG00000009035	SLC10A7	68.27	1.59	0.24	6.56	0.00	0.00
ENSSSCG00000027459	RNF13	1074.28	1.60	0.08	21.14	0.00	0.00
ENSSSCG00000028197	PFKL	6596.77	1.60	0.05	34.18	0.00	0.00
ENSSSCG00000001991	DHRS1	1242.84	1.60	0.07	23.72	0.00	0.00
ENSSSCG00000011882	GOLGB1	2654.49	1.60	0.06	26.40	0.00	0.00
ENSSSCG00000034757	FGF11	190.33	1.60	0.15	10.57	0.00	0.00
ENSSSCG00000003214	KCNC3	187.71	1.60	0.16	10.29	0.00	0.00
ENSSSCG00000011198	RFTN1	8.08	1.60	0.71	2.27	0.02	0.03
ENSSSCG00000008376	COMMD1	102.22	1.60	0.20	8.08	0.00	0.00
ENSSSCG00000030957	NFKB1	1143.06	1.60	0.07	22.29	0.00	0.00
ENSSSCG00000003772		903.07	1.60	0.08	20.49	0.00	0.00
ENSSSCG00000032761	LAMA5	18589.41	1.60	0.04	37.70	0.00	0.00
ENSSSCG00000037893		14.20	1.60	0.53	3.02	0.00	0.00
ENSSSCG00000016322	ACKR3	3055.75	1.60	0.13	12.38	0.00	0.00
ENSSSCG00000016531		110.12	1.60	0.19	8.33	0.00	0.00
ENSSSCG00000038558	GOLPH3L	1679.52	1.61	0.06	25.60	0.00	0.00
ENSSSCG00000018016	MAP2K4	1076.90	1.61	0.07	21.83	0.00	0.00
ENSSSCG00000021749	MCF2L2	61.05	1.61	0.26	6.30	0.00	0.00
ENSSSCG00000011069	ARMC4	167.11	1.61	0.16	10.33	0.00	0.00
ENSSSCG00000032803	CYP2S1	1983.82	1.61	0.07	22.66	0.00	0.00
ENSSSCG00000013875	USE1	76.02	1.61	0.23	6.88	0.00	0.00

ENSSSCG00000030502	CEP170	409.95	1.61	0.10	15.56	0.00	0.00
ENSSSCG00000028382		20.52	1.61	0.45	3.60	0.00	0.00
ENSSSCG00000004718	TTBK2	372.96	1.61	0.11	14.58	0.00	0.00
ENSSSCG00000010826	MARC2	11.51	1.61	0.59	2.73	0.01	0.01
ENSSSCG00000037754	SLC39A11	70.94	1.61	0.24	6.73	0.00	0.00
ENSSSCG00000002424		208.17	1.61	0.15	10.67	0.00	0.00
ENSSSCG00000036136	BHLHE40	11420.65	1.61	0.06	28.74	0.00	0.00
ENSSSCG00000003433	PLOD1	6169.91	1.61	0.05	32.97	0.00	0.00
ENSSSCG00000031703		226.93	1.61	0.15	11.01	0.00	0.00
ENSSSCG00000016674	MINDY4	360.47	1.61	0.11	14.63	0.00	0.00
ENSSSCG00000016885	ITGA1	342.84	1.62	0.12	13.55	0.00	0.00
ENSSSCG00000032741	TBC1D9	441.93	1.62	0.11	15.27	0.00	0.00
ENSSSCG00000006988	PDGFRL	329.01	1.62	0.12	13.84	0.00	0.00
ENSSSCG00000037158	MTURN	342.64	1.62	0.11	14.38	0.00	0.00
ENSSSCG00000015008	C11orf65	21.11	1.62	0.45	3.59	0.00	0.00
ENSSSCG00000025590		12.35	1.62	0.57	2.82	0.00	0.01
ENSSSCG00000022236	FOLR1	34.82	1.62	0.34	4.80	0.00	0.00
ENSSSCG00000016412	LMBR1	336.29	1.62	0.12	13.86	0.00	0.00
ENSSSCG00000009213		10.99	1.62	0.62	2.61	0.01	0.01
ENSSSCG00000036305		616.04	1.62	0.09	17.49	0.00	0.00
ENSSSCG00000012489	TRMT2B	242.05	1.62	0.14	11.82	0.00	0.00
ENSSSCG00000022553	TNRC18	505.45	1.62	0.10	17.04	0.00	0.00
ENSSSCG00000038731	STX18	202.28	1.62	0.15	10.68	0.00	0.00
ENSSSCG00000011775	KLHL24	891.61	1.62	0.09	17.47	0.00	0.00
ENSSSCG00000005459	ECPAS	3062.13	1.62	0.06	28.58	0.00	0.00

ENSSSCG00000020743	RABGAP1	898.96	1.62	0.08	20.26	0.00	0.00
ENSSSCG00000002525	TRAF3	902.48	1.62	0.08	21.55	0.00	0.00
ENSSSCG00000008753	ADGRA3	369.28	1.62	0.11	14.33	0.00	0.00
ENSSSCG00000035029	NOVA2	9.57	1.62	0.68	2.37	0.02	0.03
ENSSSCG00000005613	LRSAM1	207.56	1.62	0.14	11.39	0.00	0.00
ENSSSCG00000034357	DAG1	5048.00	1.62	0.05	34.56	0.00	0.00
ENSSSCG00000002134	RNASE10	6.15	1.62	0.81	2.01	0.04	0.06
ENSSSCG00000029231		8.24	1.63	0.71	2.30	0.02	0.03
ENSSSCG00000016894	ARL15	9.60	1.63	0.66	2.47	0.01	0.02
ENSSSCG00000008713	KIAA0232	410.70	1.63	0.11	14.72	0.00	0.00
ENSSSCG00000003204	VRK3	432.93	1.63	0.10	15.61	0.00	0.00
ENSSSCG00000040183	CDK6	725.92	1.63	0.08	19.89	0.00	0.00
ENSSSCG00000016882	PARP8	685.19	1.63	0.08	19.44	0.00	0.00
ENSSSCG00000039688		189.86	1.63	0.15	10.99	0.00	0.00
ENSSSCG00000040401	EEF1A2	10843.15	1.63	0.04	37.93	0.00	0.00
ENSSSCG00000002834		9.63	1.63	0.65	2.53	0.01	0.02
ENSSSCG00000035281	ZNF292	2414.24	1.63	0.07	22.15	0.00	0.00
ENSSSCG00000009162		61.14	1.63	0.26	6.16	0.00	0.00
ENSSSCG00000016918	MAP3K1	1641.11	1.63	0.08	21.75	0.00	0.00
ENSSSCG00000012633	IL13RA1	4899.93	1.63	0.05	33.03	0.00	0.00
ENSSSCG00000015273	ATP2B4	3100.30	1.63	0.05	30.74	0.00	0.00
ENSSSCG00000011888	GPR156	16.55	1.63	0.49	3.33	0.00	0.00
ENSSSCG00000003119	ZNF541	47.63	1.63	0.29	5.64	0.00	0.00
ENSSSCG00000016501	KDM7A	967.11	1.64	0.10	16.83	0.00	0.00
ENSSSCG00000016147	PIKFYVE	1019.80	1.64	0.08	21.31	0.00	0.00

ENSSSCG00000008124	NEURL3	240.69	1.64	0.15	11.04	0.00	0.00
ENSSSCG00000021706	KIF21B	726.11	1.64	0.09	17.77	0.00	0.00
ENSSSCG00000010313	VCL	4765.57	1.64	0.05	31.34	0.00	0.00
ENSSSCG00000008467	EML4	1602.83	1.64	0.07	23.24	0.00	0.00
ENSSSCG00000011672	RASA2	718.18	1.64	0.09	18.70	0.00	0.00
ENSSSCG00000033327	PDGFB	1605.79	1.64	0.07	24.70	0.00	0.00
ENSSSCG00000032176	SMURF2	1077.61	1.64	0.08	19.98	0.00	0.00
ENSSSCG00000039609	ANKRD33B	673.17	1.64	0.09	18.67	0.00	0.00
ENSSSCG00000036634	MAP4K5	1308.95	1.64	0.07	24.76	0.00	0.00
ENSSSCG00000024223	ARHGEF16	1391.44	1.64	0.07	24.58	0.00	0.00
ENSSSCG00000001966	ARHGAP5	1091.64	1.64	0.09	19.11	0.00	0.00
ENSSSCG00000012971	EFEMP2	226.66	1.64	0.14	11.85	0.00	0.00
ENSSSCG00000017301	TLK2	413.33	1.65	0.10	15.85	0.00	0.00
ENSSSCG00000033493	CACNA1B	9.69	1.65	0.63	2.61	0.01	0.01
ENSSSCG00000002311	SUSD6	952.37	1.65	0.08	20.28	0.00	0.00
ENSSSCG00000033777	GPR152	26.44	1.65	0.39	4.18	0.00	0.00
ENSSSCG00000014443	CAMK2A	463.74	1.65	0.10	15.90	0.00	0.00
ENSSSCG00000024668	KLK1	6.27	1.65	0.79	2.10	0.04	0.05
ENSSSCG00000002957	GGN	42.61	1.65	0.31	5.34	0.00	0.00
ENSSSCG00000014240	CSNK1G3	444.49	1.65	0.10	16.28	0.00	0.00
ENSSSCG00000015888	RBMS1	992.09	1.65	0.07	22.67	0.00	0.00
ENSSSCG00000010566	FBXW4	141.60	1.65	0.34	4.80	0.00	0.00
ENSSSCG00000016453	TCAF1	950.88	1.65	0.08	21.67	0.00	0.00
ENSSSCG00000016248	AGFG1	3010.67	1.66	0.05	30.43	0.00	0.00
ENSSSCG00000039684	PGPEP1	623.20	1.66	0.09	17.75	0.00	0.00

ENSSSCG00000038549	ZFP36L2	5608.65	1.66	0.05	35.70	0.00	0.00
ENSSSCG00000001972	AP4S1	36.38	1.66	0.36	4.63	0.00	0.00
ENSSSCG00000035887	C8orf34	13.94	1.66	0.53	3.13	0.00	0.00
ENSSSCG00000010922	ELF3	5131.91	1.66	0.05	32.88	0.00	0.00
ENSSSCG00000032740	TMEM106B	1229.88	1.66	0.07	23.50	0.00	0.00
ENSSSCG00000039616	ZBTB25	485.76	1.66	0.10	16.32	0.00	0.00
ENSSSCG00000015862	LIMS2	279.82	1.66	0.13	13.05	0.00	0.00
ENSSSCG00000024379	PCSK4	10.45	1.66	0.63	2.64	0.01	0.01
ENSSSCG00000027153	WARS2	250.16	1.66	0.14	12.27	0.00	0.00
ENSSSCG00000015825	ADGRA2	9.77	1.66	0.63	2.62	0.01	0.01
ENSSSCG00000007314		4977.25	1.66	0.05	36.72	0.00	0.00
ENSSSCG00000023188	ARIH1	1403.54	1.66	0.07	24.54	0.00	0.00
ENSSSCG00000011576	HRH1	87.39	1.66	0.22	7.40	0.00	0.00
ENSSSCG00000001968	NUBPL	173.27	1.66	0.16	10.42	0.00	0.00
ENSSSCG00000031307		8.40	1.66	0.68	2.45	0.01	0.02
ENSSSCG00000004558	CSNK1G1	504.36	1.66	0.10	17.33	0.00	0.00
ENSSSCG00000033385		1800.40	1.67	0.06	27.82	0.00	0.00
ENSSSCG00000035657	DIP2A	249.19	1.67	0.14	11.92	0.00	0.00
ENSSSCG00000040035		21.83	1.67	0.47	3.54	0.00	0.00
ENSSSCG00000011652	STAG1	133.51	1.67	0.19	8.93	0.00	0.00
ENSSSCG00000011042	RSU1	695.22	1.67	0.09	18.35	0.00	0.00
ENSSSCG00000014379	PCDHB2	35.19	1.67	0.34	4.93	0.00	0.00
ENSSSCG00000008789	WDR19	332.15	1.67	0.12	14.39	0.00	0.00
ENSSSCG00000010699	ATE1	360.10	1.67	0.11	15.09	0.00	0.00
ENSSSCG00000008504	CRIM1	1081.17	1.67	0.08	22.13	0.00	0.00

ENSSSCG00000000078	TNRC6B	698.64	1.67	0.09	17.86	0.00	0.00
ENSSSCG00000021822	RNF169	535.48	1.67	0.10	17.58	0.00	0.00
ENSSSCG00000004854	TJP1	4339.93	1.67	0.05	36.07	0.00	0.00
ENSSSCG00000010440		1322.98	1.67	0.07	23.84	0.00	0.00
ENSSSCG00000004364	HACE1	586.87	1.67	0.10	17.54	0.00	0.00
ENSSSCG00000024189	SIL1	261.10	1.67	0.13	12.85	0.00	0.00
ENSSSCG00000032517	DMXL2	592.03	1.67	0.09	18.00	0.00	0.00
ENSSSCG00000013461	PEAK3	38.87	1.67	0.33	5.06	0.00	0.00
ENSSSCG00000012203	TAB3	260.05	1.67	0.13	12.87	0.00	0.00
ENSSSCG00000001421	ZBTB12	32.33	1.67	0.37	4.53	0.00	0.00
ENSSSCG00000023141	RNLS	9.98	1.67	0.66	2.52	0.01	0.02
ENSSSCG00000002656	ZCCHC14	850.21	1.67	0.08	20.89	0.00	0.00
ENSSSCG00000004598		32.31	1.68	0.38	4.46	0.00	0.00
ENSSSCG00000035455	FAM219A	543.30	1.68	0.11	15.17	0.00	0.00
ENSSSCG00000040392		29.00	1.68	0.38	4.43	0.00	0.00
ENSSSCG00000011226	TGFBR2	2984.27	1.68	0.05	31.58	0.00	0.00
ENSSSCG00000028635		118.12	1.68	0.19	8.77	0.00	0.00
ENSSSCG00000001422	C2	192.12	1.68	0.15	11.26	0.00	0.00
ENSSSCG00000016290	EFHD1	88.25	1.68	0.22	7.55	0.00	0.00
ENSSSCG00000030325	C1QTNF6	510.84	1.68	0.10	17.56	0.00	0.00
ENSSSCG00000016270	C2orf72	45.92	1.68	0.30	5.57	0.00	0.00
ENSSSCG00000034614		191.39	1.68	0.15	10.89	0.00	0.00
ENSSSCG00000026559	EML6	9.24	1.69	0.65	2.58	0.01	0.01
ENSSSCG00000007547	ADAP1	169.97	1.69	0.16	10.83	0.00	0.00
ENSSSCG00000040199	AFG1L	92.80	1.69	0.23	7.44	0.00	0.00

ENSSSCG00000007311	PHF20	1303.05	1.69	0.08	22.22	0.00	0.00
ENSSSCG00000011158	PFKP	3626.25	1.69	0.05	33.89	0.00	0.00
ENSSSCG00000010974	CNTFR	14.26	1.69	0.53	3.17	0.00	0.00
ENSSSCG00000023929	TXNRD3	273.74	1.69	0.14	12.43	0.00	0.00
ENSSSCG00000027365	WNT7B	1130.33	1.69	0.07	24.41	0.00	0.00
ENSSSCG00000027447	TMTC1	222.60	1.69	0.14	11.85	0.00	0.00
ENSSSCG00000006307	RCSD1	185.94	1.69	0.16	10.37	0.00	0.00
ENSSSCG00000039723		9.19	1.69	0.66	2.57	0.01	0.02
ENSSSCG00000009015	GATB	425.32	1.69	0.11	14.85	0.00	0.00
ENSSSCG00000031462	ZNRF1	233.42	1.69	0.15	11.47	0.00	0.00
ENSSSCG00000016437	WDR86	14.22	1.69	0.55	3.10	0.00	0.00
ENSSSCG00000008449	SLC3A1	1546.06	1.70	0.07	25.51	0.00	0.00
ENSSSCG00000010068		91.18	1.70	0.21	7.93	0.00	0.00
ENSSSCG00000016564	UBE2H	2421.86	1.70	0.06	28.41	0.00	0.00
ENSSSCG00000021342	QRICH2	73.49	1.70	0.24	7.16	0.00	0.00
ENSSSCG00000006338	DDR2	1138.24	1.70	0.08	22.41	0.00	0.00
ENSSSCG00000033819		89.96	1.70	0.22	7.83	0.00	0.00
ENSSSCG00000013004	TM7SF2	21.39	1.70	0.44	3.90	0.00	0.00
ENSSSCG00000003705	CABLES1	75.81	1.70	0.23	7.35	0.00	0.00
ENSSSCG00000023408	SAMD4A	454.34	1.70	0.10	16.36	0.00	0.00
ENSSSCG00000025306	PAK3	113.59	1.70	0.19	8.89	0.00	0.00
ENSSSCG00000028983	TBC1D1	1213.65	1.71	0.07	23.14	0.00	0.00
ENSSSCG00000013568		868.96	1.71	0.08	21.98	0.00	0.00
ENSSSCG00000000906		448.19	1.71	0.11	15.73	0.00	0.00
ENSSSCG00000007576	FOXK1	927.13	1.71	0.08	22.28	0.00	0.00

ENSSSCG00000016704	HOXA4	31.52	1.71	0.39	4.42	0.00	0.00
ENSSSCG00000033591	CHD9	1285.53	1.71	0.07	22.87	0.00	0.00
ENSSSCG00000010559		115.85	1.71	0.21	8.28	0.00	0.00
ENSSSCG00000025529	FAM3D	54.55	1.71	0.28	6.11	0.00	0.00
ENSSSCG00000005287	PSAT1	1899.41	1.71	0.07	25.54	0.00	0.00
ENSSSCG00000016792	RETREG1	401.16	1.71	0.11	15.77	0.00	0.00
ENSSSCG00000032049	ZZEF1	926.21	1.71	0.08	22.59	0.00	0.00
ENSSSCG00000010054	ADORA2A	110.16	1.71	0.20	8.46	0.00	0.00
ENSSSCG00000012123	RAB9A	1194.67	1.71	0.07	24.03	0.00	0.00
ENSSSCG00000028627		7.90	1.71	0.70	2.44	0.01	0.02
ENSSSCG00000014039	RGS14	311.11	1.71	0.13	13.55	0.00	0.00
ENSSSCG00000002350	ELMSAN1	597.97	1.71	0.10	17.30	0.00	0.00
ENSSSCG00000017790	TAOK1	1459.38	1.71	0.07	23.29	0.00	0.00
ENSSSCG00000005740	SARDH	191.26	1.71	0.15	11.43	0.00	0.00
ENSSSCG00000040617	TNFAIP8	265.45	1.71	0.13	12.96	0.00	0.00
ENSSSCG00000009698	GLRA3	150.85	1.71	0.17	10.26	0.00	0.00
ENSSSCG00000011511	FRMD4B	432.37	1.71	0.11	15.50	0.00	0.00
ENSSSCG00000011456	CHDH	168.17	1.71	0.16	10.80	0.00	0.00
ENSSSCG00000006069	RGS22	9.36	1.71	0.65	2.62	0.01	0.01
ENSSSCG00000039854		158.01	1.72	0.17	10.22	0.00	0.00
ENSSSCG00000010850		233.94	1.72	0.14	12.21	0.00	0.00
ENSSSCG00000009458		25.17	1.72	0.40	4.32	0.00	0.00
ENSSSCG00000039753		34.58	1.72	0.35	4.89	0.00	0.00
ENSSSCG00000038081	AGPAT3	747.84	1.72	0.09	19.42	0.00	0.00
ENSSSCG00000009998	CASTOR1	108.77	1.72	0.20	8.45	0.00	0.00

ENSSSCG00000004644	HDC	249.36	1.72	0.14	12.66	0.00	0.00
ENSSSCG00000026471	PCDHA1	18.72	1.72	0.46	3.75	0.00	0.00
ENSSSCG00000032451		7.19	1.72	0.74	2.31	0.02	0.03
ENSSSCG00000016549	MKLN1	729.44	1.72	0.10	17.70	0.00	0.00
ENSSSCG00000036801	C6orf132	1568.20	1.72	0.08	22.24	0.00	0.00
ENSSSCG00000003812	PGM1	1623.46	1.72	0.06	27.96	0.00	0.00
ENSSSCG00000008368	UGP2	2234.06	1.72	0.06	28.99	0.00	0.00
ENSSSCG00000024614	ITSN1	705.28	1.72	0.09	20.05	0.00	0.00
ENSSSCG00000013618	EPOR	278.41	1.73	0.13	13.52	0.00	0.00
ENSSSCG00000010650	AFAP1L2	68.90	1.73	0.25	6.91	0.00	0.00
ENSSSCG00000015926	ABCB11	13.75	1.73	0.55	3.15	0.00	0.00
ENSSSCG00000012332	WNK3	548.58	1.73	0.09	18.61	0.00	0.00
ENSSSCG00000014876	MYO7A	642.30	1.73	0.09	19.59	0.00	0.00
ENSSSCG00000000804	ANO6	1093.40	1.73	0.07	24.18	0.00	0.00
ENSSSCG00000034746	CAPN8	124.68	1.73	0.18	9.45	0.00	0.00
ENSSSCG00000035593		121.98	1.73	0.19	8.97	0.00	0.00
ENSSSCG00000004199	EPB41L2	1389.73	1.73	0.06	26.96	0.00	0.00
ENSSSCG00000025984	TTLL5	465.31	1.73	0.11	15.85	0.00	0.00
ENSSSCG00000029741	RALA	1168.93	1.73	0.07	24.66	0.00	0.00
ENSSSCG00000025052	BPIFB6	91.74	1.73	0.23	7.53	0.00	0.00
ENSSSCG00000011791	MAP3K13	735.50	1.73	0.09	19.42	0.00	0.00
ENSSSCG00000011074	ARHGAP21	873.65	1.74	0.08	22.45	0.00	0.00
ENSSSCG00000004943	MAP2K1	2092.03	1.74	0.06	26.86	0.00	0.00
ENSSSCG00000039049	MAP7	1217.19	1.74	0.07	25.61	0.00	0.00
ENSSSCG00000016052	MFSD6	1419.59	1.74	0.07	25.53	0.00	0.00

ENSSSCG00000035810		34.20	1.74	0.34	5.10	0.00	0.00
ENSSSCG00000007681	CUX1	332.95	1.74	0.11	15.13	0.00	0.00
ENSSSCG00000003234		6.53	1.74	0.82	2.12	0.03	0.05
ENSSSCG00000007000	FAT1	24886.08	1.74	0.04	38.80	0.00	0.00
ENSSSCG00000005924	MROH1	938.14	1.74	0.07	23.41	0.00	0.00
ENSSSCG00000023204	AXIN1	606.54	1.74	0.09	18.99	0.00	0.00
ENSSSCG00000004825	CHSY1	900.14	1.74	0.09	19.93	0.00	0.00
ENSSSCG00000016520	CREB3L2	705.09	1.74	0.08	20.64	0.00	0.00
ENSSSCG00000007744	PHKG1	13.88	1.74	0.53	3.27	0.00	0.00
ENSSSCG00000038135		94.85	1.74	0.21	8.33	0.00	0.00
ENSSSCG00000031329	ST8SIA1	5.09	1.75	0.88	1.98	0.05	0.06
ENSSSCG00000003107	ARHGAP35	2752.86	1.75	0.05	31.81	0.00	0.00
ENSSSCG00000028144	EPHX3	20.54	1.75	0.44	3.95	0.00	0.00
ENSSSCG00000015432	ATXN7L1	437.58	1.75	0.10	16.87	0.00	0.00
ENSSSCG00000003642	INPP5B	601.59	1.75	0.09	19.40	0.00	0.00
ENSSSCG00000001646	BICRAL	289.79	1.75	0.13	13.43	0.00	0.00
ENSSSCG00000016409	UBE3C	1493.41	1.75	0.06	27.62	0.00	0.00
ENSSSCG00000009943	SSH1	848.21	1.75	0.08	21.65	0.00	0.00
ENSSSCG00000003212	NAPSA	6.61	1.75	0.80	2.20	0.03	0.04
ENSSSCG00000012463	CHM	664.30	1.76	0.09	18.85	0.00	0.00
ENSSSCG00000031417		16.82	1.76	0.50	3.54	0.00	0.00
ENSSSCG00000008392	BCL11A	67.73	1.76	0.25	7.05	0.00	0.00
ENSSSCG00000012857	CARS	759.23	1.76	0.09	20.40	0.00	0.00
ENSSSCG00000035045	DOCK1	653.38	1.76	0.09	18.83	0.00	0.00
ENSSSCG00000001780	FAH	581.13	1.76	0.09	18.75	0.00	0.00

ENSSSCG00000012059	HLCS	193.12	1.76	0.16	10.84	0.00	0.00
ENSSSCG00000017728	MYO1D	867.65	1.76	0.08	22.70	0.00	0.00
ENSSSCG00000006508	ASH1L	2768.54	1.76	0.06	27.49	0.00	0.00
ENSSSCG00000011795	IGF2BP2	1524.49	1.76	0.06	27.07	0.00	0.00
ENSSSCG00000040337	AK4	3152.57	1.76	0.06	28.01	0.00	0.00
ENSSSCG00000038202		619.98	1.76	0.09	18.99	0.00	0.00
ENSSSCG00000039355		10.31	1.76	0.64	2.76	0.01	0.01
ENSSSCG00000027609	GC	308.74	1.76	0.14	12.71	0.00	0.00
ENSSSCG00000001657	CUL7	411.03	1.76	0.11	16.29	0.00	0.00
ENSSSCG00000003438	VPS13D	1098.10	1.76	0.07	24.00	0.00	0.00
ENSSSCG00000003859	TUT4	684.95	1.76	0.09	19.57	0.00	0.00
ENSSSCG00000037312	TMEM234	44.95	1.76	0.31	5.69	0.00	0.00
ENSSSCG00000022081	PHLDB2	2578.14	1.76	0.06	29.60	0.00	0.00
ENSSSCG00000039841	SHC3	8.88	1.77	0.70	2.51	0.01	0.02
ENSSSCG00000008712	PPP2R2C	933.14	1.77	0.09	19.46	0.00	0.00
ENSSSCG00000037847	MOB2	275.59	1.77	0.13	13.72	0.00	0.00
ENSSSCG00000013281	EXT2	617.36	1.77	0.09	19.69	0.00	0.00
ENSSSCG00000020858	KIF13A	289.15	1.77	0.13	13.45	0.00	0.00
ENSSSCG00000012576	CHRDL1	121.53	1.77	0.20	8.80	0.00	0.00
ENSSSCG00000000252	KRT8	16752.43	1.77	0.04	43.49	0.00	0.00
ENSSSCG00000032444	PLXDC2	47.28	1.77	0.29	6.05	0.00	0.00
ENSSSCG00000007700	HIP1	167.46	1.77	0.16	10.98	0.00	0.00
ENSSSCG00000009650	DOCK5	791.93	1.77	0.08	22.13	0.00	0.00
ENSSSCG00000039419	SLCO4A1	1100.70	1.77	0.16	10.82	0.00	0.00
ENSSSCG00000011454	SFMBT1	151.96	1.77	0.17	10.29	0.00	0.00

ENSSSCG00000008080	PHF2	381.11	1.77	0.11	15.56	0.00	0.00
ENSSSCG00000029458	SLC16A2	18.54	1.77	0.47	3.79	0.00	0.00
ENSSSCG00000013742	NFIX	127.66	1.77	0.18	9.59	0.00	0.00
ENSSSCG00000001469	SLA-DMB	152.65	1.77	0.17	10.58	0.00	0.00
ENSSSCG00000020725	ERBB3	3910.26	1.77	0.05	32.84	0.00	0.00
ENSSSCG00000002835	TOX3	248.74	1.77	0.14	13.03	0.00	0.00
ENSSSCG00000000152	RBFOX2	999.96	1.77	0.08	23.29	0.00	0.00
ENSSSCG00000011397	SLC38A3	170.24	1.77	0.16	11.01	0.00	0.00
ENSSSCG00000021273		31.07	1.78	0.37	4.86	0.00	0.00
ENSSSCG00000023074	KLK4	96.79	1.78	0.21	8.47	0.00	0.00
ENSSSCG00000032734	VOPP1	1185.62	1.78	0.07	25.00	0.00	0.00
ENSSSCG00000006053	BAALC	64.54	1.78	0.26	6.82	0.00	0.00
ENSSSCG00000038963		592.49	1.78	0.09	19.40	0.00	0.00
ENSSSCG00000002648	CBFA2T3	31.16	1.78	0.37	4.77	0.00	0.00
ENSSSCG00000038089	COL18A1	1675.29	1.78	0.06	29.32	0.00	0.00
ENSSSCG00000009805	HPD	8.15	1.78	0.71	2.51	0.01	0.02
ENSSSCG00000026990	FAM162A	1146.29	1.78	0.07	25.75	0.00	0.00
ENSSSCG00000017583	SGCA	57.65	1.78	0.28	6.38	0.00	0.00
ENSSSCG00000037676		66.46	1.78	0.26	6.80	0.00	0.00
ENSSSCG00000011593	TMCC1	273.63	1.78	0.13	13.83	0.00	0.00
ENSSSCG00000009929	TRPV4	938.46	1.78	0.08	23.10	0.00	0.00
ENSSSCG00000014112	JMY	964.18	1.78	0.09	19.93	0.00	0.00
ENSSSCG00000036865		37.50	1.79	0.34	5.29	0.00	0.00
ENSSSCG00000020702	SENP6	1586.83	1.79	0.06	27.66	0.00	0.00
ENSSSCG00000037260		11.87	1.79	0.60	2.98	0.00	0.00

ENSSSCG00000010957	AGTPBP1	165.41	1.79	0.16	11.09	0.00	0.00
ENSSSCG00000023084	ATP2B2	6.02	1.79	0.82	2.18	0.03	0.04
ENSSSCG00000015083	FXYD6	1107.67	1.79	0.07	23.92	0.00	0.00
ENSSSCG00000000419	RDH16	66.19	1.79	0.27	6.67	0.00	0.00
ENSSSCG00000000148		912.33	1.79	0.08	21.46	0.00	0.00
ENSSSCG00000014561	NLRP6	48.20	1.79	0.31	5.80	0.00	0.00
ENSSSCG00000008624	LPIN1	278.32	1.79	0.14	12.71	0.00	0.00
ENSSSCG00000026579		5.29	1.79	0.88	2.05	0.04	0.06
ENSSSCG00000012852	CDHR5	129.84	1.80	0.18	9.95	0.00	0.00
ENSSSCG00000028317	ARHGEF28	578.34	1.80	0.09	19.12	0.00	0.00
ENSSSCG00000005386	STX17	274.68	1.80	0.13	13.91	0.00	0.00
ENSSSCG00000012884	PPP6R3	1864.98	1.80	0.06	27.76	0.00	0.00
ENSSSCG00000005190	NFIB	195.38	1.80	0.16	11.60	0.00	0.00
ENSSSCG00000012828	STARD8	451.23	1.80	0.10	17.36	0.00	0.00
ENSSSCG00000007606	TRRAP	2595.44	1.80	0.05	33.74	0.00	0.00
ENSSSCG00000003881	SPATA6	422.50	1.80	0.11	16.91	0.00	0.00
ENSSSCG00000000675	C1R	449.69	1.80	0.10	17.67	0.00	0.00
ENSSSCG00000006066	RNF19A	1295.70	1.80	0.14	12.48	0.00	0.00
ENSSSCG00000015960	MAP3K20	747.32	1.80	0.09	19.40	0.00	0.00
ENSSSCG00000027477	TBC1D2B	314.37	1.80	0.12	14.86	0.00	0.00
ENSSSCG00000014380		16.59	1.81	0.49	3.68	0.00	0.00
ENSSSCG00000011056	FRMD4A	252.97	1.81	0.13	13.66	0.00	0.00
ENSSSCG00000007874	ABCC1	568.38	1.81	0.10	18.41	0.00	0.00
ENSSSCG00000016487	MGAM2	949.36	1.81	0.08	23.17	0.00	0.00
ENSSSCG00000017054	CYFIP2	1236.81	1.81	0.07	25.99	0.00	0.00

ENSSSCG00000010556	PAX2	483.55	1.81	0.11	16.94	0.00	0.00
ENSSSCG00000008468	PKDCC	491.02	1.81	0.11	17.13	0.00	0.00
ENSSSCG00000000570	C2CD5	731.24	1.81	0.09	20.12	0.00	0.00
ENSSSCG00000039714	MIPOL1	109.21	1.81	0.20	9.07	0.00	0.00
ENSSSCG00000018039	NCOR1	2726.92	1.82	0.05	33.28	0.00	0.00
ENSSSCG00000033452		506.63	1.82	0.10	18.52	0.00	0.00
ENSSSCG00000007458	NCOA3	3336.21	1.82	0.06	28.55	0.00	0.00
ENSSSCG00000000877	SCYL2	1138.89	1.82	0.08	22.90	0.00	0.00
ENSSSCG00000036431	VPS13C	3225.32	1.82	0.08	23.46	0.00	0.00
ENSSSCG00000036830		7.62	1.82	0.75	2.44	0.01	0.02
ENSSSCG00000013025	SLC22A12	9.83	1.82	0.66	2.77	0.01	0.01
ENSSSCG00000029030	SMCHD1	1199.21	1.82	0.08	22.10	0.00	0.00
ENSSSCG00000016613	AASS	760.94	1.83	0.08	21.82	0.00	0.00
ENSSSCG00000007868	TMC5	40.45	1.83	0.33	5.60	0.00	0.00
ENSSSCG00000027608	FAM172A	32.11	1.83	0.36	5.09	0.00	0.00
ENSSSCG00000023266	FOXJ3	1353.93	1.83	0.07	26.71	0.00	0.00
ENSSSCG00000007981	NPRL3	336.31	1.83	0.12	15.58	0.00	0.00
ENSSSCG00000016887	ITGA2	5132.71	1.83	0.05	33.87	0.00	0.00
ENSSSCG00000004587	MYO1E	1434.95	1.83	0.07	27.69	0.00	0.00
ENSSSCG00000010904	NEK7	546.34	1.83	0.09	19.37	0.00	0.00
ENSSSCG00000005742	DBH	110.56	1.83	0.22	8.33	0.00	0.00
ENSSSCG00000024552		25.19	1.83	0.40	4.52	0.00	0.00
ENSSSCG00000009319	PAN3	698.45	1.83	0.08	21.73	0.00	0.00
ENSSSCG00000011162	LARP4B	863.75	1.83	0.08	22.29	0.00	0.00
ENSSSCG00000037450	SSC5D	194.36	1.83	0.16	11.80	0.00	0.00

ENSSSCG00000015984	HOXD4	33.00	1.83	0.37	5.01	0.00	0.00
ENSSSCG00000034491	PRICKLE1	62.09	1.83	0.26	7.10	0.00	0.00
ENSSSCG00000013490	PIP5K1C	414.96	1.83	0.11	17.23	0.00	0.00
ENSSSCG00000016958	PIK3R1	832.76	1.83	0.08	22.34	0.00	0.00
ENSSSCG00000004404		382.85	1.84	0.11	15.96	0.00	0.00
ENSSSCG00000012862	OSBPL5	147.38	1.84	0.17	10.50	0.00	0.00
ENSSSCG00000015446	PNPLA8	1827.45	1.84	0.15	12.15	0.00	0.00
ENSSSCG00000035739	SLC26A1	24.47	1.84	0.42	4.36	0.00	0.00
ENSSSCG00000015394	KIAA1324L	73.64	1.84	0.24	7.56	0.00	0.00
ENSSSCG00000008447	CAMKMT	83.92	1.84	0.22	8.19	0.00	0.00
ENSSSCG00000039327	DAPP1	236.98	1.84	0.14	13.43	0.00	0.00
ENSSSCG00000021997	ALS2CL	1796.35	1.84	0.07	26.55	0.00	0.00
ENSSSCG00000003455	FHAD1	578.03	1.84	0.09	19.82	0.00	0.00
ENSSSCG00000021612	ARHGAP28	26.15	1.84	0.40	4.57	0.00	0.00
ENSSSCG00000001848	MESP2	185.82	1.84	0.16	11.80	0.00	0.00
ENSSSCG00000002266	CHD2	2190.42	1.84	0.07	27.88	0.00	0.00
ENSSSCG00000007155	C20orf194	716.72	1.84	0.09	20.93	0.00	0.00
ENSSSCG00000029248		434.78	1.84	0.11	17.09	0.00	0.00
ENSSSCG00000004311	RNGTT	357.99	1.84	0.12	15.68	0.00	0.00
ENSSSCG00000004941	DIS3L	94.04	1.84	0.22	8.49	0.00	0.00
ENSSSCG00000022288	STK38L	569.50	1.85	0.10	18.91	0.00	0.00
ENSSSCG00000003690	DLGAP1	102.32	1.85	0.21	8.93	0.00	0.00
ENSSSCG00000006746	VANGL1	719.63	1.85	0.09	21.31	0.00	0.00
ENSSSCG00000038501		8.56	1.85	0.72	2.57	0.01	0.02
ENSSSCG00000007650	STAG3	72.39	1.85	0.26	7.21	0.00	0.00

ENSSSCG00000025209		14.68	1.85	0.53	3.45	0.00	0.00
ENSSSCG00000038646		2297.58	1.85	0.06	31.11	0.00	0.00
ENSSSCG00000009157	TET2	1237.92	1.85	0.08	23.79	0.00	0.00
ENSSSCG00000021259	CDA	3919.13	1.85	0.05	34.44	0.00	0.00
ENSSSCG00000005446	EPB41L4B	218.17	1.85	0.15	12.71	0.00	0.00
ENSSSCG00000017892	PITPNM3	410.78	1.85	0.11	17.26	0.00	0.00
ENSSSCG00000011802	KNG1	11.56	1.85	0.61	3.05	0.00	0.00
ENSSSCG00000015135	SORL1	1718.18	1.85	0.06	30.46	0.00	0.00
ENSSSCG00000032527	FOSL2	3897.58	1.85	0.06	32.02	0.00	0.00
ENSSSCG00000025826	BOC	4.67	1.85	0.94	1.97	0.05	0.07
ENSSSCG00000011540	SETD5	2869.60	1.85	0.06	29.02	0.00	0.00
ENSSSCG00000014119	SERINC5	384.84	1.85	0.11	16.66	0.00	0.00
ENSSSCG00000005125	LRRC19	14.77	1.85	0.53	3.47	0.00	0.00
ENSSSCG00000034581	LRIG2	706.73	1.85	0.09	19.98	0.00	0.00
ENSSSCG00000033790		50.58	1.86	0.29	6.40	0.00	0.00
ENSSSCG00000035534		17.17	1.86	0.52	3.56	0.00	0.00
ENSSSCG00000011194	ANKRD28	1183.98	1.86	0.07	25.18	0.00	0.00
ENSSSCG00000027407	MYH14	2614.29	1.86	0.06	33.56	0.00	0.00
ENSSSCG00000006179		646.12	1.86	0.09	20.90	0.00	0.00
ENSSSCG00000009516	UBAC2	403.44	1.86	0.11	17.08	0.00	0.00
ENSSSCG00000026382	PPP2R5E	723.15	1.86	0.09	21.52	0.00	0.00
ENSSSCG00000034008		8.56	1.86	0.69	2.71	0.01	0.01
ENSSSCG00000013301	ELF5	10.09	1.86	0.64	2.93	0.00	0.01
ENSSSCG00000028338	PAMR1	7.72	1.86	0.73	2.53	0.01	0.02
ENSSSCG00000017638	TSPOAP1	264.35	1.86	0.13	14.18	0.00	0.00

ENSSSCG00000006719		50.70	1.86	0.29	6.45	0.00	0.00
ENSSSCG00000004779	PLCB2	690.48	1.86	0.08	22.02	0.00	0.00
ENSSSCG00000032775		6.26	1.86	0.81	2.30	0.02	0.03
ENSSSCG00000031901	PAPSS1	5603.09	1.86	0.05	38.41	0.00	0.00
ENSSSCG00000003710	TTC39C	218.79	1.87	0.15	12.72	0.00	0.00
ENSSSCG00000010753	CLRN3	24.17	1.87	0.42	4.48	0.00	0.00
ENSSSCG00000006793		429.59	1.87	0.12	16.01	0.00	0.00
ENSSSCG00000003356	MMEL1	9.35	1.87	0.66	2.84	0.00	0.01
ENSSSCG00000008966	PARM1	1177.43	1.87	0.08	23.79	0.00	0.00
ENSSSCG00000015851	TNKS	1123.69	1.87	0.07	25.57	0.00	0.00
ENSSSCG00000039188	AK8	49.90	1.87	0.29	6.41	0.00	0.00
ENSSSCG00000003506	PINK1	871.17	1.87	0.08	23.22	0.00	0.00
ENSSSCG00000015250	ADAMTS15	85.08	1.87	0.23	8.29	0.00	0.00
ENSSSCG00000033394	VWA3B	34.42	1.87	0.35	5.38	0.00	0.00
ENSSSCG00000011253	OXSR1	1078.02	1.87	0.08	23.72	0.00	0.00
ENSSSCG00000036542	PPP1R13L	10.20	1.87	0.65	2.88	0.00	0.01
ENSSSCG00000029813	TSPAN5	422.86	1.87	0.10	17.87	0.00	0.00
ENSSSCG00000022227	BRD4	1848.82	1.87	0.07	27.81	0.00	0.00
ENSSSCG00000011892		33.70	1.87	0.35	5.37	0.00	0.00
ENSSSCG00000004008		94.10	1.87	0.22	8.43	0.00	0.00
ENSSSCG00000025463	PROS1	1473.81	1.87	0.07	27.83	0.00	0.00
ENSSSCG00000033880		531.91	1.88	0.10	18.74	0.00	0.00
ENSSSCG00000003627	ZMYM4	535.52	1.88	0.10	19.06	0.00	0.00
ENSSSCG00000029783	MKX	18.89	1.88	0.47	3.98	0.00	0.00
ENSSSCG00000006709	NOTCH2	3303.76	1.88	0.05	35.50	0.00	0.00

ENSSSCG00000011474	PXK	1069.36	1.88	0.07	25.82	0.00	0.00
ENSSSCG00000008765		438.46	1.88	0.11	17.51	0.00	0.00
ENSSSCG00000040956		24.37	1.88	0.41	4.55	0.00	0.00
ENSSSCG00000008473		15.80	1.88	0.53	3.58	0.00	0.00
ENSSSCG00000026564		15.61	1.88	0.53	3.56	0.00	0.00
ENSSSCG00000039703	EEPD1	25.84	1.88	0.40	4.67	0.00	0.00
ENSSSCG00000030561	LMTK3	36.99	1.88	0.35	5.41	0.00	0.00
ENSSSCG00000034681		156.20	1.88	0.17	10.84	0.00	0.00
ENSSSCG00000032923	PRND	4.72	1.88	0.96	1.96	0.05	0.07
ENSSSCG00000017601	TOM1L1	1089.39	1.88	0.08	24.65	0.00	0.00
ENSSSCG00000004193	ENPP1	569.72	1.88	0.10	19.54	0.00	0.00
ENSSSCG00000035522		4.69	1.88	0.95	1.98	0.05	0.07
ENSSSCG00000038188		13.42	1.89	0.55	3.41	0.00	0.00
ENSSSCG00000002688		484.88	1.89	0.10	18.01	0.00	0.00
ENSSSCG00000009473	MYCBP2	1376.12	1.89	0.07	28.17	0.00	0.00
ENSSSCG00000015569	SWT1	110.64	1.89	0.20	9.57	0.00	0.00
ENSSSCG00000039279		11.12	1.89	0.64	2.97	0.00	0.00
ENSSSCG00000000997	PPP1R3G	450.81	1.89	0.11	16.90	0.00	0.00
ENSSSCG00000025703	REV3L	554.45	1.89	0.11	17.95	0.00	0.00
ENSSSCG00000001499	DST	5441.97	1.89	0.05	35.32	0.00	0.00
ENSSSCG00000014195	MAN2A1	600.79	1.89	0.09	20.09	0.00	0.00
ENSSSCG00000007488	DOK5	497.67	1.89	0.10	18.59	0.00	0.00
ENSSSCG00000000924	C12orf50	4.72	1.89	0.93	2.03	0.04	0.06
ENSSSCG00000037559	ACOT11	1185.97	1.89	0.08	23.41	0.00	0.00
ENSSSCG00000024136	AMPH	19.78	1.89	0.46	4.15	0.00	0.00

ENSSSCG00000017254	MAP2K6	7.16	1.89	0.76	2.49	0.01	0.02
ENSSSCG00000026078	GLCE	806.80	1.89	0.09	20.99	0.00	0.00
ENSSSCG00000000672	CLSTN3	349.49	1.89	0.12	16.27	0.00	0.00
ENSSSCG00000026569	CLEC16A	176.60	1.90	0.16	12.06	0.00	0.00
ENSSSCG00000003238	SPACA6	7.91	1.90	0.72	2.64	0.01	0.01
ENSSSCG00000012713	ATP11C	2009.67	1.90	0.06	31.95	0.00	0.00
ENSSSCG00000009591	AUH	305.70	1.90	0.12	15.33	0.00	0.00
ENSSSCG00000038003	ITK	42.14	1.90	0.32	5.93	0.00	0.00
ENSSSCG00000009839	CIT	1133.83	1.90	0.07	26.20	0.00	0.00
ENSSSCG00000032728	EFNB1	2015.82	1.90	0.06	30.72	0.00	0.00
ENSSSCG00000005979	ANXA13	2386.46	1.90	0.06	30.04	0.00	0.00
ENSSSCG00000040607	MAF	230.27	1.90	0.15	12.90	0.00	0.00
ENSSSCG00000000634	STYK1	51.72	1.90	0.29	6.57	0.00	0.00
ENSSSCG00000009642	STC1	40.01	1.91	0.33	5.76	0.00	0.00
ENSSSCG00000012132	ASB9	62.94	1.91	0.26	7.25	0.00	0.00
ENSSSCG00000006864	CDC14A	289.05	1.91	0.13	14.79	0.00	0.00
ENSSSCG00000016059	STAT4	4.78	1.91	0.93	2.06	0.04	0.05
ENSSSCG00000040123		58.48	1.91	0.28	6.90	0.00	0.00
ENSSSCG00000034036		50.41	1.91	0.29	6.51	0.00	0.00
ENSSSCG00000015754	MCPH1	287.51	1.91	0.13	14.57	0.00	0.00
ENSSSCG00000038993	HOXC4	20.73	1.91	0.45	4.23	0.00	0.00
ENSSSCG00000016218	PTPRN	45.60	1.91	0.32	5.91	0.00	0.00
ENSSSCG00000006624	SELENBP1	691.06	1.91	0.09	20.18	0.00	0.00
ENSSSCG00000012663	ENOX2	121.02	1.91	0.19	10.09	0.00	0.00
ENSSSCG00000005282		898.21	1.92	0.09	20.83	0.00	0.00

ENSSSCG00000036705	RPH3AL	152.21	1.92	0.18	10.47	0.00	0.00
ENSSSCG00000004921	ATP8B1	1906.64	1.92	0.07	28.51	0.00	0.00
ENSSSCG00000023539	MBOAT2	101.77	1.92	0.21	9.06	0.00	0.00
ENSSSCG00000011127	TAF3	223.48	1.92	0.15	12.48	0.00	0.00
ENSSSCG00000001921	NEO1	1321.40	1.92	0.07	26.67	0.00	0.00
ENSSSCG00000009048	GAB1	544.64	1.92	0.10	20.13	0.00	0.00
ENSSSCG00000040418	FSIP1	53.75	1.92	0.29	6.73	0.00	0.00
ENSSSCG00000023992	CEP112	205.56	1.92	0.15	12.51	0.00	0.00
ENSSSCG00000031118	PREX1	343.80	1.92	0.12	16.20	0.00	0.00
ENSSSCG00000005191	MPDZ	885.25	1.92	0.08	23.40	0.00	0.00
ENSSSCG00000029169	MAPKAP1	427.95	1.92	0.11	17.29	0.00	0.00
ENSSSCG00000010433	SGMS1	301.01	1.92	0.12	15.71	0.00	0.00
ENSSSCG00000037358		4381.07	1.92	0.13	15.02	0.00	0.00
ENSSSCG00000010509	PIK3AP1	4535.79	1.92	0.05	35.29	0.00	0.00
ENSSSCG00000002410	CEP128	183.01	1.92	0.16	11.88	0.00	0.00
ENSSSCG00000002512	DEGS2	1143.55	1.92	0.22	8.87	0.00	0.00
ENSSSCG00000014823	P2RY6	76.77	1.93	0.26	7.48	0.00	0.00
ENSSSCG00000006240	FAM110B	37.24	1.93	0.34	5.62	0.00	0.00
ENSSSCG00000021343	ZEB2	174.46	1.93	0.16	12.02	0.00	0.00
ENSSSCG00000031175	FNIP2	334.06	1.93	0.12	16.34	0.00	0.00
ENSSSCG00000023533	ZNF236	343.88	1.93	0.12	16.69	0.00	0.00
ENSSSCG00000005130	ELAVL2	236.61	1.93	0.14	13.58	0.00	0.00
ENSSSCG00000021200		114.39	1.93	0.20	9.78	0.00	0.00
ENSSSCG00000010437	PAPSS2	409.08	1.93	0.11	16.98	0.00	0.00
ENSSSCG00000038300	ALDOB	29.16	1.93	0.38	5.09	0.00	0.00

ENSSSCG00000036096		30.07	1.93	0.38	5.01	0.00	0.00
ENSSSCG00000033509	SAMD11	21.81	1.93	0.46	4.21	0.00	0.00
ENSSSCG00000031620		28.27	1.93	0.38	5.04	0.00	0.00
ENSSSCG00000001727	TNFRSF21	2163.36	1.93	0.06	33.32	0.00	0.00
ENSSSCG00000039144		35.64	1.93	0.35	5.55	0.00	0.00
ENSSSCG00000006506	SYT11	496.35	1.93	0.10	18.82	0.00	0.00
ENSSSCG00000017268	PRKCA	268.92	1.94	0.13	14.45	0.00	0.00
ENSSSCG00000025901	LCLAT1	349.87	1.94	0.12	16.74	0.00	0.00
ENSSSCG00000008576		131.07	1.94	0.20	9.75	0.00	0.00
ENSSSCG00000008578	ADCY3	1139.36	1.94	0.07	26.53	0.00	0.00
ENSSSCG00000011296	ANO10	155.82	1.94	0.17	11.13	0.00	0.00
ENSSSCG00000004082		113.99	1.94	0.20	9.68	0.00	0.00
ENSSSCG00000027667	EML1	211.59	1.94	0.15	13.06	0.00	0.00
ENSSSCG00000006927	PKN2	1799.99	1.94	0.07	27.14	0.00	0.00
ENSSSCG00000036976		5.70	1.94	0.87	2.23	0.03	0.04
ENSSSCG00000004535	TCF4	118.24	1.95	0.20	9.94	0.00	0.00
ENSSSCG00000014430	ABLIM3	578.42	1.95	0.10	20.48	0.00	0.00
ENSSSCG00000026583	TLR1	148.59	1.95	0.17	11.30	0.00	0.00
ENSSSCG00000010554	SCD	10257.09	1.95	0.05	39.21	0.00	0.00
ENSSSCG00000016842	NIPBL	1692.25	1.95	0.07	26.52	0.00	0.00
ENSSSCG00000009215	ABCG2	481.32	1.95	0.11	17.97	0.00	0.00
ENSSSCG00000026900	C15orf41	153.39	1.95	0.17	11.25	0.00	0.00
ENSSSCG00000003172	CCDC155	11.44	1.95	0.61	3.21	0.00	0.00
ENSSSCG00000012885		466.65	1.95	0.10	18.92	0.00	0.00
ENSSSCG00000012157	PHKA2	125.99	1.95	0.19	10.14	0.00	0.00

ENSSSCG00000033918		232.82	1.95	0.14	13.63	0.00	0.00
ENSSSCG00000005486	KIF12	736.01	1.95	0.09	22.13	0.00	0.00
ENSSSCG00000015732	TFCP2L1	47.62	1.95	0.31	6.38	0.00	0.00
ENSSSCG00000028092	GNG2	1676.17	1.95	0.07	28.23	0.00	0.00
ENSSSCG00000015509	COP1	423.34	1.95	0.11	18.38	0.00	0.00
ENSSSCG00000009567	RASA3	75.97	1.95	0.24	8.14	0.00	0.00
ENSSSCG00000038259	RF02271	7.37	1.95	0.78	2.50	0.01	0.02
ENSSSCG00000000549	PPFIBP1	783.07	1.96	0.09	20.96	0.00	0.00
ENSSSCG00000021149	RNF217	339.34	1.96	0.12	16.51	0.00	0.00
ENSSSCG00000039594	SSBP3	169.86	1.96	0.17	11.84	0.00	0.00
ENSSSCG00000009021	LRBA	509.22	1.96	0.10	18.83	0.00	0.00
ENSSSCG00000022478	STK10	1428.48	1.96	0.07	28.99	0.00	0.00
ENSSSCG00000014242	ZNF608	192.19	1.96	0.15	12.87	0.00	0.00
ENSSSCG00000027550	PLCD1	1204.64	1.96	0.07	27.76	0.00	0.00
ENSSSCG00000014567	TRIM66	273.66	1.96	0.13	15.25	0.00	0.00
ENSSSCG00000038853	GPR137B	305.23	1.96	0.12	15.97	0.00	0.00
ENSSSCG00000006002	CCN3	191.60	1.96	0.16	12.62	0.00	0.00
ENSSSCG00000002330	PCNX1	1147.94	1.96	0.08	25.36	0.00	0.00
ENSSSCG00000021203	PIK3CB	512.50	1.96	0.10	18.95	0.00	0.00
ENSSSCG00000005466	PTBP3	6416.99	1.96	0.06	31.67	0.00	0.00
ENSSSCG00000019270	MIR10A	11.57	1.96	0.64	3.08	0.00	0.00
ENSSSCG00000008836		15.70	1.97	0.52	3.77	0.00	0.00
ENSSSCG00000012769	ATP2B3	974.11	1.97	0.08	25.88	0.00	0.00
ENSSSCG00000003054	ZNF575	43.77	1.97	0.32	6.20	0.00	0.00
ENSSSCG00000037781		29.79	1.97	0.38	5.23	0.00	0.00

ENSSSCG00000007529	SYCP2	1062.33	1.97	0.09	22.17	0.00	0.00
ENSSSCG00000032931		4114.35	1.97	0.06	32.06	0.00	0.00
ENSSSCG00000016574	KCP	953.86	1.97	0.08	26.17	0.00	0.00
ENSSSCG00000040944	SKI	785.65	1.97	0.09	21.37	0.00	0.00
ENSSSCG00000003493	CAPZB	1538.57	1.97	0.06	30.60	0.00	0.00
ENSSSCG00000023762	TRPM2	5.89	1.97	0.88	2.24	0.03	0.04
ENSSSCG00000010172		2026.64	1.97	0.06	31.37	0.00	0.00
ENSSSCG00000010732	FAM53B	56.46	1.98	0.27	7.20	0.00	0.00
ENSSSCG00000001539	PPARD	626.72	1.98	0.11	18.54	0.00	0.00
ENSSSCG00000016608	IQUB	101.38	1.98	0.22	8.95	0.00	0.00
ENSSSCG00000036649	FAM151A	8.30	1.98	0.75	2.64	0.01	0.01
ENSSSCG00000036812	MPRIP	1176.91	1.98	0.07	27.44	0.00	0.00
ENSSSCG00000016898		232.08	1.98	0.14	13.97	0.00	0.00
ENSSSCG00000011519	GXYLT2	23.29	1.98	0.44	4.53	0.00	0.00
ENSSSCG00000004401	DDO	213.58	1.98	0.15	13.47	0.00	0.00
ENSSSCG00000022178		1128.62	1.98	0.08	26.21	0.00	0.00
ENSSSCG00000027207	GPATCH2	225.47	1.98	0.14	13.98	0.00	0.00
ENSSSCG00000010532	LOXL4	1044.98	1.98	0.07	27.03	0.00	0.00
ENSSSCG00000015402	CACNA2D1	1248.76	1.98	0.07	26.75	0.00	0.00
ENSSSCG00000002408	ADCK1	264.38	1.98	0.13	15.05	0.00	0.00
ENSSSCG00000024864	ATXN10	518.26	1.98	0.10	20.55	0.00	0.00
ENSSSCG00000002531	MOK	200.63	1.98	0.15	13.31	0.00	0.00
ENSSSCG00000033367	PLEKHA2	1983.60	1.98	0.07	26.98	0.00	0.00
ENSSSCG00000009114	PRSS12	100.79	1.98	0.21	9.34	0.00	0.00
ENSSSCG00000016545	CHCHD3	578.77	1.98	0.10	20.82	0.00	0.00

ENSSSCG00000007135	NINL	447.06	1.99	0.11	18.19	0.00	0.00
ENSSSCG00000007334	SRC	1698.61	1.99	0.06	31.24	0.00	0.00
ENSSSCG00000027225		4.98	1.99	0.97	2.05	0.04	0.06
ENSSSCG00000032377	RALGAPA1	787.40	1.99	0.08	23.65	0.00	0.00
ENSSSCG00000029249	NAV3	825.20	1.99	0.08	24.02	0.00	0.00
ENSSSCG00000030362		158.63	1.99	0.17	11.78	0.00	0.00
ENSSSCG00000017186	RNF157	40.97	1.99	0.33	5.95	0.00	0.00
ENSSSCG00000010432		76.76	1.99	0.26	7.64	0.00	0.00
ENSSSCG00000009338		23.37	1.99	0.43	4.65	0.00	0.00
ENSSSCG00000003684	MTCL1	497.73	1.99	0.10	20.28	0.00	0.00
ENSSSCG00000036868	CTNNBIP1	319.94	1.99	0.12	16.40	0.00	0.00
ENSSSCG00000031744		66.70	1.99	0.26	7.66	0.00	0.00
ENSSSCG00000040569	PLA2G10	5.85	1.99	0.86	2.32	0.02	0.03
ENSSSCG00000023907	AFAP1	279.31	1.99	0.13	15.41	0.00	0.00
ENSSSCG00000036168	PDSS2	164.89	1.99	0.17	11.67	0.00	0.00
ENSSSCG00000017072	GALNT10	739.58	1.99	0.09	22.77	0.00	0.00
ENSSSCG00000031261	RHOQ	503.09	1.99	0.10	19.47	0.00	0.00
ENSSSCG00000033660	FIGNL2	10.04	1.99	0.67	2.97	0.00	0.00
ENSSSCG00000022034	SMYD3	27.71	2.00	0.39	5.09	0.00	0.00
ENSSSCG00000017694	ACACA	836.38	2.00	0.08	24.84	0.00	0.00
ENSSSCG00000010943	AOPEP	305.17	2.00	0.13	15.11	0.00	0.00
ENSSSCG00000002386	IFT43	66.33	2.00	0.26	7.61	0.00	0.00
ENSSSCG00000001551		5.07	2.00	0.93	2.15	0.03	0.04
ENSSSCG00000005608	ANGPTL2	1176.91	2.00	0.07	28.22	0.00	0.00
ENSSSCG00000025362	RF00003	10.09	2.00	0.68	2.94	0.00	0.01

ENSSSCG00000024290		10.94	2.00	0.64	3.13	0.00	0.00
ENSSSCG00000022895	CRYBG3	416.59	2.00	0.11	17.88	0.00	0.00
ENSSSCG00000008803	ATP8A1	34.49	2.00	0.36	5.61	0.00	0.00
ENSSSCG00000009917	SPPL3	371.27	2.00	0.11	17.49	0.00	0.00
ENSSSCG00000002440	CCDC88C	125.99	2.00	0.19	10.52	0.00	0.00
ENSSSCG00000032827	DNAH5	376.94	2.01	0.12	16.74	0.00	0.00
ENSSSCG00000005943	ST3GAL1	37.14	2.01	0.34	5.94	0.00	0.00
ENSSSCG00000011026	ARHGAP12	708.35	2.01	0.10	20.15	0.00	0.00
ENSSSCG00000030581	VGLL4	1280.60	2.01	0.07	27.63	0.00	0.00
ENSSSCG00000016164	IKZF2	147.59	2.01	0.19	10.72	0.00	0.00
ENSSSCG00000014812	FOLR2	304.24	2.01	0.13	15.98	0.00	0.00
ENSSSCG00000003586	EPB41	1944.43	2.01	0.06	33.05	0.00	0.00
ENSSSCG00000033043	SHANK2	27.95	2.01	0.39	5.11	0.00	0.00
ENSSSCG00000011699	HPS3	773.44	2.02	0.09	23.46	0.00	0.00
ENSSSCG00000039498	RBFOX3	18.69	2.02	0.49	4.14	0.00	0.00
ENSSSCG00000000036	PACSIN2	2025.65	2.02	0.06	34.35	0.00	0.00
ENSSSCG00000032814	DNAH7	34.11	2.02	0.36	5.61	0.00	0.00
ENSSSCG00000024018	SLC16A3	3890.06	2.02	0.07	30.35	0.00	0.00
ENSSSCG00000000811	PCED1B	62.23	2.02	0.26	7.70	0.00	0.00
ENSSSCG00000029578	NFATC3	576.10	2.03	0.10	21.27	0.00	0.00
ENSSSCG00000011090	NEBL	41.70	2.03	0.32	6.33	0.00	0.00
ENSSSCG00000040789	GPR52	14.50	2.03	0.54	3.77	0.00	0.00
ENSSSCG00000021784	TBCK	566.74	2.03	0.10	20.92	0.00	0.00
ENSSSCG00000032968	VTI1A	63.81	2.03	0.26	7.79	0.00	0.00
ENSSSCG00000013236	MYBPC3	68.24	2.03	0.25	8.03	0.00	0.00

ENSSSCG00000009781	PITPNM2	395.47	2.03	0.11	17.71	0.00	0.00
ENSSSCG00000011950	NXPE3	221.57	2.03	0.15	13.82	0.00	0.00
ENSSSCG00000013321	ELP4	206.75	2.03	0.15	13.59	0.00	0.00
ENSSSCG00000003761	ADGRL2	1247.90	2.03	0.08	26.21	0.00	0.00
ENSSSCG00000031683		119.30	2.03	0.20	10.02	0.00	0.00
ENSSSCG00000008314	ATP6V1B1	230.17	2.03	0.14	14.43	0.00	0.00
ENSSSCG00000008689	ZFYVE28	49.68	2.04	0.30	6.85	0.00	0.00
ENSSSCG00000015488	TNFSF4	6.82	2.04	0.79	2.57	0.01	0.02
ENSSSCG00000022557	SUN3	4.29	2.04	1.00	2.04	0.04	0.06
ENSSSCG00000009511	STK24	1704.63	2.04	0.06	32.72	0.00	0.00
ENSSSCG00000001534	ANKS1A	298.62	2.04	0.13	16.29	0.00	0.00
ENSSSCG00000040100	ITPKB	21.47	2.04	0.45	4.48	0.00	0.00
ENSSSCG00000029714	BPIFB2	90.05	2.04	0.22	9.25	0.00	0.00
ENSSSCG00000033971	ZNF407	196.63	2.04	0.15	13.33	0.00	0.00
ENSSSCG00000029251		3310.06	2.04	0.05	40.00	0.00	0.00
ENSSSCG00000004979	MYO9A	660.98	2.04	0.10	19.98	0.00	0.00
ENSSSCG00000026427	RORC	30.99	2.04	0.39	5.27	0.00	0.00
ENSSSCG00000000020	PHF21B	43.07	2.04	0.34	6.01	0.00	0.00
ENSSSCG00000006912	HFM1	41.35	2.04	0.34	6.04	0.00	0.00
ENSSSCG00000007456	SULF2	142.81	2.04	0.19	10.79	0.00	0.00
ENSSSCG00000029998	KLF7	540.71	2.04	0.10	20.59	0.00	0.00
ENSSSCG00000039252	TCN1	6.02	2.04	0.86	2.39	0.02	0.02
ENSSSCG00000013599	ANGPTL4	1870.85	2.05	0.07	29.64	0.00	0.00
ENSSSCG00000004405	FIG4	414.37	2.05	0.11	18.36	0.00	0.00
ENSSSCG00000032731	NDST1	811.94	2.05	0.09	23.43	0.00	0.00

ENSSSCG00000015853	HERC2	1405.42	2.05	0.07	29.76	0.00	0.00
ENSSSCG00000011881	IQCB1	307.91	2.05	0.13	16.18	0.00	0.00
ENSSSCG00000009809	MORN3	4.32	2.05	1.00	2.06	0.04	0.05
ENSSSCG00000032950	SLC25A34	15.55	2.06	0.52	3.93	0.00	0.00
ENSSSCG00000023920	P3H1	9357.41	2.06	0.05	37.77	0.00	0.00
ENSSSCG00000004024		268.77	2.06	0.13	15.62	0.00	0.00
ENSSSCG00000013376	OTOG	6.06	2.06	0.84	2.44	0.01	0.02
ENSSSCG00000007949	SRL	25.27	2.06	0.42	4.92	0.00	0.00
ENSSSCG00000003970	GUCA2B	8.67	2.06	0.73	2.81	0.00	0.01
ENSSSCG00000005720	MED27	108.75	2.06	0.21	9.72	0.00	0.00
ENSSSCG00000011579	PPARG	1389.47	2.06	0.07	30.26	0.00	0.00
ENSSSCG00000010212		830.39	2.06	0.09	23.89	0.00	0.00
ENSSSCG00000004955	MAP2K5	219.95	2.06	0.15	14.12	0.00	0.00
ENSSSCG00000036695	IGF2BP3	1538.33	2.06	0.07	31.66	0.00	0.00
ENSSSCG00000006871	SLC35A3	2216.94	2.06	0.07	31.53	0.00	0.00
ENSSSCG00000017978	ALOX15B	58.27	2.06	0.28	7.43	0.00	0.00
ENSSSCG00000001963	EGLN3	6240.05	2.06	0.13	15.85	0.00	0.00
ENSSSCG00000013663	ANGPTL6	9.55	2.07	0.67	3.06	0.00	0.00
ENSSSCG00000040457	EPN2	386.32	2.07	0.11	18.54	0.00	0.00
ENSSSCG00000040538	WDFY2	93.49	2.07	0.23	8.94	0.00	0.00
ENSSSCG00000022948	SLC7A9	35.70	2.07	0.35	5.89	0.00	0.00
ENSSSCG00000014904	DLG2	113.64	2.07	0.20	10.17	0.00	0.00
ENSSSCG00000014066	TMEM171	54.14	2.07	0.29	7.17	0.00	0.00
ENSSSCG00000012182	PCYT1B	17.61	2.08	0.51	4.11	0.00	0.00
ENSSSCG00000002524	AMN	45.59	2.08	0.31	6.72	0.00	0.00

ENSSSCG00000010861	COQ8A	169.95	2.08	0.17	12.50	0.00	0.00
ENSSSCG00000032392	MYO15A	137.73	2.08	0.18	11.43	0.00	0.00
ENSSSCG00000036790	AKAP7	66.76	2.08	0.25	8.17	0.00	0.00
ENSSSCG00000006250	LYN	1155.43	2.08	0.07	28.50	0.00	0.00
ENSSSCG00000005449	PTPN3	917.04	2.08	0.08	25.24	0.00	0.00
ENSSSCG00000011022	SVIL	447.42	2.08	0.11	19.71	0.00	0.00
ENSSSCG00000036148		6.13	2.08	0.86	2.43	0.02	0.02
ENSSSCG00000016590	SND1	678.64	2.08	0.09	23.26	0.00	0.00
ENSSSCG00000007719		1134.84	2.08	0.07	28.63	0.00	0.00
ENSSSCG00000012741	MAMLD1	376.22	2.08	0.12	17.12	0.00	0.00
ENSSSCG00000039677	CHRNA4	55.59	2.08	0.29	7.14	0.00	0.00
ENSSSCG00000026733	HIPK2	418.51	2.08	0.11	19.11	0.00	0.00
ENSSSCG00000001052	PHACTR1	21.94	2.09	0.45	4.61	0.00	0.00
ENSSSCG00000015010	EXPH5	87.40	2.09	0.23	9.20	0.00	0.00
ENSSSCG00000035581	SUGCT	102.40	2.09	0.21	9.74	0.00	0.00
ENSSSCG00000009870	SDSL	43.24	2.09	0.32	6.61	0.00	0.00
ENSSSCG00000028877	RAD51B	38.73	2.09	0.34	6.15	0.00	0.00
ENSSSCG00000007477	NFATC2	584.74	2.09	0.10	21.27	0.00	0.00
ENSSSCG00000009545	COL4A2	3325.38	2.09	0.06	37.83	0.00	0.00
ENSSSCG00000019059	RF00026	4.39	2.09	1.00	2.09	0.04	0.05
ENSSSCG00000009345	PDS5B	1292.68	2.09	0.07	30.05	0.00	0.00
ENSSSCG00000036706	SRMS	4.37	2.10	1.01	2.07	0.04	0.05
ENSSSCG00000040476		7.09	2.10	0.78	2.68	0.01	0.01
ENSSSCG00000006453	KIRREL1	839.48	2.10	0.08	25.85	0.00	0.00
ENSSSCG00000017791	SSH2	779.01	2.10	0.09	23.63	0.00	0.00

ENSSSCG00000036454	TMEM220	109.36	2.10	0.21	10.09	0.00	0.00
ENSSSCG00000031348		63.13	2.10	0.28	7.61	0.00	0.00
ENSSSCG00000009281	SGCG	9.77	2.10	0.67	3.13	0.00	0.00
ENSSSCG00000034114	GPR68	1015.35	2.10	0.08	27.57	0.00	0.00
ENSSSCG00000032956	BMERB1	37.35	2.10	0.34	6.15	0.00	0.00
ENSSSCG00000005170	DENND4C	1445.47	2.10	0.07	28.85	0.00	0.00
ENSSSCG00000026268	DLG5	1850.58	2.10	0.06	32.74	0.00	0.00
ENSSSCG00000035284	BMF	1177.43	2.10	0.07	29.24	0.00	0.00
ENSSSCG00000036679	SORBS2	2346.69	2.11	0.06	36.56	0.00	0.00
ENSSSCG00000015944	TLK1	784.67	2.11	0.09	24.20	0.00	0.00
ENSSSCG00000002718	FA2H	638.84	2.11	0.10	20.47	0.00	0.00
ENSSSCG00000025910	ZNF277	873.79	2.11	0.09	23.53	0.00	0.00
ENSSSCG00000034725	UBE2R2	806.57	2.11	0.08	25.60	0.00	0.00
ENSSSCG00000003579	AHDC1	438.32	2.11	0.11	18.73	0.00	0.00
ENSSSCG00000021038	NRIP1	2150.90	2.11	0.06	33.90	0.00	0.00
ENSSSCG00000003514	HSPG2	21660.68	2.12	0.04	55.55	0.00	0.00
ENSSSCG00000035940	SPSB1	488.03	2.12	0.11	19.92	0.00	0.00
ENSSSCG00000022302	BICRA	140.22	2.12	0.20	10.83	0.00	0.00
ENSSSCG00000001716	RCAN2	565.74	2.12	0.10	21.81	0.00	0.00
ENSSSCG00000008903	KIAA1211	133.86	2.12	0.20	10.63	0.00	0.00
ENSSSCG00000014132		140.94	2.12	0.18	11.74	0.00	0.00
ENSSSCG00000015346	ICA1	162.94	2.12	0.17	12.55	0.00	0.00
ENSSSCG00000032804		72.97	2.12	0.25	8.34	0.00	0.00
ENSSSCG00000011915	GRAMD1C	175.20	2.12	0.16	12.97	0.00	0.00
ENSSSCG00000005614	FAM129B	3759.30	2.12	0.06	38.30	0.00	0.00

ENSSSCG00000000025	PARVG	19.98	2.13	0.49	4.37	0.00	0.00
ENSSSCG00000036135	COL1A1	627.84	2.13	0.09	22.74	0.00	0.00
ENSSSCG00000031592	ZNF462	372.10	2.13	0.12	18.25	0.00	0.00
ENSSSCG00000012166		2109.35	2.13	0.06	33.04	0.00	0.00
ENSSSCG00000004205	ARHGAP18	1801.93	2.13	0.08	26.13	0.00	0.00
ENSSSCG00000006346	ATF6	4313.91	2.13	0.05	39.17	0.00	0.00
ENSSSCG00000005215	JAK2	541.64	2.13	0.10	20.39	0.00	0.00
ENSSSCG00000024138	RF00026	18.86	2.13	0.50	4.29	0.00	0.00
ENSSSCG00000038652		5.43	2.13	0.89	2.39	0.02	0.02
ENSSSCG00000010825	MARK1	420.17	2.13	0.11	19.37	0.00	0.00
ENSSSCG00000010056		517.58	2.13	0.10	21.74	0.00	0.00
ENSSSCG00000000999		233.49	2.13	0.14	14.90	0.00	0.00
ENSSSCG00000037530		96.38	2.13	0.23	9.34	0.00	0.00
ENSSSCG00000002436	TTC7B	204.74	2.13	0.15	13.81	0.00	0.00
ENSSSCG00000005785	PCSK6	836.38	2.13	0.08	25.35	0.00	0.00
ENSSSCG00000016295	NGEF	56.25	2.14	0.28	7.65	0.00	0.00
ENSSSCG00000030361	PRKCZ	601.55	2.14	0.10	21.54	0.00	0.00
ENSSSCG00000013448	MKNK2	8725.80	2.14	0.05	41.82	0.00	0.00
ENSSSCG00000036396	RF01956	951.54	2.14	0.20	10.70	0.00	0.00
ENSSSCG00000034795	SCAMP1	1172.20	2.14	0.09	24.56	0.00	0.00
ENSSSCG00000034068		40.96	2.14	0.33	6.57	0.00	0.00
ENSSSCG00000024061	TNNI1	95.63	2.14	0.22	9.89	0.00	0.00
ENSSSCG00000036516		176.76	2.15	0.16	13.30	0.00	0.00
ENSSSCG00000001089	GPLD1	40.38	2.15	0.35	6.09	0.00	0.00
ENSSSCG00000003592	SDC3	2001.43	2.15	0.07	28.97	0.00	0.00

ENSSSCG00000013246	C11orf49	40.30	2.15	0.35	6.13	0.00	0.00
ENSSSCG00000005425	SLC44A1	2362.52	2.15	0.07	31.93	0.00	0.00
ENSSSCG00000015874	ACVR1	454.87	2.15	0.11	19.91	0.00	0.00
ENSSSCG00000038476		17.33	2.15	0.51	4.26	0.00	0.00
ENSSSCG00000022417	HNF1B	1317.65	2.15	0.08	28.02	0.00	0.00
ENSSSCG00000003006	CYP2B22	127.16	2.15	0.20	10.88	0.00	0.00
ENSSSCG00000029331	PALLD	876.94	2.16	0.09	23.20	0.00	0.00
ENSSSCG00000004576	RORA	278.71	2.16	0.13	16.40	0.00	0.00
ENSSSCG00000016230	EPHA4	51.61	2.16	0.31	7.03	0.00	0.00
ENSSSCG00000014401	NR3C1	571.68	2.16	0.11	20.52	0.00	0.00
ENSSSCG00000022204	AGRN	11289.19	2.16	0.04	50.41	0.00	0.00
ENSSSCG00000038973	PPP1R12A	906.41	2.16	0.08	26.01	0.00	0.00
ENSSSCG00000001076	RNF144B	136.30	2.16	0.19	11.58	0.00	0.00
ENSSSCG00000026498		1325.84	2.16	0.07	31.23	0.00	0.00
ENSSSCG00000012110	MID1	165.58	2.16	0.17	12.47	0.00	0.00
ENSSSCG00000014126	MSH3	164.01	2.16	0.19	11.30	0.00	0.00
ENSSSCG00000004958	PIAS1	924.89	2.16	0.08	25.73	0.00	0.00
ENSSSCG00000011870	PDIA5	423.61	2.17	0.11	19.79	0.00	0.00
ENSSSCG00000008034	NOXO1	119.64	2.17	0.20	10.63	0.00	0.00
ENSSSCG00000040145	SNAI3	7.33	2.17	0.81	2.68	0.01	0.01
ENSSSCG00000023273	SH3YL1	291.23	2.17	0.13	16.78	0.00	0.00
ENSSSCG00000014088	IQGAP2	1390.88	2.17	0.07	31.21	0.00	0.00
ENSSSCG00000010791	FUOM	619.53	2.17	0.10	22.00	0.00	0.00
ENSSSCG00000014331	KDM3B	191.39	2.17	0.16	13.68	0.00	0.00
ENSSSCG00000005689	FNBP1	683.83	2.17	0.09	24.44	0.00	0.00

ENSSSCG00000035668	C9orf135	34.11	2.17	0.36	5.97	0.00	0.00
ENSSSCG00000018056	SRCIN1	44.31	2.17	0.33	6.57	0.00	0.00
ENSSSCG00000028827	PARVA	492.98	2.17	0.11	20.64	0.00	0.00
ENSSSCG00000024837	SYT12	235.67	2.17	0.14	15.28	0.00	0.00
ENSSSCG00000005471	SNX30	178.92	2.17	0.16	13.49	0.00	0.00
ENSSSCG00000040181	ELL	1120.34	2.18	0.08	26.40	0.00	0.00
ENSSSCG00000008302	FBXO41	96.19	2.18	0.22	9.95	0.00	0.00
ENSSSCG00000022009	DDC	353.84	2.18	0.12	17.91	0.00	0.00
ENSSSCG00000031960		13.95	2.18	0.57	3.81	0.00	0.00
ENSSSCG00000014339	CTNNA1	4326.87	2.18	0.05	43.11	0.00	0.00
ENSSSCG00000020872		1392.01	2.18	0.07	30.99	0.00	0.00
ENSSSCG00000009684	MSRA	16.76	2.18	0.51	4.24	0.00	0.00
ENSSSCG00000005095		238.65	2.18	0.14	15.35	0.00	0.00
ENSSSCG00000028327	RHOBTB1	68.03	2.18	0.26	8.35	0.00	0.00
ENSSSCG00000005715	PRRC2B	4305.05	2.18	0.06	35.25	0.00	0.00
ENSSSCG00000038196		24.13	2.18	0.43	5.06	0.00	0.00
ENSSSCG00000036401		599.85	2.19	0.10	22.72	0.00	0.00
ENSSSCG00000016005	SESTD1	324.34	2.19	0.13	16.58	0.00	0.00
ENSSSCG00000023784	SEMA3C	2127.48	2.19	0.06	34.74	0.00	0.00
ENSSSCG00000024388	BNIP3	8380.40	2.19	0.13	16.32	0.00	0.00
ENSSSCG00000039514	ID3	592.31	2.19	0.10	21.48	0.00	0.00
ENSSSCG00000000423	LRP1	4582.66	2.19	0.05	46.52	0.00	0.00
ENSSSCG00000011386	MST1	47.85	2.19	0.30	7.20	0.00	0.00
ENSSSCG00000031746	KCNQ2	119.71	2.19	0.21	10.44	0.00	0.00
ENSSSCG00000012238		358.48	2.19	0.12	17.96	0.00	0.00

ENSSSCG00000004603	RFX7	903.22	2.19	0.08	26.84	0.00	0.00
ENSSSCG00000029275	PPARGC1A	268.08	2.20	0.14	15.75	0.00	0.00
ENSSSCG00000015940	UBR3	830.81	2.20	0.08	26.14	0.00	0.00
ENSSSCG00000010185	GALNT2	756.26	2.20	0.09	25.12	0.00	0.00
ENSSSCG00000010292	P4HA1	12454.70	2.20	0.05	42.12	0.00	0.00
ENSSSCG00000017168	SEPT9	1512.19	2.20	0.07	32.87	0.00	0.00
ENSSSCG00000016806		768.49	2.20	0.09	24.34	0.00	0.00
ENSSSCG00000017738	ADAP2	7.63	2.20	0.80	2.75	0.01	0.01
ENSSSCG00000008337	AAK1	585.40	2.20	0.10	21.04	0.00	0.00
ENSSSCG00000008187	KIAA1211L	129.23	2.21	0.20	11.27	0.00	0.00
ENSSSCG00000023665	FBXO25	200.50	2.21	0.16	14.21	0.00	0.00
ENSSSCG00000012026	ADAMTS1	948.46	2.21	0.16	13.76	0.00	0.00
ENSSSCG00000005232	SMARCA2	588.30	2.21	0.10	22.89	0.00	0.00
ENSSSCG00000025156	BRWD3	749.31	2.21	0.09	23.63	0.00	0.00
ENSSSCG00000040875	ZFPM1	89.81	2.21	0.23	9.42	0.00	0.00
ENSSSCG00000034059	ST3GAL3	43.62	2.21	0.33	6.77	0.00	0.00
ENSSSCG00000008771	C4orf19	171.24	2.21	0.16	13.42	0.00	0.00
ENSSSCG00000013397	ARNTL	156.10	2.21	0.17	12.89	0.00	0.00
ENSSSCG00000015806		44.57	2.21	0.32	6.86	0.00	0.00
ENSSSCG00000009449	TDRD3	95.79	2.22	0.22	10.11	0.00	0.00
ENSSSCG00000013876	MYO9B	1901.67	2.22	0.06	34.15	0.00	0.00
ENSSSCG00000013915	CRTC1	218.02	2.22	0.15	15.12	0.00	0.00
ENSSSCG00000039770	SLC6A9	522.52	2.22	0.12	18.59	0.00	0.00
ENSSSCG00000004358	ASCC3	1364.34	2.22	0.07	29.62	0.00	0.00
ENSSSCG00000008513	BIRC6	1921.25	2.22	0.07	34.08	0.00	0.00

ENSSSCG00000005719	RAPGEF1	531.22	2.22	0.10	22.08	0.00	0.00
ENSSSCG00000010571	ARMH3	174.78	2.22	0.17	13.33	0.00	0.00
ENSSSCG00000014792	STIM1	253.45	2.22	0.15	15.28	0.00	0.00
ENSSSCG00000010544	DNMBP	297.32	2.22	0.13	17.31	0.00	0.00
ENSSSCG00000024001	PLXNA1	4726.52	2.22	0.05	45.11	0.00	0.00
ENSSSCG00000028063	TACC2	157.64	2.22	0.18	12.26	0.00	0.00
ENSSSCG00000008427	KCNK12	201.13	2.22	0.15	14.36	0.00	0.00
ENSSSCG00000008989	CNOT6L	731.89	2.23	0.09	24.97	0.00	0.00
ENSSSCG00000015657	PIGR	1075.42	2.23	0.08	28.04	0.00	0.00
ENSSSCG00000011714	MED12L	73.35	2.23	0.25	8.93	0.00	0.00
ENSSSCG00000025053	RYBP	1543.75	2.23	0.07	33.24	0.00	0.00
ENSSSCG00000039023		16.21	2.23	0.54	4.09	0.00	0.00
ENSSSCG00000029943	SCLT1	252.88	2.23	0.15	15.16	0.00	0.00
ENSSSCG00000015864	MYO7B	5746.44	2.23	0.05	49.10	0.00	0.00
ENSSSCG00000004861	TSHZ1	80.38	2.23	0.24	9.26	0.00	0.00
ENSSSCG00000024975	NR5A2	5.72	2.23	0.88	2.53	0.01	0.02
ENSSSCG00000033329	SLC47A1	10.49	2.23	0.65	3.42	0.00	0.00
ENSSSCG00000024307	PRM2	4.79	2.23	0.96	2.33	0.02	0.03
ENSSSCG00000003405	PEX14	211.97	2.23	0.16	13.90	0.00	0.00
ENSSSCG00000033262	CD74	261.39	2.23	0.15	15.02	0.00	0.00
ENSSSCG00000015780	STOX2	203.88	2.23	0.16	13.88	0.00	0.00
ENSSSCG00000025408	ULK4	119.88	2.23	0.20	11.13	0.00	0.00
ENSSSCG00000022721	CDH16	966.42	2.23	0.08	29.04	0.00	0.00
ENSSSCG00000036804	TCEA2	887.98	2.23	0.08	28.03	0.00	0.00
ENSSSCG00000012773	PNCK	548.68	2.24	0.10	21.60	0.00	0.00

ENSSSCG00000017748	NF1	5029.11	2.24	0.05	42.85	0.00	0.00
ENSSSCG00000028771	MEF2A	1884.72	2.24	0.07	33.33	0.00	0.00
ENSSSCG00000031801		5.73	2.24	0.88	2.55	0.01	0.02
ENSSSCG00000032841	RF01955	843.47	2.24	0.20	11.40	0.00	0.00
ENSSSCG00000010403	MARCH8	729.02	2.24	0.09	25.90	0.00	0.00
ENSSSCG00000012564	MID2	154.63	2.24	0.18	12.74	0.00	0.00
ENSSSCG00000017237	CD300LB	65.35	2.24	0.27	8.15	0.00	0.00
ENSSSCG00000034242	HNF4G	110.78	2.24	0.20	11.00	0.00	0.00
ENSSSCG00000014852	ARRB1	12.49	2.24	0.60	3.73	0.00	0.00
ENSSSCG00000015958	PDK1	5279.58	2.25	0.12	18.17	0.00	0.00
ENSSSCG00000004040	SLC22A2	56.96	2.25	0.29	7.83	0.00	0.00
ENSSSCG00000000528	PKP2	1499.73	2.25	0.07	33.53	0.00	0.00
ENSSSCG00000017142	BAIAP2	965.27	2.25	0.08	27.82	0.00	0.00
ENSSSCG00000001746	PKHD1	119.29	2.25	0.20	11.32	0.00	0.00
ENSSSCG00000021026	TMEM51	538.05	2.25	0.10	22.78	0.00	0.00
ENSSSCG00000023403		250.02	2.26	0.14	15.92	0.00	0.00
ENSSSCG00000007899		232.73	2.26	0.15	15.12	0.00	0.00
ENSSSCG00000036417		56.16	2.26	0.30	7.58	0.00	0.00
ENSSSCG00000013074	RAB3IL1	321.67	2.26	0.13	18.06	0.00	0.00
ENSSSCG00000016338	PER2	70.75	2.26	0.26	8.76	0.00	0.00
ENSSSCG00000026433	CCDC73	27.23	2.26	0.41	5.58	0.00	0.00
ENSSSCG00000032673	ZFP14	17.44	2.26	0.54	4.21	0.00	0.00
ENSSSCG00000040288	ARNT2	259.55	2.26	0.15	15.38	0.00	0.00
ENSSSCG00000034293	ARL4C	2056.44	2.26	0.07	33.01	0.00	0.00
ENSSSCG00000009595		37.97	2.26	0.34	6.57	0.00	0.00

ENSSSCG00000009613	DMTN	137.74	2.27	0.19	11.84	0.00	0.00
ENSSSCG00000032590		31.54	2.27	0.41	5.46	0.00	0.00
ENSSSCG00000032334		43.95	2.27	0.34	6.74	0.00	0.00
ENSSSCG00000025130	KLF12	67.41	2.27	0.26	8.57	0.00	0.00
ENSSSCG00000002517	WDR25	92.12	2.27	0.23	9.95	0.00	0.00
ENSSSCG00000021731	WWC2	952.83	2.27	0.08	28.37	0.00	0.00
ENSSSCG00000013934	CILP2	10.81	2.27	0.66	3.44	0.00	0.00
ENSSSCG00000029388	PDE2A	683.24	2.27	0.10	23.23	0.00	0.00
ENSSSCG00000009683	KIF13B	441.50	2.27	0.11	20.57	0.00	0.00
ENSSSCG00000032566	ZHX3	323.93	2.27	0.12	18.23	0.00	0.00
ENSSSCG00000006958	TOP1MT	29.48	2.27	0.40	5.64	0.00	0.00
ENSSSCG00000002526	RCOR1	1041.98	2.28	0.08	28.07	0.00	0.00
ENSSSCG00000002671	ATP2C2	14.77	2.28	0.57	3.98	0.00	0.00
ENSSSCG00000032791		3.91	2.28	1.08	2.12	0.03	0.05
ENSSSCG00000039245	RIMS3	91.31	2.28	0.23	10.08	0.00	0.00
ENSSSCG00000025273	CYP11A1	3.93	2.28	1.07	2.14	0.03	0.05
ENSSSCG00000032029	PPP2R3B	193.69	2.28	0.16	14.30	0.00	0.00
ENSSSCG00000011750	PLD1	304.91	2.28	0.14	16.66	0.00	0.00
ENSSSCG00000011819	MB21D2	89.53	2.28	0.23	9.78	0.00	0.00
ENSSSCG00000016243	RHBDD1	257.22	2.28	0.15	15.28	0.00	0.00
ENSSSCG00000001841	RHCG	3.93	2.29	1.09	2.10	0.04	0.05
ENSSSCG00000039798	SLC11A1	3.95	2.29	1.07	2.14	0.03	0.05
ENSSSCG00000038639	TOM1L2	279.43	2.29	0.14	16.83	0.00	0.00
ENSSSCG00000016457	TAS2R41	12.87	2.29	0.59	3.86	0.00	0.00
ENSSSCG00000007927	PPL	6674.38	2.29	0.05	46.14	0.00	0.00

ENSSSCG00000040169		14.84	2.30	0.57	4.06	0.00	0.00
ENSSSCG00000003795	WLS	2939.61	2.30	0.05	42.49	0.00	0.00
ENSSSCG00000022112	DOCK9	946.33	2.30	0.08	29.00	0.00	0.00
ENSSSCG00000023710	REEP1	247.18	2.30	0.15	14.97	0.00	0.00
ENSSSCG00000016873	NIM1K	68.56	2.30	0.27	8.61	0.00	0.00
ENSSSCG00000030420	GLG1	2424.76	2.30	0.06	39.15	0.00	0.00
ENSSSCG00000011489		114.66	2.30	0.21	11.10	0.00	0.00
ENSSSCG00000038774	MAPK13	7.01	2.30	0.84	2.73	0.01	0.01
ENSSSCG00000001695	VEGFA	6687.00	2.30	0.05	49.51	0.00	0.00
ENSSSCG00000004762	DNAJC17	338.52	2.30	0.13	17.13	0.00	0.00
ENSSSCG00000027675		1145.68	2.31	0.08	30.45	0.00	0.00
ENSSSCG00000026592	TLR6	73.87	2.31	0.26	8.96	0.00	0.00
ENSSSCG00000001914	LOXL1	77.97	2.31	0.24	9.44	0.00	0.00
ENSSSCG00000011670	PXYLP1	546.97	2.31	0.10	23.34	0.00	0.00
ENSSSCG00000005388	INVS	266.30	2.31	0.14	16.95	0.00	0.00
ENSSSCG00000032580	MGST1	743.04	2.31	0.09	26.22	0.00	0.00
ENSSSCG00000031831	CADM1	274.49	2.31	0.14	16.18	0.00	0.00
ENSSSCG00000004508	DYM	453.80	2.32	0.11	21.65	0.00	0.00
ENSSSCG00000031476	KLHL6	4.02	2.32	1.10	2.11	0.04	0.05
ENSSSCG00000028203		90.70	2.32	0.23	10.00	0.00	0.00
ENSSSCG00000004018	AFDN	1235.69	2.32	0.07	31.56	0.00	0.00
ENSSSCG00000034348	NBEA	134.15	2.32	0.19	12.42	0.00	0.00
ENSSSCG00000036850		9.06	2.32	0.72	3.23	0.00	0.00
ENSSSCG00000011239	TRANK1	297.70	2.32	0.13	17.93	0.00	0.00
ENSSSCG00000036961	PCDHB7	164.56	2.33	0.17	13.56	0.00	0.00

ENSSSCG00000005178	CNTLN	232.70	2.33	0.15	15.70	0.00	0.00
ENSSSCG00000013079	DAGLA	207.22	2.33	0.16	14.51	0.00	0.00
ENSSSCG00000010862	CDC42BPA	839.34	2.33	0.08	27.90	0.00	0.00
ENSSSCG00000008229	SFTPB	5.02	2.33	0.96	2.43	0.02	0.02
ENSSSCG00000006344	NOS1AP	694.47	2.33	0.09	26.18	0.00	0.00
ENSSSCG00000028896	DIO1	730.10	2.33	0.10	22.49	0.00	0.00
ENSSSCG00000036572	BCO1	38.43	2.33	0.35	6.67	0.00	0.00
ENSSSCG00000010360	BMPR1A	516.43	2.33	0.11	20.40	0.00	0.00
ENSSSCG00000001983	SDR39U1	12.19	2.33	0.61	3.82	0.00	0.00
ENSSSCG00000002341	PAPLN	524.62	2.34	0.10	22.36	0.00	0.00
ENSSSCG00000004250	SLC35F1	44.76	2.34	0.33	7.12	0.00	0.00
ENSSSCG00000016174	FN1	303366.53	2.34	0.04	57.92	0.00	0.00
ENSSSCG00000004961	ITGA11	6.10	2.34	0.87	2.68	0.01	0.01
ENSSSCG00000023894	AKNA	136.55	2.34	0.19	12.16	0.00	0.00
ENSSSCG00000001865	SCAPER	142.52	2.34	0.19	12.58	0.00	0.00
ENSSSCG00000009682	HMBOX1	460.65	2.34	0.11	21.87	0.00	0.00
ENSSSCG00000011120	USP6NL	783.94	2.34	0.09	26.91	0.00	0.00
ENSSSCG00000012569	ATG4A	122.49	2.34	0.20	11.88	0.00	0.00
ENSSSCG00000003760	TTLL7	233.55	2.34	0.15	15.23	0.00	0.00
ENSSSCG00000038553	TSPO2	24.65	2.34	0.45	5.22	0.00	0.00
ENSSSCG00000000982	TTLL8	20.36	2.34	0.48	4.88	0.00	0.00
ENSSSCG00000001869	PEAK1	337.00	2.34	0.13	18.63	0.00	0.00
ENSSSCG00000006074	STK3	549.27	2.35	0.10	23.27	0.00	0.00
ENSSSCG00000038535	ARSB	244.77	2.35	0.14	16.43	0.00	0.00
ENSSSCG00000029960	LRRC4B	63.47	2.35	0.27	8.64	0.00	0.00

ENSSSCG00000025992	ENPP3	37.98	2.35	0.35	6.63	0.00	0.00
ENSSSCG00000005604	PBX3	548.46	2.35	0.10	23.55	0.00	0.00
ENSSSCG00000037914	IL17RB	4.19	2.35	1.11	2.12	0.03	0.05
ENSSSCG00000007678	COL26A1	7.21	2.36	0.80	2.93	0.00	0.01
ENSSSCG00000009101	PRDM5	108.96	2.36	0.21	11.28	0.00	0.00
ENSSSCG00000037852		40.22	2.36	0.36	6.64	0.00	0.00
ENSSSCG00000006277	SPIDR	291.00	2.36	0.14	16.89	0.00	0.00
ENSSSCG00000011522	CNTN3	64.82	2.36	0.27	8.78	0.00	0.00
ENSSSCG00000012521	GPRASP2	47.30	2.36	0.31	7.53	0.00	0.00
ENSSSCG00000040088		54.57	2.36	0.29	8.05	0.00	0.00
ENSSSCG00000003478	CROCC	76.30	2.36	0.25	9.28	0.00	0.00
ENSSSCG00000008261	HK2	3973.57	2.36	0.16	15.10	0.00	0.00
ENSSSCG00000001470	SLA-DMA	145.53	2.36	0.19	12.61	0.00	0.00
ENSSSCG00000006023	SYBU	53.94	2.36	0.30	7.77	0.00	0.00
ENSSSCG00000016494	BRAF	904.36	2.36	0.08	29.15	0.00	0.00
ENSSSCG00000017141	AATK	20.63	2.36	0.48	4.92	0.00	0.00
ENSSSCG00000028720	NXN	593.96	2.36	0.10	24.26	0.00	0.00
ENSSSCG00000007072	SPTLC3	1015.51	2.36	0.08	28.99	0.00	0.00
ENSSSCG00000023956	NEDD9	1970.73	2.37	0.06	37.80	0.00	0.00
ENSSSCG00000036926	MVB12B	91.18	2.37	0.23	10.22	0.00	0.00
ENSSSCG00000003508	KIF17	143.92	2.37	0.19	12.62	0.00	0.00
ENSSSCG00000012637	KLHL13	20.72	2.37	0.48	4.90	0.00	0.00
ENSSSCG00000008372	EHBP1	277.53	2.37	0.13	17.56	0.00	0.00
ENSSSCG00000016462	CLCN1	60.90	2.37	0.30	7.97	0.00	0.00
ENSSSCG00000013382	PLEKHA7	727.89	2.37	0.09	25.55	0.00	0.00

ENSSSCG00000022554	MATN1	4.15	2.37	1.05	2.26	0.02	0.03
ENSSSCG00000036169	UMAD1	121.48	2.37	0.21	11.36	0.00	0.00
ENSSSCG00000038958	DNM3	766.35	2.37	0.09	26.83	0.00	0.00
ENSSSCG00000027410	CBFA2T2	456.39	2.37	0.11	21.74	0.00	0.00
ENSSSCG00000023001	CCDC149	243.76	2.37	0.15	16.27	0.00	0.00
ENSSSCG00000017506	FBXL20	427.48	2.37	0.12	20.08	0.00	0.00
ENSSSCG00000004538	WDR7	288.91	2.37	0.13	17.84	0.00	0.00
ENSSSCG00000034696		61.18	2.37	0.28	8.42	0.00	0.00
ENSSSCG00000014975		5.18	2.38	0.95	2.51	0.01	0.02
ENSSSCG00000008710	JAKMIP1	124.84	2.38	0.20	12.03	0.00	0.00
ENSSSCG00000010621	ADD3	2363.54	2.38	0.06	36.75	0.00	0.00
ENSSSCG00000021588	DAPK2	56.22	2.38	0.29	8.08	0.00	0.00
ENSSSCG00000034286		9.38	2.38	0.70	3.42	0.00	0.00
ENSSSCG00000000040		160.48	2.38	0.17	13.66	0.00	0.00
ENSSSCG00000006121	PIP4P2	179.20	2.38	0.17	14.40	0.00	0.00
ENSSSCG00000033136		16.67	2.38	0.53	4.47	0.00	0.00
ENSSSCG00000010872	AKT3	335.10	2.38	0.12	19.31	0.00	0.00
ENSSSCG00000012262	KDM6A	244.64	2.38	0.14	16.56	0.00	0.00
ENSSSCG00000007454		609.69	2.39	0.10	24.78	0.00	0.00
ENSSSCG00000006316	MAEL	9.54	2.39	0.75	3.20	0.00	0.00
ENSSSCG00000008617	FAM49A	335.27	2.39	0.13	18.82	0.00	0.00
ENSSSCG00000008475	MAP4K3	987.26	2.39	0.09	27.37	0.00	0.00
ENSSSCG00000008119	KCNIP3	257.75	2.39	0.14	17.06	0.00	0.00
ENSSSCG00000021285	RNF216	443.47	2.39	0.11	21.84	0.00	0.00
ENSSSCG00000016685		24.31	2.39	0.45	5.35	0.00	0.00

ENSSSCG00000014882	RSF1	1005.02	2.40	0.08	29.71	0.00	0.00
ENSSSCG00000025194	ZSWIM6	436.80	2.40	0.12	20.76	0.00	0.00
ENSSSCG00000004242	TBC1D32	89.68	2.40	0.24	10.06	0.00	0.00
ENSSSCG00000011384	BSN	17.92	2.40	0.51	4.70	0.00	0.00
ENSSSCG00000014225	DTWD2	31.60	2.40	0.38	6.27	0.00	0.00
ENSSSCG00000016941	RNF180	85.18	2.40	0.24	10.17	0.00	0.00
ENSSSCG00000007541	PDGFA	1247.91	2.40	0.07	32.92	0.00	0.00
ENSSSCG00000037262	PTX4	346.41	2.40	0.12	19.35	0.00	0.00
ENSSSCG00000006651	ADAMTSL4	2535.40	2.40	0.13	18.20	0.00	0.00
ENSSSCG00000010151	LYST	455.03	2.40	0.12	20.62	0.00	0.00
ENSSSCG00000012075	FAM3B	15.87	2.40	0.55	4.41	0.00	0.00
ENSSSCG00000015045	NCAM1	45.51	2.40	0.32	7.44	0.00	0.00
ENSSSCG00000037987		30.84	2.41	0.41	5.89	0.00	0.00
ENSSSCG00000030303	ACHE	446.47	2.41	0.11	21.45	0.00	0.00
ENSSSCG00000032229	WDPCP	46.77	2.41	0.33	7.40	0.00	0.00
ENSSSCG00000024960	PDGFC	257.71	2.41	0.14	17.37	0.00	0.00
ENSSSCG00000005830		53.23	2.41	0.31	7.82	0.00	0.00
ENSSSCG00000000151		20.21	2.42	0.49	4.91	0.00	0.00
ENSSSCG00000003809	JAK1	5380.91	2.42	0.05	48.66	0.00	0.00
ENSSSCG00000004829	CACHD1	419.03	2.42	0.11	21.76	0.00	0.00
ENSSSCG00000014853	GDPD5	469.13	2.43	0.11	22.12	0.00	0.00
ENSSSCG00000017752	WSB1	44746.28	2.43	0.10	23.86	0.00	0.00
ENSSSCG00000016034	COL3A1	126.06	2.43	0.20	11.92	0.00	0.00
ENSSSCG00000005229	VLDLR	1963.76	2.43	0.07	32.88	0.00	0.00
ENSSSCG00000031392		62.20	2.43	0.28	8.64	0.00	0.00

ENSSSCG00000039473		1425.52	2.43	0.07	32.96	0.00	0.00
ENSSSCG00000013262	AMBRA1	588.23	2.43	0.10	24.36	0.00	0.00
ENSSSCG00000004037	MAP3K4	358.96	2.43	0.13	19.15	0.00	0.00
ENSSSCG00000003578	FGR	239.92	2.44	0.16	15.61	0.00	0.00
ENSSSCG00000010211	CCDC6	1477.79	2.44	0.07	32.82	0.00	0.00
ENSSSCG00000027415	WWOX	15.11	2.44	0.56	4.33	0.00	0.00
ENSSSCG00000005485	AMBP	52.94	2.44	0.30	8.14	0.00	0.00
ENSSSCG00000014817	ARAP1	1091.81	2.44	0.08	30.94	0.00	0.00
ENSSSCG00000017826	SMG6	315.94	2.44	0.13	19.10	0.00	0.00
ENSSSCG00000010962	FRMD3	120.51	2.45	0.20	11.99	0.00	0.00
ENSSSCG00000007370	R3HDML	3.21	2.45	1.23	2.00	0.05	0.06
ENSSSCG00000038767	MBP	201.08	2.45	0.16	15.44	0.00	0.00
ENSSSCG00000005364	TDRD7	272.85	2.45	0.14	17.41	0.00	0.00
ENSSSCG00000029558	EXTL1	122.88	2.45	0.20	12.03	0.00	0.00
ENSSSCG00000008441	ATP6V1E2	6.58	2.46	0.87	2.82	0.00	0.01
ENSSSCG00000008211		13.07	2.46	0.61	4.05	0.00	0.00
ENSSSCG00000010640	NRAP	114.76	2.46	0.21	11.90	0.00	0.00
ENSSSCG00000033629	KLHL10	3.34	2.46	1.24	1.99	0.05	0.06
ENSSSCG00000004138	HIVEP2	2427.43	2.46	0.06	39.12	0.00	0.00
ENSSSCG00000013400	MICAL2	1136.75	2.46	0.08	30.28	0.00	0.00
ENSSSCG00000032549	GNA12	317.18	2.46	0.13	19.03	0.00	0.00
ENSSSCG00000005948	TG	45.94	2.46	0.32	7.59	0.00	0.00
ENSSSCG00000024232	CCDC91	134.83	2.46	0.19	12.66	0.00	0.00
ENSSSCG00000010928	KDM5B	2724.90	2.46	0.07	34.96	0.00	0.00
ENSSSCG00000005177	SH3GL2	313.24	2.46	0.13	19.19	0.00	0.00

ENSSSCG00000033074	ZCCHC24	27.37	2.47	0.42	5.88	0.00	0.00
ENSSSCG00000034167	SLC5A3	2795.96	2.47	0.18	14.07	0.00	0.00
ENSSSCG00000025133	ITGB2	26.35	2.47	0.42	5.82	0.00	0.00
ENSSSCG00000011079	PIP4K2A	268.47	2.47	0.14	17.69	0.00	0.00
ENSSSCG00000010915	NAV1	1007.11	2.48	0.08	31.33	0.00	0.00
ENSSSCG00000037748		31.96	2.48	0.40	6.17	0.00	0.00
ENSSSCG00000021241	ADAMTS13	4.38	2.48	1.06	2.34	0.02	0.03
ENSSSCG00000008434	TTC7A	631.54	2.48	0.10	25.70	0.00	0.00
ENSSSCG00000008382	USP34	1956.10	2.48	0.07	36.87	0.00	0.00
ENSSSCG00000031875	ZNF469	254.32	2.48	0.14	17.47	0.00	0.00
ENSSSCG00000008742	CD38	89.66	2.48	0.23	10.59	0.00	0.00
ENSSSCG00000026110	SRPK2	869.94	2.48	0.08	29.65	0.00	0.00
ENSSSCG00000017131	FN3K	30.01	2.49	0.40	6.18	0.00	0.00
ENSSSCG00000028976	PPP2R2B	120.14	2.49	0.20	12.19	0.00	0.00
ENSSSCG00000029553		106.44	2.49	0.22	11.43	0.00	0.00
ENSSSCG00000009007	TMEM131L	304.55	2.49	0.14	17.91	0.00	0.00
ENSSSCG00000003512	EIF4G3	3017.18	2.49	0.06	42.34	0.00	0.00
ENSSSCG00000036446	PALD1	105.08	2.49	0.23	10.61	0.00	0.00
ENSSSCG00000009192	PDLIM5	1542.17	2.49	0.07	35.76	0.00	0.00
ENSSSCG00000010755	PTPRE	205.17	2.50	0.16	15.87	0.00	0.00
ENSSSCG00000007575	RADIL	11.13	2.50	0.67	3.72	0.00	0.00
ENSSSCG00000001709	SUPT3H	371.71	2.50	0.12	20.61	0.00	0.00
ENSSSCG00000015537	XPR1	1686.12	2.50	0.08	31.99	0.00	0.00
ENSSSCG00000033654	FANCC	35.83	2.50	0.38	6.65	0.00	0.00
ENSSSCG00000038164	RAI1	433.31	2.50	0.11	22.12	0.00	0.00

ENSSSCG00000001062	DTNBP1	55.55	2.50	0.31	7.98	0.00	0.00
ENSSSCG00000030480	DYRK1A	697.12	2.50	0.10	26.13	0.00	0.00
ENSSSCG00000031550		17.95	2.50	0.53	4.68	0.00	0.00
ENSSSCG00000028022	COL6A2	2937.45	2.50	0.06	43.37	0.00	0.00
ENSSSCG00000005934	TRAPPC9	251.95	2.50	0.15	17.19	0.00	0.00
ENSSSCG00000013252	F2	244.44	2.51	0.15	17.02	0.00	0.00
ENSSSCG00000015071	SIK3	1181.59	2.51	0.08	32.68	0.00	0.00
ENSSSCG00000038417	LRRC8D	1461.37	2.51	0.07	35.58	0.00	0.00
ENSSSCG00000016691	JAZF1	6.76	2.52	0.84	2.98	0.00	0.00
ENSSSCG00000039706		5.64	2.52	0.94	2.69	0.01	0.01
ENSSSCG00000020813	FAM20C	1401.88	2.52	0.07	35.40	0.00	0.00
ENSSSCG00000014976	ARHGAP42	633.37	2.52	0.10	25.97	0.00	0.00
ENSSSCG00000032861	NUAK1	501.82	2.52	0.11	23.38	0.00	0.00
ENSSSCG00000022290	UMODL1	4.52	2.52	1.04	2.43	0.02	0.02
ENSSSCG00000034255		3.41	2.52	1.20	2.10	0.04	0.05
ENSSSCG00000015499		253.81	2.52	0.15	16.88	0.00	0.00
ENSSSCG00000025698	SERPINE1	17457.29	2.52	0.11	22.67	0.00	0.00
ENSSSCG00000039010	WNT7A	192.41	2.52	0.16	15.33	0.00	0.00
ENSSSCG00000008459	THADA	185.03	2.53	0.17	14.88	0.00	0.00
ENSSSCG00000000588	PLEKHA5	414.39	2.53	0.11	22.16	0.00	0.00
ENSSSCG00000009090	KIAA1109	1323.81	2.53	0.08	33.16	0.00	0.00
ENSSSCG00000009994	MTMR3	1080.64	2.53	0.08	33.08	0.00	0.00
ENSSSCG00000018599	RF00007	17.01	2.53	0.56	4.55	0.00	0.00
ENSSSCG00000017139	RPTOR	324.95	2.53	0.13	19.73	0.00	0.00
ENSSSCG00000026655	PTPN13	2095.71	2.53	0.06	39.62	0.00	0.00

ENSSSCG00000024088	TLN2	606.46	2.53	0.10	25.10	0.00	0.00
ENSSSCG00000022672	TRPM4	537.12	2.53	0.11	23.37	0.00	0.00
ENSSSCG00000005078	DAAM1	630.57	2.53	0.10	25.26	0.00	0.00
ENSSSCG00000039945		3.46	2.54	1.22	2.07	0.04	0.05
ENSSSCG00000035136	SDCCAG8	33.14	2.54	0.38	6.63	0.00	0.00
ENSSSCG00000022719	RAB20	29.73	2.54	0.40	6.28	0.00	0.00
ENSSSCG00000031903	TNNT3	8.09	2.54	0.80	3.19	0.00	0.00
ENSSSCG00000031719	SEMA4B	2836.18	2.54	0.06	39.62	0.00	0.00
ENSSSCG00000004643	SLC27A2	5.73	2.54	0.91	2.78	0.01	0.01
ENSSSCG00000007564	AMZ1	29.58	2.54	0.43	5.96	0.00	0.00
ENSSSCG00000022032		1748.83	2.54	0.07	34.67	0.00	0.00
ENSSSCG00000014437	PPARGC1B	173.07	2.54	0.17	14.85	0.00	0.00
ENSSSCG00000037885		4.61	2.55	1.03	2.47	0.01	0.02
ENSSSCG00000032610		700.02	2.55	0.20	12.59	0.00	0.00
ENSSSCG00000037796		9.23	2.55	0.74	3.43	0.00	0.00
ENSSSCG00000013399	TEAD1	1010.68	2.55	0.08	32.47	0.00	0.00
ENSSSCG00000024791		9.22	2.55	0.73	3.50	0.00	0.00
ENSSSCG00000037105	EVL	107.21	2.55	0.22	11.47	0.00	0.00
ENSSSCG00000012956	PACS1	403.82	2.55	0.12	22.12	0.00	0.00
ENSSSCG00000027074		41.39	2.55	0.35	7.28	0.00	0.00
ENSSSCG00000004952	SMAD3	1059.03	2.55	0.08	32.65	0.00	0.00
ENSSSCG00000007066	SLX4IP	55.42	2.55	0.31	8.28	0.00	0.00
ENSSSCG00000010563	BTRC	456.87	2.56	0.11	23.13	0.00	0.00
ENSSSCG00000021466	BMT2	493.77	2.56	0.12	21.97	0.00	0.00
ENSSSCG00000037556	COG5	431.29	2.56	0.12	21.86	0.00	0.00

ENSSSCG00000033483		16.27	2.56	0.55	4.65	0.00	0.00
ENSSSCG00000010549		17.29	2.56	0.55	4.65	0.00	0.00
ENSSSCG00000003663	HPCAL1	65.10	2.56	0.29	8.95	0.00	0.00
ENSSSCG00000003736	ASXL3	150.01	2.56	0.19	13.60	0.00	0.00
ENSSSCG00000002664	GSE1	927.39	2.57	0.09	30.08	0.00	0.00
ENSSSCG00000009134	EGF	16.09	2.57	0.60	4.30	0.00	0.00
ENSSSCG00000002720		18.59	2.57	0.52	4.93	0.00	0.00
ENSSSCG00000011760	TBL1XR1	1593.11	2.57	0.08	33.55	0.00	0.00
ENSSSCG00000002815	ADGRG1	3378.48	2.57	0.06	46.59	0.00	0.00
ENSSSCG00000029257		26.81	2.57	0.43	6.03	0.00	0.00
ENSSSCG00000007951	CREBBP	1657.62	2.57	0.07	38.71	0.00	0.00
ENSSSCG00000038126	MGAT3	130.74	2.57	0.20	12.66	0.00	0.00
ENSSSCG00000040466	FMN1	106.45	2.57	0.22	11.83	0.00	0.00
ENSSSCG00000016182	RUFY4	31.56	2.58	0.40	6.42	0.00	0.00
ENSSSCG00000007720	GTF2IRD1	353.74	2.58	0.12	20.87	0.00	0.00
ENSSSCG00000035392	IGFBP2	10318.93	2.58	0.05	51.83	0.00	0.00
ENSSSCG00000040611	CPSF4L	7.05	2.58	0.84	3.08	0.00	0.00
ENSSSCG00000008144	NCK2	1520.70	2.58	0.07	37.53	0.00	0.00
ENSSSCG00000009408	LRCH1	285.31	2.58	0.14	18.34	0.00	0.00
ENSSSCG00000005946	CCN4	31.70	2.58	0.40	6.49	0.00	0.00
ENSSSCG00000016824	RAI14	2229.71	2.58	0.06	40.76	0.00	0.00
ENSSSCG00000029196	DIP2B	621.91	2.58	0.10	25.42	0.00	0.00
ENSSSCG00000027275	HHLA2	11.82	2.59	0.66	3.94	0.00	0.00
ENSSSCG00000004561	HERC1	1468.54	2.59	0.07	36.52	0.00	0.00
ENSSSCG00000038437	EHMT1	596.95	2.59	0.10	26.10	0.00	0.00

ENSSSCG00000009448	DIAPH3	291.39	2.59	0.14	18.39	0.00	0.00
ENSSSCG00000032078	ZNF362	143.86	2.59	0.19	13.37	0.00	0.00
ENSSSCG00000016068	HECW2	82.82	2.59	0.25	10.21	0.00	0.00
ENSSSCG00000004387	FOXO3	1339.46	2.59	0.09	30.19	0.00	0.00
ENSSSCG00000009018	SH3D19	655.61	2.59	0.10	26.54	0.00	0.00
ENSSSCG00000034704	ZNF532	27.14	2.59	0.43	6.09	0.00	0.00
ENSSSCG00000035607	PRM3	5.91	2.60	0.91	2.85	0.00	0.01
ENSSSCG00000023709	PTPRJ	2046.66	2.60	0.07	38.38	0.00	0.00
ENSSSCG00000023018	MCCD1	118.57	2.60	0.21	12.46	0.00	0.00
ENSSSCG00000032843		385.88	2.60	0.12	21.66	0.00	0.00
ENSSSCG00000004249	CEP85L	114.20	2.60	0.22	11.91	0.00	0.00
ENSSSCG00000025087	ASPH	2524.60	2.60	0.06	45.12	0.00	0.00
ENSSSCG00000014406	PRELID2	894.22	2.60	0.08	30.79	0.00	0.00
ENSSSCG00000019803	RF00019	8.34	2.61	0.77	3.39	0.00	0.00
ENSSSCG00000009370	FOXO1	723.09	2.61	0.09	27.70	0.00	0.00
ENSSSCG00000035414	XXYLT1	66.97	2.61	0.28	9.37	0.00	0.00
ENSSSCG00000000113	SLC16A8	3.60	2.61	1.18	2.22	0.03	0.04
ENSSSCG00000016141	PLEKHM3	15.48	2.61	0.59	4.46	0.00	0.00
ENSSSCG00000035218	ADA2	383.71	2.61	0.12	22.02	0.00	0.00
ENSSSCG00000037614	RF01957	1569.25	2.61	0.17	15.33	0.00	0.00
ENSSSCG00000014267		689.89	2.61	0.10	26.33	0.00	0.00
ENSSSCG00000009716	SH3RF1	1120.98	2.62	0.08	32.05	0.00	0.00
ENSSSCG00000003463	AGMAT	687.06	2.62	0.10	27.35	0.00	0.00
ENSSSCG00000038766	MAD1L1	54.01	2.62	0.31	8.57	0.00	0.00
ENSSSCG00000035876	ZNF599	6.00	2.62	0.94	2.79	0.01	0.01

ENSSSCG00000001621	TFEB	207.85	2.62	0.16	16.40	0.00	0.00
ENSSSCG00000012197		57.81	2.62	0.29	8.92	0.00	0.00
ENSSSCG00000013363	LDHC	16.75	2.62	0.57	4.63	0.00	0.00
ENSSSCG00000038851		4.84	2.63	1.02	2.58	0.01	0.01
ENSSSCG00000010210	SLC16A9	43.66	2.63	0.35	7.48	0.00	0.00
ENSSSCG00000028488	LTC4S	63.11	2.63	0.29	9.19	0.00	0.00
ENSSSCG00000040629	IL34	139.31	2.64	0.20	13.42	0.00	0.00
ENSSSCG00000025523	COL2A1	41.25	2.64	0.35	7.55	0.00	0.00
ENSSSCG00000006956	ZC3H3	312.79	2.64	0.14	19.47	0.00	0.00
ENSSSCG00000011609	FBLN2	10.95	2.64	0.68	3.88	0.00	0.00
ENSSSCG00000011025	ZEB1	114.44	2.64	0.22	12.03	0.00	0.00
ENSSSCG00000026816		20.54	2.64	0.51	5.20	0.00	0.00
ENSSSCG00000007080		216.06	2.65	0.16	16.45	0.00	0.00
ENSSSCG00000016113	BMPR2	1492.32	2.65	0.08	34.34	0.00	0.00
ENSSSCG00000002257	MCTP2	108.69	2.65	0.22	12.00	0.00	0.00
ENSSSCG00000028117		237.59	2.65	0.15	17.57	0.00	0.00
ENSSSCG00000016555	CPA5	182.98	2.66	0.18	15.08	0.00	0.00
ENSSSCG00000030696	SLC19A1	280.48	2.66	0.14	18.95	0.00	0.00
ENSSSCG00000003129	ZSWIM9	24.70	2.67	0.46	5.76	0.00	0.00
ENSSSCG00000030626	ALDH1L1	171.54	2.67	0.18	15.20	0.00	0.00
ENSSSCG00000004384	NR2E1	27.01	2.67	0.45	5.94	0.00	0.00
ENSSSCG00000005037	ERO1A	13528.95	2.67	0.13	20.03	0.00	0.00
ENSSSCG00000001065	ATXN1	208.42	2.67	0.16	16.59	0.00	0.00
ENSSSCG00000011379	KLHDC8B	6.23	2.67	0.93	2.89	0.00	0.01
ENSSSCG00000026537	CACNA2D4	18.65	2.68	0.52	5.11	0.00	0.00

ENSSSCG00000033580	C17orf50	23.68	2.68	0.47	5.67	0.00	0.00
ENSSSCG00000000770	MICAL3	350.65	2.68	0.13	20.30	0.00	0.00
ENSSSCG00000030153	SMURF1	1131.06	2.68	0.08	32.56	0.00	0.00
ENSSSCG00000026653		3.77	2.68	1.16	2.31	0.02	0.03
ENSSSCG00000010992		267.15	2.69	0.15	17.94	0.00	0.00
ENSSSCG00000001639	TRERF1	290.79	2.69	0.28	9.47	0.00	0.00
ENSSSCG00000020717	FAM160A1	121.70	2.69	0.21	12.87	0.00	0.00
ENSSSCG00000004948	SMAD6	101.28	2.69	0.24	11.34	0.00	0.00
ENSSSCG00000001710	RUNX2	300.09	2.70	0.14	19.78	0.00	0.00
ENSSSCG00000008511		15.11	2.70	0.59	4.58	0.00	0.00
ENSSSCG00000008621	NBAS	371.14	2.70	0.12	21.94	0.00	0.00
ENSSSCG00000008522	XDH	10.11	2.70	0.72	3.73	0.00	0.00
ENSSSCG00000000260	SOAT2	311.04	2.70	0.13	20.34	0.00	0.00
ENSSSCG00000039317	SLC25A21	40.22	2.70	0.38	7.16	0.00	0.00
ENSSSCG00000007950	ADCY9	266.78	2.70	0.15	17.72	0.00	0.00
ENSSSCG00000012253		350.40	2.70	0.13	20.92	0.00	0.00
ENSSSCG00000011514	MITF	511.16	2.70	0.11	24.60	0.00	0.00
ENSSSCG00000010765	INPP5A	44.00	2.70	0.35	7.76	0.00	0.00
ENSSSCG00000020736	ADD2	5.10	2.70	1.02	2.66	0.01	0.01
ENSSSCG00000009850	TAOK3	437.73	2.71	0.12	23.37	0.00	0.00
ENSSSCG00000014223	COMMD10	290.32	2.71	0.14	19.49	0.00	0.00
ENSSSCG00000027922	UNC5CL	1721.59	2.71	0.07	37.28	0.00	0.00
ENSSSCG00000013566	INSR	405.38	2.71	0.13	20.56	0.00	0.00
ENSSSCG00000008813	CORIN	116.23	2.71	0.23	11.68	0.00	0.00
ENSSSCG00000014672		58.46	2.71	0.30	9.15	0.00	0.00

ENSSSCG00000036553	SAMD12	17.84	2.72	0.54	5.01	0.00	0.00
ENSSSCG00000004234	FABP7	12.79	2.72	0.64	4.22	0.00	0.00
ENSSSCG00000003712	OSBPL1A	475.22	2.72	0.11	24.68	0.00	0.00
ENSSSCG00000039171	MRTFB	1196.44	2.72	0.08	35.02	0.00	0.00
ENSSSCG00000014851	SLCO2B1	129.85	2.72	0.21	13.25	0.00	0.00
ENSSSCG00000013150		154.86	2.72	0.20	13.88	0.00	0.00
ENSSSCG00000011599	GRIP2	351.01	2.72	0.13	20.63	0.00	0.00
ENSSSCG00000022159	FNDC3A	1697.55	2.72	0.08	34.63	0.00	0.00
ENSSSCG00000003863	ZFYVE9	556.63	2.73	0.11	25.18	0.00	0.00
ENSSSCG00000038041	FYB2	769.57	2.73	0.10	27.92	0.00	0.00
ENSSSCG00000016473	EPHB6	5.13	2.73	1.00	2.73	0.01	0.01
ENSSSCG00000015631	GRB10	860.05	2.73	0.09	30.91	0.00	0.00
ENSSSCG00000023057	LMO1	21.76	2.73	0.50	5.51	0.00	0.00
ENSSSCG00000000145	MYH9	14686.36	2.73	0.04	61.51	0.00	0.00
ENSSSCG00000026453	ACSM5	51.42	2.73	0.32	8.61	0.00	0.00
ENSSSCG00000003694	EMILIN2	8008.67	2.73	0.05	58.95	0.00	0.00
ENSSSCG00000000110	PLA2G6	218.64	2.74	0.16	17.07	0.00	0.00
ENSSSCG00000039629		5.22	2.74	1.03	2.67	0.01	0.01
ENSSSCG00000007901	CIITA	154.88	2.74	0.20	14.01	0.00	0.00
ENSSSCG00000005487	COL27A1	95.36	2.74	0.24	11.53	0.00	0.00
ENSSSCG00000031943		145.93	2.74	0.19	14.06	0.00	0.00
ENSSSCG00000038698	RF00614	7.73	2.74	0.83	3.31	0.00	0.00
ENSSSCG00000003877	FAF1	442.77	2.74	0.12	23.57	0.00	0.00
ENSSSCG00000028148	DMD	126.93	2.74	0.21	13.15	0.00	0.00
ENSSSCG00000011133	PFKFB3	5792.39	2.74	0.07	40.32	0.00	0.00

ENSSSCG00000039626	DERL3	29.89	2.74	0.43	6.32	0.00	0.00
ENSSSCG00000006163	PKIA	178.36	2.74	0.18	15.45	0.00	0.00
ENSSSCG00000015878	PKP4	646.97	2.74	0.10	28.28	0.00	0.00
ENSSSCG00000005346	ZCCHC7	142.18	2.75	0.20	13.88	0.00	0.00
ENSSSCG00000014813	PHOX2A	11.74	2.75	0.67	4.09	0.00	0.00
ENSSSCG00000008340	ANTXR1	160.70	2.75	0.18	15.10	0.00	0.00
ENSSSCG00000013303	ABTB2	362.71	2.75	0.13	20.77	0.00	0.00
ENSSSCG00000011833	DLG1	1537.40	2.75	0.07	38.44	0.00	0.00
ENSSSCG00000026996		1522.37	2.76	0.07	37.13	0.00	0.00
ENSSSCG00000033260	TNNI2	186.41	2.76	0.17	16.20	0.00	0.00
ENSSSCG00000025086		18.25	2.76	0.56	4.95	0.00	0.00
ENSSSCG00000024377	RPS6KA5	382.55	2.77	0.12	22.14	0.00	0.00
ENSSSCG00000010466	FGFBP3	51.11	2.77	0.32	8.52	0.00	0.00
ENSSSCG00000002456	CHGA	2.63	2.77	1.40	1.98	0.05	0.06
ENSSSCG00000000755	ERC1	796.40	2.77	0.09	30.78	0.00	0.00
ENSSSCG00000031888	DDIT4	3713.95	2.77	0.15	18.75	0.00	0.00
ENSSSCG00000005393	PLPPR1	9.29	2.78	0.76	3.67	0.00	0.00
ENSSSCG00000009676	ZNF395	1726.30	2.78	0.18	15.79	0.00	0.00
ENSSSCG00000011416	DOCK3	3.94	2.78	1.16	2.40	0.02	0.02
ENSSSCG00000015238	ARHGAP32	737.89	2.78	0.10	28.92	0.00	0.00
ENSSSCG00000008881	RAPGEF2	460.97	2.78	0.11	24.37	0.00	0.00
ENSSSCG00000006231	CHD7	944.10	2.78	0.09	29.41	0.00	0.00
ENSSSCG00000025294	AIRE	58.19	2.79	0.30	9.14	0.00	0.00
ENSSSCG00000017310	KANSL1	1144.56	2.79	0.08	34.47	0.00	0.00
ENSSSCG00000014960	AMOTL1	21.15	2.79	0.51	5.42	0.00	0.00

ENSSSCG00000015707	GPR39	140.06	2.79	0.20	13.71	0.00	0.00
ENSSSCG00000021527	ATP10B	8.03	2.80	0.81	3.45	0.00	0.00
ENSSSCG00000008625	NTSR2	5.34	2.80	1.00	2.79	0.01	0.01
ENSSSCG00000006294	NME7	213.96	2.80	0.17	16.89	0.00	0.00
ENSSSCG00000030274	PPEF1	110.22	2.80	0.23	12.33	0.00	0.00
ENSSSCG00000038058	RAD51AP2	5.41	2.80	1.02	2.75	0.01	0.01
ENSSSCG00000028969	SLC26A4	5.37	2.80	1.00	2.80	0.01	0.01
ENSSSCG00000025423	KCNK5	1880.83	2.80	0.07	42.76	0.00	0.00
ENSSSCG00000028204	TREH	160.17	2.81	0.37	7.54	0.00	0.00
ENSSSCG00000015694	ZRANB3	198.06	2.81	0.17	16.28	0.00	0.00
ENSSSCG00000033137		4.10	2.81	1.20	2.35	0.02	0.03
ENSSSCG00000026221		12.14	2.81	0.66	4.28	0.00	0.00
ENSSSCG00000007305	SPAG4	109.40	2.82	0.22	12.63	0.00	0.00
ENSSSCG00000016513	KIAA1549	171.63	2.82	0.18	15.54	0.00	0.00
ENSSSCG00000029811	PLCL2	4.10	2.82	1.15	2.45	0.01	0.02
ENSSSCG00000029521	CD82	58.47	2.83	0.30	9.31	0.00	0.00
ENSSSCG00000022447	F3	9584.54	2.83	0.14	20.63	0.00	0.00
ENSSSCG00000011495	PRICKLE2	32.71	2.83	0.40	6.99	0.00	0.00
ENSSSCG00000030485	ELFN1	24.66	2.84	0.48	5.88	0.00	0.00
ENSSSCG00000000735	TSPAN9	59.92	2.84	0.31	9.06	0.00	0.00
ENSSSCG00000001080	CDKAL1	30.23	2.84	0.43	6.63	0.00	0.00
ENSSSCG00000025176	NOTCH3	61.81	2.84	0.30	9.49	0.00	0.00
ENSSSCG00000029949	CD248	6.87	2.84	0.89	3.20	0.00	0.00
ENSSSCG00000010302	USP54	438.71	2.84	0.12	24.34	0.00	0.00
ENSSSCG00000023915	SLC2A4	6.89	2.84	0.94	3.02	0.00	0.00

ENSSSCG00000007099	SCP2D1	5.52	2.85	0.99	2.87	0.00	0.01
ENSSSCG00000015882	BAZ2B	403.04	2.85	0.12	23.23	0.00	0.00
ENSSSCG00000004795	MEIS2	83.98	2.85	0.26	10.81	0.00	0.00
ENSSSCG00000033312		27.60	2.85	0.45	6.33	0.00	0.00
ENSSSCG00000004415		29.05	2.85	0.43	6.60	0.00	0.00
ENSSSCG00000021158	MCF2L	110.64	2.85	0.23	12.40	0.00	0.00
ENSSSCG00000014213	KCNN2	56.58	2.86	0.33	8.76	0.00	0.00
ENSSSCG00000003218	MYBPC2	69.25	2.86	0.28	10.14	0.00	0.00
ENSSSCG00000022126	EGFR	2854.32	2.86	0.06	45.87	0.00	0.00
ENSSSCG00000004033	AGPAT4	196.90	2.86	0.17	16.94	0.00	0.00
ENSSSCG00000000783	SLC2A13	222.80	2.86	0.16	17.68	0.00	0.00
ENSSSCG00000031174	FBP1	47.41	2.87	0.34	8.50	0.00	0.00
ENSSSCG00000011085	MLLT10	456.92	2.87	0.12	24.55	0.00	0.00
ENSSSCG00000038066	USH2A	76.60	2.87	0.27	10.64	0.00	0.00
ENSSSCG00000015476	CHI3L1	2.77	2.87	1.38	2.08	0.04	0.05
ENSSSCG00000013440	ATP8B3	433.80	2.87	0.12	24.32	0.00	0.00
ENSSSCG00000029815	SRGAP1	214.21	2.87	0.16	17.73	0.00	0.00
ENSSSCG00000004902	RNF152	35.05	2.88	0.40	7.21	0.00	0.00
ENSSSCG00000024476	CES3	1717.56	2.88	0.07	42.84	0.00	0.00
ENSSSCG00000033759	TBXA2R	14.00	2.88	0.63	4.59	0.00	0.00
ENSSSCG00000025097	TMEM61	29.58	2.88	0.44	6.48	0.00	0.00
ENSSSCG00000008464	MTA3	190.94	2.88	0.17	16.83	0.00	0.00
ENSSSCG00000028922	GABRA2	8.44	2.88	0.80	3.59	0.00	0.00
ENSSSCG00000006038	ZFPM2	40.80	2.88	0.37	7.89	0.00	0.00
ENSSSCG00000012298	CACNA1F	25.36	2.88	0.47	6.18	0.00	0.00

ENSSSCG00000022039		98.68	2.89	0.24	12.08	0.00	0.00
ENSSSCG00000032137	PCDHGA9	2.82	2.89	1.40	2.06	0.04	0.05
ENSSSCG00000006503	RXFP4	2.85	2.89	1.39	2.08	0.04	0.05
ENSSSCG00000032423	SPOCK1	14.09	2.89	0.62	4.64	0.00	0.00
ENSSSCG00000016956	MAST4	188.63	2.90	0.18	16.46	0.00	0.00
ENSSSCG00000012634	DOCK11	21.20	2.90	0.51	5.67	0.00	0.00
ENSSSCG00000012054	DOP1B	1039.51	2.90	0.08	35.33	0.00	0.00
ENSSSCG00000016330		1933.20	2.90	0.07	40.17	0.00	0.00
ENSSSCG00000039651	SLC2A5	648.21	2.91	0.10	28.95	0.00	0.00
ENSSSCG00000019963	RF00001	15.71	2.91	0.60	4.87	0.00	0.00
ENSSSCG00000023010	TMOD1	5.72	2.91	0.98	2.97	0.00	0.00
ENSSSCG00000009364	FREM2	20.14	2.91	0.54	5.44	0.00	0.00
ENSSSCG00000035977		2.89	2.91	1.44	2.02	0.04	0.06
ENSSSCG00000029163	BCAT1	6263.38	2.91	0.06	48.53	0.00	0.00
ENSSSCG00000009664	PTK2B	1357.43	2.91	0.08	38.10	0.00	0.00
ENSSSCG00000006767	MAGI3	692.17	2.92	0.10	28.91	0.00	0.00
ENSSSCG00000002950	SIPA1L3	369.85	2.92	0.14	20.95	0.00	0.00
ENSSSCG00000009953		171.85	2.92	0.18	15.88	0.00	0.00
ENSSSCG00000007814	KIAA0556	59.09	2.93	0.31	9.47	0.00	0.00
ENSSSCG00000016247	TM4SF20	17.36	2.93	0.56	5.19	0.00	0.00
ENSSSCG00000035395		7.21	2.93	0.87	3.38	0.00	0.00
ENSSSCG00000000766	CECR2	244.44	2.93	0.16	18.45	0.00	0.00
ENSSSCG00000001867	PSTPIP1	23.31	2.94	0.49	6.02	0.00	0.00
ENSSSCG00000013868	F2RL3	17.55	2.94	0.57	5.12	0.00	0.00
ENSSSCG00000039157	CLASP1	954.26	2.94	0.09	31.48	0.00	0.00

ENSSSCG00000033608	LOXL2	14499.38	2.94	0.05	62.96	0.00	0.00
ENSSSCG00000009874	RASAL1	73.21	2.95	0.28	10.62	0.00	0.00
ENSSSCG00000013042		45.82	2.96	0.35	8.44	0.00	0.00
ENSSSCG00000015880	TANC1	845.97	2.96	0.09	32.58	0.00	0.00
ENSSSCG00000016035	COL5A2	5.95	2.96	0.98	3.03	0.00	0.00
ENSSSCG00000029745	FAM193A	328.25	2.96	0.14	21.40	0.00	0.00
ENSSSCG00000033707		2.97	2.96	1.36	2.18	0.03	0.04
ENSSSCG00000035267		10.36	2.96	0.73	4.06	0.00	0.00
ENSSSCG00000028979	UVRAG	237.83	2.96	0.16	18.81	0.00	0.00
ENSSSCG00000007512		252.91	2.96	0.15	19.27	0.00	0.00
ENSSSCG00000028923	SCNN1B	200.26	2.97	0.17	16.99	0.00	0.00
ENSSSCG00000005358		618.00	2.97	0.11	27.67	0.00	0.00
ENSSSCG00000011499	LRIG1	108.18	2.97	0.23	12.92	0.00	0.00
ENSSSCG00000005098	SYT16	232.08	2.97	0.16	18.54	0.00	0.00
ENSSSCG00000005762	KCNT1	4.44	2.98	1.14	2.61	0.01	0.01
ENSSSCG00000008593	KLHL29	147.54	2.98	0.21	14.02	0.00	0.00
ENSSSCG00000022636	DENND5B	407.15	2.98	0.13	22.28	0.00	0.00
ENSSSCG00000021557	SULT1A3	13.46	2.98	0.66	4.54	0.00	0.00
ENSSSCG00000033469		16.51	2.98	0.60	5.00	0.00	0.00
ENSSSCG00000026486		40.47	2.98	0.38	7.86	0.00	0.00
ENSSSCG00000001910	ISLR	619.87	2.98	0.11	27.74	0.00	0.00
ENSSSCG00000012434	ATRX	1726.30	2.99	0.07	41.10	0.00	0.00
ENSSSCG00000034329	NAT14	9.04	2.99	0.79	3.81	0.00	0.00
ENSSSCG00000027745	ABCG1	1961.98	3.00	0.07	45.49	0.00	0.00
ENSSSCG00000028047	DTNA	420.90	3.00	0.13	23.99	0.00	0.00

ENSSSCG00000038684		15.24	3.01	0.61	4.93	0.00	0.00
ENSSSCG00000040728	EYA1	38.01	3.01	0.39	7.67	0.00	0.00
ENSSSCG00000016725	TNS3	29.00	3.01	0.44	6.78	0.00	0.00
ENSSSCG00000004044	IGF2R	11627.40	3.01	0.05	65.89	0.00	0.00
ENSSSCG00000005224	GLIS3	364.02	3.01	0.13	22.77	0.00	0.00
ENSSSCG00000015307	CDK14	347.83	3.01	0.14	21.44	0.00	0.00
ENSSSCG00000014136	VCAN	108.46	3.02	0.24	12.82	0.00	0.00
ENSSSCG00000009123	CAMK2D	753.51	3.02	0.10	30.44	0.00	0.00
ENSSSCG00000015906	CSRNP3	20.09	3.03	0.54	5.63	0.00	0.00
ENSSSCG00000000734	TSPAN11	7.76	3.03	0.89	3.41	0.00	0.00
ENSSSCG00000007878	PARN	287.70	3.03	0.15	19.88	0.00	0.00
ENSSSCG00000025308	IL17D	132.80	3.04	0.21	14.14	0.00	0.00
ENSSSCG00000034879	MAML2	122.21	3.04	0.23	13.48	0.00	0.00
ENSSSCG00000001021	RREB1	563.44	3.04	0.11	27.70	0.00	0.00
ENSSSCG00000024517	AKAP6	340.89	3.04	0.14	22.26	0.00	0.00
ENSSSCG00000004600	TCF12	759.41	3.04	0.10	31.14	0.00	0.00
ENSSSCG00000015667	MBD5	65.02	3.04	0.30	10.20	0.00	0.00
ENSSSCG00000004379	SOBP	69.50	3.04	0.29	10.42	0.00	0.00
ENSSSCG00000015846	RBPMS	1159.65	3.04	0.09	33.50	0.00	0.00
ENSSSCG00000009761	NCOR2	5943.54	3.04	0.06	55.10	0.00	0.00
ENSSSCG00000008769		6.23	3.05	0.96	3.16	0.00	0.00
ENSSSCG00000017324		12.42	3.05	0.68	4.51	0.00	0.00
ENSSSCG00000005325	TMEM8B	111.96	3.05	0.23	13.28	0.00	0.00
ENSSSCG00000008820	TEC	412.17	3.05	0.13	23.49	0.00	0.00
ENSSSCG00000033685	NOXA1	3.13	3.05	1.35	2.26	0.02	0.03

ENSSSCG00000016317	AGAP1	184.17	3.05	0.18	16.74	0.00	0.00
ENSSSCG00000016497	DENND2A	168.98	3.05	0.19	16.07	0.00	0.00
ENSSSCG00000039769		1010.33	3.06	0.09	34.71	0.00	0.00
ENSSSCG00000011496	ADAMTS9	164.92	3.06	0.19	15.74	0.00	0.00
ENSSSCG00000036531		194.68	3.06	0.18	17.44	0.00	0.00
ENSSSCG00000023187		1078.31	3.07	0.18	17.22	0.00	0.00
ENSSSCG00000013283	ACCSL	9.49	3.07	0.78	3.92	0.00	0.00
ENSSSCG00000016328	RAB17	72.52	3.07	0.29	10.74	0.00	0.00
ENSSSCG00000011259	SCN5A	146.94	3.07	0.20	15.07	0.00	0.00
ENSSSCG00000023090	BEST1	45.76	3.07	0.36	8.54	0.00	0.00
ENSSSCG00000014909		2279.04	3.07	0.07	46.29	0.00	0.00
ENSSSCG00000006737	IGSF3	211.90	3.07	0.17	17.79	0.00	0.00
ENSSSCG00000008310	EXOC6B	153.96	3.08	0.20	15.52	0.00	0.00
ENSSSCG00000032422		563.58	3.08	0.11	27.50	0.00	0.00
ENSSSCG00000029392	HUNK	364.22	3.08	0.14	22.66	0.00	0.00
ENSSSCG00000031776	TULP4	81.26	3.08	0.27	11.46	0.00	0.00
ENSSSCG00000035949	FTO	112.43	3.10	0.23	13.26	0.00	0.00
ENSSSCG00000004123	UTRN	1895.89	3.10	0.08	39.20	0.00	0.00
ENSSSCG00000009517	GPR183	54.58	3.10	0.33	9.40	0.00	0.00
ENSSSCG00000017636	LPO	33.76	3.10	0.42	7.42	0.00	0.00
ENSSSCG00000023936	MYRFL	3.17	3.10	1.38	2.25	0.02	0.03
ENSSSCG00000020590	RF00100	4.80	3.10	1.12	2.76	0.01	0.01
ENSSSCG00000011209		248.09	3.11	0.16	19.45	0.00	0.00
ENSSSCG00000038397	PTK2	1126.51	3.11	0.08	36.97	0.00	0.00
ENSSSCG00000011962	ADGRG7	51.71	3.11	0.34	9.15	0.00	0.00

ENSSSCG00000006321	FAM78B	8.13	3.11	0.87	3.57	0.00	0.00
ENSSSCG00000031389		3.20	3.11	1.39	2.24	0.02	0.04
ENSSSCG00000037706	PRKAR2B	92.22	3.11	0.26	12.04	0.00	0.00
ENSSSCG00000002427		202.42	3.11	0.18	17.77	0.00	0.00
ENSSSCG00000039793	PATJ	975.16	3.11	0.09	35.86	0.00	0.00
ENSSSCG00000023974	PHF21A	532.25	3.12	0.12	26.23	0.00	0.00
ENSSSCG00000022128	MXI1	1219.21	3.12	0.09	34.06	0.00	0.00
ENSSSCG00000003731	GAREM1	259.06	3.12	0.16	19.23	0.00	0.00
ENSSSCG00000002170		4.91	3.12	1.10	2.84	0.00	0.01
ENSSSCG00000009112	SEC24D	534.87	3.12	0.11	27.66	0.00	0.00
ENSSSCG00000010241	TET1	178.40	3.12	0.19	16.20	0.00	0.00
ENSSSCG00000039027	TRO	9.82	3.13	0.77	4.03	0.00	0.00
ENSSSCG00000001455		3.27	3.13	1.33	2.35	0.02	0.03
ENSSSCG00000014224	SEMA6A	122.77	3.13	0.22	13.99	0.00	0.00
ENSSSCG00000007346	ADIG	11.47	3.13	0.72	4.36	0.00	0.00
ENSSSCG00000014053	SNCB	6.63	3.14	0.94	3.33	0.00	0.00
ENSSSCG00000003975	KCNQ4	24.67	3.14	0.50	6.33	0.00	0.00
ENSSSCG00000002285	GPHN	170.14	3.14	0.19	16.41	0.00	0.00
ENSSSCG00000013297	CD44	10138.20	3.14	0.05	65.97	0.00	0.00
ENSSSCG00000013882	FAM129C	3.28	3.14	1.35	2.32	0.02	0.03
ENSSSCG00000027935	FHOD3	29.76	3.15	0.45	6.92	0.00	0.00
ENSSSCG00000033538	SMLR1	8.34	3.15	0.86	3.64	0.00	0.00
ENSSSCG00000009037	ZNF827	314.17	3.15	0.15	21.58	0.00	0.00
ENSSSCG00000035456	WWC1	815.17	3.15	0.10	31.76	0.00	0.00
ENSSSCG00000006035	ANGPT1	3.33	3.16	1.34	2.36	0.02	0.03

ENSSSCG00000007252	DNMT3B	617.20	3.16	0.11	29.16	0.00	0.00
ENSSSCG00000036788		19.97	3.16	0.56	5.68	0.00	0.00
ENSSSCG00000031441	GRAMD4	305.04	3.16	0.15	21.64	0.00	0.00
ENSSSCG00000000075	MRTFA	681.00	3.16	0.10	31.42	0.00	0.00
ENSSSCG00000021791	SENP7	201.65	3.16	0.18	17.40	0.00	0.00
ENSSSCG00000006235	TOX	78.37	3.16	0.29	10.77	0.00	0.00
ENSSSCG00000010358	OPN4	180.99	3.17	0.19	16.74	0.00	0.00
ENSSSCG00000017117	SLC6A18	11.87	3.17	0.72	4.38	0.00	0.00
ENSSSCG00000008943	SLC4A4	702.77	3.18	0.11	29.75	0.00	0.00
ENSSSCG00000023322		5.05	3.18	1.09	2.92	0.00	0.01
ENSSSCG00000017885	SMTNL2	724.73	3.18	0.10	32.37	0.00	0.00
ENSSSCG00000009942	DAO	176.84	3.19	0.20	16.07	0.00	0.00
ENSSSCG00000009765	DNAH10	426.60	3.19	0.13	25.19	0.00	0.00
ENSSSCG00000032271		8.47	3.19	0.84	3.80	0.00	0.00
ENSSSCG00000022486	CBLB	293.67	3.19	0.15	21.27	0.00	0.00
ENSSSCG00000027796	SLC22A7	22.19	3.20	0.52	6.11	0.00	0.00
ENSSSCG00000022830	KANSL1L	209.88	3.20	0.19	16.68	0.00	0.00
ENSSSCG00000022989	ZNF704	3.42	3.20	1.33	2.41	0.02	0.02
ENSSSCG00000027556		145.42	3.20	0.21	15.43	0.00	0.00
ENSSSCG00000040010	BCL2A1	12.00	3.20	0.71	4.51	0.00	0.00
ENSSSCG00000001553	LHFPL5	6.90	3.20	0.95	3.38	0.00	0.00
ENSSSCG00000008826	FRYL	411.62	3.21	0.13	25.19	0.00	0.00
ENSSSCG00000030305	SLC15A2	36.29	3.21	0.42	7.69	0.00	0.00
ENSSSCG00000024678	MEGF6	934.73	3.21	0.09	35.93	0.00	0.00
ENSSSCG00000009230	WDFY3	1417.37	3.22	0.08	40.46	0.00	0.00

ENSSSCG00000016690	CREB5	317.90	3.22	0.15	22.06	0.00	0.00
ENSSSCG00000030652		6.95	3.22	0.93	3.45	0.00	0.00
ENSSSCG00000011663	RBP2	236.64	3.23	0.17	18.86	0.00	0.00
ENSSSCG00000006009	EXT1	2114.44	3.23	0.07	46.38	0.00	0.00
ENSSSCG00000016033	GULP1	24.41	3.23	0.50	6.44	0.00	0.00
ENSSSCG00000002457	ITPK1	427.34	3.23	0.13	25.52	0.00	0.00
ENSSSCG00000009885	HECTD4	964.18	3.24	0.09	36.10	0.00	0.00
ENSSSCG00000009348	STARD13	447.86	3.24	0.13	25.13	0.00	0.00
ENSSSCG00000040272	TTC29	76.94	3.24	0.29	11.33	0.00	0.00
ENSSSCG00000040896		5.30	3.24	1.08	3.00	0.00	0.00
ENSSSCG00000033893		17.50	3.24	0.61	5.29	0.00	0.00
ENSSSCG00000038226		188.03	3.24	0.41	7.93	0.00	0.00
ENSSSCG00000013261	CHRM4	10.59	3.25	0.77	4.19	0.00	0.00
ENSSSCG00000010638	TCF7L2	259.51	3.25	0.16	20.17	0.00	0.00
ENSSSCG00000001101	SCGN	133.33	3.26	0.22	14.67	0.00	0.00
ENSSSCG00000012164	CNKSR2	206.61	3.26	0.18	18.25	0.00	0.00
ENSSSCG00000005020	ATL1	19.72	3.27	0.57	5.77	0.00	0.00
ENSSSCG00000010148	ERO1B	148.72	3.27	0.21	15.59	0.00	0.00
ENSSSCG00000037232	SNX29	14.37	3.27	0.66	4.92	0.00	0.00
ENSSSCG00000005997	COL14A1	96.56	3.28	0.26	12.60	0.00	0.00
ENSSSCG00000006071	VPS13B	400.66	3.28	0.13	24.88	0.00	0.00
ENSSSCG00000027926	FTCD	80.75	3.28	0.28	11.68	0.00	0.00
ENSSSCG00000026506	RALGAPA2	641.35	3.28	0.10	31.23	0.00	0.00
ENSSSCG00000010483	PLCE1	373.67	3.28	0.13	24.34	0.00	0.00
ENSSSCG00000031608		7.10	3.28	0.97	3.39	0.00	0.00

ENSSSCG00000039373	SNX24	472.49	3.28	0.12	27.00	0.00	0.00
ENSSSCG00000005585	DENND1A	235.23	3.28	0.17	19.46	0.00	0.00
ENSSSCG00000004621	MYO5C	48.74	3.28	0.37	8.86	0.00	0.00
ENSSSCG00000016857	DAB2	430.27	3.28	0.13	26.01	0.00	0.00
ENSSSCG00000037926		5.40	3.28	1.07	3.08	0.00	0.00
ENSSSCG00000040208	PAG1	685.89	3.28	0.10	31.65	0.00	0.00
ENSSSCG00000011689	PLOD2	6222.08	3.28	0.06	54.39	0.00	0.00
ENSSSCG00000029860	EPB41L4A	996.78	3.28	0.09	37.03	0.00	0.00
ENSSSCG00000015645		417.16	3.29	0.13	25.56	0.00	0.00
ENSSSCG00000015653	IL19	43.32	3.29	0.39	8.36	0.00	0.00
ENSSSCG00000038220	RXRA	508.42	3.29	0.12	28.01	0.00	0.00
ENSSSCG00000015876	CCDC148	45.41	3.30	0.38	8.67	0.00	0.00
ENSSSCG00000040554		25.55	3.30	0.51	6.49	0.00	0.00
ENSSSCG00000037429		1.94	3.30	1.64	2.02	0.04	0.06
ENSSSCG00000005481		123.94	3.30	0.23	14.36	0.00	0.00
ENSSSCG00000036011	ISLR2	16.44	3.30	0.63	5.24	0.00	0.00
ENSSSCG00000025210	ATG10	100.80	3.31	0.26	12.82	0.00	0.00
ENSSSCG00000033382		168.59	3.31	0.21	15.69	0.00	0.00
ENSSSCG00000039837		5.47	3.31	1.18	2.80	0.01	0.01
ENSSSCG00000021956	LY75	5.55	3.32	1.10	3.02	0.00	0.00
ENSSSCG00000014820	FCHSD2	722.70	3.32	0.10	32.30	0.00	0.00
ENSSSCG00000015401		705.66	3.32	0.11	31.59	0.00	0.00
ENSSSCG00000015563	RGL1	371.50	3.32	0.14	24.54	0.00	0.00
ENSSSCG00000015770	VEGFC	70.44	3.33	0.31	10.75	0.00	0.00
ENSSSCG00000004281	KCNQ5	27.79	3.33	0.48	6.98	0.00	0.00

ENSSSCG00000006018	TRPS1	289.80	3.33	0.16	21.38	0.00	0.00
ENSSSCG00000005587	NEK6	1369.42	3.33	0.08	42.83	0.00	0.00
ENSSSCG00000002494	CLMN	247.11	3.33	0.17	19.56	0.00	0.00
ENSSSCG00000009413	CPB2	41.01	3.33	0.40	8.32	0.00	0.00
ENSSSCG00000026425	ADAMTSL2	95.08	3.33	0.26	12.67	0.00	0.00
ENSSSCG00000012102		96.81	3.34	0.53	6.27	0.00	0.00
ENSSSCG00000003773	AK5	56.06	3.34	0.34	9.88	0.00	0.00
ENSSSCG00000011821	ATP13A4	13.11	3.34	0.70	4.77	0.00	0.00
ENSSSCG00000027877	TLR10	3.78	3.34	1.31	2.55	0.01	0.02
ENSSSCG00000039682		2.02	3.34	1.62	2.06	0.04	0.05
ENSSSCG00000009457	DACH1	161.26	3.35	0.20	16.50	0.00	0.00
ENSSSCG00000010739	CTBP2	980.02	3.35	0.09	37.16	0.00	0.00
ENSSSCG00000011600	SLC6A6	3021.88	3.35	0.06	54.35	0.00	0.00
ENSSSCG00000037547	SLC17A3	41.38	3.35	0.40	8.47	0.00	0.00
ENSSSCG00000005480		246.71	3.35	0.17	20.11	0.00	0.00
ENSSSCG00000035849	PTPRF	1226.09	3.36	0.08	39.74	0.00	0.00
ENSSSCG00000012202		624.31	3.36	0.11	30.56	0.00	0.00
ENSSSCG00000007873	ABCC6	66.28	3.36	0.31	10.77	0.00	0.00
ENSSSCG00000032591		26.67	3.37	0.50	6.76	0.00	0.00
ENSSSCG00000029413	DNMT3A	140.84	3.37	0.22	15.37	0.00	0.00
ENSSSCG00000005305	UNC13B	319.46	3.37	0.15	22.78	0.00	0.00
ENSSSCG00000010468	CPEB3	38.03	3.37	0.42	8.12	0.00	0.00
ENSSSCG00000026196		1453.82	3.38	0.18	18.99	0.00	0.00
ENSSSCG00000005225	RFX3	53.45	3.38	0.35	9.61	0.00	0.00
ENSSSCG00000024754	BBS9	80.47	3.38	0.29	11.79	0.00	0.00

ENSSSCG00000011943	BBX	978.14	3.39	0.09	36.35	0.00	0.00
ENSSSCG00000039996	FAM168A	1118.75	3.39	0.10	35.32	0.00	0.00
ENSSSCG00000009208		1283.85	3.39	0.09	39.69	0.00	0.00
ENSSSCG00000008214	FABP1	9.69	3.40	0.82	4.15	0.00	0.00
ENSSSCG00000009293		663.01	3.40	0.11	30.00	0.00	0.00
ENSSSCG00000010655	GFRA1	5.89	3.40	1.07	3.18	0.00	0.00
ENSSSCG00000004896	PHLPP1	11.69	3.40	0.75	4.56	0.00	0.00
ENSSSCG00000038128	CDYL2	237.55	3.40	0.17	19.93	0.00	0.00
ENSSSCG00000001472	SLA-DOA	11.74	3.40	0.75	4.52	0.00	0.00
ENSSSCG00000015894	DPP4	690.15	3.41	0.11	30.44	0.00	0.00
ENSSSCG00000008150	TMEM182	7.78	3.41	0.92	3.69	0.00	0.00
ENSSSCG00000008698	RGS12	103.35	3.41	0.26	13.10	0.00	0.00
ENSSSCG00000033537		436.06	3.41	0.13	26.13	0.00	0.00
ENSSSCG00000039512	RBP5	429.88	3.41	0.24	14.37	0.00	0.00
ENSSSCG00000035621	GABRB1	60.76	3.41	0.34	10.19	0.00	0.00
ENSSSCG00000017781	PIPOX	9.84	3.41	0.85	4.02	0.00	0.00
ENSSSCG00000009172	PPP3CA	327.63	3.42	0.15	23.06	0.00	0.00
ENSSSCG00000010949	DAPK1	101.95	3.42	0.26	13.29	0.00	0.00
ENSSSCG00000022676	OPHN1	60.90	3.42	0.33	10.22	0.00	0.00
ENSSSCG00000011023	ZNF438	21.54	3.42	0.56	6.14	0.00	0.00
ENSSSCG00000006001	ENPP2	329.35	3.42	0.15	23.43	0.00	0.00
ENSSSCG00000021702	XRRA1	43.28	3.43	0.39	8.74	0.00	0.00
ENSSSCG00000038125	NUP210L	163.78	3.43	0.21	16.41	0.00	0.00
ENSSSCG00000039783		21.70	3.43	0.55	6.22	0.00	0.00
ENSSSCG00000009893	TMEM116	69.17	3.43	0.31	11.04	0.00	0.00

ENSSSCG00000004511		2237.35	3.43	0.07	49.73	0.00	0.00
ENSSSCG00000010900	DENND1B	433.41	3.44	0.13	26.50	0.00	0.00
ENSSSCG00000007022	ANK1	23.93	3.44	0.52	6.56	0.00	0.00
ENSSSCG00000004565	CA12	143.57	3.44	0.22	15.74	0.00	0.00
ENSSSCG00000040633	RF00001	2.14	3.45	1.60	2.16	0.03	0.04
ENSSSCG00000030947	ZFAT	63.72	3.45	0.33	10.31	0.00	0.00
ENSSSCG00000014079		77.92	3.45	0.29	11.77	0.00	0.00
ENSSSCG00000037897		4.02	3.45	1.31	2.64	0.01	0.01
ENSSSCG00000005182	CCDC171	8.01	3.45	0.91	3.78	0.00	0.00
ENSSSCG00000006917	LRRC8C	174.93	3.46	0.20	16.89	0.00	0.00
ENSSSCG00000038153	PACRG	2.16	3.46	1.61	2.15	0.03	0.04
ENSSSCG00000001061	JARID2	263.35	3.46	0.16	21.05	0.00	0.00
ENSSSCG00000004736	SPTBN5	36.21	3.46	0.43	8.02	0.00	0.00
ENSSSCG00000040742		8.06	3.46	0.91	3.81	0.00	0.00
ENSSSCG00000017577		28.37	3.46	0.49	7.07	0.00	0.00
ENSSSCG00000005110	SYNE2	2154.76	3.47	0.07	49.63	0.00	0.00
ENSSSCG00000005852	ENTPD8	74.67	3.47	0.31	11.21	0.00	0.00
ENSSSCG00000005944	NDRG1	9628.35	3.47	0.16	21.52	0.00	0.00
ENSSSCG00000004659	SLC12A1	79.33	3.47	0.30	11.51	0.00	0.00
ENSSSCG00000009285		2.18	3.47	1.60	2.16	0.03	0.04
ENSSSCG00000016781	TRIO	2223.46	3.47	0.07	47.26	0.00	0.00
ENSSSCG00000007360	CHD6	558.96	3.48	0.12	29.14	0.00	0.00
ENSSSCG00000040013	MTUS1	910.49	3.48	0.10	35.84	0.00	0.00
ENSSSCG00000031866	TIMP3	9350.61	3.48	0.05	76.63	0.00	0.00
ENSSSCG00000007674	ZAN	229.21	3.48	0.18	19.10	0.00	0.00

ENSSSCG00000008738	CC2D2A	235.73	3.48	0.17	20.06	0.00	0.00
ENSSSCG00000025834	FNDC5	114.73	3.49	0.25	13.97	0.00	0.00
ENSSSCG00000013370		2.21	3.49	1.61	2.16	0.03	0.04
ENSSSCG00000008171	NPAS2	333.17	3.49	0.15	23.04	0.00	0.00
ENSSSCG00000016153	UNC80	12.43	3.50	0.75	4.68	0.00	0.00
ENSSSCG00000008606	OSR1	10.40	3.50	0.83	4.21	0.00	0.00
ENSSSCG00000001004	SLC22A23	427.85	3.50	0.13	26.57	0.00	0.00
ENSSSCG00000027002	KLHL32	6.20	3.50	1.05	3.34	0.00	0.00
ENSSSCG00000003590	PTPRU	516.67	3.50	0.22	16.10	0.00	0.00
ENSSSCG00000032301		5197.55	3.51	0.20	17.38	0.00	0.00
ENSSSCG00000015144	GRAMD1B	403.86	3.51	0.14	25.72	0.00	0.00
ENSSSCG00000034191	SOX6	410.43	3.52	0.14	25.63	0.00	0.00
ENSSSCG00000009200		6.30	3.52	1.06	3.32	0.00	0.00
ENSSSCG00000026863	FARP1	1288.35	3.53	0.08	43.16	0.00	0.00
ENSSSCG00000016061	MYO1B	1847.57	3.53	0.07	49.28	0.00	0.00
ENSSSCG00000013339	ANO3	67.41	3.53	0.32	10.95	0.00	0.00
ENSSSCG00000033845		2.23	3.53	1.59	2.21	0.03	0.04
ENSSSCG00000024417	ERC2	8.45	3.53	0.90	3.91	0.00	0.00
ENSSSCG00000037608	LRRC70	36.01	3.54	0.44	8.07	0.00	0.00
ENSSSCG00000016554	MEST	6526.34	3.54	0.05	65.21	0.00	0.00
ENSSSCG00000005180	BNC2	101.52	3.54	0.26	13.42	0.00	0.00
ENSSSCG00000014083	ANKDD1B	8.49	3.54	0.90	3.91	0.00	0.00
ENSSSCG00000033012		2.27	3.54	1.58	2.23	0.03	0.04
ENSSSCG00000026465	BABAM2	193.67	3.55	0.20	18.13	0.00	0.00
ENSSSCG00000017604	HLF	29.89	3.55	0.49	7.28	0.00	0.00

ENSSSCG00000036499	LMO3	12.80	3.55	0.73	4.85	0.00	0.00
ENSSSCG00000021051	PCDHGA10	2.29	3.56	1.58	2.26	0.02	0.03
ENSSSCG00000014447	SLC6A7	55.72	3.56	0.36	9.89	0.00	0.00
ENSSSCG00000016123	PARD3B	54.09	3.57	0.37	9.73	0.00	0.00
ENSSSCG00000008581	NCOA1	539.93	3.57	0.12	29.91	0.00	0.00
ENSSSCG00000035020	STK32B	34.66	3.57	0.46	7.78	0.00	0.00
ENSSSCG00000037670	TMEM164	173.33	3.57	0.20	17.44	0.00	0.00
ENSSSCG00000009490	DCT	10.77	3.57	0.81	4.41	0.00	0.00
ENSSSCG00000003839		1772.75	3.57	0.08	45.67	0.00	0.00
ENSSSCG00000022741	PDGFRB	598.46	3.57	0.12	30.13	0.00	0.00
ENSSSCG00000031940	GAS2	19.56	3.58	0.61	5.85	0.00	0.00
ENSSSCG00000029744	PLCH2	239.65	3.58	0.18	20.27	0.00	0.00
ENSSSCG00000011485	PTPRG	1440.67	3.58	0.08	44.88	0.00	0.00
ENSSSCG00000039056		630.06	3.59	0.12	30.96	0.00	0.00
ENSSSCG00000032778	PLEKHG1	138.60	3.60	0.23	15.40	0.00	0.00
ENSSSCG00000004180		85.86	3.60	0.29	12.23	0.00	0.00
ENSSSCG00000039161	MEIS1	151.93	3.60	0.22	16.44	0.00	0.00
ENSSSCG00000036520	EFNA5	253.40	3.60	0.17	20.96	0.00	0.00
ENSSSCG00000038649		11.04	3.60	0.80	4.50	0.00	0.00
ENSSSCG00000004209	PTPRK	1049.06	3.60	0.09	39.44	0.00	0.00
ENSSSCG00000035243	RAB27B	119.40	3.60	0.25	14.49	0.00	0.00
ENSSSCG00000014203	MCC	226.17	3.61	0.19	18.75	0.00	0.00
ENSSSCG00000005743	VAV2	570.74	3.62	0.12	30.74	0.00	0.00
ENSSSCG00000039858		8.96	3.63	0.92	3.95	0.00	0.00
ENSSSCG00000016402	AGXT	4.47	3.63	1.28	2.83	0.00	0.01

ENSSSCG00000010683	GRK5	312.68	3.63	0.16	22.84	0.00	0.00
ENSSSCG00000003828	FGGY	97.05	3.64	0.29	12.72	0.00	0.00
ENSSSCG00000017120	SLC6A19	51.95	3.64	0.39	9.42	0.00	0.00
ENSSSCG00000011412	CACNA2D2	136.23	3.64	0.24	14.88	0.00	0.00
ENSSSCG00000022592	FIBIN	74.72	3.64	0.31	11.64	0.00	0.00
ENSSSCG00000000614	GRIN2B	2.43	3.65	1.55	2.35	0.02	0.03
ENSSSCG00000023791	TMEM229B	2.44	3.65	1.55	2.35	0.02	0.03
ENSSSCG00000002451	RIN3	59.60	3.66	0.35	10.32	0.00	0.00
ENSSSCG00000001457	SLA-DQB1	125.88	3.66	0.24	14.95	0.00	0.00
ENSSSCG00000037690		2.46	3.66	1.59	2.30	0.02	0.03
ENSSSCG00000015329	PPP1R9A	253.41	3.67	0.18	20.79	0.00	0.00
ENSSSCG00000004545	ANKDD1A	13.83	3.67	0.73	5.06	0.00	0.00
ENSSSCG00000000778	CPNE8	221.96	3.67	0.19	19.28	0.00	0.00
ENSSSCG00000030016	PDE9A	23.18	3.68	0.56	6.55	0.00	0.00
ENSSSCG00000016244	COL4A4	465.36	3.68	0.13	27.78	0.00	0.00
ENSSSCG00000035568		20.94	3.68	0.59	6.19	0.00	0.00
ENSSSCG00000001100	CARMIL1	810.86	3.69	0.10	35.72	0.00	0.00
ENSSSCG00000001863	TMEM266	4.68	3.70	1.26	2.94	0.00	0.01
ENSSSCG00000009125	ANK2	215.79	3.70	0.19	19.21	0.00	0.00
ENSSSCG00000005981	FBXO32	2805.78	3.70	0.07	51.20	0.00	0.00
ENSSSCG00000018042	SLC47A2	73.06	3.70	0.32	11.44	0.00	0.00
ENSSSCG00000022177	DIS3L2	218.77	3.70	0.19	19.41	0.00	0.00
ENSSSCG00000009831	CUX2	132.16	3.70	0.24	15.25	0.00	0.00
ENSSSCG00000033688	ZDHHC22	9.42	3.70	0.89	4.18	0.00	0.00
ENSSSCG00000009864	MED13L	1970.09	3.71	0.07	49.60	0.00	0.00

ENSSSCG00000037424	CDK19	325.09	3.71	0.16	23.85	0.00	0.00
ENSSSCG00000028759	ASAP1	361.08	3.72	0.15	24.56	0.00	0.00
ENSSSCG00000002412	STON2	90.12	3.72	0.30	12.54	0.00	0.00
ENSSSCG00000005589	NR6A1	168.99	3.72	0.21	17.31	0.00	0.00
ENSSSCG00000026412	PLXNA2	21.42	3.72	0.60	6.26	0.00	0.00
ENSSSCG00000031361	CELSR1	1871.16	3.72	0.08	48.09	0.00	0.00
ENSSSCG00000039531		224.39	3.73	0.20	18.63	0.00	0.00
ENSSSCG00000015270	FMOD	878.11	3.73	0.11	34.46	0.00	0.00
ENSSSCG00000012104	SHROOM2	235.50	3.73	0.19	19.99	0.00	0.00
ENSSSCG00000007539	RBBP8NL	7.26	3.74	1.02	3.67	0.00	0.00
ENSSSCG00000023243	NFIA	113.26	3.74	0.26	14.41	0.00	0.00
ENSSSCG00000023593	AFF1	1529.67	3.74	0.09	42.10	0.00	0.00
ENSSSCG00000012001	ROBO1	1981.90	3.74	0.08	47.31	0.00	0.00
ENSSSCG00000032826	KCTD16	9.68	3.75	0.89	4.19	0.00	0.00
ENSSSCG00000008799	LIMCH1	1120.22	3.76	0.09	40.77	0.00	0.00
ENSSSCG00000025602	DOCK4	355.44	3.77	0.15	24.80	0.00	0.00
ENSSSCG00000011752	FNDC3B	1723.39	3.77	0.08	46.20	0.00	0.00
ENSSSCG00000033875		4.91	3.77	1.24	3.04	0.00	0.00
ENSSSCG00000002474	PPP4R4	113.00	3.77	0.26	14.29	0.00	0.00
ENSSSCG00000039395	GAS2L2	14.82	3.77	0.72	5.27	0.00	0.00
ENSSSCG00000016687		500.56	3.77	0.13	28.86	0.00	0.00
ENSSSCG00000023186	CA4	110.80	3.77	0.26	14.29	0.00	0.00
ENSSSCG00000004791	RASGRP1	343.36	3.78	0.16	24.04	0.00	0.00
ENSSSCG00000004064	ARID1B	334.23	3.78	0.16	24.20	0.00	0.00
ENSSSCG00000010204	BICC1	2171.75	3.78	0.07	54.08	0.00	0.00

ENSSSCG00000036751	PPM1H	1552.34	3.78	0.08	45.43	0.00	0.00
ENSSSCG00000025993	SGPP2	2868.47	3.78	0.13	29.40	0.00	0.00
ENSSSCG00000020248	RF00026	2.67	3.79	1.53	2.47	0.01	0.02
ENSSSCG00000010879	KIF26B	14.87	3.79	0.72	5.25	0.00	0.00
ENSSSCG00000039597	RF02168	27.52	3.79	0.54	7.05	0.00	0.00
ENSSSCG00000039541	ANKRD11	1174.93	3.79	0.23	16.72	0.00	0.00
ENSSSCG00000014169	PCSK1	35.14	3.80	0.48	7.88	0.00	0.00
ENSSSCG00000004919	NEDD4L	1156.87	3.80	0.09	40.73	0.00	0.00
ENSSSCG00000012835		7.54	3.80	1.01	3.78	0.00	0.00
ENSSSCG00000010359		10.08	3.81	0.88	4.34	0.00	0.00
ENSSSCG00000011199	TBC1D5	194.36	3.81	0.20	18.60	0.00	0.00
ENSSSCG00000023437	ITPR1	1144.74	3.81	0.09	40.44	0.00	0.00
ENSSSCG00000015582	PTPN14	1727.18	3.81	0.08	48.40	0.00	0.00
ENSSSCG00000034062	SV2C	12.61	3.81	0.79	4.84	0.00	0.00
ENSSSCG00000014431	AFAP1L1	1.30	3.81	1.83	2.08	0.04	0.05
ENSSSCG00000028974	UST	78.33	3.82	0.32	11.84	0.00	0.00
ENSSSCG00000013294	LDLRAD3	30.58	3.82	0.51	7.46	0.00	0.00
ENSSSCG00000006878	DPYD	206.20	3.82	0.20	19.26	0.00	0.00
ENSSSCG00000002259		595.98	3.82	0.12	31.32	0.00	0.00
ENSSSCG00000024158	ANO1	196.05	3.82	0.20	18.82	0.00	0.00
ENSSSCG00000004490	SETBP1	206.94	3.83	0.20	19.07	0.00	0.00
ENSSSCG00000030560	IGF1R	918.81	3.83	0.10	38.65	0.00	0.00
ENSSSCG00000001909	STRA6	437.56	3.83	0.14	26.63	0.00	0.00
ENSSSCG00000008241	TCF7L1	84.68	3.84	0.31	12.56	0.00	0.00
ENSSSCG00000031487	LSP1	59.26	3.84	0.37	10.31	0.00	0.00

ENSSSCG00000003715	ZNF521	72.40	3.84	0.34	11.34	0.00	0.00
ENSSSCG00000035707		1527.38	3.84	0.18	21.79	0.00	0.00
ENSSSCG00000013335	LGR4	1308.92	3.84	0.09	42.78	0.00	0.00
ENSSSCG00000011906	IGSF11	338.26	3.85	0.16	24.50	0.00	0.00
ENSSSCG00000002283	FUT8	496.86	3.85	0.13	29.24	0.00	0.00
ENSSSCG00000037507	BTBD9	62.27	3.85	0.36	10.61	0.00	0.00
ENSSSCG00000000130	CYTH4	194.30	3.85	0.21	18.69	0.00	0.00
ENSSSCG00000035093	KIF26A	20.81	3.85	0.62	6.23	0.00	0.00
ENSSSCG00000009968	TTC28	1.36	3.86	1.85	2.09	0.04	0.05
ENSSSCG00000031700	SLC2A9	66.08	3.88	0.36	10.90	0.00	0.00
ENSSSCG00000002752	ZFHX3	261.08	3.88	0.18	21.58	0.00	0.00
ENSSSCG00000017753	KSR1	264.20	3.88	0.18	21.86	0.00	0.00
ENSSSCG00000021683		5.36	3.88	1.24	3.14	0.00	0.00
ENSSSCG00000012474	DIAPH2	165.57	3.89	0.23	17.26	0.00	0.00
ENSSSCG00000036000		8.00	3.90	1.01	3.85	0.00	0.00
ENSSSCG00000024392	THEMIS	8.10	3.90	1.02	3.83	0.00	0.00
ENSSSCG00000002429	FOXN3	272.55	3.90	0.18	21.90	0.00	0.00
ENSSSCG00000004434	FRK	380.73	3.90	0.15	25.87	0.00	0.00
ENSSSCG00000008444	PRKCE	83.17	3.91	0.32	12.24	0.00	0.00
ENSSSCG00000010316	KAT6B	492.10	3.91	0.14	28.48	0.00	0.00
ENSSSCG00000037119		18.90	3.91	0.66	5.91	0.00	0.00
ENSSSCG00000002464	PRIMA1	62.27	3.92	0.37	10.58	0.00	0.00
ENSSSCG00000029990	DEFB1	13.54	3.92	0.79	4.99	0.00	0.00
ENSSSCG00000027161	PIP5K1B	2.91	3.93	1.50	2.61	0.01	0.01
ENSSSCG00000005166	MLLT3	210.03	3.93	0.21	19.14	0.00	0.00

ENSSSCG00000011163	DIP2C	287.73	3.94	0.17	22.73	0.00	0.00
ENSSSCG00000031273	IMPG2	2.93	3.94	1.49	2.63	0.01	0.01
ENSSSCG00000040935		1.41	3.94	1.91	2.06	0.04	0.05
ENSSSCG00000006726	SPAG17	107.73	3.95	0.28	14.16	0.00	0.00
ENSSSCG00000008615	VSNL1	63.68	3.95	0.36	10.87	0.00	0.00
ENSSSCG00000005423	ABCA1	969.73	3.95	0.10	38.56	0.00	0.00
ENSSSCG00000035400	YPEL2	957.31	3.96	0.20	19.67	0.00	0.00
ENSSSCG00000012724	SLITRK2	1.43	3.96	1.81	2.19	0.03	0.04
ENSSSCG00000039644	TRPM5	50.07	3.96	0.42	9.50	0.00	0.00
ENSSSCG00000003486	ARHGEF10L	617.88	3.96	0.12	32.21	0.00	0.00
ENSSSCG00000017298	TANC2	565.19	3.96	0.13	31.27	0.00	0.00
ENSSSCG00000014310	CXCL14	139.35	3.96	0.25	16.00	0.00	0.00
ENSSSCG00000016611	CADPS2	50.15	3.96	0.41	9.64	0.00	0.00
ENSSSCG00000025598	COBLL1	114.45	3.97	0.27	14.55	0.00	0.00
ENSSSCG00000013409	SBF2	1127.22	3.97	0.10	41.44	0.00	0.00
ENSSSCG00000023041	PCBP3	199.20	3.97	0.21	18.84	0.00	0.00
ENSSSCG00000029621	BMPR1B	284.31	3.98	0.18	22.66	0.00	0.00
ENSSSCG00000012309	SHROOM4	720.42	3.98	0.11	34.68	0.00	0.00
ENSSSCG00000033829	RIMS2	95.68	3.98	0.30	13.34	0.00	0.00
ENSSSCG00000038194	RF02166	11.27	3.98	0.87	4.57	0.00	0.00
ENSSSCG00000034351	B3GALT1	39.60	3.98	0.47	8.52	0.00	0.00
ENSSSCG00000014725	HBB	1.47	3.99	1.79	2.23	0.03	0.04
ENSSSCG00000033038		1.47	3.99	1.79	2.23	0.03	0.04
ENSSSCG00000017797	SLC6A4	136.15	3.99	0.25	15.80	0.00	0.00
ENSSSCG00000036086	PAX5	1.47	3.99	1.77	2.25	0.02	0.03

ENSSSCG00000011455	CACNA1D	14.19	3.99	0.77	5.20	0.00	0.00
ENSSSCG00000037931		1.47	3.99	1.82	2.19	0.03	0.04
ENSSSCG00000035308		1.48	4.00	1.77	2.26	0.02	0.03
ENSSSCG00000037868	NPBWR2	1.48	4.00	1.77	2.26	0.02	0.03
ENSSSCG00000012607		25.69	4.00	0.58	6.93	0.00	0.00
ENSSSCG00000024520		1.50	4.01	1.80	2.23	0.03	0.04
ENSSSCG00000040591		1.50	4.01	1.80	2.23	0.03	0.04
ENSSSCG00000040164		11.50	4.01	0.86	4.68	0.00	0.00
ENSSSCG00000016331	RAMP1	94.59	4.01	0.30	13.20	0.00	0.00
ENSSSCG00000014351		5.71	4.01	1.22	3.28	0.00	0.00
ENSSSCG00000003603	COL16A1	63.28	4.01	0.37	10.86	0.00	0.00
ENSSSCG00000022649	SLC7A11	577.19	4.02	0.13	31.41	0.00	0.00
ENSSSCG00000026130	EPHA3	1.51	4.02	1.77	2.27	0.02	0.03
ENSSSCG00000023627	LYPD6	51.94	4.02	0.41	9.85	0.00	0.00
ENSSSCG00000024146	MIR1249-1	3.09	4.02	1.48	2.71	0.01	0.01
ENSSSCG00000031193		1.52	4.02	1.79	2.25	0.02	0.03
ENSSSCG00000035300		20.36	4.02	0.66	6.11	0.00	0.00
ENSSSCG00000039952	ZFAND3	938.43	4.03	0.11	38.10	0.00	0.00
ENSSSCG00000000212	AQP6	20.44	4.03	0.66	6.14	0.00	0.00
ENSSSCG00000010604	SH3PXD2A	695.90	4.04	0.12	33.37	0.00	0.00
ENSSSCG00000029865	GRM5	3.14	4.04	1.48	2.73	0.01	0.01
ENSSSCG00000001453	HLA-DRA	35.11	4.04	0.50	8.12	0.00	0.00
ENSSSCG00000032532	CHRM2	94.14	4.05	0.31	13.12	0.00	0.00
ENSSSCG00000007034	PSD3	180.34	4.05	0.23	17.87	0.00	0.00
ENSSSCG00000010651	ABLIM1	853.21	4.05	0.11	37.51	0.00	0.00

ENSSSCG00000040312		1.56	4.06	1.80	2.25	0.02	0.03
ENSSSCG00000024633	JAKMIP3	26.62	4.06	0.58	7.04	0.00	0.00
ENSSSCG00000030359	ARHGEF3	234.48	4.06	0.20	20.22	0.00	0.00
ENSSSCG00000033185	PPP2R3A	678.43	4.06	0.12	33.47	0.00	0.00
ENSSSCG00000033763	GNAQ	478.69	4.06	0.14	28.72	0.00	0.00
ENSSSCG00000015519	RASAL2	306.52	4.06	0.17	23.54	0.00	0.00
ENSSSCG00000027232		9.11	4.08	1.00	4.06	0.00	0.00
ENSSSCG00000035757	SSBP2	256.31	4.09	0.19	20.97	0.00	0.00
ENSSSCG00000000955	TBC1D22A	163.44	4.09	0.23	17.42	0.00	0.00
ENSSSCG00000016656	ELMO1	135.71	4.09	0.64	6.42	0.00	0.00
ENSSSCG00000008642	ASAP2	1301.13	4.10	0.09	45.08	0.00	0.00
ENSSSCG00000013551	C3	20934.25	4.10	0.06	68.95	0.00	0.00
ENSSSCG00000033532	SBK2	24.53	4.11	0.60	6.80	0.00	0.00
ENSSSCG00000026113	ZBTB20	211.86	4.11	0.21	19.48	0.00	0.00
ENSSSCG00000000854		1.60	4.12	1.75	2.35	0.02	0.03
ENSSSCG00000024325	SGK2	188.04	4.12	0.22	18.68	0.00	0.00
ENSSSCG00000002260		49.51	4.12	0.43	9.61	0.00	0.00
ENSSSCG00000009584	SEMA4D	722.53	4.12	0.12	34.42	0.00	0.00
ENSSSCG00000011453	ITIH4	37.20	4.12	0.51	8.05	0.00	0.00
ENSSSCG00000011230	OSBPL10	653.82	4.13	0.12	33.40	0.00	0.00
ENSSSCG00000035038	CGNL1	4942.05	4.13	0.12	35.25	0.00	0.00
ENSSSCG00000009503		43.37	4.13	0.46	8.97	0.00	0.00
ENSSSCG00000028425	UBL4B	1.63	4.14	1.75	2.37	0.02	0.03
ENSSSCG00000005488		37.51	4.14	0.50	8.31	0.00	0.00
ENSSSCG00000029715	OLFM1	31.42	4.14	0.54	7.72	0.00	0.00

ENSSSCG00000024179	NAGS	176.37	4.15	0.23	18.00	0.00	0.00
ENSSSCG00000015565	C1orf21	926.40	4.15	0.11	38.41	0.00	0.00
ENSSSCG00000011880	EAF2	1.65	4.15	1.75	2.37	0.02	0.03
ENSSSCG00000034759		1.66	4.16	1.71	2.43	0.02	0.02
ENSSSCG00000040907		1.67	4.16	1.72	2.42	0.02	0.02
ENSSSCG00000036379		185.12	4.17	0.23	17.92	0.00	0.00
ENSSSCG00000003747	KIAA1328	19.20	4.18	0.70	6.01	0.00	0.00
ENSSSCG00000004551	ZNF609	1272.81	4.18	0.09	44.61	0.00	0.00
ENSSSCG00000017993	NTN1	740.37	4.18	0.22	19.08	0.00	0.00
ENSSSCG00000031985		1.70	4.18	1.75	2.40	0.02	0.02
ENSSSCG00000024152	ETV6	319.21	4.18	0.18	23.74	0.00	0.00
ENSSSCG00000023924	PDE1A	184.24	4.19	0.23	18.43	0.00	0.00
ENSSSCG00000015866	FMNL2	563.87	4.19	0.14	31.00	0.00	0.00
ENSSSCG00000036022		13.05	4.20	0.85	4.93	0.00	0.00
ENSSSCG00000031290	GRIN2A	319.56	4.22	0.18	24.03	0.00	0.00
ENSSSCG00000037579	PPBP	42.96	4.22	0.48	8.87	0.00	0.00
ENSSSCG00000025924	IGFBP5	1046.63	4.22	0.10	41.13	0.00	0.00
ENSSSCG00000002012	CARMIL3	3.53	4.23	1.46	2.89	0.00	0.01
ENSSSCG00000020663	KMT2C	2190.78	4.23	0.08	51.89	0.00	0.00
ENSSSCG00000017274	PITPNC1	3.61	4.23	1.48	2.86	0.00	0.01
ENSSSCG00000037588		6.76	4.25	1.19	3.57	0.00	0.00
ENSSSCG00000009537		1.77	4.26	1.69	2.51	0.01	0.02
ENSSSCG00000040948		1.77	4.26	1.69	2.51	0.01	0.02
ENSSSCG00000012156	CDKL5	57.49	4.26	0.41	10.45	0.00	0.00
ENSSSCG00000005751	COL5A1	4901.83	4.26	0.06	72.92	0.00	0.00

ENSSSCG00000002689	CMIP	1084.83	4.27	0.10	41.80	0.00	0.00
ENSSSCG00000006834	MYBPHL	207.99	4.27	0.23	18.96	0.00	0.00
ENSSSCG00000016746	NPC1L1	709.03	4.27	0.12	34.37	0.00	0.00
ENSSSCG00000035911	P2RY8	3.67	4.27	1.46	2.93	0.00	0.01
ENSSSCG00000003882	SLC5A9	6.88	4.28	1.19	3.60	0.00	0.00
ENSSSCG00000038785	CA7	6.87	4.28	1.19	3.59	0.00	0.00
ENSSSCG00000001456		277.53	4.30	0.19	22.36	0.00	0.00
ENSSSCG00000000802	NELL2	3.70	4.30	1.44	2.98	0.00	0.00
ENSSSCG00000001083		3.71	4.30	1.44	2.98	0.00	0.00
ENSSSCG00000017942		3.75	4.31	1.44	3.00	0.00	0.00
ENSSSCG00000035083		14.10	4.33	0.84	5.16	0.00	0.00
ENSSSCG00000025141	PCDHGB1	3.81	4.33	1.43	3.03	0.00	0.00
ENSSSCG00000006194	NCOA2	844.84	4.34	0.11	37.75	0.00	0.00
ENSSSCG00000000131	ELFN2	93.24	4.34	0.33	13.14	0.00	0.00
ENSSSCG00000002332	SIPA1L1	921.99	4.35	0.11	39.08	0.00	0.00
ENSSSCG00000020522	RF00322	1.89	4.36	1.70	2.57	0.01	0.02
ENSSSCG00000011811	LPP	394.90	4.36	0.17	26.28	0.00	0.00
ENSSSCG00000010272	ADAMTS14	69.14	4.37	0.38	11.36	0.00	0.00
ENSSSCG00000036474	GJB6	91.24	4.37	0.34	12.98	0.00	0.00
ENSSSCG00000034993	NREP	5727.56	4.38	0.06	75.94	0.00	0.00
ENSSSCG00000022829	RF00026	1.93	4.39	1.66	2.64	0.01	0.01
ENSSSCG00000037291	ELAVL4	1.99	4.42	1.65	2.67	0.01	0.01
ENSSSCG00000000875	NR1H4	86.51	4.42	0.35	12.70	0.00	0.00
ENSSSCG00000039553	ASPG	18.89	4.42	0.75	5.93	0.00	0.00
ENSSSCG00000001975	PRKD1	106.62	4.43	0.32	13.88	0.00	0.00

ENSSSCG00000038337		2.02	4.43	1.65	2.68	0.01	0.01
ENSSSCG00000037090	NEU4	2.02	4.44	1.68	2.64	0.01	0.01
ENSSSCG00000007056	PLCB1	765.31	4.44	0.12	35.83	0.00	0.00
ENSSSCG00000023133	OSBPL6	430.47	4.44	0.16	27.55	0.00	0.00
ENSSSCG00000016810	PDZD2	610.57	4.45	0.14	31.73	0.00	0.00
ENSSSCG00000038929	CEMIP	934.64	4.45	0.12	38.42	0.00	0.00
ENSSSCG00000027502	MYL5	2.06	4.46	1.68	2.65	0.01	0.01
ENSSSCG00000011102	NRP1	242.04	4.47	0.21	20.86	0.00	0.00
ENSSSCG00000012000	GBE1	1765.50	4.49	0.09	51.28	0.00	0.00
ENSSSCG00000035557		2.09	4.50	1.63	2.76	0.01	0.01
ENSSSCG00000016639	FOXP2	47.57	4.50	0.48	9.37	0.00	0.00
ENSSSCG00000001787	IL16	309.60	4.50	0.19	23.67	0.00	0.00
ENSSSCG00000004614	UNC13C	19.87	4.50	0.75	5.98	0.00	0.00
ENSSSCG00000016245		214.38	4.50	0.23	19.81	0.00	0.00
ENSSSCG00000022168	APBB2	1188.37	4.50	0.10	43.57	0.00	0.00
ENSSSCG00000032200		4.26	4.50	1.41	3.20	0.00	0.00
ENSSSCG00000008118	PROM2	1058.68	4.50	0.11	41.44	0.00	0.00
ENSSSCG00000011070	MPP7	172.10	4.51	0.25	17.72	0.00	0.00
ENSSSCG00000014726	HBE1	16.04	4.52	0.82	5.49	0.00	0.00
ENSSSCG00000015607	HHAT	88.65	4.52	0.35	12.78	0.00	0.00
ENSSSCG00000005198	KDM4C	465.16	4.53	0.16	28.78	0.00	0.00
ENSSSCG00000013869		44.98	4.55	0.51	9.01	0.00	0.00
ENSSSCG00000008510	LTBP1	1182.75	4.55	0.11	42.76	0.00	0.00
ENSSSCG00000005269	TRPM6	57.71	4.56	0.44	10.32	0.00	0.00
ENSSSCG00000021515	HS3ST1	2.21	4.56	1.63	2.81	0.01	0.01

ENSSSCG00000017084	FAT2	265.41	4.57	0.21	21.42	0.00	0.00
ENSSSCG00000016784	ANKH	298.56	4.57	0.20	22.99	0.00	0.00
ENSSSCG00000034245		4.46	4.57	1.40	3.26	0.00	0.00
ENSSSCG00000033834	NDST4	4.48	4.57	1.40	3.28	0.00	0.00
ENSSSCG00000038162	CCL21	8.37	4.58	1.15	3.97	0.00	0.00
ENSSSCG00000004506	CTIF	184.34	4.58	0.25	18.25	0.00	0.00
ENSSSCG00000000837	CHST11	105.35	4.59	0.33	13.88	0.00	0.00
ENSSSCG00000023520	PCSK5	351.18	4.60	0.18	24.86	0.00	0.00
ENSSSCG00000035424		12.70	4.60	0.94	4.88	0.00	0.00
ENSSSCG00000009029	ARHGAP10	564.89	4.60	0.15	30.75	0.00	0.00
ENSSSCG00000010494	SORBS1	2799.95	4.61	0.07	62.30	0.00	0.00
ENSSSCG00000021586	ZHX2	42.67	4.61	0.52	8.92	0.00	0.00
ENSSSCG00000039745	HMOX1	14562.88	4.61	0.06	77.56	0.00	0.00
ENSSSCG00000031533	PCDHGB6	4.60	4.62	1.39	3.31	0.00	0.00
ENSSSCG00000000555	ITPR2	918.59	4.64	0.25	18.88	0.00	0.00
ENSSSCG00000009197	GRID2	2.34	4.65	1.58	2.94	0.00	0.01
ENSSSCG00000014891	GAB2	8.76	4.65	1.15	4.04	0.00	0.00
ENSSSCG00000009060	MAML3	277.07	4.65	0.21	22.28	0.00	0.00
ENSSSCG00000033650		2.37	4.66	1.59	2.93	0.00	0.01
ENSSSCG00000015701	MGAT5	357.19	4.68	0.19	25.01	0.00	0.00
ENSSSCG00000009564	TMEM255B	17.96	4.69	0.81	5.76	0.00	0.00
ENSSSCG00000039907		2.39	4.70	1.61	2.91	0.00	0.01
ENSSSCG00000033202		9.04	4.71	1.16	4.07	0.00	0.00
ENSSSCG00000034376		2.42	4.71	1.60	2.95	0.00	0.00
ENSSSCG00000014448	ARSI	4.92	4.72	1.37	3.43	0.00	0.00

ENSSSCG00000002632	SLC28A1	124.07	4.72	0.31	15.01	0.00	0.00
ENSSSCG00000021227	HGD	2.47	4.73	1.56	3.04	0.00	0.00
ENSSSCG00000039334		13.91	4.74	0.93	5.07	0.00	0.00
ENSSSCG00000031957		56.24	4.75	0.47	10.05	0.00	0.00
ENSSSCG00000035794		23.68	4.76	0.73	6.54	0.00	0.00
ENSSSCG00000005194	PTPRD	279.95	4.77	0.21	22.23	0.00	0.00
ENSSSCG00000034133		5.16	4.80	1.37	3.49	0.00	0.00
ENSSSCG00000013403	GALNT18	180.18	4.80	0.27	17.90	0.00	0.00
ENSSSCG00000012100		5.23	4.81	1.36	3.53	0.00	0.00
ENSSSCG00000005723	NTNG2	102.36	4.81	0.36	13.50	0.00	0.00
ENSSSCG00000026067	TMPRSS6	137.35	4.82	0.31	15.63	0.00	0.00
ENSSSCG00000001698	CAPN11	54.04	4.82	0.49	9.90	0.00	0.00
ENSSSCG00000024914		188.26	4.83	0.26	18.34	0.00	0.00
ENSSSCG00000000801	TMEM117	124.03	4.83	0.33	14.79	0.00	0.00
ENSSSCG00000024570	KDM4B	1675.74	4.84	0.10	50.23	0.00	0.00
ENSSSCG00000013351	NAV2	899.82	4.84	0.13	38.40	0.00	0.00
ENSSSCG00000035198		24.89	4.84	0.72	6.71	0.00	0.00
ENSSSCG00000016543	EXOC4	175.32	4.85	0.28	17.62	0.00	0.00
ENSSSCG00000033031		5.46	4.87	1.36	3.58	0.00	0.00
ENSSSCG00000011635	TMEM108	2.70	4.88	1.55	3.14	0.00	0.00
ENSSSCG00000016657	AOAH	5.51	4.88	1.36	3.58	0.00	0.00
ENSSSCG00000011103	PARD3	606.50	4.89	0.15	32.02	0.00	0.00
ENSSSCG00000038452	ADAMTS17	170.31	4.89	0.28	17.20	0.00	0.00
ENSSSCG00000013044	FLRT1	2.75	4.90	1.52	3.22	0.00	0.00
ENSSSCG00000023434	PPM1L	214.02	4.91	0.25	19.44	0.00	0.00

ENSSSCG00000012121	EGFL6	41.84	4.92	0.57	8.67	0.00	0.00
ENSSSCG00000010814	ESRRG	26.29	4.92	0.72	6.85	0.00	0.00
ENSSSCG00000006911	TGFBR3	411.67	4.93	0.19	26.37	0.00	0.00
ENSSSCG00000028905	TNIK	1342.08	4.93	0.11	45.93	0.00	0.00
ENSSSCG00000011640	TF	1225.73	4.93	0.11	44.01	0.00	0.00
ENSSSCG00000038811	MOB3B	95.99	4.94	0.38	13.00	0.00	0.00
ENSSSCG00000033118		5.76	4.96	1.37	3.63	0.00	0.00
ENSSSCG00000038890	SMIM31	2.94	4.99	1.53	3.25	0.00	0.00
ENSSSCG00000031287		2.97	5.00	1.50	3.33	0.00	0.00
ENSSSCG00000009228	MAPK10	2.98	5.00	1.50	3.34	0.00	0.00
ENSSSCG00000012034	TIAM1	6.03	5.02	1.35	3.73	0.00	0.00
ENSSSCG00000021764	ARHGEF38	3.10	5.07	1.49	3.40	0.00	0.00
ENSSSCG00000025784	CDH4	17.49	5.08	0.92	5.54	0.00	0.00
ENSSSCG00000015462	TPK1	169.66	5.08	0.30	17.12	0.00	0.00
ENSSSCG00000011030	CUBN	279.36	5.10	0.24	21.66	0.00	0.00
ENSSSCG00000024743	PRR15L	11.94	5.11	1.12	4.56	0.00	0.00
ENSSSCG00000027669	TNS1	388.83	5.11	0.20	25.55	0.00	0.00
ENSSSCG00000016074	ANKRD44	300.99	5.12	0.23	22.35	0.00	0.00
ENSSSCG00000039535	CAMK2B	24.20	5.13	0.79	6.47	0.00	0.00
ENSSSCG00000037206		18.11	5.13	0.91	5.61	0.00	0.00
ENSSSCG00000002653		24.16	5.13	0.79	6.47	0.00	0.00
ENSSSCG00000034689	GPIHBP1	3.25	5.14	1.49	3.46	0.00	0.00
ENSSSCG00000014321	KLHL3	6.58	5.15	1.34	3.85	0.00	0.00
ENSSSCG00000008121		31.41	5.19	0.71	7.35	0.00	0.00
ENSSSCG00000014434	IL17B	3.38	5.19	1.47	3.53	0.00	0.00

ENSSSCG00000005975	MTSS1	987.02	5.22	0.14	38.26	0.00	0.00
ENSSSCG00000026129		25.67	5.22	0.79	6.63	0.00	0.00
ENSSSCG00000028977	DOK6	13.09	5.24	1.11	4.70	0.00	0.00
ENSSSCG00000038499		3.48	5.24	1.49	3.51	0.00	0.00
ENSSSCG00000007909	ABAT	91.58	5.25	0.43	12.33	0.00	0.00
ENSSSCG00000004835	MAGEL2	7.08	5.26	1.32	3.99	0.00	0.00
ENSSSCG00000016609	SLC13A1	3.53	5.26	1.46	3.60	0.00	0.00
ENSSSCG00000001849	ANPEP	2850.01	5.27	0.09	60.25	0.00	0.00
ENSSSCG00000003699	GREB1L	81.30	5.30	0.45	11.66	0.00	0.00
ENSSSCG00000033765	PCDHGA3	3.67	5.30	1.45	3.66	0.00	0.00
ENSSSCG00000037535	SLC34A1	40.97	5.31	0.64	8.31	0.00	0.00
ENSSSCG00000038257	SGCZ	7.66	5.37	1.31	4.09	0.00	0.00
ENSSSCG00000005836	LCN10	3.85	5.37	1.44	3.72	0.00	0.00
ENSSSCG00000039009		28.94	5.39	0.78	6.89	0.00	0.00
ENSSSCG00000000759		3.97	5.42	1.43	3.80	0.00	0.00
ENSSSCG00000015258	GLB1L2	3.99	5.42	1.43	3.78	0.00	0.00
ENSSSCG00000031944		22.59	5.46	0.90	6.05	0.00	0.00
ENSSSCG00000011965	TMEM45A	679.66	5.46	0.17	32.46	0.00	0.00
ENSSSCG00000012585	DCX	30.38	5.47	0.78	7.02	0.00	0.00
ENSSSCG00000005484	ZNF618	108.53	5.50	0.42	13.15	0.00	0.00
ENSSSCG00000037416	CLIC5	38.85	5.50	0.70	7.88	0.00	0.00
ENSSSCG00000033628		4.24	5.52	1.41	3.91	0.00	0.00
ENSSSCG00000011744		8.49	5.52	1.30	4.24	0.00	0.00
ENSSSCG00000005584	CRB2	31.86	5.54	0.78	7.14	0.00	0.00
ENSSSCG00000029419	SCN11A	63.94	5.54	0.55	10.10	0.00	0.00

ENSSSCG00000012709		15.97	5.54	1.10	5.06	0.00	0.00
ENSSSCG00000015632		240.00	5.54	0.29	19.44	0.00	0.00
ENSSSCG00000005240	DOCK8	513.56	5.55	0.20	27.92	0.00	0.00
ENSSSCG00000033800	PELI2	40.60	5.56	0.70	7.96	0.00	0.00
ENSSSCG00000039081		8.76	5.58	1.29	4.31	0.00	0.00
ENSSSCG00000004332	BACH2	81.98	5.58	0.49	11.40	0.00	0.00
ENSSSCG00000003963		8.85	5.59	1.30	4.31	0.00	0.00
ENSSSCG00000038505	MSI2	479.04	5.62	0.21	26.57	0.00	0.00
ENSSSCG00000040746	LRP2	2724.17	5.63	0.09	59.33	0.00	0.00
ENSSSCG00000035125		4.62	5.64	1.39	4.05	0.00	0.00
ENSSSCG00000005457		70.11	5.68	0.55	10.39	0.00	0.00
ENSSSCG00000004402	METTL24	257.02	5.69	0.29	19.73	0.00	0.00
ENSSSCG00000000247	KRT71	4.82	5.71	1.40	4.09	0.00	0.00
ENSSSCG00000005498	PAPPA	27.80	5.76	0.89	6.46	0.00	0.00
ENSSSCG00000000478	GRIP1	55.78	5.76	0.63	9.20	0.00	0.00
ENSSSCG00000001107	SLC17A1	46.62	5.77	0.69	8.39	0.00	0.00
ENSSSCG00000010329	ZMIZ1	610.11	5.78	0.19	29.66	0.00	0.00
ENSSSCG00000029541	PSD2	10.57	5.85	1.28	4.57	0.00	0.00
ENSSSCG00000034248		5.41	5.86	1.39	4.20	0.00	0.00
ENSSSCG00000034454		30.01	5.87	0.88	6.64	0.00	0.00
ENSSSCG00000002011	CPNE6	151.33	5.88	0.40	14.81	0.00	0.00
ENSSSCG00000031830		111.44	5.89	0.46	12.75	0.00	0.00
ENSSSCG00000007858		832.06	5.89	0.18	32.69	0.00	0.00
ENSSSCG00000009504	HS6ST3	40.59	5.89	0.76	7.71	0.00	0.00
ENSSSCG00000040093		10.94	5.90	1.28	4.63	0.00	0.00

ENSSSCG00000021490	PRDM16	82.95	5.92	0.54	10.95	0.00	0.00
ENSSSCG00000038705		5.70	5.94	1.35	4.39	0.00	0.00
ENSSSCG00000004291	NT5E	21.25	5.96	1.08	5.53	0.00	0.00
ENSSSCG00000039025	FREM1	5.82	5.97	1.35	4.41	0.00	0.00
ENSSSCG00000003846	GLIS1	5.80	5.97	1.36	4.40	0.00	0.00
ENSSSCG00000009486	SLITRK6	5.96	6.00	1.35	4.44	0.00	0.00
ENSSSCG00000016401		21.90	6.01	1.08	5.57	0.00	0.00
ENSSSCG00000011497	MAGI1	176.03	6.01	0.38	15.75	0.00	0.00
ENSSSCG00000024777	HDAC4	133.29	6.03	0.44	13.67	0.00	0.00
ENSSSCG00000007857	ACSM3	133.43	6.03	0.44	13.66	0.00	0.00
ENSSSCG00000011641	SLCO2A1	726.82	6.03	0.19	31.21	0.00	0.00
ENSSSCG00000004678	DUOX2	113.34	6.06	0.49	12.41	0.00	0.00
ENSSSCG00000011598	FGD5	160.57	6.07	0.41	14.90	0.00	0.00
ENSSSCG00000032607	ALPL	189.25	6.12	0.39	15.78	0.00	0.00
ENSSSCG00000024621	KAT2B	283.97	6.12	0.31	19.62	0.00	0.00
ENSSSCG00000028805	PTPRM	630.97	6.13	0.21	28.70	0.00	0.00
ENSSSCG00000034773		6.48	6.13	1.35	4.56	0.00	0.00
ENSSSCG00000008101	FBLN7	7.33	6.31	1.32	4.79	0.00	0.00
ENSSSCG00000040397	COLEC12	67.94	6.32	0.67	9.38	0.00	0.00
ENSSSCG00000008196	FAM178B	15.01	6.36	1.25	5.10	0.00	0.00
ENSSSCG00000038035		7.98	6.43	1.31	4.91	0.00	0.00
ENSSSCG00000035371		81.13	6.58	0.67	9.80	0.00	0.00
ENSSSCG00000005278		66.40	6.61	0.75	8.83	0.00	0.00
ENSSSCG00000031142		72.83	6.74	0.75	9.00	0.00	0.00
ENSSSCG00000035396		10.08	6.77	1.28	5.28	0.00	0.00

ENSSSCG00000007005		20.96	6.85	1.25	5.48	0.00	0.00
ENSSSCG00000004646	ATP8B4	21.09	6.86	1.23	5.59	0.00	0.00
ENSSSCG00000021132	PLD5	11.01	6.90	1.28	5.38	0.00	0.00
ENSSSCG00000027300	TNP2	11.39	6.94	1.27	5.46	0.00	0.00
ENSSSCG00000027417	LDLRAD4	190.78	6.97	0.50	14.04	0.00	0.00
ENSSSCG00000006141	CA3	12.75	7.10	1.26	5.63	0.00	0.00
ENSSSCG00000003777	SLC44A5	224.07	7.20	0.49	14.55	0.00	0.00
ENSSSCG00000038221	HSD17B2	59.35	7.45	1.04	7.14	0.00	0.00
ENSSSCG00000002032	SLC7A8	760.09	7.49	0.30	25.11	0.00	0.00
ENSSSCG00000008312	DYSF	416.90	8.69	0.60	14.60	0.00	0.00
